# Atroposelective Bromination for the Synthesis of Chiral Biaryl Phosphines via Cross-Assembled Catalysis with Chiral Phosphoric Acid and Achiral Phenol

**DOI:** 10.1021/jacs.6c06203

**Published:** 2026-06-23

**Authors:** Zhiqian Yu, Hui Yang, Ming Wah Wong, Ying-Yeung Yeung

**Affiliations:** † Department of Chemistry and State Key Laboratory of Synthetic Chemistry, 26451 The Chinese University of Hong Kong, Shatin, NT, Hong Kong 999077, China; ‡ Department of Chemistry, 37580 National University of Singapore, 3 Science Drive 3, Republic of Singapore, 117543

## Abstract

Enantioselective electrophilic halogenation using organocatalysts has emerged as a powerful tool in asymmetric synthesis, yet its application has been largely concentrated on substrates bearing hydrogen bond donors. This prerequisite aligns with the design of conventional bifunctional catalysts, which operate through complementary hydrogen bond interactions with both the substrate and *N*-haloamide reagents. Chiral Buchwald-type ligands are useful monophosphine ligands, but their preparation mainly relies on kinetic resolution. Catalytic atroposelective halogenation of achiral Buchwald-type ligands would be an efficient strategy to prepare valuable *C*_1_-symmetric axially chiral biaryl monophosphine ligands. However, Buchwald-type ligands lack hydrogen bond donors, making them incompatible with typical asymmetric halogenation systems. We hypothesized that an achiral halophenol additive could cross-assemble with a chiral phosphoric acid catalyst in situ, reconfiguring the active site from a electron donor/acceptor to an acceptor/acceptor bifunctional system. This strategy bridges the functional group mismatch between the catalyst and substrate. Herein, we report a catalytic desymmetrization of achiral Buchwald-type ligand derivatives via this cross-assembled catalysis. The resulting axially chiral halogenated ligands are amenable to late-stage diversification, providing facile access to a diverse array of chiral monodentate phosphine ligands for asymmetric catalysis.

## Graphical abstract

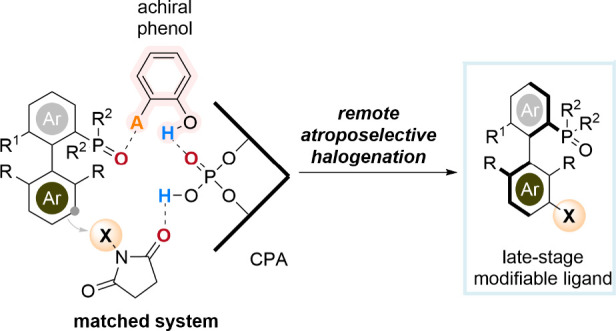

## Introduction

Enantioselective electrophilic halogenation of unsaturated compounds is a versatile method in asymmetric synthesis.^1–6^ These reactions have garnered considerable attention for their dual capability to introduce a halogen atom that facilitates downstream derivatization while concurrently imparting chirality to the molecules. Catalytic asymmetric halogenation using small-molecule organocatalysts represents one of the most important approaches.^7–11^ Significant progress has been made in the development of such reactions with a diverse array of organocatalytic protocols, and the field has received continuous attention.^12–17^ Existing studies have largely concentrated on the asymmetric halogenation of alkenes.^18–24^

This approach could also be applied to the desymmetrization of biaryl compounds to give valuable haloarene building blocks, but relevant reports remain scarce.^25^ To achieve effective asymmetric induction in these reactions, an interaction site on the substrate or intermediate, typically a hydrogen bond (HB)^26^ donor,^27–29^ is generally required to link the substrate with the organocatalyst (Figure 1A).^30–38^ This requirement arises largely from the reliance on stable, commercially available *N*-haloamides as the halogen source, prompting catalyst designs that incorporate functional groups complementary to both the amide moiety of the halogen source and the HB donors of the substrate. Such designs aim to bring the HB donor substrate and the amide-based halogen source into close proximity, thereby achieving a matched catalytic system.^39^ One of the representative catalysts for asymmetric halogenation is the class of chiral phosphoric acids (CPAs).^40–42^ They function as HB donor/acceptor-type bifunctional systems, integrating a defined HB donor (the P–OH group) and HB acceptor (the P=O group) within a rigid, chiral environment.^43–46^ Their utility stems from the ability to form directional interactions that can orient the HB-donor substrates and the *N*-haloamide reagents via complementary HB donors and acceptors. While this strategy is suitable for substrates bearing HB donors, a limitation also emerged whereas substrates without HB donors can become inapplicable.

**Figure 1:**
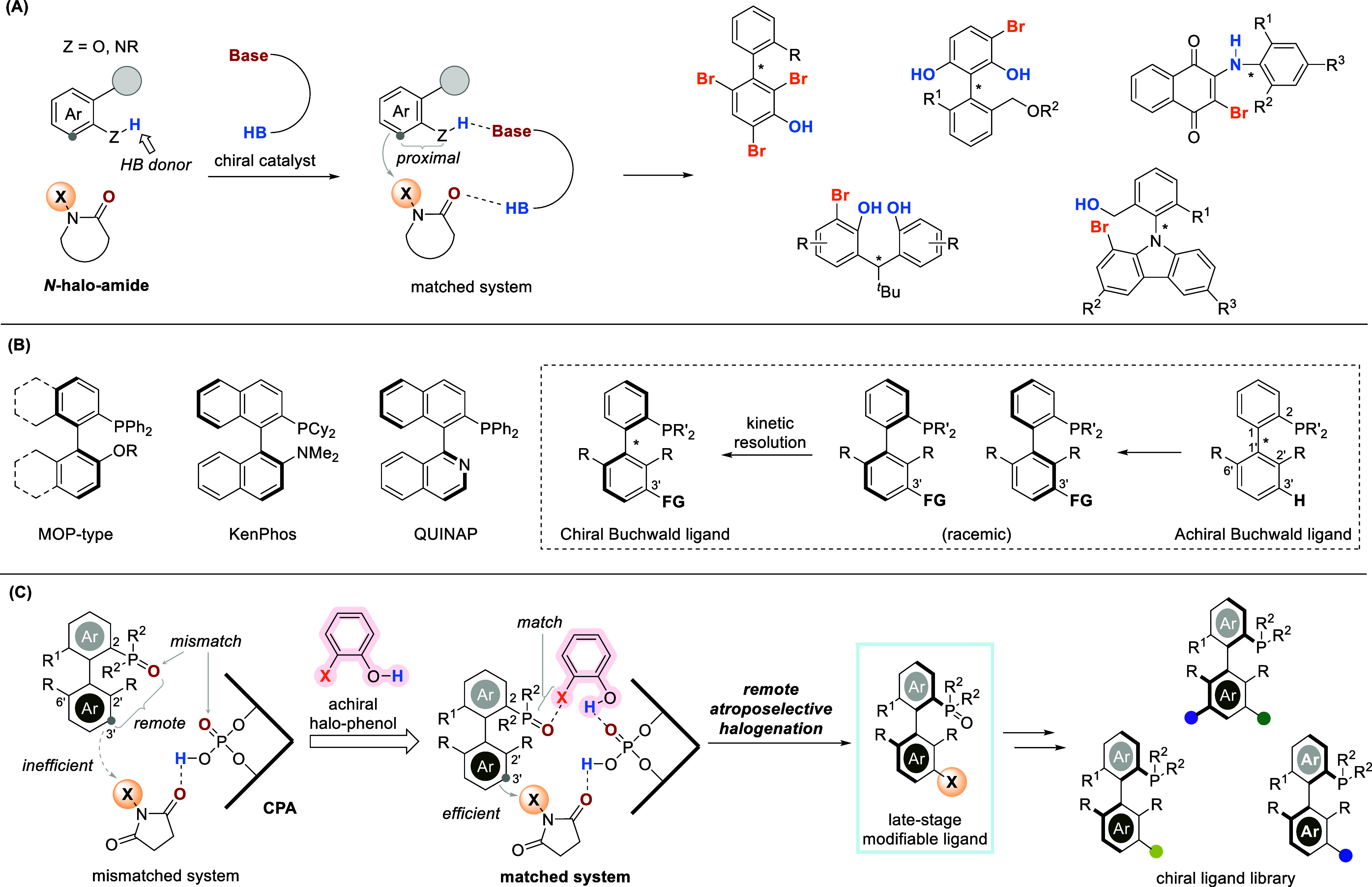
Atroposelective halogenation for the synthesis of chiral biaryl monophosphine ligands. (A) Typical design of organocatalytic asymmetric halogenation with hydrogen bond donor substrates. (B) Examples of axially chiral monophosphine ligands. (C) Our study on using achiral phenols in the cross-assembled catalytic system for remote atroposelective and regioselective monohalogenation and its application in the synthesis of late-stage modifiable monophosphine ligands.

*C*_1_-symmetric axially chiral biaryl monophosphine ligands play a crucial role in transition-metal-catalyzed asymmetric transformations. Examples including MOP-type, KenPhos, QUINAP, and chiral Buchwald-type ligand derivatives are documented (Figure 1B).^47–49^ By independently tuning their steric and electronic properties, these ligands allow precise control over the coordination environment of the metal center, optimizing the catalytic complex’s efficiency at different stages of the reaction cycle. In recent years, the development of new chiral monodentate Buchwald-type ligands has attracted increasing attention because they can be derivatized by incorporating different substituents to cater to various challenging reactions.^50–54^ While many achiral Buchwald-type ligands are commercially available and their analogues are straightforward to synthesize,^55^ preparation of their chiral versions via catalytic methods is synthetically challenging, with only very limited cases reported,^56,57^ leaving kinetic resolution of racemic mixtures as the primary method.^50–54^ Chiral resolutions are not only challenging to optimize resolution conditions for different ligands but also sometimes necessitates the use of stoichiometric amounts of precious metals, rendering it less appealing for the development of new ligand frameworks. Therefore, there is a strong demand for the development of efficient and practical catalytic routes to access structurally diverse chiral biaryl monophosphine ligands.

Achiral Buchwald-type ligands contain symmetrical substituents at the C(2’) and C(6’) positions of the aryl groups. We envisioned that catalytic, atroposelective and regioselective monohalogenation at the C(3′)-H position of these achiral ligands would break their symmetry (Figure 1C). However, achieving this goal presents two major challenges. First, these ligands contain Lewis basic sites rather than HB donors, rendering them mismatches with typical organocatalytic asymmetric halogenation systems that rely on HB donor/acceptor-type bifunctional catalysts. Second, the pre-existing *ortho*-substituents necessitate remote halogenation to break the symmetry of those ligands. These factors preclude the application of conventional asymmetric halogenation strategies, which depend on HB-donor substrates for proximal halogenation (Figure 1A).^30–37^

Multicatalysis, which harnesses the simultaneous action of multiple catalysts, has emerged as a powerful strategy for achieving efficient and selective chemical transformations,^58–60^ as exemplified by enzymes that attain remarkable reactivity and selectivity through the cooperative interplay of active-site residues.^61^ Inspired by this principle and building on our previous experience in cross-assembled catalysis for asymmetric halogenation,^37,62,63^ we hypothesized that a suitable additive could cross-assemble *in situ* with the CPA catalyst, reconfiguring the active site from an electron donor/acceptor to an acceptor/acceptor bifunctional system, thereby bridging the functional group mismatch between CPA and the substrate. To achieve this goal, herein we report a catalyst blend consisting of CPA and an achiral halophenol, which cross-assembled through HB to link initially mismatched catalyst/substrate pairs (Figure 1C). Unlike typical multicatalysis, where catalytic components operate independently, or the literature approach of using acid additives solely to enhance the acidity of CPA,^64–66^ our approach employs cross-assembly of the CPA with an achiral phenol to form a bifunctional catalyst mimic. This mimic brings the substrate and reactant into close proximity. Our strategy enabled reaction optimization primarily through the modification of inexpensive achiral phenol derivatives, circumventing the need for laborious covalent modification of the chiral catalyst. This cross-assembled catalytic system enabled remote desymmetrization of a wide range of biaryl phosphine derivatives, generating axially chiral halo-phosphines that can be readily modified at a late stage to access diverse chiral monodentate Buchwald-type ligands for various asymmetric catalytic applications.

## Results and Discussion

### Reaction Optimization

Our study commenced with the bromination of substrate **S1**, which is the derivative of a representative Buchwald-type ligand SPhos. **CPA-1**–**CPA-4** that are commonly employed to activate *N*-haloamide reagents (e.g., *N*-bromosuccinimide, NBS) were used as the catalysts in our initial experimentations (Scheme 1).^37,67,68^ The Brønsted acidic site of the CPA could form HB with the halogenating reagent’s amide; however, a mismatch between the P=O groups of CPA and substrate **S1** could lead to a mismatched reaction system (Figure 1C). Unsurprisingly, the asymmetric bromination of **S1** with NBS yielded poor enantioselectivity (Scheme 1, entries 1–4), even with CPAs bearing bulky substituents that should have provided adequate catalyst pocket confinement. A number of known HB donor/donor bifunctional catalysts, such as bis-thioureas (**HB-1**, **HB-2**)^69^ and oligopeptides (**HB-3**, **HB-4**)^70^ were also studied, aiming at coordinating to both the P=O and C=O of the substrate and the halogen source. However, the results were found to be unsatisfactory (Scheme 1, entries 5–8).

**Scheme 1:**
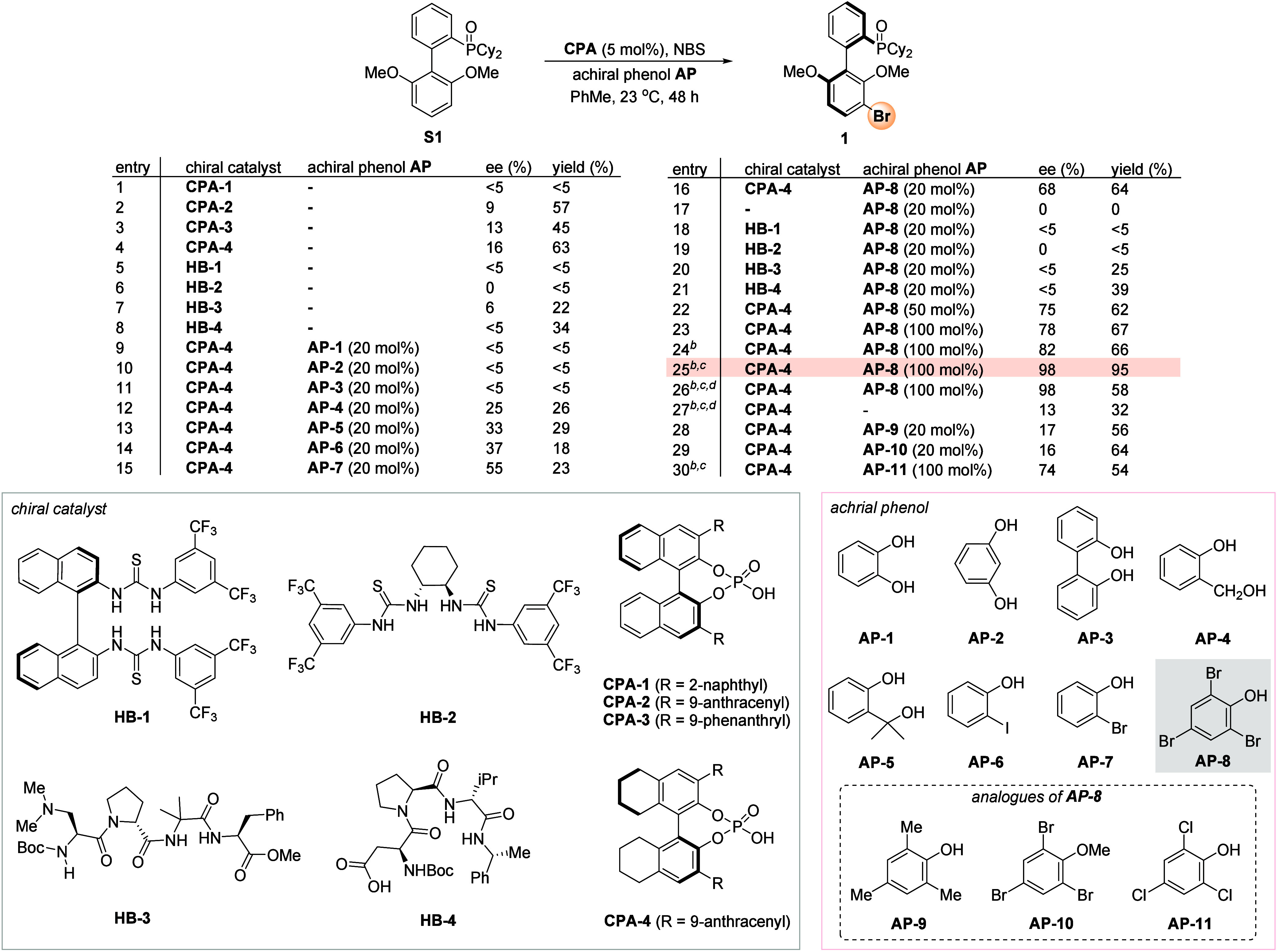
Reaction Optimization^a^

a Reactions were carried out with substrate **S1** (0.05 mmol), chiral catalyst (5 mol %), **AP**, and NBS (1.05 equiv) in toluene (0.05 M) in the absence of light at 23 °C for 48 h. The e.e. was determined by HPLC on a Daicel Chiralpak IC column. NMR yields were given based on **S1** using CH_2_Br_2_ as the internal standard.
b 5 Å molecular sieves (30 mg) were used.
c
**CPA-4** (10 mol %) in toluene (0.025 M) at −20 °C.
d Reaction time was 12 h.

Next, we examined the addition of alcohol additives, and phenol derivatives were chosen because we anticipated that their rigid aryl scaffold could provide better stereocontrol. The introduction of aryl diols, such as catechol (**AP-1**), resorcinol (**AP-2**), and bisphenol **AP-3**, did not improve the enantioselectivity (Scheme 1, entries 9–11). However, using achiral diol **AP-4** (25% ee) and **AP-5** (33% ee) as the phenol additives resulted in a significant enhancement in the asymmetric performance (entries 12–13). Further study revealed that halophenols **AP-6** and **AP-7**, which we believe could serve as halogen bond (XB) donors, gave good ee of the desired product **1** (entries 14–15).^71^ The use of phenolic additives **AP-1**–**AP-7** consistently led to diminished yields compared to the case with **CPA-4** alone, likely due to consumption of the halogen source by the phenols, as evidenced by the detection of brominated phenols in the reactions. To circumvent the undesired bromination side pathway, tribromophenol **AP-8** was employed, and good yield and ee were obtained (entry 16). However, phenol **AP-8** alone showed no catalytic effect (entry 17). In addition, the combination of HB bifunctional catalysts (**HB-1**–**HB-4**) with **AP-8** did not improve the ee (entries 18–21). These results suggested that **CPA-4** and **AP-8** might operate synergistically to enhance enantiocontrol. Therefore, we anticipated that adding more **AP-8** could shift the equilibrium in favor of the **CPA-4**/**AP-8** system, leading to improved enantioselectivity. To our delight, increasing the loading of **AP-8** from 20 to 100 mol % led to a significant improvement in enantioselectivity from 68% to 78% ee (Scheme 1, entries 16, 22–23). To minimize potential disruption of the HB catalytic system by moisture, molecular sieves were added to absorb trace water and the enantioselectivity was slightly improved (entry 24). Further optimization of the reaction by tuning the catalyst loading (10 mol %), concentration (0.025 M), and temperature (−20 °C) led to the excellent enantioselectivity (98% ee) of product **1** (entry 25). Using a shorter reaction time at −20 °C for both the reaction with and without the **AP-8** additive revealed that the additive not only improved enantioselectivity but also accelerated the reaction (entry 26 vs 27 and SI, Figure S3). The analogues of **AP-8,** including **AP-9** (no Br), **AP-10** (with OH masked in the form of OMe), and **AP-11** (with Br replaced by Cl), were studied and their results were inferior (entries 28–30), indicating that both OH and Br in **AP-8** are important in the additive design.

### Substrate Scope

After verifying our concept, the scope of phosphine oxide substrates **S** was systematically investigated using the optimal phenol additive (Scheme 2). Gratifyingly, the reaction demonstrated exceptional substrate generality and functional group tolerance. Substrates bearing bulky phosphine oxide groups, such as cyclohexyl, cyclopentyl, isopropyl, *tert*-butyl, and even 1-adamantyl, delivered products with excellent yields and enantioselectivity (**1**–**5**). Remarkably, product **1** could be obtained on a 2 mmol scale with a 95% yield and 98% ee, while catalyst **CPA-4** was efficiently recovered, highlighting the scalability and practicality of this reaction. Substrates with electron-donating groups like alkyl, phenyl, alkoxy, trifluoromethoxy, benzyl, lactam as well as electron-withdrawing groups like halogens, trifluoromethyl, and cyano positioned at various sites on the phosphonyl arenes, consistently afforded products with good yields and excellent ee (**6**–**26**). Although the substrates bearing electron-withdrawing groups required an extended reaction time, satisfactory yields were still achieved.

**Scheme 2:**
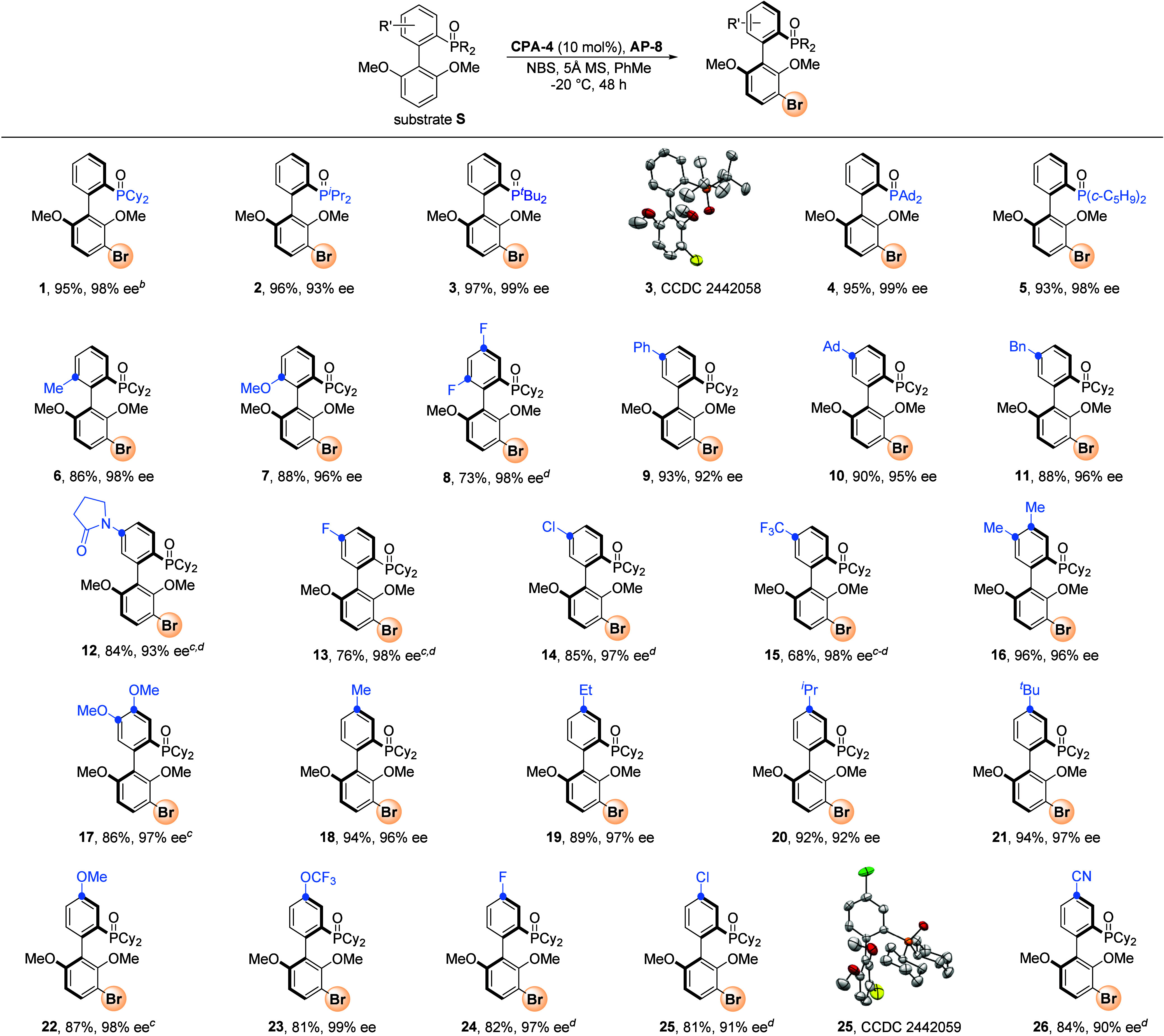
Substrate Scope with Different Phosphine Substituents^a^

a Reactions were carried out with substrate **S** (0.05 mmol), **CPA-4** (0.005 mmol), **AP-8** (0.05 mmol), NBS (1.05 equiv), and 5 Å MS (30 mg) in toluene (0.025 M) in the absence of light at −20 °C for 48 h.
b Two mmol scale with **CPA-4** recovered.
c TBCO/CCl_4_ were used instead of NBS/toluene.
d Five days.

The catalytic protocol was also compatible with various alkyl ether-substituted substrates, even with the sterically demanding isopropyl group, giving **27**–**34** in good-to-excellent yields and ee (Scheme 3). Other than biphenyl scaffold, substrates with cycloalkyl and cycloether were compatible, giving **35**–**37** successfully. Furthermore, indole, naphthalene and phenanthrene frameworks were well-tolerated, affording axially chiral polyaromatic biaryl phosphine oxide compounds with diverse skeletons (**38**–**41**). These compounds are potential ligands for various asymmetric cross-coupling reactions.^72^ The brominated product **1** readily underwent further chlorination, and the resulting dihalogenated compound **42** served as a versatile intermediate, offering multiple handles for efficient derivatization.

**Scheme 3:**
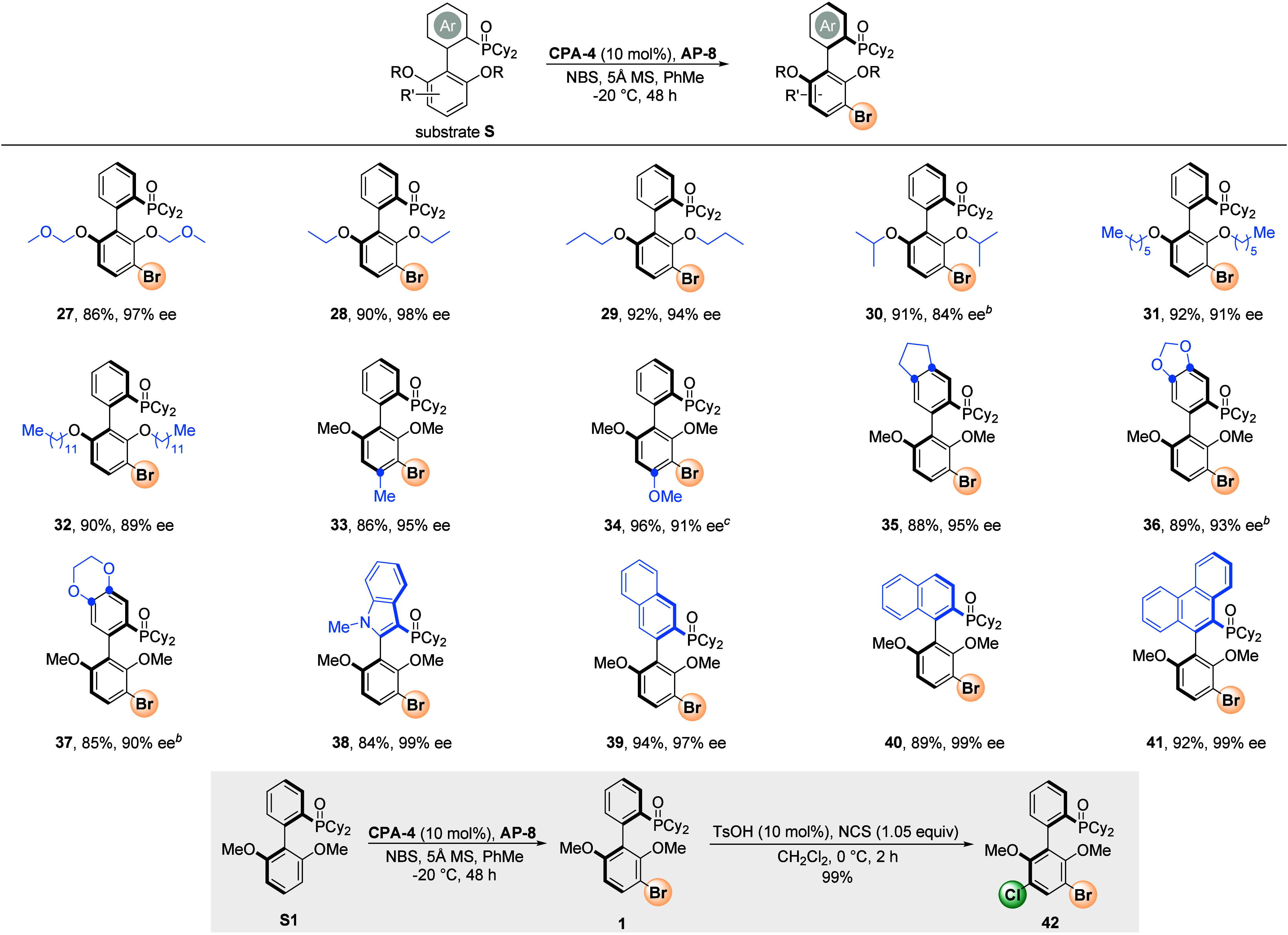
Substrate Scope of Sterically Hindered Substrates and Polycyclic Systems^a^

a Reactions were carried out with substrate **S** (0.05 mmol), **CPA-4** (0.005 mmol), **AP-8** (0.05 mmol), NBS (1.05 equiv), and 5 Å MS (30 mg) in toluene (0.025 M) in the absence of light at −20 °C for 48 h. The e.e. was determined by HPLC.
b TBCO/CCl_4_ were used instead of NBS/toluene.
c –60 °C.

### Preparation and Application of the Ligands

To demonstrate the versatility of the halogenated intermediate and to explore how structural modification of the ligand scaffold could influence catalytic properties, a series of derivatizations was carried out (Scheme 4A). These modifications were designed to systematically vary key ligand parameters, including steric environment, electronic properties, secondary interaction sites, and coordination modes, all of which are known to play important roles in asymmetric transition-metal catalysis. The reduction of the phosphine oxide was efficiently achieved using trichlorosilane in the presence of triethylamine, yielding **(** *R* **)-SPhos-Br** in 82% yield with no erosion of ee.^73^ Subsequently, the bromine in **(** *R* **)-SPhos-Br** could readily be transformed into various valuable phosphine ligands. For instance, carboxylic acid **(** *R* **)-SPhos-CO**_**2**_ **H** and a sulfonate salt **(** *R* **)-SPhos-SO**_**3**_ **Na** were prepared smoothly, both of which were proved to enhance the selectivity in asymmetric cross-coupling reactions through ionic interactions but their literature synthesis relied on separation by chiral resolution.^50–54^

**Scheme 4:**
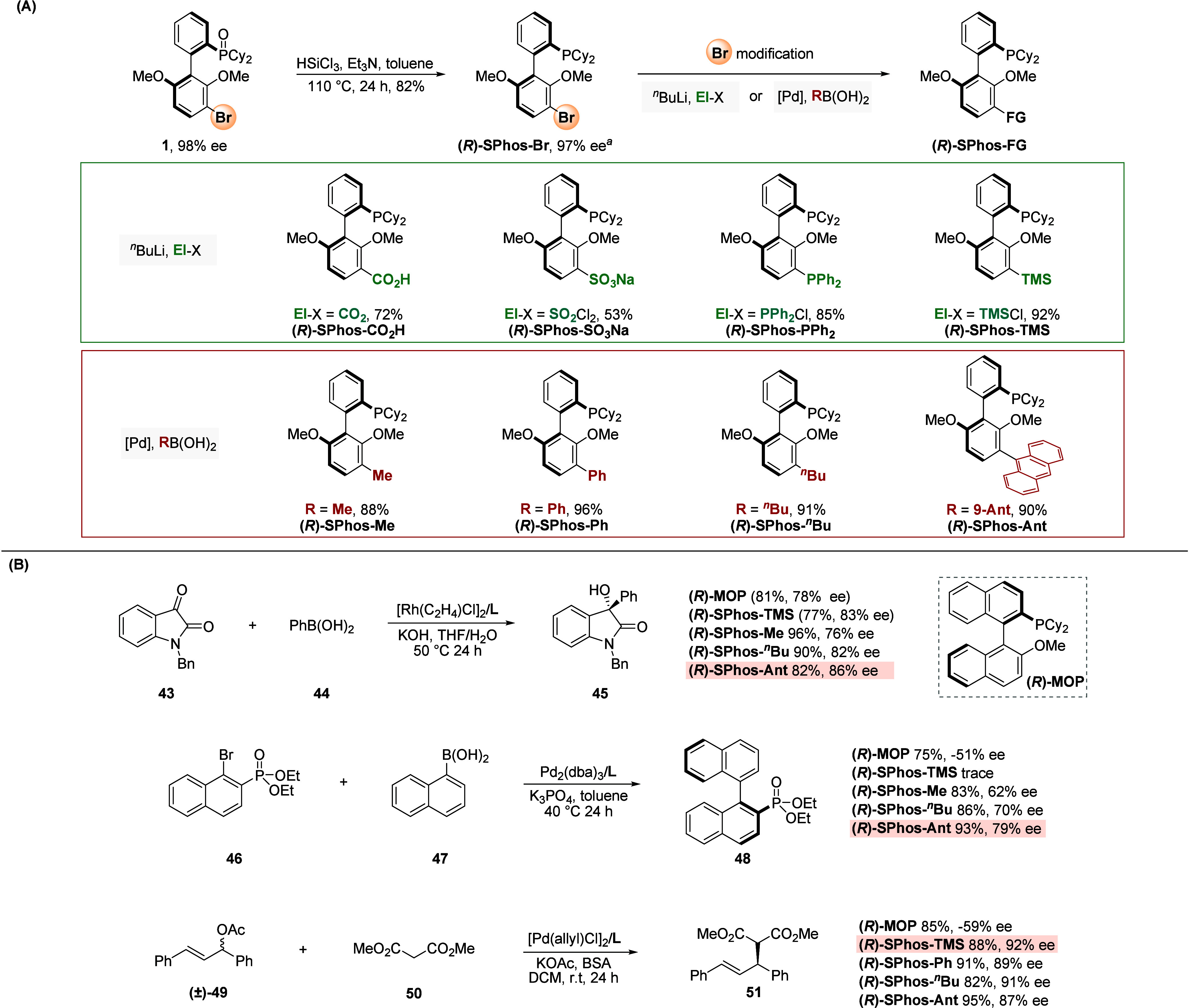
Derivatization and Application of the Ligands: (A) Derivatization of the Chiral Bromo-Biaryl to Monodentate Chiral Phosphine Ligands; (B) Application of the New Monodentate Chiral Phosphine Ligands

a The enantiomeric excess of **(** *R* **)-SPhos-Br** was determined by measuring the ee of its oxidation product, compound **1**.

Furthermore, a series of ligands that were not readily accessible could easily be prepared from **(** *R* **)-SPhos-Br**. For example, the introduction of diphenylphosphine resulted in the synthesis of a structurally unique axially chiral bidentate phosphine ligand, **(** *R* **)-SPhos-PPh**_**2**_. Notably, the steric properties of the functional groups could be flexibly tuned, ranging from small groups such as phenyl **(** *R* **)-SPhos-Ph** and methyl **(** *R* **)-SPhos-Me** to bulkier groups like butyl **(** *R* **)-SPhos-** *^n^* **Bu**, trimethylsilyl **(** *R* **)-SPhos-TMS**, and anthracenyl **(** *R* **)-SPhos-Ant**. This remarkable editability of **(** *R* **)-SPhos-Br** allows the monodentate chiral phosphine ligand to be readily customized to meet the specific demands of various catalytic reactions.

We subsequently carried out investigations to evaluate the performance of some selected new ligands in a series of useful catalytic transformations (Scheme 4B). For comparison, the commercially available and commonly used chiral monodentate phosphine ligand **(** *R* **)-MOP** ^74^ was included as a benchmark. Notably, the anthracene-substituted ligand **(** *R* **)-SPhos-Ant** demonstrated exceptional enantioselectivity in both the addition reaction between isatin **43** and phenylboronic acid **44**, as well as in asymmetric Suzuki-Miyaura coupling reaction between **46** and **47**, affording the corresponding products **45** and **48** with yields of 82% and 93% and ee of 86% and 79%, respectively. In contrast, the trimethylsilyl-containing ligand **(** *R* **)-SPhos-TMS** exhibited outstanding performance in the asymmetric Tsuji-Trost reaction of (±)-**49** with **50**, furnishing **51** in 92% ee. By comparison, the catalytic performance of the commercial monodentate phosphine ligand **(** *R* **)-MOP** was consistently suboptimal across these reactions. These results highlight the value of the newly developed catalytic protocol for producing the halogenated biaryl phosphines. The ease with which this compound can be derivatized into various monodentate phosphine ligands makes it a highly promising platform for developing new enantioselective cross-coupling catalysts.

### Mechanistic Studies

A nonlinear effect study revealed a linear relationship between catalyst and product ee (SI, Figure S2), suggesting that the reaction involves a single catalyst molecule. Kinetic studies were also carried out (SI, Figures S9–S12), revealing that the reaction orders as 0.9 (w.r.t. **CPA-4**), 0.3 (w.r.t. **AP-8**), 0.9 (w.r.t. **S1**), and 0.9 (w.r.t. NBS). The fractional order observed with **AP-8** could arise from its ability to form complexes with other reaction components.

2D ROESY NMR experiments were carried out to get a better understanding of the interactions among the reaction components (Scheme 5 and SI, Figures S4–S6). In the experiment on a mixture of **AP-8** and NBS, ROE between H^a^ and H^b^ was observed. A similar ROE was also observed when using **AP-10** instead of **AP-8**. Since the phenol was masked in the form of methyl ether in **AP-10**, there should be no HB interaction between **AP-10** and NBS. We believe that the ROE between H^a^ and H^b^ could be attributed to the formation of species **A** (with **AP-8**) and **B** (with **AP-10**) via π-π interaction. However, HB appears important because species **A** has a higher binding constant than species **B** (SI, Figures S7 and S8). Further experiment was carried out by adding **CPA-4** to species **A**, and multiple ROE (H^b^-H^a^, H^b^-H^c^, H^b^-H^d^) were observed. These ROE could be attributed to the formation of species **C** via HB and π–π interactions.

**Scheme 5:**
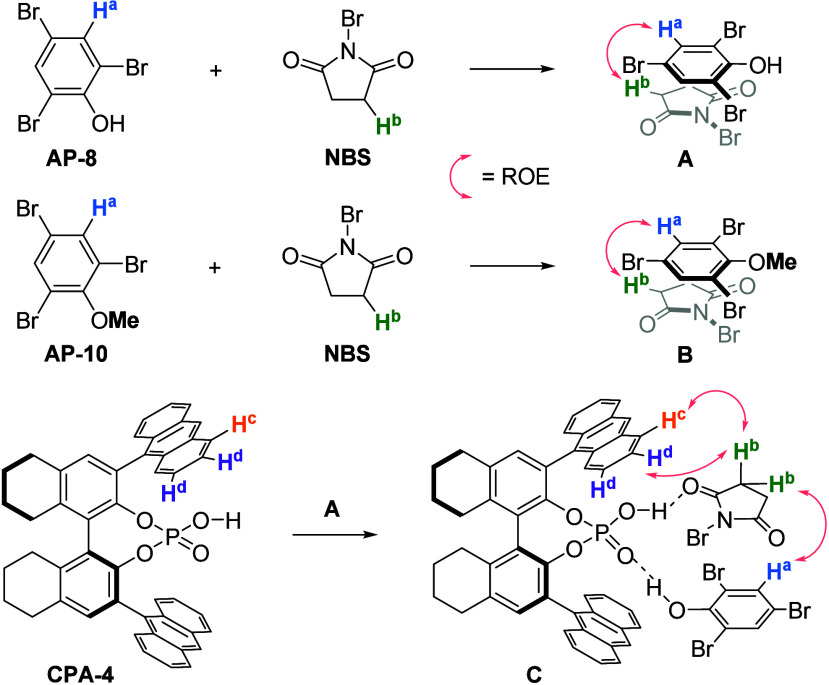
2D ROESY NMR Experiments

### Computational Studies

Based on the catalyst studies in Scheme 1, it appears that **CPA-4** and **AP-8** were both needed and might work synergistically in the reaction. To investigate the reaction mechanism and elucidate the origin of the enantioselectivity observed in the **CPA-4**-catalyzed bromination of biaryl substrate **S1** by NBS in the presence of **AP-8** as the phenol additive, we performed density functional theory (DFT) calculations at SMD­(toluene)/M06-2X-D3/6-311+G­(2d,p)//SMD­(toluene) /M06-2X/6-31G­(d) level of theory (see Supporting Information for details).^75–77^ The calculated reaction profile is presented in Figure 2.

**Figure 2:**
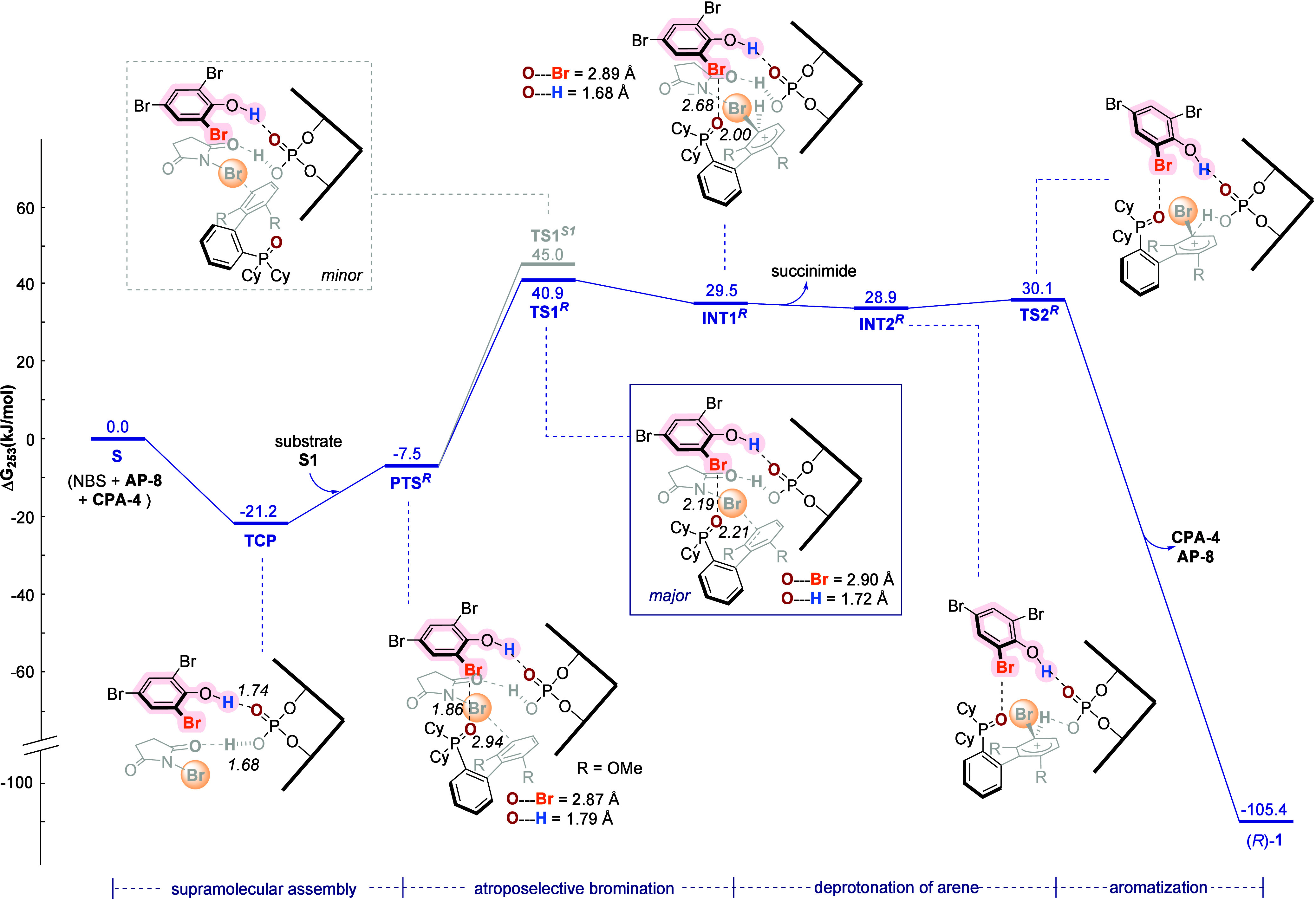
Energy profile for the remote atroposelective halogenation.

Our computational study reveals a thermodynamically stable ternary complex **TCP** (−21.2 kJ/mol) consisting of **CPA-4**, **AP-8**, and NBS, in which the catalytic components are preorganized within a supramolecular scaffold. The ternary complex is stabilized through multiple noncovalent interactions (SI, Figure S13). The computed **TCP** aligns well with the result of species **C** from our NMR experiments (Scheme 5).

Subsequently, the reaction was found to proceed through two elementary steps. First, the ternary complex **TCP** engages with the substrate **S1** to give the pretransition state complex **PTS** *^R^* (toward the formation of the major product (*R*)-**1**) stabilized by HB, XB, and π-π stacking (SI, Figure S14). The HB between phosphoric acid and NBS enhances the electrophilicity of the bromine atom, facilitating the Br transfer to the substrate to give intermediate **INT1** *^R^* via transition state **TS1** *^R^*, while NBS is simultaneously converted into succinimide in the process. The observed regiospecific bromination at the carbon atoms ortho to the methoxy groups of substrate **S1** can thus be rationalized by the site-specific nature of XB interactions (SI, Figure S17). Second, the generated succinimide, no longer tightly bound, dissociates from **INT1** *^R^* to give intermediate **INT2** *^R^*. This is followed by a fast transfer of the vicinal C–H proton to the phosphate of **CPA-4** via **TS2** *^R^*, affording the desired brominated product (*R*)-**1**. The proton transfer and aromatization step exhibits a negligible free-energy barrier (1.2 kJ/mol), suggesting that this step is kinetically insignificant and likely governed by diffusion rather than intrinsic activation energy.

Overall, the reaction is highly exergonic and nonreversible, with a calculated reaction energy of – 105.4 kJ/mol. Based on our mechanistic investigation, we concluded that the rate and enantio-determining steps are the atroposelective bromination transition state **TS1** *^R^*, having a low activation barrier of 62.1 kJ/mol. To rationalize the origin of enantiodifferentiation, we first explored various possible conformations of the (*R*)-**1**-forming transition states by analyzing the relevant rotatable bonds (SI, Table S1), and the lowest-energy structure **TS1** *^R^* was identified. Next, three low-energy transition states for the formation of the minor product (*S*)-**1** were located (Figure 3), with **TS1** *^S1^* being the most stable. The calculated free energy difference between the major **TS1** *^R^* and minor **TS1** *^S1^* is 4.1 kJ/mol, which is in good agreement with the experimental ee of 98% (Scheme 1, entry 25). We have also calculated the transition state in the absence of **AP-8**. The calculated activation barrier was found to be much higher compared with **TS1** *^R^*, suggesting the important role of **AP-8** in the atroposelective bromination (SI, Figure S15).

**Figure 3:**
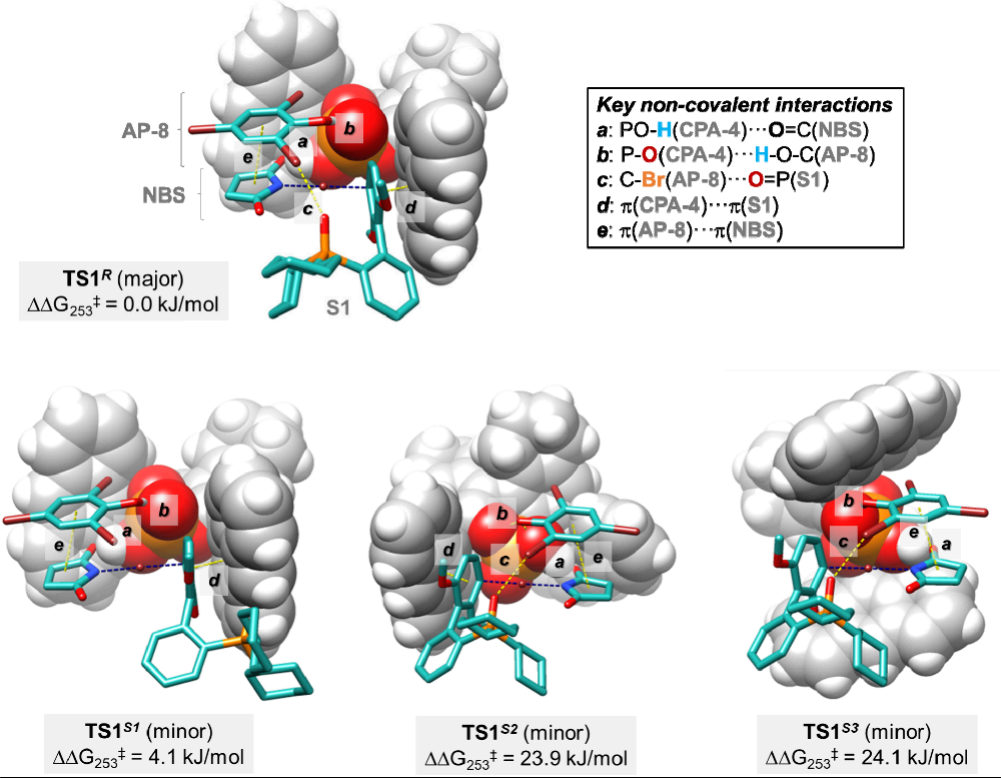
Comparative analysis of low-lying transition states.

We next analyzed the key noncovalent interactions in the major **TS1** *^R^* and the role of the achiral phenol **AP-8** in the stereocontrolling transition states (Figure 3). It appears that various electrostatic interactions might be involved in the transition states, which consist of multiple components. In the following discussion, we have selected several key interactions for analysis.

The acidic proton of **CPA-4** engages in a HB interaction with the carbonyl group of NBS (interaction *a*), enhancing the Br’s electrophilicity. In addition, **CPA-4**’s P=O moiety forms a HB with the hydroxyl group of **AP-8** (interaction *b*), which modulates the confinement of the catalyst pocket and alters the catalyst’s active site from a HB acceptor (P=O) to a XB donor (C–Br). A XB interaction between a bromine atom in **AP-8** and the P=O moiety of **S1** (interaction *c*) was also identified. Since the original Lewis basic oxygen in **CPA-4** is incapable of such interaction with **S1**, this underscores the critical role of the phenol **AP-8** in dynamically editing the catalytic site to accommodate the substrate. Moreover, two π–π stacking interactions were found to be involved in the stabilization of **TS1** *^R^*: (1) anthracenyl­(**CPA-4**)-dimethoxyphenyl­(**S1**) in interaction *d*; (2) phenol­(**AP-8**) and succinimide­(**NBS**) in interaction *e*.

Three *S*-forming transition states (**TS1** *^S1^*, **TS1** *^S2^*, and **TS1** *^S3^*) were identified, with **TS1** *^S1^* calculated as the lowest in energy. In contrast to the *R*-forming **TS1** *^R^*, the minor **TS1** *^S1^* lacks XB interaction *c*, highlighting the critical role of the XB in governing stereoselectivity. Furthermore, neither of the other two *S*-forming transition states exhibits the full network of noncovalent interactions observed in **TS1** *^R^*: in **TS1** ^***S2***^, the HB (interaction *b*) forms between **AP-8**’s OH and the neutral oxygen atom instead of the negatively charged oxygen atom of **CPA-4**; and in **TS1** ^***S3***^, the π–π stacking interaction *d* is missing (Figure 3).

Noncovalent interaction (NCI) analysis was performed to validate the key interactions. The stacking interactions are evident from the green discs between aromatic rings, while the HB interaction *b* appears particularly strong, producing a distinct blue disc (Figure 4A). While the XB interaction *c* is missing in **TS1** *^S1^*, a C–H···O HB is observed between the anthracenyl moiety of **CPA-4** and the P=O moiety of the substrate (SI, Figure S16).

**Figure 4:**
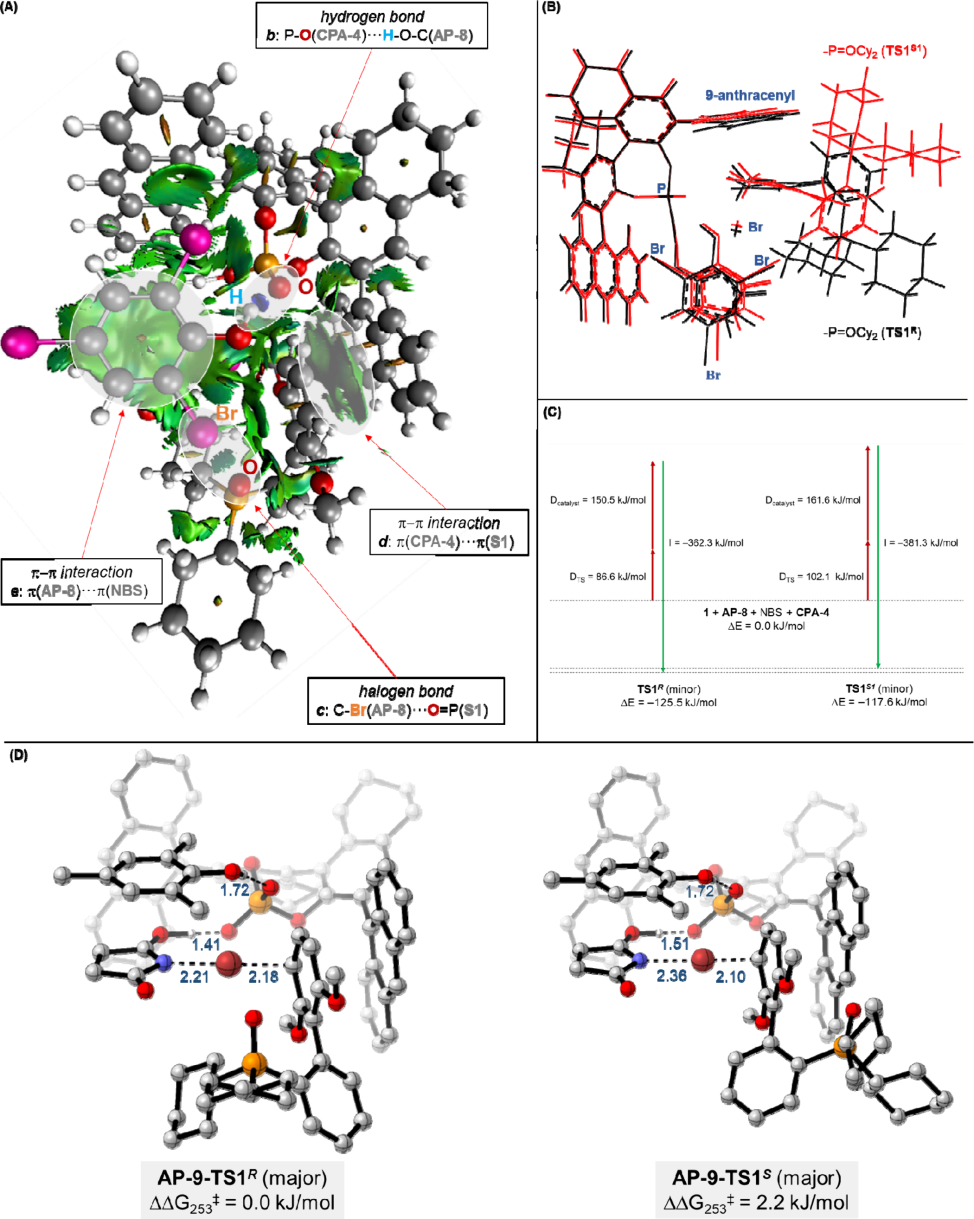
Analysis of the transition states. (A) The noncovalent interactions in the key transition states. (B) Overlaying the major and minor TS. (C) Distortion–interaction analysis. (D) Study on the **TS1** with **AP-9**.

Overlay of the major **TS1** *^R^* (black color) and minor **TS1** *^S1^* (red color) transition states shows that the O=PCy_2_ moiety bends significantly away from the 9-anthracenyl moiety in **TS1** *^S1^*, an indication of steric repulsion between the two (Figure 4B). This interpretation is further supported by distortion-interaction analysis (Figure 4C).^78^ The energy decomposition reveals that the overall activation barrier arises from two main components: (i) the distortion energy (D_catalyst_) required to deform the catalytic components **CPA-4**/**AP-8**, and the substrate/NBS pair from their equilibrium geometries to the transition-state conformations (D_TS_); (ii) the interaction energy (I) released upon their association at the transition state. The result shows a lowering of both the distortion and interaction energies of the more favorable transition state **TS1** *^R^* when compared to **TS1** *^S1^*. This is likely due to the extra stabilization from the additional XB in **TS1** *^R^* that shifts the transition state to an earlier point along the reaction coordinate, akin to the use of a better nucleophile in S_N_2 reactions as discussed in the literature.^76^ Thus, the energy difference between the major (**TS1** *^R^*) and minor (**TS1** *^S1^*) transition states appears to arise from the favorable XB in **TS1** *^R^* and the unfavorable steric interaction in **TS1** *^S1^*, partially offset by the stabilizing C–H···O HB.

We also studied the transition states involving the less efficient phenol **AP-9** (Figure 4D), which lacks bromine atoms and thus cannot participate in XB interaction *c* showing in Figure 3. Consistent with the experimental result, the calculated activation free energy barrier between the major and minor transition states (**AP-9-TS1** *^R^* and **AP-9-TS1** *^S1^*) for **AP-9** is relatively small (ΔΔG^‡^_253_ = 2.2 kJ/mol), which is lower than the difference calculated for **AP-8**.

## Conclusion

In summary, we have developed a cross-assembled catalytic system for the atroposelective electrophilic halogenation of Buchwald-type ligands. The chiral phosphoric acid catalyst and achiral halophenol additive cross-assembled in situ, dynamically modifying the catalytic site to achieve a good match between the originally mismatched catalyst/substrate pair, thereby circumventing the need for laborious synthesis of new chiral catalyst variants. The utility of this protocol was demonstrated through the facile synthesis of a diverse library of chiral Buchwald-type ligands. We anticipate that this cross-assembled catalysis platform will offer a practical and tunable route to chiral Buchwald-type ligands and accelerate the development of enantioselective transition-metal catalysis.

## Supplementary Material



## Notes

Experimental procedures, computational details, and characterization data for the compounds (PDF)
