# Supplementary material for: Atroposelective Bromination for the Synthesis of Chiral Biaryl Phosphines via Cross-Assembled Catalysis with Chiral Phosphoric Acid and Achiral Phenol
Source: J Am Chem Soc. 2026 Jun 23;148(26):27550–61. doi: 10.1021/jacs.6c06203 (PMC13352631; doi:10.1021/jacs.6c06203)
Supplement: Supplementary file 1 [file ja6c06203_si_001.pdf]

Atroposelective Bromination for the Synthesis of Chiral Biaryl Phosphines via Cross-Assembled Catalysis with Chiral Phosphoric Acid and Achiral Phenol

Zhiqian Yu,[1] Hui Yang,[2] Ming Wah Wong,[2]* Ying-Yeung Yeung[1]*

[1]Department of Chemistry and State Key Laboratory of Synthetic Chemistry, The Chinese University of Hong Kong, China

[2]Department of Chemistry, National University of Singapore, 3 Science Drive 3, Republic of Singapore, 117543

*Corresponding author emails: chmwmw@nus.edu.sg, yyyeung@cuhk.edu.hk

Table of Contents

I. General information S2
II. Substrate list S3
III. Mechanistic Studies S4
IV. Computational studies S14
V. Procedures for the preparation of substrates S22
VI. General procedure for the atroposelective remote-bromination S28
VII. Procedures for product diversification S29
VIII. Procedures for application of the new ligands in asymmetric catalysis S32
IX Experimental data for the described substances S34
X. X-Ray crystallographic data S83
XI. Supplementary references S105
XII. Copies of NMR spectra S107
XIII. Cartesian coordinates S213

I. General information

All reactions that required anhydrous conditions were carried out using standard procedures under a nitrogen atmosphere. All commercially available chemicals were used as received unless stated otherwise. Yields given refer to chromatographically purified compounds unless stated otherwise. Halogen sources were purified prior to use. The solvents were dried by the solvent purification system (Inert PS-MD-7). ^{1}H NMR and ^{13}C NMR spectra were recorded on a Bruker ADVANCE III (400 MHz) or Bruker AMX500 (500 MHz) spectrometer. Chemical shifts (δ) are reported in ppm using residual solvent protons (^{1}H NMR, δH = 7.26 ppm for $CDCl_3$, ^{13}C NMR, δC = 77.16 ppm for $CDCl_3$) as internal standard unless specified. High-resolution mass spectra were obtained on a Thermo Finnigan MAT 95 XL spectrometer. X-ray crystallography data were collected on a Bruker AXS Kappa ApexII Duo Diffractometer. Optical rotations were measured by Rudolph Autolol II polarimeter at 589 nm, 25 ºC. Analytical thin-layer chromatography (TLC) was performed with Merck pre-coated TLC plates, silica gel 60F-254, layer thickness 0.25 mm. Flash chromatography separations were performed on Merck 60 (0.040-0.063 mm) mesh silica gel.

II. Substrate list

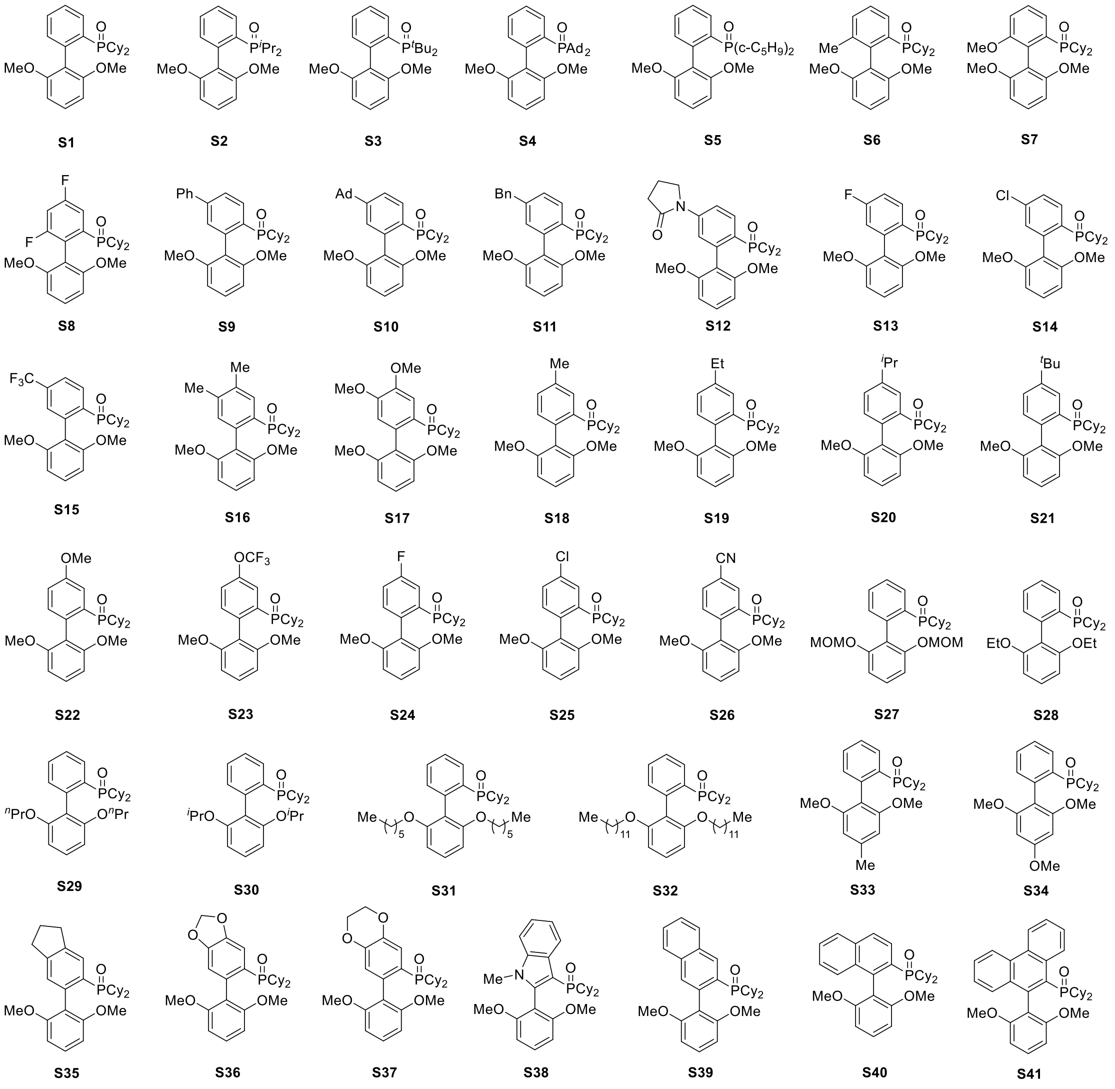

Figure S1. Substrates of biarylphosphine oxides in the manuscript

III. Mechanistic Studies

(1) Non-linear effect study

To a solution of phosphine oxides (0.05 mmol), catalyst **CPA-4** (0.005 mmol), **AP-8** (0.05 mmol), and 5Å MS in toluene (2 mL) was added NBS (0.05 mmol) at -20 °C in the absence of light. The resultant mixture was stirred for 48 h and quenched with saturated aqueous Na_2SO_3 solution (2 mL). The solution was extracted with EtOAc (3 × 5 mL). The combined organic layer was dried over anhydrous Na_2SO_4 and concentrated under reduced pressure. The residue was subjected to flash column chromatography (hexane/EtOAc 1:1 – $CHCl_3$/EtOAc 1:3) to afford the target product **1**. The e.e. was determined by HPLC on a Daicel Chiralpak IC column. The linear relationship between the e.e. of the catalyst and product suggests that the reaction should involve a single-molecule catalyst.

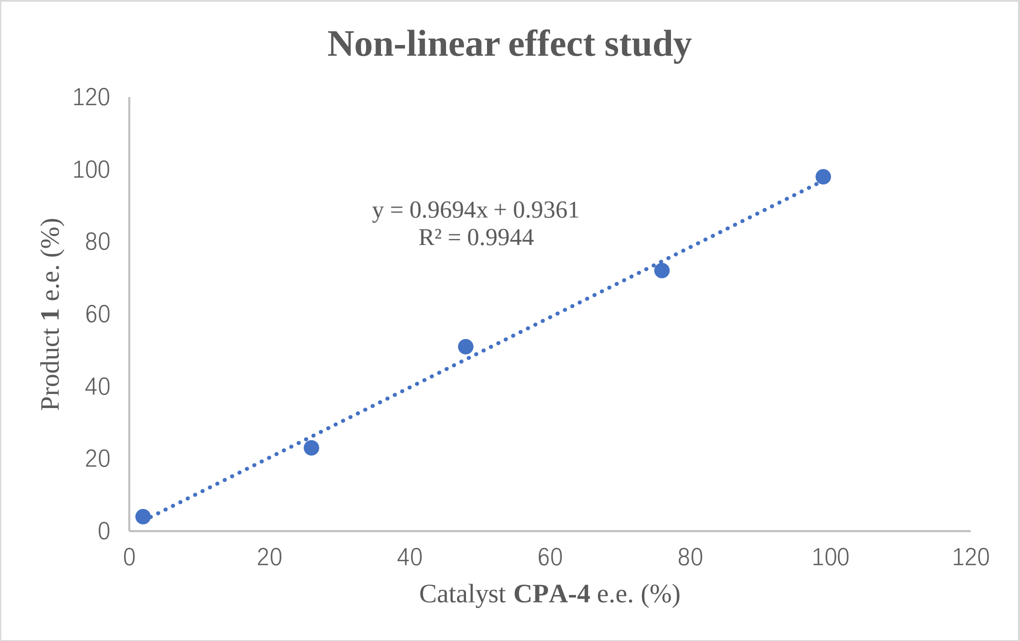

Catalyst e.e. (%)	2	26	48	76	99
Product e.e. (%)	4	23	51	72	98

Figure S2. Non-linear effect study

(2) Time-yield-plot

To a solution of phosphine oxides (0.05 mmol), catalyst **CPA-4** (0.005 mmol), **AP-8** (none or 0.05 mmol), and 5Å MS in toluene (2 mL) was added NBS (0.05 mmol) at -20 °C in the absence of light. The resultant mixture was stirred for 48 h. During the reaction, samples were taken at the indicated times and quenched with saturated aqueous Na_2SO_3 solution (2 mL), followed by measurement of yields using NMR with CH_2Br_2 as the internal standard.

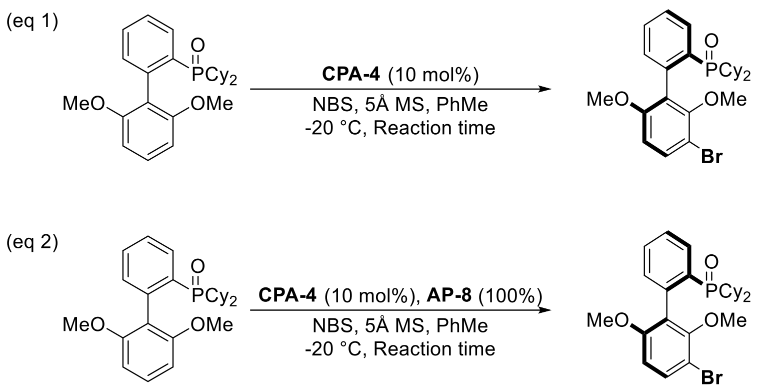

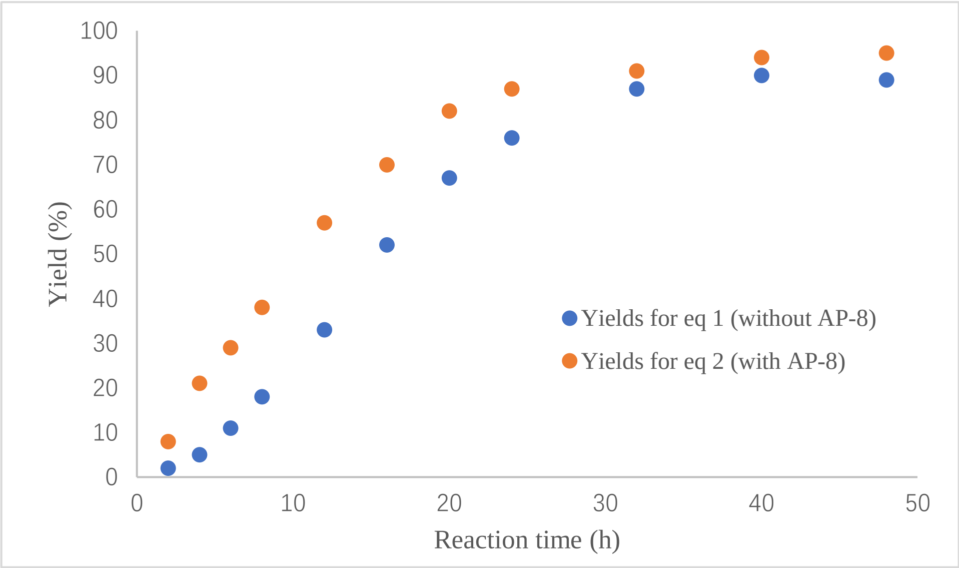

Figure S3. Time-yield-plot

(3) 2D NMR experiments

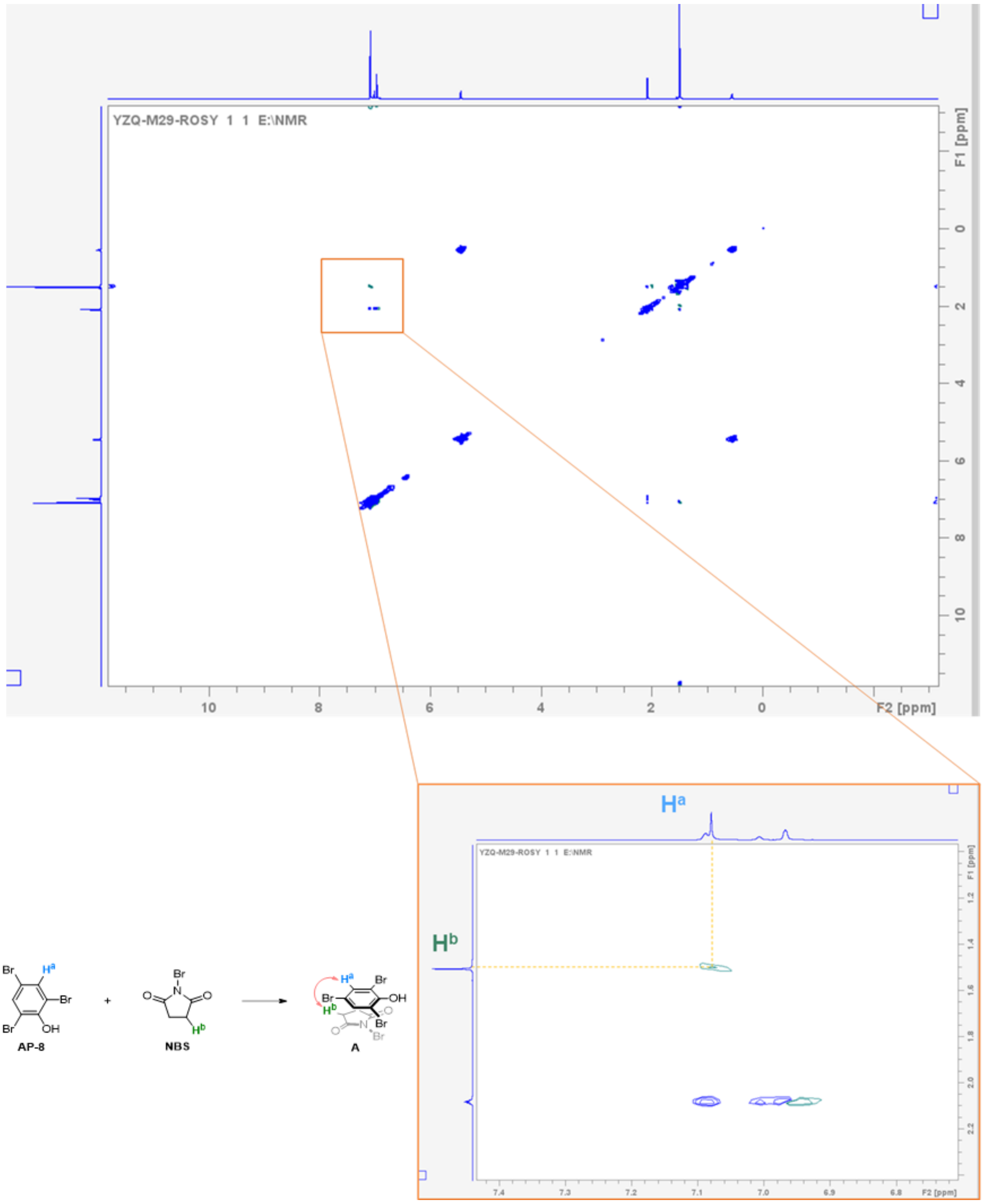

Figure S4. 2D ROESY NMR on a mixture of **AP-8** and NBS.

Note: 2D ROESY NMR (mixing time = 200 ms) experiment on a mixture of **AP-8** (0.01 mmol) and NBS (0.01 mmol) in d_8-toluene was performed.

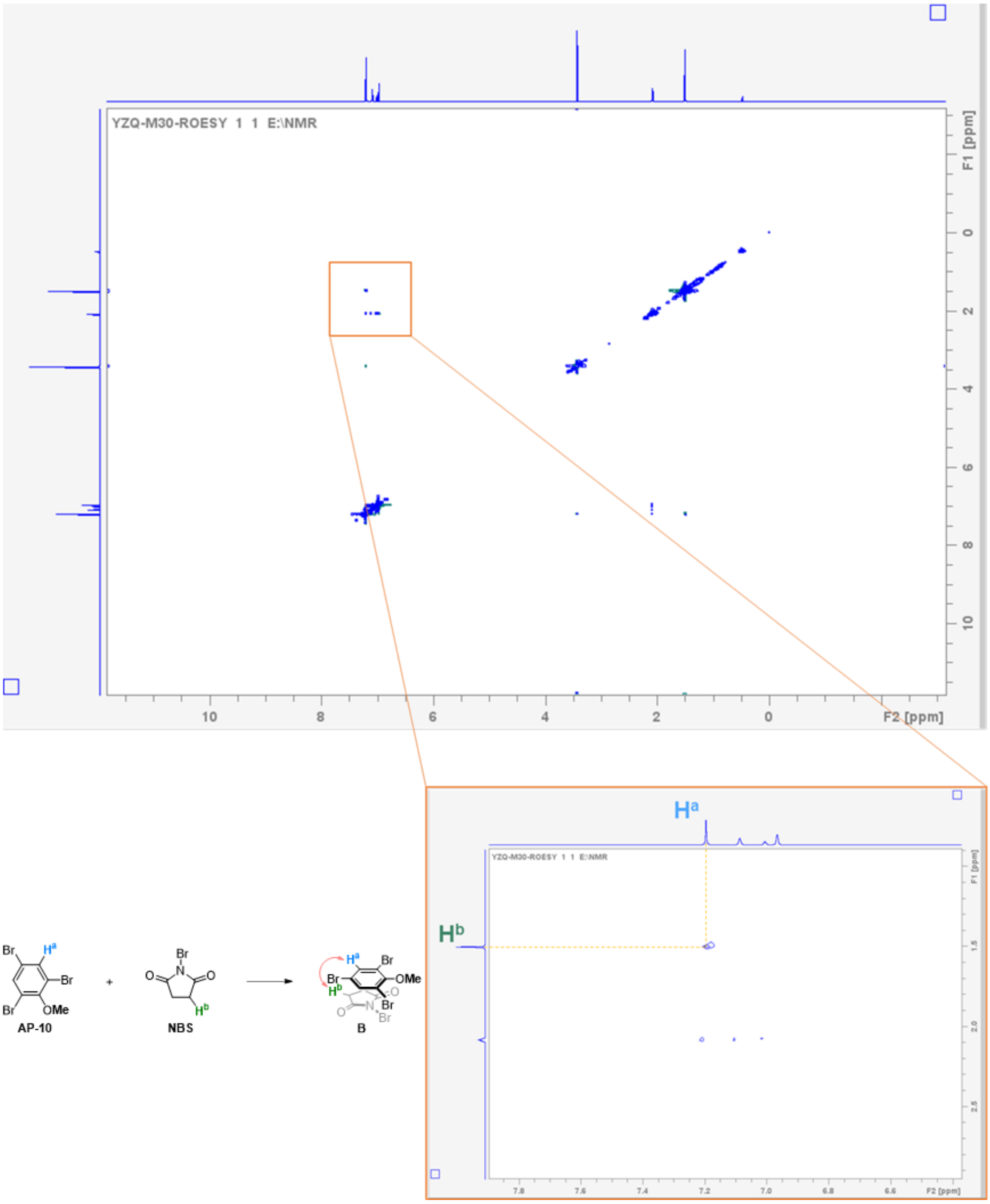

Figure S5. 2D ROESY NMR on a mixture of **AP-10** and NBS.

Note: 2D ROESY NMR (mixing time = 200 ms) experiment on a mixture of **AP-10** (0.01 mmol) and NBS (0.01 mmol) in d_8-toluene was performed.

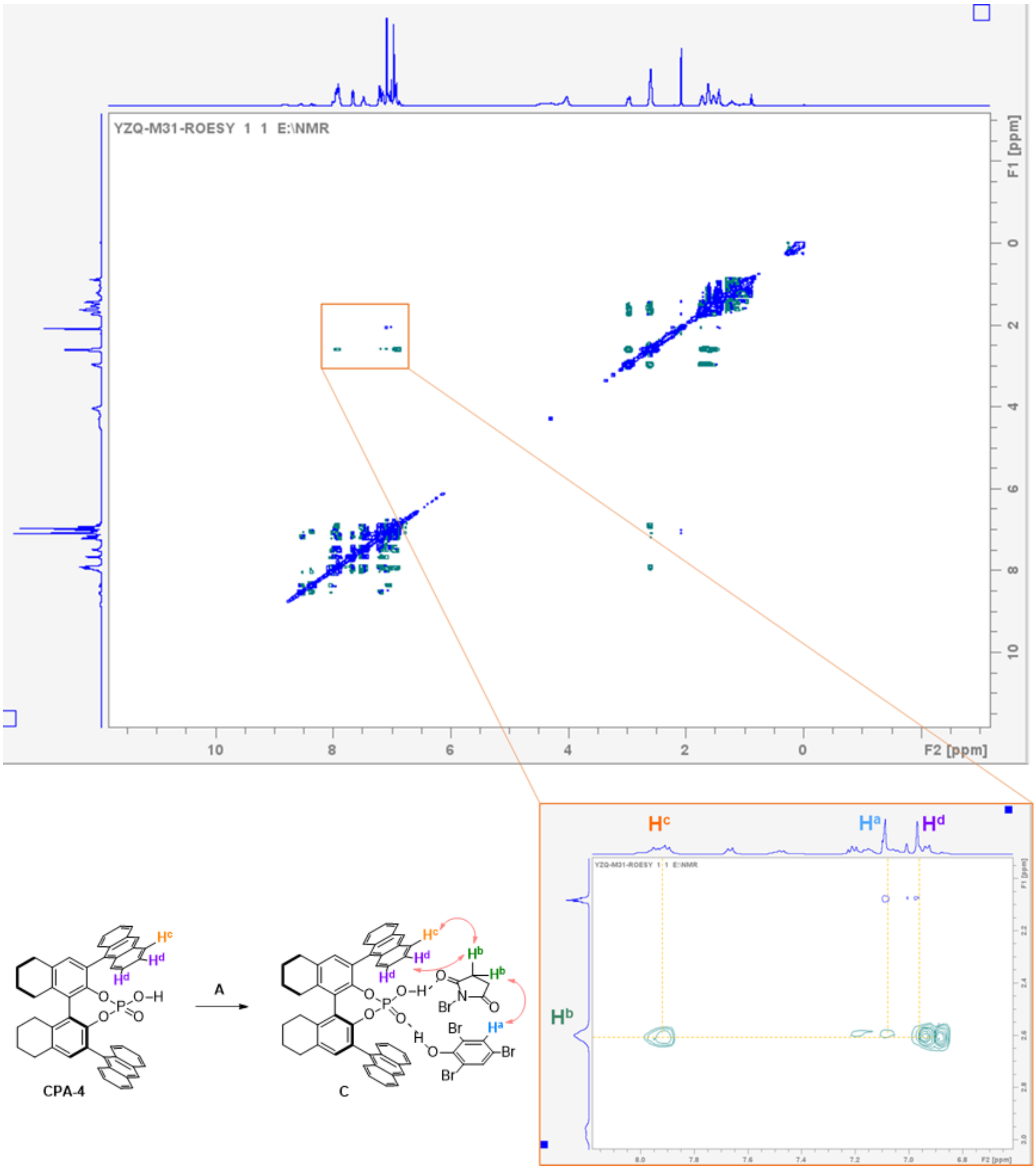

Figure S6. 2D ROESY NMR on a mixture of **AP-8**, NBS, and **CPA-4**.

Note: based on the sample from the experiment shown in Figure **S4**, another 2D ROESY NMR (mixing time = 200 ms) experiment was performed by adding **CPA-4** (0.01 mmol) to species **A** in d_8-toluene.

(4) ^{1}H NMR titration experiments

^{1}H NMR titration experiments were carried out on a mixture of **AP-8** or **AP-10** (0.02 mmol) and NBS in toluene-*d8* (0.5 mL). The measured chemical shifts were plotted against the equivalents of **AP-8** or **AP-10**, and the resulting curves were fitted using software in http://supramolecular.org.

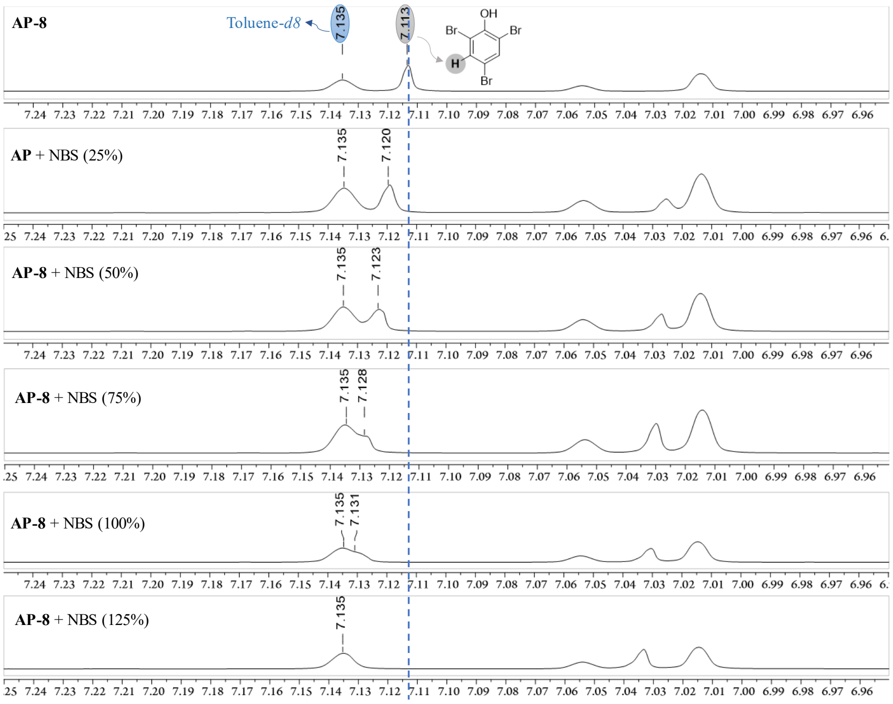

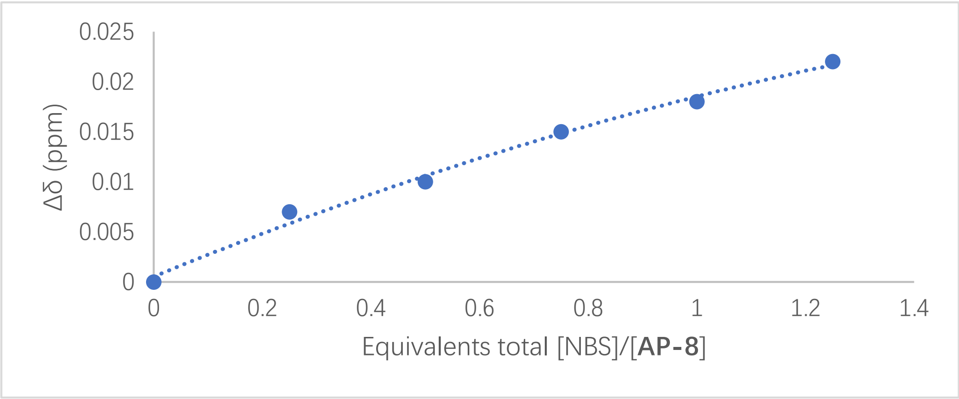

NBS	δ (ppm)	Δδ (ppm)
0	7.113	0
25%	7.120	0.007
50%	7.123	0.010
75%	7.128	0.015
100%	7.131	0.018
125%	7.135	0.022

Figure S7. ^{1}H NMR titration experiments on a mixture of **AP-8** and NBS**.** For the calculations of the binding constant (*K*), a 1:1 binding was assumed. The binding constant (*K*) was found to be 23.55 M^{-1} ± 0.16.

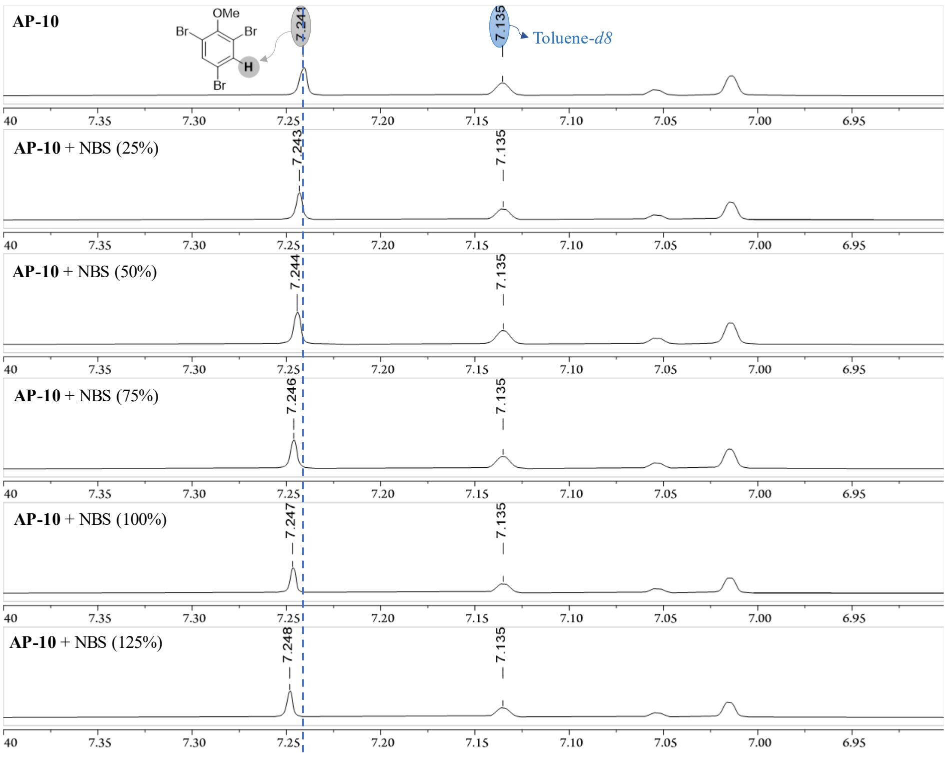

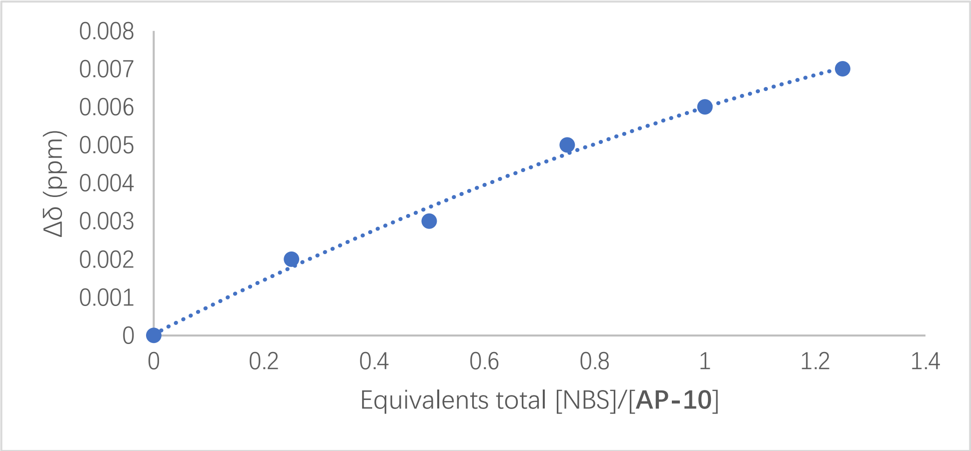

NBS	δ (ppm)	Δδ (ppm)
0	7.241	0
25%	7.243	0.002
50%	7.244	0.003
75%	7.246	0.005
100%	7.247	0.006
125%	7.248	0.007

Figure S8. ^{1}H NMR titration experiments on a mixture of **AP-10** and NBS. For the calculations of the binding constant (*K*), a 1:1 binding was assumed. The binding constant (*K*) was found to be 16.85 $M^{-1} \pm 0.13$.

(5) Kinetic studies

Reaction orders were studied using the initial rate method. Components including substrate **S1**, NBS, catalyst **CPA-4,** and **AP-8** were used in the study. The reactions were carried out at a 0.05 mmol scale, and the amount of one of the components was varied according to the values indicated in Figure **S9-S12**. The amount of product was measured after 2 h in each of the reactions.

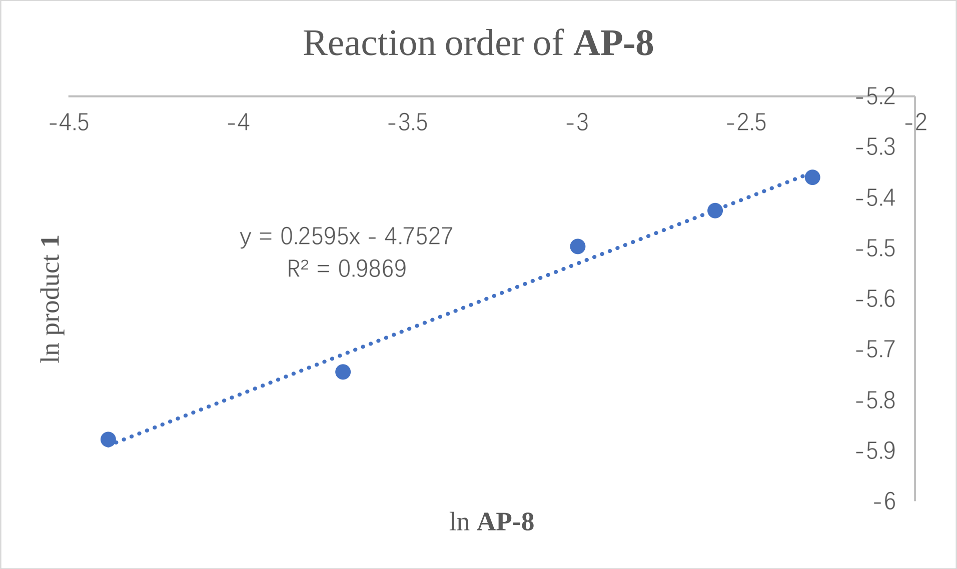

**AP-8** (mmol)	ln **(AP-8)**	**1** (mmol)	ln (**1**)
0.0125	-4.382	0.0028	-5.8781
0.025	-3.689	0.0032	-5.7446
0.05	-2.996	0.0041	-5.4968
0.075	-2.5903	0.0044	-5.4262
0.1	-2.3026	0.0047	-5.3602

Figure S9. Reaction order of **AP-8**

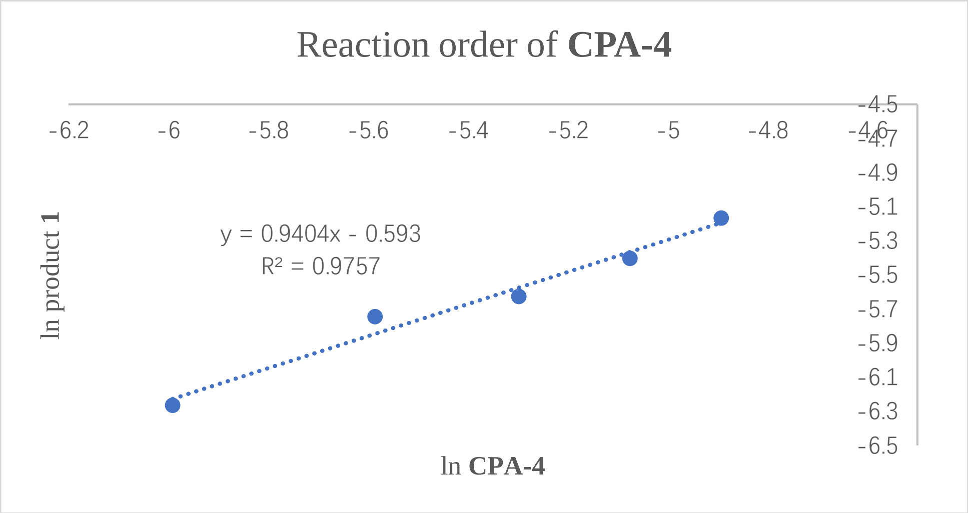

**CPA-4** (mmol)	ln (**CPA-4**)	**1** (mmol)	ln (**1**)
0.0025	-5.9915	0.0019	-6.2659
0.00375	-5.5861	0.0032	-5.7446
0.005	-5.2983	0.0036	-5.6268
0.00625	-5.0752	0.0045	-5.4037
0.0075	-4.8929	0.0057	-5.1673

Figure S10. Reaction order of **CPA-4**

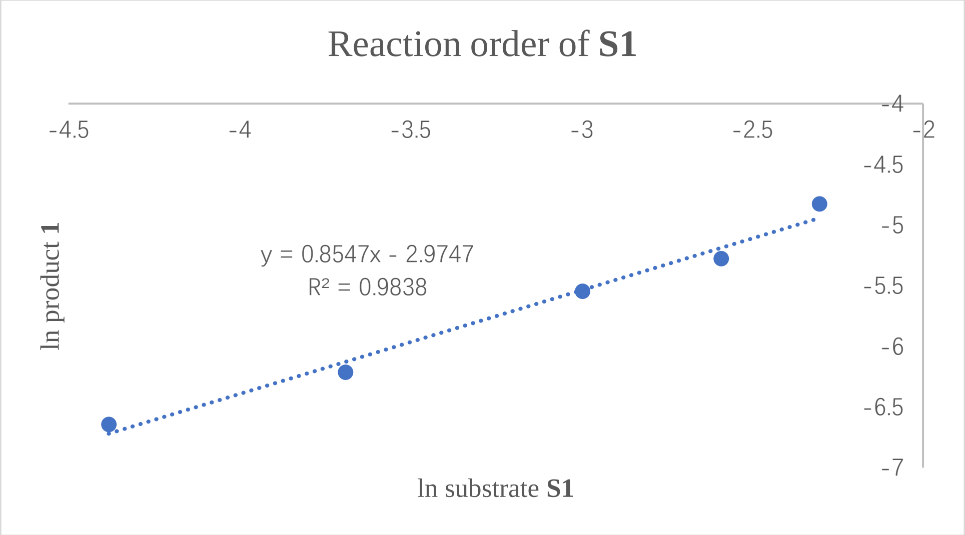

**S1** (mmol)	ln (**S1**)	**1** (mmol)	ln (**1**)
0.0125	-4.382	0.0013	-6.6454
0.025	-3.689	0.002	-6.2146
0.05	-2.996	0.0039	-5.5468
0.075	-2.5903	0.0051	-5.2785
0.1	-2.3026	0.008	-4.8283

Figure S11. Reaction order of **S1**

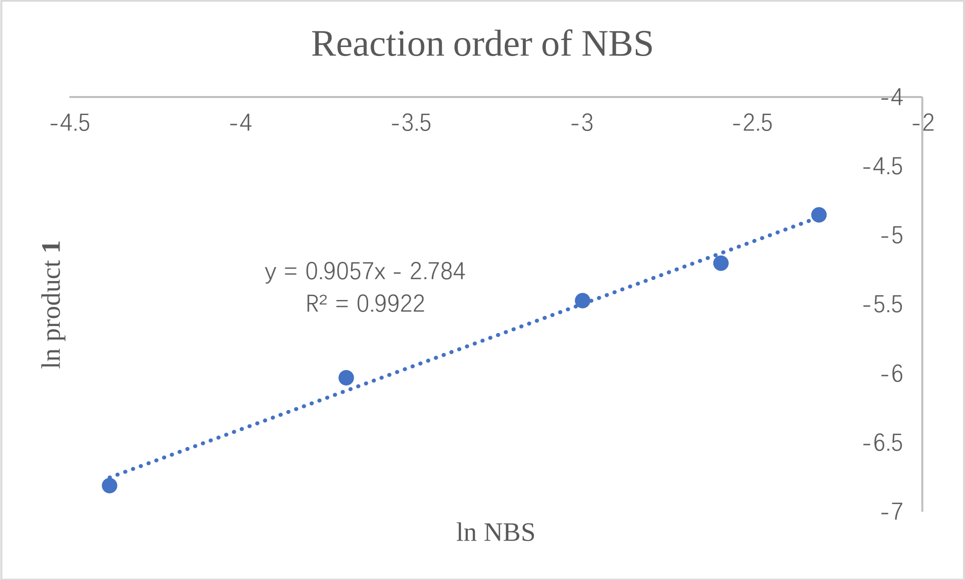

NBS (mmol)	ln (NBS)	**1** (mmol)	ln (**1**)
0.0125	-4.382	0.0011	-6.8124
0.025	-3.689	0.0024	-6.0323
0.05	-2.996	0.0042	-5.4727
0.075	-2.5903	0.0055	-5.203
0.1	-2.3026	0.0078	-4.8536

Figure S12. Reaction order of NBS

IV. Computational studies

(1) Computational Methods

All density functional theory (DFT) calculations were performed with the Gaussian 16 suite of programs.[1] The M06-2X functional has been shown in literature to give excellent results for a variety of organic transformations. Both geometry optimizations and frequency calculations were performed with the functional and the affordable 6-31G(d) basis set. The effect of the toluene solvent was simulated using Truhlar's SMD continuum model,[2] with a modified atomic radius of 2.60 Å, as described in the SMD18 article,[3] for geometries containing bromine atoms. Thermodynamic corrections at 253.15 K were obtained with the GoodVibes program,[4] employing a quasi-harmonic treatment for all low frequencies below the cut-off value of 100 cm^{-1} and adjusting the gas-phase thermochemistry to the 1 M standard state. Each stationary point was characterized as either a local energy minimum or a transition state by vibrational frequency analysis. Single-point energies were computed at the level of SMD(toluene)-M06-2X/6-311+G(2d,p) with the D3 version of Grimme's dispersion correction with Becke-Johnson damping.[5]

(2) CPA-TBP-NBS Ternary Complex

Our calculations show that **CPA-4**, **AP-8**, and NBS readily assemble into a thermodynamically stable ternary complex **TCP**, with a binding free energy of ~10–20 kJ/mol. This finding is further supported by 2D-ROESY NMR experiments (see SI Section III). The optimized structures (Figure S13) show that both key conformations of the ternary complex are stabilized by two key hydrogen bonds, OH(**CPA-4**)···O(NBS) and OH(**AP-8**)···O(**CPA-4**), as well as a strong π–π stacking interaction between **AP-8** and NBS. These cooperative non-covalent interactions pre-organize the components into a catalytically competent geometry. Functioning as a supramolecular scaffold, the ternary complex aligns the catalytic species in a spatial configuration that promotes selective substrate recognition. Upon substrate binding, the complex evolves into distinct pre-transition state (**PTS**) assemblies that guide the formation of the major (*R*) and minor (*S*) enantiomeric products.

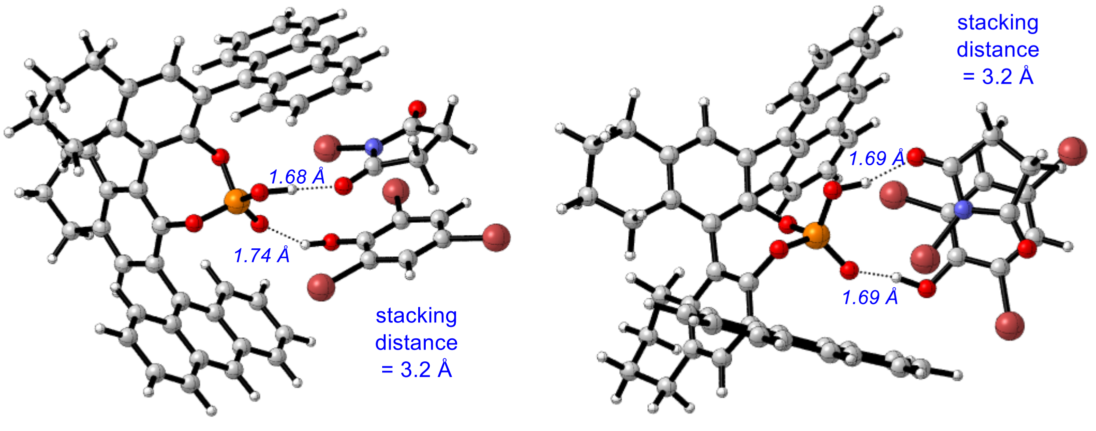

ΔG_{bind} = –21.2 kJ/mol ΔG_{bind} = –10.5 kJmol

Figure S13. Two key conformations of ternary complex **TCP**, with binding free energies (ΔG_{bind}) calculated at SMD-M06-2X-D3/6-311+G(2d,p)//SMD-M06-2X level in toluene solvent.

(3) Pre-Transition-State Geometry of AP-8-PTS^{R}

The pre-transition-state (**AP-8-PTS^{R}**) with **AP-8** is stabilized through hydrogen bonds, halogen bonds, and π–π stacking interactions.

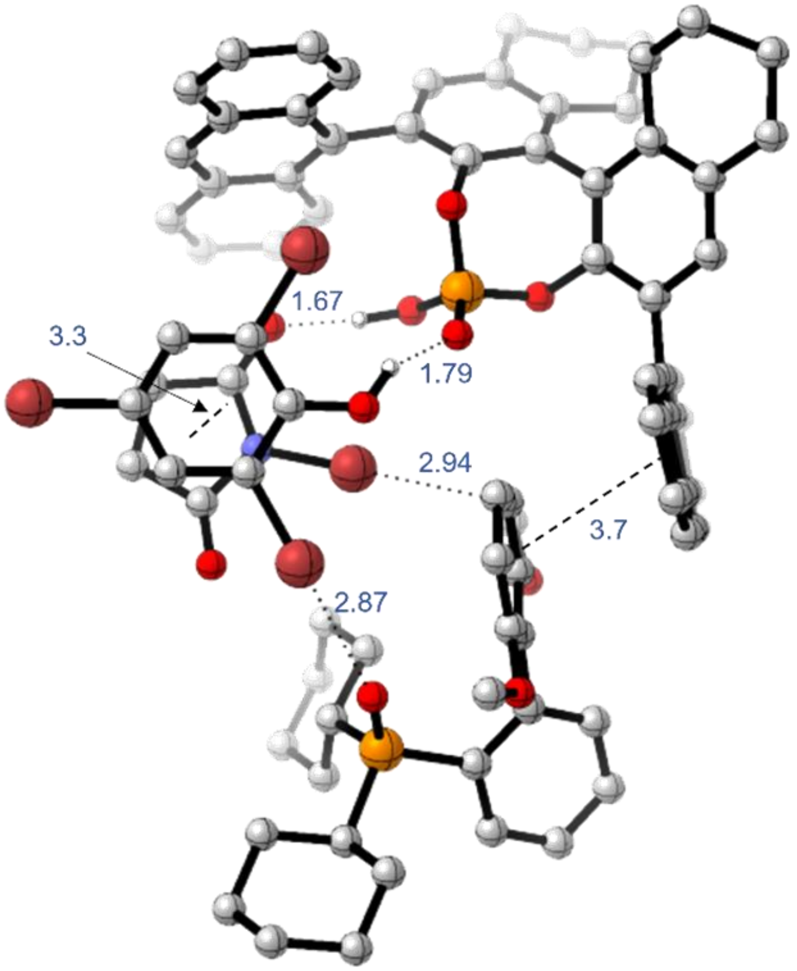

AP-8-PTS^{R}

Figure S14. Optimized geometry of pre-transition state complex **AP-8-PTS^{R}**. Interaction distances are given in Å.

(4) Transition State Configuration

It is generally advisable to explore the conformation of key transition states thoroughly to better estimate their energies and associated reaction barriers. Since the transition state **AP-8-TS1**^{R} is the key one to determine the reaction selectivity, its conformation was analyzed in detail to improve the reliability of the computational result. The phosphate catalyst, NBS and **AP-8** are all rigid molecules with only one low-lying conformation. On the other hand, the substrate **S1** contains a -P=OCy$_2$ group. While the P=O group is required to form a halogen bond with **AP-8**, the two P-Cy bonds are rotatable. In our study, we defined two angles θ_1 and θ_2 (Table S1) for the two rotatable bonds. A total of nine different conformations were optimized successfully and their relative energies are listed in Table S1. The lowest-energy conformation is **AP-8-TS1**^{R}, the one reported in the main text. In addition, we also considered one conformation where the additive **AP-8** forms a halogen bond with one of the oxygen atoms of the **CPA-4** catalyst and a hydrogen bond (HB) with the -P=OCy$_2$ group of the substrate, namely **AP-8-TS1**^{HB} in Table S1. Its energy was calculated to be 20.4 kJ/mol higher than **AP-8-TS1**^{R}, which has similar θ_1 and θ_2 values. In other words, it is preferable for the **AP-8** additive to form a hydrogen bond with **CPA-4** and a halogen bond with the -P=OCy$_2$ group of the substrate. Based on the conclusion of this study, the conformation of the -P=OCy$_2$ group was maintained in subsequent studies as the one in **AP-8-TS1**^{R}.

Table S1. Conformers of **AP-8-TS1*^R^*** by varying two torsions of the -POCy2 moiety of substrate **S1** or the HB binding pattern.

Conformer	θ_1/°	θ_2/°	ΔG/kJ mol^{-1}
**AP-8-TS1^{R}**	-46.3	-179.5	0.0
**AP-8-TS1^{R2}**	-75.2	38.4	18.2
**AP-8-TS1^{R3}**	-49.7	-42.1	24.3
**AP-8-TS1^{R4}**	48.1	159.9	15.7
**AP-8-TS1^{R5}**	47.3	42.9	20.7
**AP-8-TS1^{R6}**	46.5	-82.4	26.3
**AP-8-TS1^{R7}**	165.0	158.2	8.4
**AP-8-TS1^{R8}**	-173.0	37.7	11.5
**AP-8-TS1^{R9}**	-169.2	-96.6	14.8
**AP-8-TS1^{HB}**	-59.5	-155.8	20.4

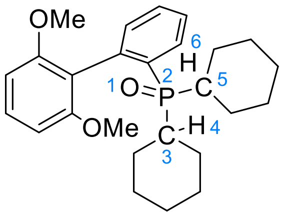

θ_1 = ∠O^1-**AP-8**-C^3-H^4
θ_2 = ∠O^1-**AP-8**-C^5-H^6

Maintaining the low-energy conformation of the -POCy2 moiety, three different low-lying transition states for the formation of the minor product (*S*)-**1** were located (Table S1). In contrast to the *R*-forming **AP-8-TS1*^R^***, none of the *S*-forming transition states exhibit the full network of non-covalent interactions. Specifically, in **AP-8-TS1*^S1^***, the halogen bond interaction is absent; in **AP-8-TS1*^S2^***, the hydrogen bonds form with neutral oxygen atoms rather than the negatively charged one; and in **AP-8-TS1*^S3^***, the π–π stacking interaction is missing. Among the three**, AP-8-TS1*^S1^*** is the lowest in energy, highlighting the critical role of halogen bond in the *R*-forming **AP-8-TS1*^R^*** in governing stereoselectivity.

(5) Transition State CPA-4-TS1*R*

The **CPA-4**-catalyzed bromination of biaryl substrate **S1** by NBS in the absence of **AP-8** was investigated computationally to elucidate the role of **AP-8** in enhancing the reaction rate. The bromination step proceeding through a **TS1*R***-like transition state (i.e. **CPA4-TS1*R***) was assumed to be the rate-determining step. The calculated activation barrier relative to the reactants was 56.0 kJ/mol, which is higher than the 40.9 kJ/mol barrier obtained for the corresponding reaction in the presence of **AP-8**. These computational results are consistent with the experimental observation of significantly lower product yield in the absence of **AP-8**.

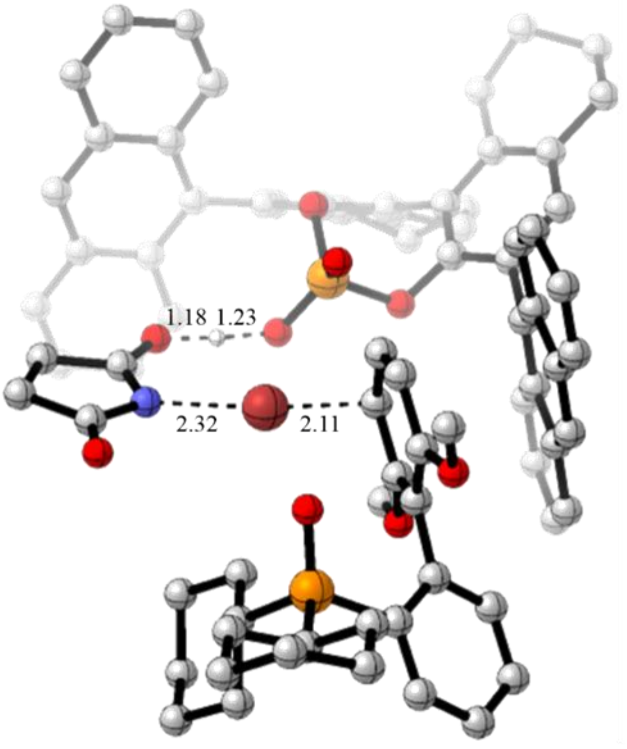

CPA-4-TS1*R*
$\Delta\Delta G_{253}{}^{\ddagger}$ = 56.0 kJ/mol

Figure S15. Optimized Structure of **CPA4-TS1*R***. Interaction distances are given in Å.

(6) NCI plot of AP-8-TS1^{S1}

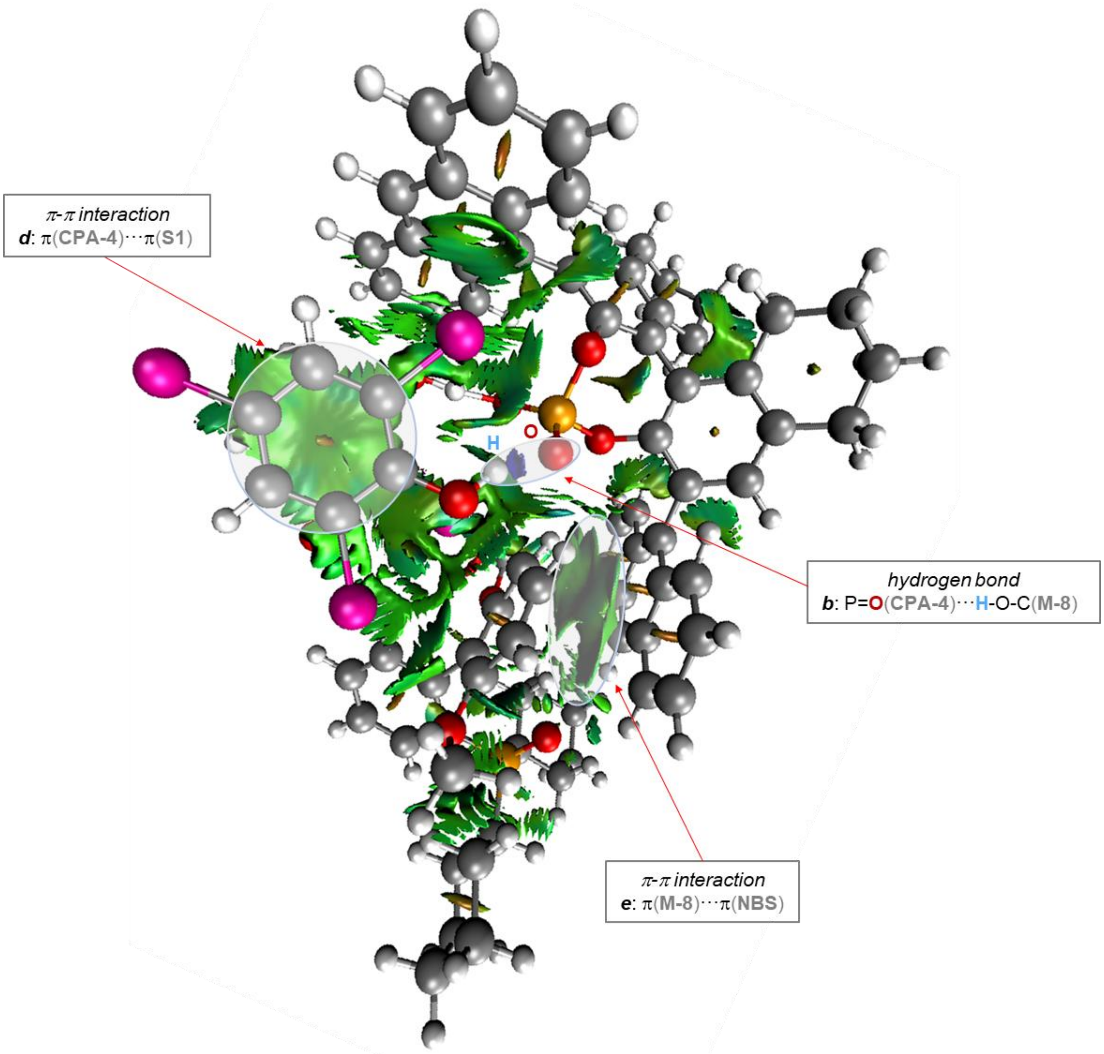

Figure S16. Analysis of the non-covalent interactions in the minor **TS1**^{S1} transition state.

(7) Halogen Bond and Regiospecificity

As discussed in the main text, a halogen bond (XB) exists in the pre-transition state complex **PTS*^R^***, with *N*-bromine moiety of NBS directed towards the *ortho*-position of the substituted phenyl ring of substrate **S1** (Figure S17). Previous theoretical studies have shown that XB interactions involving an aromatic XB acceptor are rim and site specific and the favorable interaction sites can be explained in terms of the highest occupied molecular orbital (HOMO) and atomic charges.[7-9] To further investigate the interaction sites of XB interaction in substrate **S1**, we examined XB complexes between **S1** and NBS. We identified stable XB complexes at 2- and 4-positions of the substituted phenyl ring with the over-atom (η_1) binding mode. These XB complexes exhibit significant interaction energies of −30.7 and −30.2 kJ/mol, respectively, at M06-2X-D3/6-311+G(2d,p)//M06-2X-6-31G* level of theory. No stable XB complexes were located at the C1, C3 and C5 positions. The favorable XB binding sites at C2 and C4 align well with those predicted by the HOMO and atomic charge distributions of the phenyl ring. In particular, the strong negative charges at the C2 and C4 positions interact favorably with the positively charged bromine σ-hole, accounting for the over-atom binding geometries. In summary, the site-specific halogen bond in **PTS^{R}** plays a pivotal role in dictating the observed regioselectivity of the bromination reaction.

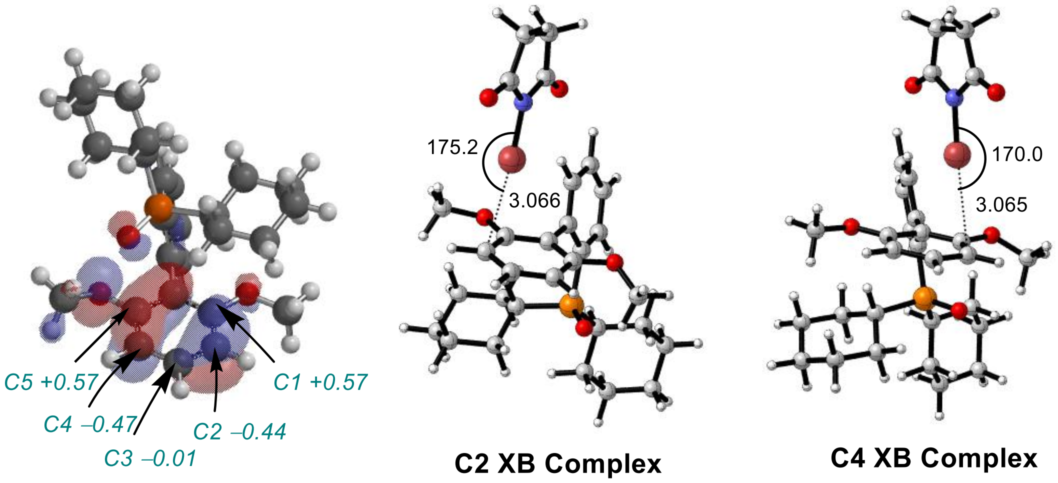

Figure S17. HOMO and atomic charges of **S1** and optimized geometries of XB complexes between substrate **S1** and NBS. Distances are reported in Å and angles in degrees.

V. Procedures for the preparation of substrates

(1) *General Procedure A*[10]

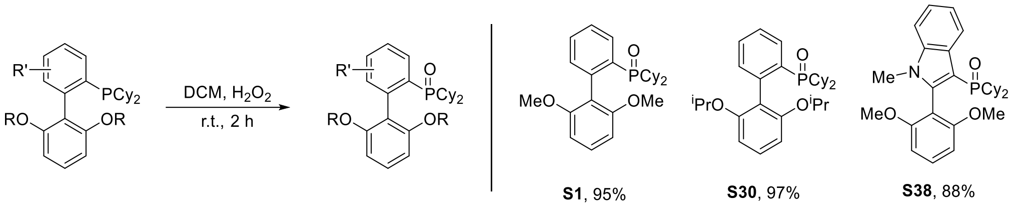

To a solution of biarylphosphines (2 mmol) in DCM (20 mL, 0.1 M) was added H_2O_2 (30% aqueous solution, 20 mmol, 10.0 equiv) and the reaction was stirred at room temperature for 2 hours. The layers were then separated, and the organics were dried over anhydrous Na_2SO_4 and concentrated under reduced pressure. The residue was purified by column chromatography (hexane/EtOAc 1:1 to 1:3) to afford the biarylphosphine oxides.

(2) *General Procedure B*[11]

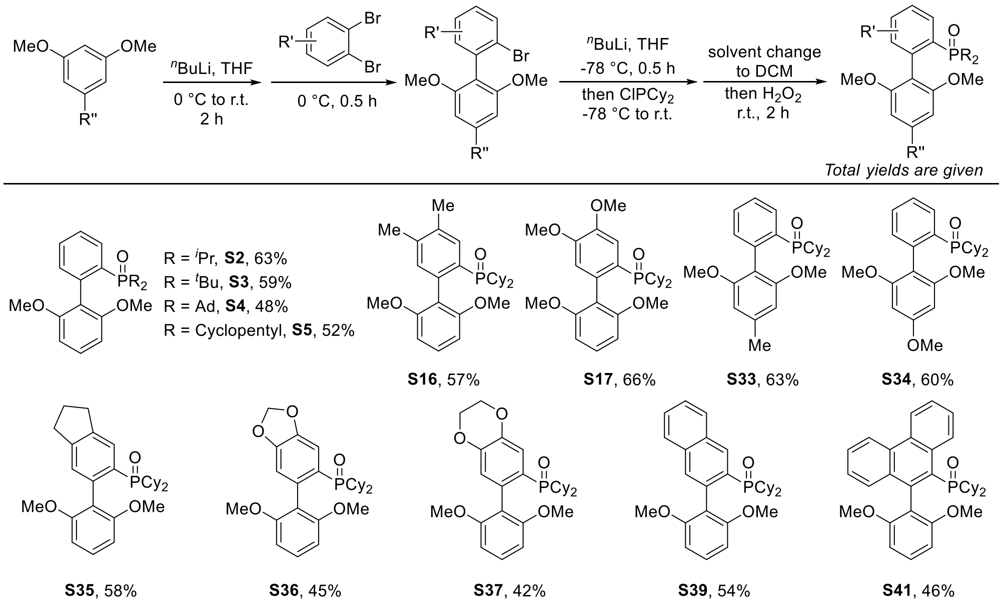

To a solution of substituted 1,3-dimethoxybenzene (11 mmol, 1.1 equiv) in THF (25 mL, 0.4 M) at 0 ºC was added ^{n}BuLi (11 mmol, 2.7 M in hexanes, 1.1 equiv) dropwise over 10 minutes. The resulting solution was allowed to warm to room temperature and stirred for an additional 2 h. The mixture was cooled to 0 ºC again and substituted dibromobenzene (10 mmol, 1.0 equiv) was added dropwise over 5 minutes. The resulting red solution was stirred for an additional 30 minutes at 0 ºC before the addition

of MeOH (2 ml) to quench the reaction. The solvent was then removed under reduced pressure and the residue was dissolved in DCM. The solution was washed with water and brine, dried over anhydrous Na_2SO_4, filtered, and concentrated under reduced pressure. The residue was recrystallized from methanol to afford the biaryl bromides.

To a solution of biaryl bromides (2.0 mmol, 1.0 equiv) in dry THF (20 mL, 0.1 M) at -78 ºC was added ^{n}BuLi (2.2 mmol, 2.7 M in hexanes,1.1 equiv) dropwise over 10 minutes. The resulting mixture was stirred at -78 °C for 0.5 h. Neat $ClPR_2$ (2.4 mmol, 1.2 equiv) was then added with a syringe. The reaction mixture was stirred at -78 °C for 0.5 h and then allowed to slowly warm to room temperature. The solvent was then removed under reduced pressure. The residue was dissolved in DCM (20 ml, 0.1 M) and H_2O_2 (20 mmol, 30% aqueous solution, 10.0 equiv), and the reaction was stirred at room temperature for 2 hours. The organic layer was separated, dried over Na_2SO_4, filtered, and concentrated under reduced pressure. The residue was purified by column chromatography (hexane/EtOAc 1:1 to 1:3) to give the target phosphine oxides.

(3) *General Procedure C*[12]

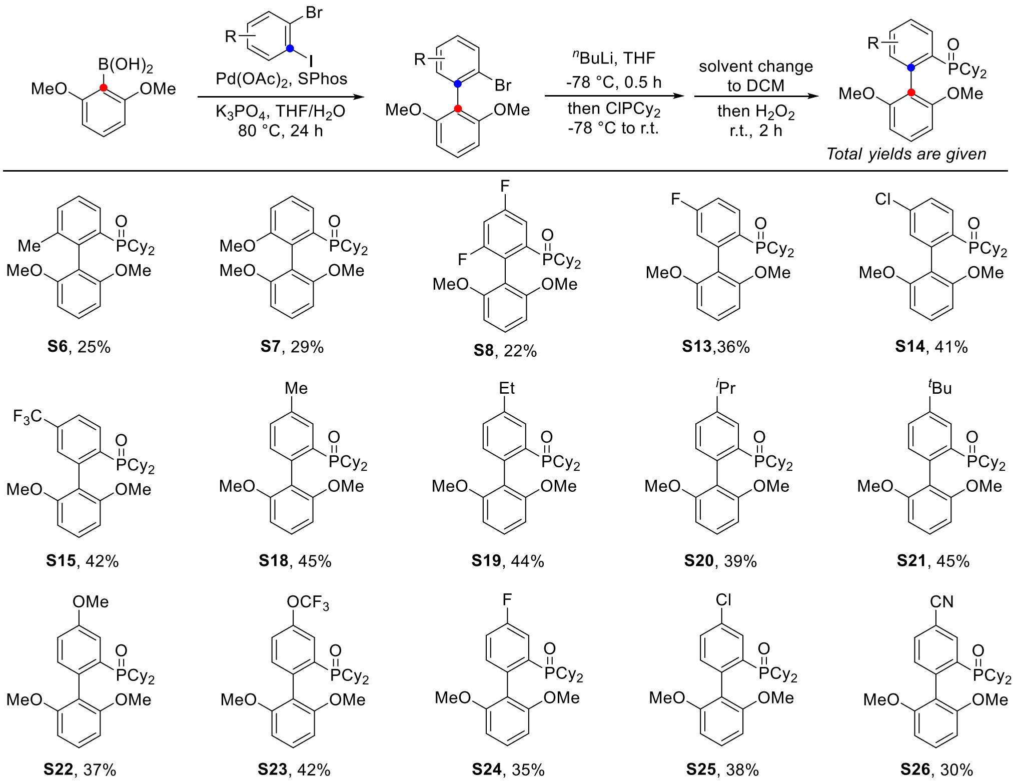

A Schlenk sealed tube with a magnetic stirrer bar was charged with 2,6-dimethoxyphenylboronic acid (5 mmol, 1.0 equiv), 1-bromo-2-iodobenzene (6 mmol, 1.2 equiv), $Pd(OAc)_2$ (0.1 mmol, 2.0 mol%), SPhos (0.2 mmol, 4.0 mol%), K_3PO_4 (15

mmol, 3.0 equiv) and degassed THF/H_2O (4/1, v/v, 0.4 M) under N_2. The reaction mixture was heated at 80 °C for 24 hours. The reaction mixture was cooled to room temperature and extracted with EtOAc. The combined organic layer was dried over anhydrous Na_2SO_4, filtered and evaporated under reduced pressure. The residue was purified by column chromatography (hexane/EtOAc 1:1 to 1:3) to afford the biaryl bromides.

To a -78 ºC solution of biaryl bromides (2 mmol, 1.0 equiv) in dry THF (20 ml, 0.1 M) was added ^{n}BuLi (2.2 mmol, 2.7 M in hexanes, 1.1 equiv) dropwise over 10 minutes. The resulting mixture was stirred at -78 °C for 0.5 h. Neat chlorodicyclohexylphosphine (2.4 mmol, 1.2 equiv) was then added with a syringe. The reaction mixture was stirred at -78 °C for 0.5 h and then allowed to slowly warm to room temperature. The solvents were then removed under reduced pressure. DCM (20 ml, 0.1 M) and H_2O_2 (20 mmol, 30% aqueous solution, 10.0 equiv) were added and the reaction was stirred at room temperature for 2 hours. The layers were then separated and the organics were dried over Na_2SO_4 and concentrated under reduced pressure. The residue was purified by column chromatography (hexane/EtOAc 1:1 to 1:3) gave the target phosphine oxides.

(4) *General Procedure D*[13]

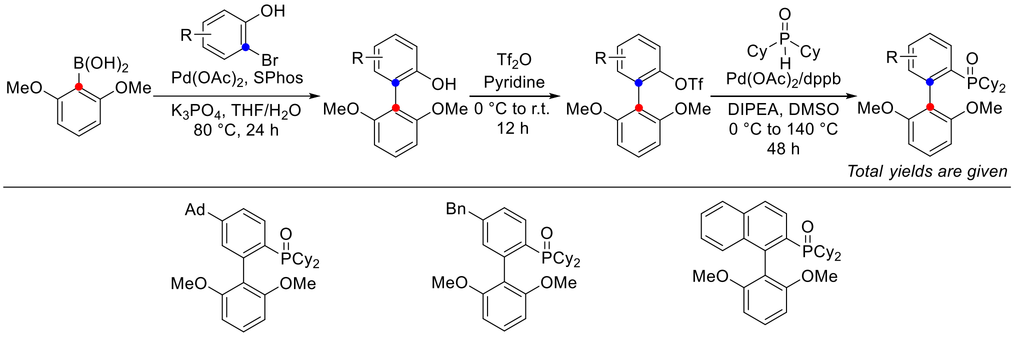

S10,46% **S11**, 52% **S40**, 49%

A Schlenk sealed tube with a magnetic stirrer bar was charged with 2-bromophenol (5 mmol, 1.0 equiv), 2,6-dimethoxyphenylboronic acid (7.5 mmol, 1.5 equiv), $Pd(OAc)_2$ (0.1 mmol, 2.0 mol%), SPhos (0.2 mmol, 4.0 mol%), K_3PO_4 (15 mmol, 3.0 equiv) and degassed THF/H_2O (4/1, v/v, 0.4 M) under N_2. The reaction mixture was heated at 80 °C for 24 hours. The reaction mixture was cooled to room temperature then extracted with EtOAc. The combined organic layer was dried over anhydrous Na_2SO_4, filtered and concentrated under reduced pressure. The residue was purified by column chromatography (hexane/EtOAc 10:1 – hexane/EtOAc 5:1) to afford the biaryl phenols.

To a solution of biaryl phenols (4 mmol, 1.0 equiv) in pyridine (8 mL, 0.5 M) was added trifluoromethanesulfonic anhydride (8 mmol, 2.0 equiv) dropwise at 0 °C. Then, the mixture was stirred at room temperature for 12 h. The reaction mixture was poured into H_2O and extracted with EtOAc. The combined organic layer was washed with aqueous HCl (10%) and saturated brine. The organic layer was dried over anhydrous Na_2SO_4. The solvent was concentrated under reduced pressure and the residue was purified by column chromatography (hexane/EtOAc 10:1) to afford the trifluoromethanesulfonyloxy biphenyls.

A dry Schlenk sealed tube with a magnetic stirrer bar was charged with trifluoromethanesulfonyloxy biphenyls (2 mmol, 1.0 equiv), dicyclohexylphosphine oxide (2.4 mmol, 1.2 equiv), $Pd(OAc)_2$ (0.2 mmol, 10.0 mol%), dppb (0.24 mmol, 12.0 mol%), dry DIPEA (8 mmol, 4.0 equiv) and dry DMSO (10 mL, 0.2 M) under N_2. The reaction mixture was stirred at room temperature for 30 minutes and then heated at 140 °C for 48 h. The reaction mixture was cooled to room temperature and extracted with EtOAc. The combined organic layer was dried over anhydrous Na_2SO_4, filtered, and concentrated under reduced pressure. The residue was purified by column chromatography (hexane/EtOAc 1:1 to 1:3) to afford the target phosphine oxides.

(5) *General Procedure E*[14]

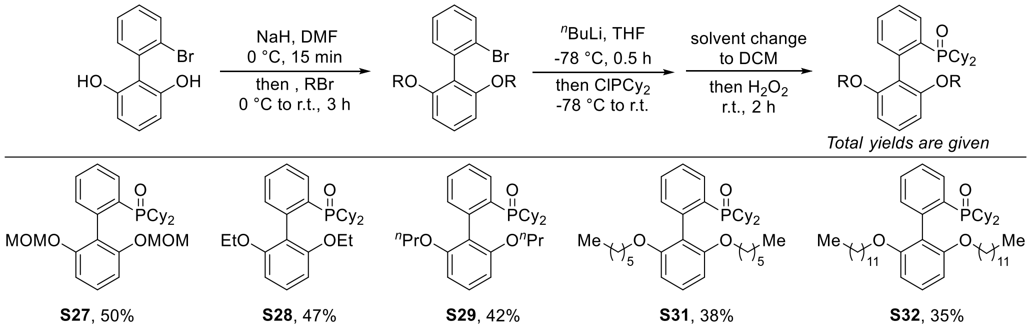

To a solution of 2-bromobiphenyl-2',6'-diol (2 mmol, 1 equiv.) in dry DMF (4 mL, 0.5 M), NaH (6 mmol, 3 equiv.) was added portionwise at 0 ºC. After stirring the solution for 15 min at the same temperature, RBr (5 mmol, 2.5 equiv.) was added dropwise. The solution was allowed to warm to room temperature and stirred for 3 hours. Saturated aqueous $NaHCO_3$ was carefully added to the solution. The aqueous phase was extracted with EtOAc. The combined organic layer was dried over anhydrous Na_2SO_4, filtered, and concentrated under reduced pressure. The crude products were used in the next step without further purification.

To a solution of biaryl bromides (2 mmol, 1.0 equiv) in dry THF (20 mL, 0.1 M) at -78 °C was added ^{n}BuLi (2.2 mmol, 2.7 M in hexanes, 1.1 equiv) dropwise over 10 minutes. The resulting mixture was stirred at -78 °C for 0.5 h. Neat chlorodicyclohexylphosphine (2.4 mmol, 1.2 equiv) was then added with a syringe. The reaction mixture was stirred at -78 °C for 0.5 h and then allowed to slowly warm to room temperature. The solvents were then removed under reduced pressure. The residue was dissolved in DCM (20 mL, 0.1 M) and H_2O_2 (20 mmol, 30% aqueous solution, 10.0 equiv), and the reaction was stirred at room temperature for 2 hours. The organic layer was separated, dried over anhydrous Na_2SO_4, and concentrated under reduced pressure. The residue was purified by column chromatography (hexane/EtOAc 1:1 to 1:3) to give the target phosphine oxides.

(6) *The preparation of substrate S9*[12]

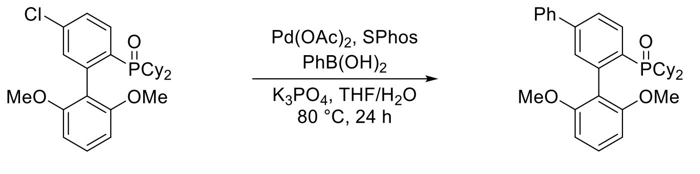

S14 **S9**, 93%

A Schlenk sealed tube with a magnetic stirrer bar was charged with **S14** (0.5 mmol, 1.0 equiv), benzeneboronic acid (0.75 mmol, 1.5 equiv), $Pd(OAc)_2$ (0.01 mmol, 2.0 mol%), SPhos (0.02 mmol, 4.0 mol%), K_3PO_4 (1.5 mmol, 3.0 equiv), and degassed THF/H_2O (4/1, v/v, 0.4 M) under N_2. The reaction mixture was heated at 80 °C for 24 h. The reaction mixture was cooled to room temperature and extracted with EtOAc. The combined organic layer was dried over anhydrous Na_2SO_4, filtered, and concentrated under reduced pressure. The residue was purified by column chromatography (hexane/EtOAc 1:1 to 1:3) to afford **S9** as a white solid.

(7) ***The preparation of substrate S12***[15]

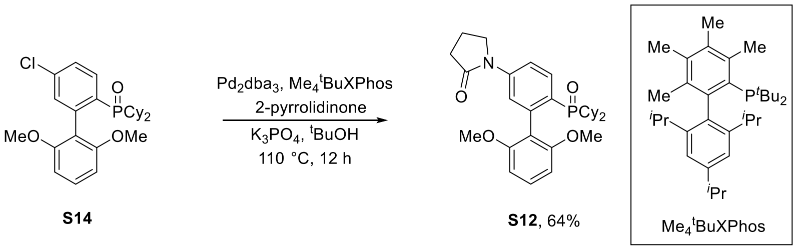

A Schlenk sealed tube with a magnetic stirrer bar was charged with **S14** (1.0 mmol, 1.0 equiv), 2-pyrrolidinone (1.2 mmol, 1.2 equiv), Pd_2dba_3 (0.005 mmol, 0.5 mol%), $Me_4{}^tBuXPhos$ (0.025 mmol, 2.5 mol%), K_3PO_4 (1.2 mmol, 1.2 equiv), and degassed ^tBuOH (2 ml, 0.5 M) under N_2. The reaction mixture was heated at 110 °C for 12 h. The reaction mixture was cooled to room temperature and extracted with EtOAc. The combined organic layer was dried over anhydrous Na_2SO_4, filtered, and concentrated under reduced pressure. The residue was purified by column chromatography ($CHCl_3$/EtOAc 1:3) to afford **S12** as a white solid.

VI. General procedure for the atroposelective remote bromination

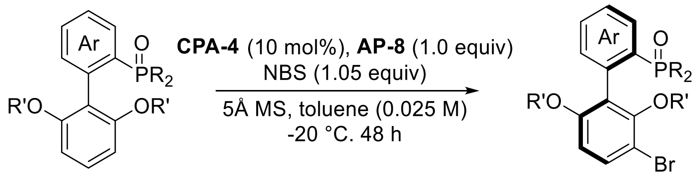

To a solution of phosphine oxides (0.05 mmol), catalyst **CPA-4** (0.005 mmol), **AP-8** (0.05 mmol) and 5Å MS (30 mg) in toluene (2 mL) was added NBS (0.0525 mmol) at -20 °C in the absence of light. The resultant mixture was stirred for 48 h and quenched with saturated aqueous Na_2SO_3 solution (2 mL). The solution was extracted with EtOAc (3 × 5 mL). The combined organic layer was dried over anhydrous Na_2SO_4 and concentrated under reduced pressure. The residue was subjected to flash column chromatography (hexane/EtOAc 1:1 – $CHCl_3$/EtOAc 1:3) to afford target products.

VII. Procedures for product diversification

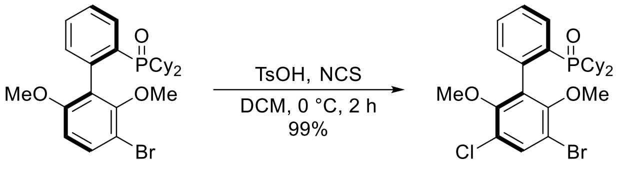

1 **42**

To a mixture of phosphine oxide **1** (0.1 mmol) and TsOH (0.01 mmol) in DCM (4 mL) was added NCS (0.105 mmol) at 0 °C. The resultant mixture was stirred for 2 h and quenched with saturated aqueous Na_2SO_3 solution (2 mL). The organic layer was separated and the aqueous layer was extracted with DCM (3 × 15 mL). The combined layer was dried over anhydrous Na_2SO_4, filtered, and concentrated under reduced pressure. The residue was purified by column chromatography ($CHCl_3$/EtOAc 1:3) to give **42** as a colorless foam.

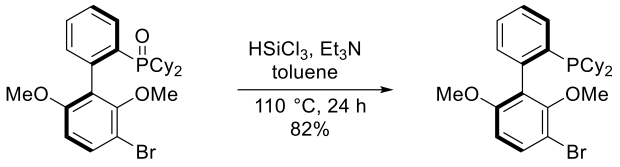

1 **(*R*)-SPhos-Br**

(*R*)-SPhos-Br was synthesized according to a literature procedure.[13] To a mixture of phosphine oxide **1** (0.2 mmol) and Et_3N (2.0 mmol) in toluene (5 mL) was added trichlorosilane (4.0 mmol) at 0 °C. The reaction mixture was stirred at 110 °C for 24 h. After cooling to room temperature, the mixture was quenched with saturated sodium hydroxide at 0 °C. The layers were separated, and the aqueous layer was extracted with DCM (3 × 15 mL). The combined layer was dried over anhydrous Na_2SO_4, filtered, and concentrated under reduced pressure. The residue was purified by column chromatography (hexane/DCM 2:1) to give the monophosphine ligand **(*R*)-SPhos-Br**.

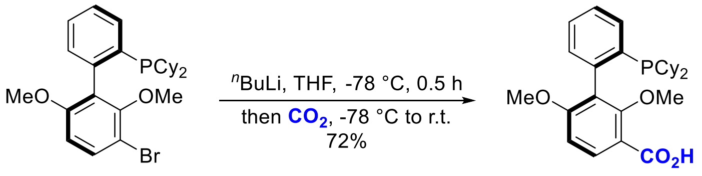

(*R*)-SPhos-Br **(*R*)-SPhos-CO_2H**

To a solution of **(*R*)-SPhos-Br** (0.2 mmol, 1.0 equiv) in dry THF (2 mL, 0.1 M) at -78

°C was added ^nBuLi (0.22 mmol, 1.0 M in hexanes, 1.1 equiv) dropwise over 1 minute under nitrogen atmosphere. The resulting mixture was stirred at -78 °C for 0.5 h. The nitrogen atmosphere in the reaction vessel was removed under reduced pressure and the vessel was recharged with carbon dioxide. The reaction mixture was stirred at -78 °C for 0.5 h and then allowed to slowly warm to room temperature. Upon completion, the reaction was quenched with aqueous NH_4Cl solution (20 mL) and extracted with EtOAc (20 mL × 3). The combined organic layer was dried over anhydrous Na_2SO_4, filtered, and concentrated under reduced pressure. The residue was purified by column chromatography (DCM/MeOH 30:1 – 15:1) to give the target phosphine ligand **(*R*)-SPhos-CO_2H** as a colorless foam.

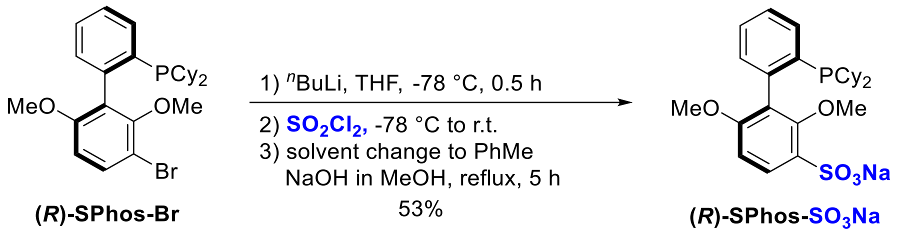

To a solution of **(*R*)-SPhos-Br** (0.2 mmol, 1.0 equiv) in dry THF (2 mL, 0.1 M) at -78 °C was added ^nBuLi (0.22 mmol, 1.0 M in hexanes, 1.1 equiv) dropwise over 1 minute under a nitrogen atmosphere. The resulting mixture was stirred at -78 °C for 0.5 h. Then, a solution of SO_2Cl_2 (0.24 mmol, 1.2 equiv) in dry THF (2.4 mL, 0.1 M) was added slowly to the reaction mixture at -78 °C. The reaction was allowed to slowly warm to room temperature. The solvent was then removed under reduced pressure. The residue was dissolved in toluene (4 mL, 0.05 M) and NaOH (2 M in MeOH, 50 equiv) under N_2. The reaction mixture was heated at reflux for 5 h before cooling to room temperature. The solution was extracted with EtOAc. The combined organic layer was dried over anhydrous Na_2SO_4, filtered, and concentrated under reduced pressure. The residue was purified by column chromatography to afford the **(*R*)-SPhos-SO_3Na** as a white solid.

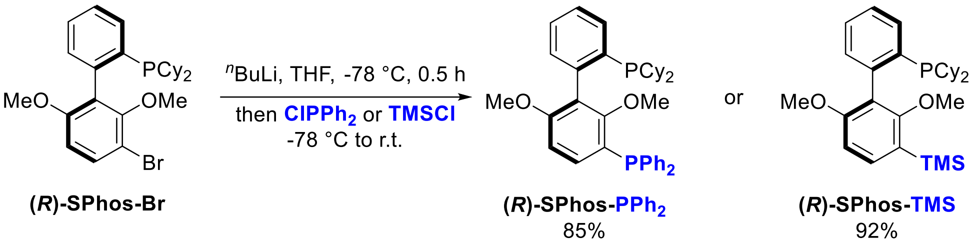

To a solution of **(*R*)-SPhos-Br** (0.2 mmol, 1.0 equiv) in dry THF (2 mL, 0.1 M) at -78 ºC was added ^{n}BuLi (0.22 mmol, 1.0 M in hexanes, 1.1 equiv) dropwise over 1 minute under nitrogen atmosphere. The resulting mixture was stirred at -78 °C for 0.5 h. Neat chlorodiphenylphosphine or trimethylchlorosilane (0.24 mmol, 1.2 equiv) was then added with a syringe. The reaction mixture was stirred at -78 °C for 0.5 h and then allowed to slowly warm to room temperature. Upon completion, the reaction was quenched with saturated aqueous NH_4Cl solution (20 mL) and extracted with EtOAc (20 mL × 3). The combined organic layer was dried over anhydrous Na_2SO_4, filtered, and concentrated under reduced pressure. The residue was purified by column chromatography (hexane/DCM 10:1 – 2:1) to give the target phosphine ligand **(*R*)-SPhos-PPh₂** or **(*R*)-SPhos-TMS** as a colorless foam.

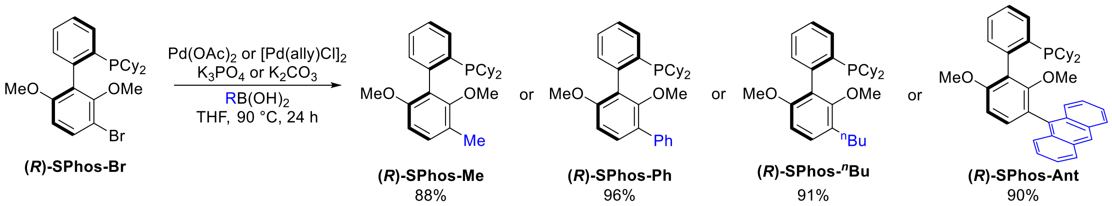

A Schlenk sealed tube with a magnetic stirrer bar was charged with **(*R*)-SPhos-Br** (0.1 mmol, 1.0 equiv), $RB(OH)_2$ (0.12 – 2.0 mmol, 1.2 – 2.0 equiv), $Pd(OAc)_2$ (0.002 mmol, 2.0 mol%) or $[Pd(ally)Cl]_2$ (0.001 mmol, 1.0 mol%), K_3PO_4 or K_2CO_3 (0.3 mmol, 3.0 equiv), and degassed THF/H_2O (4/1, v/v, 0.5 mL, 0.2 M) under N_2. The reaction mixture was heated at 90 °C for 24 h. The reaction mixture was cooled to room temperature then extracted with EtOAc. The combined organic layer was dried over anhydrous Na_2SO_4, filtered, and concentrated under reduced pressure. The residue was purified by column chromatography (hexane/DCM 10:1 – 2:1) to afford the target phosphine ligands.

VIII. Procedures for application of the new ligands in asymmetric catalysis

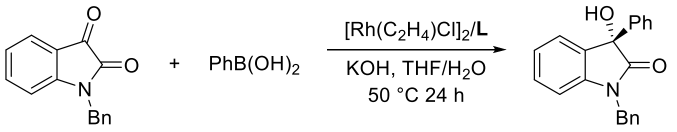

A Schlenk sealed tube with a magnetic stirrer bar was charged with $[RhCl(C_2H_4)_2]_2$ (0.01 mmol Rh) and **L** (0.02 mmol) and THF (1.0 mL) under N_2. The reaction mixture was stirred for 10 min at room temperature. Aqueous KOH (0.3 M, 0.10 mL, 0.03 mmol), isatin **43** (0.20 mmol), and $PhB(OH)_2$ (**44**, 0.40 mmol) were added successively with additional THF (1.0 mL). The resulting mixture was stirred at 50 °C for 24 h. The solution was concentrated under reduced pressure and the residue was purified by column chromatography to afford the desired chiral product **45** as a white solid.

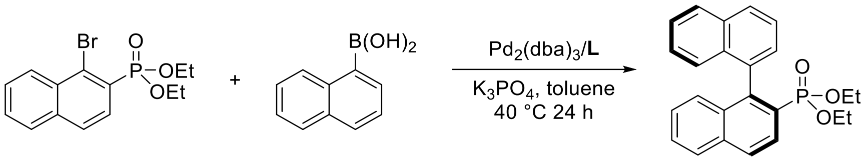

A Schlenk sealed tube with a magnetic stirrer bar was charged with **46** (0.10 mmol), **47** (0.15 mmol), $Pd_2(dba)_3$ (1.0 mol%), **L** (2.4 mol%), K_3PO_4 (0.40 equiv), and degassed toluene (1 mL) under N_2. The reaction mixture was heated at 40 °C for 24 h. The reaction mixture was cooled to room temperature and extracted with EtOAc. The combined organic layer was dried over anhydrous Na_2SO_4, filtered, and concentrated under reduced pressure. The residue was purified by column chromatography to afford the desired chiral product **48** as a white solid.

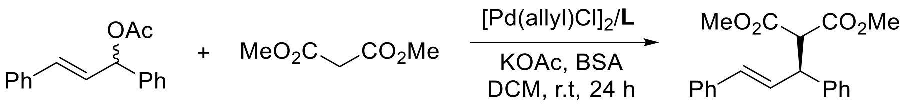

A Schlenk sealed tube with a magnetic stirrer bar was charged with $[Pd(allyl)Cl]_2$ (0.002 mmol), **L** (0.004 mmol), KOAc (0.006 mmol), *rac*-1,3-diphenyl-2-propenyl acetate (**49**, 0.1 mmol), dimethyl malonate (**50**, 0.03 mmol), BSA (0.3 mmol), and DCM (1.0 mL) under N_2. The resulting mixture was stirred at room temperature for 24 h. The reaction

was diluted with EtOAc (50.0 mL), and washed with water (50 mL) and saturated brine (50 mL). The organic layer was dried over anhydrous Na_2SO_4, filtered, and concentrated under reduced pressure. The residue was purified by column chromatography to afford the desired chiral product **51** as a colorless oil.

IX Experimental data for the described substances

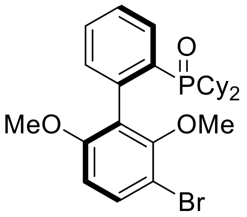

(*R*)-(3'-bromo-2',6'-dimethoxy-[1,1'-biphenyl]-2-yl)dicyclohexylphosphine oxide (1)

Appearance: colorless foam;

Yield: 98%; **e.e**: 98%; $[\alpha]_D^{25}$ = 21 (*c* 0.1, $CHCl_3$);

^{1}H NMR (400 MHz, $CDCl_3$): δ = 7.94 – 7.82 (m, 1H), 7.60 – 7.41 (m, 3H), 7.23 – 7.15 (m, 1H), 6.62 (d, *J* = 8.8 Hz, 1H), 3.67 (s, 3H), 3.48 (s, 3H), 1.89 – 1.37 (m, 12H), 1.36 – 0.93 (m, 1H) ppm.

^{13}C NMR (101 MHz, $CDCl_3$): δ = 157.30, 155.13, 137.30 (d, *J* = 6.4 Hz), 132.96 (d, *J* = 7.9 Hz), 132.68, 132.41 (d, *J* = 8.8 Hz), 131.95 (d, *J* = 81.0 Hz), 130.10 (d, *J* = 2.7 Hz), 127.02 (d, *J* = 10.1 Hz), 126.59 (d, *J* = 2.3 Hz), 108.18, 107.48, 60.52, 55.72, 37.79 (d, *J* = 60.3 Hz), 37.27 (d, *J* = 60.3 Hz), 26.86 – 26.46 (m), 26.23 (d, *J* = 3.2 Hz), 25.96, 25.87 – 25.64 (m) ppm.

^{31}P NMR (162 MHz, $CDCl_3$): δ = 47.87 ppm.

HRMS (ESI$^+$): calcd $[M+Na]^+$ for $[C_{26}H_{34}BrO_3PNa]^+$: 527.13212, found 527.13237.

HPLC condition: Daicel Chiralpak IC, i-PrOH/n-hexane = 10/90, 0.8 mL/min, 254 nm UV detector; t_1= 33.2 min (major), t_2 = 38.1 min (minor).

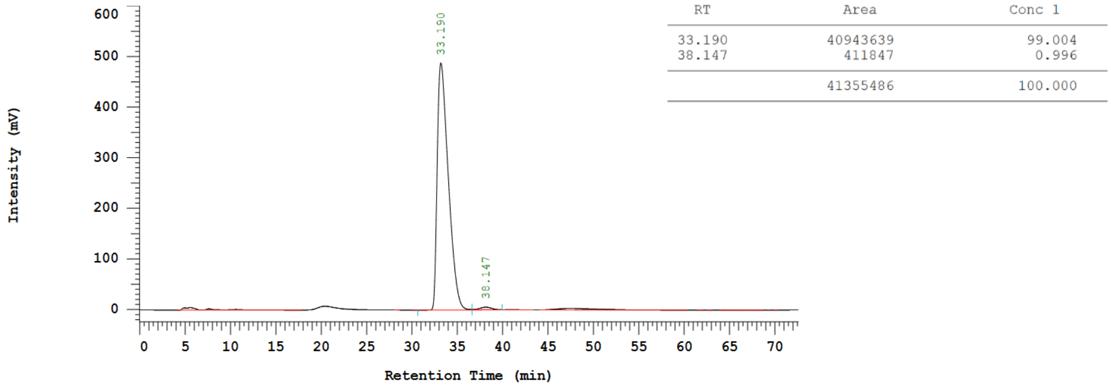

RT	Area	Conc 1
33.190	40943639	99.004
38.147	411847	0.996
	41355486	100.000

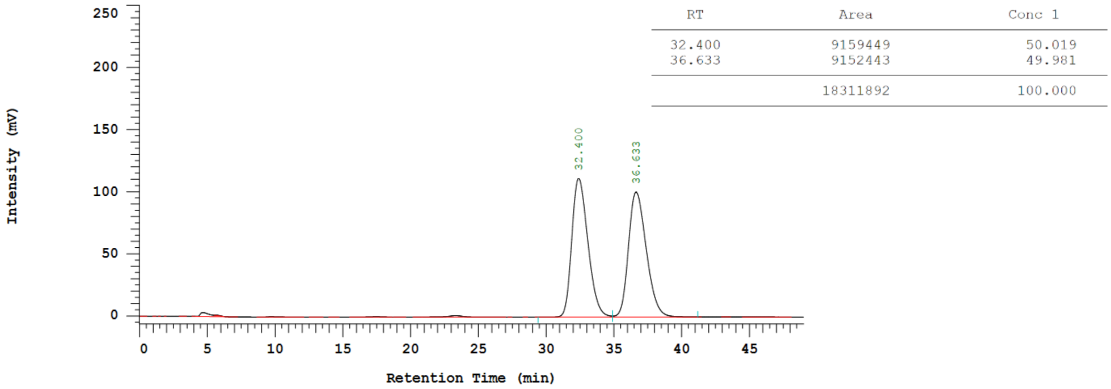

RT	Area	Conc 1
32.400	9159449	50.019
36.633	9152443	49.981
	18311892	100.000

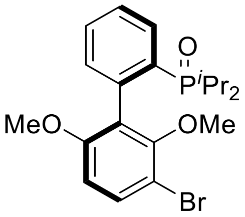

(*R*)-(3'-bromo-2',6'-dimethoxy-[1,1'-biphenyl]-2-yl)diisopropylphosphine oxide (2)

Appearance: colorless foam;

Yield: 96%; **e.e**: 93%; $[\alpha]_D^{25}$ = 32 (*c* 0.1, $CHCl_3$);

^{1}H NMR (400 MHz, $CDCl_3$): δ = 7.85 – 7.75 (m, 1H), 7.56 – 7.42 (m, 3H), 7.25 – 7.18 (m, 1H), 6.60 (d, *J* = 8.9 Hz, 1H), 3.65 (s, 3H), 3.52 (s, 3H), 2.10 (hept, *J* = 7.2 Hz, 1H), 1.88 (hept, *J* = 7.0 Hz, 1H), 1.19 – 1.07 (m, 6H), 1.06 – 0.94 (m, 6H) ppm.

^{13}C NMR (101 MHz, $CDCl_3$): δ = 157.44, 155.06, 138.11 (d, *J* = 5.9 Hz), 132.66, 132.50 (d, *J* = 9.2 Hz), 132.33 (d, *J* = 8.6 Hz), 131.57 (d, *J* = 82.5 Hz), 130.28 (d, *J* = 2.7 Hz), 127.01 (d, *J* = 10.5 Hz), 126.54 (d, *J* = 2.4 Hz), 108.02, 107.40, 60.71, 55.53, 27.48 (d, *J* = 37.5 Hz), 26.96 (d, *J* = 37.6 Hz), 17.09 (d, *J* = 3.2 Hz), 16.82 (d, *J* = 3.1 Hz), 16.03 (dd, *J* = 5.9, 2.9 Hz) ppm.

^{31}P NMR (162 MHz, $CDCl_3$): δ = 53.13 ppm.

HRMS (ESI$^+$): calcd $[M+Na]^+$ for $[C_{20}H_{26}BrO_3PNa]^+$: 447.06952, found 447.06948.

HPLC condition: Daicel Chiralpak IC, i-PrOH/n-hexane = 20/80, 0.8 mL/min, 254 nm UV detector; t_1= 18.5 min (major), t_2 = 21.5 min (minor).

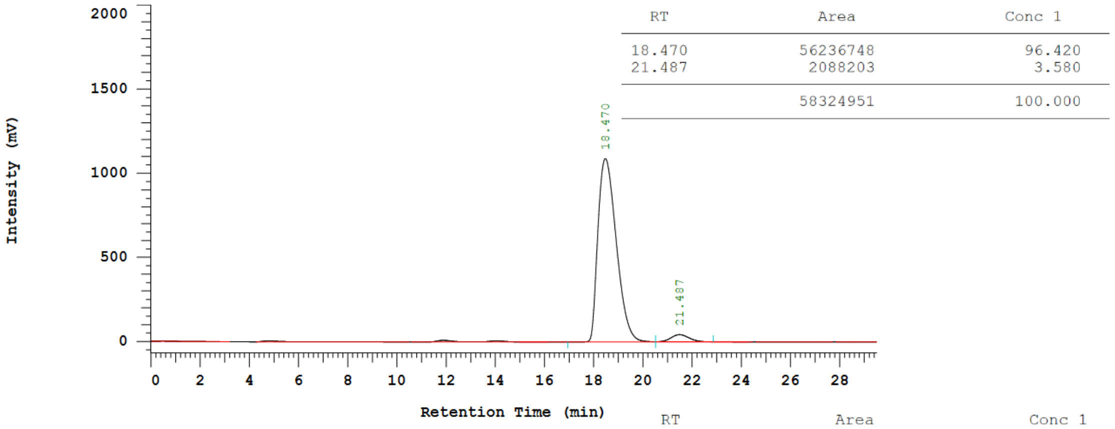

RT	Area	Conc 1
18.470	56236748	96.420
21.487	2088203	3.580
	58324951	100.000

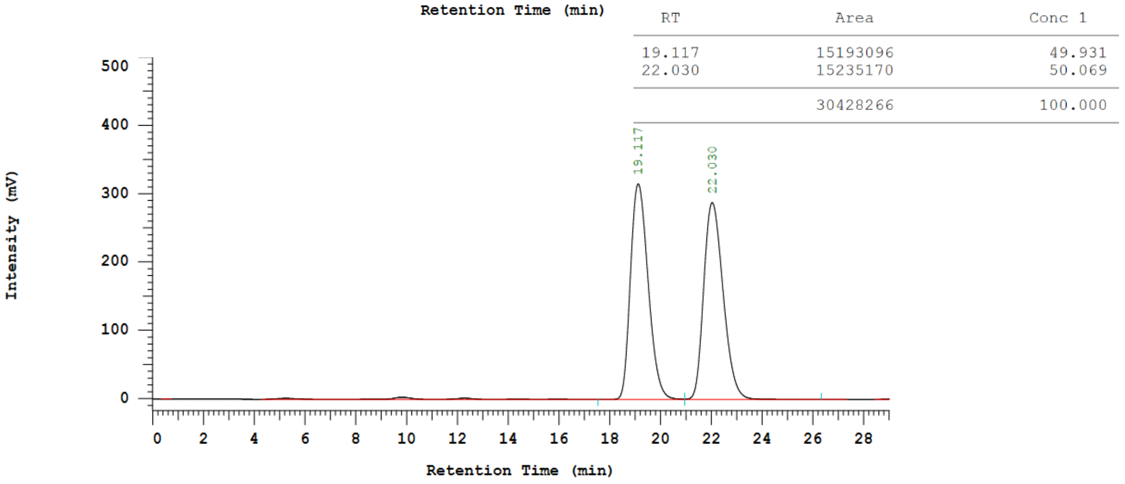

RT	Area	Conc 1
19.117	15193096	49.931
22.030	15235170	50.069
	30428266	100.000

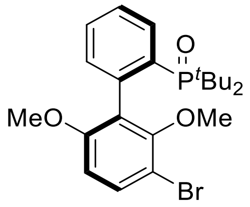

(*R*)-(3'-bromo-2',6'-dimethoxy-[1,1'-biphenyl]-2-yl)di-tert-butylphosphine oxide (3)

Appearance: colorless foam;

Yield: 97%; **e.e**: 99%; **$[\alpha]_D^{25}$** = 49 (*c* 0.1, $CHCl_3$);

^{1}H NMR (400 MHz, $CDCl_3$): δ = 7.70 – 7.62 (m, 1H), 7.55 – 7.47 (m, 1H), 7.46 – 7.35 (m, 2H), 7.26 – 7.21 (m, 1H), 6.56 (d, *J* = 8.9 Hz, 1H), 3.63 (s, 3H), 3.49 (s, 3H), 1.26 (d, *J* = 13.5 Hz, 9H), 1.21 (d, *J* = 13.3 Hz, 9H) ppm.

^{13}C NMR (101 MHz, $CDCl_3$): δ = 157.09, 154.90, 141.18 (d, *J* = 2.9 Hz), 133.15 (d, *J* = 8.9 Hz), 131.94, 131.53 (d, *J* = 11.6 Hz), 130.66 (d, *J* = 77.2 Hz), 129.83 (d, *J* = 2.6 Hz), 127.19 (d, *J* = 2.5 Hz), 125.63 (d, *J* = 11.3 Hz), 107.56, 106.98, 60.45, 55.33, 37.42 (d, *J* = 59.0 Hz), 27.52 (d, *J* = 12.0 Hz) ppm.

^{31}P NMR (162 MHz, $CDCl_3$): δ = 54.45 ppm.

HRMS (ESI$^+$): calcd $[M+Na]^+$ for $[C_{22}H_{30}BrO_3PNa]^+$: 475.10082, found 475.10089.

HPLC condition: Daicel Chiralpak IC, i-PrOH/n-hexane = 20/80, 0.8 mL/min, 254 nm UV detector; t_1= 8.2 min (major), t_2 = 12.4 min (minor).

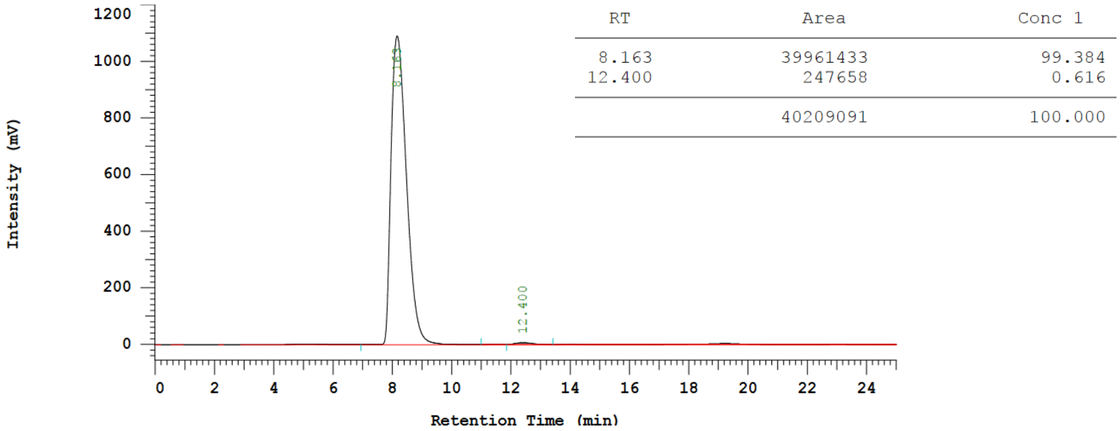

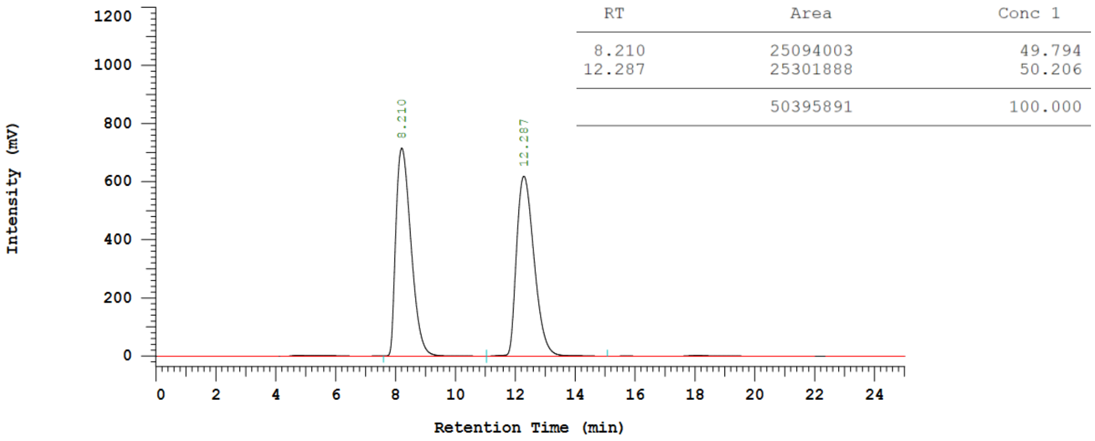

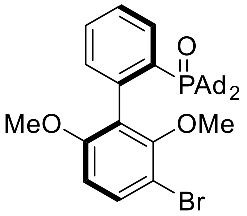

(*R*)-diadamantan-1-yl-(3'-bromo-2',6'-dimethoxy-[1,1'-biphenyl]-2-yl)phosphine oxide (4)

Appearance: colorless foam;

Yield: 95%; **e.e**: 99%; **$[\alpha]_D^{25}$** = 25 (*c* 0.1, $CHCl_3$);

^{1}H NMR (400 MHz, $CDCl_3$): δ = 7.67 – 7.59 (m, 1H), 7.52 (t, *J* = 7.5 Hz, 1H), 7.45 – 7.38 (m, 2H), 7.25 – 7.20 (m, 1H), 6.55 (d, *J* = 8.9 Hz, 1H), 3.63 (s, 3H), 3.48 (s, 3H), 2.10 – 1.86 (m, 18H), 1.75 – 1.60 (m, 12H) ppm.

^{13}C NMR (101 MHz, $CDCl_3$): δ = 156.93, 154.98, 141.60, 133.28 (d, *J* = 6.6 Hz), 131.86, 131.41 (d, *J* = 12.0 Hz), 129.78, 128.90 (d, *J* = 74.8 Hz), 127.08, 125.36 (d, *J* = 8.7 Hz), 107.58, 107.00, 60.39, 55.30, 41.66 (d, *J* = 58.3 Hz), 37.16 (d, *J* = 10.8 Hz), 36.67, 28.11 (d, *J* = 9.2 Hz) ppm.

^{31}P NMR (162 MHz, $CDCl_3$): δ = 46.61 ppm.

HRMS (ESI$^+$): calcd $[M+Na]^+$ for $[C_{34}H_{42}BrO_3PNa]^+$: 631.19472, found 631.19457.

HPLC condition: Daicel Chiralpak ID, i-PrOH/n-hexane = 10/90, 0.8 mL/min, 254 nm UV detector; t_1= 12.5 min (major), t_2 = 16.0 min (minor).

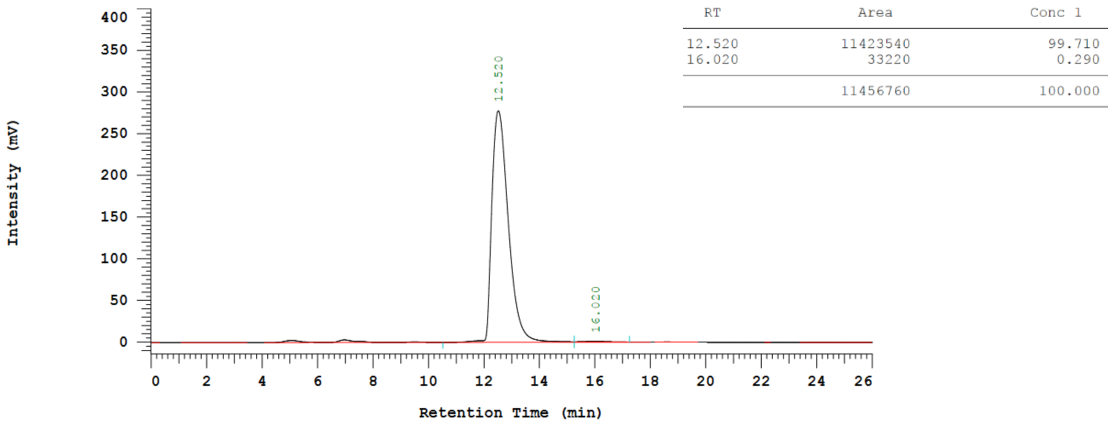

RT	Area	Conc 1
12.520	11423540	99.710
16.020	33220	0.290
	11456760	100.000

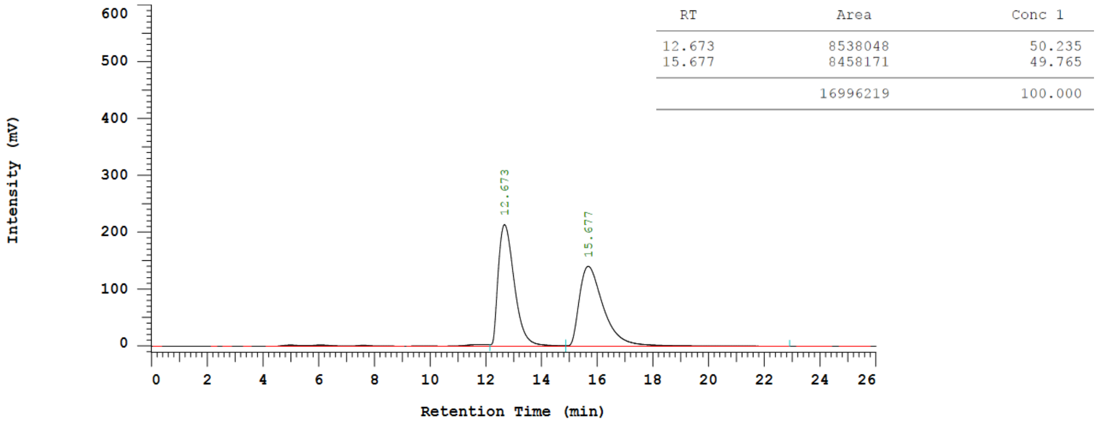

RT	Area	Conc 1
12.673	8538048	50.235
15.677	8458171	49.765
	16996219	100.000

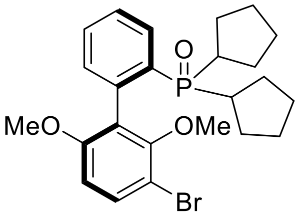

(*R*)-(3'-bromo-2',6'-dimethoxy-[1,1'-biphenyl]-2-yl)dicyclopentylphosphine oxide (5)

Appearance: colorless foam;

Yield: 93%; **e.e**: 98%; $[\alpha]_D^{25}$ = 20 (c 0.1, $CHCl_3$);

^{1}H NMR (500 MHz, $CDCl_3$): δ = 8.21 – 8.13 (m, 1H), 7.52 (d, J = 8.9 Hz, 1H), 7.51 – 7.46 (m, 2H), 7.20 – 7.13 (m, 1H), 6.60 (d, J = 9.0 Hz, 1H), 3.64 (s, 3H), 3.48 (s, 3H), 1.96 – 1.80 (m, 4H), 1.76 – 1.51 (m, 12H), 1.51 – 1.39 (m, 2H) ppm.

^{13}C NMR (126 MHz, $CDCl_3$): δ = 157.45, 155.58, 135.83 (d, J = 8.2 Hz), 133.78 (d, J = 84.5 Hz), 133.77 (d, J = 6.6 Hz), 133.07, 131.77 (d, J = 9.4 Hz), 130.15, 127.39 (d, J = 9.8 Hz), 126.35 (d, J = 2.1 Hz), 108.28, 107.38, 60.81, 55.47, 38.97 (d, J = 35.9 Hz), 38.41 (d, J = 35.8 Hz), 27.73 (d, J = 2.1 Hz), 27.41 (d, J = 1.9 Hz), 27.19 – 26.14 (m), 25.79 (d, J = 3.2 Hz), 25.70 (d, J = 3.1 Hz) ppm.

^{31}P NMR (202 MHz, $CDCl_3$): δ = 45.65 ppm.

HRMS (ESI$^+$): calcd $[M+Na]^+$ for $[C_{24}H_{30}BrO_3PNa]^+$: 499.10082, found 499.10059.

HPLC condition: Daicel Chiralpak IA, i-PrOH/n-hexane = 10/90, 0.8 mL/min, 254 nm UV detector; t_1= 14.6 min (minor), t_2 = 16.6 min (major).

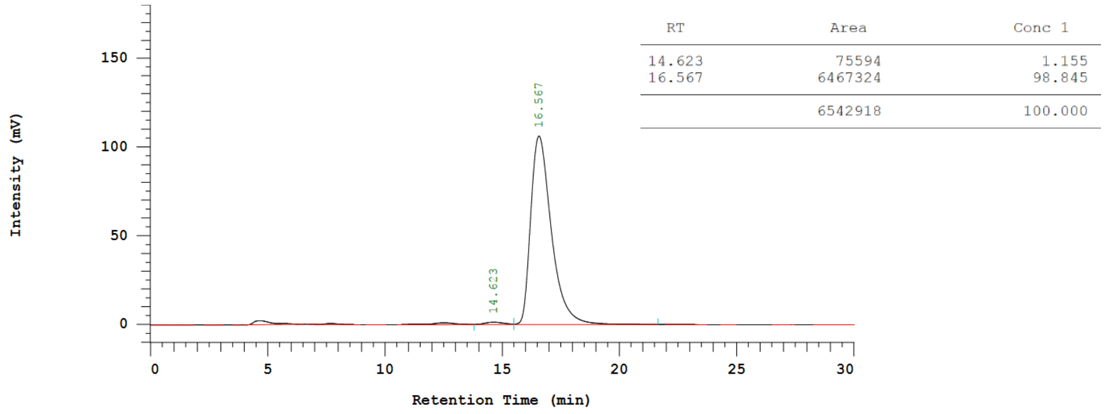

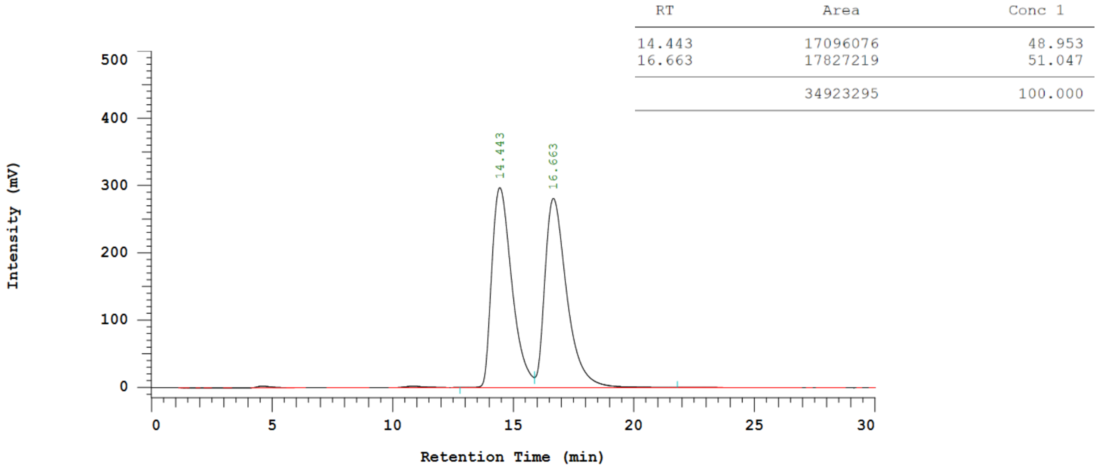

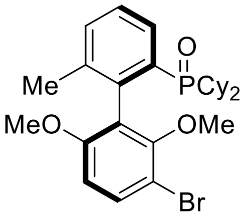

(*R*)-(3'-bromo-2',6'-dimethoxy-6-methyl-[1,1'-biphenyl]-2-yl)dicyclohexylphosphine oxide (6)

Appearance: colorless foam;

Yield: 86%; **e.e**: 98%; $[\alpha]_D^{25}$ = -21 (*c* 0.1, $CHCl_3$);

^{1}H NMR (500 MHz, $CDCl_3$): δ = 7.71 – 7.65 (m, 1H), 7.53 (d, *J* = 8.9 Hz, 1H), 7.42 – 7.32 (m, 2H), 6.62 (d, *J* = 8.9 Hz, 1H), 3.68 (s, 3H), 3.45 (s, 3H), 2.02 (s, 3H), 1.87 – 1.43 (m, 15H), 1.38 – 1.27 (m, 2H), 1.22 – 1.13 (m, 3H), 1.13 – 1.01 (m, 2H) ppm.

^{13}C NMR (126 MHz, $CDCl_3$): δ = 156.78, 154.77, 138.36 (d, *J* = 9.1 Hz), 136.78 (d, *J* = 6.4 Hz), 132.75, 132.31 (d, *J* = 2.7 Hz), 131.83 (d, *J* = 81.8 Hz), 130.51 (d, *J* = 8.2 Hz), 127.03 (d, *J* = 11.3 Hz), 124.67 (d, *J* = 2.7 Hz), 108.40, 107.41, 59.63, 55.62, 38.09 (d, *J* = 65.4 Hz), 36.99 (d, *J* = 66.1 Hz), 26.92 – 26.52 (m), 26.25 (d, *J* = 3.4 Hz), 26.07 – 25.40 (m), 20.56 ppm.

^{31}P NMR (202 MHz, $CDCl_3$): δ = 47.31 ppm.

HRMS (ESI$^+$): calcd $[M+Na]^+$ for $[C_{27}H_{36}BrO_3PNa]^+$: 541.14777, found 541.14736.

HPLC condition: Daicel Chiralpak IC, i-PrOH/n-hexane = 10/90, 0.8 mL/min, 254 nm UV detector; t_1= 32.0 min (major), t_2 = 38.3 min (minor).

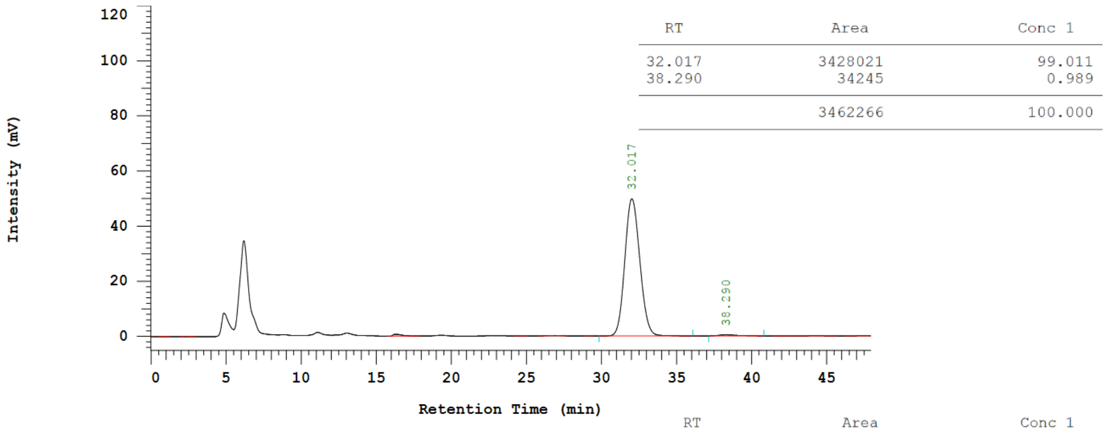

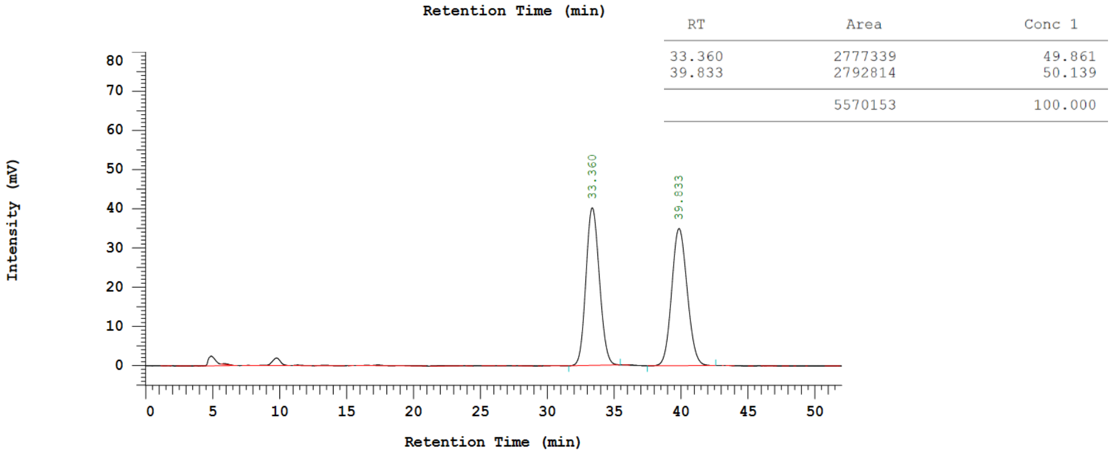

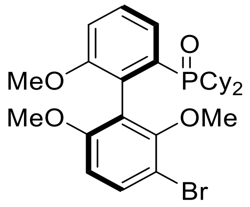

(*R*)-(3'-bromo-2',6,6'-trimethoxy-[1,1'-biphenyl]-2-yl)dicyclohexylphosphine oxide (7)

Appearance: colorless foam;

Yield: 88%; **e.e**: 96%; **[α]$_D^{25}$** = 31 (*c* 0.1, $CHCl_3$);

^{1}H NMR (500 MHz, $CDCl_3$): δ = 7.62 – 7.56 (m, 1H), 7.52 (d, *J* = 8.8 Hz, 1H), 7.45 (td, *J* = 8.0, 2.5 Hz, 1H), 7.09 (d, *J* = 8.2 Hz, 1H), 6.62 (d, *J* = 8.9 Hz, 1H), 3.71 (s, 3H), 3.66 (s, 3H), 3.46 (s, 3H), 1.83 – 1.53 (m, 10H), 1.52 – 1.41 (m, 4H), 1.33 – 1.22 (m, 3H), 1.21 – 1.09 (m, 3H), 1.06 – 0.94 (m, 2H) ppm.

^{13}C NMR (126 MHz, $CDCl_3$): δ = 157.85, 157.48 (d, *J* = 12.8 Hz), 155.51, 133.58 (d, *J* = 80.8 Hz), 132.71, 128.43 (d, *J* = 11.8 Hz), 125.93 (d, *J* = 8.5 Hz), 125.59 (d, *J* = 6.9 Hz), 121.94 (d, *J* = 2.7 Hz), 113.18 (d, *J* = 2.5 Hz), 108.18, 107.47, 59.82, 55.80 (d, *J* = 3.8 Hz), 37.84 (d, *J* = 32.7 Hz), 37.31 (d, *J* = 32.6 Hz), 26.89 – 26.29 (m), 26.15 – 25.58 (m) ppm.

^{31}P NMR (202 MHz, $CDCl_3$): δ = 47.86 ppm.

HRMS (ESI$^+$): calcd [M+Na]$^+$ for [$C_{27}H_{36}BrO_4PNa$]$^+$: 557.14268, found 557.14269.

HPLC condition: Daicel Chiralpak IC, i-PrOH/n-hexane = 10/90, 0.8 mL/min, 254 nm UV detector; t_1= 52.8 min (major), t_2 = 62.0 min (minor).

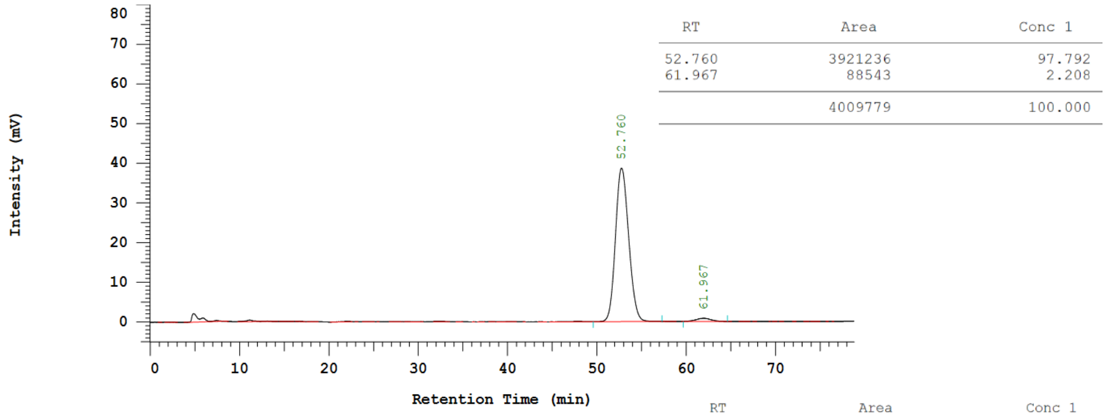

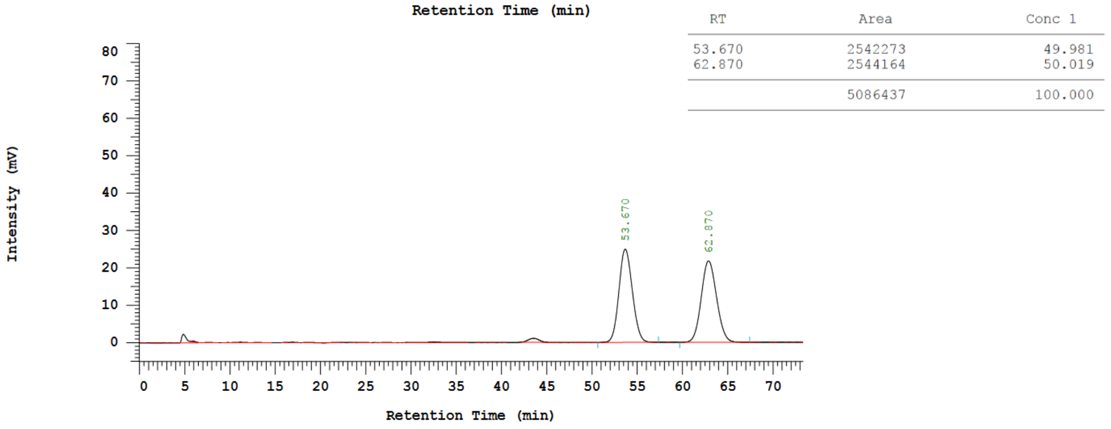

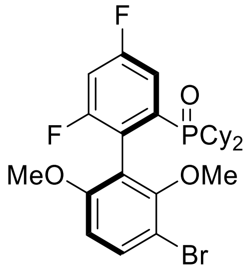

(R)-(3'-bromo-4,6-difluoro-2',6'-dimethoxy-[1,1'-biphenyl]-2-yl)dicyclohexylphosphine oxide (8)

Appearance: colorless foam;

Yield: 73%; **e.e**: 98%; $[\alpha]_D^{25}$ = 17 (c 0.1, $CHCl_3$);

^{1}H NMR (500 MHz, $CDCl_3$): δ = 7.70 – 7.63 (m, 1H), 7.60 (d, J = 8.8 Hz, 1H), 7.05 – 6.97 (m, 1H), 6.67 (d, J = 8.8 Hz, 1H), 3.70 (s, 3H), 3.52 (s, 3H), 1.82 – 1.60 (m, 8H), 1.59 – 1.40 (m, 5H), 1.40 – 1.22 (m, 4H), 1.22 – 1.08 (m, 3H), 1.04 – 0.91 (m, 2H) ppm.

^{13}C NMR (126 MHz, $CDCl_3$): δ = 163.40 – 162.78 (m), 161.60 – 160.81 (m), 159.43 (dd, J = 15.9, 10.6 Hz), 157.85, 155.94, 136.94 – 136.10 (m), 134.20, 129.16 (d, J = 140.3 Hz), 118.04, 117.07 (d, J = 22.6 Hz), 108.26, 107.80, 106.31 (t, J = 26.2 Hz), 60.39, 56.02, 37.78 (d, J = 12.8 Hz), 37.26 (d, J = 12.9 Hz), 26.80 – 26.06 (m), 25.94 – 25.45 (m) ppm.

^{19}F NMR (471 MHz, $CDCl_3$): δ = -105.86 (d, J = 8.7 Hz, 1F), -108.86 (d, J = 8.9 Hz, 1F) ppm.

^{31}P NMR (202 MHz, $CDCl_3$): δ = 50.45 ppm.

HRMS (ESI$^+$): calcd $[M+Na]^+$ for $[C_{26}H_{32}BrF_2O_3PNa]^+$: 563.11327, found 563.11309.

HPLC condition: Daicel Chiralpak IC, i-PrOH/n-hexane = 10/90, 0.8 mL/min, 254 nm UV detector; t_1= 23.8 min (minor), t_2 = 26.6 min (major).

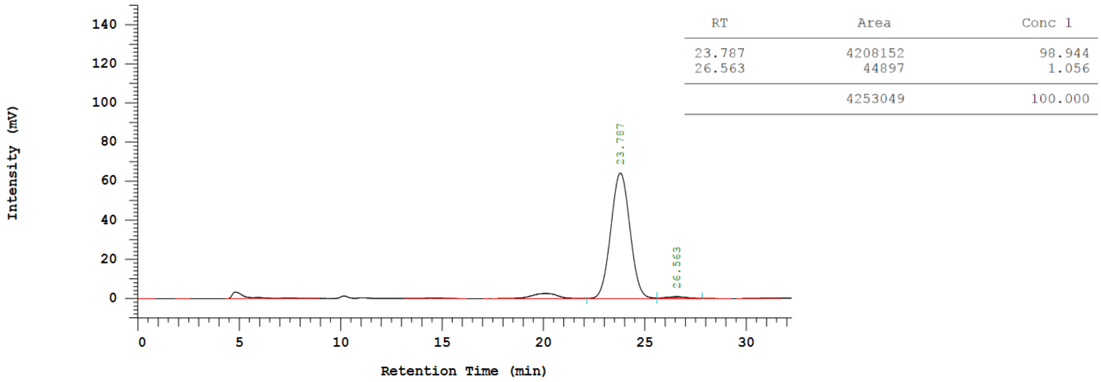

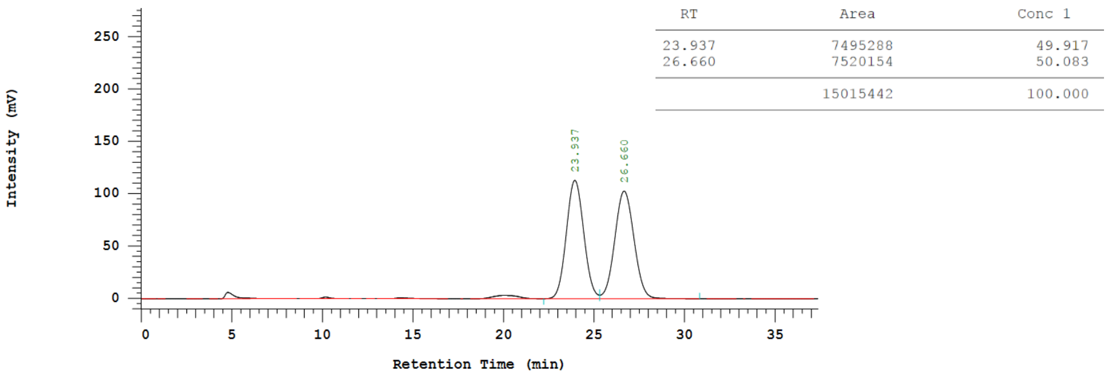

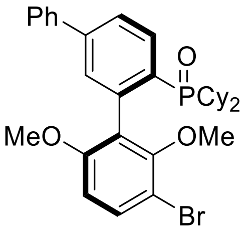

(*R*)-(3''-bromo-2'',6''-dimethoxy-[1,1':3',1''-terphenyl]-4'-yl)dicyclohexylphosphine oxide (9)

Appearance: colorless foam;

Yield: 93%; **e.e**: 92%; $[\alpha]_D^{25}$ = 96 (*c* 0.1, $CHCl_3$);

^{1}H NMR (500 MHz, $CDCl_3$): δ = 8.00 – 7.93 (m, 1H), 7.70 (dt, *J* = 8.1, 1.7 Hz, 1H), 7.66 – 7.61 (m, 2H), 7.54 (d, *J* = 8.9 Hz, 1H), 7.47 – 7.41 (m, 3H), 7.40 – 7.33 (m, 1H), 6.64 (d, *J* = 9.0 Hz, 1H), 3.69 (s, 3H), 3.54 (s, 3H), 1.87 – 1.69 (m, 8H), 1.67 – 1.44 (m, 6H), 1.39 – 1.14 (m, 6H), 1.14 – 0.99 (m, 2H) ppm.

^{13}C NMR (126 MHz, $CDCl_3$): δ = 157.37, 155.20, 142.58 (d, *J* = 2.7 Hz), 139.71, 133.67 (d, *J* = 8.1 Hz), 132.77, 130.99, 130.91, 130.64 (d, *J* = 82.4 Hz), 128.87, 127.97, 127.19, 126.57, 125.50 (d, *J* = 10.7 Hz), 108.22, 107.53, 60.67, 55.73, 37.88 (d, *J* = 51.2 Hz), 37.36 (d, *J* = 51.2 Hz), 26.92 – 26.20 (m), 26.05 – 25.61 (m) ppm.

^{31}P NMR (202 MHz, $CDCl_3$): δ = 47.27 ppm.

HRMS (ESI$^+$): calcd $[M+Na]^+$ for $[C_{32}H_{38}BrO_3PNa]^+$: 603.16342, found 603.16304.

HPLC condition: Daicel Chiralpak ID, i-PrOH/n-hexane = 15/85, 0.8 mL/min, 254 nm UV detector; t_1= 25.6 min (minor), t_2 = 30.5 min (major).

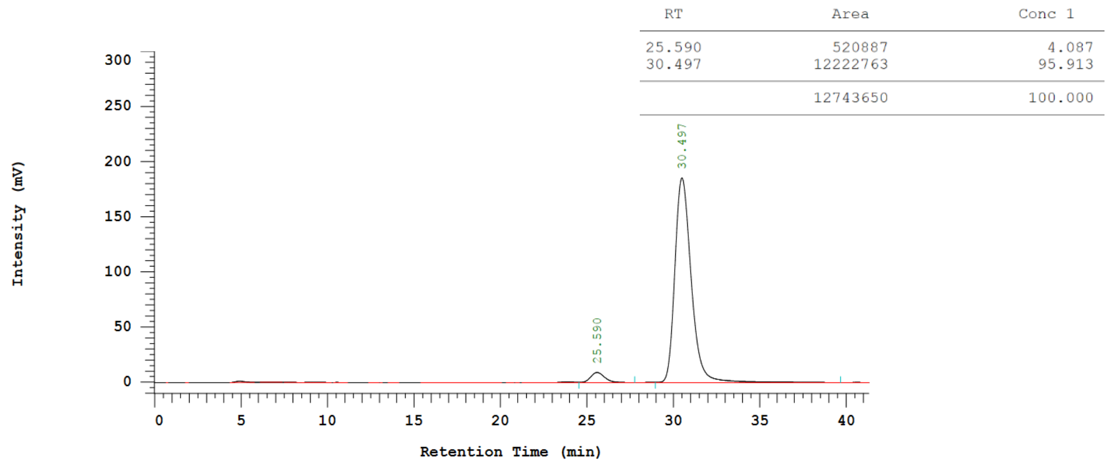

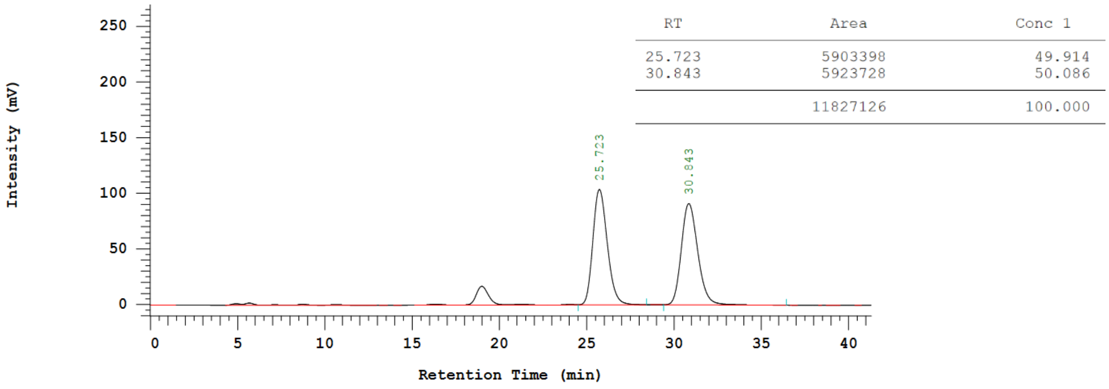

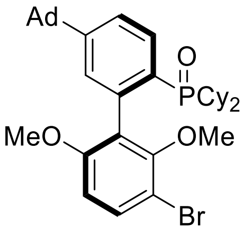

((*R*)-5-(-adamantan-1-yl)-3'-bromo-2',6'-dimethoxy-[1,1'-biphenyl]-2-yl)dicyclohexylphosphine oxide (10)

Appearance: colorless foam;

Yield: 90%; **e.e**: 95%; $[\alpha]_D^{25}$ = 57 (*c* 0.1, $CHCl_3$);

^{1}H NMR (500 MHz, $CDCl_3$): δ = 7.80 (dd, *J* = 10.7, 8.2 Hz, 1H), 7.50 (d, *J* = 8.9 Hz, 1H), 7.39 (dt, *J* = 8.2, 1.8 Hz, 1H), 7.15 (dd, *J* = 3.5, 2.0 Hz, 1H), 6.61 (d, *J* = 8.9 Hz, 1H), 3.66 (s, 3H), 3.46 (s, 3H), 2.08 (s, 3H), 1.92 (d, *J* = 2.9 Hz, 6H), 1.83 – 1.61 (m, 14H), 1.60 – 1.41 (m, 6H), 1.35 – 1.12 (m, 6H), 1.12 – 0.98 (m, 2H) ppm.

^{13}C NMR (126 MHz, $CDCl_3$): δ = 157.45, 155.17, 153.17 (d, *J* = 2.6 Hz), 136.64 (d, *J* = 6.9 Hz), 132.88 (d, *J* = 8.0 Hz), 132.40, 129.46 (d, *J* = 9.5 Hz), 128.53 (d, *J* = 84.1 Hz), 127.36 (d, *J* = 2.0 Hz), 123.37 (d, *J* = 10.2 Hz), 108.25, 107.56, 60.53, 55.71, 42.89, 37.82 (d, *J* = 44.3 Hz), 37.29 (d, *J* = 44.4 Hz), 36.71, 36.24, 28.84, 26.95 – 26.37 (m), 26.24 (d, *J* = 3.2 Hz), 26.08 – 25.61 (m) ppm.

^{31}P NMR (202 MHz, $CDCl_3$): δ = 46.73 ppm.

HRMS (ESI$^+$): calcd $[M+Na]^+$ for $[C_{36}H_{48}BrO_3PNa]^+$: 661.24167, found 661.24106.

HPLC condition: Daicel Chiralpak ID, i-PrOH/n-hexane = 10/90, 0.8 mL/min, 254 nm UV detector; t_1= 30.3 min (minor), t_2 = 33.2 min (major).

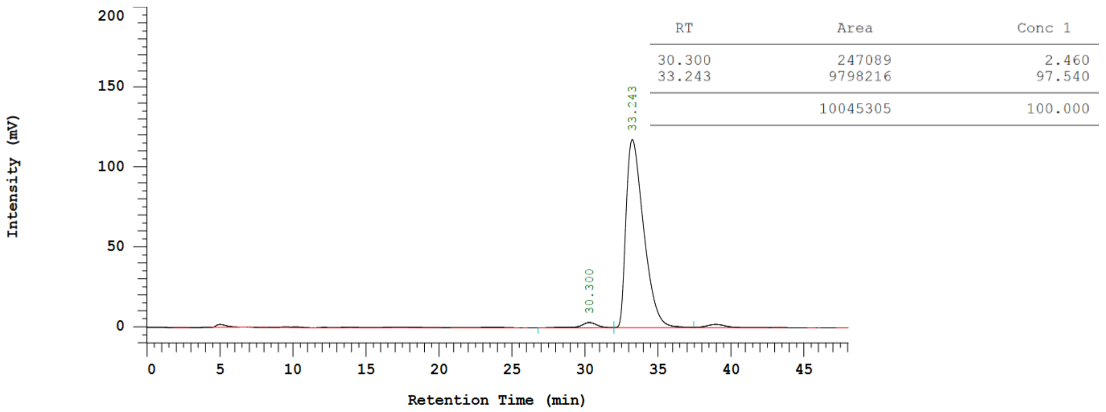

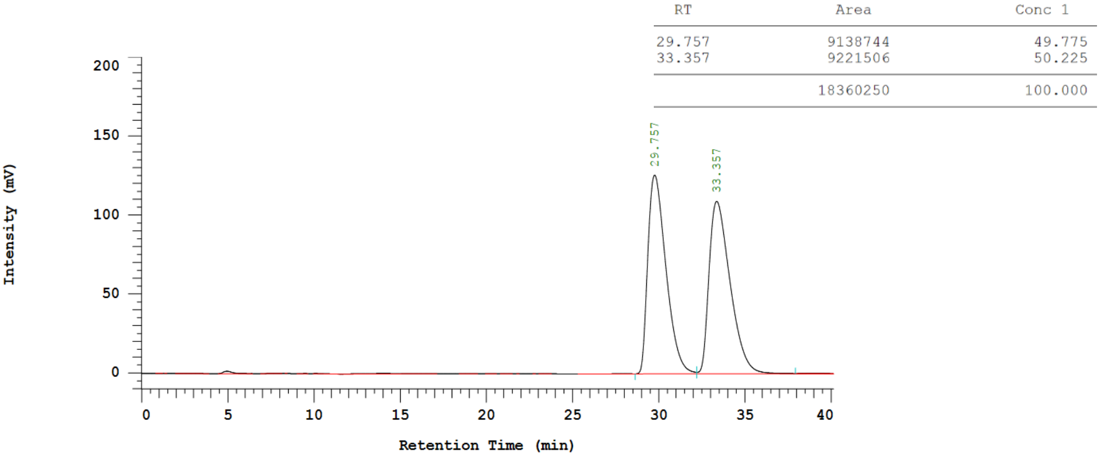

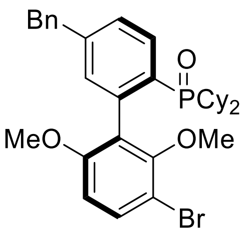

(R)-(5-benzyl-3'-bromo-2',6'-dimethoxy-[1,1'-biphenyl]-2-yl)dicyclohexylphosphine oxide (11)

Appearance: colorless foam;

Yield: 88%; **e.e**: 96%; $[\alpha]_D^{25}$ = 53 (c 0.1, $CHCl_3$);

^{1}H NMR (500 MHz, $CDCl_3$): δ = 7.80 (dd, J = 10.8, 8.0 Hz, 1H), 7.49 (d, J = 8.9 Hz, 1H), 7.30 – 7.23 (m, 3H), 7.22 – 7.15 (m, 3H), 7.02 – 6.97 (m, 1H), 6.59 (d, J = 8.9 Hz, 1H), 4.03 (s, 2H), 3.67 (s, 3H), 3.36 (s, 3H), 1.82 – 1.61 (m, 8H), 1.60 – 1.40 (m, 6H), 1.34 – 1.12 (m, 6H), 1.11 – 0.94 (m, 2H) ppm.

^{13}C NMR (126 MHz, $CDCl_3$): δ = 157.33, 155.13, 143.26 (d, J = 2.6 Hz), 140.35, 137.28 (d, J = 6.8 Hz), 133.32 (d, J = 8.1 Hz), 133.01 (d, J = 9.6 Hz), 132.59, 129.47 (d, J = 83.1 Hz), 129.04, 128.50, 127.62 (d, J = 10.5 Hz), 126.67 (d, J = 1.9 Hz), 126.29, 108.20, 107.49, 60.43, 55.72, 41.60, 37.81 (d, J = 46.0 Hz), 37.28 (d, J = 46.1 Hz), 27.03 – 26.33 (m), 26.22 (d, J = 3.2 Hz), 26.09 – 25.68 (m) ppm.

^{31}P NMR (202 MHz, $CDCl_3$): δ = 46.92 ppm.

HRMS (ESI$^+$): calcd $[M+Na]^+$ for $[C_{33}H_{40}BrO_3PNa]^+$: 617.17907, found 617.17856.

HPLC condition: Daicel Chiralpak IA, i-PrOH/n-hexane = 10/90, 0.8 mL/min, 254 nm UV detector; t_1= 17.3 min (minor), t_2 = 20.5 min (major).

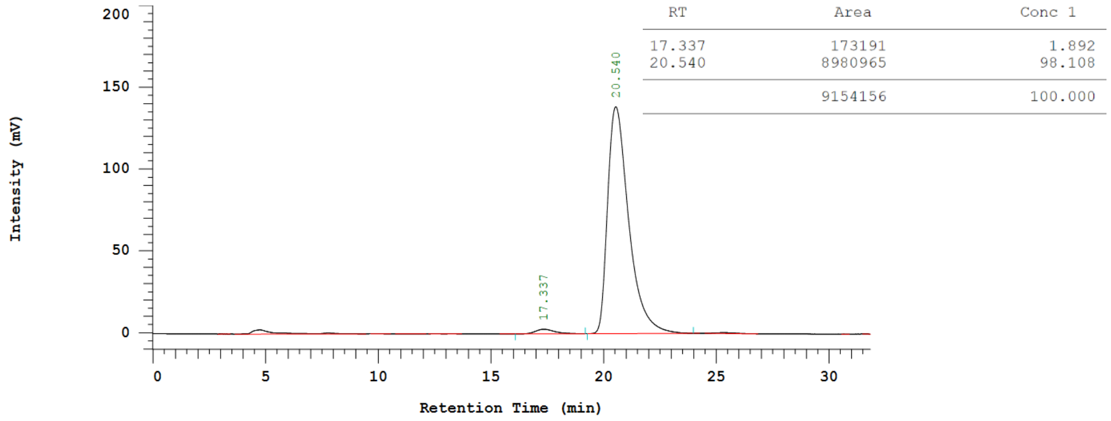

RT	Area	Conc 1
17.337	173191	1.892
20.540	8980965	98.108
	9154156	100.000

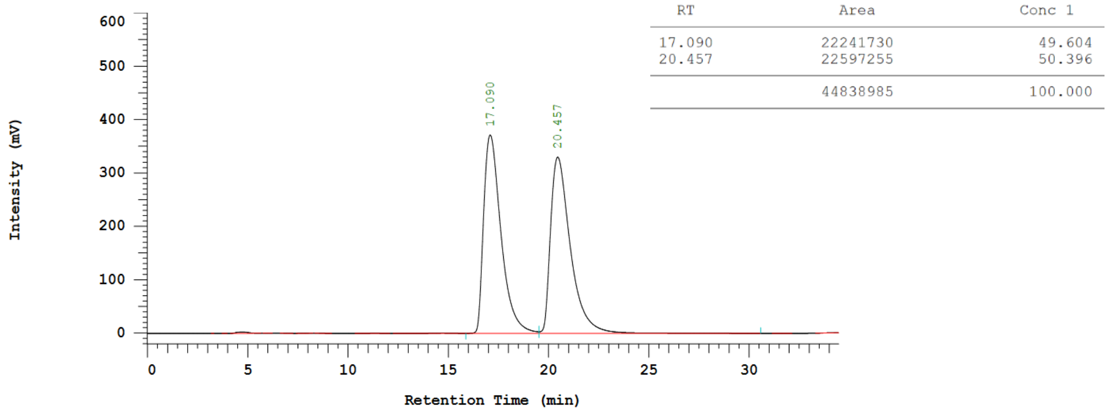

RT	Area	Conc 1
17.090	22241730	49.604
20.457	22597255	50.396
	44838985	100.000

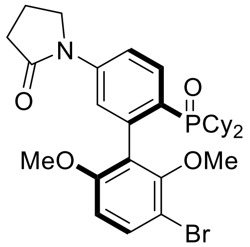

(*R*)-1-(3'-bromo-6-(dicyclohexylphosphoryl)-2',6'-dimethoxy-[1,1'-biphenyl]-3-yl)pyrrolidin-2-one (12)

Appearance: colorless foam;

Yield: 84%; **e.e**: 93%; **$[\alpha]_D^{25}$** = 25 (*c* 0.1, $CHCl_3$);

^{1}H NMR (500 MHz, $CDCl_3$): δ = 7.85 (t, *J* = 9.5 Hz, 1H), 7.70 (d, *J* = 8.6 Hz, 1H), 7.61 – 7.58 (m, 1H), 7.51 (d, *J* = 8.9 Hz, 1H), 6.61 (d, *J* = 9.0 Hz, 1H), 3.97 – 3.81 (m, 2H), 3.67 (s, 3H), 3.57 (s, 3H), 2.61 (t, *J* = 8.1 Hz, 2H), 2.21 – 2.11 (m, 2H), 1.85 – 1.61 (m, 8H), 1.61 – 1.38 (m, 4H), 1.35 – 0.97 (m, 10H) ppm.

^{13}C NMR (126 MHz, $CDCl_3$): δ = 174.66, 157.31, 155.13, 140.95 (d, *J* = 2.7 Hz), 138.18 (d, *J* = 7.3 Hz), 133.74 (d, *J* = 8.4 Hz), 132.82, 128.64 (d, *J* = 101.7 Hz), 126.34 (d, *J* = 2.0 Hz), 125.31, 122.83 (d, *J* = 9.3 Hz), 117.26 (d, *J* = 10.4 Hz), 108.20, 107.50, 60.80, 55.75, 48.39, 37.81 (d, *J* = 60.9 Hz), 37.29 (d, *J* = 60.9 Hz), 32.96, 26.84, 26.79 – 26.57 (m), 26.52, 26.45 (d, *J* = 3.2 Hz), 26.20 (d, *J* = 3.2 Hz), 25.95, 25.85 – 25.68 (m), 17.95 ppm.

^{31}P NMR (202 MHz, $CDCl_3$): δ = 46.34 ppm.

HRMS (ESI$^+$): calcd $[M+Na]^+$ for $[C_{30}H_{39}BrNO_4PNa]^+$: 610.16923, found 610.16918.

HPLC condition: Daicel Chiralpak IF, i-PrOH/n-hexane = 20/80, 1.0 mL/min, 254 nm UV detector; t_1= 43.4 min (minor), t_2 = 49.3 min (major).

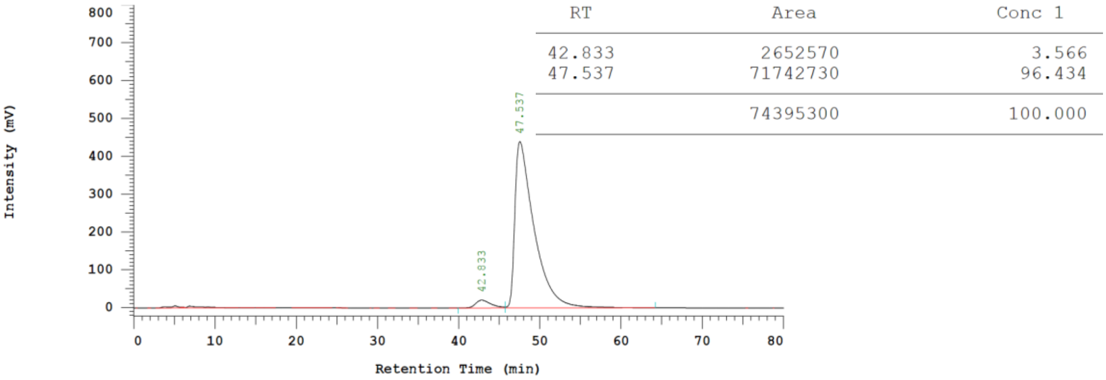

RT	Area	Conc 1
42.833	2652570	3.566
47.537	71742730	96.434
	74395300	100.000

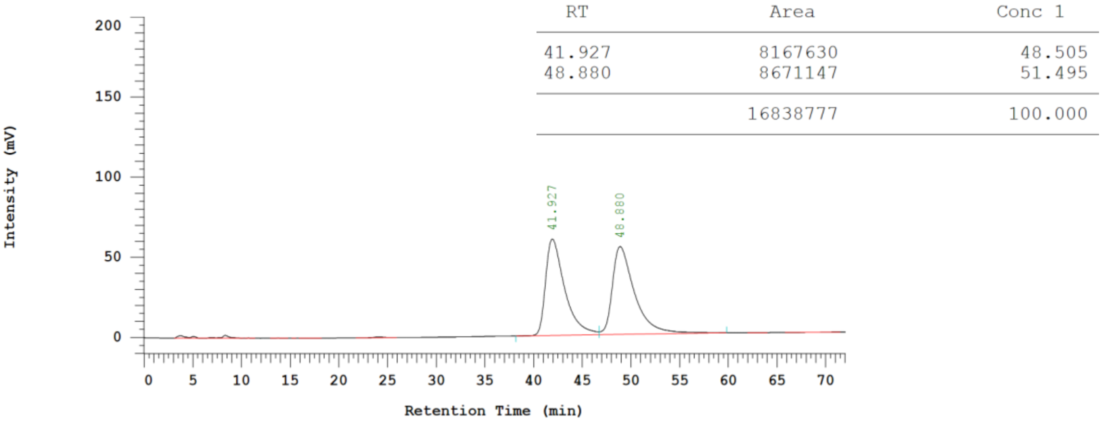

RT	Area	Conc 1
41.927	8167630	48.505
48.880	8671147	51.495
	16838777	100.000

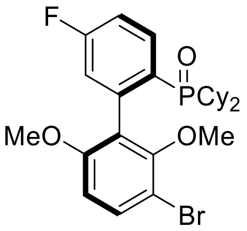

(R)-(3'-bromo-5-fluoro-2',6'-dimethoxy-[1,1'-biphenyl]-2-yl)dicyclohexylphosphine oxide (13)

Appearance: colorless foam;

Yield: 76%; **e.e**: 98%; $[\alpha]_D^{25}$ = 16 (c 0.1, $CHCl_3$);

^{1}H NMR (500 MHz, $CDCl_3$): δ = 7.96 – 7.88 (m, 1H), 7.53 (d, J = 8.8 Hz, 1H), 7.19 – 7.12 (m, 1H), 6.91 (dt, J = 9.4, 2.7 Hz, 1H), 6.62 (d, J = 8.9 Hz, 1H), 3.68 (s, 3H), 3.51 (s, 3H), 1.81 – 1.60 (m, 8H), 1.59 – 1.39 (m, 6H), 1.33 – 1.20 (m, 3H), 1.20 – 1.07 (m, 3H), 1.06 – 0.91 (m, 2H) ppm.

^{13}C NMR (126 MHz, $CDCl_3$): δ = 163.62 (dd, J = 252.4, 3.0 Hz), 157.09, 155.05, 139.79 (t, J = 8.4 Hz), 135.69 (t, J = 8.7 Hz), 133.30, 129.15 (d, J = 141.8 Hz), 127.47 (d, J = 83.6 Hz), 125.22, 119.45 (dd, J = 20.9, 10.4 Hz), 114.37 (dd, J = 20.2, 11.1 Hz), 108.16, 107.67, 60.67, 55.80, 37.79 (d, J = 41.8 Hz), 37.27 (d, J = 41.9 Hz), 26.83 – 26.16 (m), 25.97 – 25.49 (m) ppm.

^{19}F NMR (471 MHz, $CDCl_3$): δ = -110.2 (s, 1F) ppm.

^{31}P NMR (202 MHz, $CDCl_3$): δ = 48.54 ppm.

HRMS (ESI$^+$): calcd $[M+Na]^+$ for $[C_{26}H_{33}BrFO_3PNa]^+$: 545.12269, found 545.12291.

HPLC condition: Daicel Chiralpak IA, i-PrOH/n-hexane = 5/95, 0.8 mL/min, 254 nm UV detector; t_1= 35.7 min (minor), t_2 = 39.6 min (major).

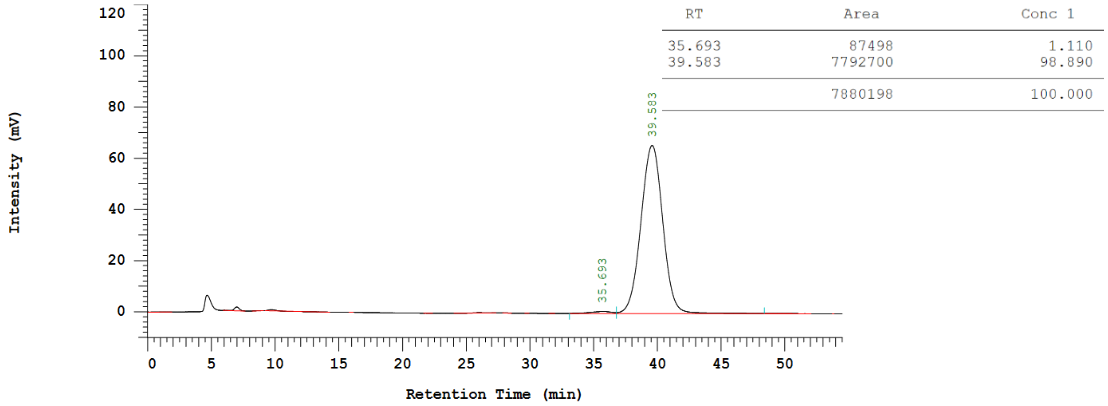

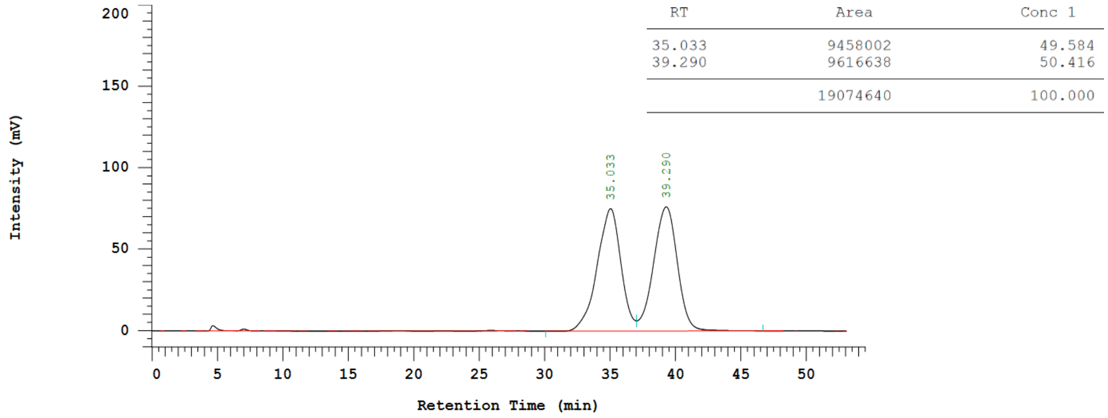

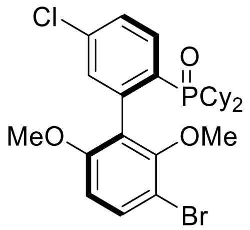

(R)-(3'-bromo-5-chloro-2',6'-dimethoxy-[1,1'-biphenyl]-2-yl)dicyclohexylphosphine oxide (14)

Appearance: colorless foam;

Yield: 85%; **e.e**: 97%; **$[\alpha]_D^{25}$** = 18.0 (c 0.1, $CHCl_3$);

^{1}H NMR (500 MHz, $CDCl_3$): δ = 7.87 – 7.80 (m, 1H), 7.51 (d, J = 8.9 Hz, 1H), 7.41 (d, J = 10.3 Hz, 1H), 7.18 (t, J = 2.6 Hz, 1H), 6.61 (d, J = 8.9 Hz, 1H), 3.67 (s, 3H), 3.51 (s, 3H), 1.82 – 1.58 (m, 8H), 1.59 – 1.34 (m, 6H), 1.32 – 1.11 (m, 6H), 1.13 – 0.93 (m, 2H) ppm.

^{13}C NMR (126 MHz, $CDCl_3$): δ = 157.10, 155.07, 138.91 (d, J = 7.4 Hz), 136.35 (d, J = 3.1 Hz), 134.58 (d, J = 8.5 Hz), 133.22, 132.16 (d, J = 9.7 Hz), 130.67 (d, J = 81.3 Hz), 127.28 (d, J = 10.6 Hz), 125.20 (d, J = 2.0 Hz), 108.10, 107.57, 60.64, 55.76, 37.79 (d, J = 50.9 Hz), 37.26 (d, J = 50.9 Hz), 26.81 – 26.13 (m), 26.02 – 25.46 (m) ppm.

^{31}P NMR (202 MHz, $CDCl_3$): δ = 47.27 ppm.

HRMS (ESI$^+$): calcd $[M+Na]^+$ for $[C_{26}H_{33}BrClO_3PNa]^+$: 561.09314, found 561.09304.

HPLC condition: Daicel Chiralpak IA, i-PrOH/n-hexane = 5/95, 0.8 mL/min, 254 nm UV detector; t_1= 45.8 min (minor), t_2 = 54.7 min (major).

RT	Area	Conc 1
45.777	28415	1.260
54.670	2226784	98.740
	2255199	100.000

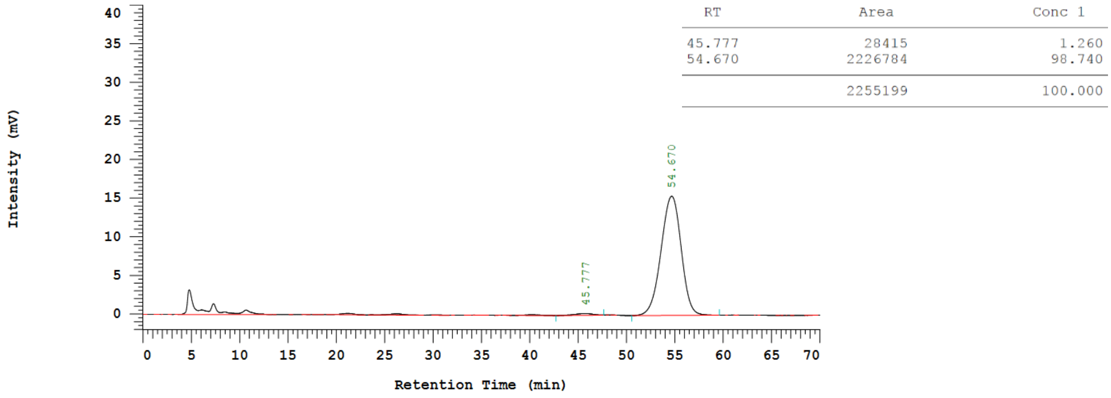

RT	Area	Conc 1
44.130	12017999	49.938
53.407	12047700	50.062
	24065699	100.000

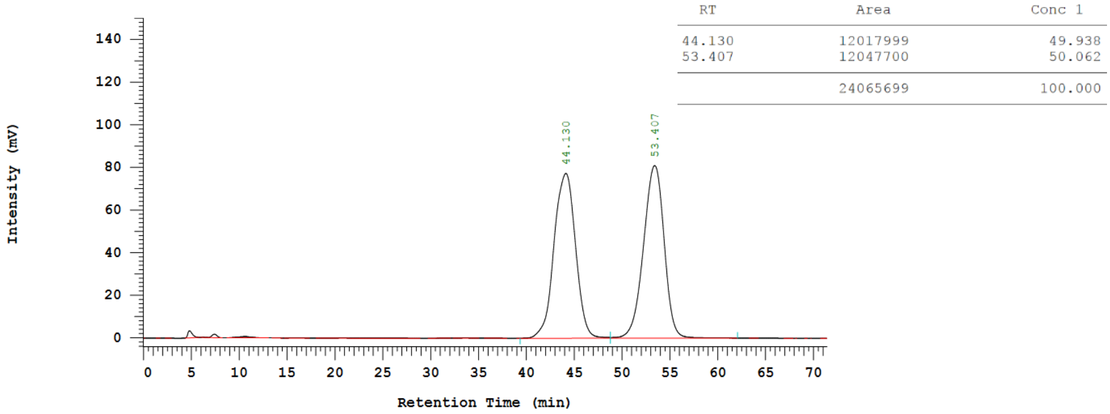

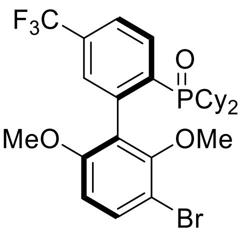

(*R*)-(3'-bromo-2',6'-dimethoxy-5-(trifluoromethyl)-[1,1'-biphenyl]-2-yl)dicyclohexylphosphine oxide (15)

Appearance: colorless foam;

Yield: 68%; **e.e**: 98%; $[\alpha]_D^{25}$ = 20 (*c* 0.1, $CHCl_3$);

^{1}H NMR (500 MHz, $CDCl_3$): δ = 8.09 (t, *J* = 9.2 Hz, 1H), 7.71 (d, *J* = 7.8 Hz, 1H), 7.56 (d, *J* = 8.8 Hz, 1H), 7.44 (s, 1H), 6.65 (d, *J* = 8.8 Hz, 1H), 3.69 (s, 3H), 3.48 (s, 3H), 1.87 – 1.60 (m, 8H), 1.60 – 1.40 (m, 6H), 1.36 – 1.26 (m, 2H), 1.22 – 1.11 (m, 4H), 1.11 – 0.91 (m, 2H) ppm.

^{13}C NMR (126 MHz, $CDCl_3$): δ = 157.15, 155.18, 138.10 (d, *J* = 7.4 Hz), 136.33 (d, *J* = 78.3 Hz), 134.01 (d, *J* = 7.9 Hz), 133.56, 132.21 (q, *J* = 32.8 Hz), 129.02 (dd, *J* = 9.3, 4.1 Hz), 125.06 (d, *J* = 1.8 Hz), 123.75 (dd, *J* = 10.0, 3.9 Hz), 123.70 (q, *J* = 272.6 Hz), 108.16, 107.75, 60.62, 55.85, 37.73 (d, *J* = 38.6 Hz), 37.21 (d, *J* = 38.7 Hz), 26.88 – 26.04 (m), 25.83 – 25.47 (m) ppm.

^{19}F NMR (471 MHz, $CDCl_3$): δ = -62.91 ppm.

^{31}P NMR (202 MHz, $CDCl_3$): δ = 48.91 ppm.

HRMS (ESI$^+$): calcd $[M+Na]^+$ for $[C_{27}H_{33}BrF_3O_3PNa]^+$: 595.11950, found 595.11939.

HPLC condition: Daicel Chiralpak ID, i-PrOH/n-hexane = 10/90, 0.8 mL/min, 254 nm UV detector; t_1= 18.3 min (minor), t_2 = 20.2 min (major).

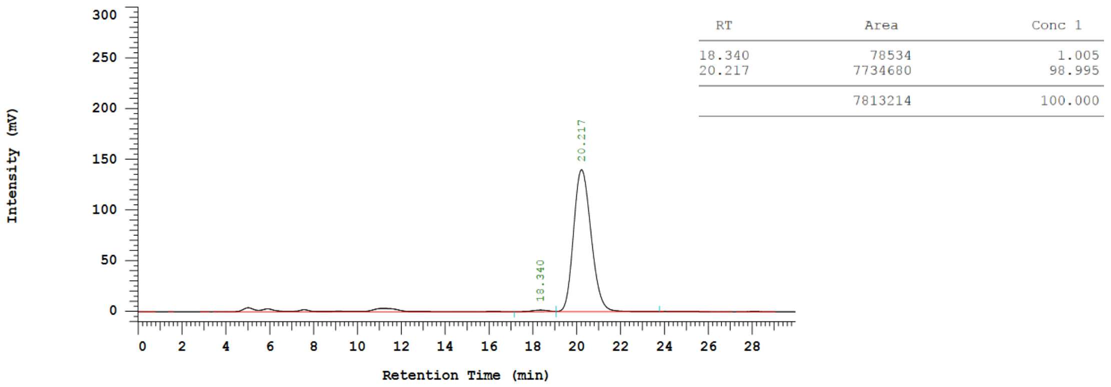

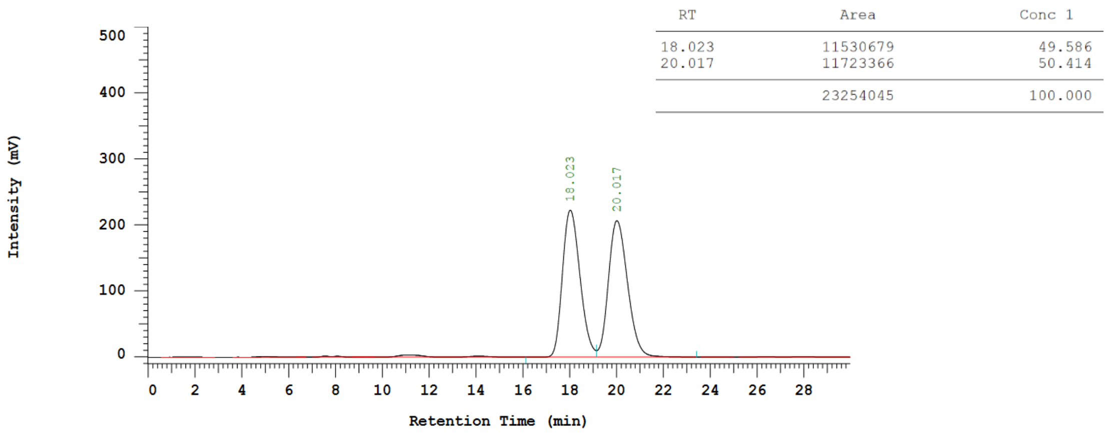

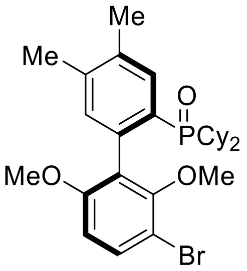

(*R*)-(3'-bromo-2',6'-dimethoxy-4,5-dimethyl-[1,1'-biphenyl]-2-yl)dicyclohexylphosphine oxide (16)

Appearance: colorless foam;

Yield: 96%; **e.e**: 96%; $[\alpha]_D^{25}$ = 31 (*c* 0.1, $CHCl_3$);

^{1}H NMR (400 MHz, $CDCl_3$): δ = 7.66 (d, *J* = 11.1 Hz, 1H), 7.49 (d, *J* = 8.9 Hz, 1H), 6.95 (d, *J* = 3.9 Hz, 1H), 6.60 (d, *J* = 8.9 Hz, 1H), 3.68 (s, 3H), 3.49 (s, 3H), 2.35 (s, 3H), 2.29 (s, 3H), 1.86 – 1.41 (m, 12H), 1.37 – 0.92 (m, 10H) ppm.

^{13}C NMR (101 MHz, $CDCl_3$): δ = 157.44, 155.23, 139.06, 135.63 (d, *J* = 10.0 Hz), 134.35, 134.25 (d, *J* = 7.9 Hz), 134.12 (d, *J* = 7.3 Hz), 133.61 (d, *J* = 9.9 Hz), 132.46, 126.63, 108.23, 107.43, 60.58, 55.73, 37.85 (d, *J* = 43.3 Hz), 37.20 (d, *J* = 43.3 Hz), 29.67, 26.91 – 26.45 (m), 26.34 (d, *J* = 3.3 Hz), 26.02 – 25.81 (m), 25.78 – 25.53 (m), 19.68 (d, *J* = 6.6 Hz) ppm.

^{31}P NMR (162 MHz, $CDCl_3$): δ = 48.46 ppm.

HRMS (ESI$^+$): calcd $[M+Na]^+$ for $[C_{28}H_{38}BrO_3PNa]^+$: 555.16342, found 555.16356.

HPLC condition: Daicel Chiralpak IA, i-PrOH/n-hexane = 10/90, 0.8 mL/min, 254 nm UV detector; t_1= 11.3 min (minor), t_2 = 14.1 min (major).

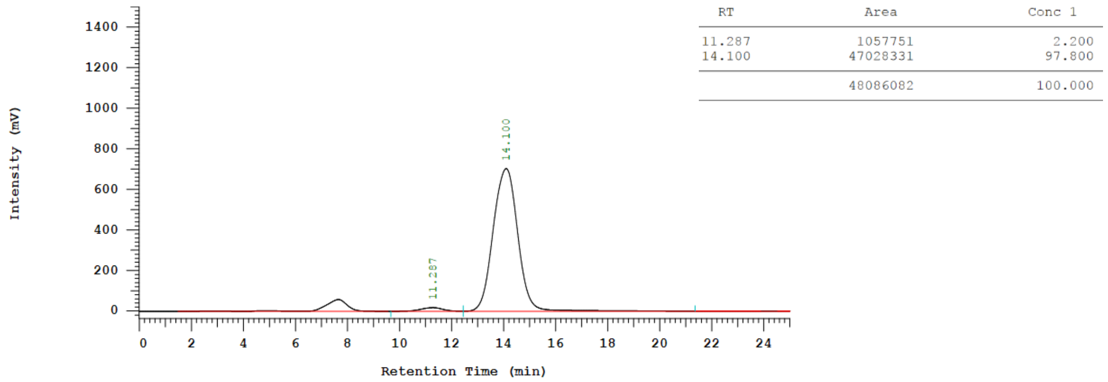

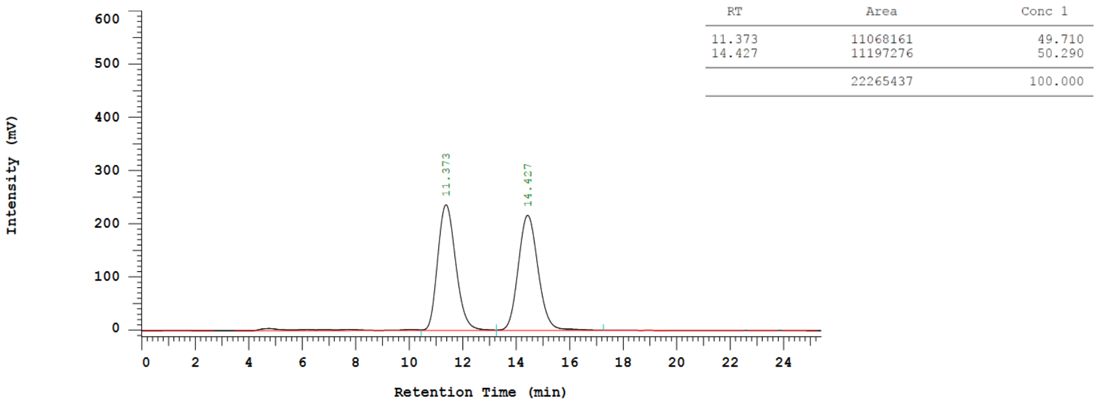

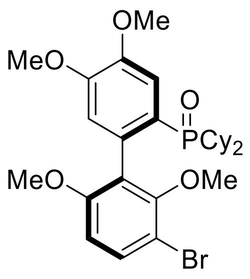

(*R*)-(3'-bromo-2',4,5,6'-tetramethoxy-[1,1'-biphenyl]-2-yl)dicyclohexylphosphine oxide (17)

Appearance: colorless foam;

Yield: 86%; **e.e**: 97%; $[\alpha]_D^{25}$ = 36 (c 0.1, $CHCl_3$);

^1H NMR (500 MHz, $CDCl_3$): δ = 7.60 – 7.47 (m, 2H), 6.68 – 6.60 (m, 2H), 3.98 (s, 3H), 3.86 (s, 3H), 3.69 (s, 3H), 3.51 (s, 3H), 1.80 – 1.59 (m, 8H), 1.58 – 1.41 (m, 4H), 1.39 – 1.09 (m, 8H), 1.06 – 0.88 (m, 2H) ppm.

^{13}C NMR (126 MHz, $CDCl_3$): δ = 157.54, 155.47, 149.99, 147.76 (d, J = 12.0 Hz), 132.87, 129.36, 126.26, 123.40 (d, J = 89.3 Hz), 116.04 (d, J = 8.8 Hz), 115.24 (d, J = 12.0 Hz), 108.38, 107.58, 60.64, 56.14, 55.98, 55.77, 37.94 (d, J = 37.6 Hz), 37.41 (d, J = 37.2 Hz), 26.92 – 26.17 (m), 26.12 – 25.25 (m), 21.21 (d, J = 37.2 Hz) ppm.

^{31}P NMR (202 MHz, $CDCl_3$): δ = 49.21 ppm.

HRMS (ESI$^+$): calcd $[M+Na]^+$ for $[C_{28}H_{38}BrO_5PNa]^+$: 587.15324, found 587.15315.

HPLC condition: Daicel Chiralpak IC, i-PrOH/n-hexane = 20/80, 0.8 mL/min, 254 nm UV detector; t_1= 20.6 min (major), t_2 = 23.7 min (minor).

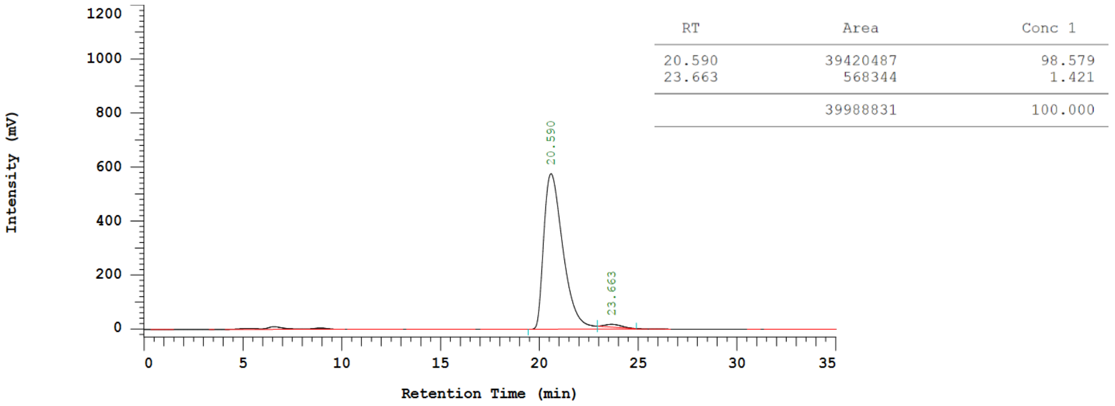

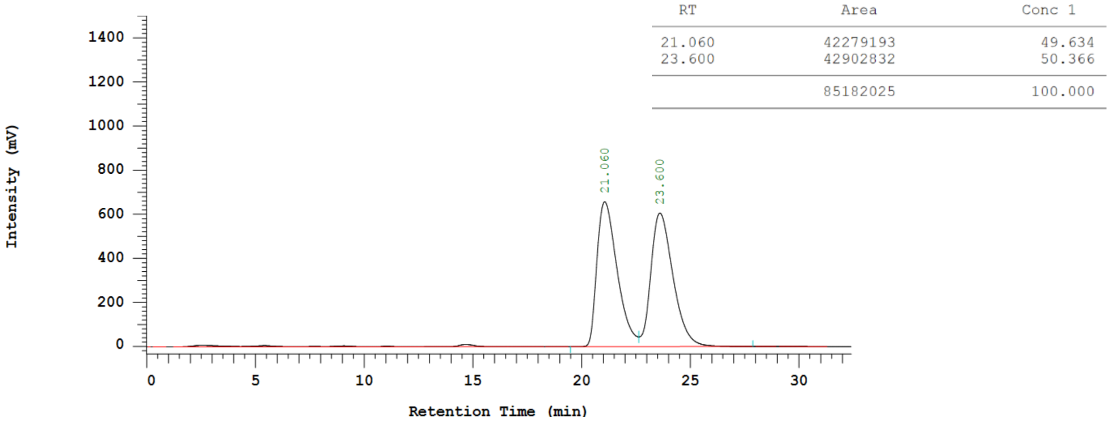

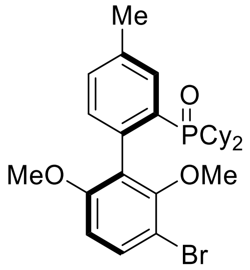

(*R*)-(3'-bromo-2',6'-dimethoxy-4-methyl-[1,1'-biphenyl]-2-yl)dicyclohexylphosphine oxide (18)

Appearance: colorless foam;

Yield: 94%; **e.e**: 96%; $[\alpha]_D^{25}$ = 23 (*c* 0.1, $CHCl_3$);

^{1}H NMR (500 MHz, $CDCl_3$): δ = 7.75 (d, *J* = 11.3 Hz, 1H), 7.51 (d, *J* = 8.9 Hz, 1H), 7.29 (d, *J* = 7.8 Hz, 1H), 7.09 – 7.04 (m, 1H), 6.61 (d, *J* = 8.9 Hz, 1H), 3.67 (s, 3H), 3.47 (s, 3H), 2.44 (s, 3H), 1.85 – 1.60 (m, 88H), 1.58 – 1.41 (m, 6H), 1.33 – 1.11 (m, 6H), 1.08 – 0.89 (m, 2H) ppm.

^{13}C NMR (126 MHz, $CDCl_3$): δ = 157.46, 155.32, 136.86 (d, *J* = 10.0 Hz), 133.85 (d, *J* = 7.5 Hz), 133.71 (d, *J* = 7.1 Hz), 132.62, 132.23 (d, *J* = 9.9 Hz), 131.52 (d, *J* = 81.7 Hz), 131.02 (d, *J* = 2.5 Hz), 126.55 (d, *J* = 2.0 Hz), 108.27, 107.52, 60.51, 55.75, 37.78 (d, *J* = 46.3 Hz), 37.26 (d, *J* = 46.5 Hz), 26.95 – 26.25 (m), 26.08 – 25.55 (m), 21.41 ppm.

^{31}P NMR (202 MHz, $CDCl_3$): δ = 47.88 ppm.

HRMS (ESI$^+$): calcd $[M+Na]^+$ for $[C_{27}H_{36}BrO_3PNa]^+$: 541.14777, found 541.14790.

HPLC condition: Daicel Chiralpak IA, i-PrOH/n-hexane = 10/90, 0.8 mL/min, 254 nm UV detector; t_1= 15.2 min (minor), t_2 = 18.4 min (major).

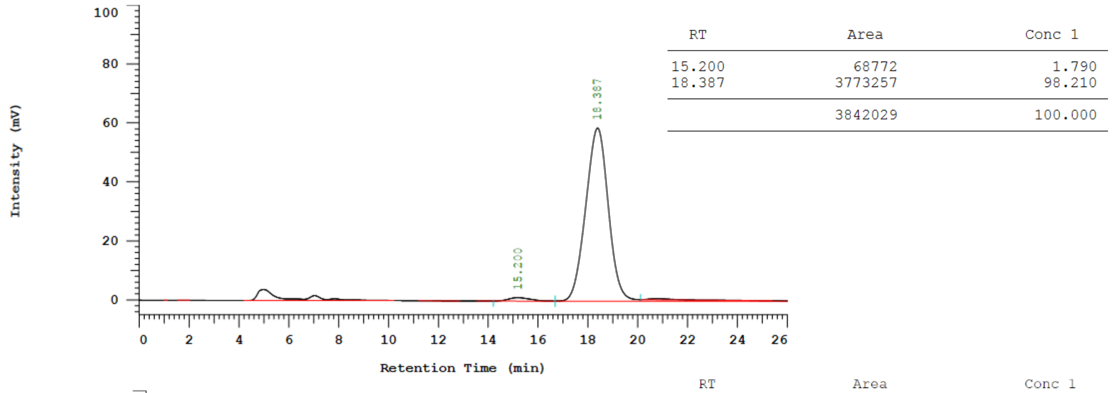

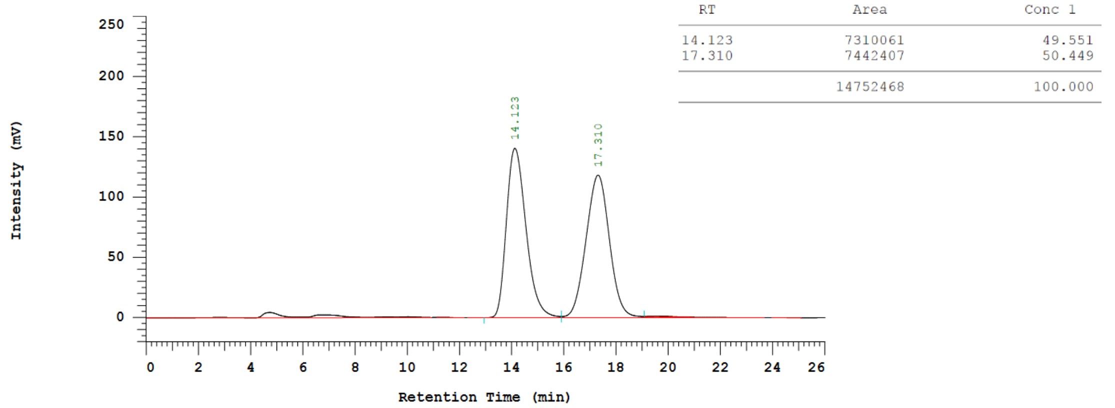

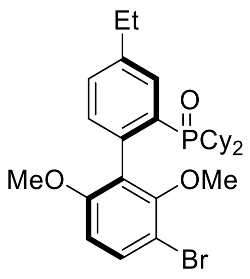

(R)-(3'-bromo-4-ethyl-2',6'-dimethoxy-[1,1'-biphenyl]-2-yl)dicyclohexylphosphine oxide (19)

Appearance: colorless foam;

Yield: 89%; **e.e**: 97%; $[\alpha]_D^{25}$ = 21 (c 0.1, $CHCl_3$);

^{1}H NMR (500 MHz, $CDCl_3$): δ = 7.76 (dd, J = 11.3, 1.9 Hz, 1H), 7.51 (d, J = 8.9 Hz, 1H), 7.35 – 7.29 (m, 1H), 7.12 – 7.07 (m, 1H), 6.61 (d, J = 8.9 Hz, 1H), 3.68 (s, 3H), 3.48 (s, 3H), 2.75 (q, J = 7.6 Hz, 2H), 1.85 – 1.61 (m, 8H), 1.59 – 1.43 (m, 6H), 1.30 (t, J = 7.6 Hz, 3H), 1.28 – 1.08 (m, 6H), 1.07 – 0.93 (m, 2H) ppm.

^{13}C NMR (126 MHz, $CDCl_3$): δ = 157.49, 155.32, 142.90 (d, J = 9.8 Hz), 133.89 (d, J = 7.0 Hz), 132.83 (d, J = 7.5 Hz), 132.59, 132.25 (d, J = 9.9 Hz), 131.67 (d, J = 81.5 Hz), 129.57 (d, J = 2.7 Hz), 126.65 (d, J = 2.2 Hz), 108.27, 107.49, 60.54, 55.75, 37.81 (d, J = 45.4 Hz), 37.29 (d, J = 45.4 Hz), 28.57, 27.11 – 26.24 (m), 26.16 – 25.48 (m), 15.23 ppm.

^{31}P NMR (202 MHz, $CDCl_3$): δ = 47.42 ppm.

HRMS (ESI$^+$): calcd $[M+Na]^+$ for $[C_{28}H_{38}BrO_3PNa]^+$: 555.16342, found 555.16318.

HPLC condition: Daicel Chiralpak IA, i-PrOH/n-hexane = 10/90, 0.8 mL/min, 254 nm UV detector; t_1= 12.0 min (minor), t_2 = 14.0 min (major).

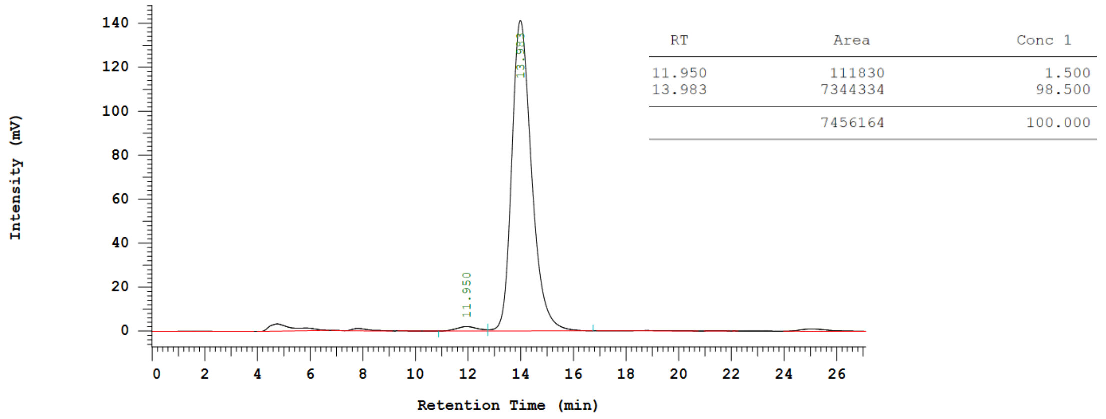

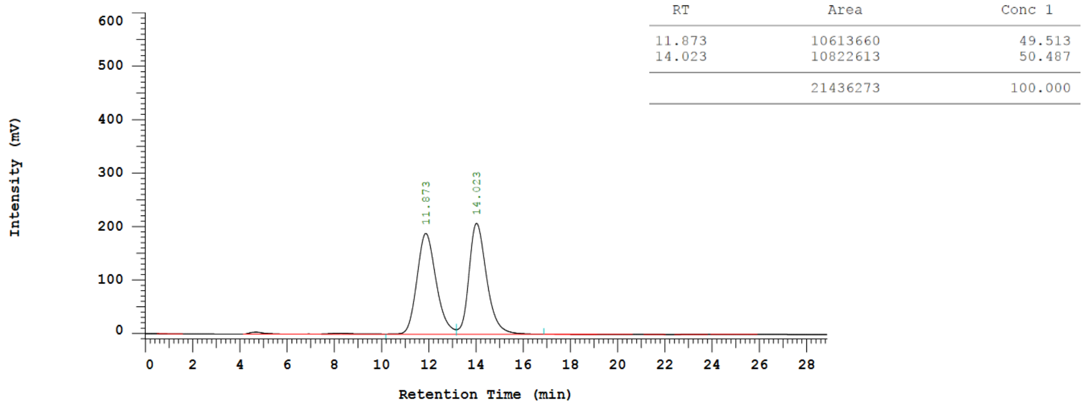

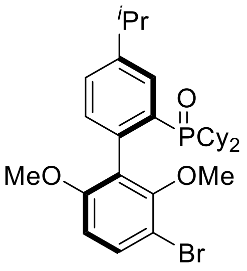

(*R*)-(3'-bromo-4-isopropyl-2',6'-dimethoxy-[1,1'-biphenyl]-2-yl)dicyclohexylphosphine oxide (20)

Appearance: colorless foam;

Yield: 92%; **e.e**: 92%; **$[\alpha]_D^{25}$** = 23 (*c* 0.1, $CHCl_3$);

^{1}H NMR (500 MHz, $CDCl_3$): δ = 7.75 (dd, *J* = 11.5, 2.0 Hz, 1H), 7.50 (d, *J* = 8.9 Hz, 1H), 7.37 – 7.31 (m, 1H), 7.12 – 7.06 (m, 1H), 6.61 (d, *J* = 8.9 Hz, 1H), 3.67 (s, 3H), 3.47 (s, 3H), 3.01 (hept, *J* = 6.9 Hz, 1H), 1.83 – 1.60 (m, 8H), 1.59 – 1.43 (m, 6H), 1.30 (dd, *J* = 7.0, 1.7 Hz, 6H), 1.28 – 1.09 (m, 6H), 1.09 – 0.91 (m, 2H) ppm.

^{13}C NMR (126 MHz, $CDCl_3$): δ = 157.48, 155.32, 147.40 (d, *J* = 9.5 Hz), 134.07 (d, *J* = 7.0 Hz), 132.55, 132.25 (d, *J* = 9.9 Hz), 131.58 (d, *J* = 7.7 Hz), 131.43 (d, *J* = 82.0 Hz), 127.99 (d, *J* = 2.6 Hz), 126.66 (d, *J* = 2.2 Hz), 108.26, 107.48, 60.55, 55.73, 37.79 (d, *J* = 48.8 Hz), 37.26 (d, *J* = 49.1 Hz), 33.76, 26.90 – 26.50 (m), 26.36 (d, *J* = 3.3 Hz), 26.13 – 25.54 (m), 23.86 (d, *J* = 11.4 Hz) ppm.

^{31}P NMR (202 MHz, $CDCl_3$): δ = 47.63 ppm.

HRMS (ESI$^+$): calcd $[M+Na]^+$ for $[C_{29}H_{40}BrO_3PNa]^+$: 569.17907, found 569.17887.

HPLC condition: Daicel Chiralpak IA, i-PrOH/n-hexane = 5/95, 0.8 mL/min, 254 nm UV detector; t_1= 21.4 min (minor), t_2 = 26.5 min (major).

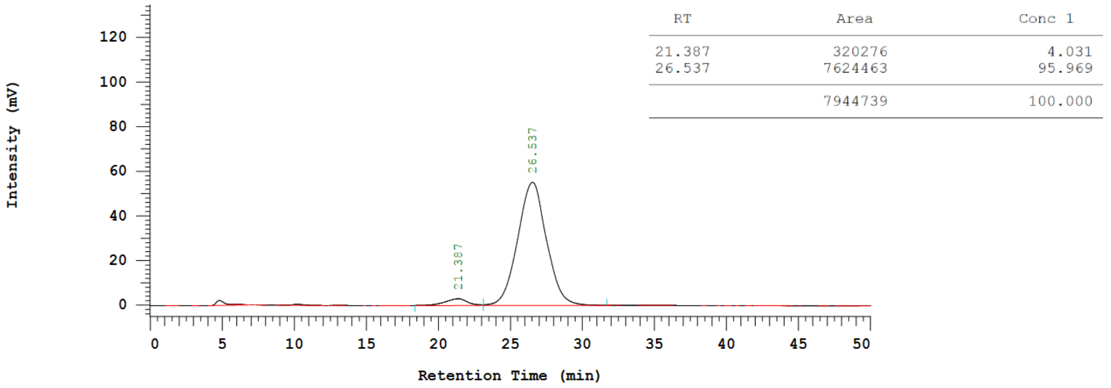

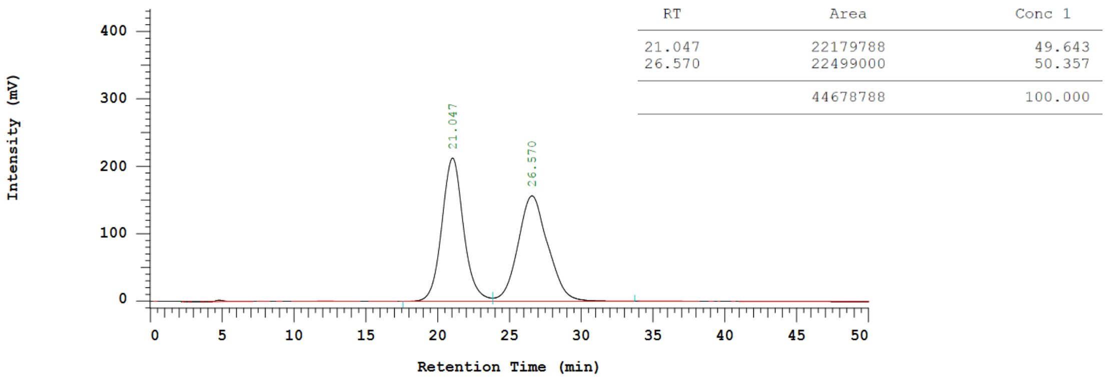

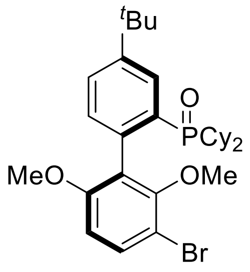

(*R*)-(3'-bromo-4-(*tert*-butyl)-2',6'-dimethoxy-[1,1'-biphenyl]-2-yl)dicyclohexylphosphine oxide (21)

Appearance: colorless foam;

Yield: 94%; **e.e**: 97%; $[\alpha]_D^{25}$ = 16 (c 0.1, $CHCl_3$);

^{1}H NMR (500 MHz, $CDCl_3$): δ = 7.83 (d, *J* = 11.9 Hz, 1H), 7.52 – 7.46 (m, 2H), 7.10 (dd, *J* = 8.0, 3.9 Hz, 1H), 6.60 (d, *J* = 8.9 Hz, 1H), 3.67 (s, 3H), 3.47 (s, 3H), 1.85 – 1.42 (m, 14H), 1.37 (s, 9H), 1.32 – 0.93 (m, 8H) ppm.

^{13}C NMR (126 MHz, $CDCl_3$): δ = 157.43, 155.29, 149.64 (d, *J* = 9.5 Hz), 134.11 (d, *J* = 6.7 Hz), 132.46, 132.06 (d, *J* = 10.0 Hz), 130.78 (d, *J* = 82.7 Hz), 129.99 (d, *J* = 8.5 Hz), 126.99 (d, *J* = 2.6 Hz), 126.58 (d, *J* = 2.1 Hz), 108.21, 107.45, 60.57, 55.70, 37.76 (d, *J* = 62.3 Hz), 37.24 (d, *J* = 62.6 Hz), 34.72, 31.28, 26.90 – 26.47 (m), 26.30 – 25.64 (m) ppm.

^{31}P NMR (202 MHz, $CDCl_3$): δ = 47.94 ppm.

HRMS (ESI$^+$): calcd $[M+Na]^+$ for $[C_{30}H_{42}BrO_3PNa]^+$: 583.19472, found 583.19467.

HPLC condition: Daicel Chiralpak IA, i-PrOH/n-hexane = 5/95, 0.8 mL/min, 254 nm UV detector; t_1= 21.2 min (minor), t_2 = 26.2 min (major).

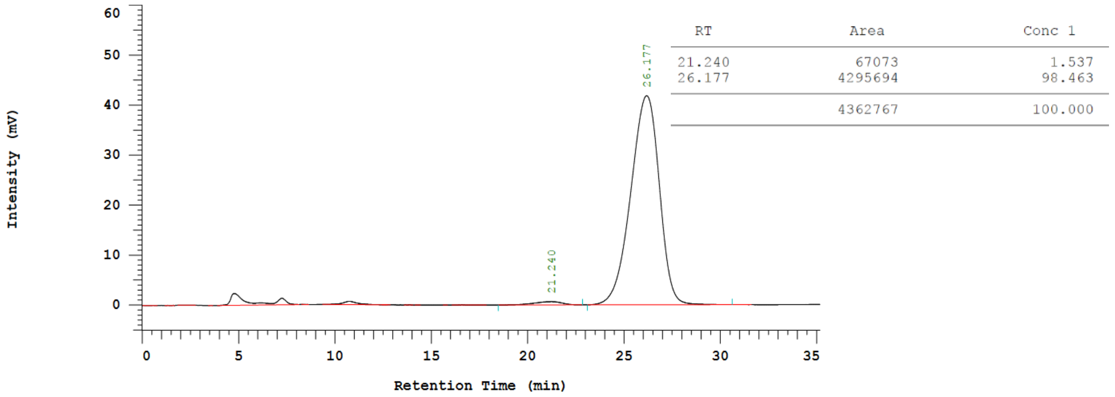

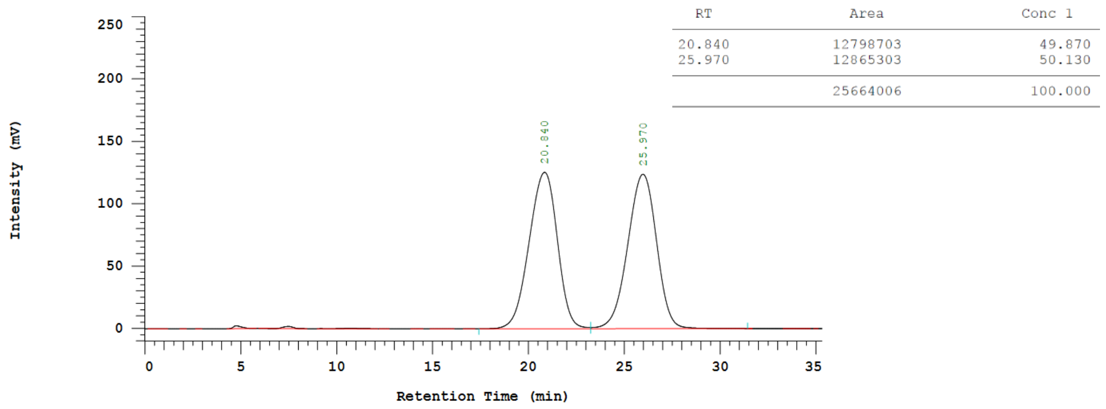

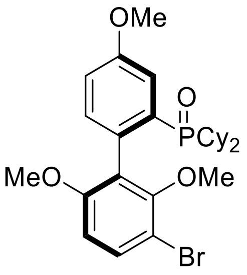

(*R*)-(3'-bromo-2',4,6'-trimethoxy-[1,1'-biphenyl]-2-yl)dicyclohexylphosphine oxide (22)

Appearance: colorless foam;

Yield: 88%; **e.e**: 96%; $[\alpha]_D^{25}$ = 22 (*c* 0.1, $CHCl_3$);

^{1}H NMR (500 MHz, $CDCl_3$): δ = 7.55 – 7.49 (m, 2H), 7.13 – 7.07 (m, 1H), 7.05 – 7.00 (m, 1H), 6.61 (d, *J* = 8.9 Hz, 1H), 3.89 (s, 3H), 3.68 (s, 3H), 3.48 (s, 3H), 1.83 – 1.62 (m, 8H), 1.59 – 1.42 (m, 6H), 1.35 – 1.09 (m, 6H), 1.07 – 0.93 (m, 2H) ppm.

^{13}C NMR (126 MHz, $CDCl_3$): δ = 158.37 (d, *J* = 12.2 Hz), 157.68, 155.53, 133.63 (d, *J* = 79.9 Hz), 133.49 (d, *J* = 10.9 Hz), 132.63, 128.45 (d, *J* = 6.9 Hz), 126.30 (d, *J* = 2.1 Hz), 118.32 (d, *J* = 8.2 Hz), 116.01 (d, *J* = 2.5 Hz), 108.30, 107.48, 60.47, 55.76, 55.43, 37.78 (d, *J* = 45.8 Hz), 37.26 (d, *J* = 45.8 Hz), 26.88 – 26.34 (m), 26.09 – 25.54 (m) ppm.

^{31}P NMR (202 MHz, $CDCl_3$): δ = 47.45 ppm.

HRMS (ESI$^+$): calcd $[M+Na]^+$ for $[C_{27}H_{36}BrO_4PNa]^+$: 557.14268, found 557.14268.

HPLC condition: Daicel Chiralpak IF, i-PrOH/n-hexane = 10/90, 0.8 mL/min, 254 nm UV detector; t_1= 62.0 min (major), t_2 = 67.3 min (minor).

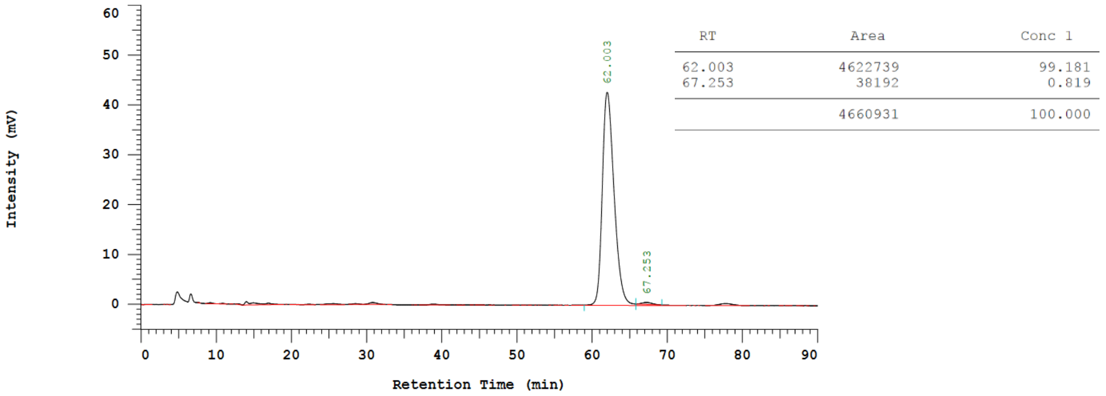

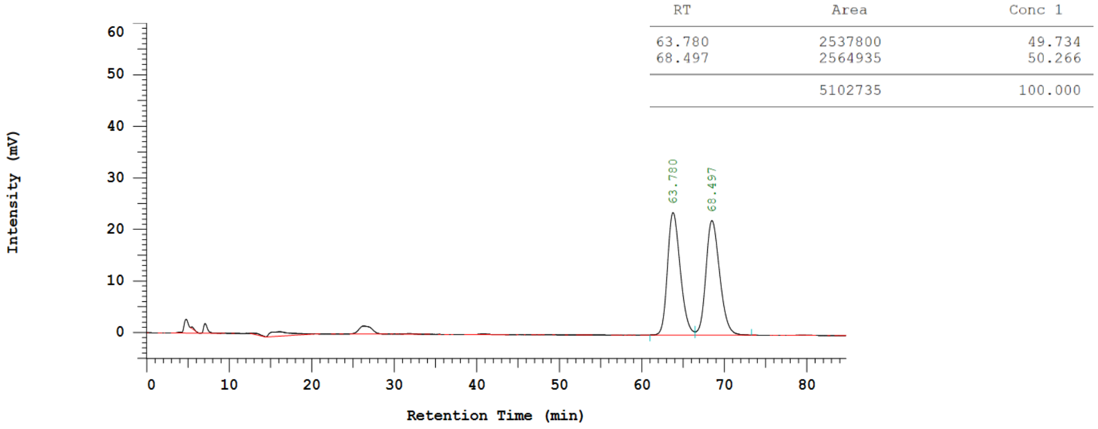

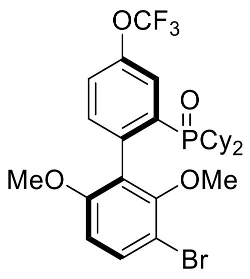

(*R*)-(3'-bromo-2',6'-dimethoxy-4-(trifluoromethoxy)-[1,1'-biphenyl]-2-yl)dicyclohexylphosphine oxide (23)

Appearance: colorless foam;

Yield: 81%; **e.e**: 99%; $[\alpha]_D^{25}$ = 19 (*c* 0.1, $CHCl_3$);

^{1}H NMR (500 MHz, $CDCl_3$): δ = 7.77 (dd, *J* = 11.2, 2.6 Hz, 1H), 7.54 (d, *J* = 8.9 Hz, 1H), 7.36 – 7.30 (m, 1H), 7.23 – 7.20 (m, 1H), 6.63 (d, *J* = 9.0 Hz, 1H), 3.69 (s, 3H), 3.50 (s, 3H), 1.83 – 1.62 (m, 8H), 1.60 – 1.41 (m, 6H), 1.35 – 1.12 (m, 6H), 1.11 – 0.95 (m, 2H) ppm.

^{13}C NMR (126 MHz, $CDCl_3$): δ = 157.26, 155.23, 148.21 (d, *J* = 12.1 Hz), 135.65 (d, *J* = 6.0 Hz), 135.20 (d, *J* = 77.7 Hz), 133.85 (d, *J* = 10.0 Hz), 133.19, 125.54 (d, *J* = 8.5 Hz), 125.24 (d, *J* = 1.9 Hz), 122.15, 120.50 (q, *J* = 257.8 Hz), 108.18, 107.57, 60.57, 55.74, 37.74 (d, *J* = 51.3 Hz), 37.44, 26.98 – 26.13 (m), 26.01 – 25.42 (m) ppm.

^{31}P NMR (202 MHz, $CDCl_3$): δ = 46.42 ppm.

^{19}F NMR (471 MHz, $CDCl_3$): δ = -57.66 ppm.

HRMS (ESI$^+$): calcd $[M+Na]^+$ for $[C_{27}H_{33}BrF_3O_4PNa]^+$: 611.11441, found 611.11435.

HPLC condition: Daicel Chiralpak ID, i-PrOH/n-hexane = 5/95, 0.8 mL/min, 254 nm UV detector; t_1= 45.2 min (major), t_2 = 49.0 min (minor).

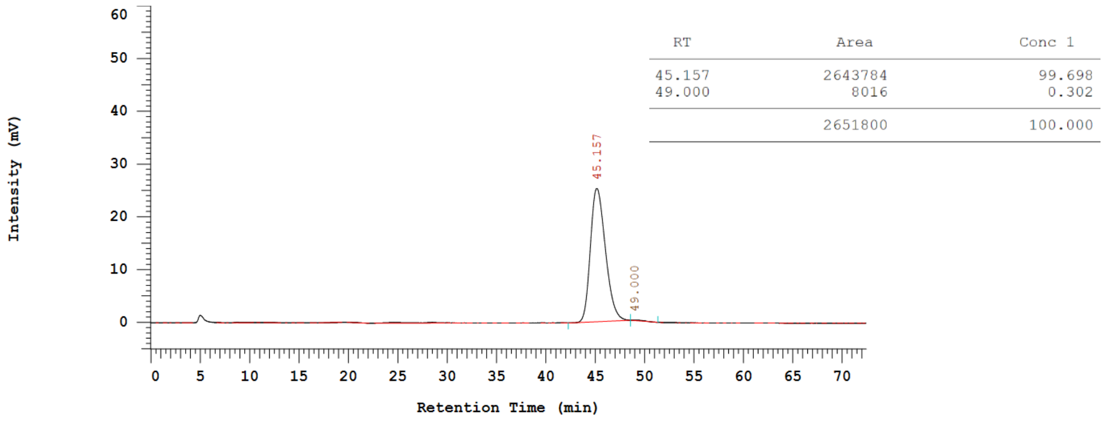

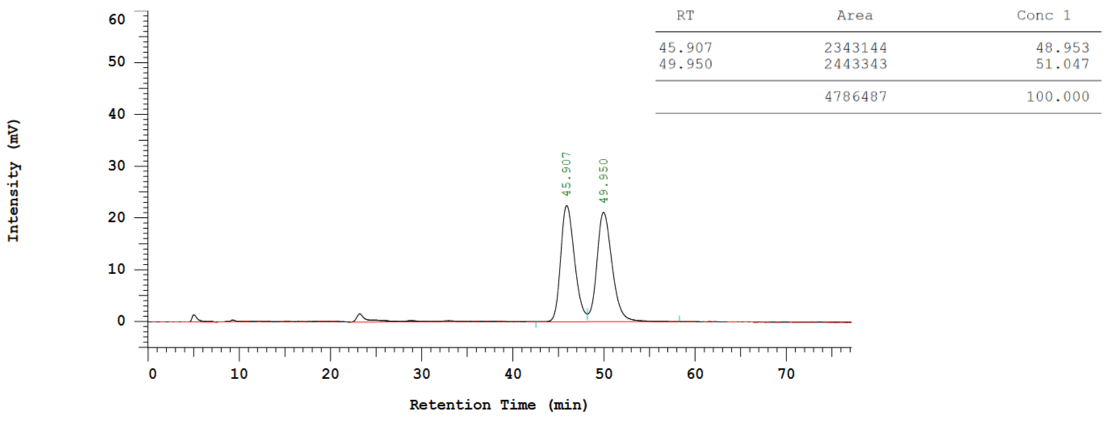

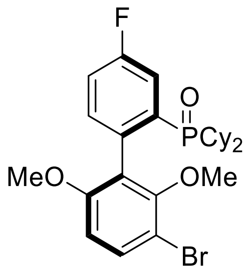

(*R*)-(3'-bromo-4-fluoro-2',6'-dimethoxy-[1,1'-biphenyl]-2-yl)dicyclohexylphosphine oxide (24)

Appearance: colorless foam;

Yield: 82%; **e.e**: 97%; $[\alpha]_D^{25}$ = 21 (*c* 0.1, $CHCl_3$);

^{1}H NMR (500 MHz, $CDCl_3$): δ = 7.73 – 7.65 (m, 1H), 7.54 (d, *J* = 8.9 Hz, 1H), 7.23 – 7.13 (m, 2H), 6.64 (d, *J* = 9.0 Hz, 1H), 3.70 (s, 3H), 3.50 (s, 3H), 1.86 – 1.62 (m, 8H), 1.61 – 1.42 (m, 6H), 1.39 – 1.11 (m, 6H), 1.09 – 0.98 (m, 2H) ppm.

^{13}C NMR (126 MHz, $CDCl_3$): δ = 162.69 (d, *J* = 13.6 Hz), 160.71 (d, *J* = 13.5 Hz), 157.41, 155.35, 134.08 (dd, *J* = 10.6, 7.0 Hz), 133.02, 132.64, 125.54, 120.20 (dd, *J* = 22.4, 8.1 Hz), 117.24 (dd, *J* = 21.2, 2.4 Hz), 108.22, 107.56, 60.49, 55.76, 37.72 (d, *J* = 49.9 Hz), 37.20 (d, *J* = 49.9 Hz), 26.81 – 26.22 (m), 25.90 – 25.61 (m) ppm.

^{19}F NMR (471 MHz, $CDCl_3$): δ = -113.95 (d, *J* = 3.2 Hz, 1F) ppm.

^{31}P NMR (202 MHz, $CDCl_3$): δ = 46.69 ppm.

HRMS (ESI$^+$): calcd $[M+Na]^+$ for $[C_{26}H_{33}BrFO_3PNa]^+$: 545.12269, found 545.12291.

HPLC condition: Daicel Chiralpak IA, i-PrOH/n-hexane = 5/95, 0.8 mL/min, 254 nm UV detector; t_1= 43.5 min (minor), t_2 = 50.3 min (major).

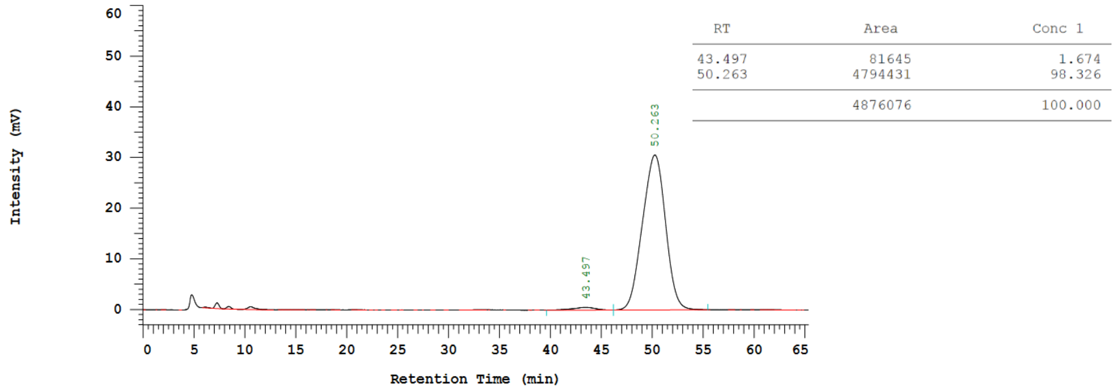

RT	Area	Conc 1
43.497	81645	1.674
50.263	4794431	98.326
	4876076	100.000

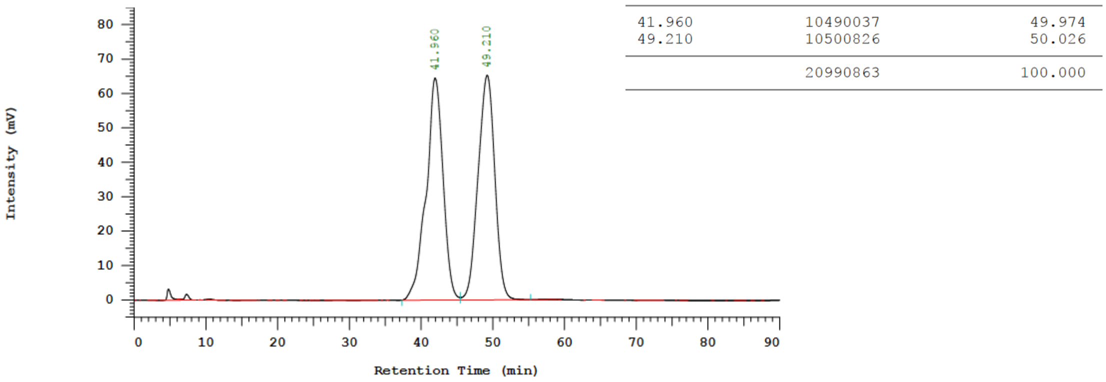

RT	Area	Conc 1
41.960	10490037	49.974
49.210	10500826	50.026
	20990863	100.000

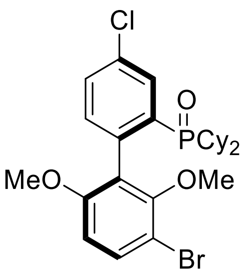

(*R*)-(3'-bromo-4-chloro-2',6'-dimethoxy-[1,1'-biphenyl]-2-yl)dicyclohexylphosphine oxide (25)

Appearance: colorless foam;

Yield: 81%; **e.e**: 91%; **[α]$_D^{25}$** = 27 (*c* 0.1, $CHCl_3$);

^{1}H NMR (500 MHz, $CDCl_3$): δ = 7.94 (dd, *J* = 11.0, 2.2 Hz, 1H), 7.54 (d, *J* = 8.8 Hz, 1H), 7.47 (dd, *J* = 8.1, 2.3 Hz, 1H), 7.13 (dd, *J* = 8.1, 4.0 Hz, 1H), 6.63 (d, *J* = 8.8 Hz, 1H), 3.68 (s, 3H), 3.49 (s, 3H), 1.89 – 1.61 (m, 8H), 1.60 – 1.42 (m, 6H), 1.38 – 1.08 (m, 6H), 1.00 (tdd, *J* = 20.5, 10.8, 5.6 Hz, 2H) ppm.

^{13}C NMR (126 MHz, $CDCl_3$): δ = 157.25, 155.19, 135.27 (d, *J* = 6.8 Hz), 133.90 (d, *J* = 12.9 Hz), 133.85 (d, *J* = 78.7 Hz), 133.78 (d, *J* = 10.1 Hz), 133.32, 133.20 (d, *J* = 8.1 Hz), 130.57 (d, *J* = 2.6 Hz), 125.20 (d, *J* = 2.0 Hz), 108.24, 107.71, 60.61, 55.87, 37.66 (d, *J* = 40.7 Hz), 37.14 (d, *J* = 40.9 Hz), 26.79 – 26.21 (m), 25.90 – 25.55 (m) ppm.

^{31}P NMR (202 MHz, $CDCl_3$): δ = 49.53 ppm.

HRMS (ESI$^+$): calcd [M+Na]$^+$ for [$C_{26}H_{33}BrClO_3PNa$]$^+$: 563.09117, found 563.09066.

HPLC condition: Daicel Chiralpak IF, i-PrOH/n-hexane = 10/90, 0.8 mL/min, 254 nm UV detector; t_1= 31.9 min (major), t_2 = 37.1 min (minor).

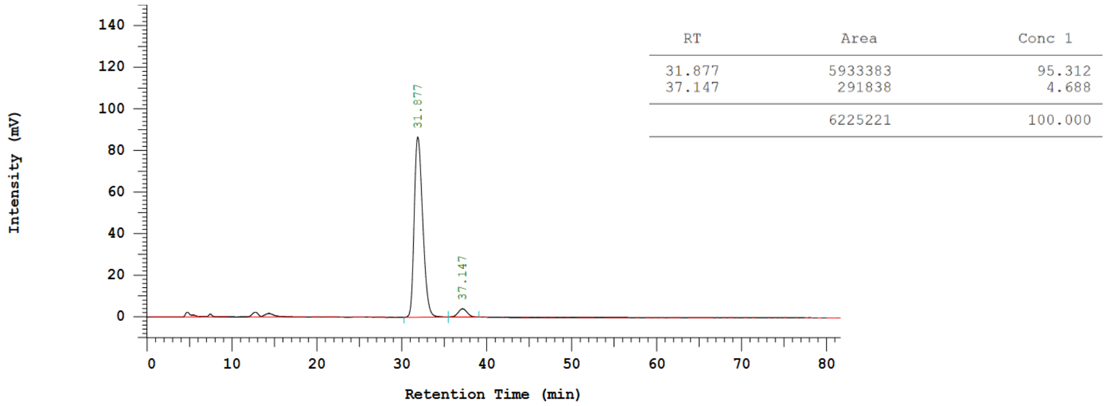

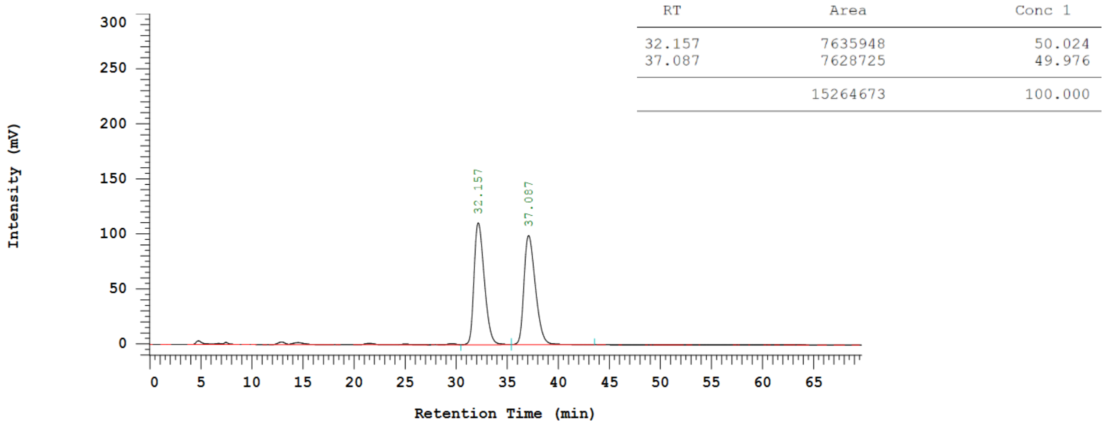

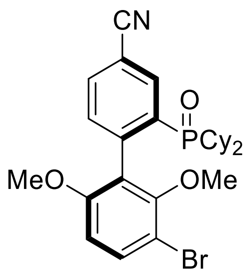

(*R*)-3'-bromo-2-(dicyclohexylphosphoryl)-2',6'-dimethoxy-[1,1'-biphenyl]-4-carbonitrile (26)

Appearance: colorless foam;

Yield: 84%; **e.e**: 90%; $[\alpha]_D^{25}$ = 31 (*c* 0.1, $CHCl_3$);

^{1}H NMR (500 MHz, $CDCl_3$): δ = 8.26 (dd, *J* = 10.4, 1.8 Hz, 1H), 7.82 – 7.70 (m, 1H), 7.56 (d, *J* = 8.9 Hz, 1H), 7.31 (dd, *J* = 7.9, 3.2 Hz, 1H), 6.64 (d, *J* = 8.9 Hz, 1H), 3.68 (s, 3H), 3.49 (s, 3H), 1.83 – 1.61 (m, 8H), 1.56 – 1.36 (m, 6H), 1.35 – 1.12 (m, 6H), 1.09 – 0.93 (m, 2H) ppm.

^{13}C NMR (126 MHz, $CDCl_3$): δ = 156.83, 154.85, 142.16 (d, *J* = 6.3 Hz), 137.00 (d, *J* = 8.3 Hz), 134.98 (d, *J* = 76.3 Hz), 133.75, 133.21 (d, *J* = 2.6 Hz), 133.14 (d, *J* = 8.8 Hz), 124.86 (d, *J* = 1.8 Hz), 118.37, 111.67 (d, *J* = 11.1 Hz), 108.16, 107.73, 60.67, 55.83, 37.76 (d, *J* = 37.7 Hz), 37.24 (d, *J* = 37.7 Hz), 26.81 – 26.14 (m), 25.95 – 25.45 (m) ppm.

^{31}P NMR (202 MHz, $CDCl_3$): δ = 46.97 ppm.

HRMS (ESI$^+$): calcd $[M+Na]^+$ for $[C_{27}H_{33}BrNO_3PNa]^+$: 552.12736, found 552.12710.

HPLC condition: Daicel Chiralpak IG, i-PrOH/n-hexane = 15/85, 0.8 mL/min, 254 nm UV detector; t_1= 67.8 min (minor), t_2 = 79.3 min (major).

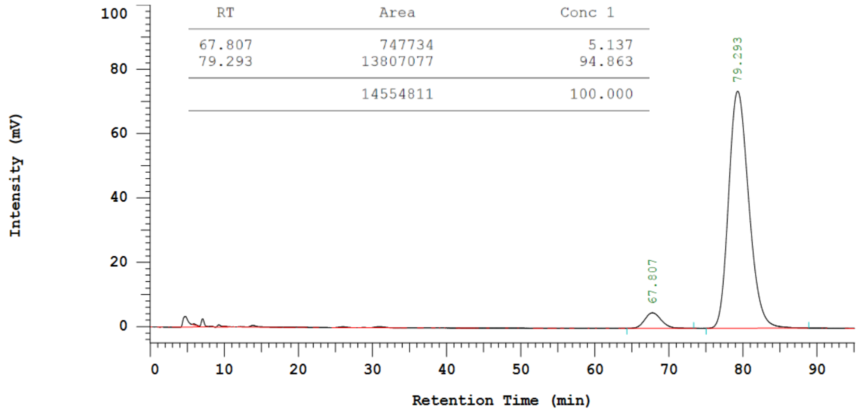

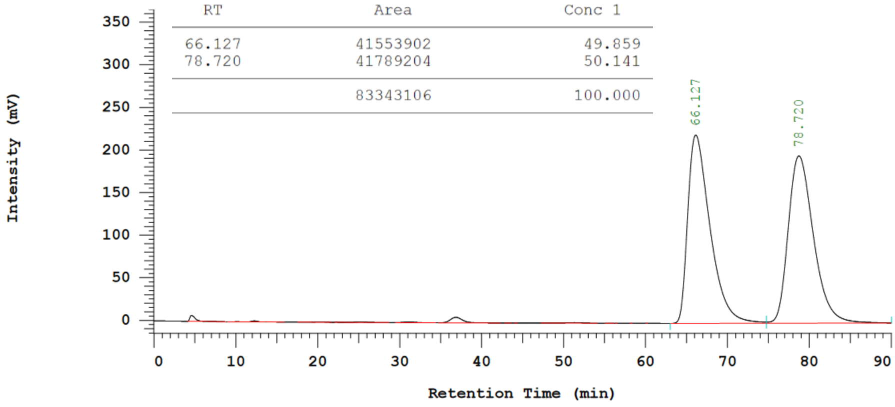

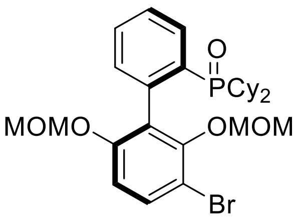

(*R*)-(3'-bromo-2',6'-bis(methoxymethoxy)-[1,1'-biphenyl]-2-yl)dicyclohexylphosphine oxide (27)

Appearance: colorless foam;

Yield: 86%; **e.e**: 97%; $[\alpha]_D^{25}$ = 26 (*c* 0.1, $CHCl_3$);

^{1}H NMR (500 MHz, $CDCl_3$): δ = 7.85 – 7.76 (m, 1H), 7.52 – 7.45 (m, 2H), 7.47 – 7.40 (m, 1H), 7.28 – 7.23 (m, 1H), 6.88 (d, *J* = 8.9 Hz, 1H), 5.18 (d, *J* = 6.7 Hz, 1H), 4.86 – 4.80 (m, 2H), 4.79 (d, *J* = 5.8 Hz, 1H), 3.31 (s, 3H), 2.95 (s, 3H), 1.88 – 1.54 (m, 12H), 1.51 – 1.39 (m, 2H), 1.37 – 1.27 (m, 2H), 1.27 – 0.99 (m, 6H) ppm.

^{13}C NMR (126 MHz, $CDCl_3$): δ = 155.00, 152.61, 137.85 (d, *J* = 5.9 Hz), 133.12 (d, *J* = 9.1 Hz), 132.83, 132.46 (d, *J* = 8.3 Hz), 131.92 (d, *J* = 81.3 Hz), 130.01 (d, *J* = 2.5 Hz), 127.95 (d, *J* = 2.3 Hz), 126.98 (d, *J* = 10.2 Hz), 111.77, 109.35, 99.10, 94.74, 57.16, 56.11, 37.70 (d, *J* = 12.8 Hz), 37.17 (d, *J* = 12.7 Hz), 26.71 (dd, *J* = 12.7, 5.8 Hz), 26.50 (d, *J* = 12.7 Hz), 26.24 (d, *J* = 3.4 Hz), 26.08 – 25.66 (m) ppm.

^{31}P NMR (202 MHz, $CDCl_3$): δ = 46.81 ppm.

HRMS (ESI$^+$): calcd $[M+Na]^+$ for $[C_{28}H_{38}BrO_5PNa]^+$: 587.15324, found 587.15305.

HPLC condition: Daicel Chiralpak IA, i-PrOH/n-hexane = 5/95, 0.8 mL/min, 254 nm UV detector; t_1= 77.9 min (minor), t_2 = 87.5 min (major).

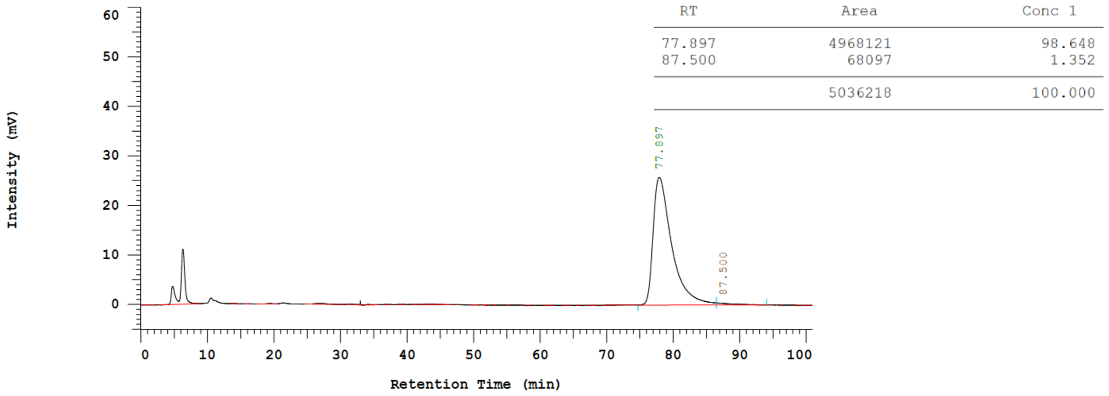

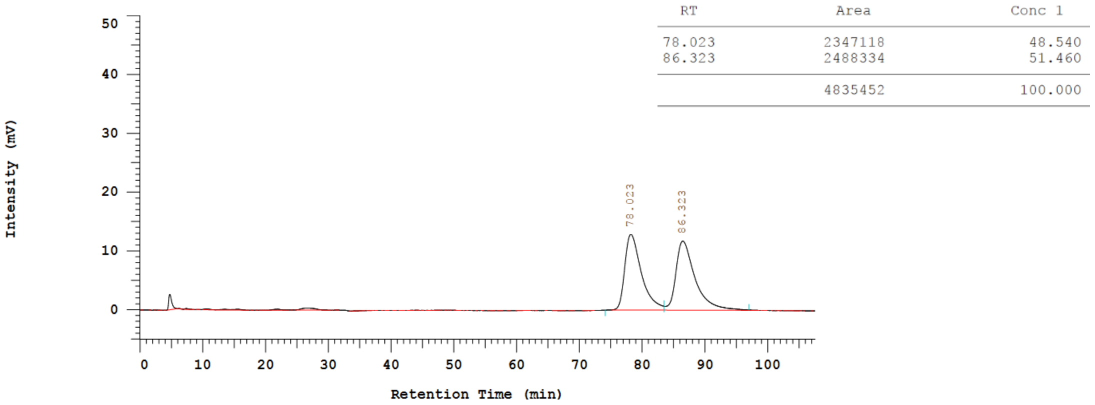

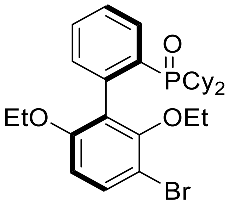

(*R*)-(3'-bromo-2',6'-diethoxy-[1,1'-biphenyl]-2-yl)dicyclohexylphosphine oxide (28)

Appearance: colorless foam;

Yield: 90%; **e.e**: 98%; $[\alpha]_D^{25}$ = 13 (*c* 0.1, $CHCl_3$);

^{1}H NMR (400 MHz, $CDCl_3$): δ = 8.05 – 7.87 (m, 1H), 7.54 – 7.40 (m, 3H), 7.21 – 7.12 (m, 1H), 6.60 (d, *J* = 8.8 Hz, 1H), 4.02 – 3.81 (m, 3H), 3.52 – 3.40 (m, 1H), 1.87 – 1.60 (m, 8H), 1.59 – 1.40 (m, 6H), 1.37 – 1.17 (m, 6H), 1.14 (t, *J* = 7.0 Hz, 3H), 1.10 – 0.99 (m, 2H), 0.96 (t, *J* = 7.0 Hz, 3H) ppm.

^{13}C NMR (101 MHz, $CDCl_3$): δ = 156.83, 154.84, 137.96, 137.21 (d, *J* = 8.0 Hz), 133.36 (d, *J* = 8.1 Hz), 132.58, 132.19 (d, *J* = 9.3 Hz), 129.83, 128.62 (d, *J* = 81.7 Hz), 126.89 (d, *J* = 9.9 Hz), 108.51, 108.37, 69.25, 64.16, 37.75 (d, *J* = 18.6 Hz), 37.09 (d, *J* = 18.4 Hz), 26.70 (d, *J* = 12.9 Hz), 26.60 – 26.20 (m), 26.07 – 25.65 (m), 15.16, 14.50 ppm.

^{31}P NMR (162 MHz, $CDCl_3$): δ = 48.28 ppm.

HRMS (ESI$^+$): calcd $[M+Na]^+$ for $[C_{28}H_{38}BrO_3PNa]^+$: 555.16342, found 555.16348.

HPLC condition: Daicel Chiralpak IC, i-PrOH/n-hexane = 5/95, 0.8 mL/min, 254 nm UV detector; t_1= 59.2 min (major), t_2 = 65.8 min (minor).

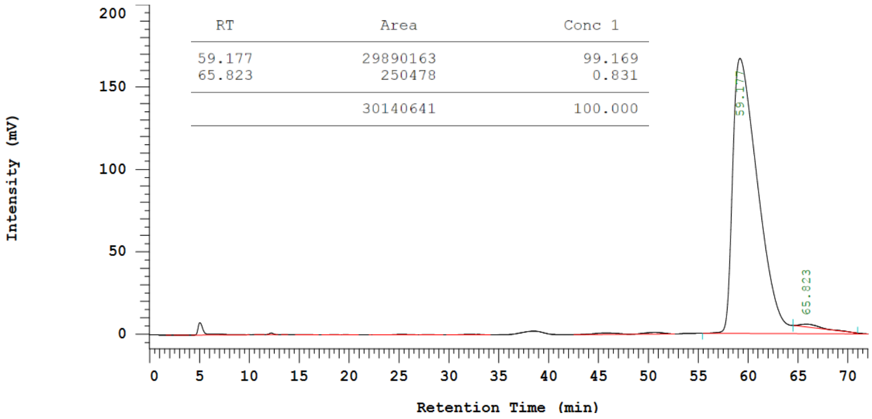

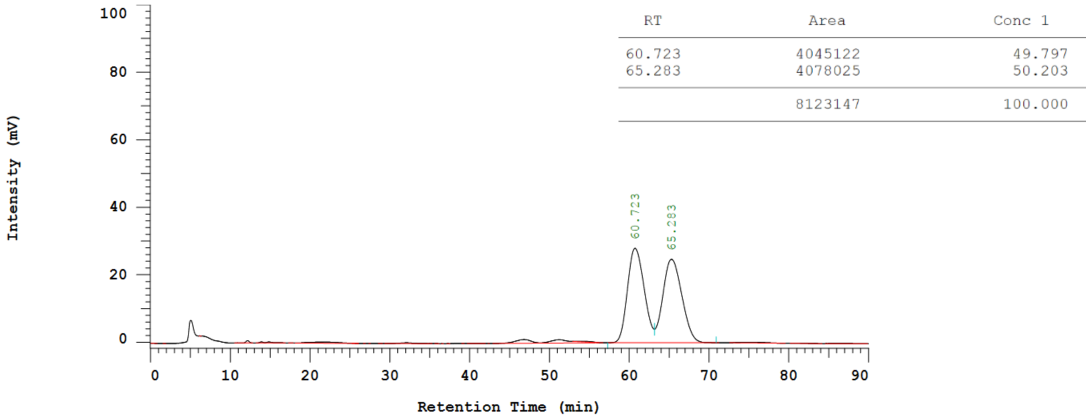

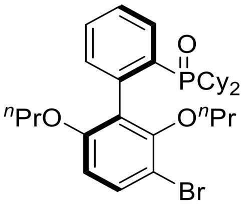

(*R*)-(3'-bromo-2',6'-dipropoxy-[1,1'-biphenyl]-2-yl)dicyclohexylphosphine oxide (29)

Appearance: colorless foam;

Yield: 91%; **e.e**: 94%; $[\alpha]_D^{25}$ = 14 (*c* 0.1, $CHCl_3$);

^{1}H NMR (400 MHz, $CDCl_3$): δ = 7.99 – 7.92 (m, 1H), 7.52 – 7.40 (m, 3H), 7.19 – 7.16 (m, 1H), 6.59 (d, *J* = 8.9 Hz, 1H), 3.92 – 3.79 (m, 2H), 3.73 (q, *J* = 7.5 Hz, 1H), 3.36 (q, *J* = 7.2 Hz, 1H), 1.88 – 1.60 (m, 8H), 1.60 – 1.37 (m, 8H), 1.36 – 1.09 (m, 8H), 1.07 – 0.93 (m, 2H), 0.75 (t, *J* = 7.4 Hz, 3H), 0.66 (t, *J* = 7.4 Hz, 3H) ppm.

^{13}C NMR (101 MHz, $CDCl_3$): δ = 157.00, 154.72, 137.20 (d, *J* = 7.1 Hz), 133.36 (d, *J* = 7.3 Hz), 132.68, 132.24 (d, *J* = 9.3 Hz), 131.80 (d, *J* = 60.8 Hz), 129.80 (d, *J* = 2.5 Hz), 126.94 (d, *J* = 10.2 Hz), 126.82, 108.56, 108.23, 75.22, 70.10, 60.41, 37.63 (d, *J* = 26.8 Hz), 37.11 (d, *J* = 26.8 Hz), 26.86 – 26.41 (m), 26.33 – 25.71 (m), 22.64 (d, *J* = 81.8 Hz), 21.48, 14.21, 10.27 (d, *J* = 17.6 Hz) ppm.

^{31}P NMR (162 MHz, $CDCl_3$): δ = 48.10 ppm.

HRMS (ESI$^+$): calcd $[M+Na]^+$ for $[C_{30}H_{42}BrO_3PNa]^+$: 583.19472, found 583.19469.

HPLC condition: Daicel Chiralpak ID, i-PrOH/n-hexane = 5/95, 0.6 mL/min, 254 nm UV detector; t_1= 63.9 min (major), t_2 = 70.3 min (minor).

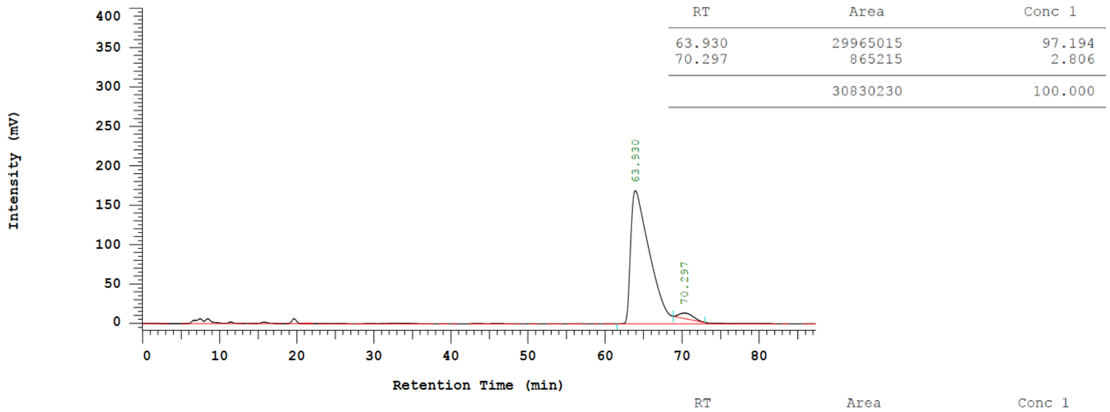

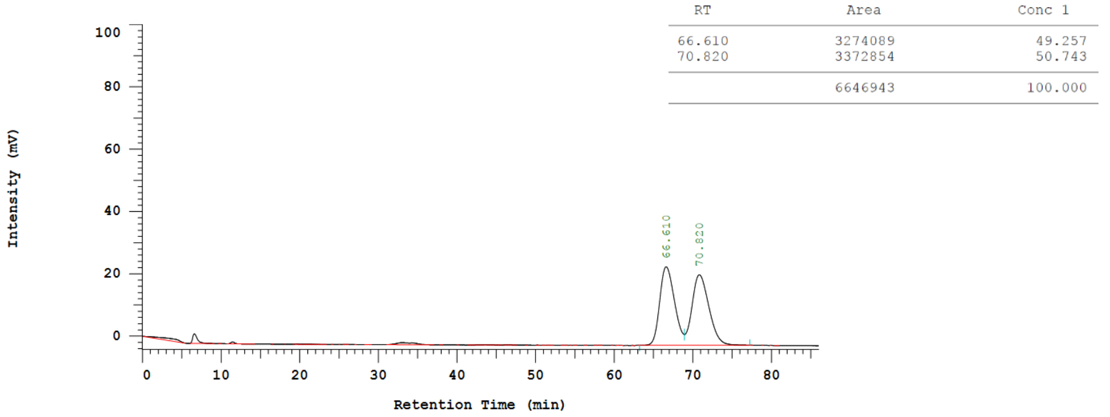

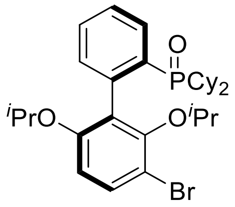

(*R*)-(3'-bromo-2',6'-diisopropoxy-[1,1'-biphenyl]-2-yl)dicyclohexylphosphine oxide (30)

Appearance: colorless foam;

Yield: 93%; **e.e**: 84%; $[\alpha]_D^{25}$ = 8 (*c* 0.1, $CHCl_3$);

^{1}H NMR (400 MHz, $CDCl_3$): δ = 7.93 – 7.84 (m, 1H), 7.50 – 7.37 (m, 3H), 7.21 – 7.13 (m, 1H), 6.60 (d, *J* = 8.8 Hz, 1H), 4.41 (hept, *J* = 6.1 Hz, 1H), 4.13 (hept, *J* = 6.2 Hz, 1H), 1.96 – 1.77 (m, 2H), 1.75 – 1.38 (m, 12H), 1.37 – 1.15 (m, 6H), 1.12 (d, *J* = 6.0 Hz, 6H), 1.08 (d, *J* = 6.2 Hz, 3H), 1.06 – 0.95 (m, 2H), 0.81 (d, *J* = 6.1 Hz, 3H) ppm.

^{13}C NMR (101 MHz, $CDCl_3$): δ = 155.73, 153.78, 137.55 (d, *J* = 82.2 Hz), 133.26 (d, *J* = 9.4 Hz), 133.00 (d, *J* = 7.8 Hz), 132.43, 131.68, 129.51 (d, *J* = 2.3 Hz), 128.10 (d, *J* = 2.2 Hz), 126.67 (d, *J* = 10.3 Hz), 110.32, 108.90, 76.19, 71.04, 37.49 (d, *J* = 20.3 Hz), 36.84 (d, *J* = 20.3 Hz), 26.81 – 26.49 (m), 26.28 (d, *J* = 3.3 Hz), 26.21 (d, *J* = 3.5 Hz), 26.02 (d, *J* = 3.0 Hz), 25.87 (d, *J* = 13.9 Hz), 25.69 (d, *J* = 3.3 Hz), 22.48, 22.43, 21.60 ppm.

^{31}P NMR (162 MHz, $CDCl_3$): δ = 48.48 ppm.

HRMS (ESI$^+$): calcd $[M+Na]^+$ for $[C_{30}H_{42}BrO_3PNa]^+$: 583.19472, found 583.19479.

HPLC condition: Daicel Chiralpak IA, i-PrOH/n-hexane = 10/90, 0.8 mL/min, 254 nm UV detector; t_1= 10.0 min (major), t_2 = 11.8 min (minor).

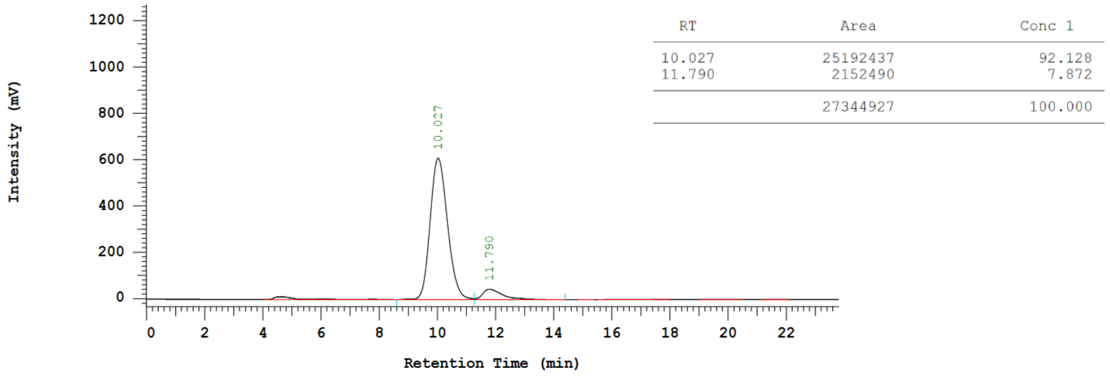

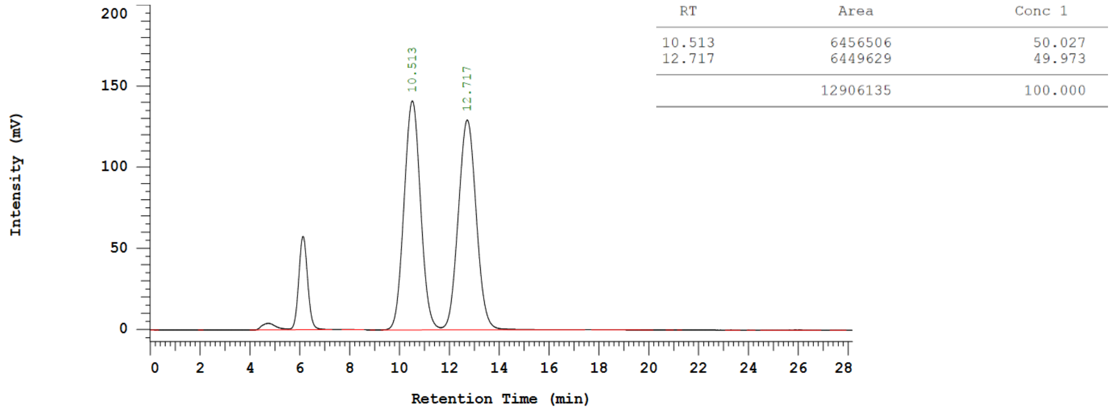

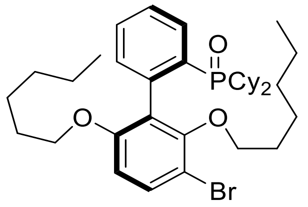

(*R*)-(3'-bromo-2',6'-bis(hexyloxy)-[1,1'-biphenyl]-2-yl)dicyclohexylphosphine oxide (31)

Appearance: colorless foam;

Yield: 92%; **e.e**: 91%; **[α]$_D^{25}$** = 18 (c 0.1, $CHCl_3$);

^{1}H NMR (500 MHz, $CDCl_3$): δ = 7.96 – 7.89 (m, 1H), 7.47 (d, J = 8.9 Hz, 1H), 7.42 (q, J = 7.8, 6.1 Hz, 2H), 7.18 – 7.12 (m, 1H), 6.58 (d, J = 8.9 Hz, 1H), 3.93 – 3.83 (m, 2H), 3.75 (q, J = 7.4 Hz, 1H), 3.41 – 3.33 (m, 1H), 1.84 – 1.40 (m, 16H), 1.39 – 1.20 (m, 5H), 1.20 – 0.96 (m, 11H), 0.83 – 0.76 (m, 6H) ppm.

^{13}C NMR (126 MHz, $CDCl_3$): δ = 157.04, 154.76, 137.34 (d, J = 7.1 Hz), 133.24 (d, J = 7.7 Hz), 132.63, 132.21 (d, J = 9.2 Hz), 132.05 (d, J = 81.3 Hz), 129.76 (d, J = 2.6 Hz), 126.96 – 126.88 (m), 126.85, 108.54, 108.21, 73.63, 68.60, 37.65 (d, J = 29.2 Hz), 37.12 (d, J = 29.2 Hz), 31.48, 31.28, 29.68, 28.82, 26.92 – 26.36 (m), 26.23 (d, J = 3.4 Hz), 26.10 – 25.60 (m), 25.32, 22.50, 22.44, 14.00, 13.91 ppm.

^{31}P NMR (202 MHz, $CDCl_3$): δ = 47.38 ppm.

HRMS (ESI$^+$): calcd $[M+Na]^+$ for $[C_{36}H_{54}BrO_3PNa]^+$: 667.28862, found 667.28822.

HPLC condition: Daicel Chiralpak ID, i-PrOH/n-hexane = 3/97, 0.8 mL/min, 254 nm UV detector; t_1= 91.8 min (major), t_2 = 103.0 min (minor).

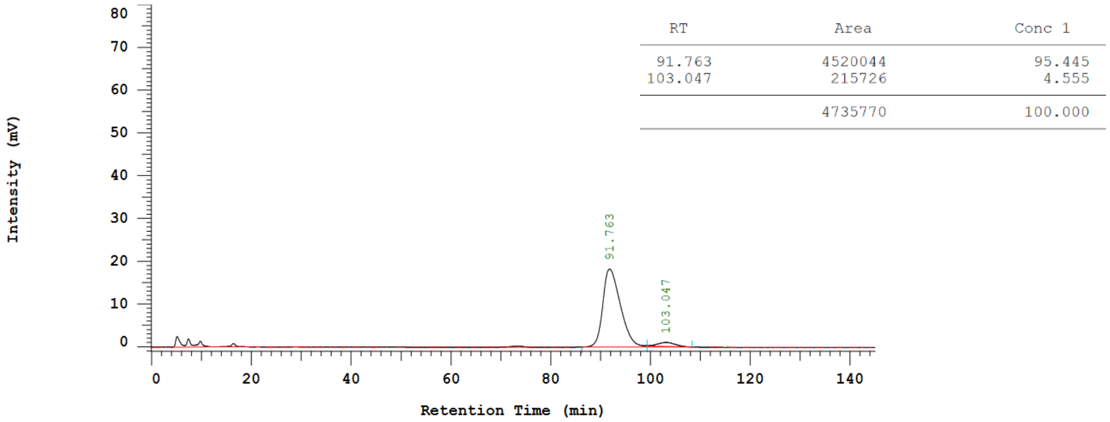

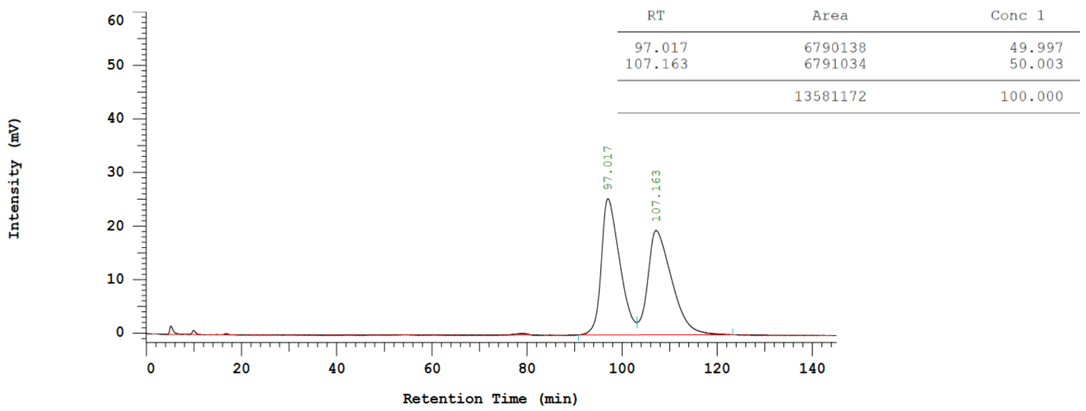

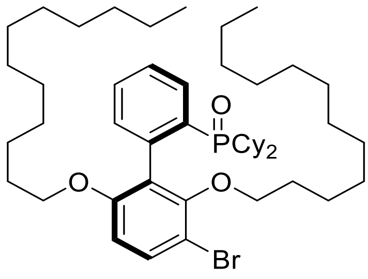

(*R*)-(3'-bromo-2',6'-bis(dodecyloxy)-[1,1'-biphenyl]-2-yl)dicyclohexylphosphine oxide (32)

Appearance: colorless foam;

Yield: 90%; **e.e**: 89%; **[*α*]$_D^{25}$** = 11 (*c* 0.1, $CHCl_3$);

^{1}H NMR (500 MHz, $CDCl_3$): δ = 8.00 – 7.89 (m, 1H), 7.48 (d, *J* = 8.8 Hz, 1H), 7.46 – 7.39 (m, 2H), 7.19 – 7.12 (m, 1H), 6.58 (d, *J* = 8.9 Hz, 1H), 3.93 – 3.83 (m, 2H), 3.81 – 3.71 (m, 1H), 3.43 – 3.33 (m, 1H), 1.85 – 1.67 (m, 5H), 1.67 – 1.41 (m, 11H), 1.39 – 1.09 (m, 40H), 1.09 – 0.94 (m, 6H), 0.87 (t, *J* = 6.9 Hz, 6H) ppm.

^{13}C NMR (126 MHz, $CDCl_3$): δ = 157.06, 154.76, 137.29, 133.29 (d, *J* = 7.4 Hz), 132.62, 132.23 (d, *J* = 81.2 Hz), 132.19 (d, *J* = 9.2 Hz), 129.71 (d, *J* = 2.7 Hz), 126.95 (d, *J* = 2.2 Hz), 126.87 (d, *J* = 10.3 Hz), 108.54, 108.23, 73.64, 68.60, 37.67 (d, *J* = 23.9 Hz), 37.15 (d, *J* = 23.9 Hz), 31.93, 29.73, 29.65, 29.64, 29.57, 29.49, 29.46, 29.36, 29.30, 29.13, 28.88, 26.90 – 26.43 (m), 26.34 – 25.72 (m), 25.66 (d, *J* = 1.6 Hz), 22.70, 14.14 ppm.

^{31}P NMR (202 MHz, $CDCl_3$): δ = 47.02 ppm.

HRMS (ESI$^+$): calcd $[M+Na]^+$ for $[C_{48}H_{78}BrO_3PNa]^+$: 835.47642, found 835.47588.

HPLC condition: Daicel Chiralpak ID, i-PrOH/n-hexane = 3/97, 0.8 mL/min, 254 nm UV detector; t_1= 41.1 min (major), t_2 = 45.4 min (minor).

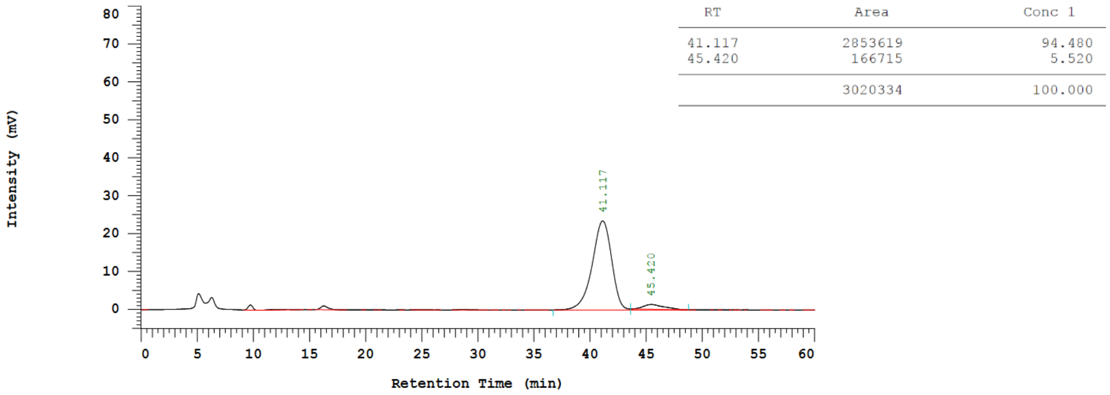

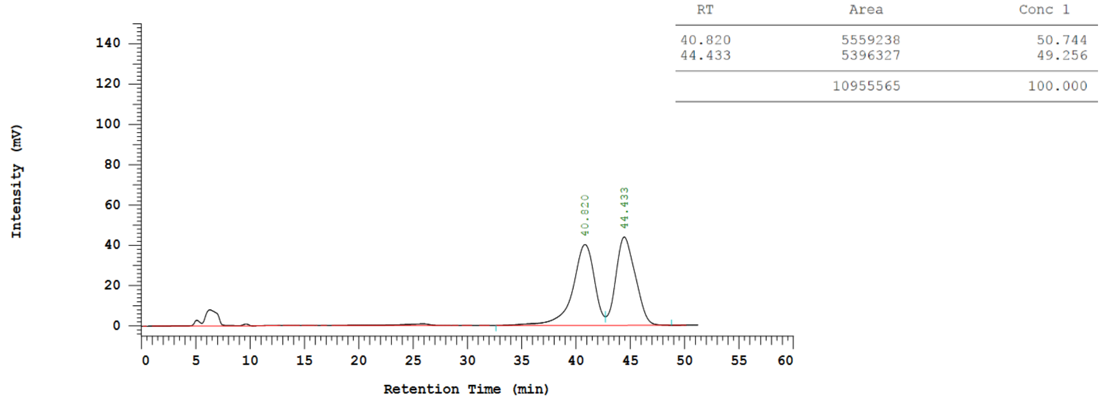

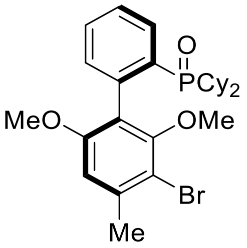

(*R*)-(3'-bromo-2',6'-dimethoxy-4'-methyl-[1,1'-biphenyl]-2-yl)dicyclohexylphosphine oxide (33)

Appearance: colorless foam;

Yield: 86%; **e.e**: 95%; **$[\alpha]_D^{25}$** = 34 (*c* 0.1, $CHCl_3$);

^{1}H NMR (500 MHz, $CDCl_3$): δ = 7.97 – 7.85 (m, 1H), 7.52 – 7.42 (m, 2H), 7.23 – 7.15 (m, 1H), 6.63 (s, 1H), 3.67 (s, 3H), 3.46 (s, 3H), 2.47 (s, 3H), 1.91 – 1.61 (m, 8H), 1.59 – 1.41 (m, 6H), 1.38 – 1.26 (m, 2H), 1.23 – 1.11 (m, 4H), 1.10 – 0.96 (m, 2H) ppm.

^{13}C NMR (126 MHz, $CDCl_3$): δ = 156.42, 155.10, 139.45, 137.28 (d, *J* = 6.9 Hz), 133.17 (d, *J* = 7.6 Hz), 132.68 (d, *J* = 9.3 Hz), 131.94 (d, *J* = 82.7 Hz), 130.13, 126.98 (d, *J* = 10.1 Hz), 123.73, 111.08, 108.54, 60.35, 55.64, 37.75 (d, *J* = 58.9 Hz), 37.23 (d, *J* = 59.3 Hz), 26.93 – 26.24 (m), 26.21 – 25.63 (m), 23.89 ppm.

^{31}P NMR (202 MHz, $CDCl_3$): δ = 48.38 ppm.

HRMS (ESI$^+$): calcd $[M+Na]^+$ for $[C_{27}H_{36}BrO_3PNa]^+$: 541.14777, found 541.14776.

HPLC condition: Daicel Chiralpak IC, i-PrOH/n-hexane = 10/90, 0.8 mL/min, 254 nm UV detector; t_1= 29.8 min (major), t_2 = 35.4 min (minor).

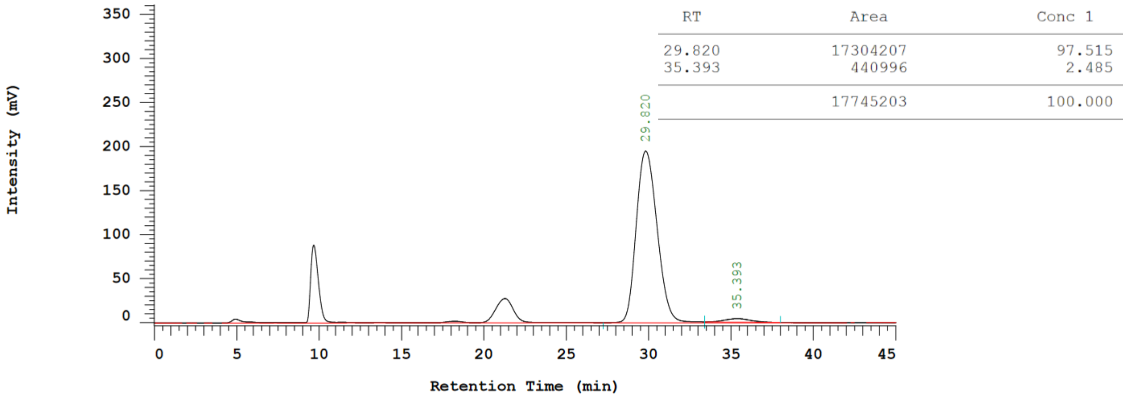

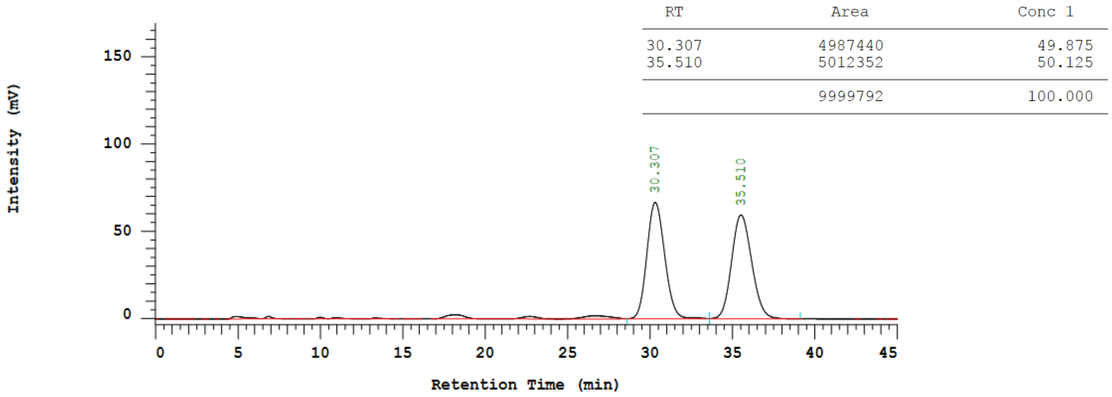

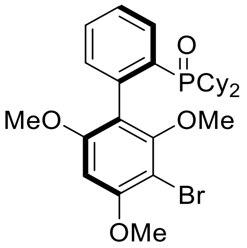

(*R*)-(3'-bromo-2',4',6'-trimethoxy-[1,1'-biphenyl]-2-yl)dicyclohexylphosphine oxide (34)

Appearance: colorless foam;

Yield: 96%; **e.e**: 91%; $[\alpha]_D^{25}$ = 48 (*c* 0.1, $CHCl_3$);

^{1}H NMR (400 MHz, $CDCl_3$): δ = 7.96 – 7.87 (m, 1H), 7.52 – 7.40 (m, 2H), 7.21 – 7.14 (m, 1H), 6.35 (s, 1H), 3.95 (s, 3H), 3.71 (s, 3H), 3.46 (s, 3H), 1.87 – 1.60 (m, 8H), 1.59 – 1.39 (m, 5H), 1.38 – 1.11 (m, 7H), 1.10 – 0.93 (m, 2H) ppm.

^{13}C NMR (101 MHz, $CDCl_3$): δ = 157.49, 157.15, 156.14, 137.10 (d, *J* = 6.9 Hz), 133.20 (d, *J* = 7.9 Hz), 133.01 (d, *J* = 9.3 Hz), 132.21 (d, *J* = 82.5 Hz), 130.12, 126.94 (d, *J* = 10.2 Hz), 118.66, 97.95, 91.86, 60.47, 56.48, 55.68, 37.85 (d, *J* = 34.6 Hz), 37.19 (d, *J* = 34.6 Hz), 26.92 – 26.25 (m), 26.00 – 25.64 (m), 21.23 (d, *J* = 35.4 Hz) ppm.

^{31}P NMR (162 MHz, $CDCl_3$): δ = 48.36 ppm.

HRMS (ESI$^+$): calcd $[M+Na]^+$ for $[C_{27}H_{36}BrO_4PNa]^+$: 557.14268, found 557.14250.

HPLC condition: Daicel Chiralpak IC, i-PrOH/n-hexane = 10/90, 0.8 mL/min, 254 nm UV detector; t_1= 50.9 min (minor), t_2 = 56.9 min (major).

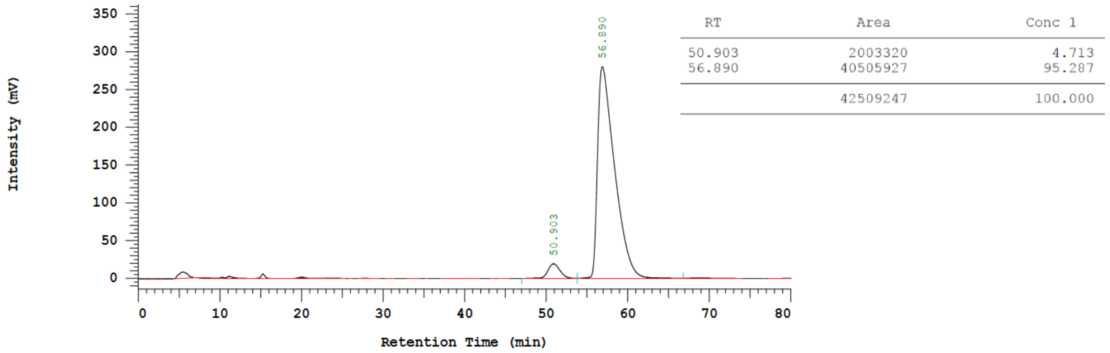

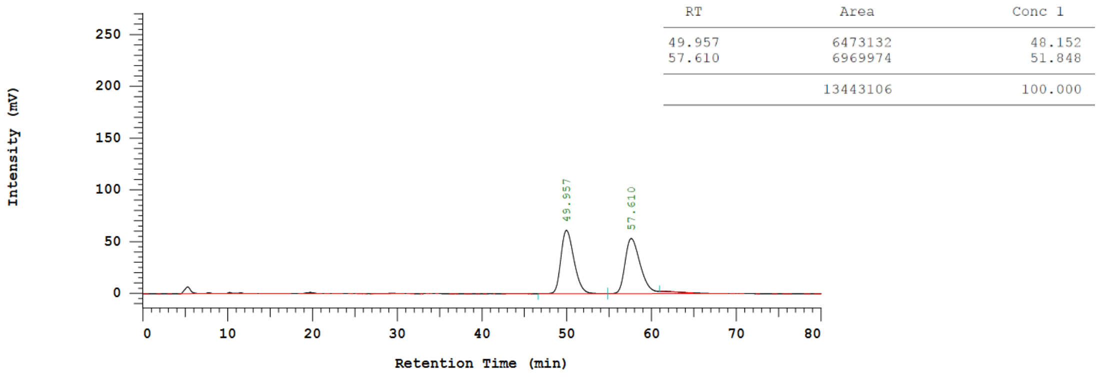

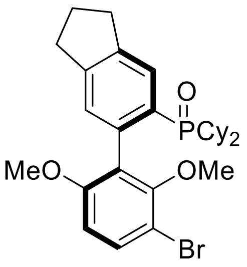

(*R*)-(6-(3-bromo-2,6-dimethoxyphenyl)-2,3-dihydro-1H-inden-5-yl)dicyclohexylphosphine oxide (35)

Appearance: colorless foam;

Yield: 88%; **e.e**: 95%; $[\alpha]_D^{25}$ = 33 (c 0.1, $CHCl_3$);

^{1}H NMR (500 MHz, $CDCl_3$): δ = 7.75 (d, J = 11.1 Hz, 1H), 7.50 (d, J = 8.8 Hz, 1H), 7.03 (d, J = 3.6 Hz, 1H), 6.61 (d, J = 8.9 Hz, 1H), 3.68 (s, 3H), 3.49 (s, 3H), 3.05 – 2.91 (m, 4H), 2.18 – 2.10 (m, 2H), 1.85 – 1.61 (m, 8H), 1.60 – 1.42 (m, 6H), 1.35 – 1.12 (m, 6H), 1.11 – 0.98 (m, 2H) ppm.

^{13}C NMR (126 MHz, $CDCl_3$): δ = 157.47, 155.27, 147.34, 143.55 (d, J = 11.0 Hz), 134.80 (d, J = 8.0 Hz), 132.62, 128.88 (d, J = 7.9 Hz), 128.46 (d, J = 10.8 Hz), 127.62 (d, J = 79.0 Hz), 126.77, 108.24, 107.54, 60.64, 55.78, 37.66 (d, J = 49.3 Hz), 37.15 (d, J = 49.6 Hz), 32.85, 32.68, 26.91 – 26.17 (m), 25.99 – 25.51 (m), 25.05 ppm.

^{31}P NMR (202 MHz, $CDCl_3$): δ = 51.84 ppm.

HRMS (ESI$^+$): calcd $[M+Na]^+$ for $[C_{29}H_{38}BrO_3PNa]^+$: 567.16342, found 567.16325.

HPLC condition: Daicel Chiralpak ID, i-PrOH/n-hexane = 10/90, 0.8 mL/min, 254 nm UV detector; t_1= 35.4 min (minor), t_2 = 39.4 min (major).

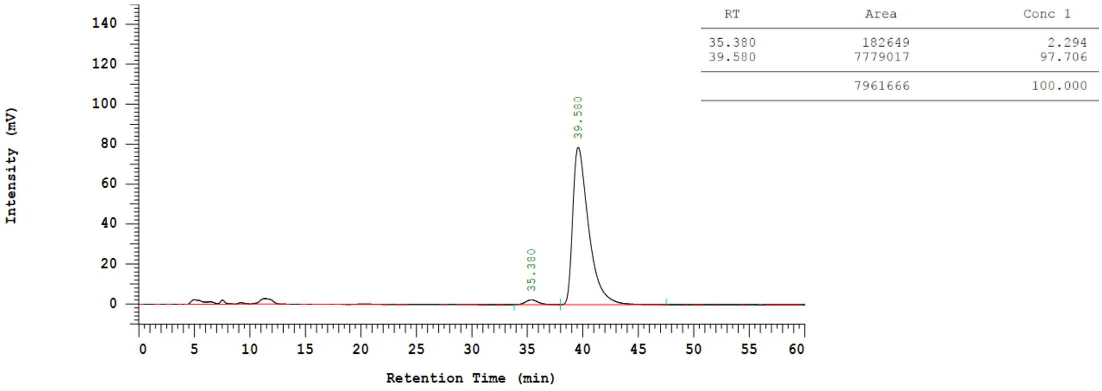

RT	Area	Conc 1
35.380	182649	2.294
39.580	7779017	97.706
	7961666	100.000

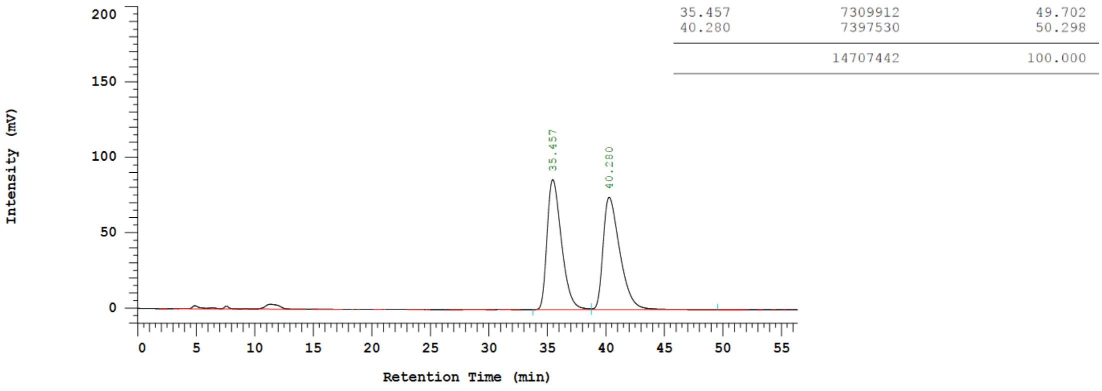

RT	Area	Conc 1
35.457	7309912	49.702
40.280	7397530	50.298
	14707442	100.000

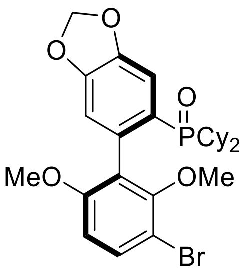

(*R*)-(6-(3-bromo-2,6-dimethoxyphenyl)benzo[*d*][1,3]dioxol-5-yl)dicyclohexylphosphine oxide (36)

Appearance: colorless foam;

Yield: 89%; **e.e**: 93%; $[\alpha]_D^{25}$ = 31 (*c* 0.1, $CHCl_3$);

^{1}H NMR (500 MHz, $CDCl_3$): δ = 7.50 (d, *J* = 9.0 Hz, 1H), 7.35 (d, *J* = 10.4 Hz, 1H), 6.64 (d, *J* = 2.8 Hz, 1H), 6.60 (d, *J* = 9.0 Hz, 1H), 6.06 (d, *J* = 5.7 Hz, 2H), 3.69 (s, 3H), 3.54 (s, 3H), 1.82 – 1.67 (m, 6H), 1.65 – 1.50 (m, 4H), 1.49 – 1.39 (m, 3H), 1.37 – 1.25 (m, 3H), 1.23 – 1.09 (m, 4H), 1.08 – 0.95 (m, 2H) ppm.

^{13}C NMR (126 MHz, $CDCl_3$): δ = 157.46, 155.37, 149.19 (d, *J* = 2.7 Hz), 147.03 (d, *J* = 15.1 Hz), 132.76, 131.89 (d, *J* = 7.8 Hz), 126.09 (d, *J* = 2.4 Hz), 124.94 (d, *J* = 84.2 Hz), 112.59 (d, *J* = 11.6 Hz), 112.36 (d, *J* = 9.6 Hz), 108.18, 107.44, 101.72, 60.66, 55.73, 37.90 (d, *J* = 63.9 Hz), 37.37 (d, *J* = 63.9 Hz), 26.85 – 26.49 (m), 26.31 (d, *J* = 3.2 Hz), 26.02 – 25.64 (m) ppm.

^{31}P NMR (202 MHz, $CDCl_3$): δ = 47.40 ppm.

HRMS (ESI$^+$): calcd $[M+Na]^+$ for $[C_{27}H_{34}BrO_5PNa]^+$: 571.12194, found 571.12176.

HPLC condition: Daicel Chiralpak IF, i-PrOH/n-hexane = 20/80, 0.8 mL/min, 254 nm UV detector; t_1= 26.5 min (major), t_2 = 31.6 min (minor).

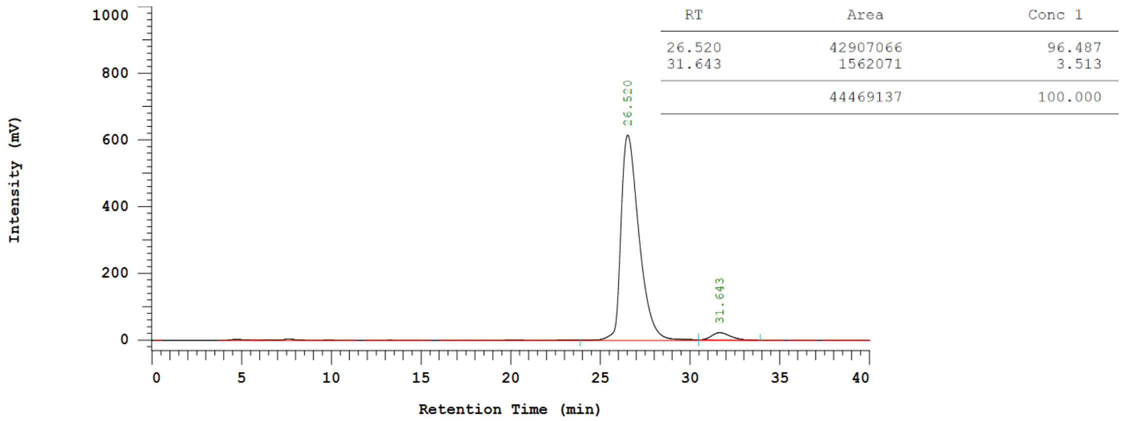

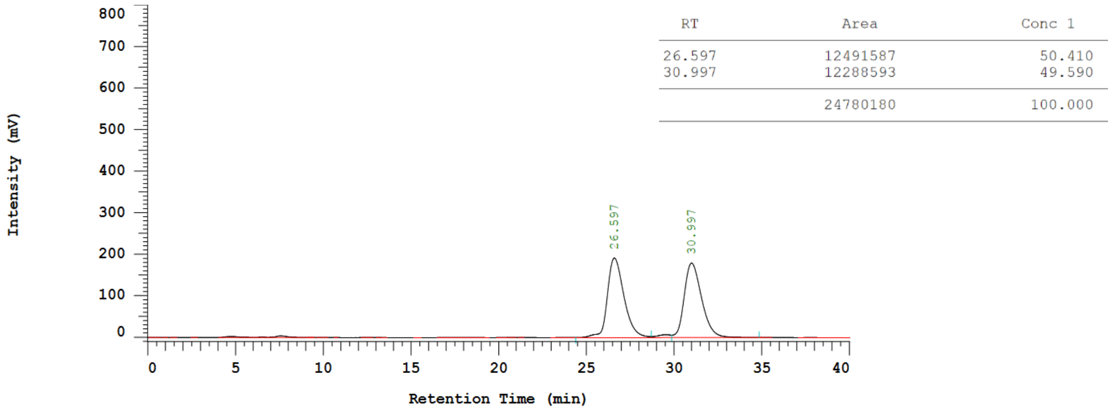

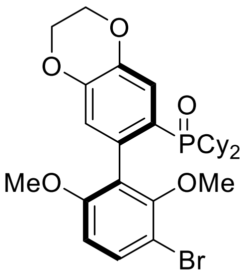

(*R*)-(7-(3-bromo-2,6-dimethoxyphenyl)-2,3-dihydrobenzo[*b*][1,4]dioxin-6-yl)dicyclohexylphosphine oxide (37)

Appearance: colorless foam;

Yield: 85%; **e.e**: 90%; $[\alpha]_D^{25}$ = 38 (*c* 0.1, $CHCl_3$);

^{1}H NMR (500 MHz, $CDCl_3$): δ = 7.48 (d, *J* = 8.8 Hz, 1H), 7.37 (d, *J* = 11.3 Hz, 1H), 6.69 (s, 1H), 6.58 (d, *J* = 8.9 Hz, 1H), 4.32 (s, 4H), 3.68 (s, 3H), 3.53 (s, 3H), 1.82 – 1.37 (m, 14H), 1.35 – 1.11 (m, 6H), 1.10 – 0.93 (m, 2H) ppm.

^{13}C NMR (126 MHz, $CDCl_3$): δ = 157.55, 155.39, 144.88, 142.49 (d, *J* = 14.3 Hz), 132.57, 130.35, 125.96, 124.21 (d, *J* = 84.2 Hz), 122.07 (d, *J* = 9.7 Hz), 121.14 (d, *J* = 11.0 Hz), 108.15, 107.41, 64.55, 64.27, 60.69, 55.73, 37.79 (d, *J* = 57.4 Hz), 37.26 (d, *J* = 57.2 Hz), 26.90 – 26.39 (m), 26.34 – 25.50 (m) ppm.

^{31}P NMR (202 MHz, $CDCl_3$): δ = 46.76 ppm.

HRMS (ESI$^+$): calcd $[M+Na]^+$ for $[C_{28}H_{36}BrO_5PNa]^+$: 585.13759, found 585.13721.

HPLC condition: Daicel Chiralpak IC, i-PrOH/n-hexane = 20/80, 0.8 mL/min, 254 nm UV detector; t_1= 40.2 min (major), t_2 = 46.4 min (minor).

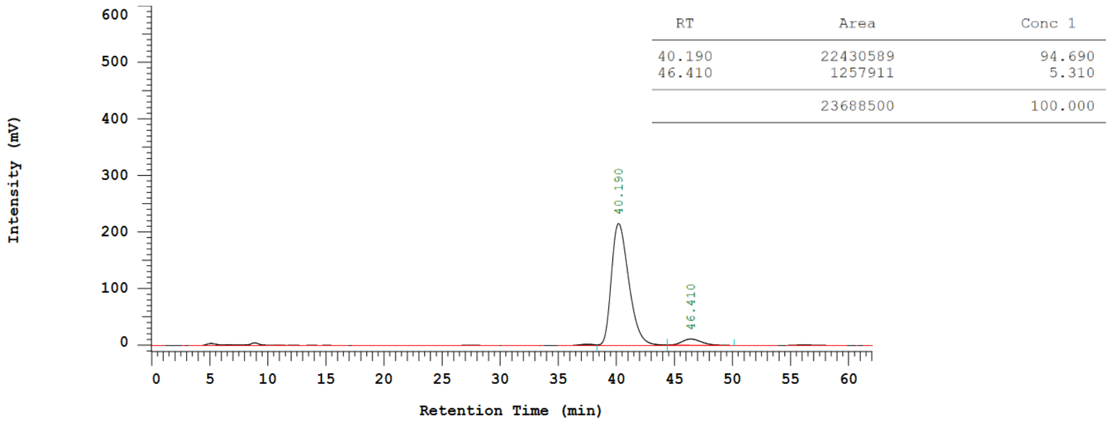

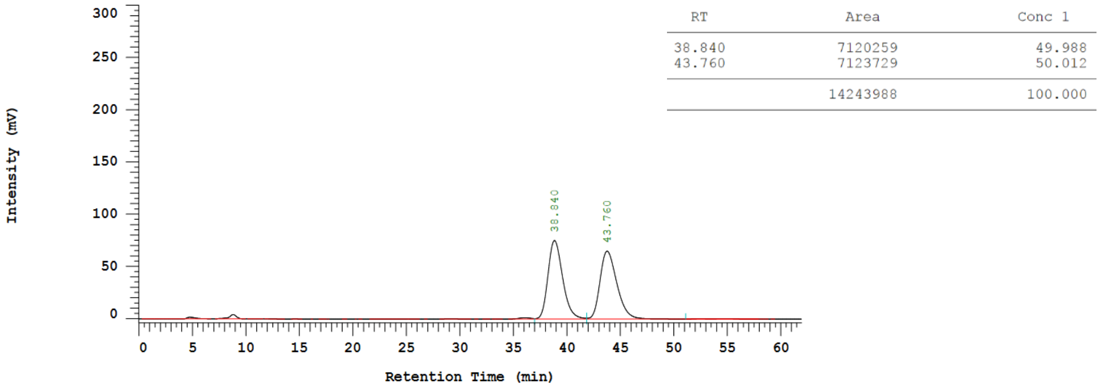

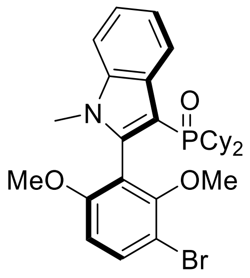

(R)-(2-(3-bromo-2,6-dimethoxyphenyl)-1-methyl-1H-indol-3-yl)dicyclohexylphosphine oxide (38)

Appearance: colorless foam;

Yield: 84%; **e.e**: 99%; **$[\alpha]_D^{25}$** = -22 (c 0.1, $CHCl_3$);

^{1}H NMR (500 MHz, $CDCl_3$): δ = 7.93 (d, J = 8.0 Hz, 1H), 7.63 (d, J = 8.9 Hz, 1H), 7.43 (d, J = 8.2 Hz, 1H), 7.33 (t, J = 7.6 Hz, 1H), 7.24 (t, J = 7.5 Hz, 1H), 6.68 (d, J = 9.0 Hz, 1H), 3.72 (s, 3H), 3.59 (s, 3H), 3.51 (s, 3H), 2.04 – 1.88 (m, 4H), 1.86 – 1.70 (m, 4H), 1.68 – 1.53 (m, 5H), 1.49 – 1.33 (m, 3H), 1.25 – 1.13 (m, 5H), 1.12 – 1.01 (m, 1H) ppm.

^{13}C NMR (126 MHz, $CDCl_3$): δ = 175.72, 172.27 (d, J = 267.7 Hz), 158.31, 156.59, 137.68 (d, J = 9.9 Hz), 134.56, 129.30 (d, J = 9.8 Hz), 122.06, 121.61, 120.73, 116.61, 109.92, 107.96, 107.45, 82.36 – 71.64 (m), 60.69, 55.56, 39.02 (d, J = 70.3 Hz), 37.80 (d, J = 69.2 Hz), 30.37, 27.07 – 26.46 (m), 25.95 (d, J = 24.4 Hz), 25.73 (d, J = 2.8 Hz), 25.49 (d, J = 3.2 Hz), 25.25 (d, J = 3.2 Hz), 18.55 ppm.

^{31}P NMR (202 MHz, $CDCl_3$): δ = 45.53 ppm.

HRMS (ESI$^+$): calcd $[M+Na]^+$ for $[C_{29}H_{37}BrNO_3PNa]^+$: 580.15866, found 580.15849.

HPLC condition: Daicel Chiralpak IA, i-PrOH/n-hexane = 10/90, 0.8 mL/min, 254 nm UV detector; t_1= 21.4 min (minor), t_2 = 27.0 min (major).

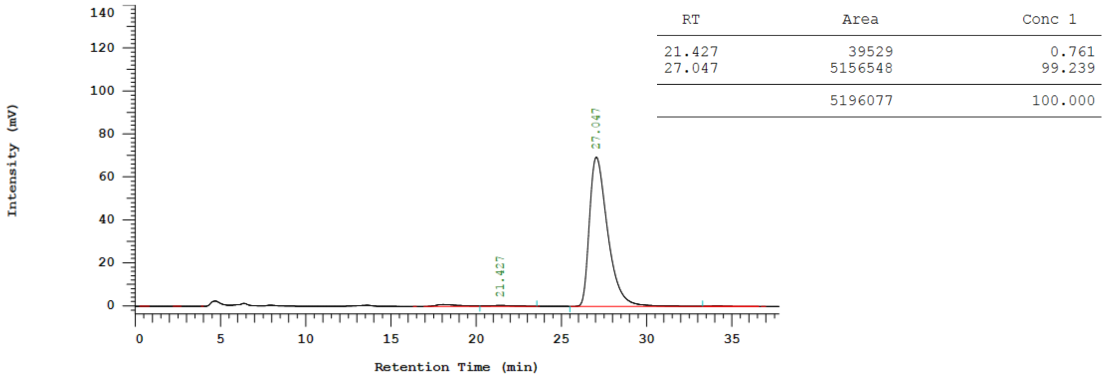

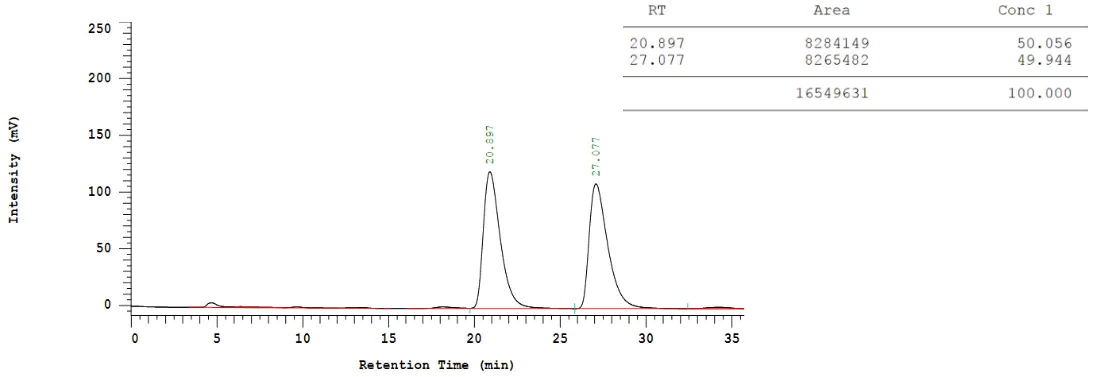

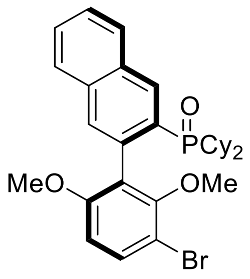

(*R*)-(3-(3-bromo-2,6-dimethoxyphenyl)naphthalen-2-yl)dicyclohexylphosphine oxide (39)

Appearance: white solid;

Yield: 94%; **e.e**: 97%; **[*α*]$_D^{25}$** = 82 (*c* 0.1, $CHCl_3$);

^{1}H NMR (500 MHz, $CDCl_3$): δ = 8.60 (d, *J* = 12.4 Hz, 1H), 8.02 (d, *J* = 8.6 Hz, 1H), 7.84 – 7.79 (m, 1H), 7.66 (d, *J* = 3.6 Hz, 1H), 7.61 – 7.53 (m, 3H), 6.67 (d, *J* = 8.9 Hz, 1H), 3.67 (s, 3H), 3.49 (s, 3H), 1.89 – 1.69 (m, 5H), 1.69 – 1.43 (m, 8H), 1.38 – 1.10 (m, 7H), 1.08 – 0.92 (m, 2H). ppm.

^{13}C NMR (126 MHz, $CDCl_3$): δ = 157.74, 155.63, 135.57 (d, *J* = 6.5 Hz), 133.64 (d, *J* = 2.1 Hz), 132.96, 132.11 (d, *J* = 8.0 Hz), 131.64 (d, *J* = 11.0 Hz), 131.16 (d, *J* = 9.0 Hz), 129.92 (d, *J* = 81.5 Hz), 128.87, 127.84, 127.41, 126.71, 126.57, 108.34, 107.64, 60.64, 55.83, 37.93 (d, *J* = 36.4 Hz), 37.40 (d, *J* = 36.2 Hz), 26.88, 26.83 – 26.53 (m), 26.35 (d, *J* = 13.1 Hz), 26.14 (d, *J* = 3.5 Hz), 25.97 – 25.64 (m) ppm.

^{31}P NMR (202 MHz, $CDCl_3$): δ = 48.17 ppm.

HRMS (ESI$^+$): calcd $[M+Na]^+$ for $[C_{30}H_{36}BrO_3PNa]^+$: 577.14777, found 577.14777.

HPLC condition: Daicel Chiralpak IA, i-PrOH/n-hexane = 10/90, 0.8 mL/min, 254 nm UV detector; t_1= 22.2 min (minor), t_2 = 33.1 min (major).

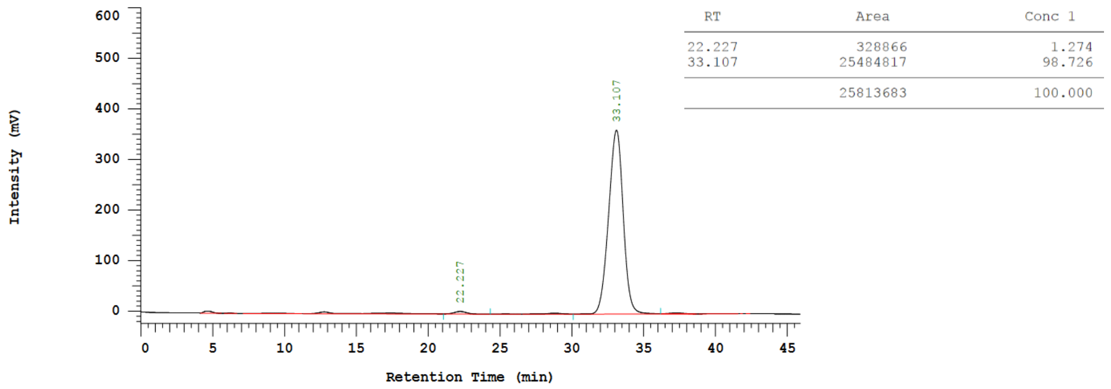

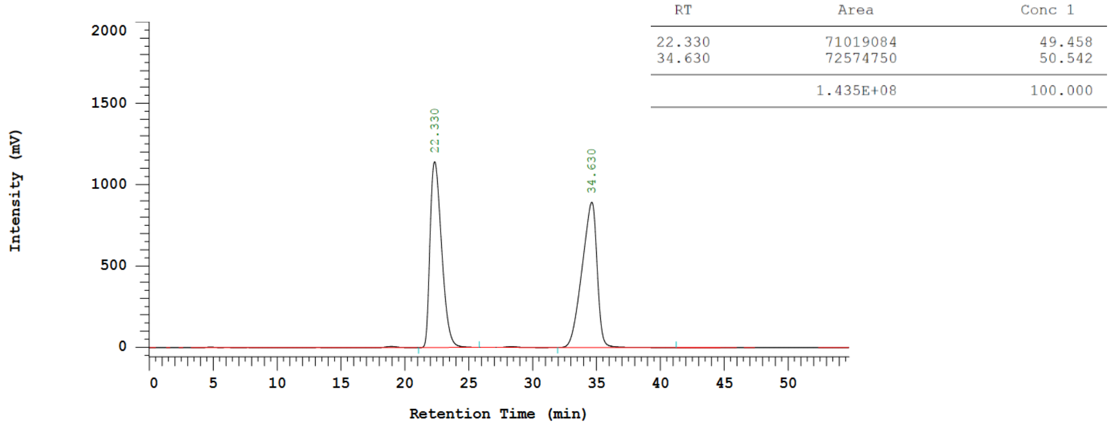

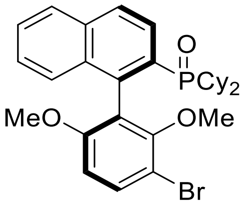

(*R*)-(1-(3-bromo-2,6-dimethoxyphenyl)naphthalen-2-yl)dicyclohexylphosphine oxide (40)

Appearance: colorless foam;

Yield: 89%; **e.e**: 99%; **$[\alpha]_D^{25}$** = 30 (*c* 0.1, $CHCl_3$);

^{1}H NMR (500 MHz, $CDCl_3$): δ = 7.96 – 7.91 (m, 1H), 7.90 – 7.82 (m, 2H), 7.62 (d, *J* = 8.9 Hz, 1H), 7.57 – 7.47 (m, 1H), 7.42 – 7.35 (m, 2H), 6.69 (d, *J* = 8.9 Hz, 1H), 3.59 (s, 3H), 3.24 (s, 3H), 1.99 – 1.51 (m, 12H), 1.51 – 1.27 (m, 4H), 1.24 – 1.03 (m, 6H) ppm.

^{13}C NMR (126 MHz, $CDCl_3$): δ = 157.69, 155.77, 137.46, 134.14 (d, *J* = 2.2 Hz), 133.21 (d, *J* = 10.4 Hz), 133.20, 129.26 (d, *J* = 82.1 Hz), 128.05, 127.72 (d, *J* = 9.2 Hz), 127.28 (d, *J* = 10.6 Hz), 127.27, 126.45, 126.39, 123.46 (d, *J* = 3.2 Hz), 108.34, 107.44, 60.34, 55.68, 38.11 (d, *J* = 65.6 Hz), 37.29 (d, *J* = 66.0 Hz), 26.95 – 26.47 (m), 26.13 (d, *J* = 3.1 Hz), 26.04 – 25.69 (m) ppm.

^{31}P NMR (202 MHz, $CDCl_3$): δ = 47.09 ppm.

HRMS (ESI$^+$): calcd $[M+Na]^+$ for $[C_{30}H_{36}BrO_3PNa]^+$: 577.14777, found 577.14749.

HPLC condition: Daicel Chiralpak ID, i-PrOH/n-hexane = 10/90, 0.8 mL/min, 254 nm UV detector; t_1= 33.7 min (minor), t_2 = 36.2 min (major).

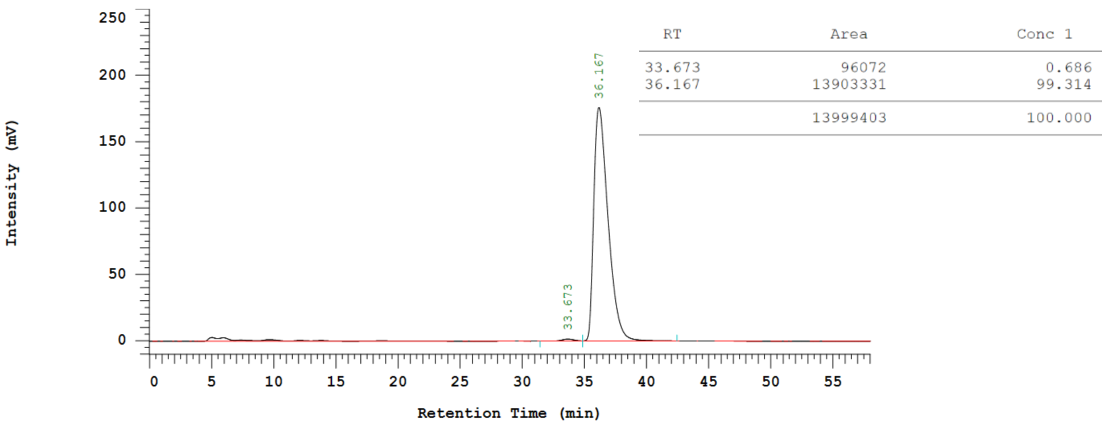

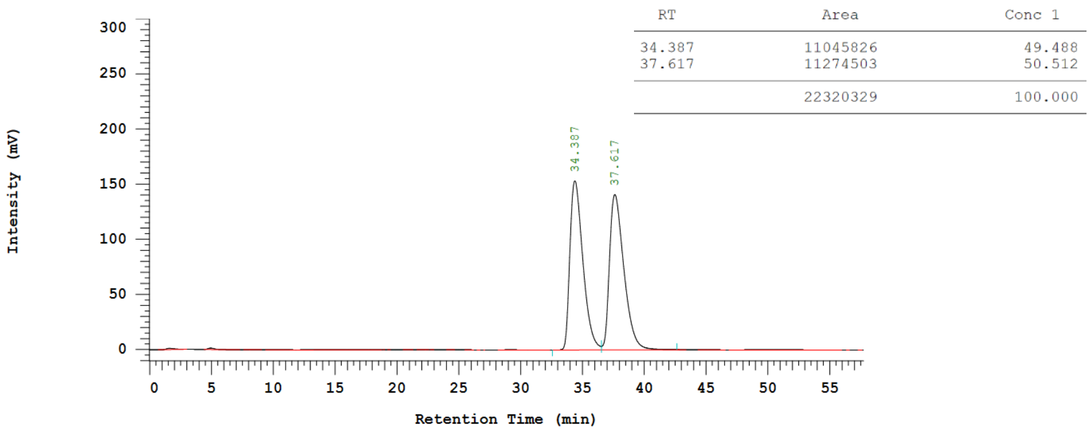

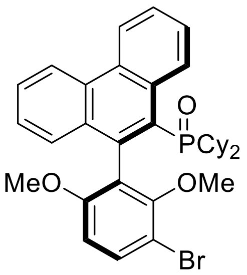

(*R*)-(10-(3-bromo-2,6-dimethoxyphenyl)phenanthren-9-yl)dicyclohexylphosphine oxide (41)

Appearance: colorless foam;

Yield: 92%; **e.e**: 99%; $[\alpha]_D^{25}$ = 63 (*c* 0.1, $CHCl_3$);

^{1}H NMR (500 MHz, $CDCl_3$): δ = 8.83 (d, *J* = 8.2 Hz, 1H), 8.73 (d, *J* = 8.4 Hz, 1H), 8.10 – 7.92 (m, 1H), 7.75 – 7.63 (m, 3H), 7.56 (d, *J* = 8.9 Hz, 1H), 7.51 – 7.43 (m, 2H), 6.66 (d, *J* = 8.9 Hz, 1H), 3.59 (s, 3H), 3.15 (s, 3H), 2.41 – 2.23 (m, 2H), 2.07 – 1.98 (m, 2H), 1.90 – 1.77 (m, 2H), 1.74 – 1.45 (m, 9H), 1.37 – 1.19 (m, 5H), 1.18 – 1.01 (m, 2H) ppm.

^{13}C NMR (126 MHz, $CDCl_3$): δ = 157.51, 154.90, 142.26, 132.19, 131.22, 130.75 (d, *J* = 9.0 Hz), 130.21 (d, *J* = 8.2 Hz), 129.04, 128.18, 127.39, 126.86, 126.53, 126.34, 125.99, 124.91 (d, *J* = 3.9 Hz), 123.63, 122.39, 107.83, 106.85, 99.42 (d, *J* = 15.5 Hz), 59.93, 55.44, 41.58 (d, *J* = 17.3 Hz), 41.06 (d, *J* = 17.7 Hz), 27.77 – 26.45 (m), 26.35 – 25.57 (m) ppm.

^{31}P NMR (202 MHz, $CDCl_3$): δ = 48.32 ppm.

HRMS (ESI$^+$): calcd $[M+Na]^+$ for $[C_{34}H_{38}BrO_3PNa]^+$: 627.16342, found 627.16294.

HPLC condition: Daicel Chiralpak IC, i-PrOH/n-hexane = 5/95, 0.8 mL/min, 254 nm UV detector; t_1= 19.5 min (major), t_2 = 27.5 min (minor).

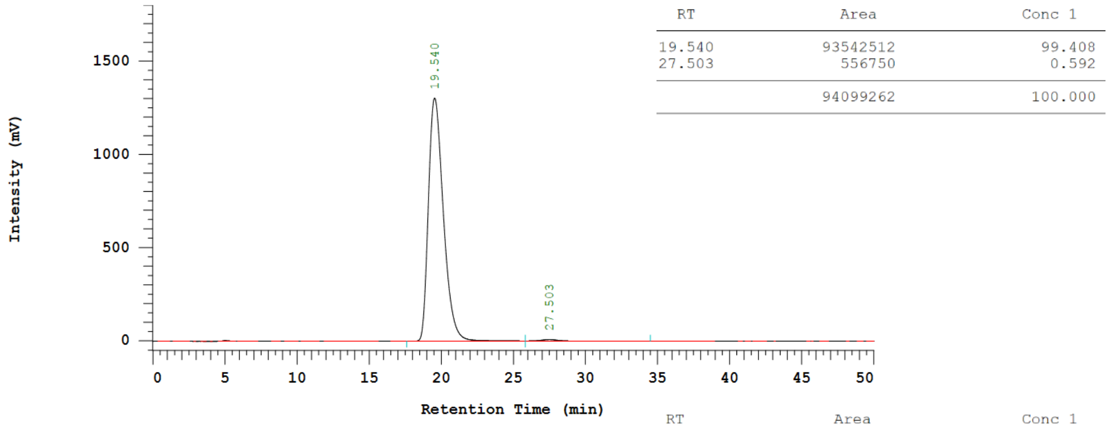

RT	Area	Conc 1
19.540	93542512	99.408
27.503	556750	0.592
	94099262	100.000

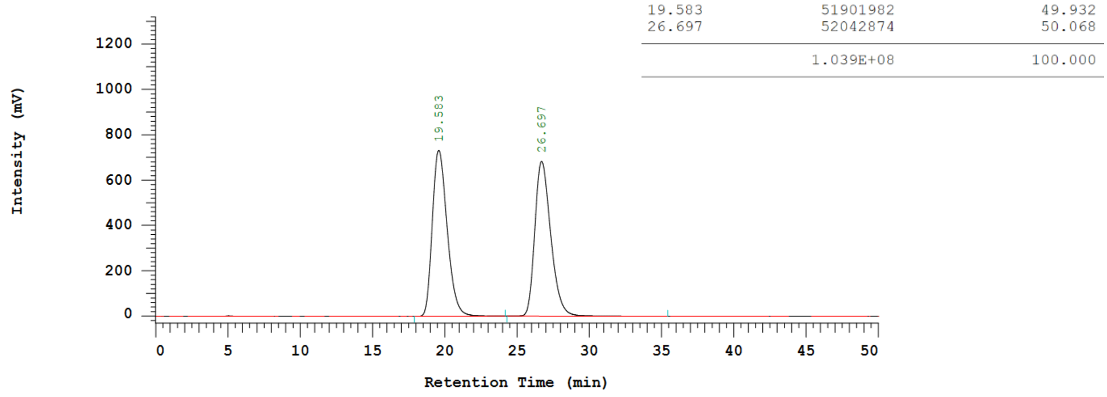

RT	Area	Conc 1
19.583	51901982	49.932
26.697	52042874	50.068
	1.039E+08	100.000

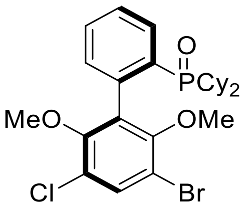

(*R*)-(3'-bromo-5'-chloro-2',6'-dimethoxy-[1,1'-biphenyl]-2-yl)dicyclohexylphosphine oxide (42)

Appearance: colorless foam;

Yield: 99%; $[\alpha]_D^{25}$ = 38 (*c* 0.1, $CHCl_3$);

^{1}H NMR (500 MHz, $CDCl_3$): δ = 7.80 – 7.71 (m, 1H), 7.58 (s, 1H), 7.56 – 7.44 (m, 2H), 7.27 – 7.22 (m, 1H), 3.54 (s, 3H), 3.49 (s, 3H), 1.87 – 1.72 (m, 6H), 1.71 – 1.52 (m, 6H), 1.51 – 1.39 (m, 2H), 1.33 – 1.22 (m, 2H), 1.21 – 1.08 (m, 6H) ppm.

^{13}C NMR (126 MHz, $CDCl_3$): δ = 153.84 (d, *J* = 51.5 Hz), 152.96, 137.07 (d, *J* = 5.9 Hz), 133.15 (d, *J* = 2.3 Hz), 132.95, 132.41 (d, *J* = 8.6 Hz), 132.04 (d, *J* = 9.0 Hz), 131.83 (d, *J* = 82.3 Hz), 130.00, 127.48 (d, *J* = 10.2 Hz), 123.79, 111.93, 60.71, 60.63, 37.70 (d, *J* = 7.8 Hz), 37.18 (d, *J* = 7.8 Hz), 26.83 – 26.44 (m), 26.17 – 25.76 (m) ppm.

^{31}P NMR (202 MHz, $CDCl_3$): δ = 47.67 ppm.

HRMS (ESI$^+$): calcd $[M+Na]^+$ for $[C_{26}H_{33}BrClO_3PNa]^+$: 561.09314, found 561.09288.

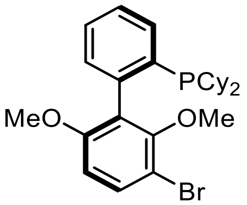

(*R*)-(3'-bromo-2',6'-dimethoxy-[1,1'-biphenyl]-2-yl)dicyclohexylphosphane ((*R*)-SPhos-Br)

Appearance: colorless foam;

Yield: 82%; $[\alpha]_D^{25}$ = -26 (*c* 0.1, $CHCl_3$);

^{1}H NMR (400 MHz, $CDCl_3$): δ = 7.66 – 7.55 (m, 1H), 7.50 (d, *J* = 8.9 Hz, 1H), 7.44 – 7.31 (m, 2H), 7.24 – 7.17 (m, 1H), 6.60 (d, *J* = 8.8 Hz, 1H), 3.67 (s, 3H), 3.44 (s, 3H), 2.01 – 1.81 (m, 1H), 1.80 – 1.57 (m, 10H), 1.57 – 1.44 (m, 1H), 1.33 – 0.95 (m, 10H) ppm.

^{13}C NMR (101 MHz, $CDCl_3$): δ = 157.11, 154.90, 141.89 (d, *J* = 32.3 Hz), 136.62 (d, *J* = 21.6 Hz), 132.55 (d, *J* = 3.0 Hz), 132.20, 130.94 (d, *J* = 6.0 Hz), 127.99, 127.43 (d, *J* = 8.1 Hz), 126.82, 108.24, 107.28, 60.28, 55.51, 34.95 (d, *J* = 14.3 Hz), 33.63 (d, *J* = 13.2 Hz), 30.11 (d, *J* = 17.6 Hz), 29.54 (dd, *J* = 20.4, 11.9 Hz), 27.86 – 27.47 (m), 27.35 (dd, *J* = 10.2, 5.1 Hz), 26.48 (d, *J* = 8.0 Hz) ppm.

^{31}P NMR ($CDCl_3$): δ = -8.39 ppm.

HRMS (ESI$^-$): calcd $[M+Na]^+$ for $[C_{26}H_{34}BrO_2PNa]^-$: 511.13720, found 511.13718.

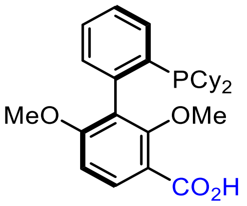

(*R*)-2'-(dicyclohexylphosphaneyl)-2,6-dimethoxy-[1,1'-biphenyl]-3-carboxylic acid ((*R*)-SPhos-CO_2H)

Appearance: white solid;

Yield: 72%; $[\alpha]_D^{25}$ = -18 (*c* 0.1, $CHCl_3$);

^{1}H NMR (400 MHz, Acetone-*d*$_6$ and CD_3OD): δ = 8.01 (d, *J* = 8.9 Hz, 1H), 7.67 – 7.59 (m, 1H), 7.43 – 7.34 (m, 2H), 7.31 – 7.20 (m, 1H), 6.91 (d, *J* = 8.9 Hz, 1H), 3.76 (s, 3H), 3.44 (s, 3H), 1.79 – 1.47 (m, 11H), 1.43 – 0.80 (m, 11H) ppm.

^{13}C NMR (101 MHz, Acetone-*d*$_6$ and CD_3OD): δ = 165.59, 161.57, 158.37, 141.40 (d, *J* = 32.4 Hz), 136.61 (d, *J* = 20.3 Hz), 132.73, 132.62 (d, *J* = 3.5 Hz), 131.52 (d, *J* = 5.9 Hz), 127.97, 126.83, 125.73 (d, *J* = 7.0 Hz), 115.46, 106.41, 61.13, 55.32, 34.99 (d, *J* = 15.6 Hz), 33.00 (d, *J* = 14.2 Hz), 29.44 (dd, *J* = 38.8, 19.4 Hz), 27.47 – 26.74 (m), 26.36 ppm.

^{31}P NMR (202 MHz, Acetone-*d*$_6$ and CD_3OD): δ = -10.08 ppm.

HRMS (ESI$^-$): calcd $[M-H]^-$ for $[C_{27}H_{34}O_4P]^-$: 453.22002, found 453.22011.

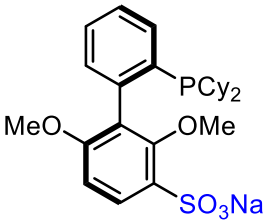

(*R*)-sodium 2'-(dicyclohexylphosphaneyl)-2,6-dimethoxy-[1,1'-biphenyl]-3-sulfonate ((*R*)-SPhos-SO_3Na)

Appearance: colorless foam;

Yield: 53%; $[\alpha]_D^{25}$ = 28 (*c* 0.1, $CHCl_3$);

^{1}H NMR (500 MHz, CD_3OD): δ = 7.89 (d, *J* = 8.8 Hz, 1H), 7.66 – 7.55 (m, 1H), 7.41 – 7.29 (m, 2H), 7.27 – 7.18 (m, 1H), 6.77 (d, *J* = 8.8 Hz, 1H), 3.69 (s, 3H), 3.40 (s, 3H), 2.06 – 1.94 (m, 1H), 1.87 – 1.49 (m, 11H), 1.38 – 0.94 (m, 10H) ppm.

^{13}C NMR (126 MHz, CD_3OD): δ = 161.83, 157.74, 143.73 (d, *J* = 32.4 Hz), 138.47 (d, *J* = 17.3 Hz), 134.21 (d, *J* = 3.3 Hz), 133.79 (d, *J* = 6.3 Hz), 132.47, 130.56, 129.73, 128.44, 128.38, 106.35, 62.11, 56.59, 37.43 (d, *J* = 14.5 Hz), 35.29 (d, *J* = 12.9 Hz), 32.16 (d, *J* = 5.0 Hz), 32.04, 31.61 (d, *J* = 12.3 Hz), 31.31 (d, *J* = 12.6 Hz), 29.31 (t, *J* = 10.2 Hz), 29.03 – 28.66 (m), 28.23 (d, *J* = 13.0 Hz) ppm.

^{31}P NMR (202 MHz, CD_3OD): δ = -7.19 ppm.

HRMS (ESI$^-$): calcd $[M-Na]^-$ for $[C_{26}H_{34}O_5PS]^-$: 489.18700, found 489.18728.

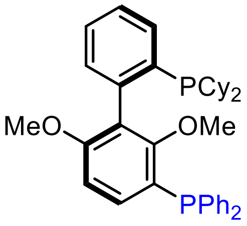

(*R*)-dicyclohexyl(3'-(diphenylphosphaneyl)-2',6'-dimethoxy-[1,1'-biphenyl]-2-yl)phosphane ((*R*)-SPhos-PPh$_2$)

Appearance: white solid;

Yield: 85%; **[*α*]$_D^{25}$** = -34 (*c* 0.1, CHCl$_3$);

^{1}H NMR (500 MHz, CD$_2$Cl$_2$): δ = 7.63 – 7.56 (m, 1H), 7.42 – 7.30 (m, 12H), 7.28 – 7.23 (m, 1H), 6.67 – 6.59 (m, 2H), 3.65 (s, 3H), 3.23 (s, 3H), 2.00 – 1.90 (m, 1H), 1.84 – 1.53 (m, 10H), 1.45 – 1.35 (m, 1H), 1.31 – 0.91 (m, 10H) ppm.

^{13}C NMR (126 MHz, CD$_2$Cl$_2$): δ = 170.84, 160.54 (d, *J* = 17.7 Hz), 159.15, 142.23 (d, *J* = 33.0 Hz), 137.90 (d, *J* = 8.9 Hz), 137.81 (d, *J* = 9.2 Hz), 137.06 (d, *J* = 19.9 Hz), 134.58 (d, *J* = 20.9 Hz), 133.41 (d, *J* = 2.9 Hz), 133.27, 133.25, 132.68 (d, *J* = 3.6 Hz), 131.55 (d, *J* = 5.9 Hz), 128.70, 128.43 (d, *J* = 7.1 Hz), 128.17 (d, *J* = 6.4 Hz), 127.91, 127.75, 126.58, 125.34, 121.59 (d, *J* = 9.3 Hz), 106.25, 60.29 (d, *J* = 4.3 Hz), 55.25, 35.39 (d, *J* = 15.6 Hz), 33.36 (d, *J* = 13.7 Hz), 30.30 (d, *J* = 18.9 Hz), 29.98 (d, *J* = 14.7 Hz), 29.42 (dd, *J* = 10.8, 5.0 Hz), 27.60 (dd, *J* = 9.4, 4.6 Hz), 27.17 (dd, *J* = 10.2, 6.1 Hz), 26.49 (d, *J* = 9.5 Hz), 20.79, 14.00 ppm.

^{31}P NMR (202 MHz, CD$_2$Cl$_2$): δ = -9.31, -15.81 ppm.

HRMS (ESI$^+$): calcd [M+Na]$^+$ for [C$_{38}$H$_{44}$O$_2$P$_2$Na]$^+$: 617.27087, found 617.27070.

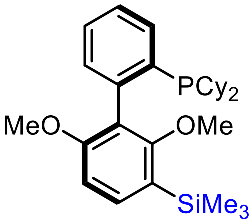

(*R*)-dicyclohexyl(2',6'-dimethoxy-3'-(trimethylsilyl)-[1,1'-biphenyl]-2-yl)phosphane ((*R*)-SPhos-TMS)

Appearance: colorless foam;

Yield: 92%; **[*α*]$_D^{25}$** = -11 (*c* 0.1, CHCl$_3$);

^{1}H NMR (500 MHz, CD$_2$Cl$_2$): δ = 7.65 – 7.53 (m, 1H), 7.41 – 7.32 (m, 3H), 7.31 – 7.24 (m, 1H), 6.71 (d, *J* = 8.2 Hz, 1H), 3.67 (s, 3H), 3.13 (s, 3H), 2.00 – 1.88 (m, 1H), 1.85 – 1.44 (m, 10H), 1.36 – 0.83 (m, 10H), 0.27 (s, 9H) ppm.

^{13}C NMR (126 MHz, CD$_2$Cl$_2$): δ = 163.80, 159.46, 142.85 (d, *J* = 32.7 Hz), 137.32, 134.57, 132.86 (d, *J* = 3.3 Hz), 131.85 (d, *J* = 6.0 Hz), 127.78, 126.42, 124.02, 123.19, 105.32, 60.17, 55.25, 35.67 (d, *J* = 14.9 Hz), 33.30 (d, *J* = 14.6 Hz), 30.60 (d, *J* = 19.6 Hz), 29.99 – 28.88 (m), 28.08 – 26.88 (m), 26.54 (d, *J* = 13.5 Hz), 20.79, 14.00, -0.66 ppm.

^{31}P NMR (202 MHz, CD$_2$Cl$_2$): δ = -9.06 ppm.

HRMS (ESI$^+$): calcd [M+Na]$^+$ for [C$_{29}$H$_{43}$O$_2$PSiNa]$^+$: 505.26621, found 505.26622.

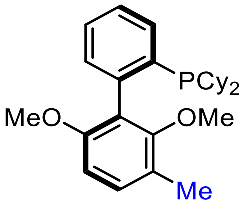

(*R*)-dicyclohexyl(2',6'-dimethoxy-3'-methyl-[1,1'-biphenyl]-2-yl)phosphane ((*R*)-SPhos-Me)

Appearance: colorless foam;

Yield: 88%; $[\alpha]_D^{25}$ = -21 (*c* 0.1, $CHCl_3$);

^{1}H NMR (500 MHz, CD_2Cl_2): δ = 7.64 – 7.55 (m, 1H), 7.40 – 7.29 (m, 2H), 7.24 – 7.17 (m, 1H), 7.14 (d, *J* = 8.4 Hz, 1H), 6.62 (d, *J* = 8.4 Hz, 1H), 3.65 (s, 3H), 3.32 (s, 3H), 2.25 (s, 3H), 2.00 – 1.89 (m, 1H), 1.82 – 1.46 (m, 11H), 1.35 – 0.95 (m, 10H) ppm.

^{13}C NMR (126 MHz, CD_2Cl_2): δ = 156.47, 155.98, 142.95 (d, *J* = 32.6 Hz), 136.97 (d, *J* = 18.6 Hz), 132.47 (d, *J* = 3.4 Hz), 131.27 (d, *J* = 6.0 Hz), 129.84, 127.65, 126.30, 125.43 (d, *J* = 6.7 Hz), 122.64, 105.40, 59.51, 55.14, 35.12 (d, *J* = 15.0 Hz), 33.57 (d, *J* = 13.9 Hz), 30.73 – 29.28 (m), 27.89 – 26.97 (m), 26.54, 15.69 ppm.

^{31}P NMR (202 MHz, CD_2Cl_2): δ = -9.13 ppm.

HRMS (ESI$^+$): calcd $[M+Na]^+$ for $[C_{27}H_{37}O_2PNa]^+$: 447.24234, found 447.24193.

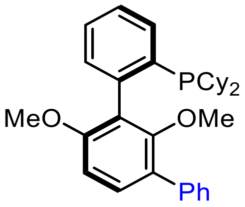

(*R*)-dicyclohexyl(2',6'-dimethoxy-[1,1':3',1''-terphenyl]-2-yl)phosphane ((*R*)-SPhos-Ph)

Appearance: colorless foam;

Yield: 96%; $[\alpha]_D^{25}$ = -14 (*c* 0.1, $CHCl_3$);

^{1}H NMR (500 MHz, CD_2Cl_2): δ = 7.65 – 7.60 (m, 1H), 7.59 – 7.55 (m, 2H), 7.43 – 7.28 (m, 6H), 7.25 – 7.18 (m, 1H), 6.78 (d, *J* = 8.6 Hz, 1H), 3.72 (s, 3H), 3.09 (s, 3H), 1.93 – 1.52 (m, 12H), 1.36 – 0.91 (m, 10H) ppm.

^{13}C NMR (126 MHz, CD_2Cl_2): δ = 157.09, 155.88, 143.03 (d, *J* = 32.7 Hz), 139.14, 136.87 (d, *J* = 19.0 Hz), 132.60 (d, *J* = 3.6 Hz), 130.78 (d, *J* = 6.1 Hz), 130.24, 128.95, 128.19, 127.82, 127.03, 126.49, 126.37, 105.76, 60.05, 55.30, 34.47 (d, *J* = 14.2 Hz), 34.04 (d, *J* = 14.2 Hz), 30.39 (d, *J* = 17.6 Hz), 30.08 – 29.73 (m), 29.53 (d, *J* = 9.9 Hz), 27.87 – 27.09 (m), 26.57 ppm.

^{31}P NMR (202 MHz, CD_2Cl_2): δ = -8.74 ppm.

HRMS (ESI$^+$): calcd $[M+Na]^+$ for $[C_{32}H_{39}O_2PNa]^+$: 509.25799, found 509.25790.

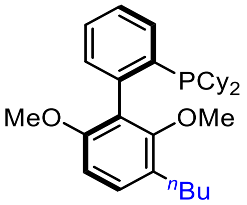

(*R*)-(3'-butyl-2',6'-dimethoxy-[1,1'-biphenyl]-2-yl)dicyclohexylphosphane ((*R*)-SPhos-^{n}Bu)

Appearance: colorless foam;

Yield: 91%; **$[\alpha]_D^{25}$** = -12 (*c* 0.1, $CHCl_3$);

^{1}H NMR (500 MHz, CD_2Cl_2): δ = 7.63 – 7.53 (m, 1H), 7.40 – 7.30 (m, 2H), 7.26 – 7.19 (m, 1H), 7.14 (d, *J* = 8.5 Hz, 1H), 6.64 (d, *J* = 8.5 Hz, 1H), 3.65 (s, 3H), 3.29 (s, 3H), 2.65 – 2.49 (m, 2H), 1.98 – 1.83 (m, 1H), 1.79 – 1.61 (m, 8H), 1.60 – 1.47 (m, 5H), 1.40 (h, *J* = 7.4 Hz, 2H), 1.33 – 1.11 (m, 7H), 1.10 – 0.98 (m, 3H), 0.95 (t, *J* = 7.3 Hz, 3H) ppm.

^{13}C NMR (126 MHz, CD_2Cl_2): δ = 156.54, 155.87, 143.09 (d, *J* = 32.6 Hz), 137.10 (d, *J* = 19.1 Hz), 132.53 (d, *J* = 3.5 Hz), 131.29 (d, *J* = 6.0 Hz), 128.93, 127.66, 127.62, 126.26, 125.42 (d, *J* = 7.3 Hz), 105.39, 60.24, 55.13, 35.09 (d, *J* = 15.0 Hz), 33.65 (d, *J* = 14.2 Hz), 33.22, 30.34 (d, *J* = 18.5 Hz), 30.02 – 29.60 (m), 29.50, 27.70 – 27.08 (m), 26.54 (d, *J* = 3.0 Hz), 22.68, 13.83 ppm.

^{31}P NMR (202 MHz, CD_2Cl_2): δ = -8.96 ppm.

HRMS (ESI$^+$): calcd $[M+Na]^+$ for $[C_{30}H_{43}O_2PNa]^+$: 489.28929, found 489.28921.

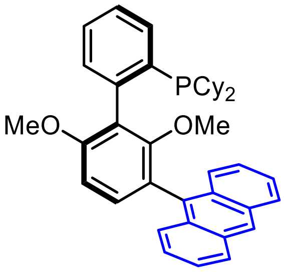

(*R*)-(3'-(anthracen-9-yl)-2',6'-dimethoxy-[1,1'-biphenyl]-2-yl)dicyclohexylphosphane ((*R*)-SPhos-Ant)

Appearance: white solid;

Yield: 90%; **$[\alpha]_D^{25}$** = -33 (*c* 0.1, $CHCl_3$);

^{1}H NMR (500 MHz, CD_2Cl_2): δ = 8.53 (s, 1H), 8.18 – 8.02 (m, 3H), 7.93 (d, *J* = 8.6 Hz, 1H), 7.70 – 7.63 (m, 1H), 7.57 – 7.34 (m, 7H), 7.23 (d, *J* = 8.4 Hz, 1H), 6.93 (d, *J* = 8.5 Hz, 1H), 3.83 (s, 3H), 2.75 (s, 3H), 2.10 – 1.91 (m, 2H), 1.89 – 1.53 (m, 10H), 1.41 – 1.03 (m, 8H), 0.99 – 0.83 (m, 2H) ppm.

^{13}C NMR (126 MHz, CD_2Cl_2): δ = 157.64, 156.97, 143.13 (d, *J* = 32.6 Hz), 137.19 (d, *J* = 18.7 Hz), 134.24, 132.72 (d, *J* = 3.7 Hz), 132.48, 131.53 (d, *J* = 20.5 Hz), 130.97 (d, *J* = 5.9 Hz), 130.86, 130.80, 130.69, 128.57 (d, *J* = 7.0 Hz), 128.44, 127.95, 127.83 (d, *J* = 2.7 Hz), 126.88, 126.45 (d, *J* = 6.8 Hz), 125.85, 125.74, 125.66, 125.41, 125.29 (d, *J* = 2.2 Hz), 124.95, 123.23, 105.60, 59.94, 55.32, 53.45 (p, *J* = 27.2 Hz), 35.38 (d, *J* = 15.0 Hz), 34.01 (d, *J* = 13.7 Hz), 30.82 (d, *J* = 19.1 Hz), 30.50 (d, *J* = 16.3 Hz), 29.96 – 28.95 (m), 27.95 – 27.00 (m), 26.62 (d, *J* = 4.9 Hz) ppm.

^{31}P NMR (202 MHz, CD_2Cl_2): δ = -8.53 ppm.

HRMS (ESI$^+$): calcd $[M+Na]^+$ for $[C_{40}H_{43}O_2PNa]^+$: 609.28929, found 609.28903.

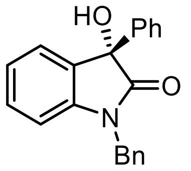

(*S*)-1-benzyl-3-hydroxy-3-phenylindolin-2-one (45)

Appearance: white solid;

e.e: 86%

^{1}H NMR (500 MHz, CDCl$_3$): δ = 7.47 – 7.41 (m, 2H), 7.39 – 7.28 (m, 9H), 7.27 – 7.22 (m, 1H), 7.10 – 7.03 (m, 1H), 6.81 (d, *J* = 7.9 Hz, 1H), 5.07 (d, *J* = 15.7 Hz, 1H), 4.84 (d, *J* = 15.6 Hz, 1H), 3.93 (s, 1H) ppm.

^{13}C NMR (126 MHz, CDCl$_3$): δ = 177.78, 142.63, 140.24, 135.44, 131.80, 129.76, 128.91, 128.67, 128.33, 127.81, 127.33, 125.39, 125.04, 123.62, 109.79, 78.06, 67.96 ppm.

HPLC condition: Daicel Chiralpak IA, i-PrOH/n-hexane = 15/85, 0.8 mL/min, 254 nm UV detector; t_1= 24.4 min (minor), t_2 = 29.5 min (major).

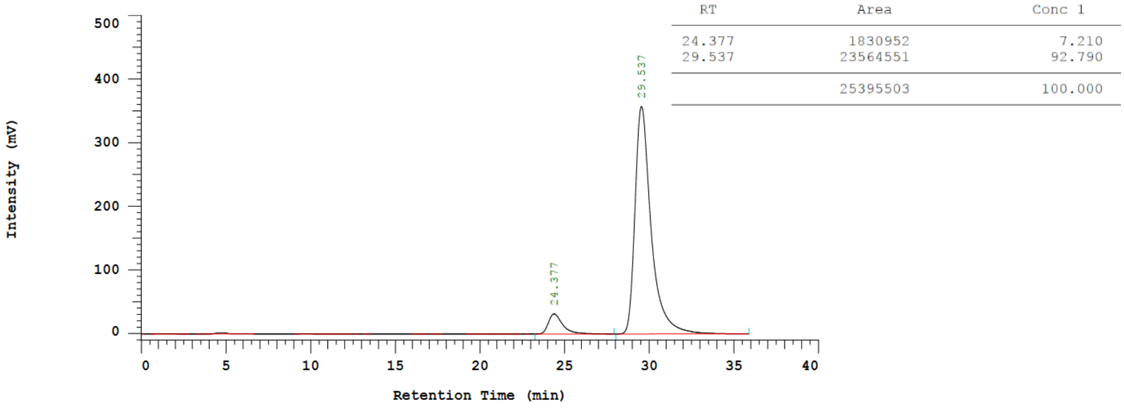

RT	Area	Conc 1
24.377	1830952	7.210
29.537	23564551	92.790
	25395503	100.000

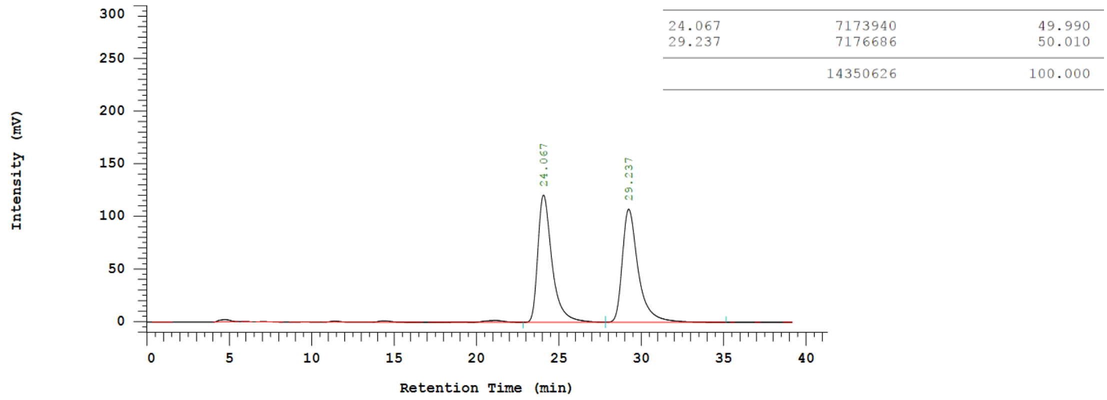

RT	Area	Conc 1
24.067	7173940	49.990
29.237	7176686	50.010
	14350626	100.000

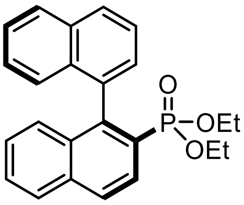

diethyl (*R*)-[1,1'-binaphthalen]-2-ylphosphonate (48)

Appearance: white solid;

e.e: 79%

^{1}H NMR (400 MHz, CDCl$_3$): δ = 8.26 – 8.16 (m, 1H), 8.06 – 7.99 (m, 1H), 8.00 – 7.90 (m, 3H), 7.64 – 7.56 (m, 1H), 7.57 – 7.47 (m, 2H), 7.47 – 7.39 (m, 1H), 7.30 – 7.14 (m, 4H), 7.09 (d, *J* = 8.5 Hz, 1H), 3.85 – 3.49 (m, 4H), 0.97 (t, *J* = 7.1 Hz, 3H), 0.71 (t, *J* = 7.1 Hz, 3H) ppm.

^{13}C NMR (101 MHz, CDCl$_3$): δ = 143.51 (d, *J* = 9.6 Hz), 135.89 (d, *J* = 5.4 Hz), 134.93 (d, *J* = 2.6 Hz), 133.35, 133.19, 133.13, 128.73, 128.71, 128.70, 128.63, 128.15 (d, *J* = 25.0 Hz), 127.93, 127.84, 127.70 (d, *J* = 14.5 Hz), 127.11, 126.72, 126.70, 125.75 (d, *J* = 27.0 Hz), 125.23, 124.95, 61.78 (d, *J* = 6.2 Hz), 61.59 (d, *J* = 6.1 Hz), 15.96 (d, *J* = 6.8 Hz), 15.47 (d, *J* = 7.1 Hz) ppm.

^{31}P NMR (162 MHz, CDCl$_3$): δ = 18.47 ppm.

HPLC condition: Daicel Chiralpak IC, i-PrOH/n-hexane = 7.5/92.5, 0.8 mL/min, 254 nm UV detector; t_1= 38.2 min (major), t_2 = 41.0 min (minor).

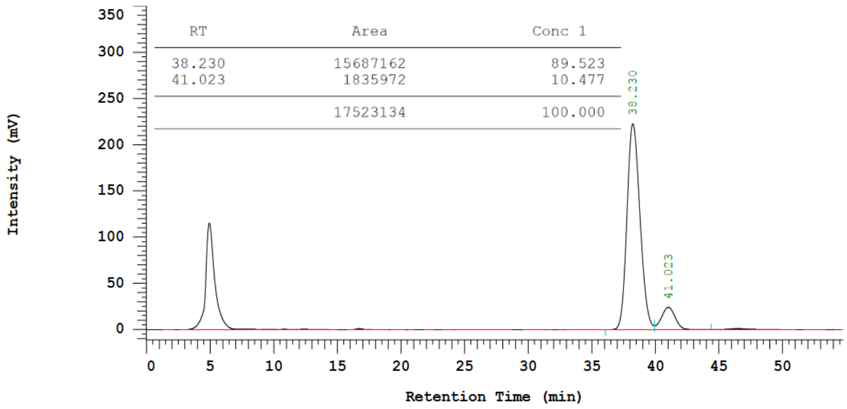

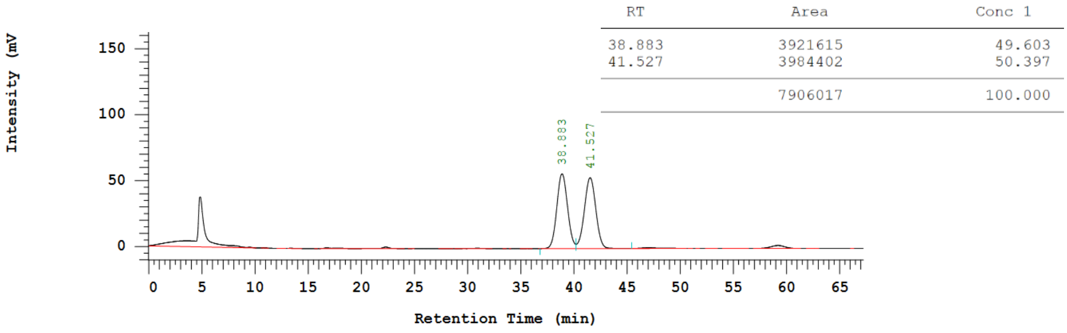

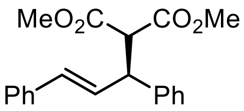

dimethyl (*S*,*E*)-2-(1,3-diphenylallyl)malonate (51)

Appearance: colourless oil; **e.e**: 92%

^{1}H NMR (400 MHz, $CDCl_3$): δ = 7.41 – 7.16 (m, 10H), 6.50 (d, *J* = 15.7 Hz, 1H), 6.40 – 6.31 (m, 1H), 4.29 – 4.22 (m, 1H), 3.98 (d, *J* = 11.0 Hz, 1H), 3.70 (s, 3H), 3.51 (s, 3H) ppm.

^{13}C NMR (101 MHz, $CDCl_3$): δ = 168.12, 167.72, 140.43, 136.88, 131.67, 129.32, 128.69, 128.51, 127.89, 127.59, 127.12, 126.30, 57.44, 52.50, 52.32, 49.17 ppm.

HPLC condition: Daicel Chiralpak IA, i-PrOH/n-hexane = 10/90, 0.8 mL/min, 254 nm UV detector; t_1= 12.5 min (minor), t_2 = 15.4 min (major).

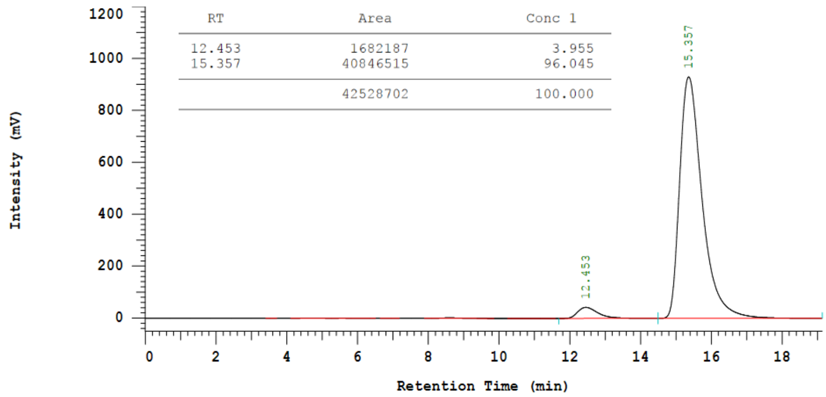

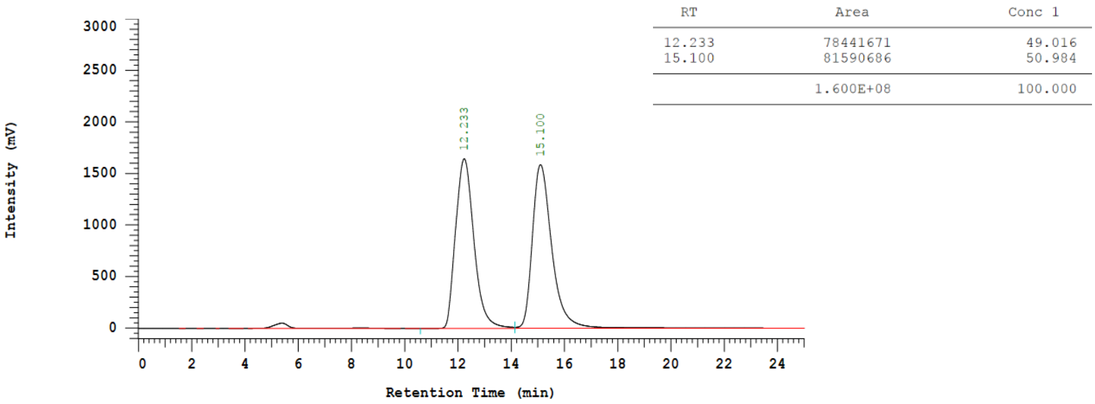

X. X-Ray crystallographic data

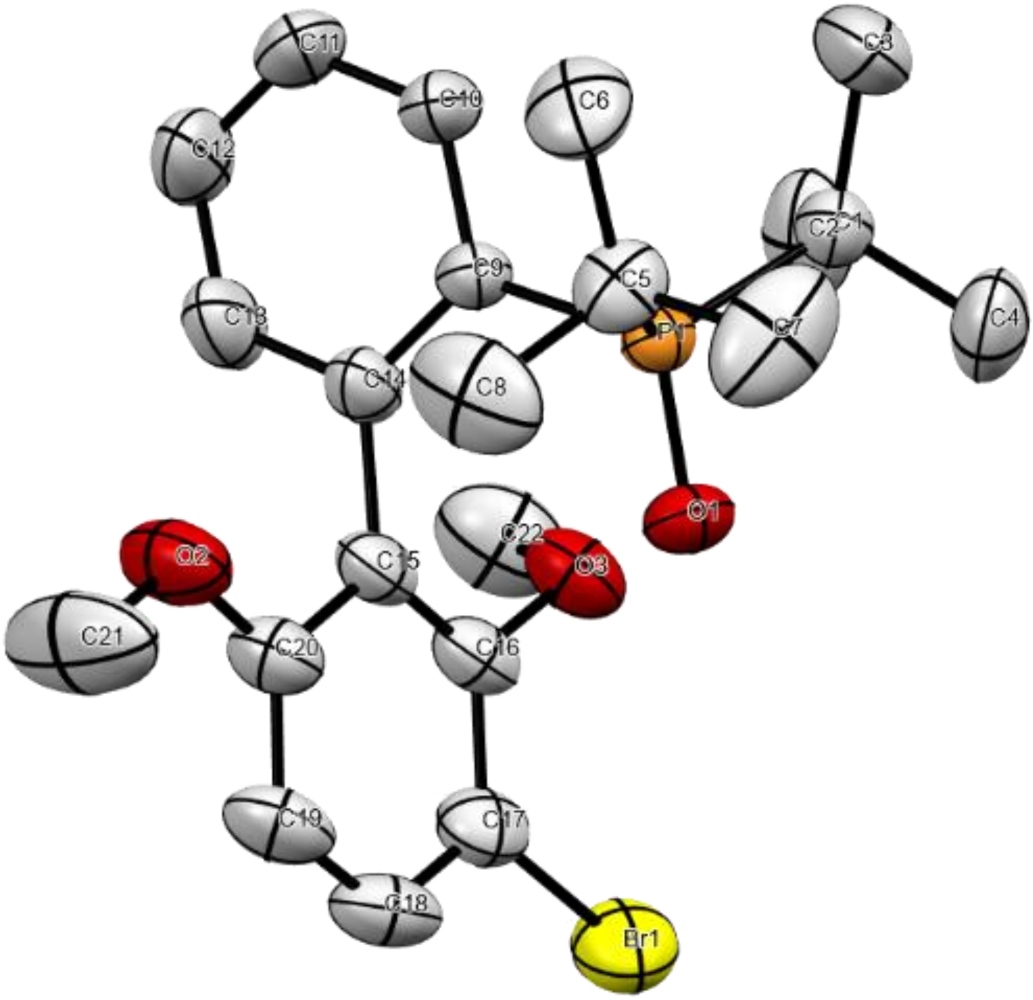

Figure S18. ORTEP diagram of **3**. Thermal ellipsoids are shown at the 50% probability level. CCDC 2442058 contains the supplementary crystallographic data for this paper. These data can be obtained free of charge from The Cambridge Crystallographic Data Centre via www.ccdc.cam.ac.uk/data request/cif.

Crystal data and structure refinement for **3**.

Identification code	yzqa209	
Empirical formula	C22 H30 Br O3 P	
Formula weight	453.34	
Temperature	248(2) K	
Wavelength	0.71073 Å	
Crystal system	Orthorhombic	
Space group	$P2_12_12_1$	
Unit cell dimensions	a = 11.4992(3) Å	α= 90°.
	b = 13.1927(3) Å	β= 90°.
	c = 15.1215(4) Å	γ = 90°.
Volume	2294.01(10) $Å^3$	
Z	4	
Density (calculated)	1.313 Mg/m^3	
Absorption coefficient	1.880 mm^{-1}	
F(000)	944	
Crystal size	0.500 x 0.400 x 0.300 mm^3	
Theta range for data collection	2.049 to 28.296°.	
Index ranges	-15<=h<=15, -17<=k<=14, -20<=l<=20	
Reflections collected	38545	
Independent reflections	5617 [R(int) = 0.0427]	
Completeness to theta = 25.242°	98.5 %	
Absorption correction	multi-scan	
Max. and min. transmission	0.7457 and 0.4490	
Refinement method	Full-matrix least-squares on F^2	
Data / restraints / parameters	5617 / 0 / 244	
Goodness-of-fit on F^2	1.037	
Final R indices [I>2sigma(I)]	R1 = 0.0354, wR2 = 0.0948	
R indices (all data)	R1 = 0.0423, wR2 = 0.0981	
Absolute structure parameter	0.068(4)	
Extinction coefficient	n/a	
Largest diff. peak and hole	0.299 and -0.296 e.$Å^{-3}$	

Table 2. Atomic coordinates (x 10^4) and equivalent isotropic displacement parameters ($Å^2$x 10^3) for **3**. U(eq) is defined as one third of the trace of the orthogonalized U^{ij} tensor.

	x	y	z	U(eq)
Br(1)	8617(1)	5293(1)	6964(1)	77(1)
P(1)	3908(1)	5462(1)	5320(1)	37(1)
O(1)	4638(2)	5675(2)	6109(1)	50(1)
O(2)	4506(3)	2516(2)	6524(2)	76(1)
O(3)	7100(2)	4936(2)	5324(2)	63(1)
C(1)	4074(3)	6521(2)	4513(2)	48(1)
C(2)	5217(4)	6348(3)	4023(3)	81(1)
C(3)	3082(5)	6679(4)	3846(3)	89(1)
C(4)	4189(5)	7506(3)	5049(3)	74(1)
C(5)	2382(3)	5248(3)	5704(2)	57(1)
C(6)	1485(3)	4968(3)	5004(3)	77(1)
C(7)	1996(4)	6214(5)	6170(4)	100(2)
C(8)	2473(4)	4378(5)	6359(4)	97(2)
C(9)	4331(3)	4299(2)	4749(2)	40(1)
C(10)	3782(3)	4065(2)	3944(2)	55(1)
C(11)	4018(4)	3178(3)	3490(2)	69(1)
C(12)	4815(5)	2523(3)	3815(3)	79(1)
C(13)	5392(4)	2743(3)	4593(2)	70(1)
C(14)	5149(3)	3623(2)	5077(2)	46(1)
C(15)	5782(3)	3731(2)	5937(2)	48(1)
C(16)	6750(3)	4347(2)	6021(2)	49(1)
C(17)	7332(3)	4415(3)	6833(2)	54(1)
C(18)	6979(3)	3837(3)	7536(2)	62(1)
C(19)	6038(4)	3180(3)	7453(2)	64(1)
C(20)	5436(3)	3136(3)	6663(2)	55(1)
C(21)	4071(6)	1962(6)	7250(3)	129(3)
C(22)	7909(5)	4452(5)	4748(3)	92(1)

Table 3. Bond lengths [Å] and angles [°] for **3**.

Br(1)-C(17)	1.887(4)
P(1)-O(1)	1.485(2)
P(1)-C(9)	1.827(3)
P(1)-C(1)	1.865(3)
P(1)-C(5)	1.870(3)
O(2)-C(20)	1.363(5)
O(2)-C(21)	1.410(5)
O(3)-C(16)	1.370(4)
O(3)-C(22)	1.426(5)
C(1)-C(2)	1.527(5)
C(1)-C(3)	1.537(6)
C(1)-C(4)	1.537(5)
C(2)-H(2A)	0.9700
C(2)-H(2B)	0.9700
C(2)-H(2C)	0.9700
C(3)-H(3A)	0.9700
C(3)-H(3B)	0.9700
C(3)-H(3C)	0.9700
C(4)-H(4A)	0.9700
C(4)-H(4B)	0.9700
C(4)-H(4C)	0.9700
C(5)-C(8)	1.519(6)
C(5)-C(7)	1.522(6)
C(5)-C(6)	1.524(5)
C(6)-H(6A)	0.9700
C(6)-H(6B)	0.9700
C(6)-H(6C)	0.9700
C(7)-H(7A)	0.9700
C(7)-H(7B)	0.9700
C(7)-H(7C)	0.9700
C(8)-H(8A)	0.9700
C(8)-H(8B)	0.9700
C(8)-H(8C)	0.9700
C(9)-C(14)	1.388(4)
C(9)-C(10)	1.405(4)
C(10)-C(11)	1.384(4)

C(10)-H(10A)	0.9400
C(11)-C(12)	1.352(6)
C(11)-H(11A)	0.9400
C(12)-C(13)	1.382(6)
C(12)-H(12A)	0.9400
C(13)-C(14)	1.402(4)
C(13)-H(13A)	0.9400
C(14)-C(15)	1.497(4)
C(15)-C(16)	1.383(5)
C(15)-C(20)	1.406(4)
C(16)-C(17)	1.401(4)
C(17)-C(18)	1.370(5)
C(18)-C(19)	1.392(6)
C(18)-H(18A)	0.9400
C(19)-C(20)	1.382(5)
C(19)-H(19A)	0.9400
C(21)-H(21A)	0.9700
C(21)-H(21B)	0.9700
C(21)-H(21C)	0.9700
C(22)-H(22A)	0.9700
C(22)-H(22B)	0.9700
C(22)-H(22C)	0.9700

O(1)-P(1)-C(9)	112.82(13)
O(1)-P(1)-C(1)	109.05(14)
C(9)-P(1)-C(1)	107.03(13)
O(1)-P(1)-C(5)	108.02(14)
C(9)-P(1)-C(5)	105.63(15)
C(1)-P(1)-C(5)	114.37(16)
C(20)-O(2)-C(21)	118.1(3)
C(16)-O(3)-C(22)	114.0(3)
C(2)-C(1)-C(3)	110.0(3)
C(2)-C(1)-C(4)	108.0(3)
C(3)-C(1)-C(4)	107.2(3)
C(2)-C(1)-P(1)	107.1(2)
C(3)-C(1)-P(1)	117.1(3)
C(4)-C(1)-P(1)	107.2(2)
C(1)-C(2)-H(2A)	109.5

C(1)-C(2)-H(2B)	109.5
H(2A)-C(2)-H(2B)	109.5
C(1)-C(2)-H(2C)	109.5
H(2A)-C(2)-H(2C)	109.5
H(2B)-C(2)-H(2C)	109.5
C(1)-C(3)-H(3A)	109.5
C(1)-C(3)-H(3B)	109.5
H(3A)-C(3)-H(3B)	109.5
C(1)-C(3)-H(3C)	109.5
H(3A)-C(3)-H(3C)	109.5
H(3B)-C(3)-H(3C)	109.5
C(1)-C(4)-H(4A)	109.5
C(1)-C(4)-H(4B)	109.5
H(4A)-C(4)-H(4B)	109.5
C(1)-C(4)-H(4C)	109.5
H(4A)-C(4)-H(4C)	109.5
H(4B)-C(4)-H(4C)	109.5
C(8)-C(5)-C(7)	110.6(4)
C(8)-C(5)-C(6)	108.4(4)
C(7)-C(5)-C(6)	109.1(4)
C(8)-C(5)-P(1)	104.6(3)
C(7)-C(5)-P(1)	106.9(3)
C(6)-C(5)-P(1)	117.1(3)
C(5)-C(6)-H(6A)	109.5
C(5)-C(6)-H(6B)	109.5
H(6A)-C(6)-H(6B)	109.5
C(5)-C(6)-H(6C)	109.5
H(6A)-C(6)-H(6C)	109.5
H(6B)-C(6)-H(6C)	109.5
C(5)-C(7)-H(7A)	109.5
C(5)-C(7)-H(7B)	109.5
H(7A)-C(7)-H(7B)	109.5
C(5)-C(7)-H(7C)	109.5
H(7A)-C(7)-H(7C)	109.5
H(7B)-C(7)-H(7C)	109.5
C(5)-C(8)-H(8A)	109.5
C(5)-C(8)-H(8B)	109.5
H(8A)-C(8)-H(8B)	109.5

C(5)-C(8)-H(8C)	109.5
H(8A)-C(8)-H(8C)	109.5
H(8B)-C(8)-H(8C)	109.5
C(14)-C(9)-C(10)	118.2(3)
C(14)-C(9)-P(1)	123.4(2)
C(10)-C(9)-P(1)	118.3(2)
C(11)-C(10)-C(9)	121.9(3)
C(11)-C(10)-H(10A)	119.0
C(9)-C(10)-H(10A)	119.0
C(12)-C(11)-C(10)	119.5(3)
C(12)-C(11)-H(11A)	120.3
C(10)-C(11)-H(11A)	120.3
C(11)-C(12)-C(13)	120.1(3)
C(11)-C(12)-H(12A)	119.9
C(13)-C(12)-H(12A)	119.9
C(12)-C(13)-C(14)	121.5(3)
C(12)-C(13)-H(13A)	119.2
C(14)-C(13)-H(13A)	119.2
C(9)-C(14)-C(13)	118.7(3)
C(9)-C(14)-C(15)	125.4(3)
C(13)-C(14)-C(15)	115.8(3)
C(16)-C(15)-C(20)	118.9(3)
C(16)-C(15)-C(14)	121.8(3)
C(20)-C(15)-C(14)	119.1(3)
O(3)-C(16)-C(15)	119.9(3)
O(3)-C(16)-C(17)	119.8(3)
C(15)-C(16)-C(17)	120.2(3)
C(18)-C(17)-C(16)	120.2(3)
C(18)-C(17)-Br(1)	119.5(3)
C(16)-C(17)-Br(1)	120.4(3)
C(17)-C(18)-C(19)	120.5(3)
C(17)-C(18)-H(18A)	119.8
C(19)-C(18)-H(18A)	119.8
C(20)-C(19)-C(18)	119.6(3)
C(20)-C(19)-H(19A)	120.2
C(18)-C(19)-H(19A)	120.2
O(2)-C(20)-C(19)	123.4(3)
O(2)-C(20)-C(15)	115.9(3)

C(19)-C(20)-C(15)	120.6(3)
O(2)-C(21)-H(21A)	109.5
O(2)-C(21)-H(21B)	109.5
H(21A)-C(21)-H(21B)	109.5
O(2)-C(21)-H(21C)	109.5
H(21A)-C(21)-H(21C)	109.5
H(21B)-C(21)-H(21C)	109.5
O(3)-C(22)-H(22A)	109.5
O(3)-C(22)-H(22B)	109.5
H(22A)-C(22)-H(22B)	109.5
O(3)-C(22)-H(22C)	109.5
H(22A)-C(22)-H(22C)	109.5
H(22B)-C(22)-H(22C)	109.5

Symmetry transformations used to generate equivalent atoms:

Table 4. Anisotropic displacement parameters ($Å^2$x 10^3) for **3**. The anisotropic displacement factor exponent takes the form: $-2\pi^2[h^2 a^{*2}U^{11} + ... + 2 h k a^* b^* U^{12}]$

	U^{11}	U^{22}	U^{33}	U^{23}	U^{13}	U^{12}
Br(1)	62(1)	80(1)	88(1)	6(1)	-20(1)	-3(1)
P(1)	38(1)	40(1)	34(1)	-4(1)	-3(1)	1(1)
O(1)	51(1)	59(1)	38(1)	-10(1)	-7(1)	-1(1)
O(2)	78(2)	93(2)	57(1)	27(1)	-12(1)	-21(2)
O(3)	63(2)	71(1)	56(1)	19(1)	0(1)	6(1)
C(1)	55(2)	43(1)	45(2)	0(1)	-3(1)	-2(1)
C(2)	99(3)	63(2)	81(3)	7(2)	37(2)	-4(2)
C(3)	121(4)	71(2)	75(3)	22(2)	-38(3)	-6(3)
C(4)	106(3)	42(2)	75(2)	-9(2)	10(2)	-4(2)
C(5)	42(2)	64(2)	64(2)	-4(2)	6(1)	-5(2)
C(6)	48(2)	81(2)	102(3)	1(2)	-12(2)	-15(2)
C(7)	56(2)	114(4)	131(4)	-49(3)	36(3)	-5(2)
C(8)	72(3)	130(4)	89(3)	41(3)	19(2)	-19(3)
C(9)	48(1)	40(1)	33(1)	-1(1)	-5(1)	-1(1)
C(10)	72(2)	50(2)	44(2)	-6(1)	-18(2)	6(2)
C(11)	106(3)	57(2)	44(2)	-13(1)	-18(2)	1(2)
C(12)	129(4)	49(2)	59(2)	-14(2)	-11(2)	20(2)
C(13)	101(3)	50(2)	59(2)	-4(2)	-12(2)	28(2)
C(14)	58(2)	44(1)	37(1)	2(1)	-2(1)	6(1)
C(15)	55(2)	49(2)	40(1)	7(1)	-5(1)	13(1)
C(16)	50(2)	55(2)	41(1)	9(1)	-1(1)	13(1)
C(17)	45(2)	61(2)	56(2)	3(1)	-9(1)	9(1)
C(18)	55(2)	90(2)	40(2)	2(2)	-9(1)	11(2)
C(19)	64(2)	88(2)	41(2)	16(2)	-3(2)	5(2)
C(20)	55(2)	65(2)	46(2)	10(1)	-6(1)	2(2)
C(21)	138(5)	173(6)	75(3)	53(4)	-21(3)	-81(5)
C(22)	86(3)	122(4)	69(2)	18(3)	20(2)	9(3)

Table 5. Hydrogen coordinates (x 10^4) and isotropic displacement parameters ($Å^2$x 10^{3}) for **3**.

	x	y	z	U(eq)
H(2A)	5839	6249	4448	121
H(2B)	5150	5751	3651	121
H(2C)	5390	6934	3658	121
H(3A)	2358	6789	4162	133
H(3B)	3250	7265	3481	133
H(3C)	3010	6083	3474	133
H(4A)	3471	7632	5367	111
H(4B)	4824	7440	5467	111
H(4C)	4344	8067	4652	111
H(6A)	1416	5517	4580	115
H(6B)	1729	4356	4701	115
H(6C)	738	4854	5285	115
H(7A)	1942	6761	5743	150
H(7B)	1241	6106	6440	150
H(7C)	2557	6391	6623	150
H(8A)	2720	3770	6052	145
H(8B)	3037	4547	6812	145
H(8C)	1720	4262	6630	145
H(10A)	3240	4524	3708	66
H(11A)	3626	3032	2959	83
H(12A)	4977	1918	3511	95
H(13A)	5962	2291	4802	84
H(18A)	7376	3884	8078	74
H(19A)	5813	2770	7931	77
H(21A)	3414	1555	7060	193
H(21B)	4676	1522	7478	193
H(21C)	3823	2427	7710	193
H(22A)	8117	4911	4273	139
H(22B)	8602	4270	5078	139
H(22C)	7560	3845	4502	139

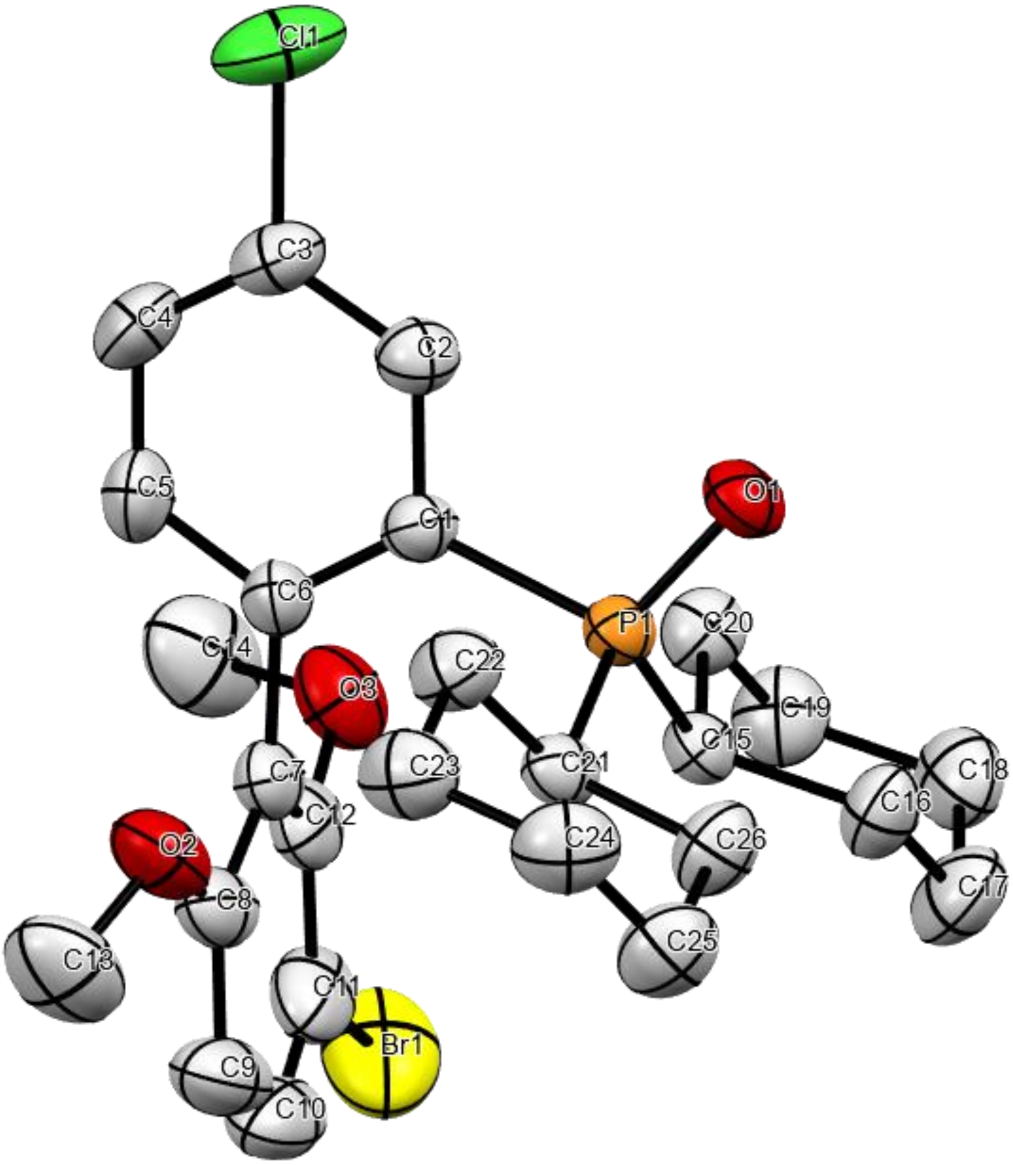

Figure S19. ORTEP diagram of **25**. Thermal ellipsoids are shown at the 50% probability level. CCDC 2442059 contains the supplementary crystallographic data for this paper. These data can be obtained free of charge from The Cambridge Crystallographic Data Centre via www.ccdc.cam.ac.uk/data request/cif.

Crystal data and structure refinement for **25**.

Identification code	yzqa303	
Empirical formula	C26 H33 Br Cl O3 P	
Formula weight	539.85	
Temperature	296(2) K	
Wavelength	0.71073 Å	
Crystal system	Orthorhombic	
Space group	$P2_12_12$	
Unit cell dimensions	a = 15.577(8) Å	α= 90°.
	b = 18.007(11) Å	β= 90°.
	c = 9.441(5) Å	γ = 90°.
Volume	2648(3) $Å^3$	
Z	4	
Density (calculated)	1.354 Mg/m^3	
Absorption coefficient	1.738 mm^{-1}	
F(000)	1120	
Crystal size	0.400 x 0.300 x 0.200 mm^3	
Theta range for data collection	2.262 to 27.794°.	
Index ranges	-19<=h<=20, -21<=k<=23, -12<=l<=12	
Reflections collected	29736	
Independent reflections	6150 [R(int) = 0.0485]	
Completeness to theta = 25.242°	99.9 %	
Absorption correction	multi-scan	
Max. and min. transmission	0.7456 and 0.5644	
Refinement method	Full-matrix least-squares on F^2	
Data / restraints / parameters	6150 / 0 / 289	
Goodness-of-fit on F^2	1.029	
Final R indices [I>2sigma(I)]	R1 = 0.0360, wR2 = 0.0821	
R indices (all data)	R1 = 0.0535, wR2 = 0.0900	
Absolute structure parameter	0.016(4)	
Extinction coefficient	n/a	
Largest diff. peak and hole	0.254 and -0.395 e.$Å^{-3}$	

Table 2. Atomic coordinates (x 10^4) and equivalent isotropic displacement parameters ($Å^2$x 10^3) for **25**. U(eq) is defined as one third of the trace of the orthogonalized U^{ij} tensor.

	x	y	z	U(eq)
Cl(1)	9245(1)	6430(1)	1804(1)	80(1)
P(1)	6943(1)	7869(1)	5096(1)	36(1)
Br(1)	6119(1)	5778(1)	10377(1)	105(1)
O(1)	7557(1)	8459(1)	4577(2)	47(1)
O(2)	5302(2)	6159(2)	4172(3)	63(1)
O(3)	7367(2)	5863(2)	7816(3)	64(1)
C(1)	7336(2)	6932(2)	4547(3)	37(1)
C(2)	8022(2)	6986(2)	3569(3)	43(1)
C(3)	8391(2)	6349(2)	3003(3)	50(1)
C(4)	8100(2)	5646(2)	3389(4)	60(1)
C(5)	7425(2)	5585(2)	4351(4)	56(1)
C(6)	7035(2)	6216(2)	4948(3)	40(1)
C(7)	6317(2)	6103(2)	6006(4)	45(1)
C(8)	5443(2)	6111(2)	5592(4)	52(1)
C(9)	4782(3)	6058(2)	6587(5)	68(1)
C(10)	4993(3)	5985(2)	8006(5)	77(1)
C(11)	5837(3)	5912(2)	8421(4)	68(1)
C(12)	6509(2)	5952(2)	7421(4)	53(1)
C(13)	4465(3)	5967(3)	3661(6)	84(2)
C(14)	7638(3)	5100(3)	7981(6)	90(2)
C(15)	6882(2)	7873(2)	7033(3)	40(1)
C(16)	6713(2)	8672(2)	7582(4)	54(1)
C(17)	6742(3)	8709(3)	9205(4)	68(1)
C(18)	7597(2)	8418(2)	9782(4)	62(1)
C(19)	7767(3)	7624(2)	9258(4)	59(1)
C(20)	7743(2)	7588(2)	7643(3)	47(1)
C(21)	5873(2)	7968(2)	4278(3)	39(1)
C(22)	5945(2)	7771(2)	2697(3)	51(1)
C(23)	5091(2)	7880(2)	1908(4)	60(1)
C(24)	4747(2)	8675(2)	2082(4)	62(1)
C(25)	4652(2)	8869(2)	3641(4)	61(1)
C(26)	5498(2)	8762(2)	4457(4)	55(1)

Table 3. Bond lengths [Å] and angles [°] for **25**.

Cl(1)-C(3)	1.752(3)
P(1)-O(1)	1.511(2)
P(1)-C(15)	1.831(3)
P(1)-C(21)	1.846(3)
P(1)-C(1)	1.867(3)
Br(1)-C(11)	1.913(4)
O(2)-C(8)	1.361(5)
O(2)-C(13)	1.432(5)
O(3)-C(12)	1.398(5)
O(3)-C(14)	1.447(5)
C(1)-C(2)	1.415(4)
C(1)-C(6)	1.424(4)
C(2)-C(3)	1.389(5)
C(2)-H(2A)	0.9300
C(3)-C(4)	1.394(6)
C(4)-C(5)	1.393(5)
C(4)-H(4A)	0.9300
C(5)-C(6)	1.406(4)
C(5)-H(5A)	0.9300
C(6)-C(7)	1.513(4)
C(7)-C(12)	1.396(5)
C(7)-C(8)	1.416(5)
C(8)-C(9)	1.398(5)
C(9)-C(10)	1.386(7)
C(9)-H(9A)	0.9300
C(10)-C(11)	1.378(7)
C(10)-H(10A)	0.9300
C(11)-C(12)	1.411(5)
C(13)-H(13A)	0.9600
C(13)-H(13B)	0.9600
C(13)-H(13C)	0.9600
C(14)-H(14A)	0.9600
C(14)-H(14B)	0.9600
C(14)-H(14C)	0.9600
C(15)-C(20)	1.548(4)
C(15)-C(16)	1.551(5)

C(15)-H(15A)	0.9800
C(16)-C(17)	1.534(5)
C(16)-H(16A)	0.9700
C(16)-H(16B)	0.9700
C(17)-C(18)	1.531(5)
C(17)-H(17A)	0.9700
C(17)-H(17B)	0.9700
C(18)-C(19)	1.536(5)
C(18)-H(18A)	0.9700
C(18)-H(18B)	0.9700
C(19)-C(20)	1.526(5)
C(19)-H(19A)	0.9700
C(19)-H(19B)	0.9700
C(20)-H(20A)	0.9700
C(20)-H(20B)	0.9700
C(21)-C(22)	1.539(4)
C(21)-C(26)	1.552(5)
C(21)-H(21A)	0.9800
C(22)-C(23)	1.538(5)
C(22)-H(22A)	0.9700
C(22)-H(22B)	0.9700
C(23)-C(24)	1.537(6)
C(23)-H(23A)	0.9700
C(23)-H(23B)	0.9700
C(24)-C(25)	1.520(6)
C(24)-H(24A)	0.9700
C(24)-H(24B)	0.9700
C(25)-C(26)	1.539(5)
C(25)-H(25A)	0.9700
C(25)-H(25B)	0.9700
C(26)-H(26A)	0.9700
C(26)-H(26B)	0.9700
O(1)-P(1)-C(15)	110.68(14)
O(1)-P(1)-C(21)	111.58(13)
C(15)-P(1)-C(21)	111.72(13)
O(1)-P(1)-C(1)	109.75(13)
C(15)-P(1)-C(1)	107.35(14)

C(21)-P(1)-C(1)	105.54(13)
C(8)-O(2)-C(13)	117.6(3)
C(12)-O(3)-C(14)	114.7(3)
C(2)-C(1)-C(6)	118.9(3)
C(2)-C(1)-P(1)	111.5(2)
C(6)-C(1)-P(1)	129.5(2)
C(3)-C(2)-C(1)	120.5(3)
C(3)-C(2)-H(2A)	119.8
C(1)-C(2)-H(2A)	119.8
C(2)-C(3)-C(4)	121.0(3)
C(2)-C(3)-Cl(1)	119.6(3)
C(4)-C(3)-Cl(1)	119.4(3)
C(5)-C(4)-C(3)	119.1(3)
C(5)-C(4)-H(4A)	120.5
C(3)-C(4)-H(4A)	120.5
C(4)-C(5)-C(6)	121.7(3)
C(4)-C(5)-H(5A)	119.2
C(6)-C(5)-H(5A)	119.2
C(5)-C(6)-C(1)	118.8(3)
C(5)-C(6)-C(7)	118.4(3)
C(1)-C(6)-C(7)	122.7(3)
C(12)-C(7)-C(8)	118.2(3)
C(12)-C(7)-C(6)	120.0(3)
C(8)-C(7)-C(6)	121.8(3)
O(2)-C(8)-C(9)	123.2(4)
O(2)-C(8)-C(7)	115.3(3)
C(9)-C(8)-C(7)	121.5(4)
C(10)-C(9)-C(8)	118.8(4)
C(10)-C(9)-H(9A)	120.6
C(8)-C(9)-H(9A)	120.6
C(11)-C(10)-C(9)	120.7(4)
C(11)-C(10)-H(10A)	119.7
C(9)-C(10)-H(10A)	119.7
C(10)-C(11)-C(12)	120.8(4)
C(10)-C(11)-Br(1)	120.4(3)
C(12)-C(11)-Br(1)	118.8(4)
C(7)-C(12)-O(3)	118.8(3)
C(7)-C(12)-C(11)	119.4(4)

O(3)-C(12)-C(11)	121.7(4)
O(2)-C(13)-H(13A)	109.5
O(2)-C(13)-H(13B)	109.5
H(13A)-C(13)-H(13B)	109.5
O(2)-C(13)-H(13C)	109.5
H(13A)-C(13)-H(13C)	109.5
H(13B)-C(13)-H(13C)	109.5
O(3)-C(14)-H(14A)	109.5
O(3)-C(14)-H(14B)	109.5
H(14A)-C(14)-H(14B)	109.5
O(3)-C(14)-H(14C)	109.5
H(14A)-C(14)-H(14C)	109.5
H(14B)-C(14)-H(14C)	109.5
C(20)-C(15)-C(16)	109.3(3)
C(20)-C(15)-P(1)	109.0(2)
C(16)-C(15)-P(1)	110.3(2)
C(20)-C(15)-H(15A)	109.4
C(16)-C(15)-H(15A)	109.4
P(1)-C(15)-H(15A)	109.4
C(17)-C(16)-C(15)	111.6(3)
C(17)-C(16)-H(16A)	109.3
C(15)-C(16)-H(16A)	109.3
C(17)-C(16)-H(16B)	109.3
C(15)-C(16)-H(16B)	109.3
H(16A)-C(16)-H(16B)	108.0
C(18)-C(17)-C(16)	111.5(3)
C(18)-C(17)-H(17A)	109.3
C(16)-C(17)-H(17A)	109.3
C(18)-C(17)-H(17B)	109.3
C(16)-C(17)-H(17B)	109.3
H(17A)-C(17)-H(17B)	108.0
C(17)-C(18)-C(19)	110.7(3)
C(17)-C(18)-H(18A)	109.5
C(19)-C(18)-H(18A)	109.5
C(17)-C(18)-H(18B)	109.5
C(19)-C(18)-H(18B)	109.5
H(18A)-C(18)-H(18B)	108.1
C(20)-C(19)-C(18)	111.0(3)

C(20)-C(19)-H(19A)	109.4
C(18)-C(19)-H(19A)	109.4
C(20)-C(19)-H(19B)	109.4
C(18)-C(19)-H(19B)	109.4
H(19A)-C(19)-H(19B)	108.0
C(19)-C(20)-C(15)	112.2(3)
C(19)-C(20)-H(20A)	109.2
C(15)-C(20)-H(20A)	109.2
C(19)-C(20)-H(20B)	109.2
C(15)-C(20)-H(20B)	109.2
H(20A)-C(20)-H(20B)	107.9
C(22)-C(21)-C(26)	110.3(3)
C(22)-C(21)-P(1)	108.4(2)
C(26)-C(21)-P(1)	112.6(2)
C(22)-C(21)-H(21A)	108.5
C(26)-C(21)-H(21A)	108.5
P(1)-C(21)-H(21A)	108.5
C(23)-C(22)-C(21)	112.1(3)
C(23)-C(22)-H(22A)	109.2
C(21)-C(22)-H(22A)	109.2
C(23)-C(22)-H(22B)	109.2
C(21)-C(22)-H(22B)	109.2
H(22A)-C(22)-H(22B)	107.9
C(24)-C(23)-C(22)	111.7(3)
C(24)-C(23)-H(23A)	109.3
C(22)-C(23)-H(23A)	109.3
C(24)-C(23)-H(23B)	109.3
C(22)-C(23)-H(23B)	109.3
H(23A)-C(23)-H(23B)	107.9
C(25)-C(24)-C(23)	110.6(3)
C(25)-C(24)-H(24A)	109.5
C(23)-C(24)-H(24A)	109.5
C(25)-C(24)-H(24B)	109.5
C(23)-C(24)-H(24B)	109.5
H(24A)-C(24)-H(24B)	108.1
C(24)-C(25)-C(26)	111.8(3)
C(24)-C(25)-H(25A)	109.2
C(26)-C(25)-H(25A)	109.2

C(24)-C(25)-H(25B)	109.2
C(26)-C(25)-H(25B)	109.2
H(25A)-C(25)-H(25B)	107.9
C(25)-C(26)-C(21)	112.6(3)
C(25)-C(26)-H(26A)	109.1
C(21)-C(26)-H(26A)	109.1
C(25)-C(26)-H(26B)	109.1
C(21)-C(26)-H(26B)	109.1
H(26A)-C(26)-H(26B)	107.8

Symmetry transformations used to generate equivalent atoms:

Table 4. Anisotropic displacement parameters ($Å^2$x 10^3) for **25**. The anisotropic displacement factor exponent takes the form: $-2\pi^2[h^2 a^{*2}U^{11} + ... + 2 h k a^* b^* U^{12}]$

	U^{11}	U^{22}	U^{33}	U^{23}	U^{13}	U^{12}
Cl(1)	67(1)	110(1)	64(1)	6(1)	29(1)	26(1)
P(1)	35(1)	33(1)	41(1)	4(1)	4(1)	-1(1)
Br(1)	156(1)	98(1)	62(1)	8(1)	26(1)	-31(1)
O(1)	45(1)	39(1)	58(1)	10(1)	8(1)	-9(1)
O(2)	44(1)	65(2)	81(2)	11(1)	-1(1)	-10(1)
O(3)	71(2)	52(2)	68(2)	17(1)	-5(1)	-12(1)
C(1)	34(1)	39(2)	39(1)	-1(1)	4(1)	3(1)
C(2)	43(2)	45(2)	41(2)	3(1)	3(1)	1(1)
C(3)	43(2)	64(2)	43(2)	-6(2)	7(1)	11(2)
C(4)	59(2)	52(3)	67(2)	-16(2)	13(2)	12(2)
C(5)	62(2)	35(2)	73(2)	-9(2)	6(2)	-2(2)
C(6)	38(1)	34(2)	49(2)	-4(1)	1(1)	-2(1)
C(7)	49(2)	30(2)	58(2)	1(1)	10(2)	-3(1)
C(8)	49(2)	35(2)	72(2)	8(2)	13(2)	0(1)
C(9)	52(2)	55(3)	98(3)	12(2)	25(2)	1(2)
C(10)	80(3)	55(3)	98(3)	5(2)	44(3)	-5(2)
C(11)	95(3)	44(3)	65(2)	4(2)	27(2)	-15(2)
C(12)	68(2)	31(2)	61(2)	4(2)	11(2)	-8(2)
C(13)	50(2)	88(4)	114(4)	15(3)	-17(2)	-1(2)
C(14)	88(3)	66(3)	114(4)	34(3)	-5(3)	5(2)
C(15)	40(2)	36(2)	43(2)	0(1)	4(1)	-5(1)
C(16)	58(2)	46(2)	57(2)	-10(2)	-5(2)	12(2)
C(17)	72(2)	75(3)	58(2)	-25(2)	0(2)	12(2)
C(18)	72(2)	68(3)	47(2)	-11(2)	-4(2)	-8(2)
C(19)	69(2)	61(2)	47(2)	5(2)	-5(2)	0(2)
C(20)	54(2)	45(2)	43(2)	4(1)	0(1)	4(2)
C(21)	37(1)	35(2)	45(2)	6(1)	4(1)	1(1)
C(22)	48(2)	59(2)	46(2)	-4(2)	-1(1)	5(2)
C(23)	50(2)	78(3)	51(2)	-4(2)	-6(2)	0(2)
C(24)	55(2)	71(3)	60(2)	10(2)	-5(2)	10(2)
C(25)	57(2)	62(3)	66(2)	2(2)	-5(2)	18(2)
C(26)	60(2)	49(2)	54(2)	-3(2)	-3(2)	14(2)

Table 5. Hydrogen coordinates (x 10^4) and isotropic displacement parameters ($Å^2$x 10^3) for **25**.

	x	y	z	U(eq)
H(2A)	8227	7450	3303	52
H(4A)	8354	5222	3010	72
H(5A)	7228	5116	4604	68
H(9A)	4211	6072	6302	82
H(10A)	4560	5984	8685	93
H(13A)	4449	6023	2650	126
H(13B)	4340	5461	3905	126
H(13C)	4046	6289	4085	126
H(14A)	8232	5087	8251	134
H(14B)	7298	4865	8701	134
H(14C)	7565	4841	7101	134
H(15A)	6416	7546	7341	47
H(16A)	7141	9005	7193	64
H(16B)	6154	8837	7255	64
H(17A)	6662	9219	9507	82
H(17B)	6275	8416	9592	82
H(18A)	7583	8422	10809	75
H(18B)	8059	8741	9475	75
H(19A)	8324	7460	9591	71
H(19B)	7336	7292	9644	71
H(20A)	7831	7078	7344	56
H(20B)	8209	7884	7263	56
H(21A)	5481	7615	4730	47
H(22A)	6382	8080	2263	61
H(22B)	6125	7257	2606	61
H(23A)	5175	7776	909	71
H(23B)	4671	7531	2270	71
H(24A)	5138	9022	1635	74
H(24B)	4194	8717	1616	74
H(25A)	4211	8558	4059	74
H(25B)	4468	9382	3728	74
H(26A)	5399	8856	5455	66

H(26B)	5915	9121	4122	66

XI. Supplementary references

1. Frisch, M. J.; Trucks, G. W.; Schlegel, H. B.; Scuseria, G. E.; Robb, M. A.; Cheeseman, J. R.; Scalmani, G.; Barone, V.; Petersson, G. A.; Nakatsuji, H.; et al. *Gaussian 16 Rev. C.01*; Wallingford, CT, 2016.
2. Marenich, A. V.; Cramer, C. J.; Truhlar, D. G. Universal Solvation Model Based on Solute Electron Density and on a Continuum Model of the Solvent Defined by the Bulk Dielectric Constant and Atomic Surface Tensions. *J. Phys. Chem. B* **2009**, *113*, 6378–6396.
3. Engelage, E.; Schulz, N.; Heinen, F.; Huber, S. M.; Truhlar, D. G.; Cramer, C. J. Refined SMD Parameters for Bromine and Iodine Accurately Model Halogen-Bonding Interactions in Solution. *Chem. - Eur. J.* **2018**, *24*, 15983–15987.
4. Luchini, G.; Alegre-Requena, J.; Funes-Ardoiz, I.; Paton, R. GoodVibes: Automated Thermochemistry for Heterogeneous Computational Chemistry Data. *F1000Research* **2020**, *9*, 291.
5. Grimme, S.; Ehrlich, S.; Goerigk, L. Effect of the Damping Function in Dispersion Corrected Density Functional Theory. *J. Comput. Chem.* **2011**, *32*, 1456–1465.
6. Bickelhaupt, F. M.; Houk, K. N. Analyzing Reaction Rates with the Distortion/Interaction-Activation Strain Model. *Angew. Chem., Int. Ed.* **2017**, *56*, 10070–10086.
7. Ang, S. J.; Mak, A. M.; Sullivan, M. B.; Wong, M. W. Site Specificity of Halogen Bonding Involving Aromatic Acceptors. *Phys. Chem. Chem. Phys.* **2018**, *20*, 8685–8694.
8. Ang, S. J.; Mak, A. M.; Wong, M. W. Nature of Halogen Bonding Involving π-Systems, Nitroxide Radicals and Carbenes: A Highlight on the Importance of Charge Transfer. *Phys. Chem. Chem. Phys.* **2018**, *20*, 26463–26478.
9. Huang, J.; Wang, Z.; Chen, Y.; Zhang, X.; Zhou, L.; Sun, J. Desymmetrizing Atroposelective Bromination of N-Arylcarbazoles Enabled by Cross-Assembled Bifunctional Catalysts. *Chem* **2025**, *11*, 102439.
10. Yu, H.; Li, T.; Liao, P.; Duan, W.-L. Remote Selective C−H Halogenation of Arylphosphine Oxides with Ferric Halides. *Adv. Synth. Catal.* **2023**, *365*, 3101–3106.
11. Barder, T. E.; Walker, S. D.; Martinelli, J. R.; Buchwald, S. L. Catalysts for Suzuki–Miyaura Coupling Processes: Scope and Studies of the Effect of Ligand Structure. *J. Am. Chem. Soc.* **2005**, *127*, 4685–4696.
12. Yang, Y.; Wu, C.; Xing, J.; Dou, X. Developing Biarylhemiboronic Esters for Biaryl Atropisomer Synthesis via Dynamic Kinetic Atroposelective Suzuki–Miyaura Cross-Coupling. *J. Am. Chem. Soc.* **2024**, *146*, 6283–6293.
13. Xie, J.-H.; Wang, L.-X.; Fu, Y.; Zhu, S.-F.; Fan, B.-M.; Duan, H.-F.; Zhou, Q.-L. Synthesis of Spiro Diphosphines and Their Application in Asymmetric Hydrogenation of Ketones. *J. Am. Chem. Soc.* **2003**, *125*, 4404–4405.
14. Milne, J. E.; Buchwald, S. L. An Extremely Active Catalyst for the Negishi Cross-Coupling Reactions. *J. Am. Chem. Soc.* **2004**, *126*, 13028–13032.

15. Ikawa, T.; Barder, T. E.; Biscoe, M. R.; Buchwald, S. L. Pd-Catalyzed Amidations of Aryl Chlorides Using Monodentate Biaryl Phosphine Ligands: A Kinetic, Computational, and Synthetic Investigation. *J. Am. Chem. Soc.* **2007**, *129*43, 13001–13007.

XII. Copies of NMR spectra

(*R*)-(3'-bromo-2',6'-dimethoxy-[1,1'-biphenyl]-2-yl)dicyclohexylphosphine oxide (1); $CDCl_3$

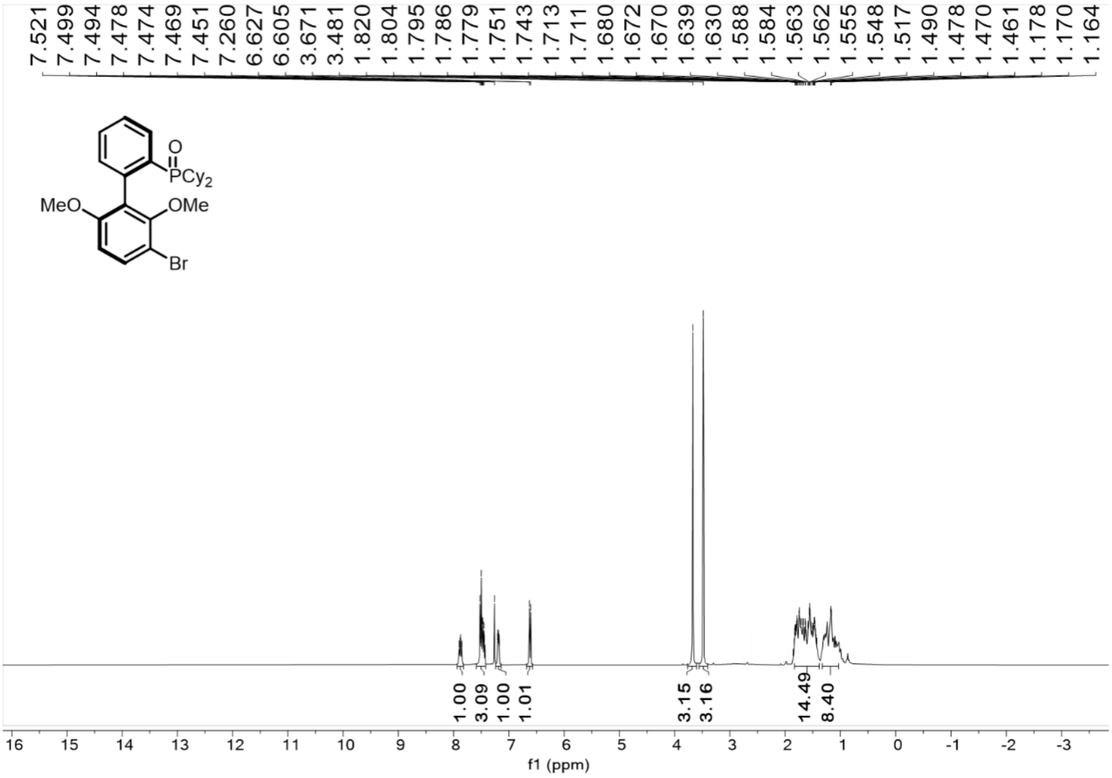

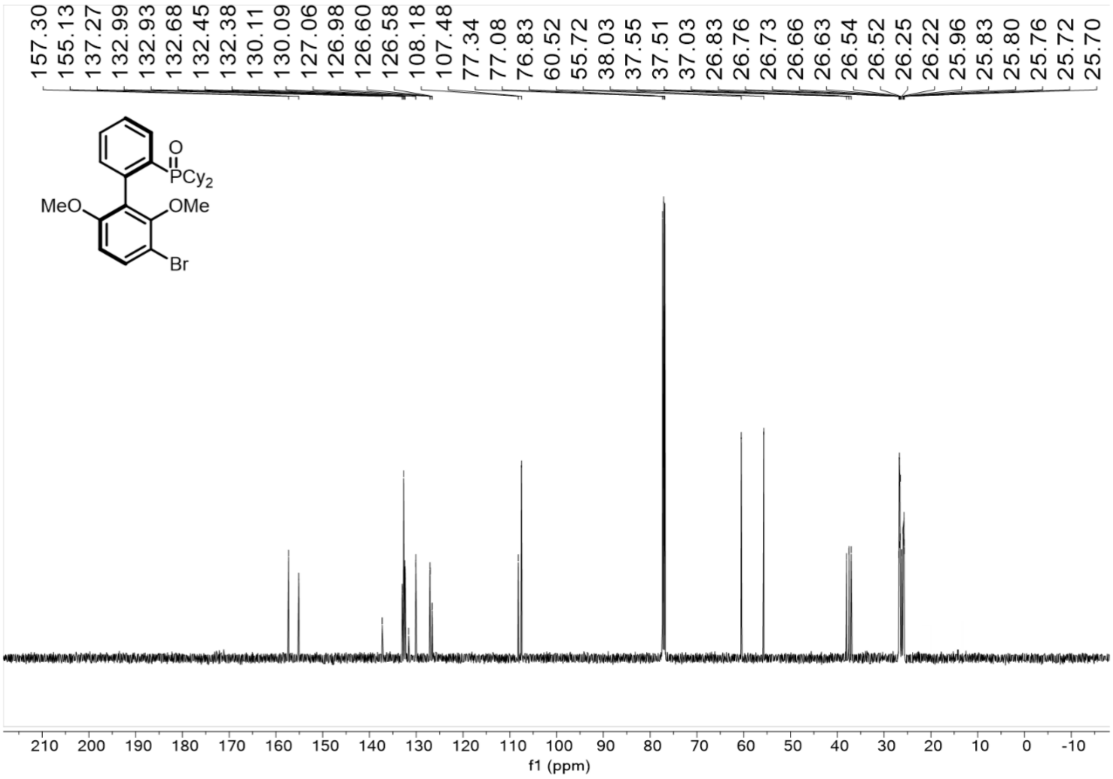

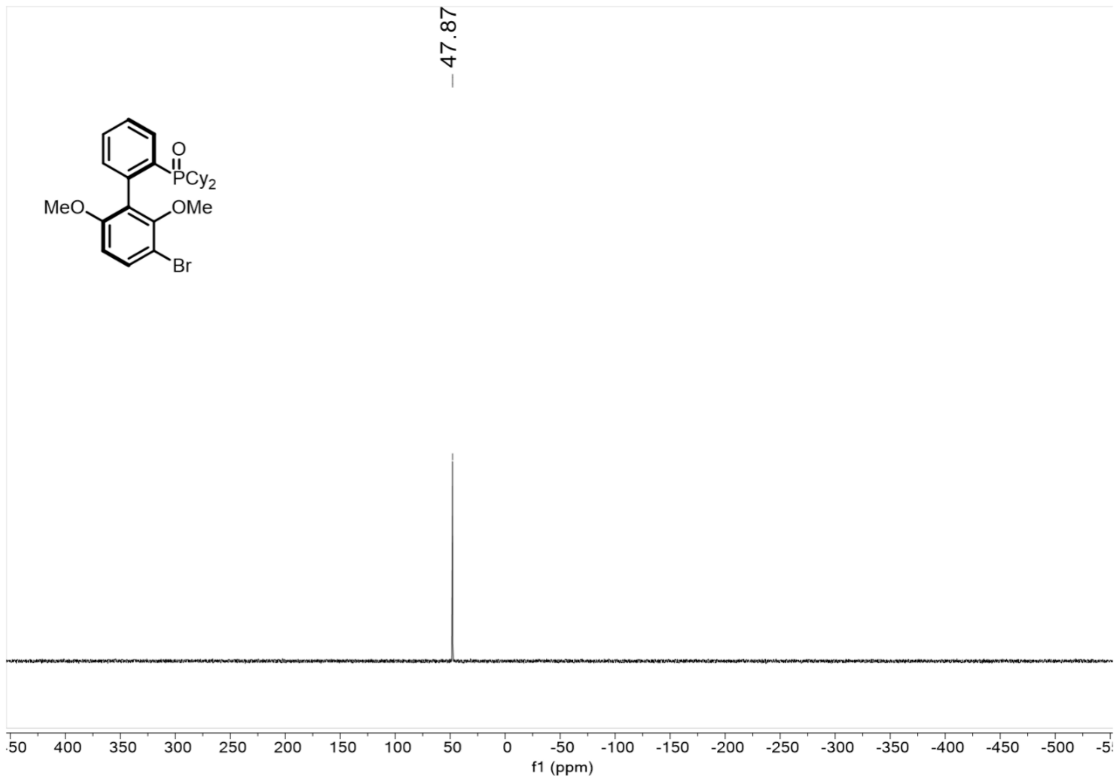

47.87
O
PCy2
MeO
OMe
Br
-50 400 350 300 250 200 150 100 50 0 -50 -100 -150 -200 -250 -300 -350 -400 -450 -500
f1 (ppm)

(*R*)-(3'-bromo-2',6'-dimethoxy-[1,1'-biphenyl]-2-yl)diisopropylphosphine oxide (2); $CDCl_3$

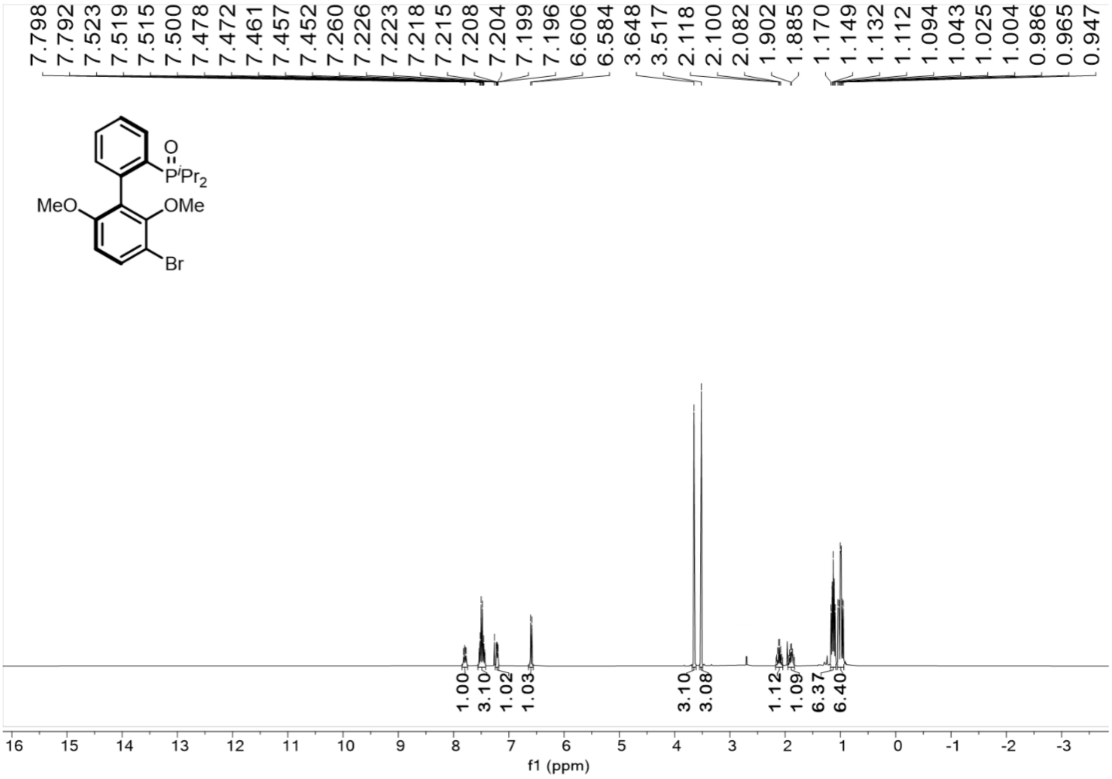

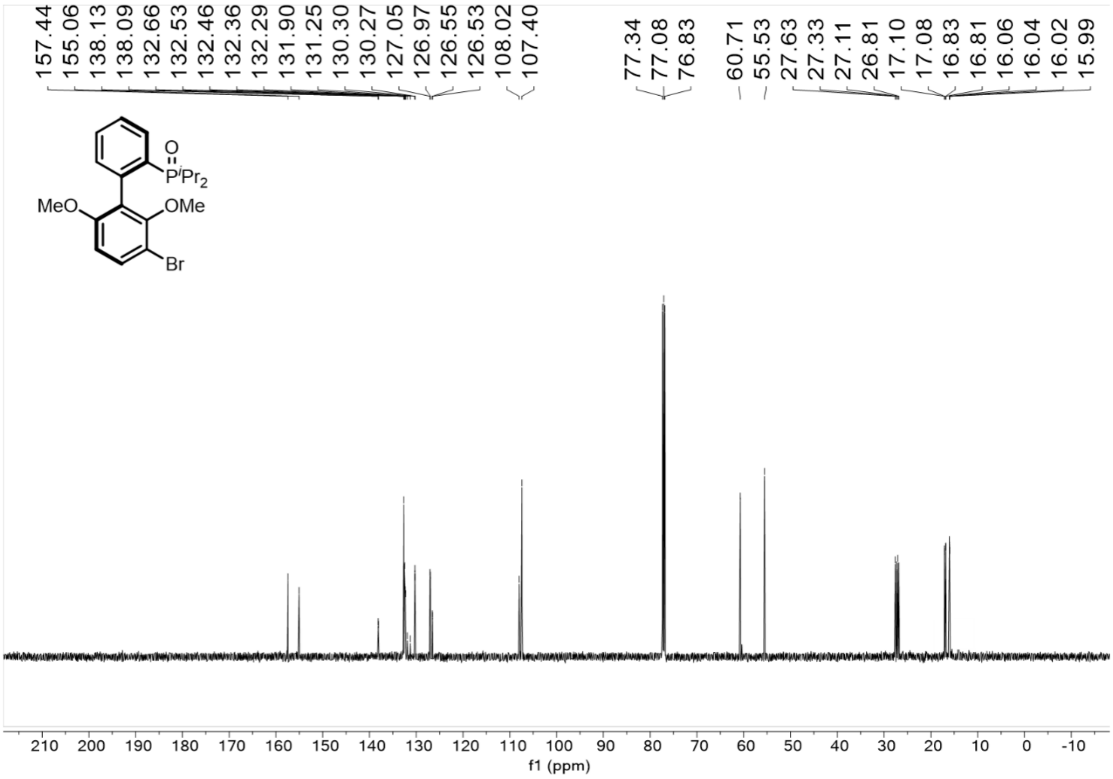

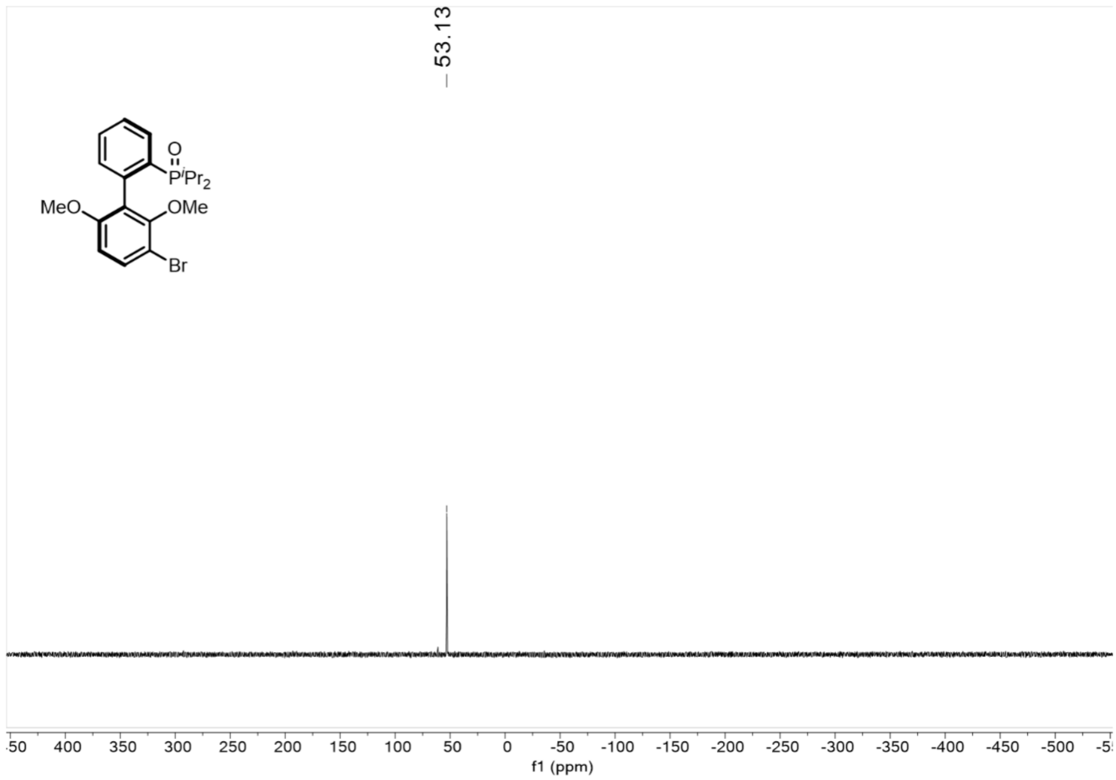
53.13
MeO
OMe
Br
O
P^{i}Pr$_2$
-50 400 350 300 250 200 150 100 50 0 -50 -100 -150 -200 -250 -300 -350 -400 -450 -500
f1 (ppm)

(*R*)-(3'-bromo-2',6'-dimethoxy-[1,1'-biphenyl]-2-yl)di-tert-butylphosphine oxide (3); $CDCl_3$

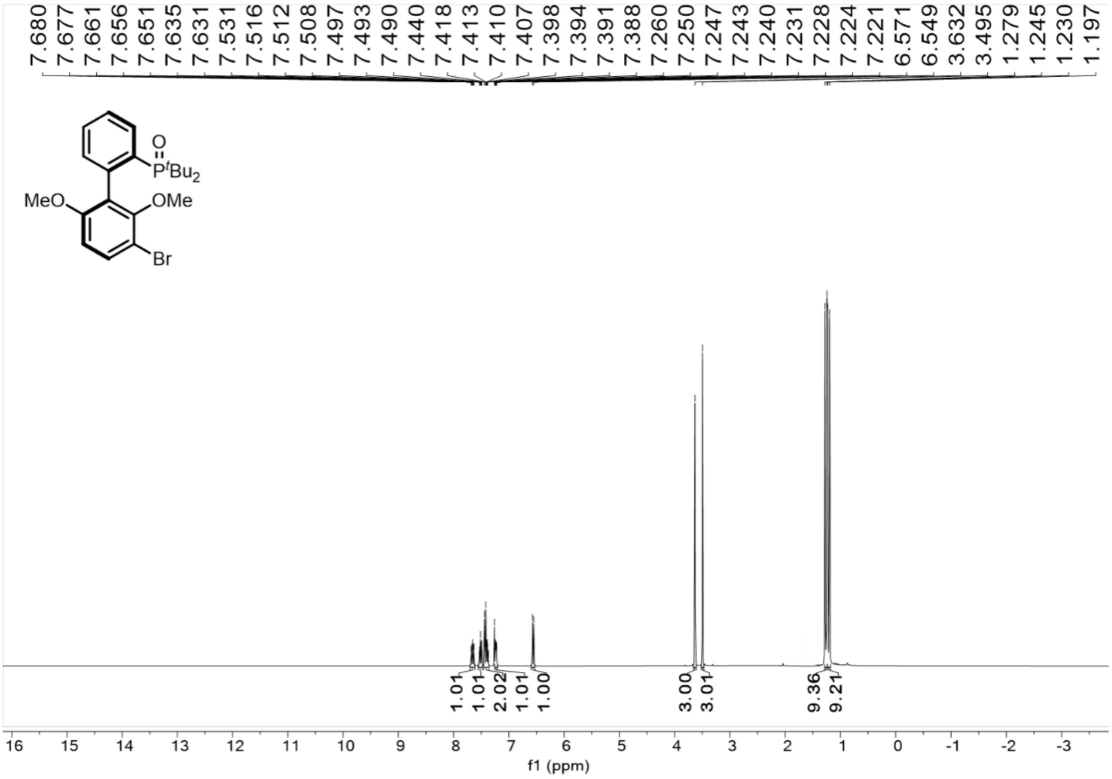

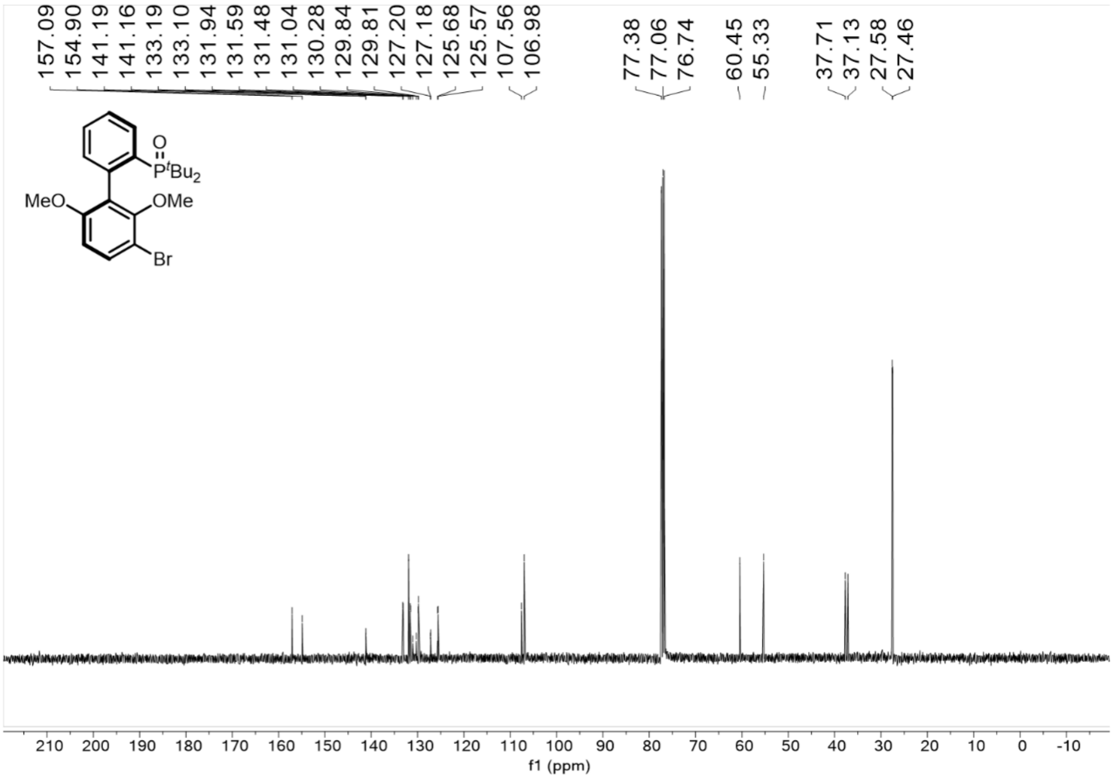

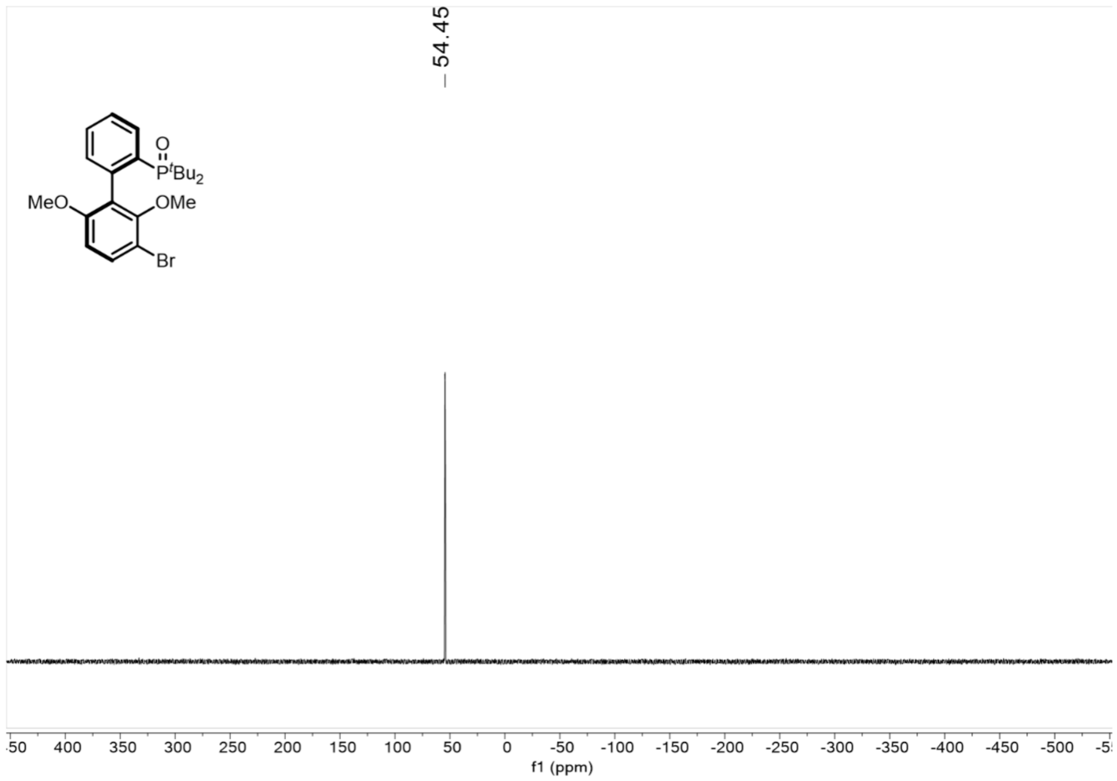
54.45
O
P^tBu2
MeO
OMe
Br
50 400 350 300 250 200 150 100 50 0 -50 -100 -150 -200 -250 -300 -350 -400 -450 -500
f1 (ppm)

(*R*)-diadamantan-1-yl-(3'-bromo-2',6'-dimethoxy-[1,1'-biphenyl]-2-yl)phosphine oxide (4); $CDCl_3$

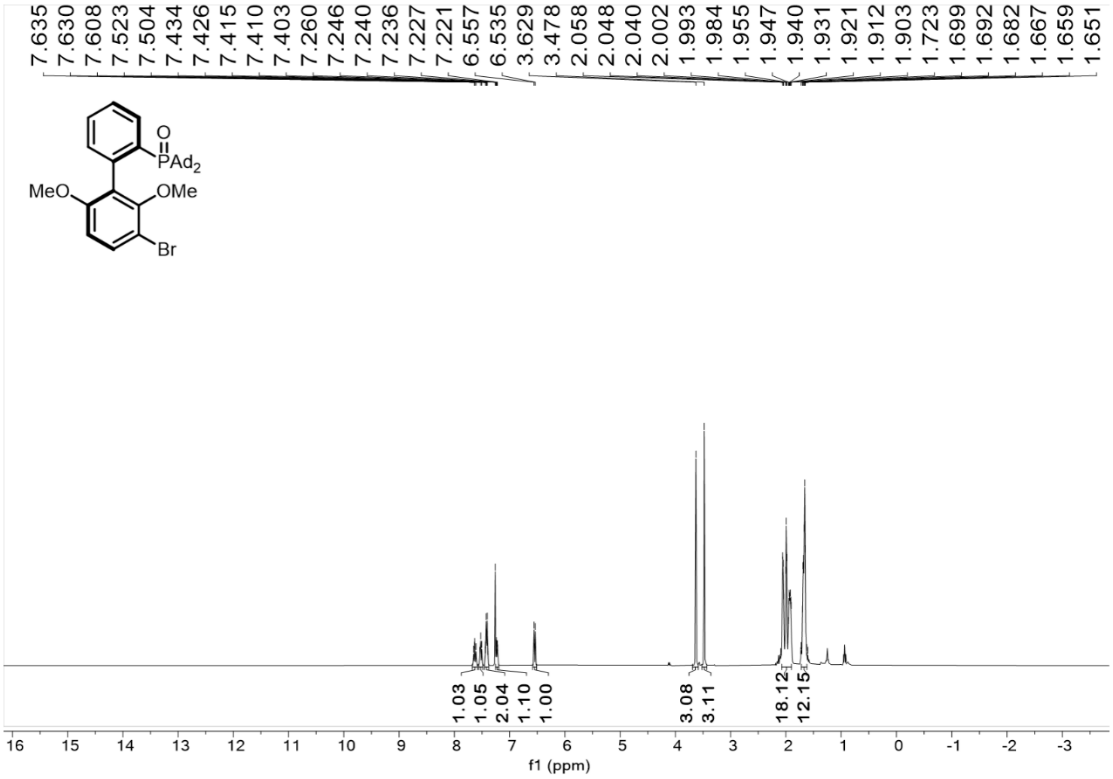

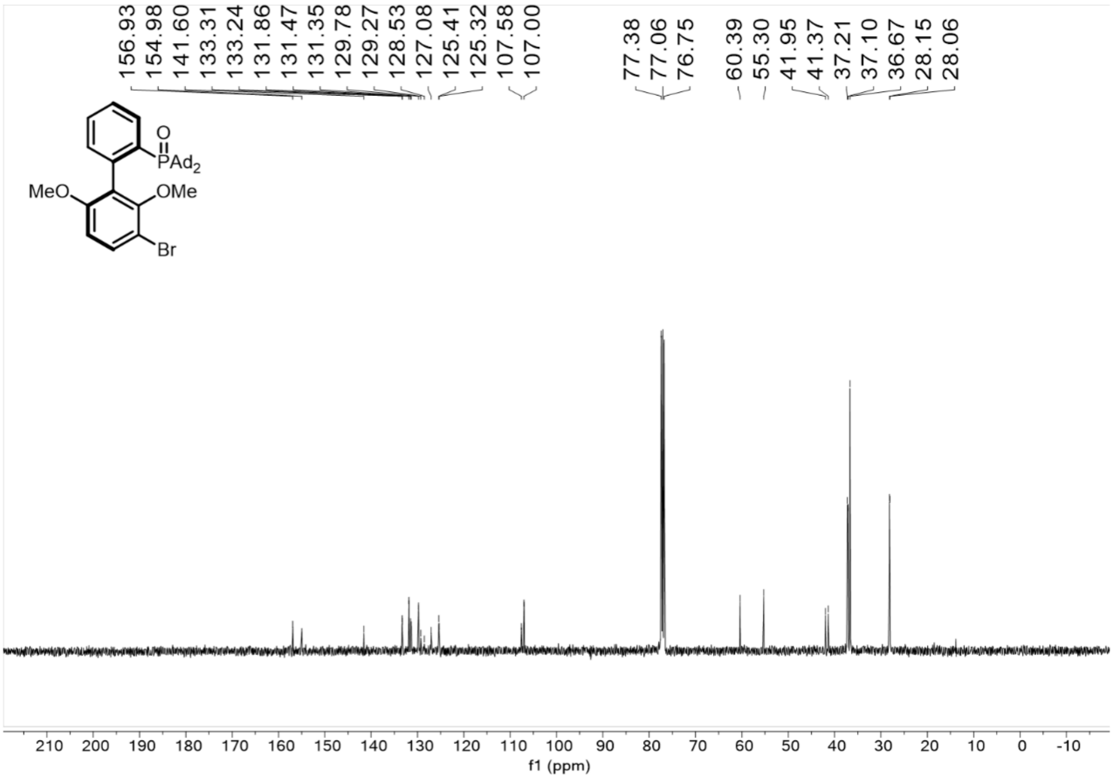

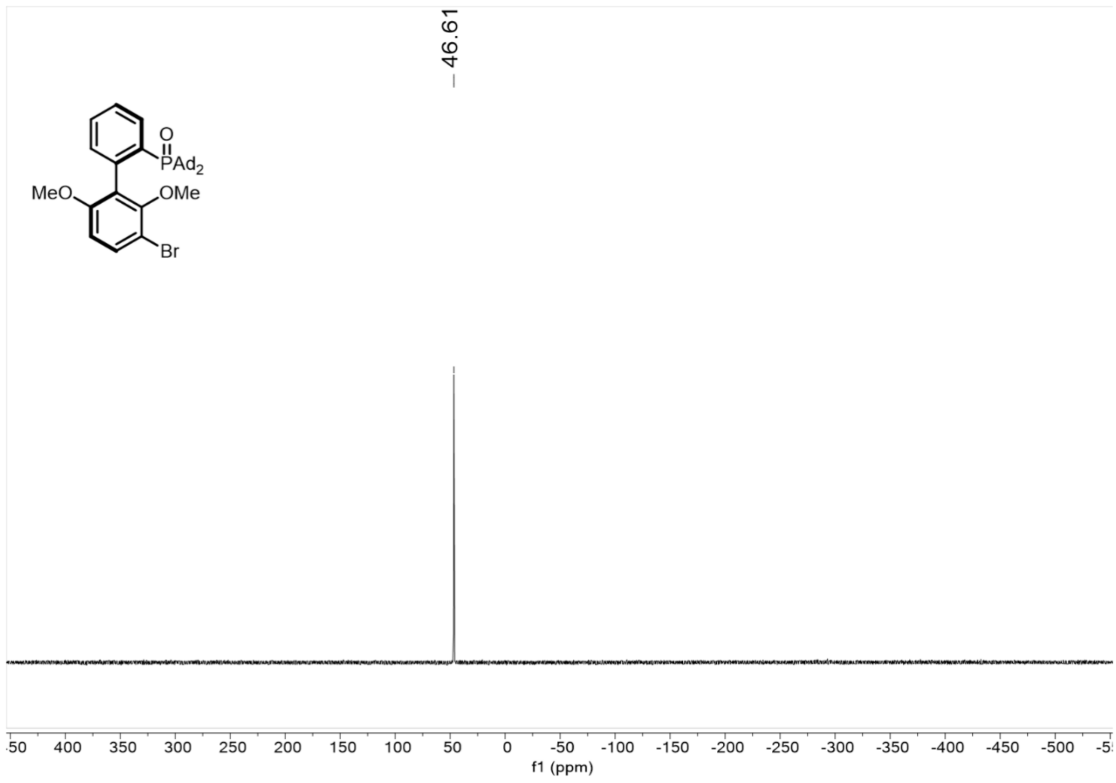

46.61
MeO
OMe
PAd2
O
Br
f1 (ppm)

(*R*)-(3'-bromo-2',6'-dimethoxy-[1,1'-biphenyl]-2-yl)dicyclopentylphosphine oxide (5); $CDCl_3$

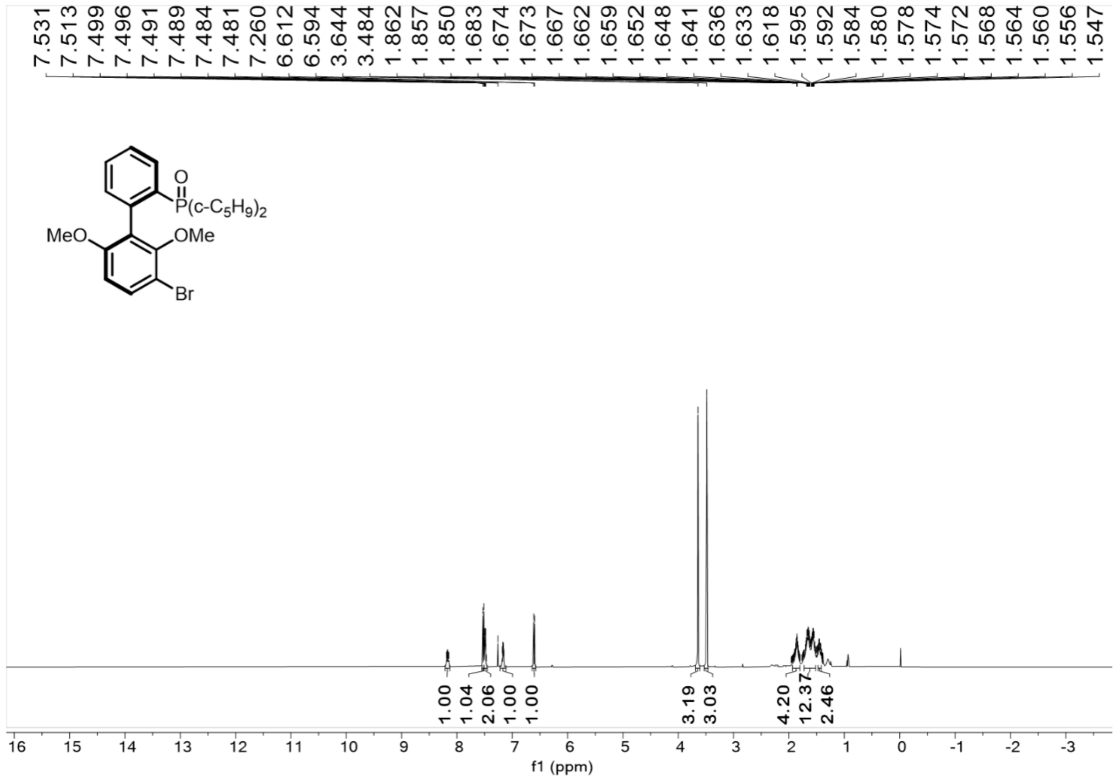

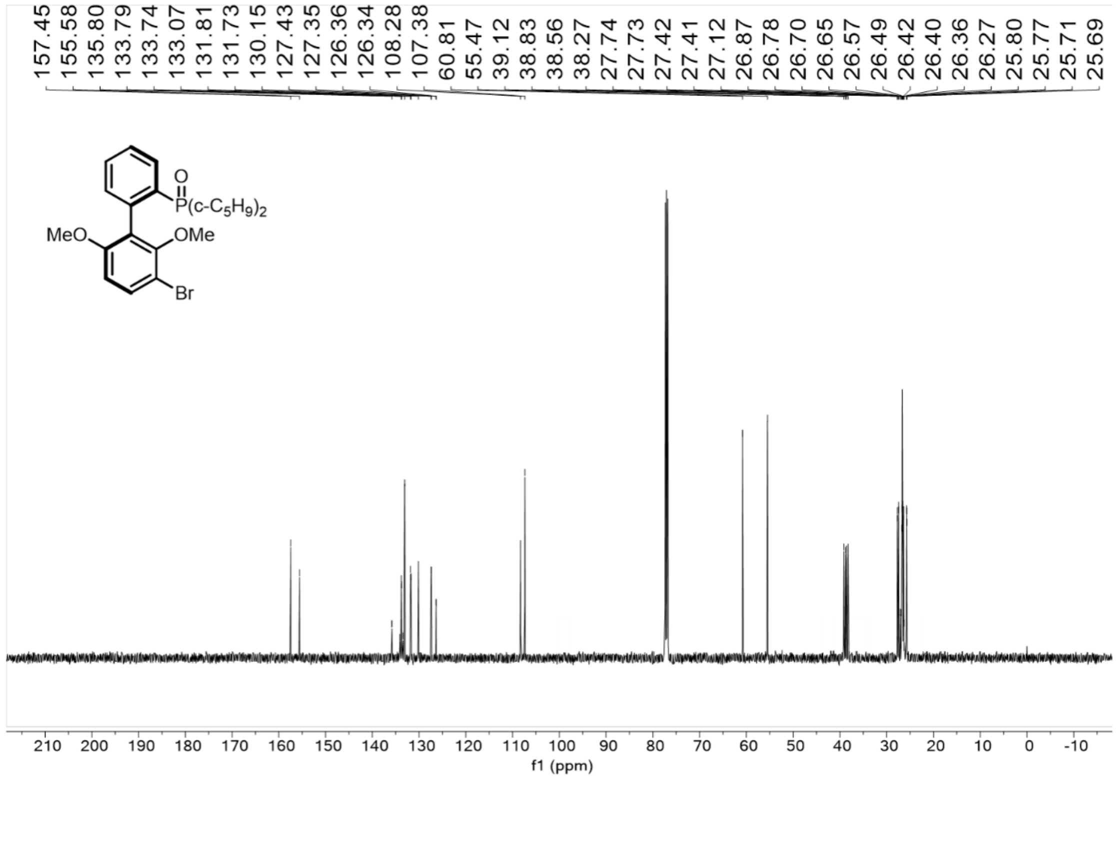

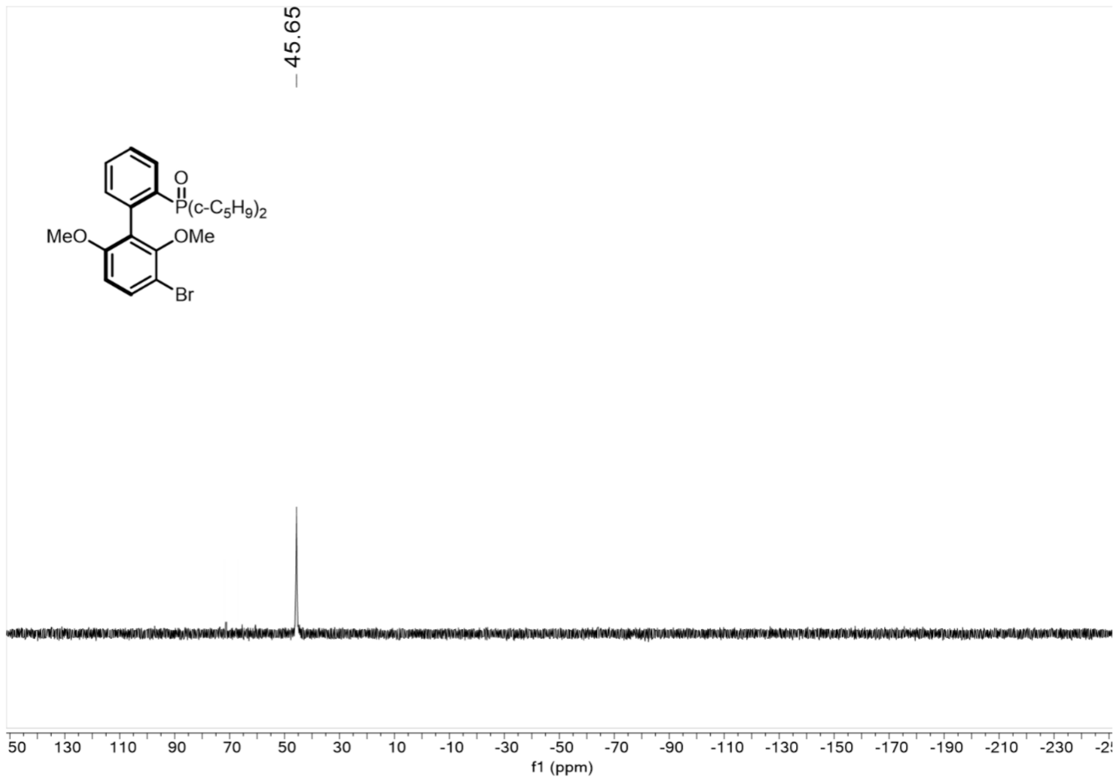

45.65
O
P(c-C5H9)2
MeO
OMe
Br
50 130 110 90 70 50 30 10 -10 -30 -50 -70 -90 -110 -130 -150 -170 -190 -210 -230
f1 (ppm)

(*R*)-(3'-bromo-2',6'-dimethoxy-6-methyl-[1,1'-biphenyl]-2-yl)dicyclohexylphosphine oxide (6); $CDCl_3$

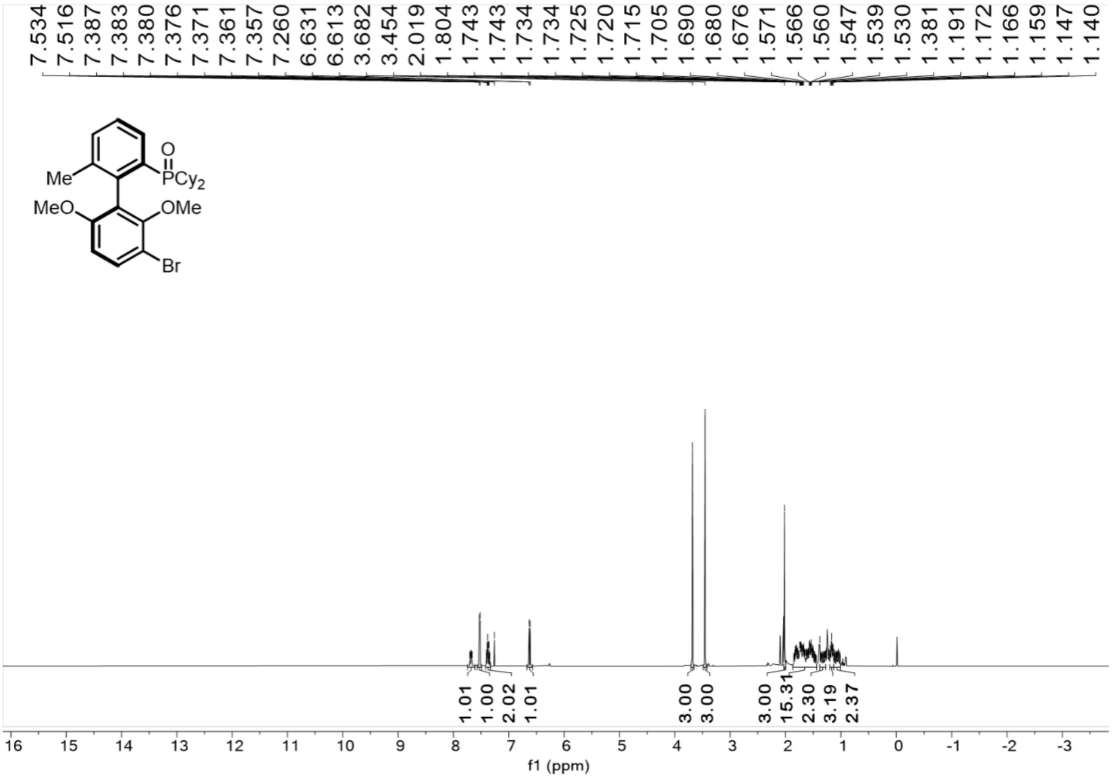

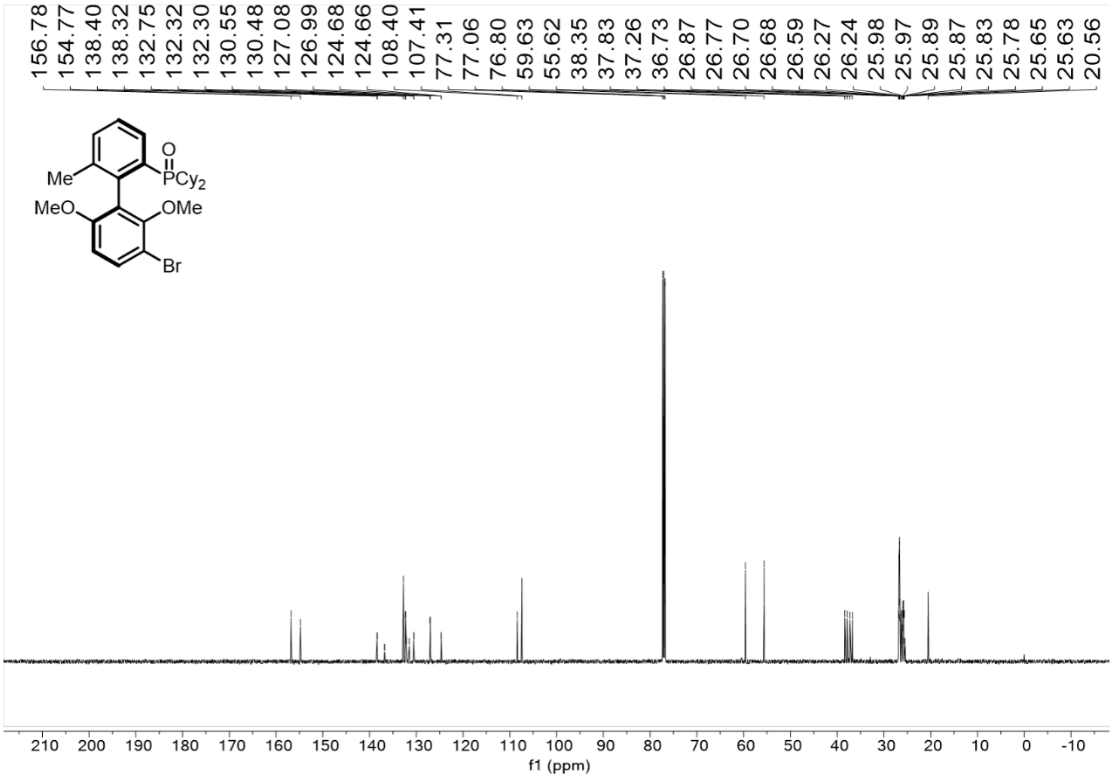

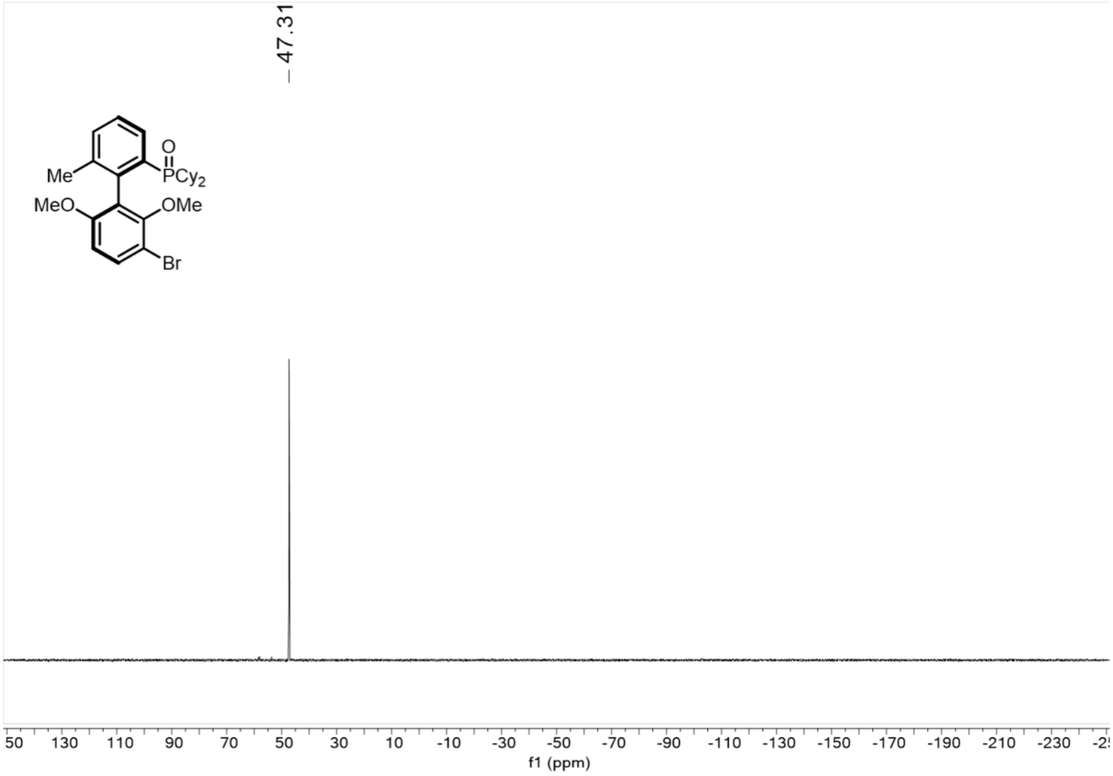
47.31
O
Me
PCy2
MeO
OMe
Br
50
130
110
90
70
50
30
10
-10
-30
-50
-70
-90
-110
-130
-150
-170
-190
-210
-230
f1 (ppm)

(*R*)-(3'-bromo-2',6,6'-trimethoxy-[1,1'-biphenyl]-2-yl)dicyclohexylphosphine oxide (7); $CDCl_3$

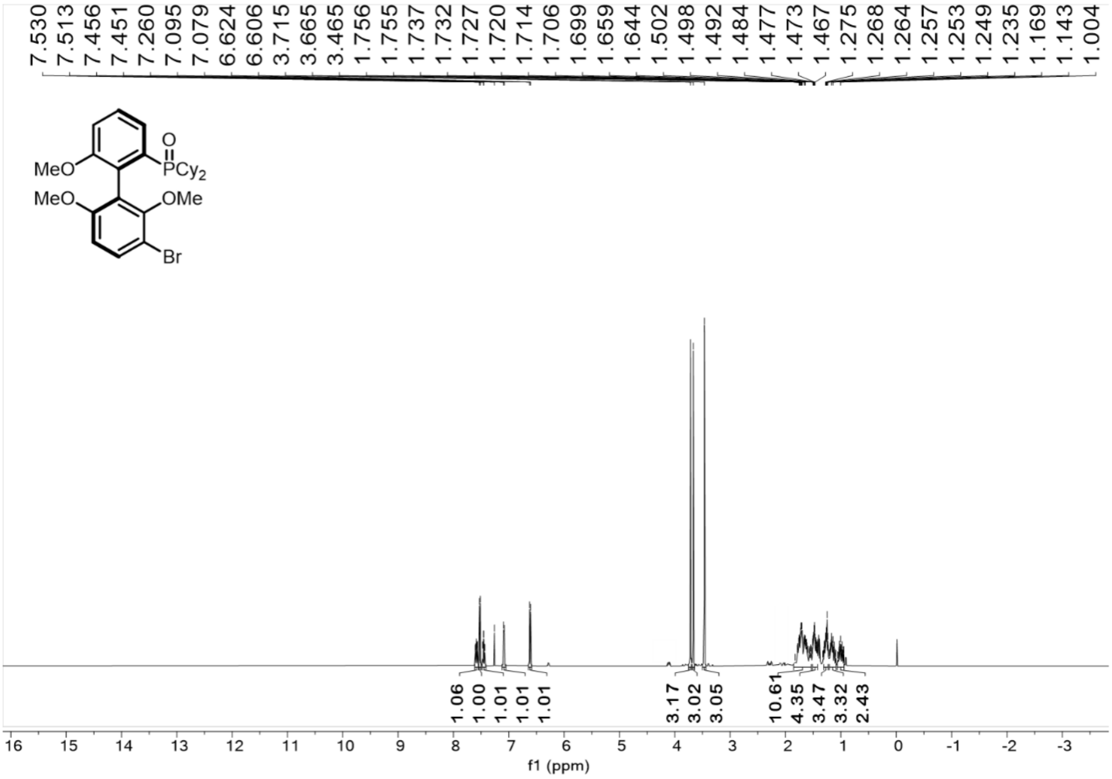

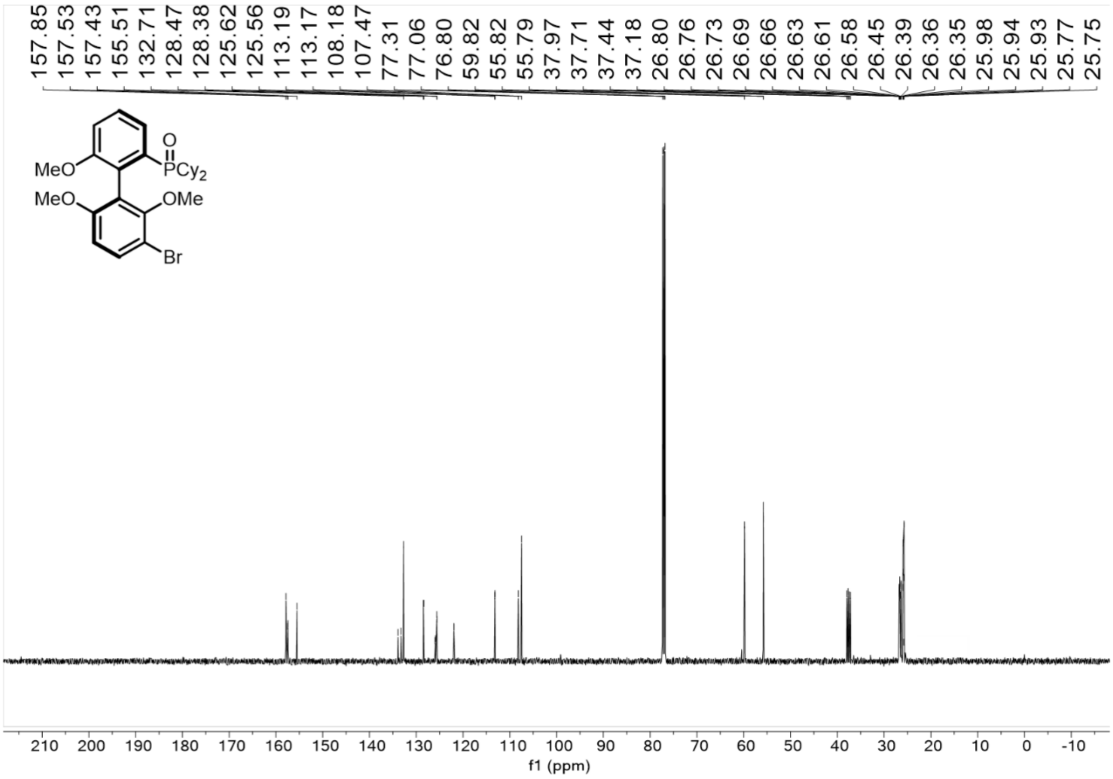

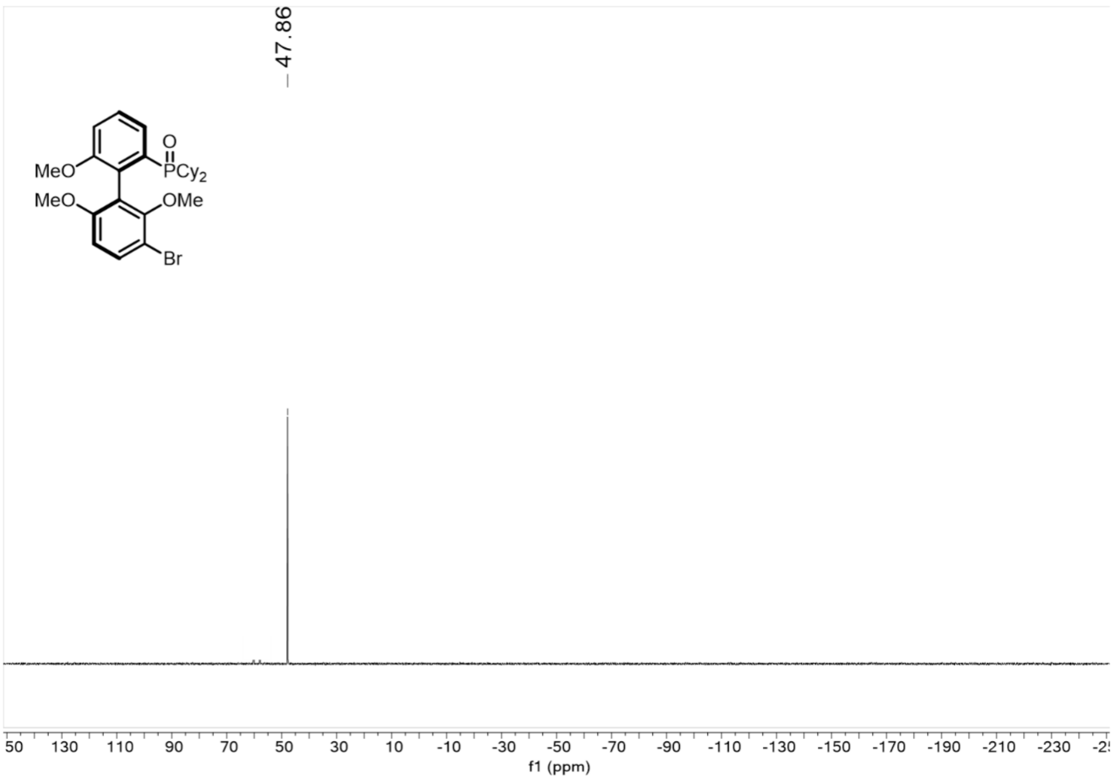

47.86
MeO
PCy2
O
MeO
OMe
Br
f1 (ppm)

(*R*)-(3'-bromo-4,6-difluoro-2',6'-dimethoxy-[1,1'-biphenyl]-2-yl)dicyclohexylphosphine oxide (8); $CDCl_3$

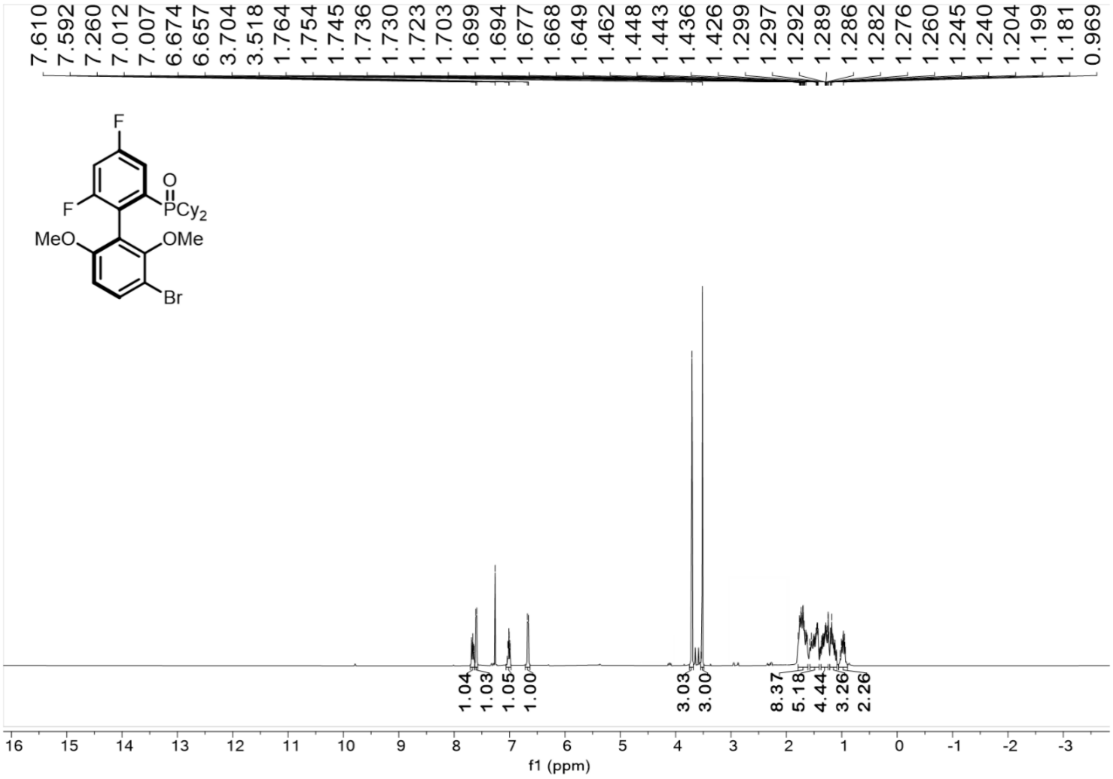

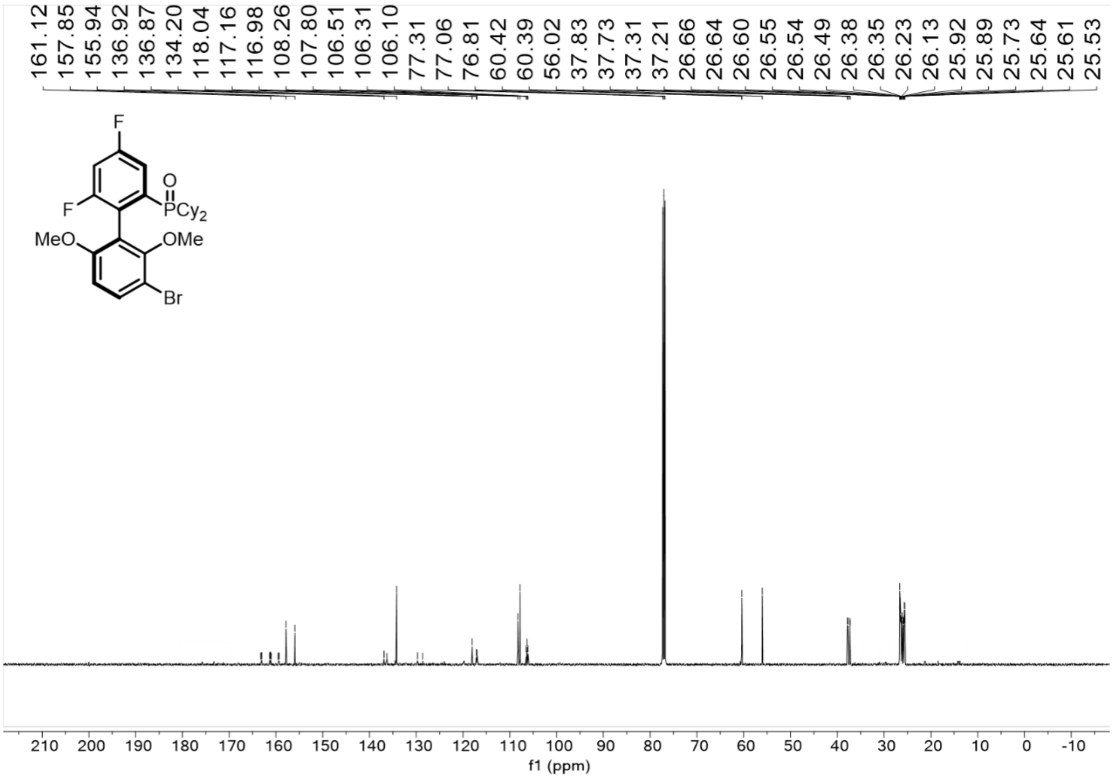

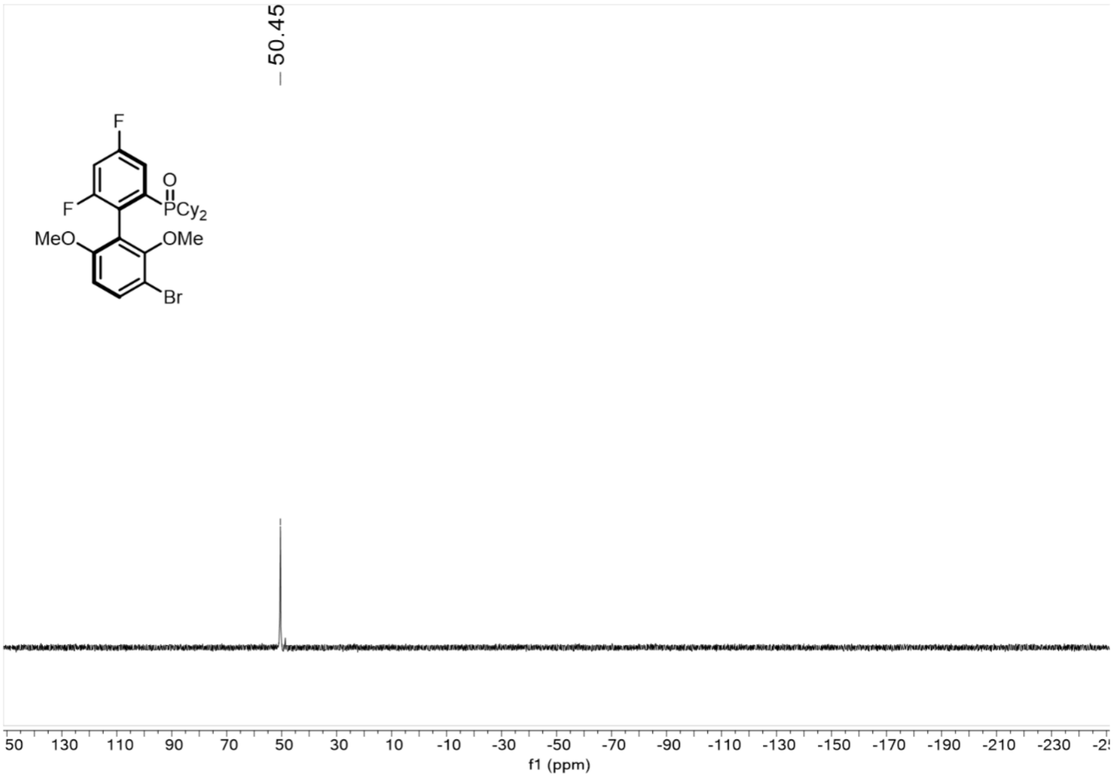
50.45
F
F
O
PCy2
MeO
OMe
Br
150 130 110 90 70 50 30 10 -10 -30 -50 -70 -90 -110 -130 -150 -170 -190 -210 -230 -2
f1 (ppm)

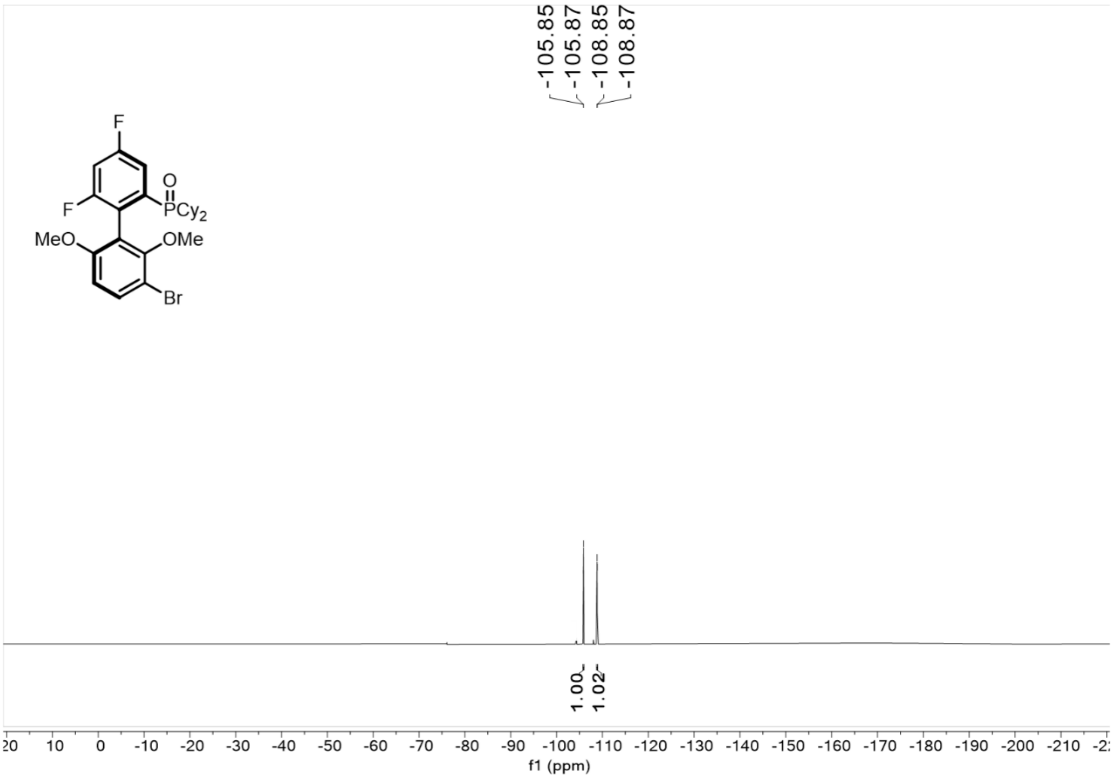
-105.85
-105.87
-108.85
-108.87
F
F
O
PCy2
MeO
OMe
Br
1.00
1.02
20 10 0 -10 -20 -30 -40 -50 -60 -70 -80 -90 -100 -110 -120 -130 -140 -150 -160 -170 -180 -190 -200 -210 -2
f1 (ppm)

(*R*)-(3''-bromo-2'',6''-dimethoxy-[1,1':3',1''-terphenyl]-4'-yl)dicyclohexylphosphine oxide (9); $CDCl_3$

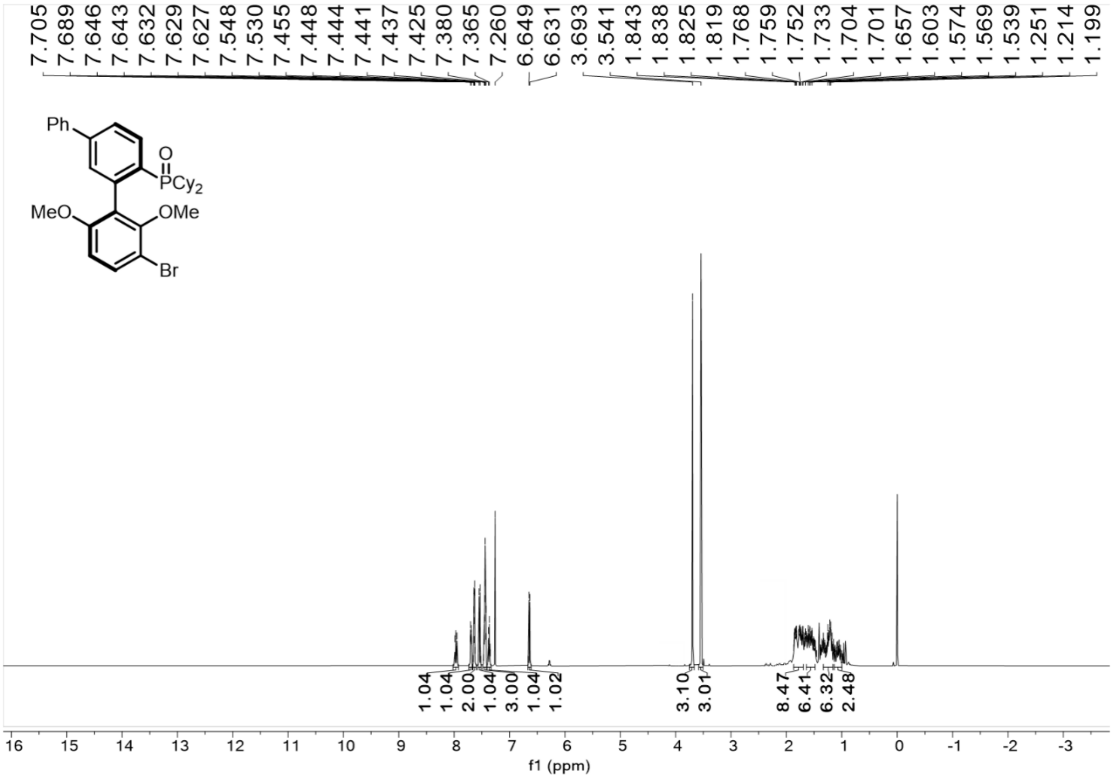

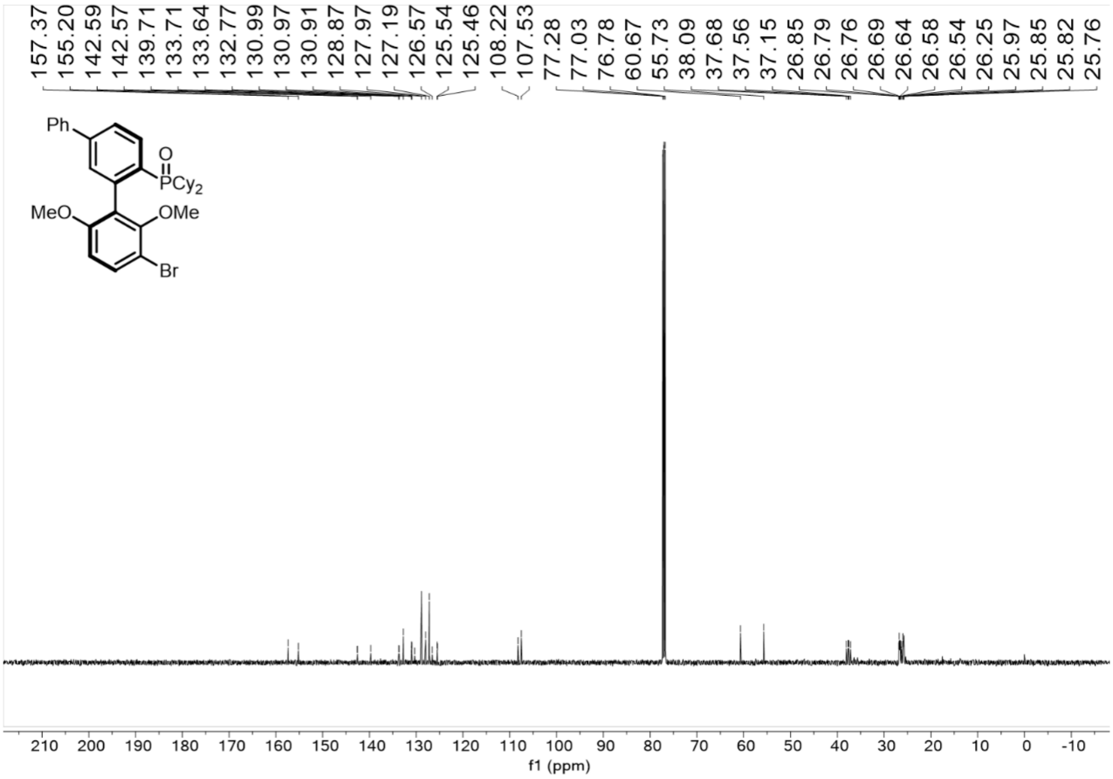

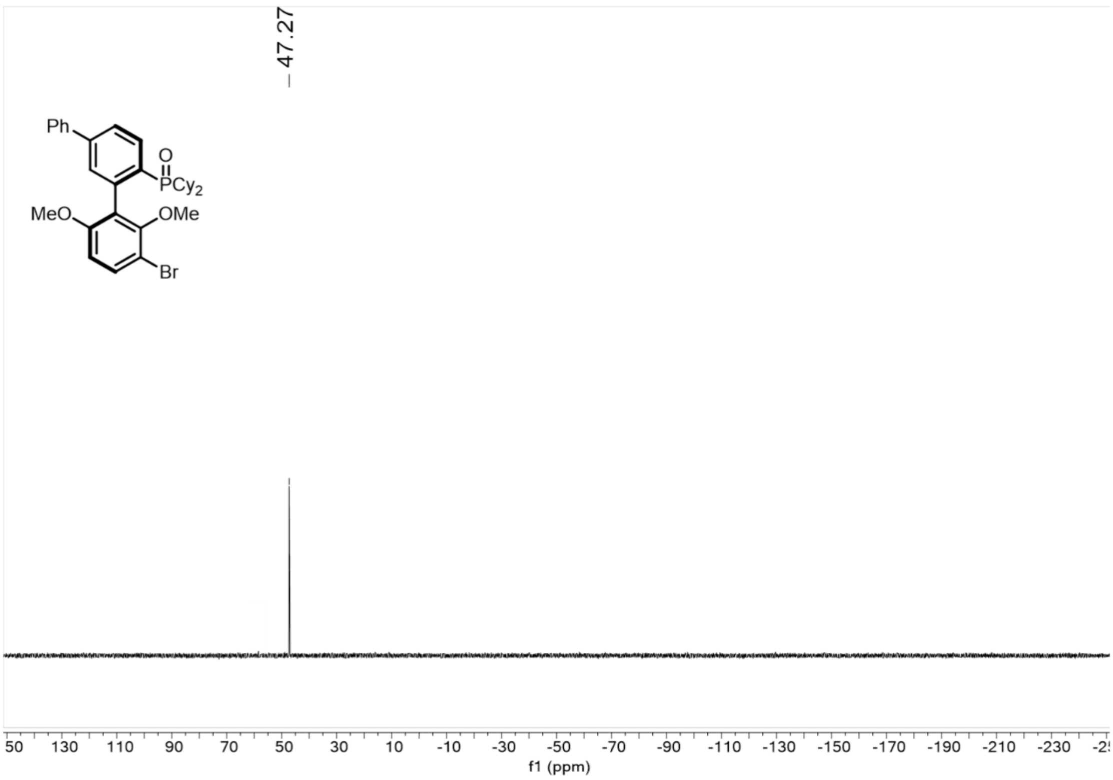

47.27
Ph
O
PCy2
MeO
OMe
Br
50
130
110
90
70
50
30
10
-10
-30
-50
-70
-90
-110
-130
-150
-170
-190
-210
-230
f1 (ppm)

((*R*)-5-(-adamantan-1-yl)-3'-bromo-2',6'-dimethoxy-[1,1'-biphenyl]-2-yl)dicyclohexylphosphine oxide (10); $CDCl_3$

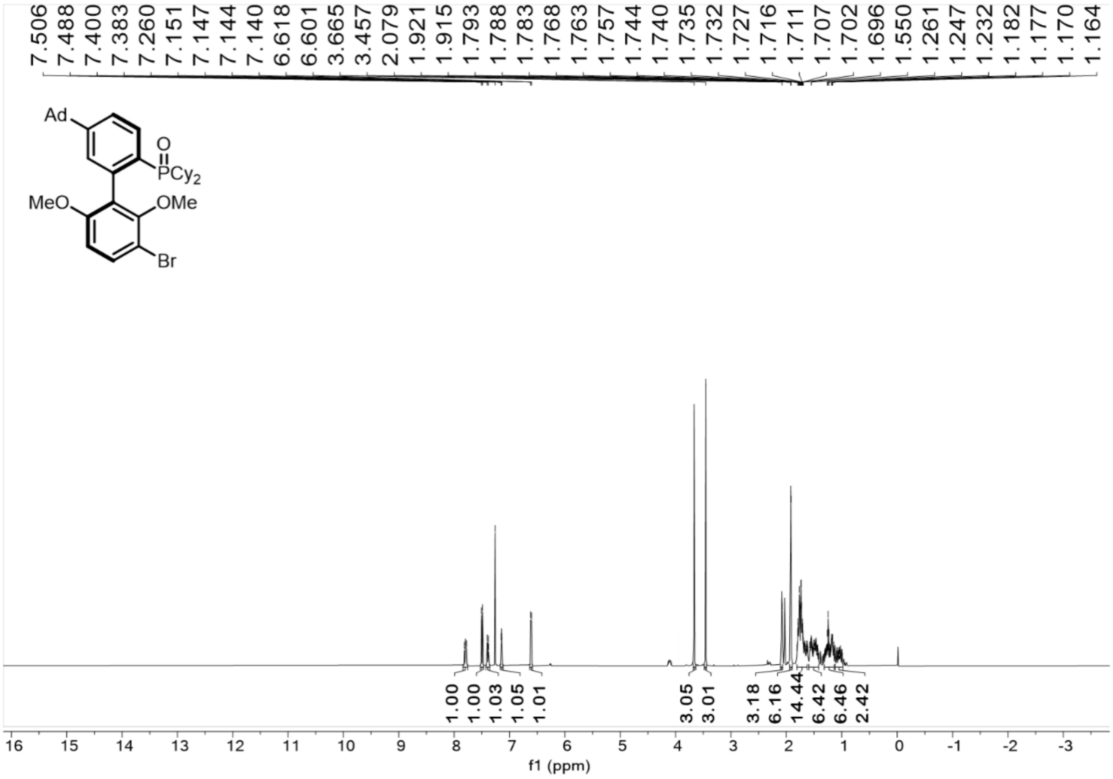

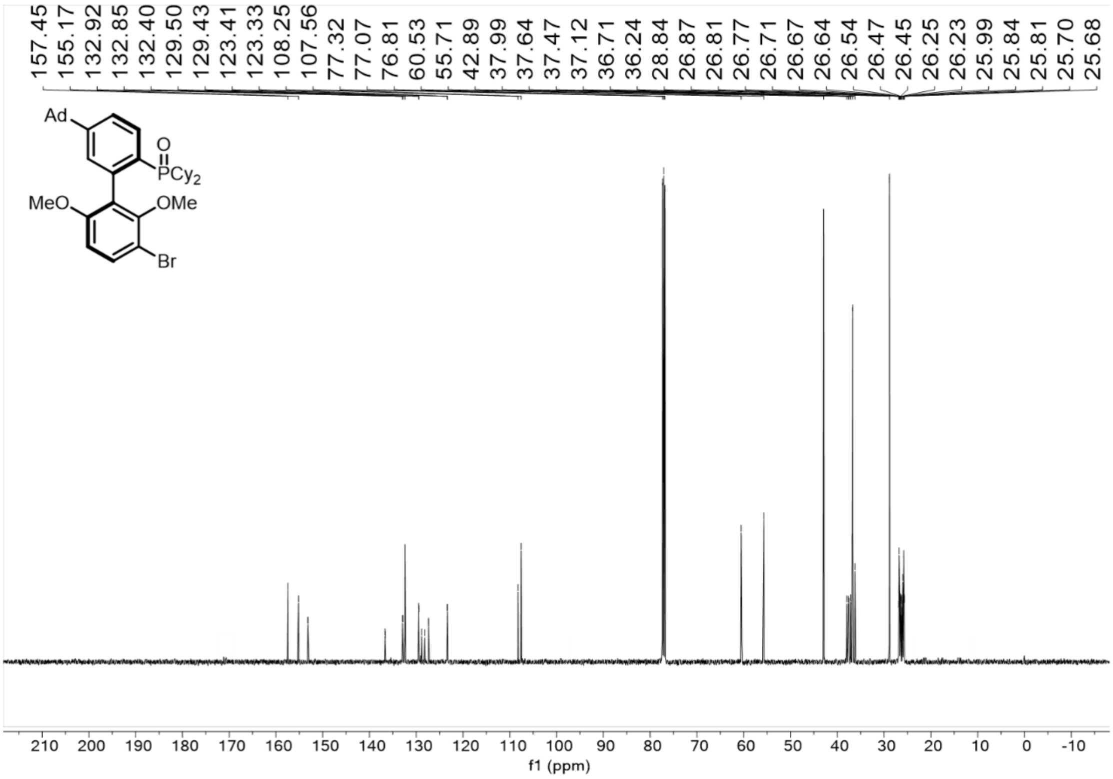

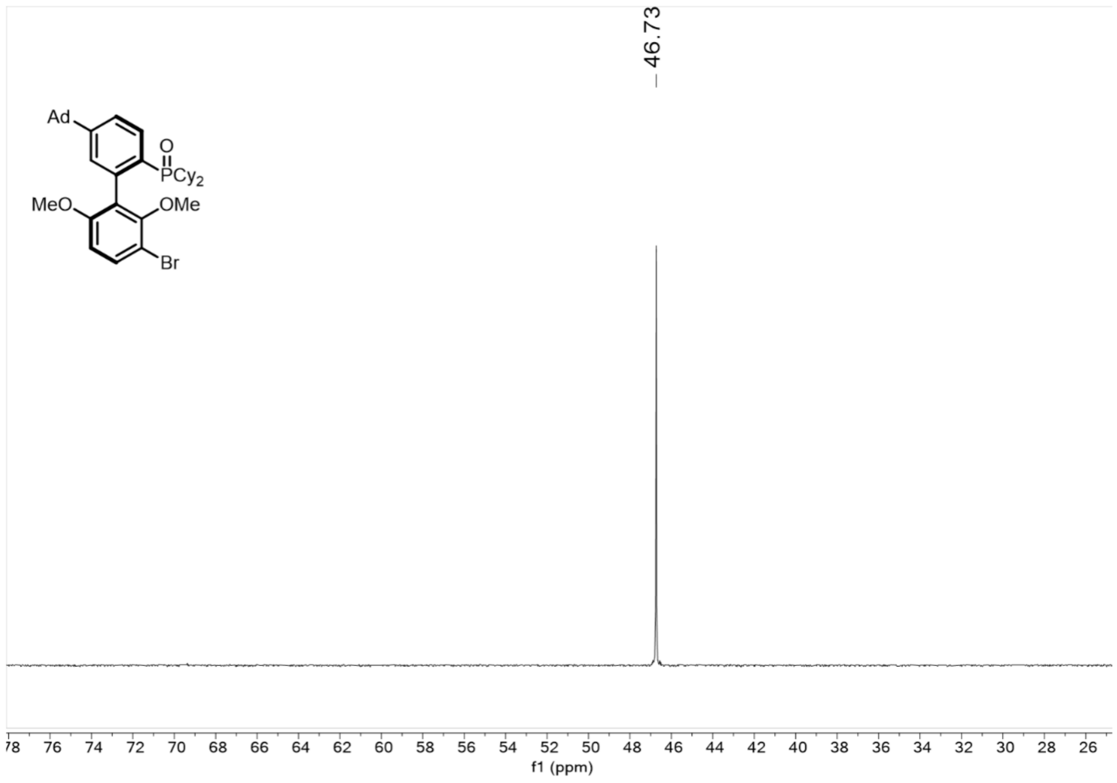

46.73
Ad
O
PCy2
MeO
OMe
Br
78 76 74 72 70 68 66 64 62 60 58 56 54 52 50 48 46 44 42 40 38 36 34 32 30 28 26
f1 (ppm)

(*R*)-(5-benzyl-3'-bromo-2',6'-dimethoxy-[1,1'-biphenyl]-2-yl)dicyclohexylphosphine oxide (11); CDCl₃

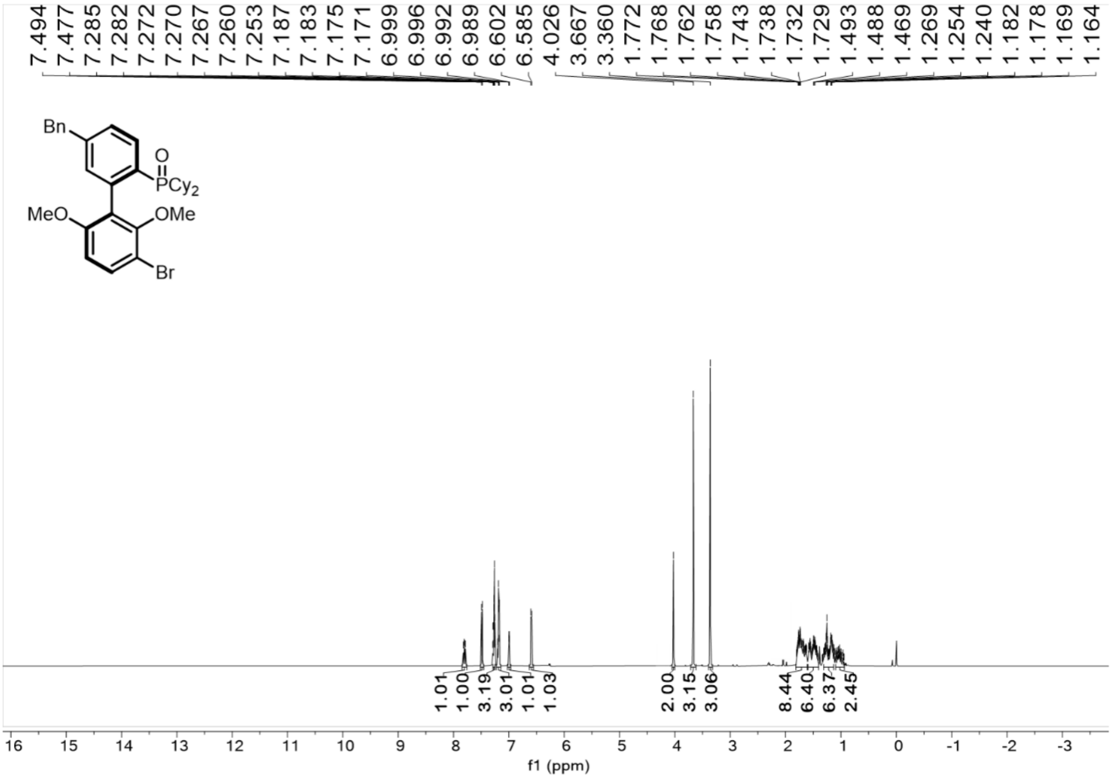

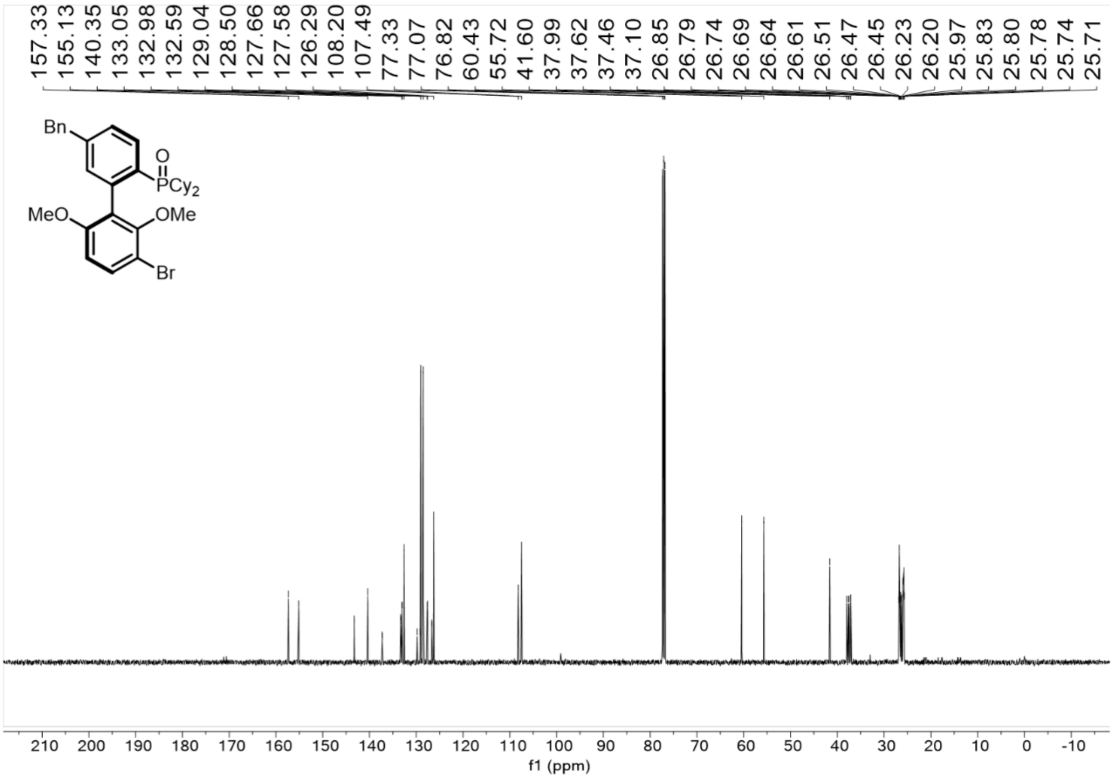

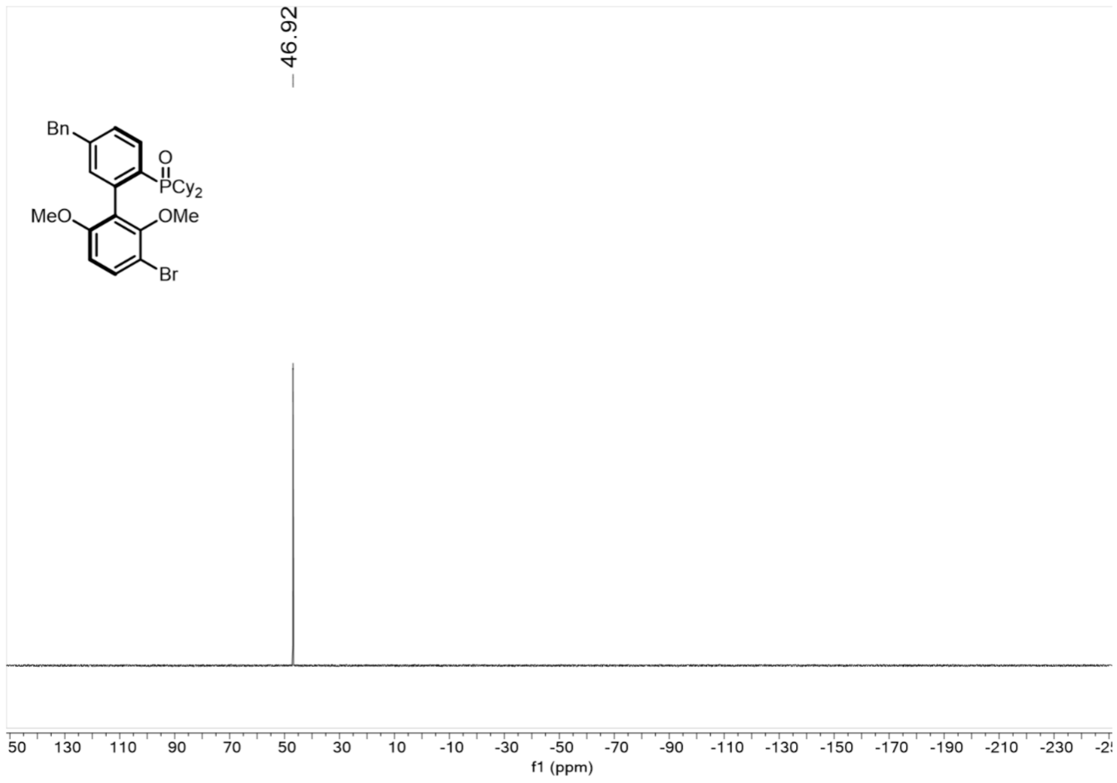

46.92
Bn
O
PCy2
MeO
OMe
Br
50
130
110
90
70
50
30
10
-10
-30
-50
-70
-90
-110
-130
-150
-170
-190
-210
-230
f1 (ppm)

(*R*)-1-(3'-bromo-6-(dicyclohexylphosphoryl)-2',6'-dimethoxy-[1,1'-biphenyl]-3-yl)pyrrolidin-2-one (12); $CDCl_3$

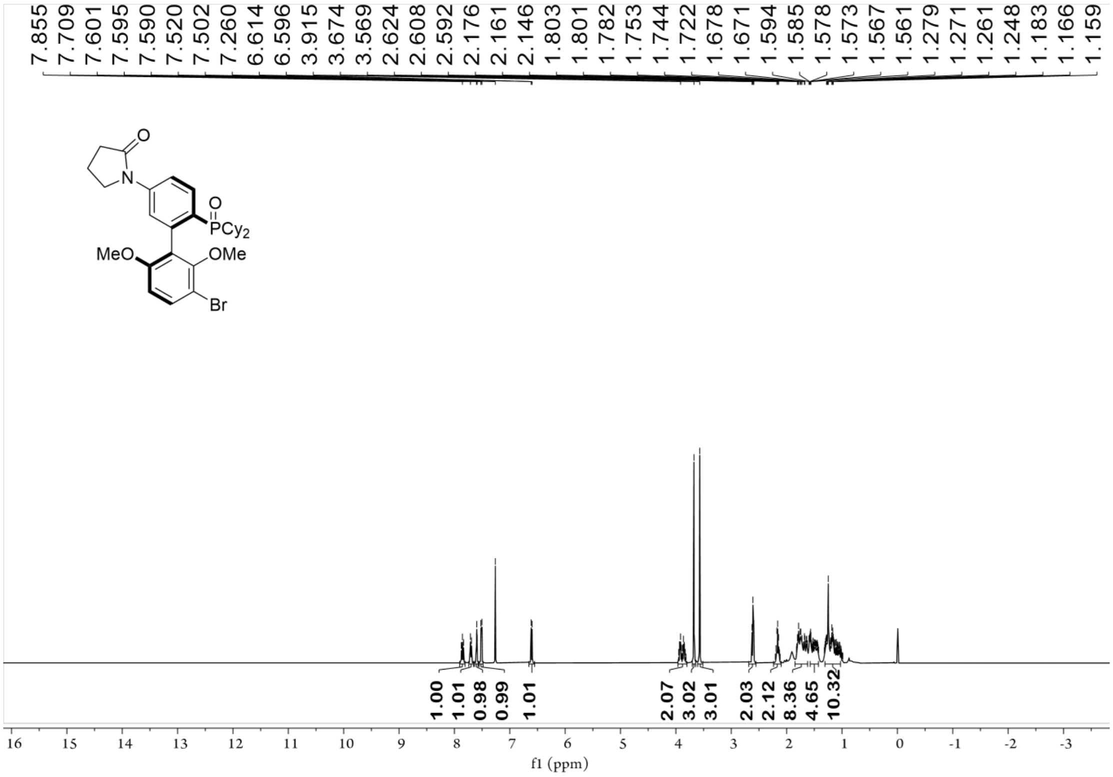

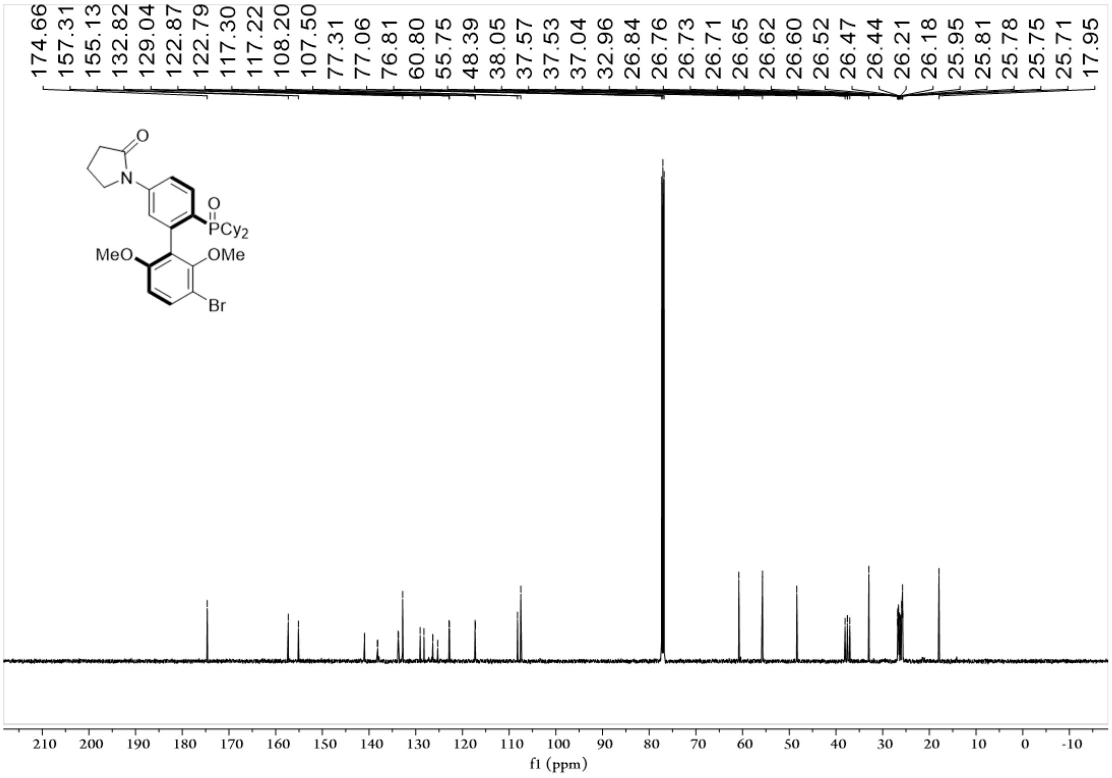

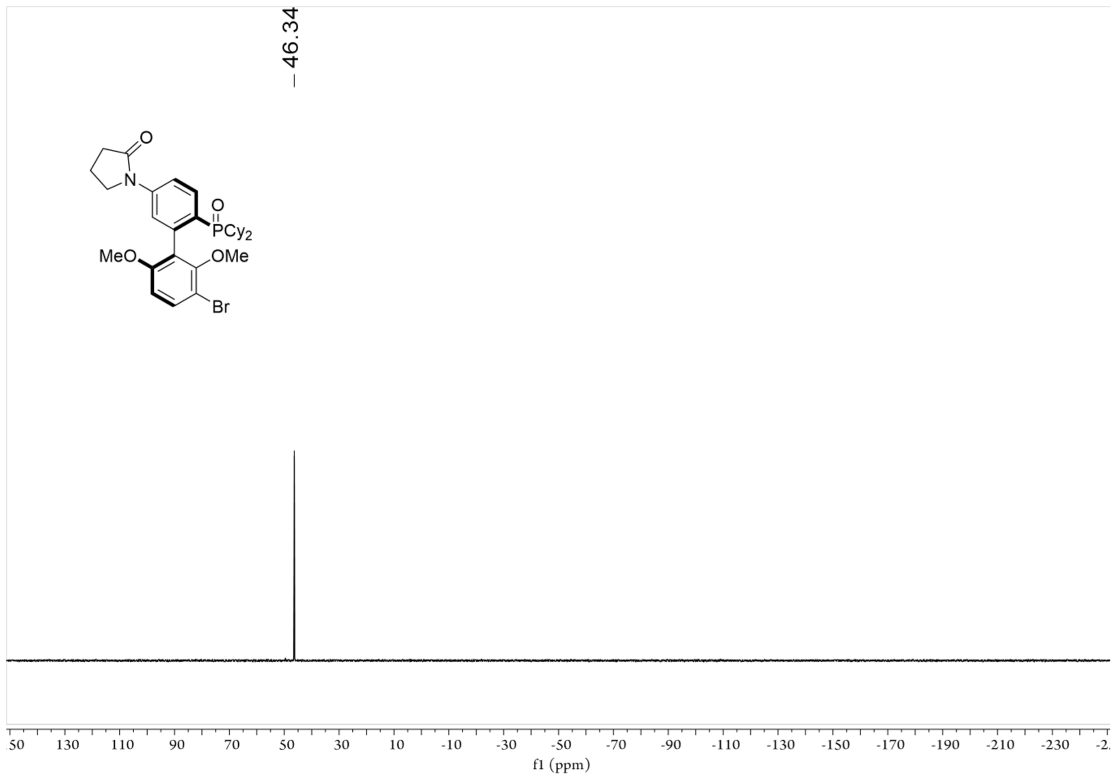
46.34
O
N
O
PCy2
MeO
OMe
Br
50
130
110
90
70
50
30
10
-10
-30
-50
-70
-90
-110
-130
-150
-170
-190
-210
-230
f1 (ppm)

(*R*)-(3'-bromo-5-fluoro-2',6'-dimethoxy-[1,1'-biphenyl]-2-yl)dicyclohexylphosphine oxide (13); $CDCl_3$

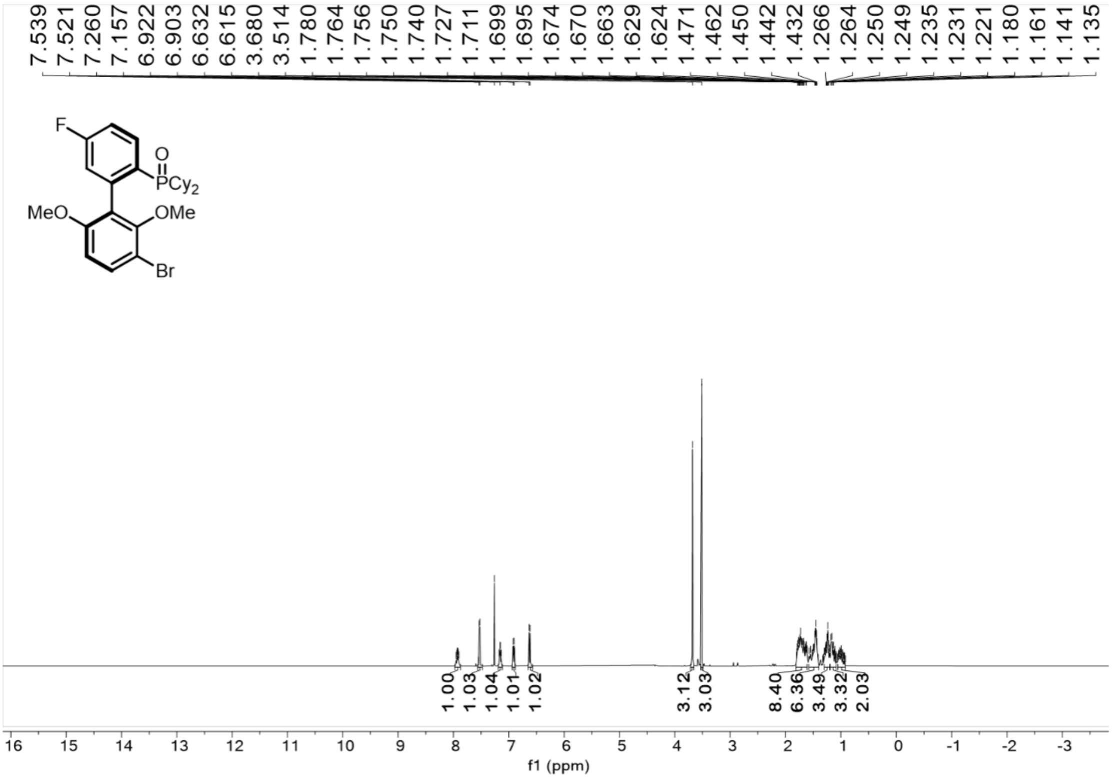

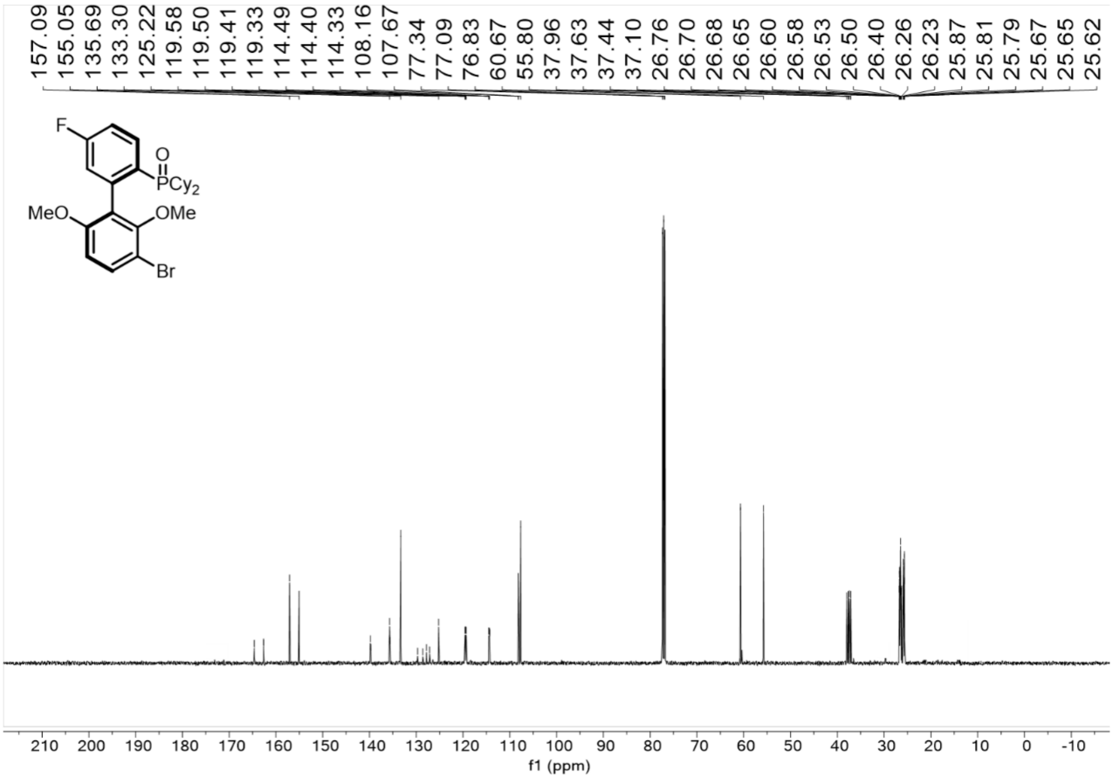

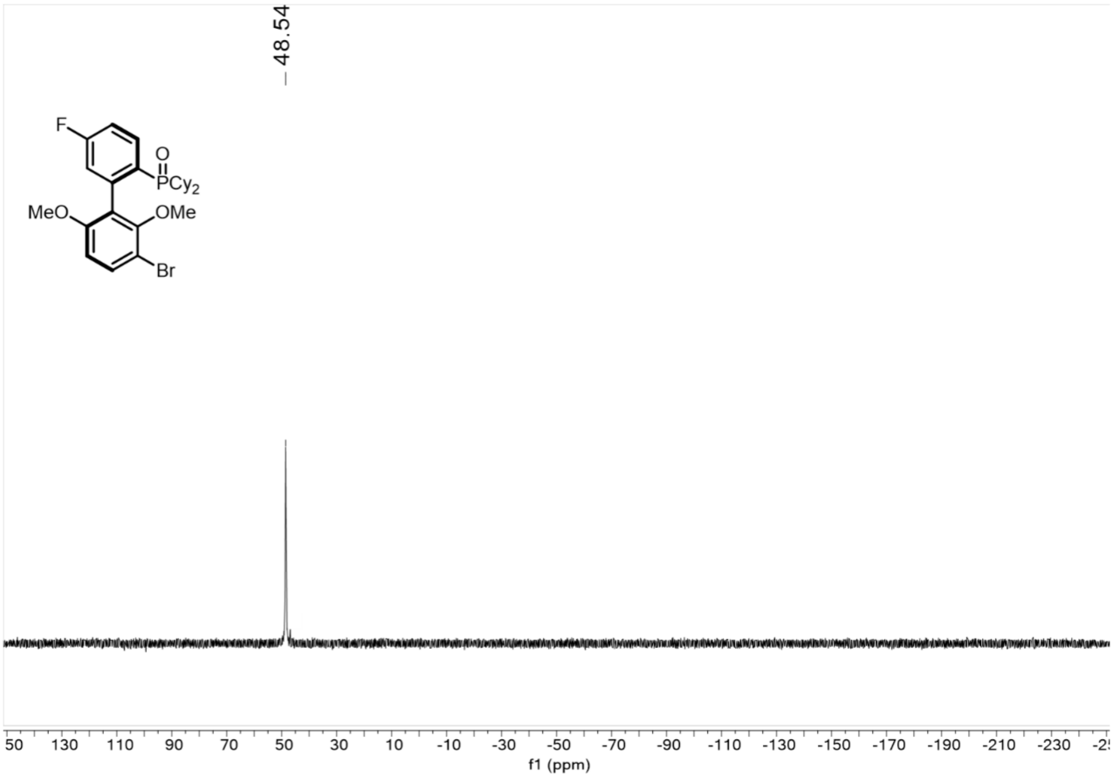
48.54
F
O
PCy2
MeO
OMe
Br
f1 (ppm)

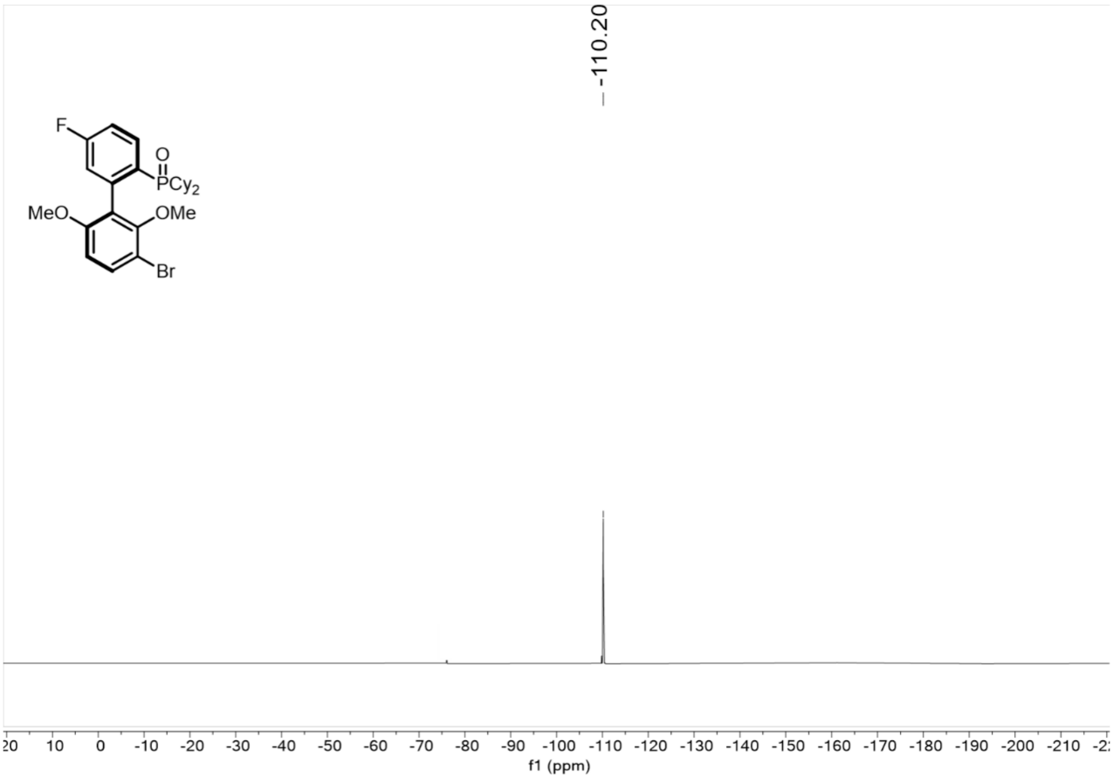
-110.20
F
O
PCy2
MeO
OMe
Br
f1 (ppm)

(*R*)-(3'-bromo-5-chloro-2',6'-dimethoxy-[1,1'-biphenyl]-2-yl)dicyclohexylphosphine oxide (14); **$CDCl_3$**

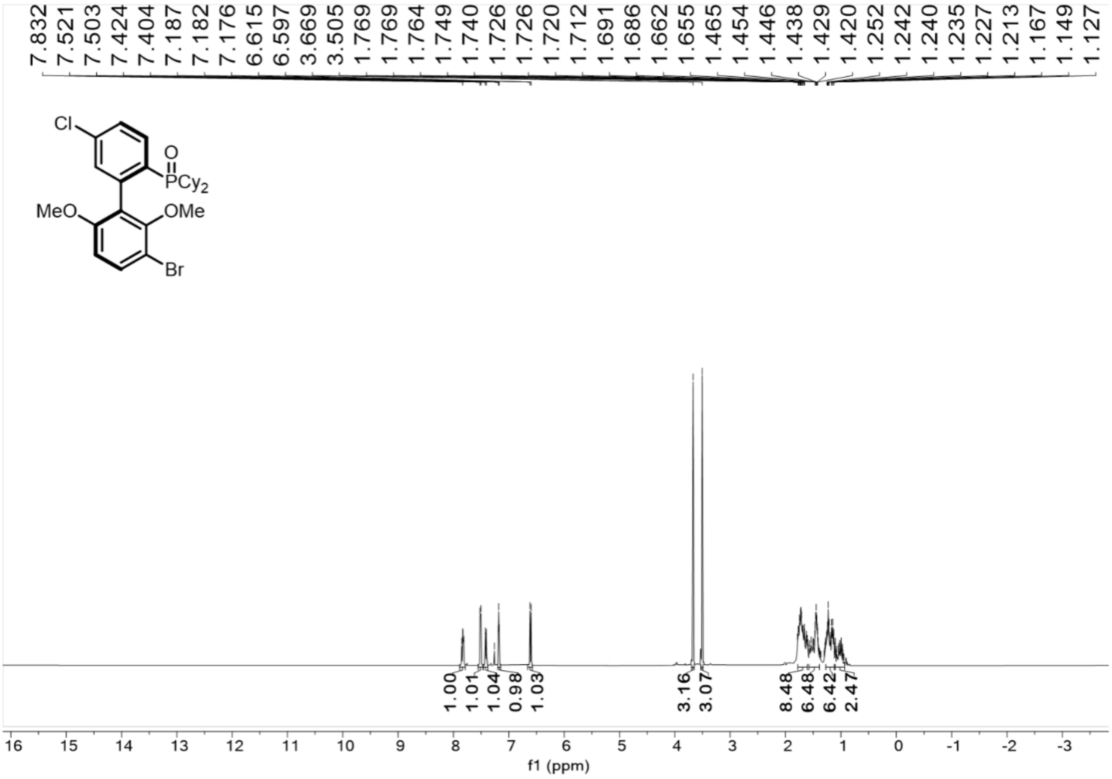

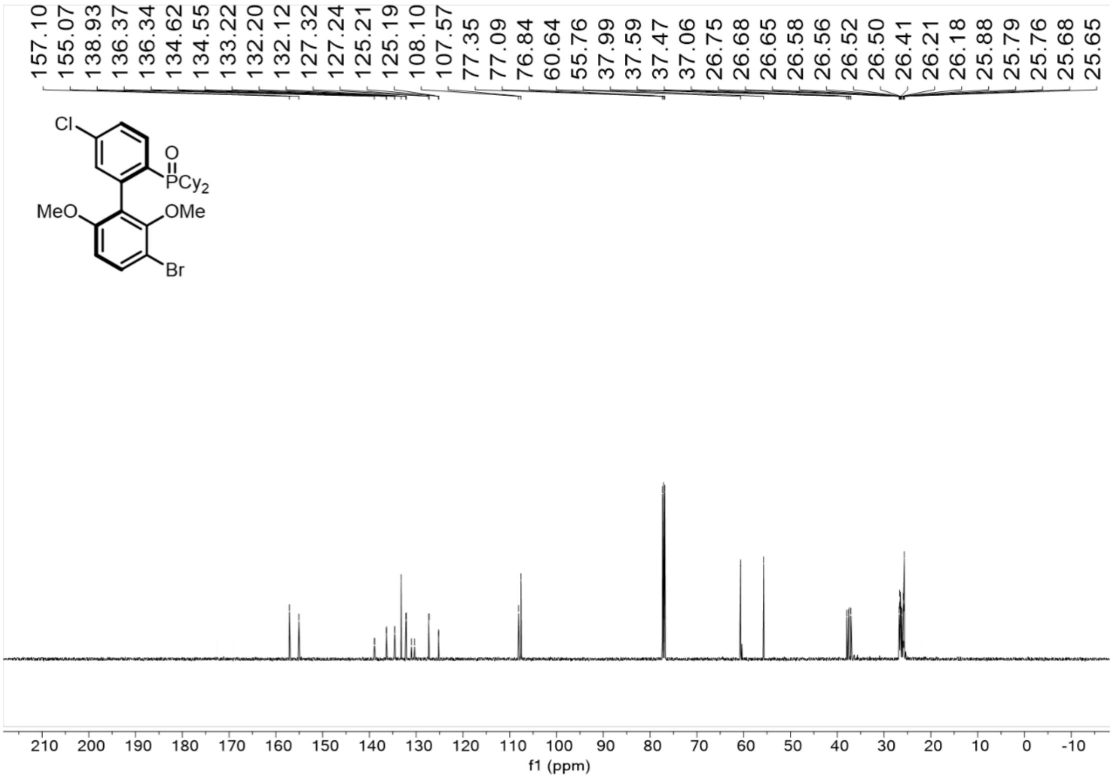

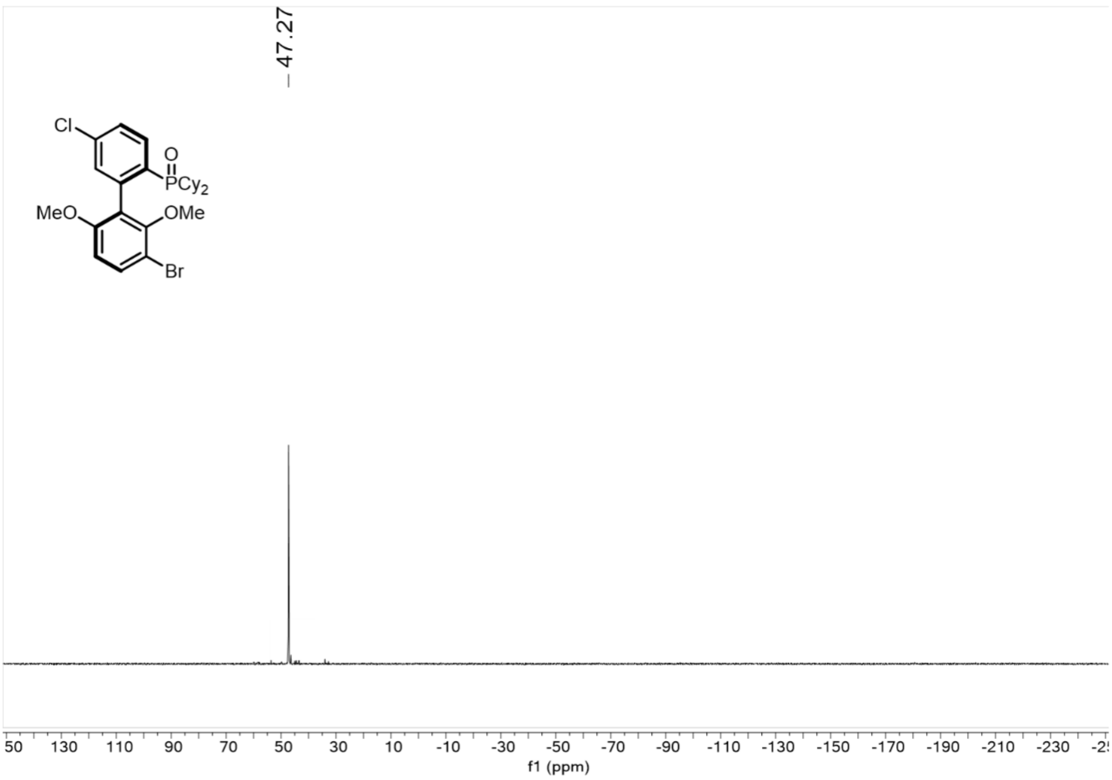

47.27
Cl
O
PCy2
MeO
OMe
Br
50 130 110 90 70 50 30 10 -10 -30 -50 -70 -90 -110 -130 -150 -170 -190 -210 -230
f1 (ppm)

(*R*)-(3'-bromo-2',6'-dimethoxy-5-(trifluoromethyl)-[1,1'-biphenyl]-2-yl)dicyclohexylphosphine oxide (15); $CDCl_3$

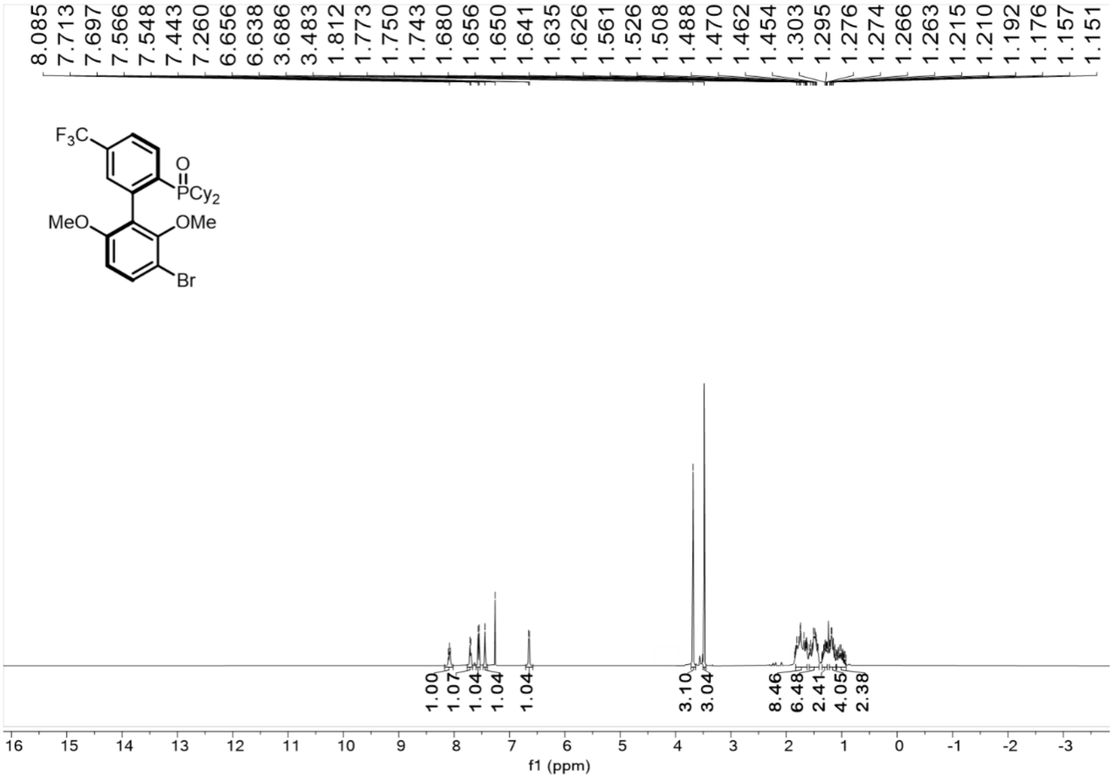

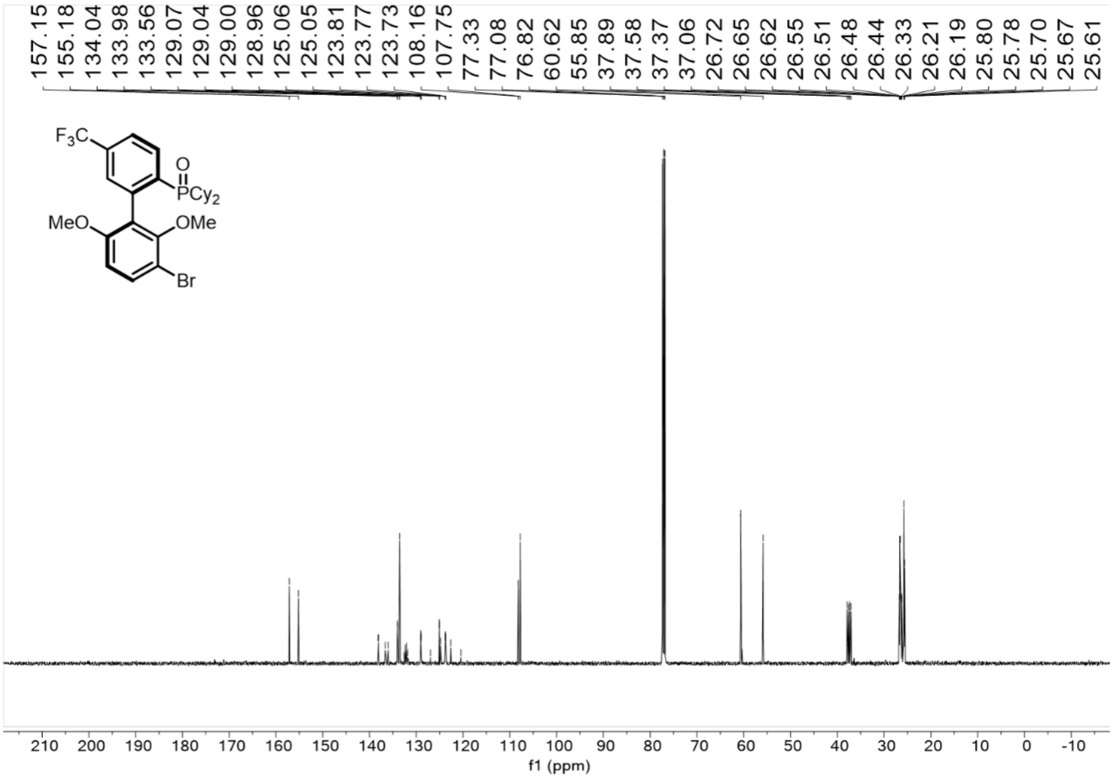

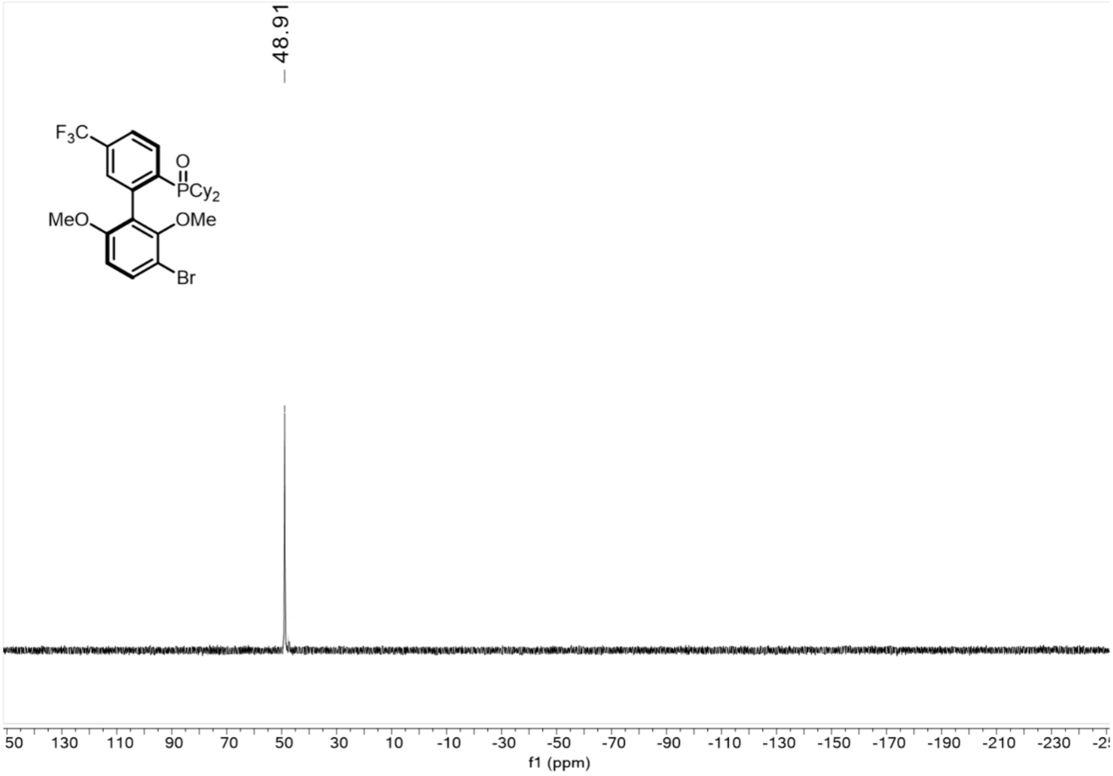

48.91
F3C
O
PCy2
MeO
OMe
Br
50 130 110 90 70 50 30 10 -10 -30 -50 -70 -90 -110 -130 -150 -170 -190 -210 -230
f1 (ppm)

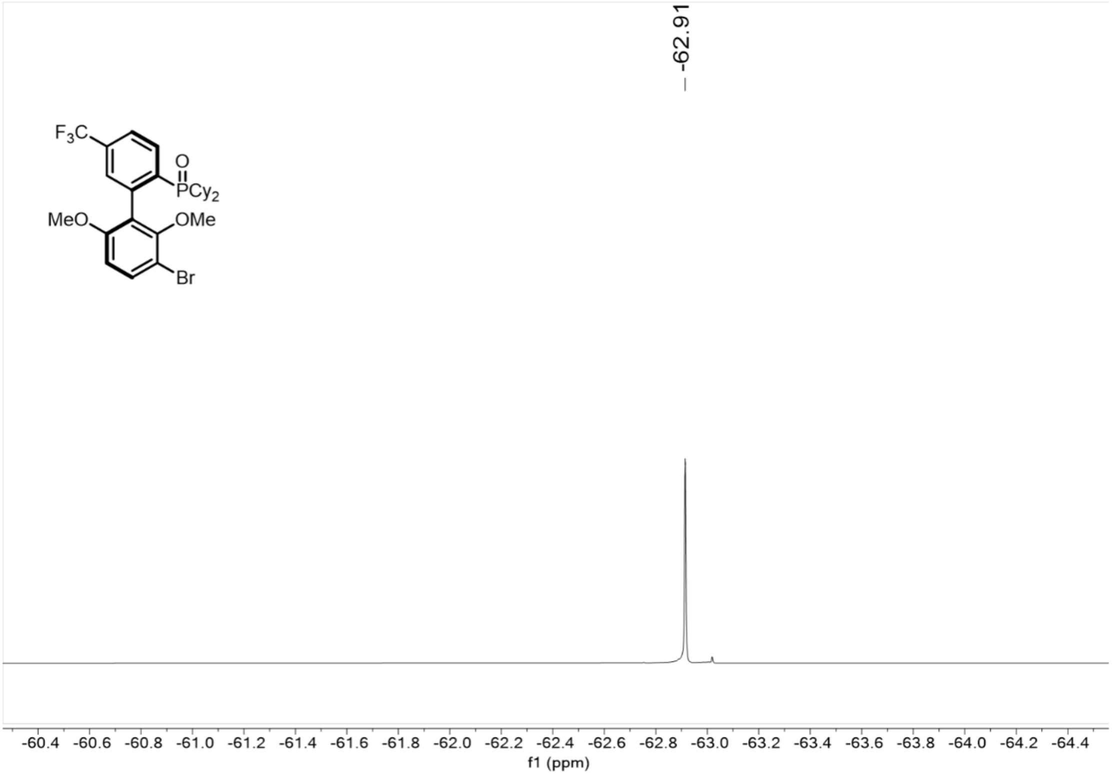

-62.91
F3C
O
PCy2
MeO
OMe
Br
-60.4 -60.6 -60.8 -61.0 -61.2 -61.4 -61.6 -61.8 -62.0 -62.2 -62.4 -62.6 -62.8 -63.0 -63.2 -63.4 -63.6 -63.8 -64.0 -64.2 -64.4
f1 (ppm)

(*R*)-(3'-bromo-2',6'-dimethoxy-4,5-dimethyl-[1,1'-biphenyl]-2-yl)dicyclohexylphosphine oxide (16); $CDCl_3$

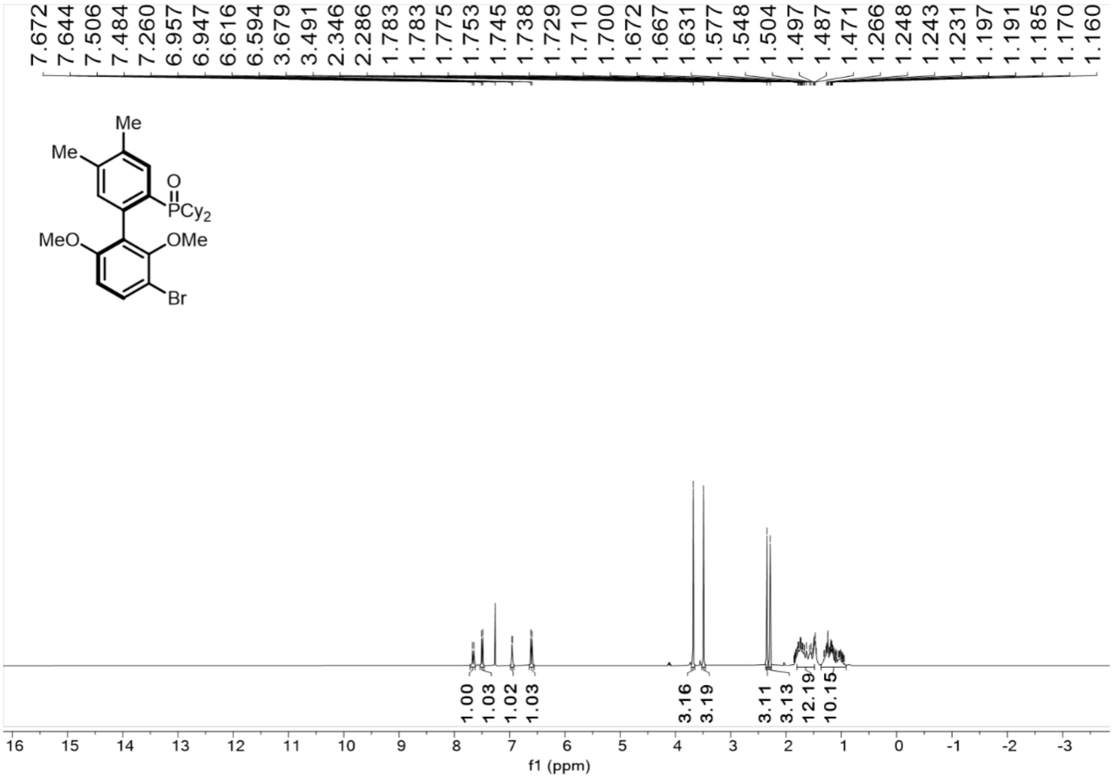

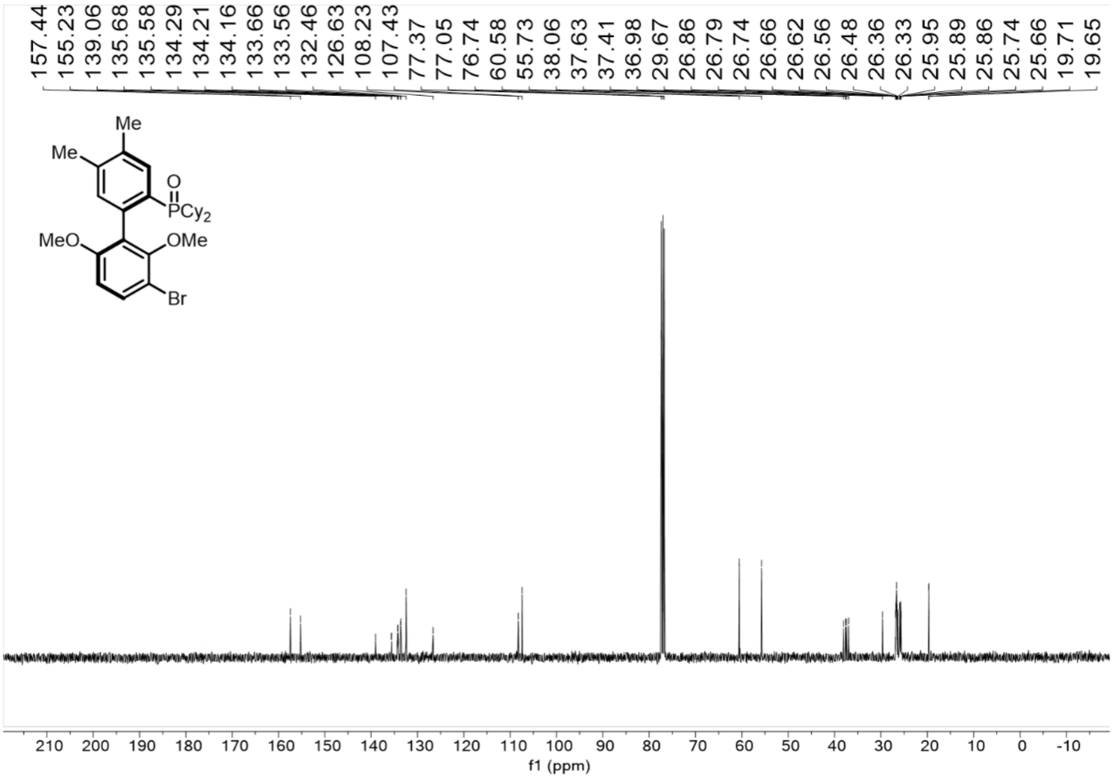

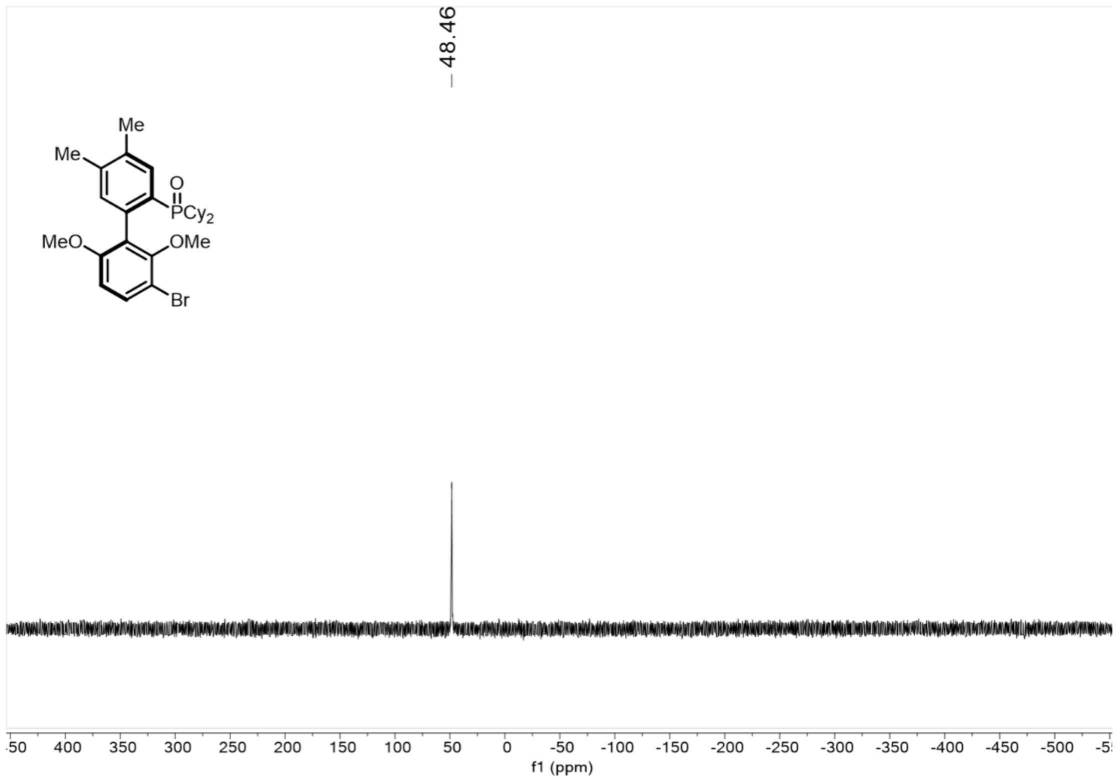

48.46
Me
Me
O
PCy2
MeO
OMe
Br
50
400
350
300
250
200
150
100
50
0
-50
-100
-150
-200
-250
-300
-350
-400
-450
-500
f1 (ppm)

(*R*)-(3'-bromo-2',4,5,6'-tetramethoxy-[1,1'-biphenyl]-2-yl)dicyclohexylphosphine oxide (17); $CDCl_3$

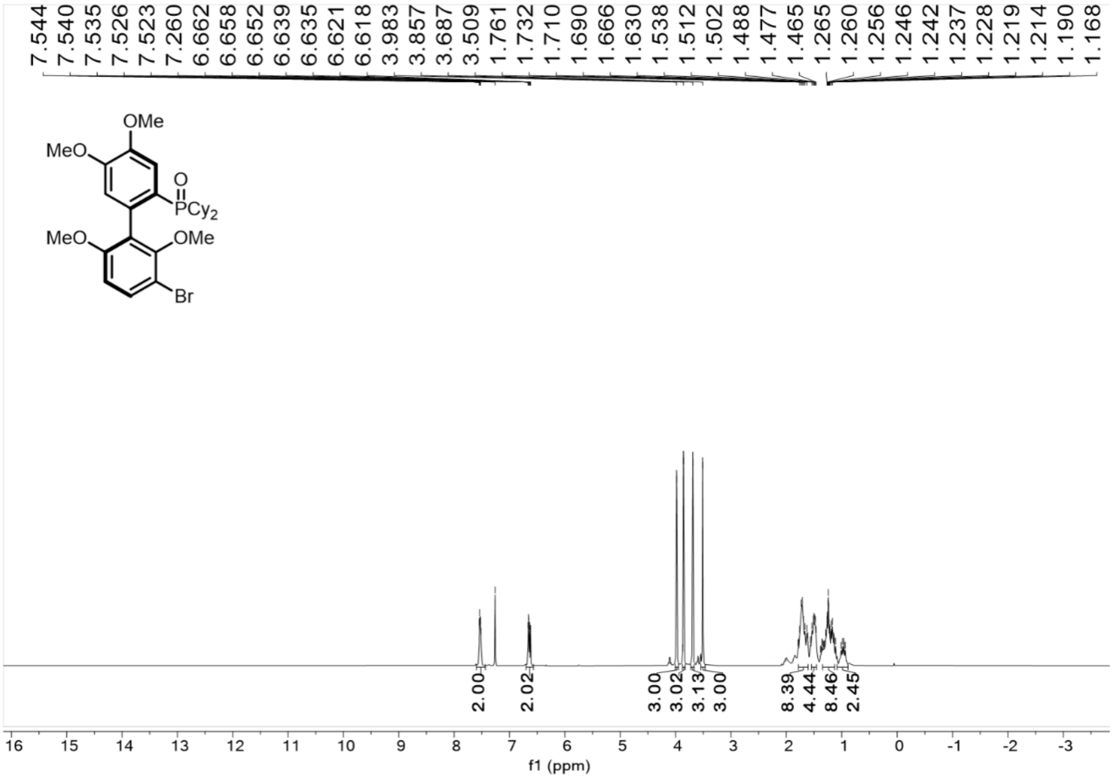

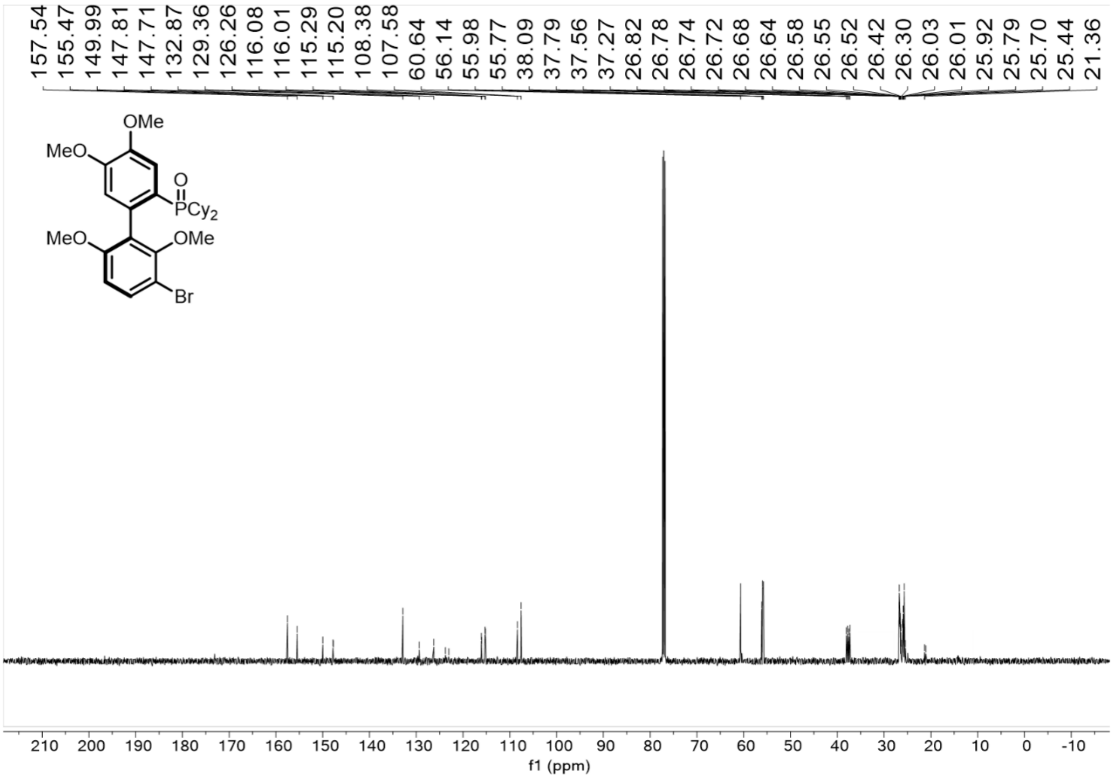

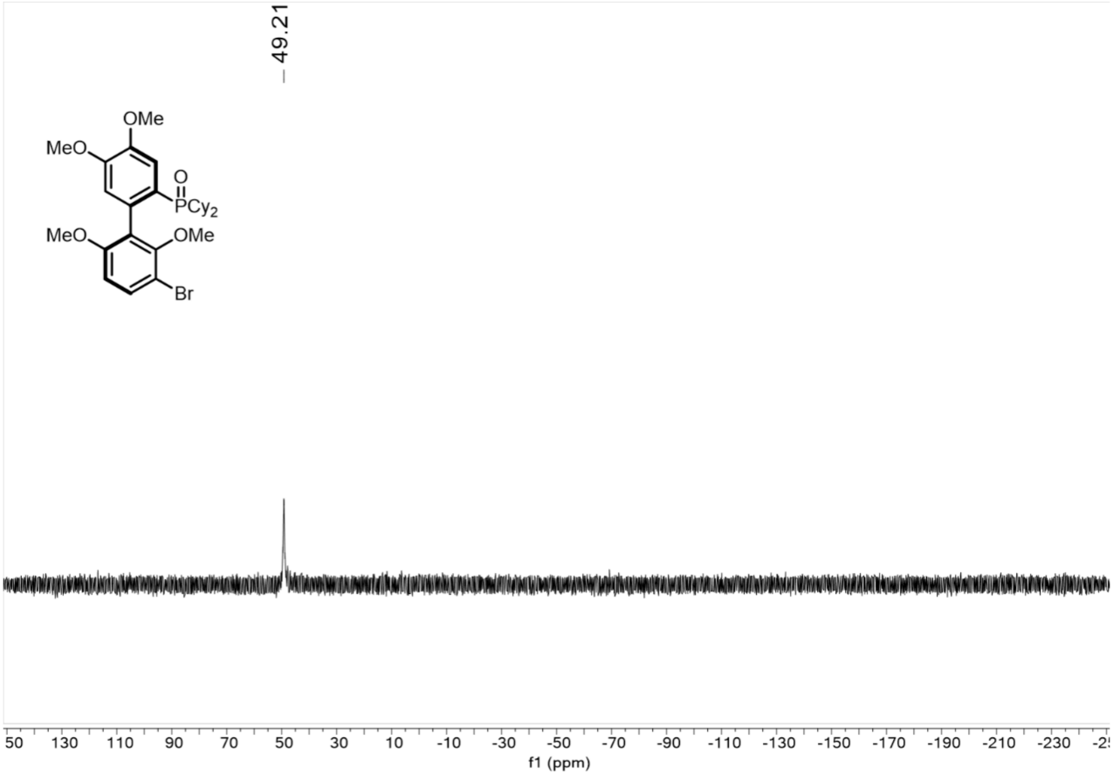

49.21
OMe
MeO
O
PCy2
MeO
OMe
Br
50
130
110
90
70
50
30
10
-10
-30
-50
-70
-90
-110
-130
-150
-170
-190
-210
-230
f1 (ppm)

(*R*)-(3'-bromo-2',6'-dimethoxy-4-methyl-[1,1'-biphenyl]-2-yl)dicyclohexylphosphine oxide (18); CDCl₃

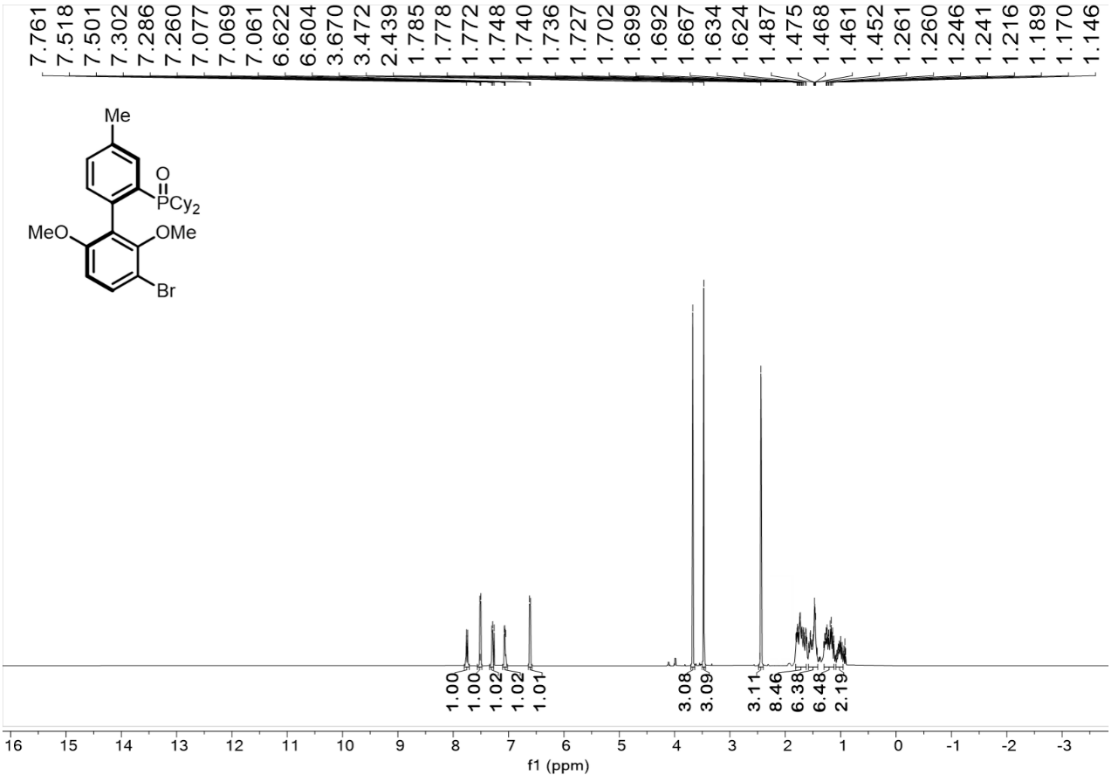

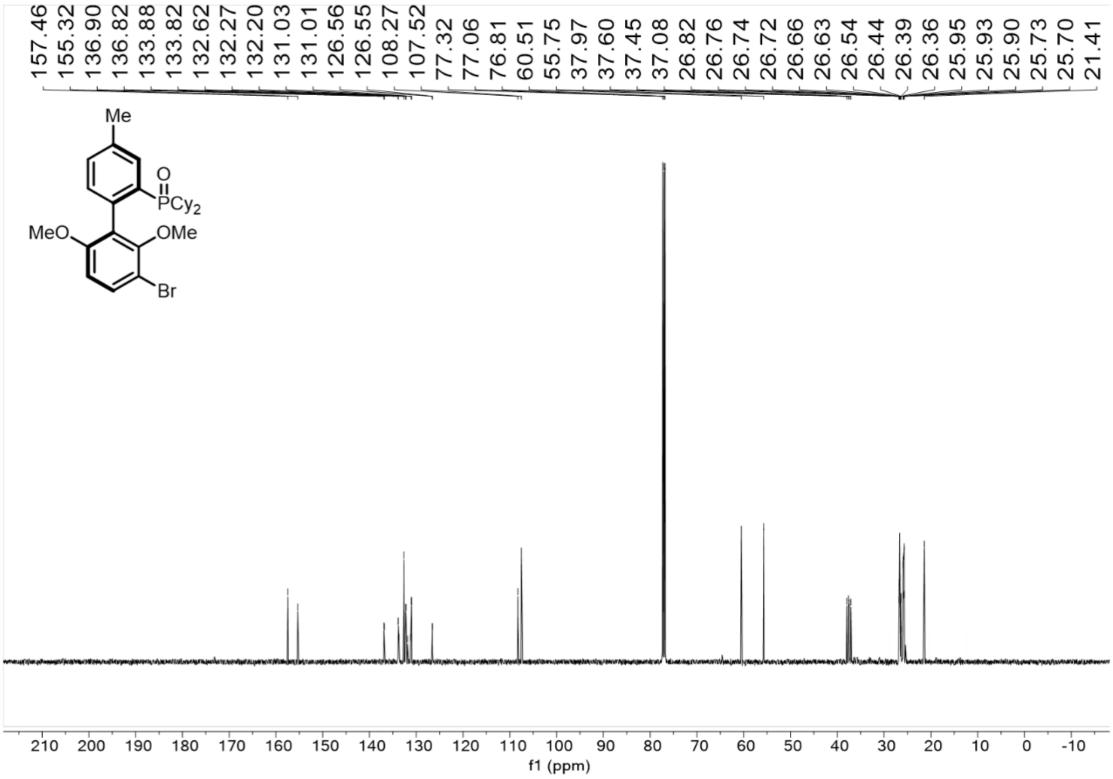

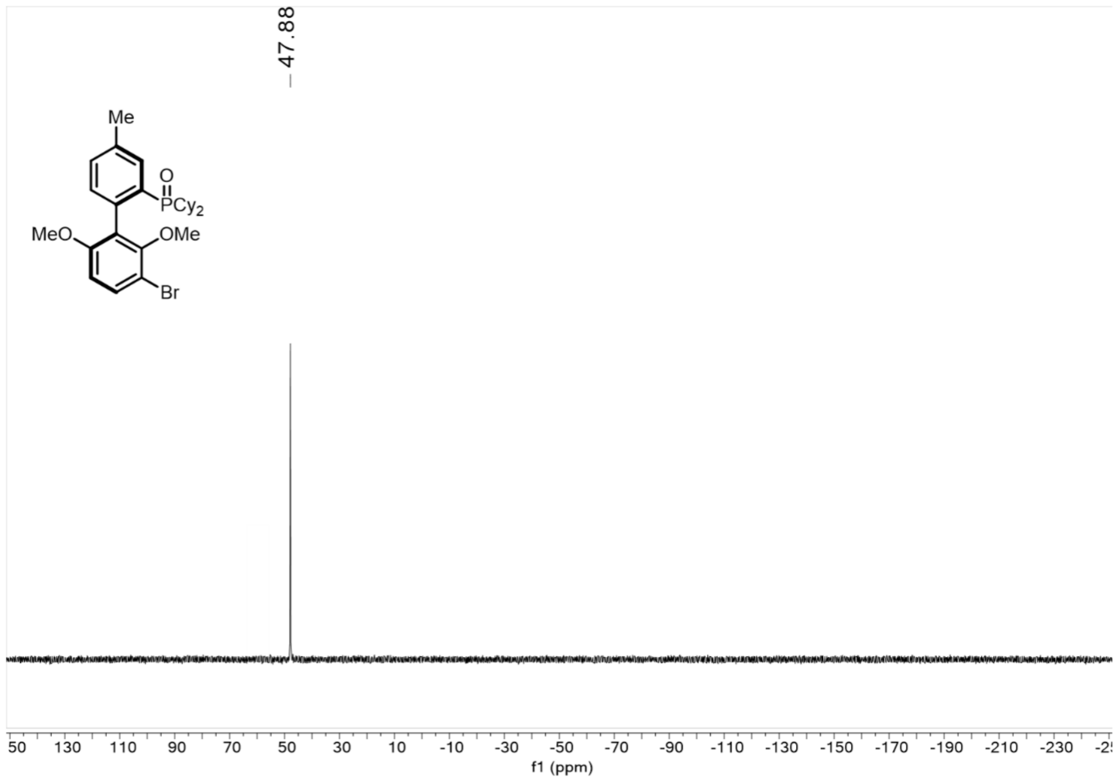

47.88
Me
O
PCy2
MeO
OMe
Br
50
130
110
90
70
50
30
10
-10
-30
-50
-70
-90
-110
-130
-150
-170
-190
-210
-230
f1 (ppm)

(*R*)-(3'-bromo-4-ethyl-2',6'-dimethoxy-[1,1'-biphenyl]-2-yl)dicyclohexylphosphine oxide (19); $CDCl_3$

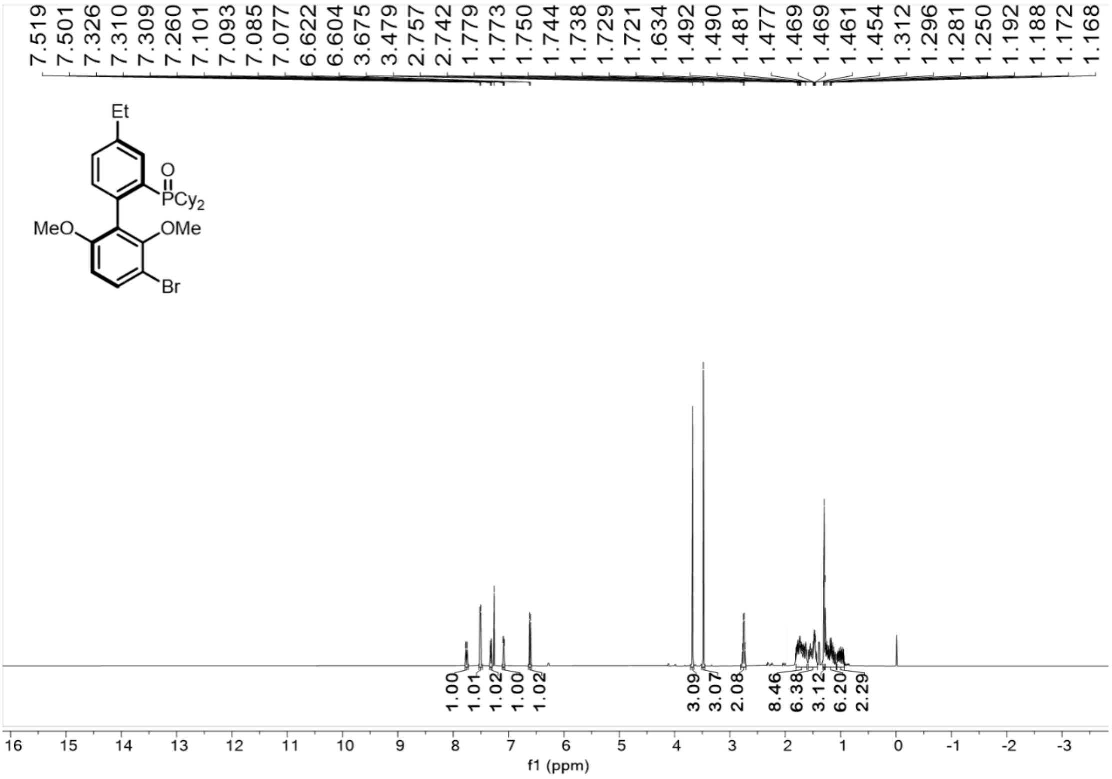

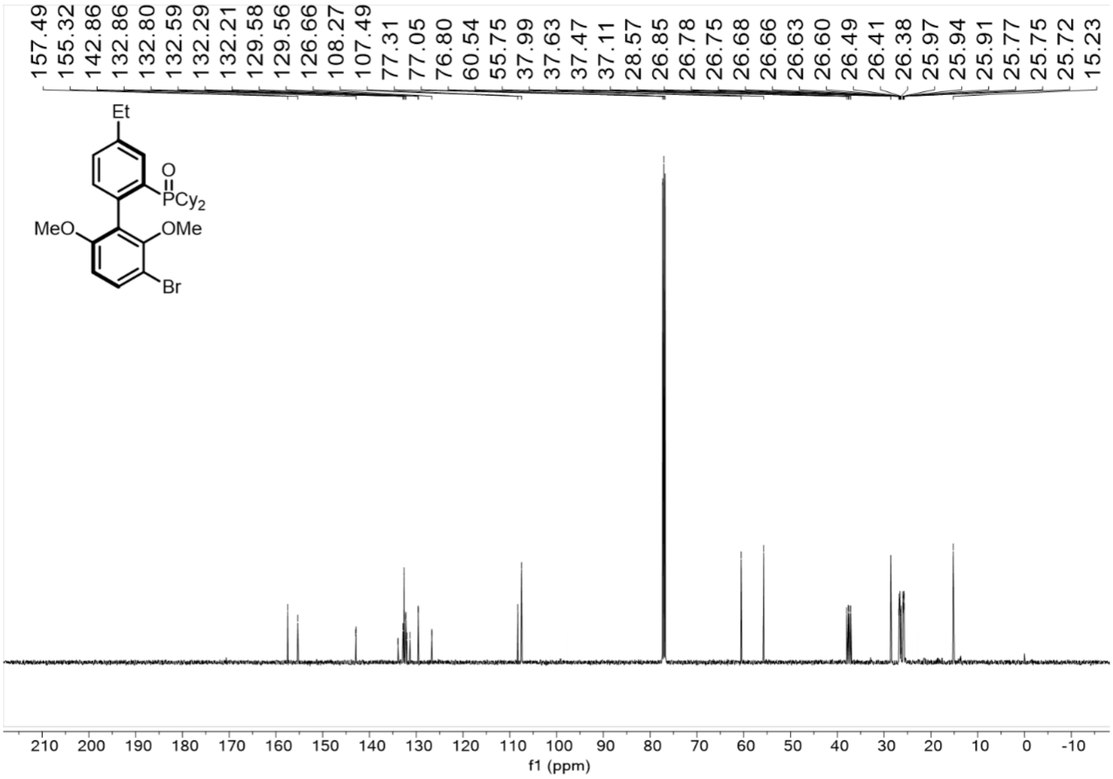

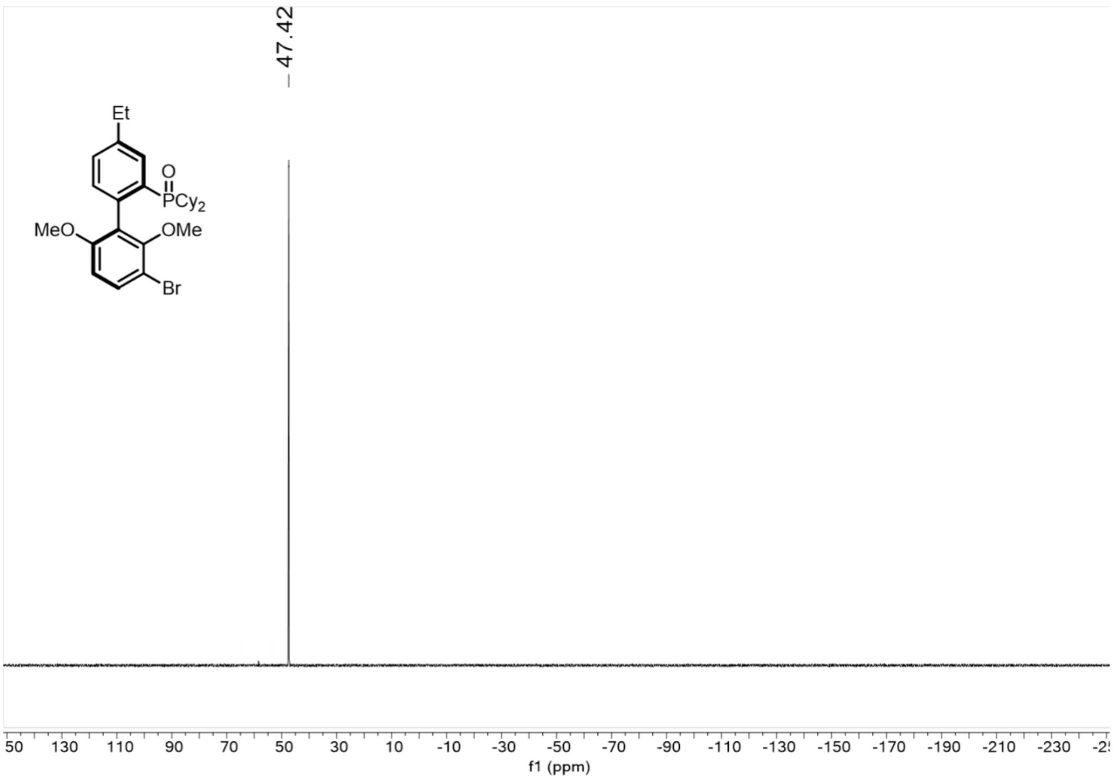
47.42
Et
O
PCy2
MeO
OMe
Br
50
130
110
90
70
50
30
10
-10
-30
-50
-70
-90
-110
-130
-150
-170
-190
-210
-230
f1 (ppm)

(*R*)-(3'-bromo-4-isopropyl-2',6'-dimethoxy-[1,1'-biphenyl]-2-yl)dicyclohexylphosphine oxide (20); CDCl3

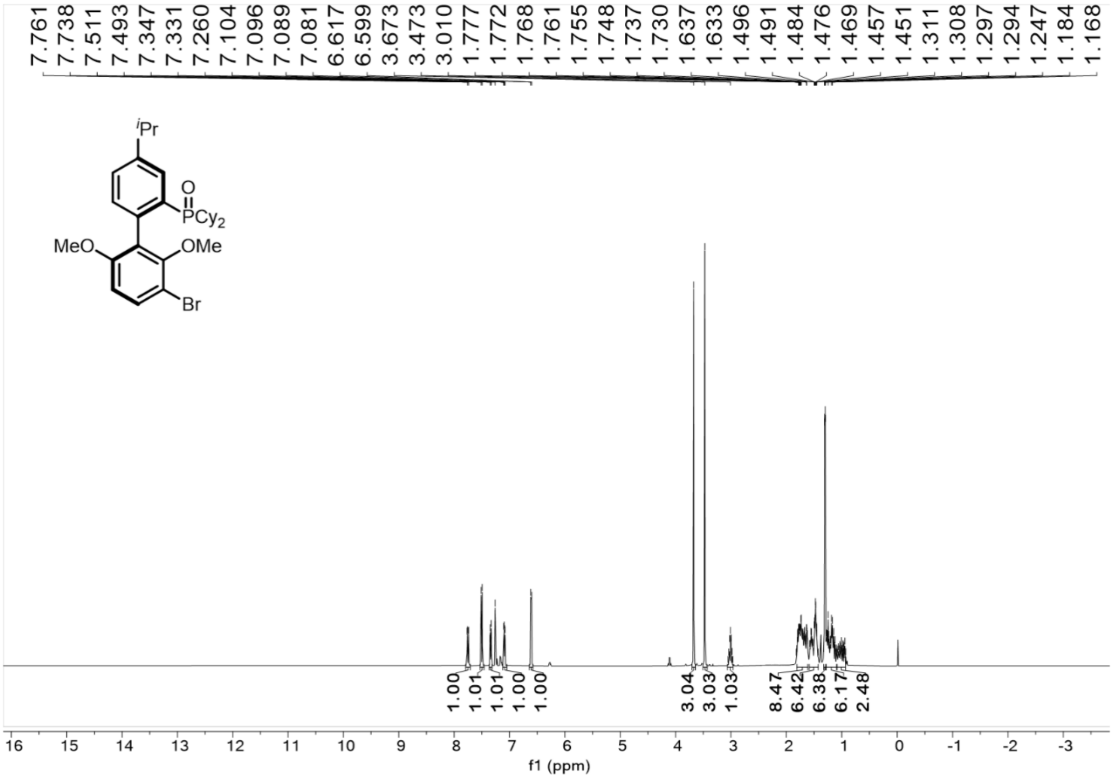

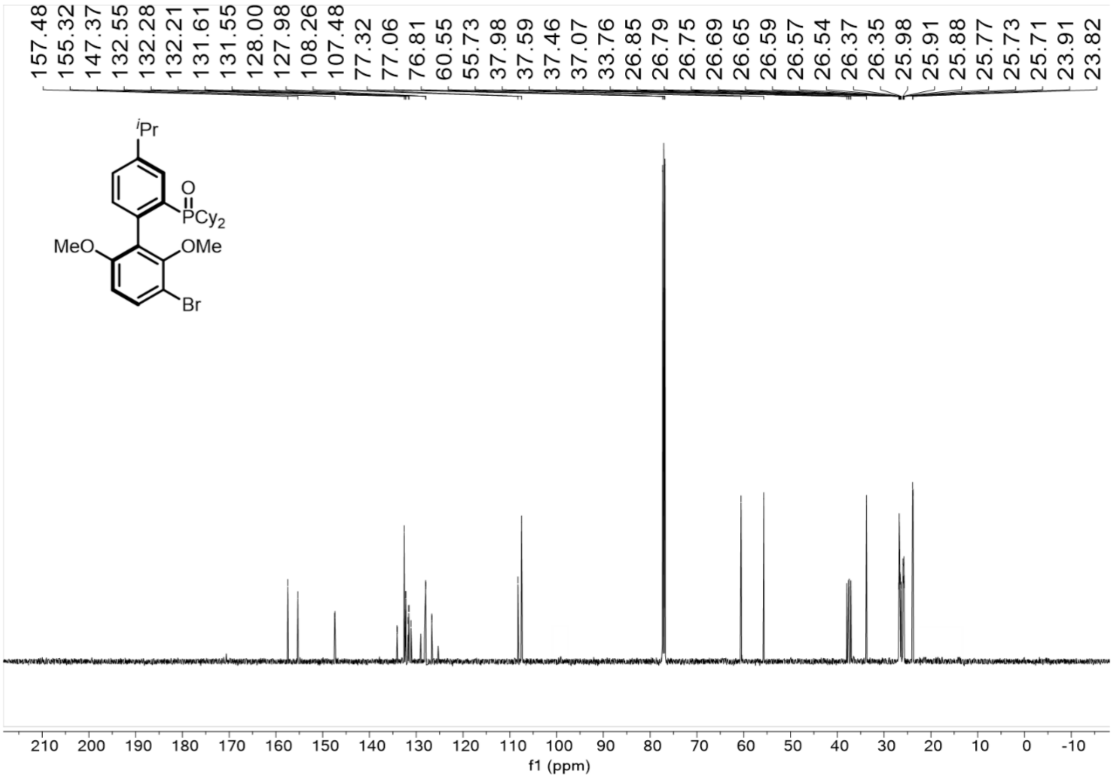

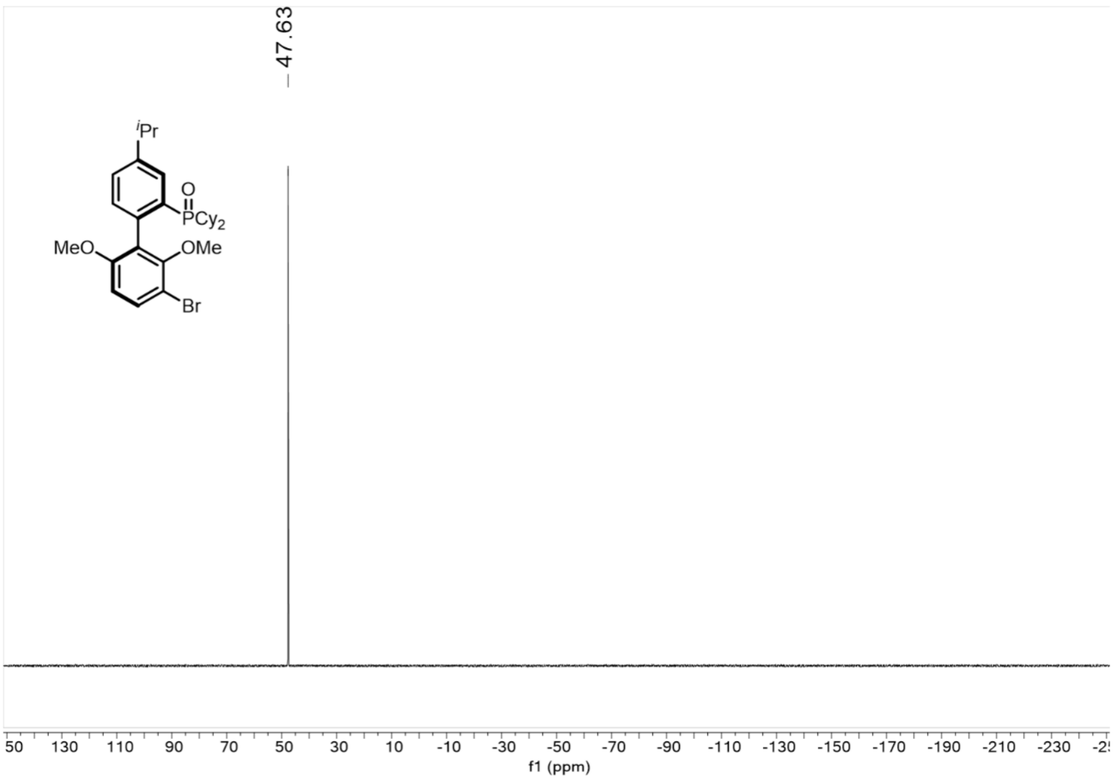
47.63
iPr
O
PCy2
MeO
OMe
Br
50 130 110 90 70 50 30 10 -10 -30 -50 -70 -90 -110 -130 -150 -170 -190 -210 -230
f1 (ppm)

(*R*)-(3'-bromo-4-(*tert*-butyl)-2',6'-dimethoxy-[1,1'-biphenyl]-2-yl)dicyclohexylphosphine oxide (21); CDCl₃

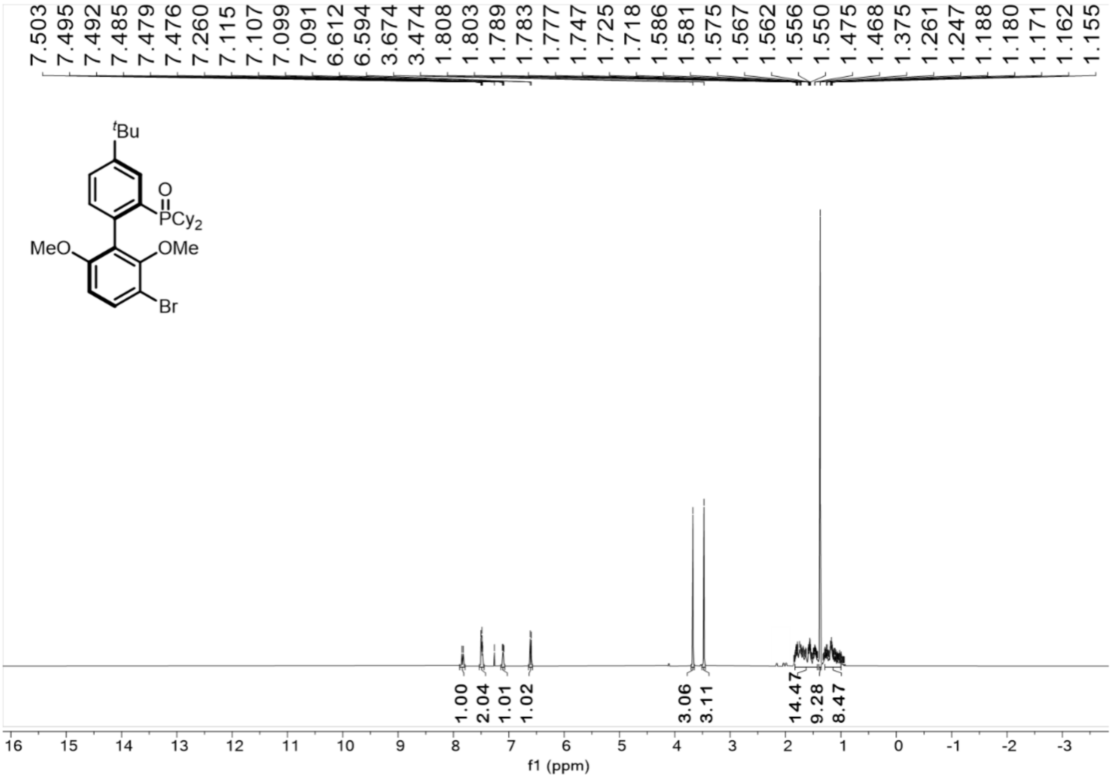

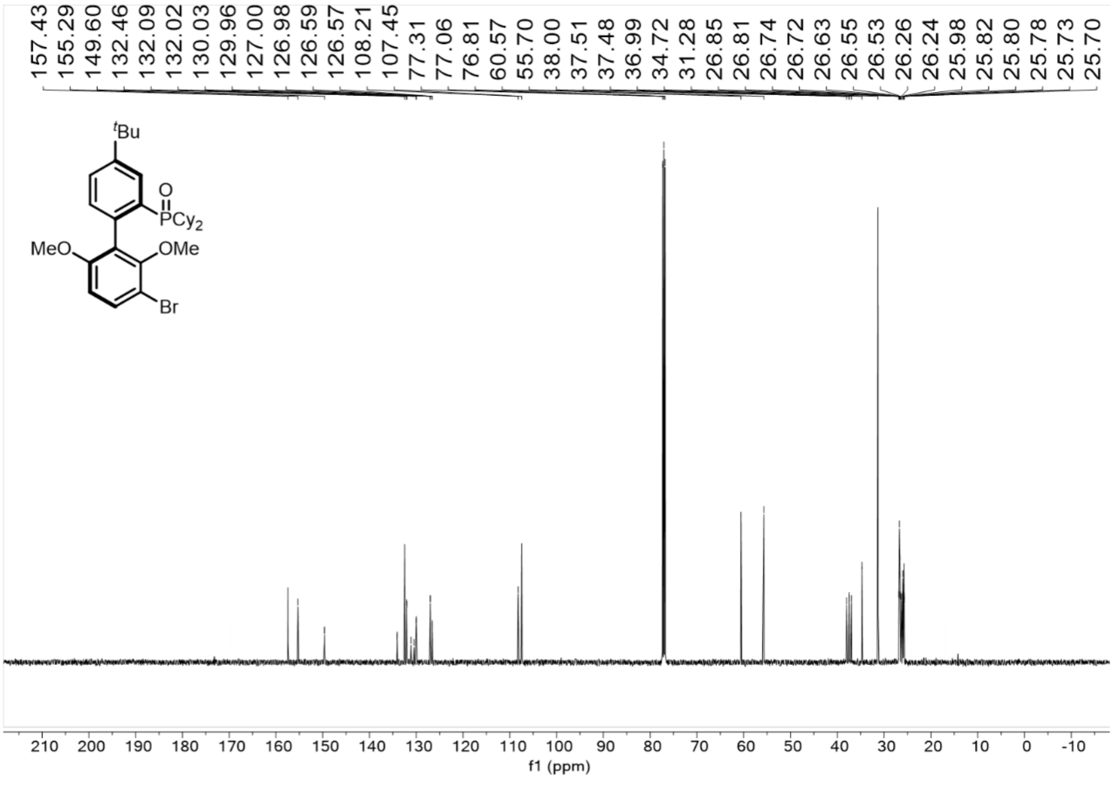

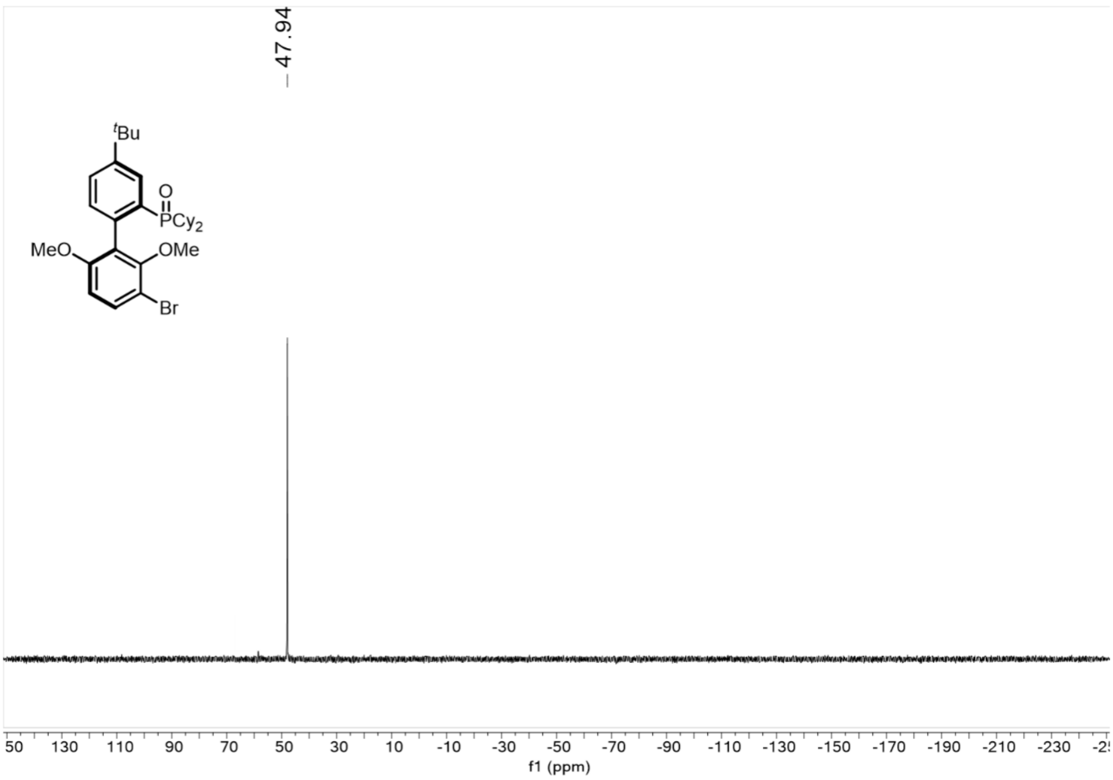
47.94
tBu
O
PCy2
MeO
OMe
Br
50 130 110 90 70 50 30 10 -10 -30 -50 -70 -90 -110 -130 -150 -170 -190 -210 -230
f1 (ppm)

(*R*)-(3'-bromo-2',4,6'-trimethoxy-[1,1'-biphenyl]-2-yl)dicyclohexylphosphine oxide (22); $CDCl_3$

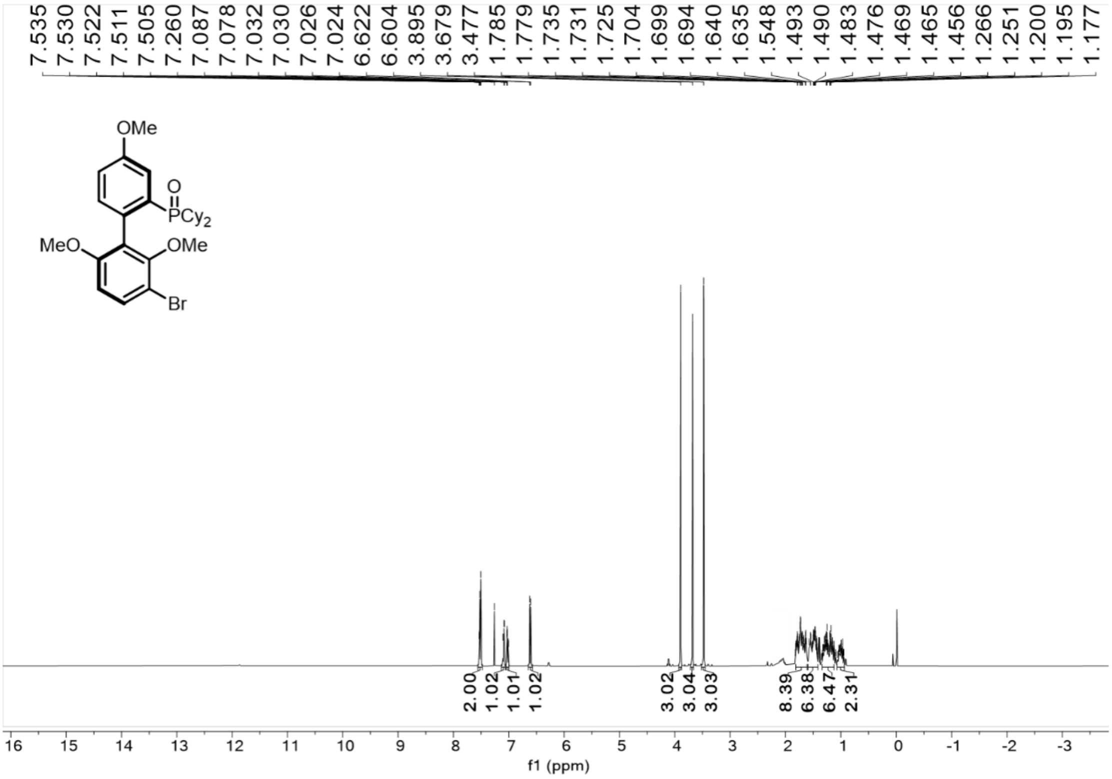

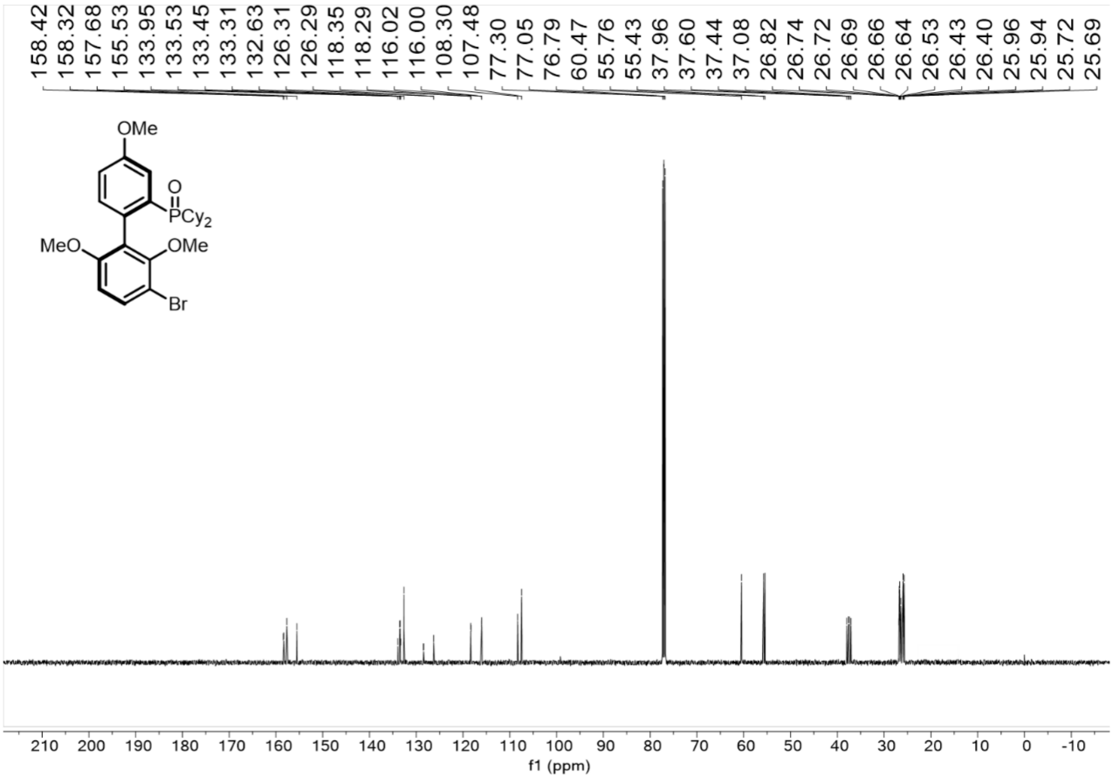

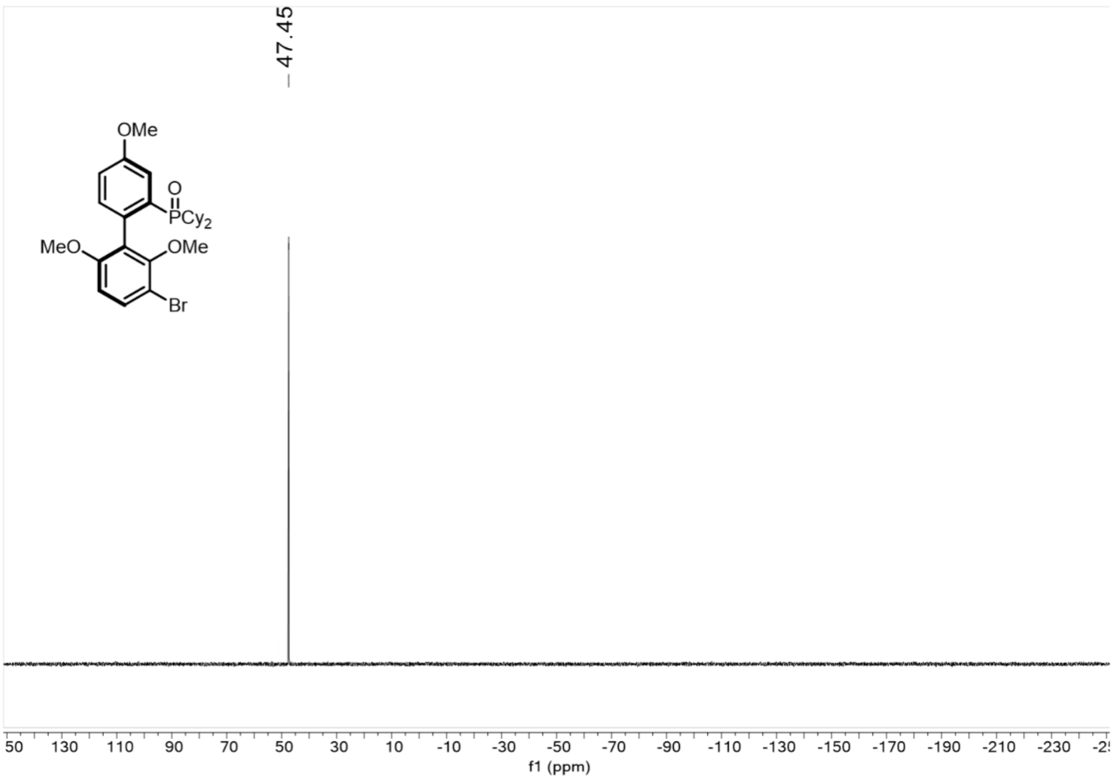

47.45
OMe
O
PCy2
MeO
OMe
Br
50 130 110 90 70 50 30 10 -10 -30 -50 -70 -90 -110 -130 -150 -170 -190 -210 -230
f1 (ppm)

(*R*)-(3'-bromo-2',6'-dimethoxy-4-(trifluoromethoxy)-[1,1'-biphenyl]-2-yl)dicyclohexylphosphine oxide (23); $CDCl_3$

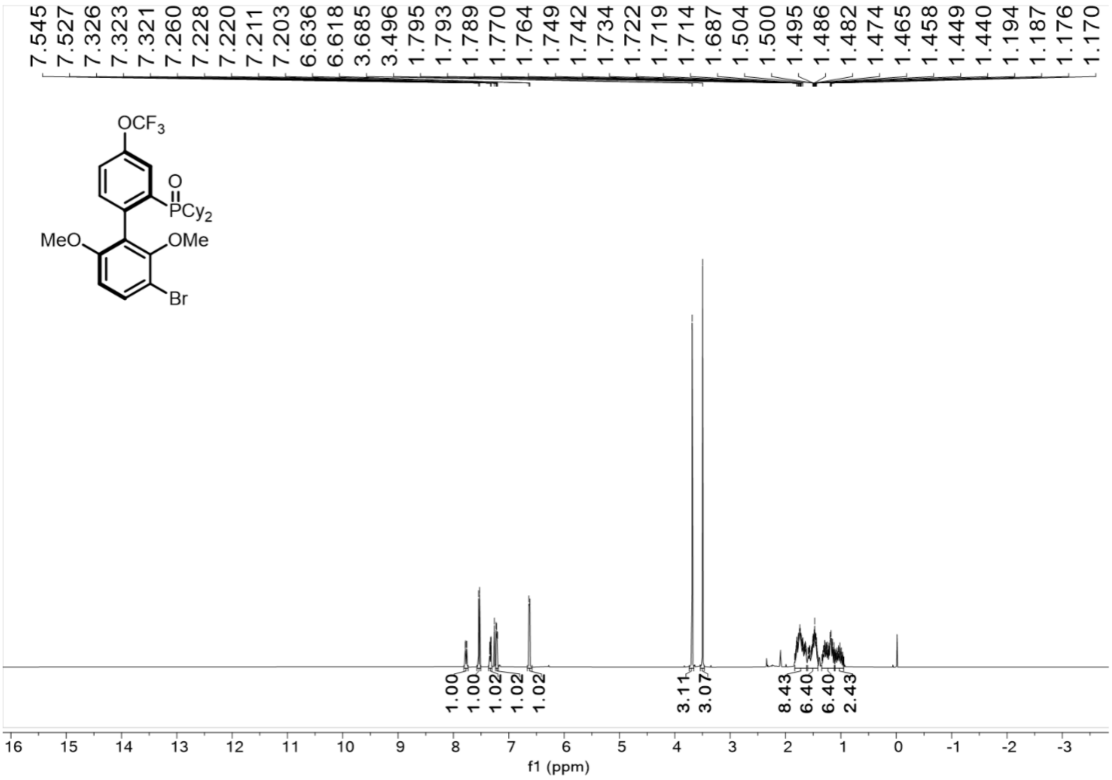

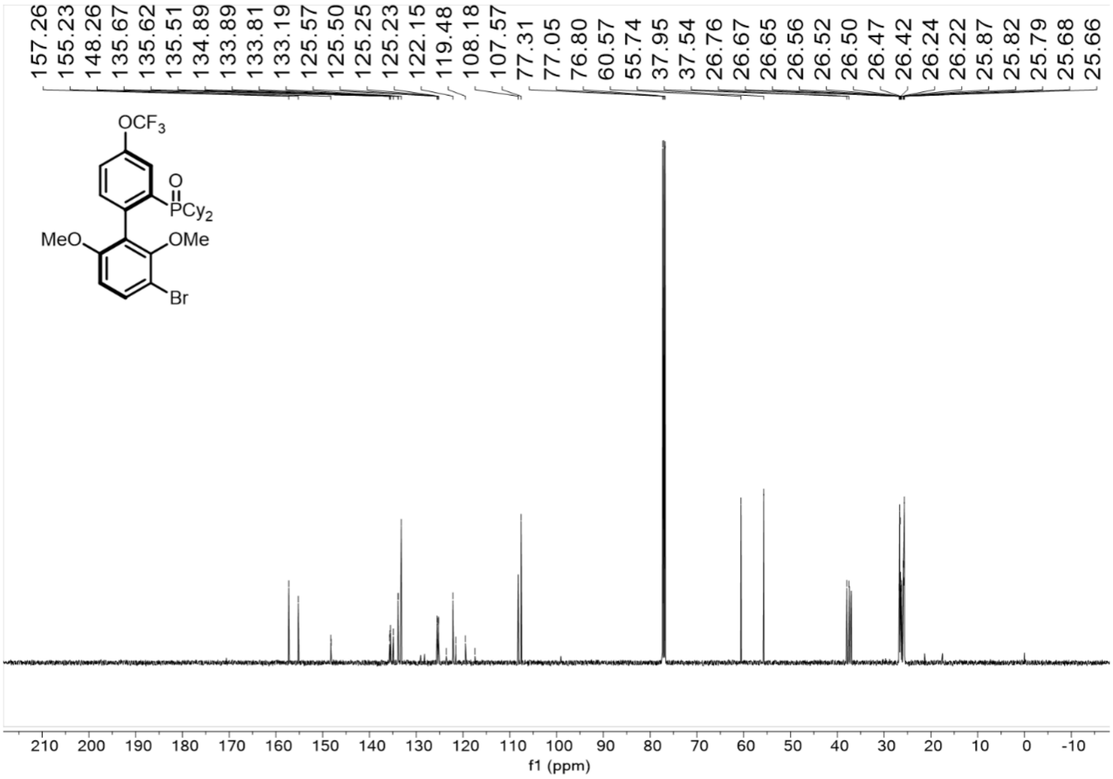

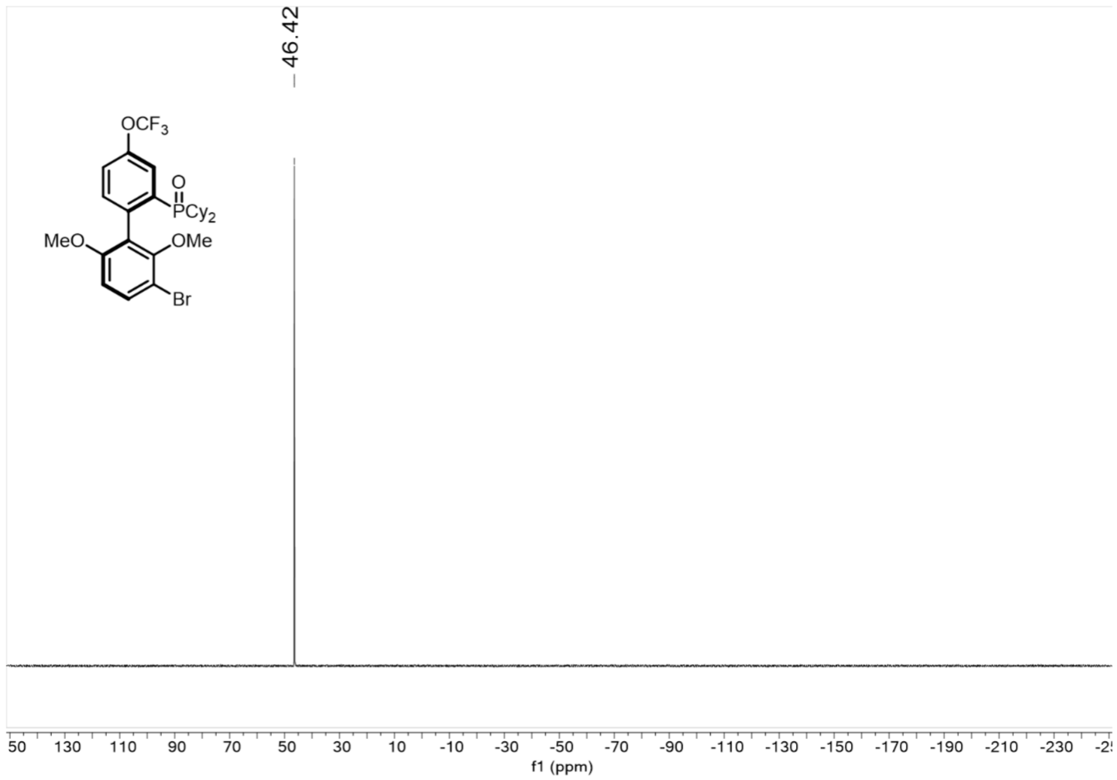

46.42
OCF3
O
PCy2
MeO
OMe
Br
50
130
110
90
70
50
30
10
-10
-30
-50
-70
-90
-110
-130
-150
-170
-190
-210
-230
f1 (ppm)

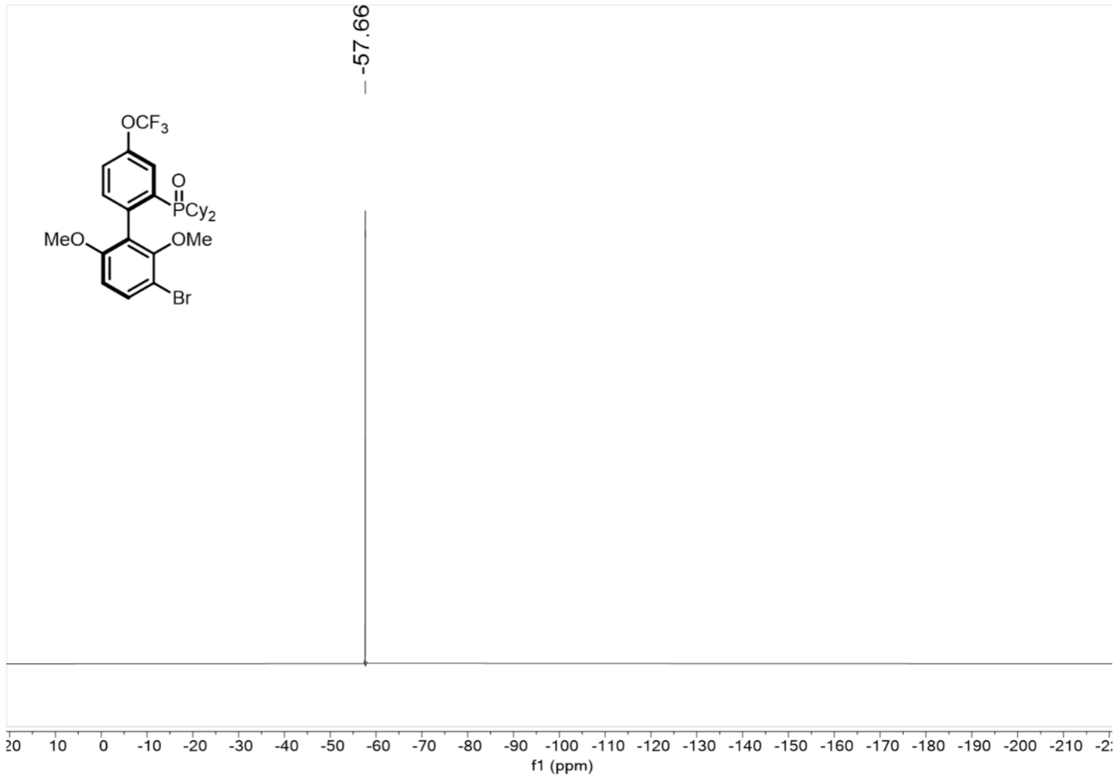

-57.66
OCF3
O
PCy2
MeO
OMe
Br
20
10
0
-10
-20
-30
-40
-50
-60
-70
-80
-90
-100
-110
-120
-130
-140
-150
-160
-170
-180
-190
-200
-210
f1 (ppm)

(*R*)-(3'-bromo-4-fluoro-2',6'-dimethoxy-[1,1'-biphenyl]-2-yl)dicyclohexylphosphine oxide (24); **$CDCl_3$**

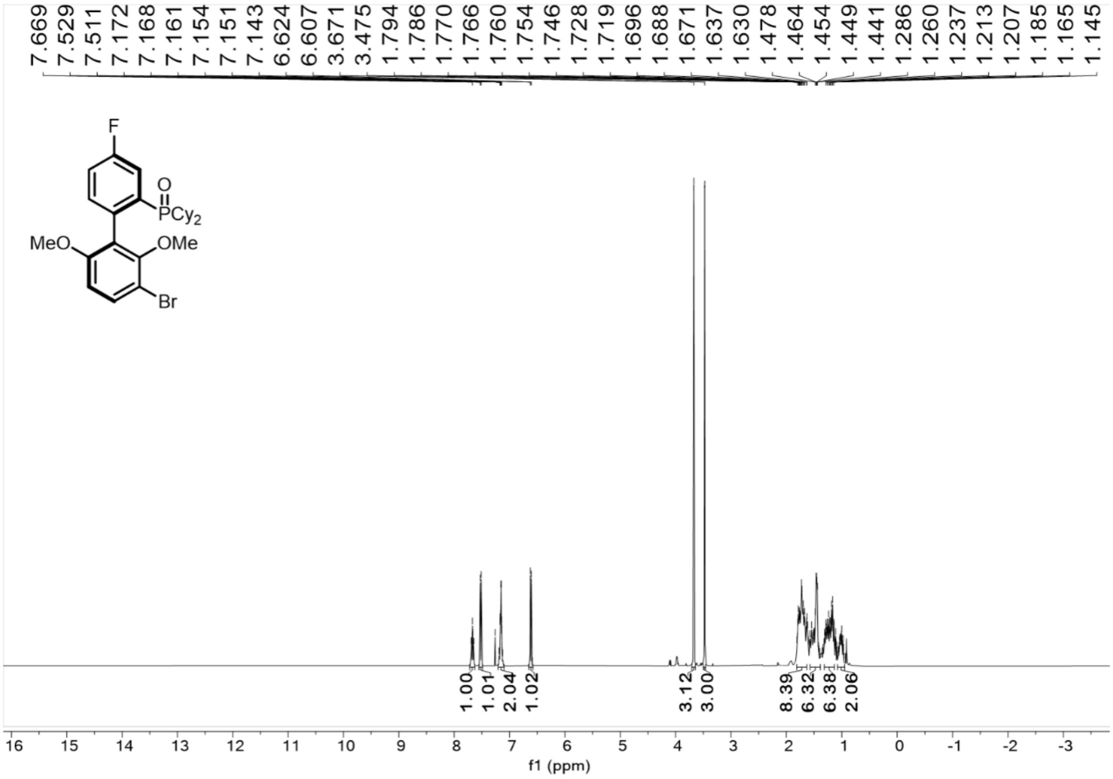

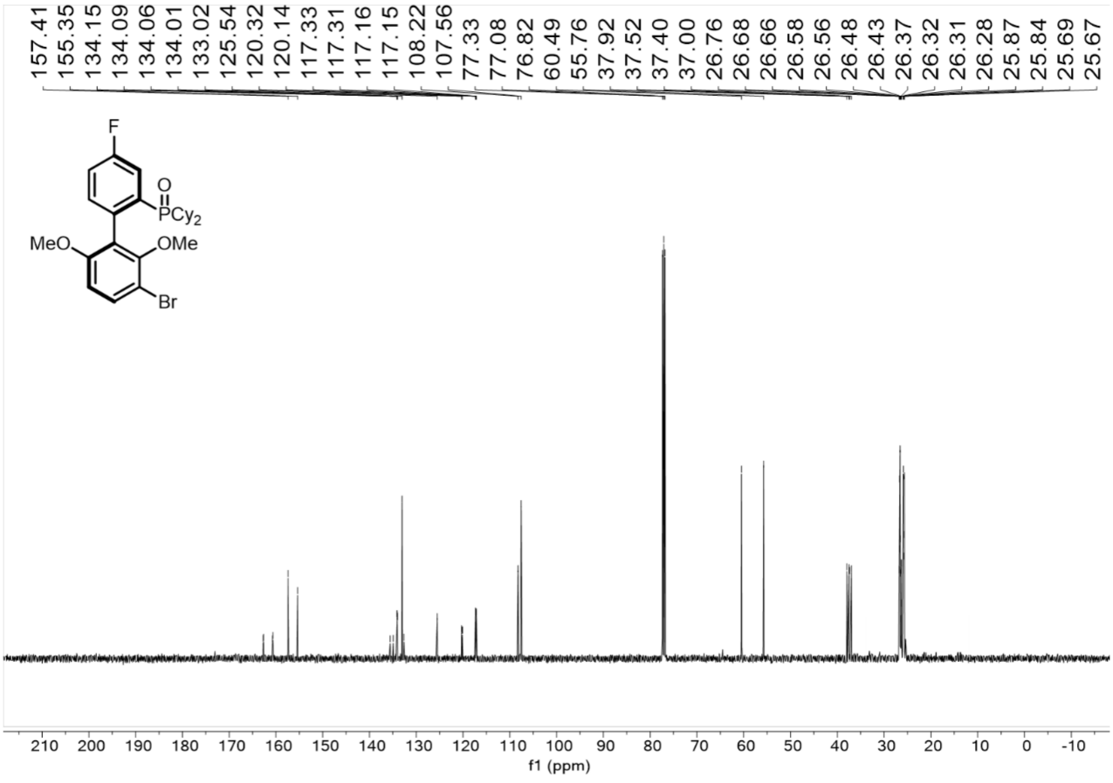

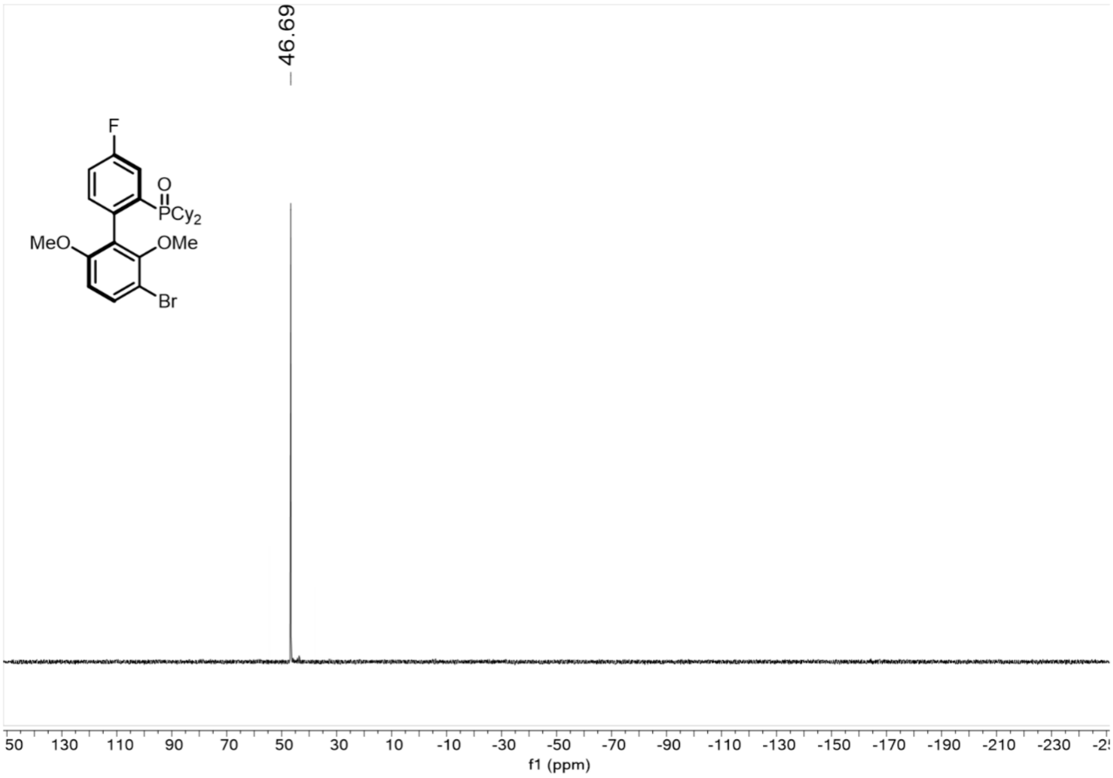

46.69
F
O
PCy2
MeO
OMe
Br
50
130
110
90
70
50
30
10
-10
-30
-50
-70
-90
-110
-130
-150
-170
-190
-210
-230
f1 (ppm)

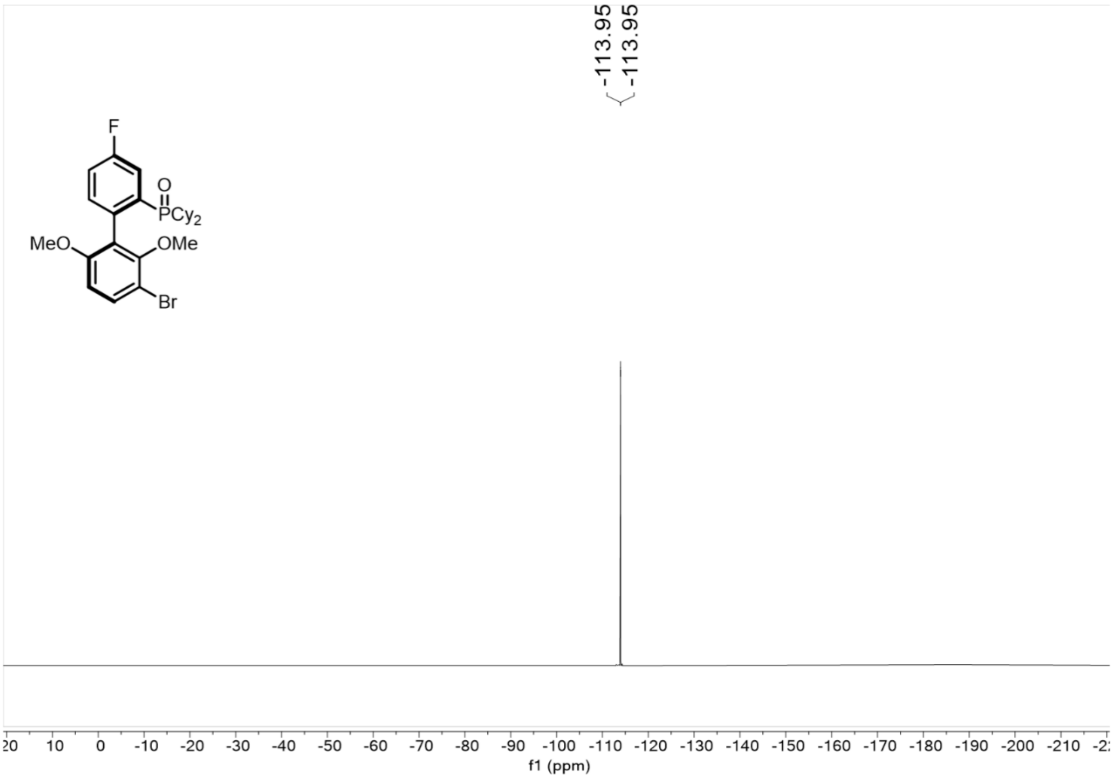

-113.95
-113.95
F
O
PCy2
MeO
OMe
Br
20
10
0
-10
-20
-30
-40
-50
-60
-70
-80
-90
-100
-110
-120
-130
-140
-150
-160
-170
-180
-190
-200
-210
f1 (ppm)

(*R*)-(3'-bromo-4-chloro-2',6'-dimethoxy-[1,1'-biphenyl]-2-yl)dicyclohexylphosphine oxide (25); $CDCl_3$

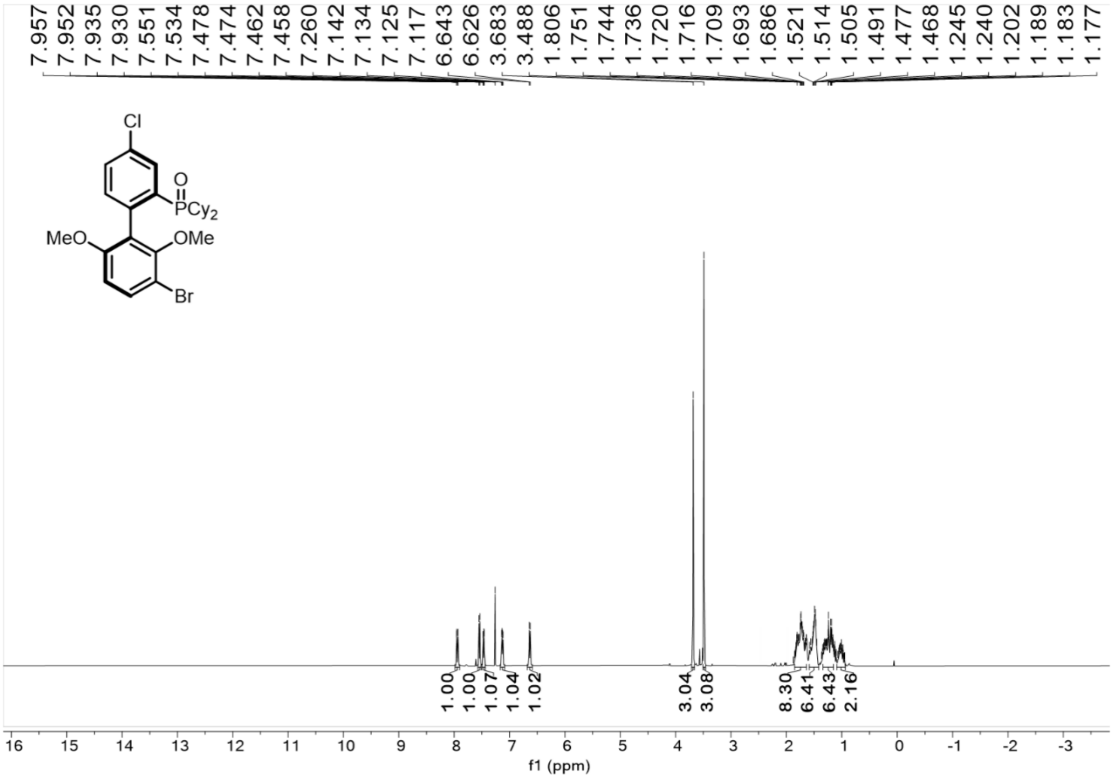

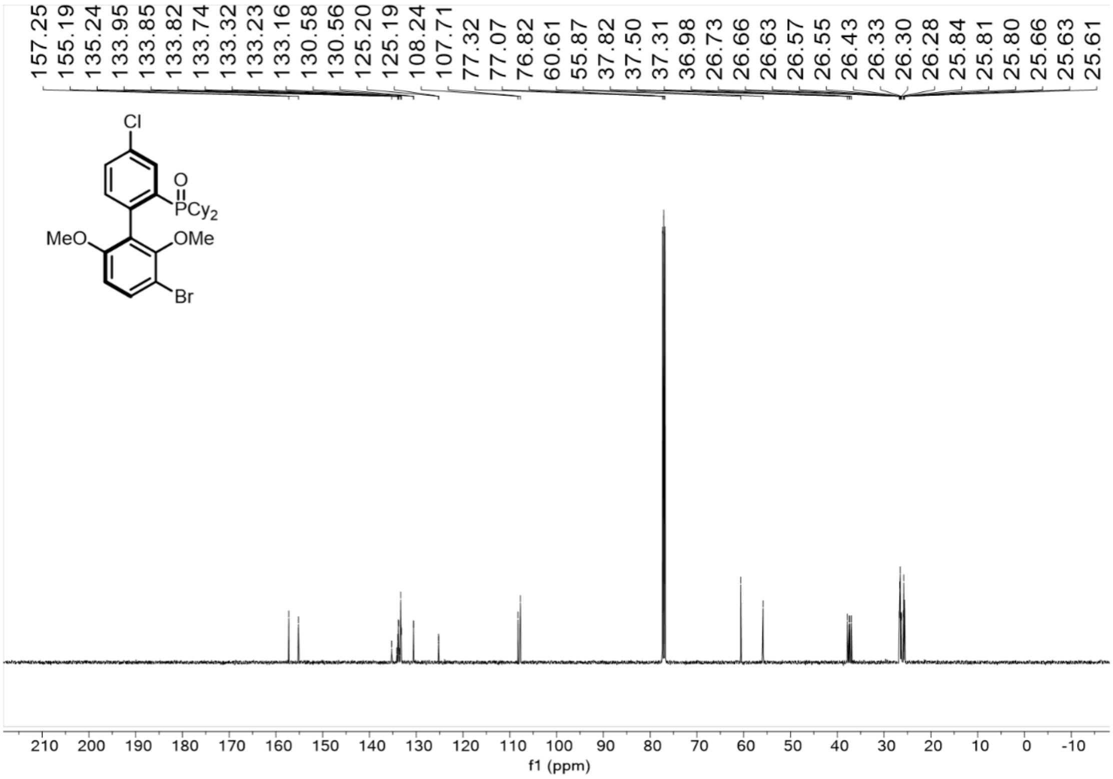

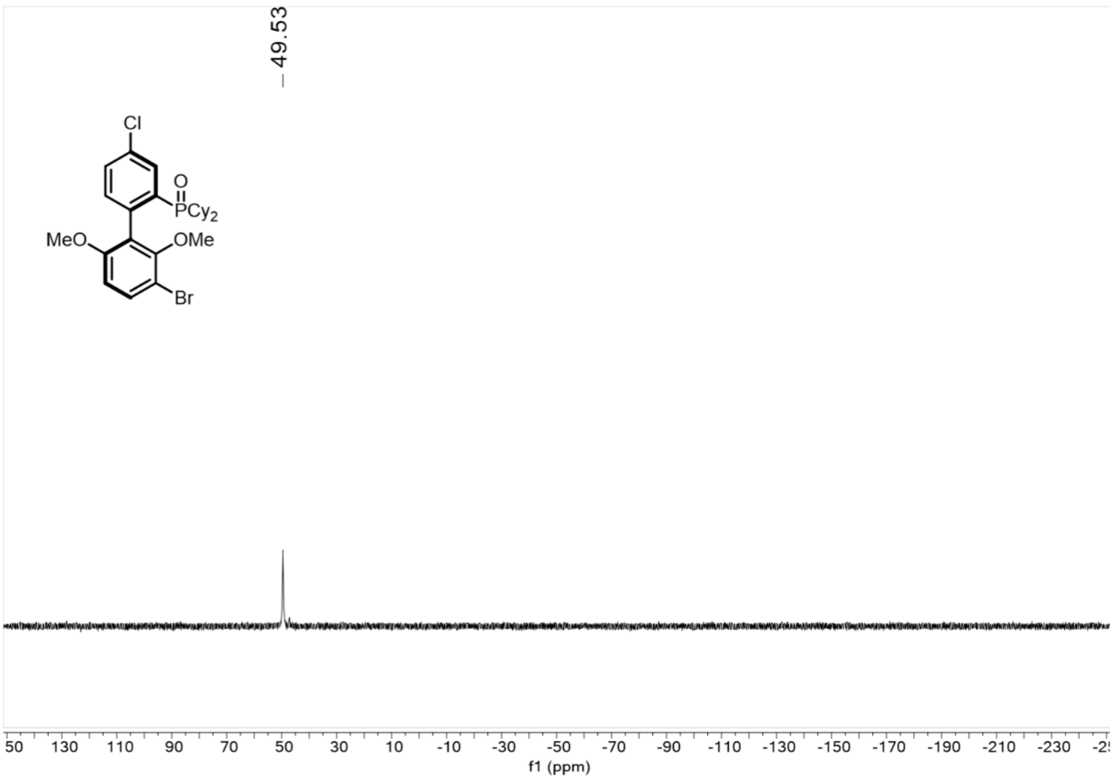

49.53
Cl
O
PCy2
MeO
OMe
Br
50
130
110
90
70
50
30
10
-10
-30
-50
-70
-90
-110
-130
-150
-170
-190
-210
-230
f1 (ppm)

(*R*)-3'-bromo-2-(dicyclohexylphosphoryl)-2',6'-dimethoxy-[1,1'-biphenyl]-4-carbonitrile (26); $CDCl_3$

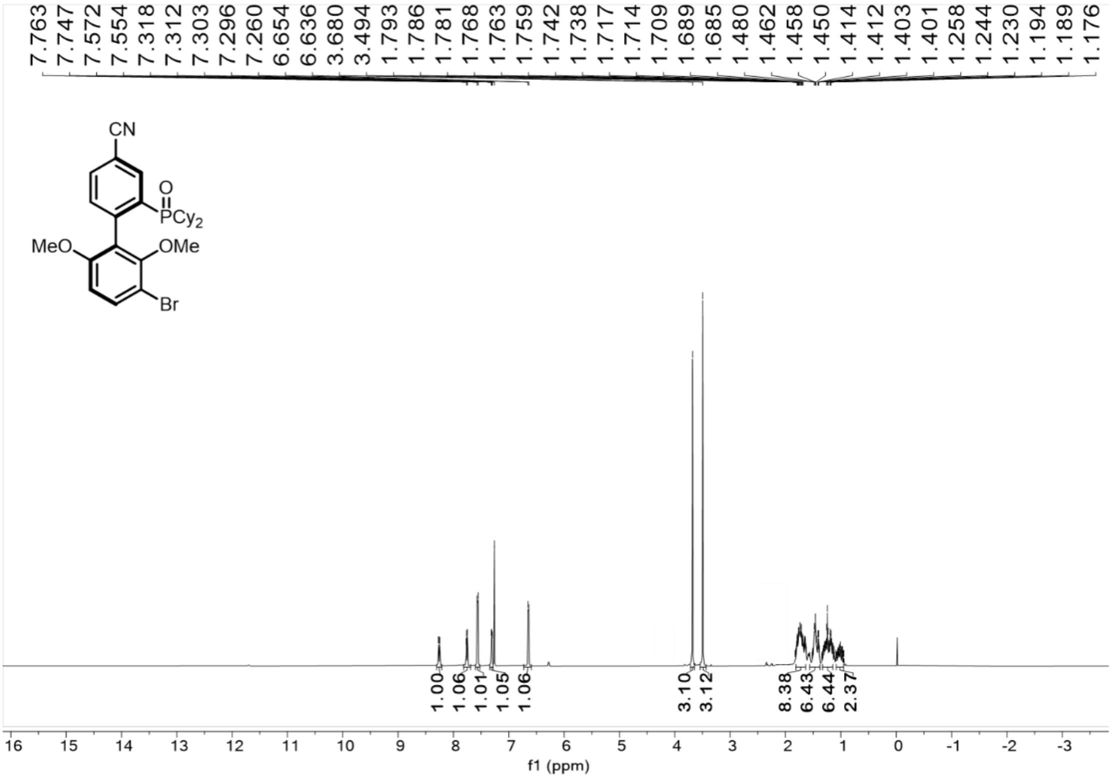

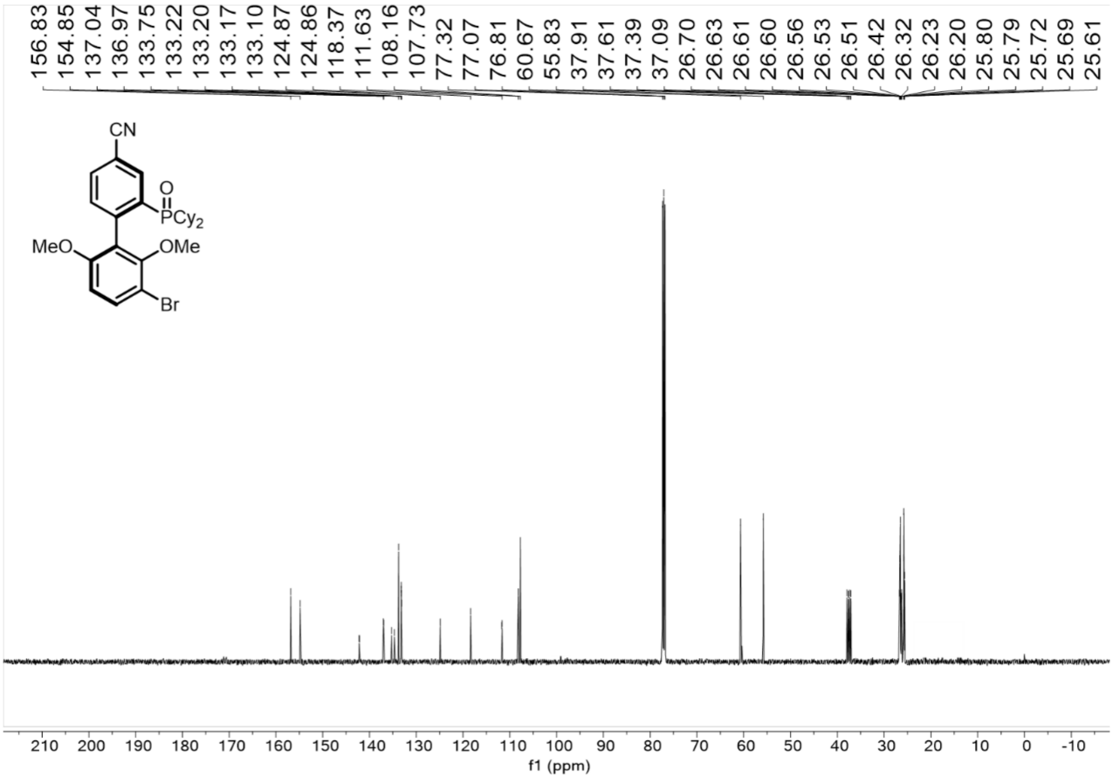

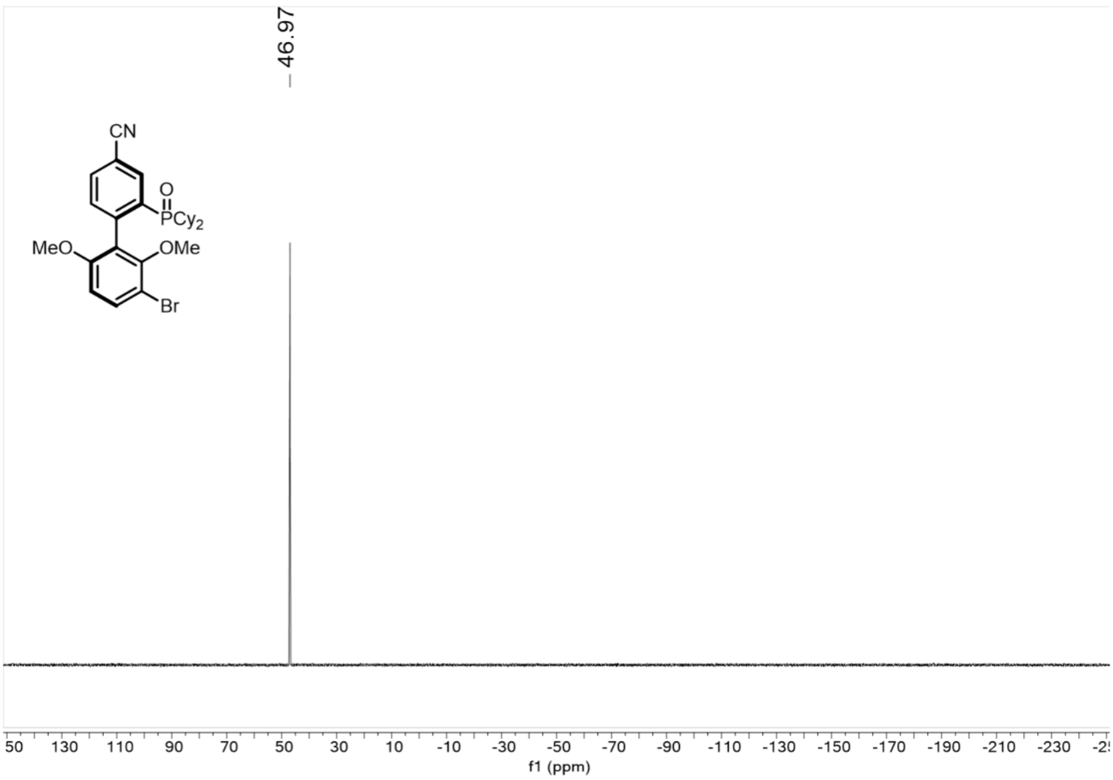
46.97
CN
O
PCy2
MeO
OMe
Br
50
130
110
90
70
50
30
10
-10
-30
-50
-70
-90
-110
-130
-150
-170
-190
-210
-230
f1 (ppm)

(*R*)-(3'-bromo-2',6'-bis(methoxymethoxy)-[1,1'-biphenyl]-2-yl)dicyclohexylphosphine oxide (27); $CDCl_3$

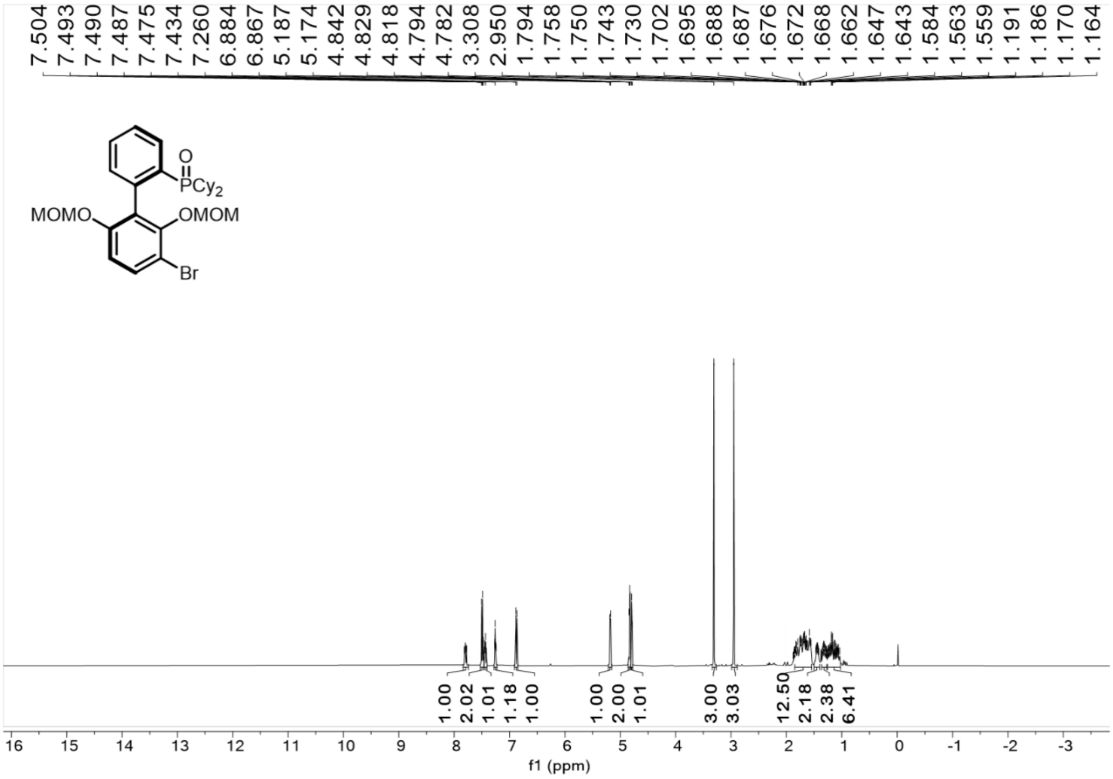

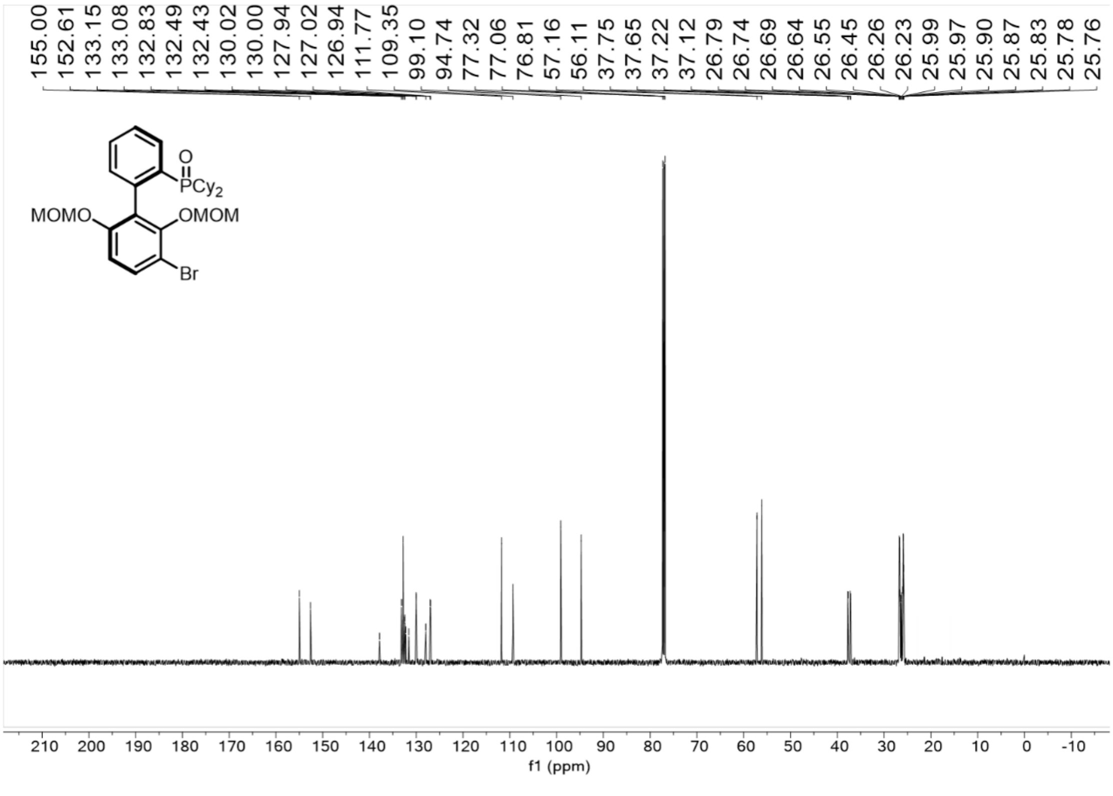

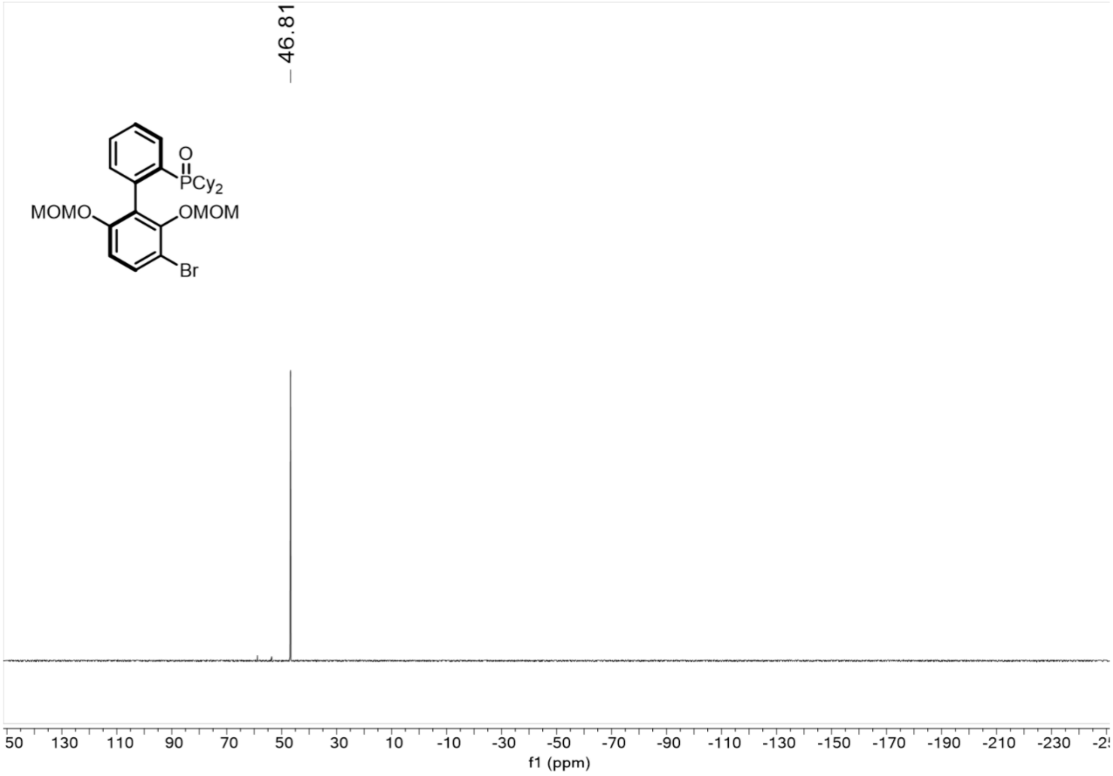

46.81
MOMO
OMOM
PCy2
O
Br
50 130 110 90 70 50 30 10 -10 -30 -50 -70 -90 -110 -130 -150 -170 -190 -210 -230
f1 (ppm)

(*R*)-(3'-bromo-2',6'-diethoxy-[1,1'-biphenyl]-2-yl)dicyclohexylphosphine oxide (28); $CDCl_3$

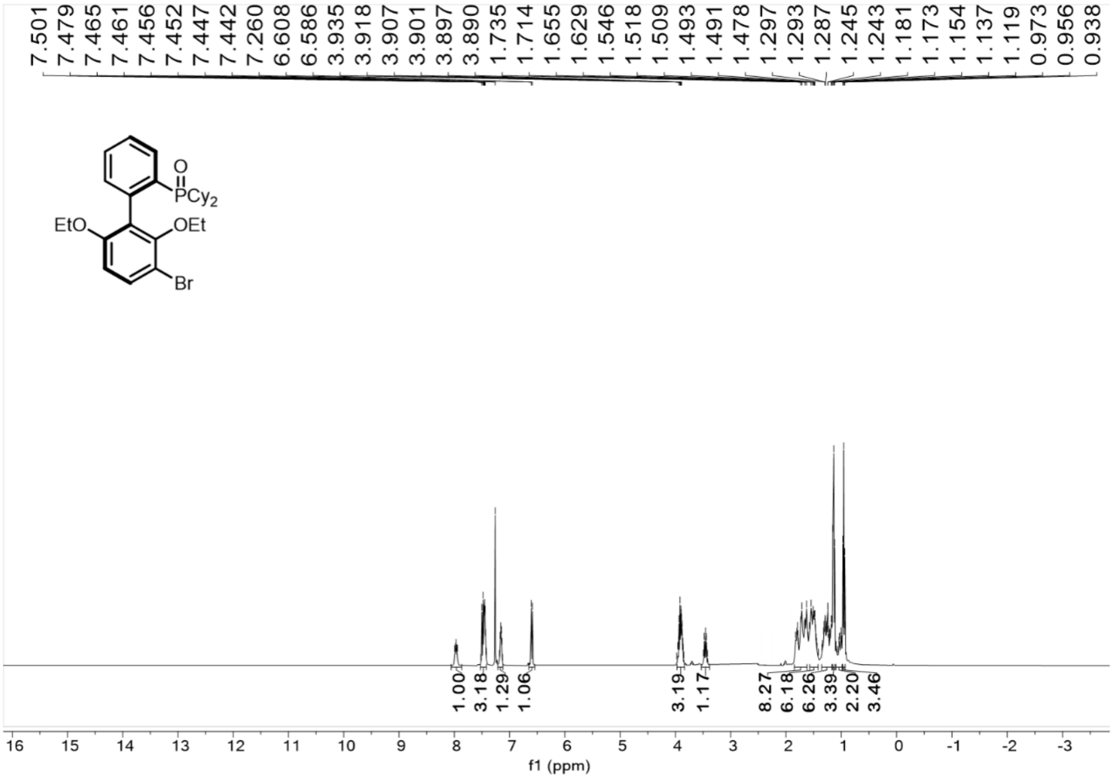

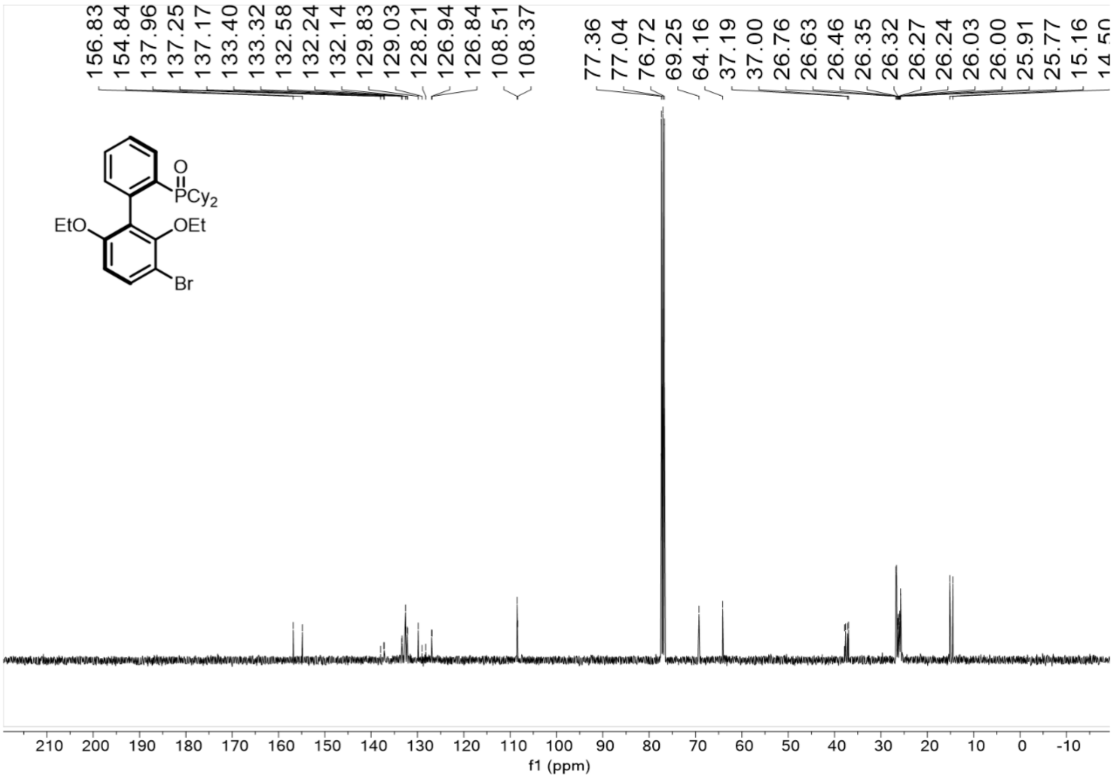

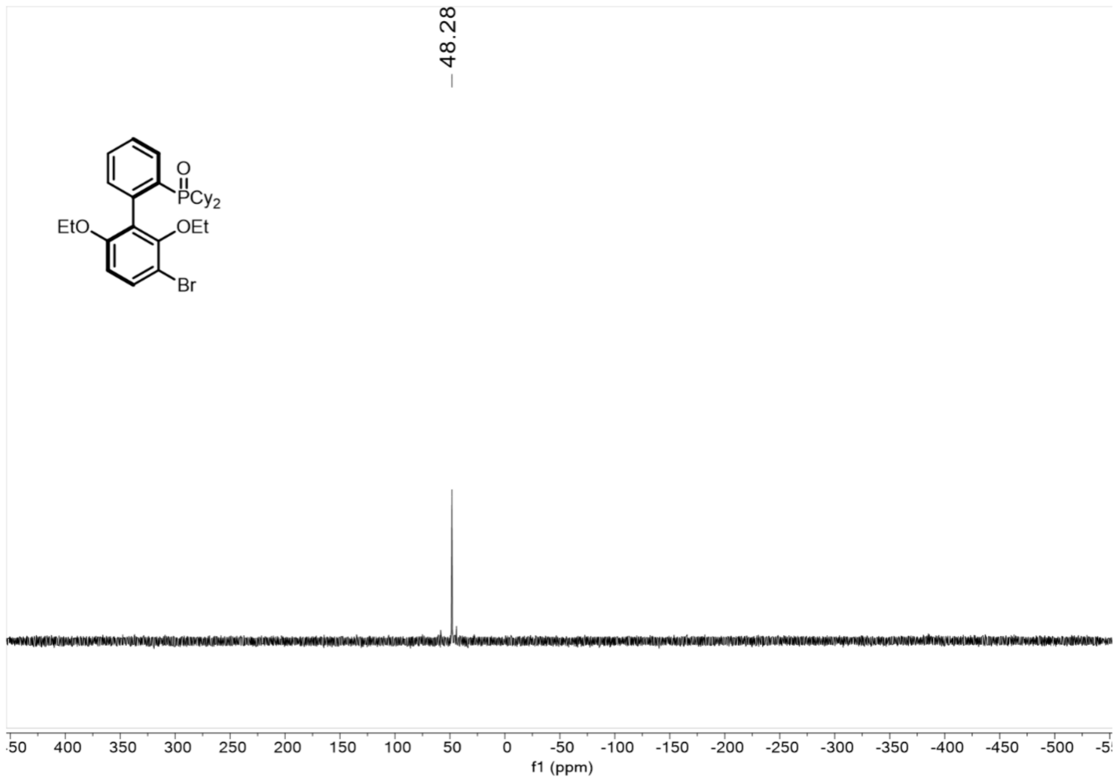

48.28
O
PCy2
EtO
OEt
Br
f1 (ppm)

(*R*)-(3'-bromo-2',6'-dipropoxy-[1,1'-biphenyl]-2-yl)dicyclohexylphosphine oxide (29); CDCl₃

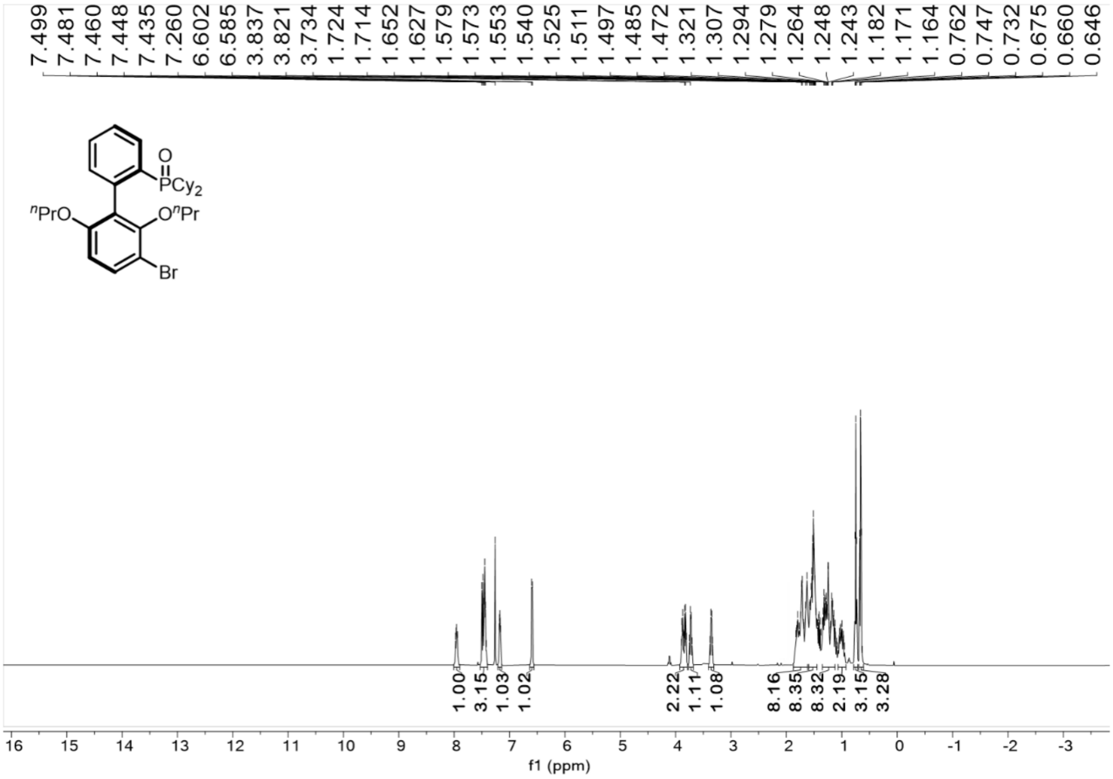

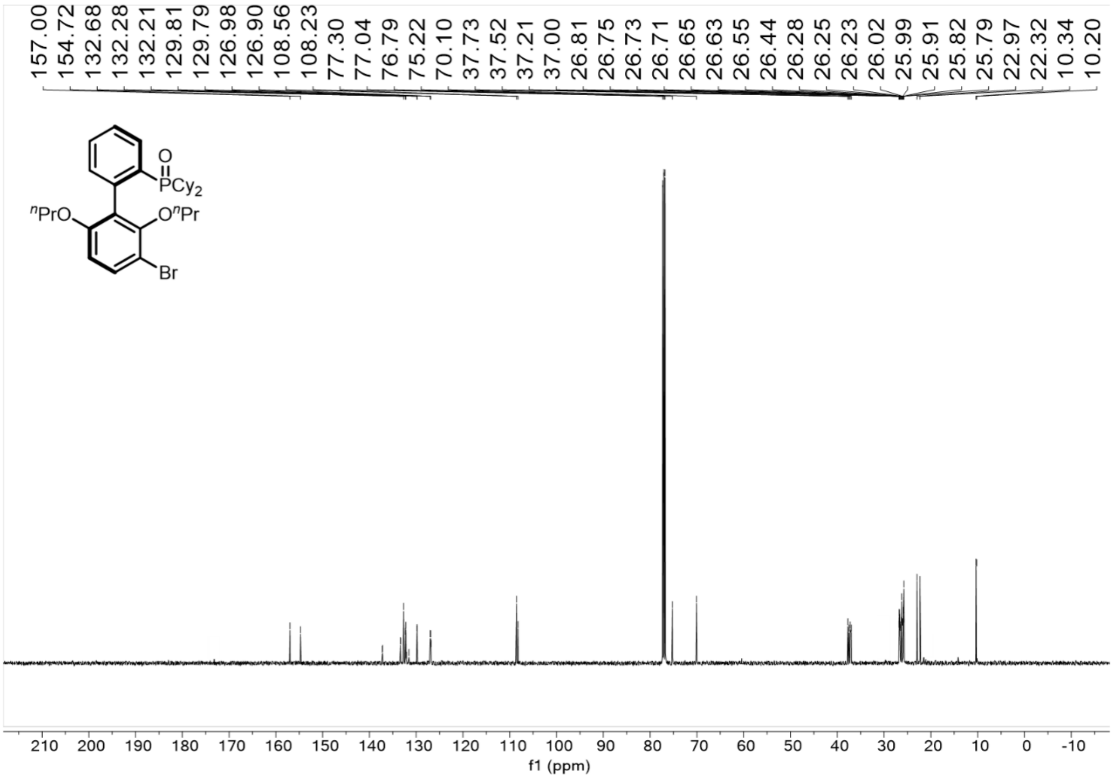

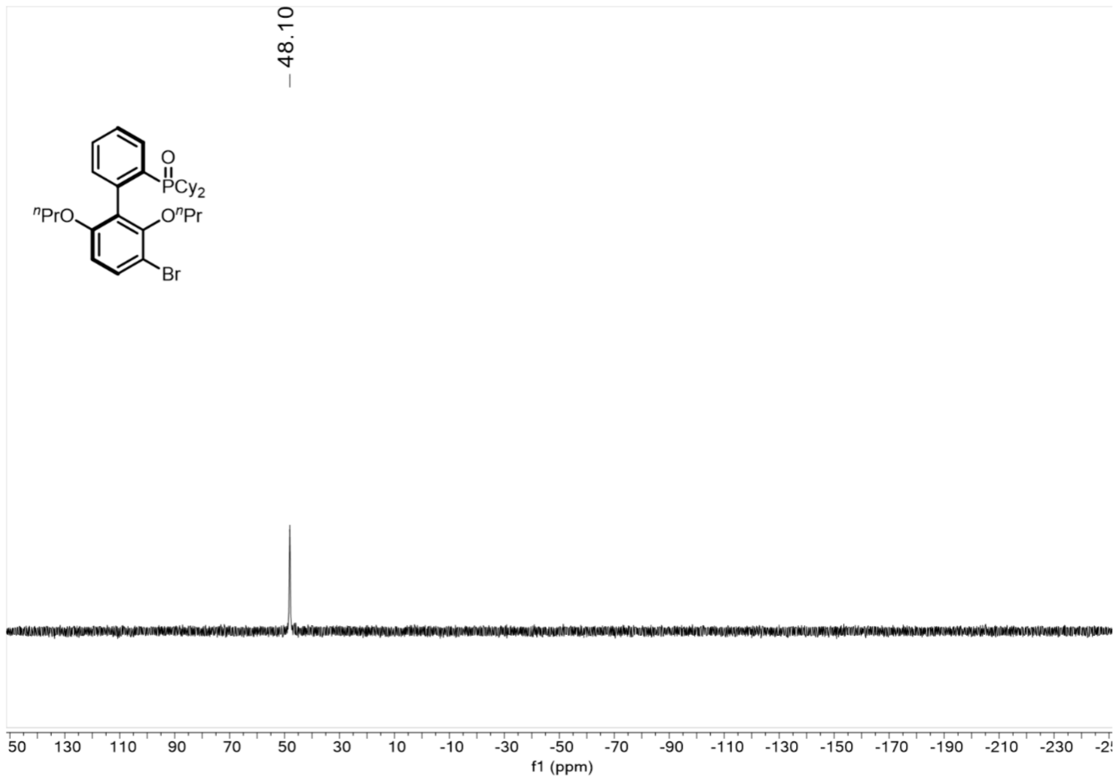

48.10
O
PCy2
ⁿPrO
OⁿPr
Br
50
130
110
90
70
50
30
10
-10
-30
-50
-70
-90
-110
-130
-150
-170
-190
-210
-230
f1 (ppm)

(*R*)-(3'-bromo-2',6'-diisopropoxy-[1,1'-biphenyl]-2-yl)dicyclohexylphosphine oxide (30); $CDCl_3$

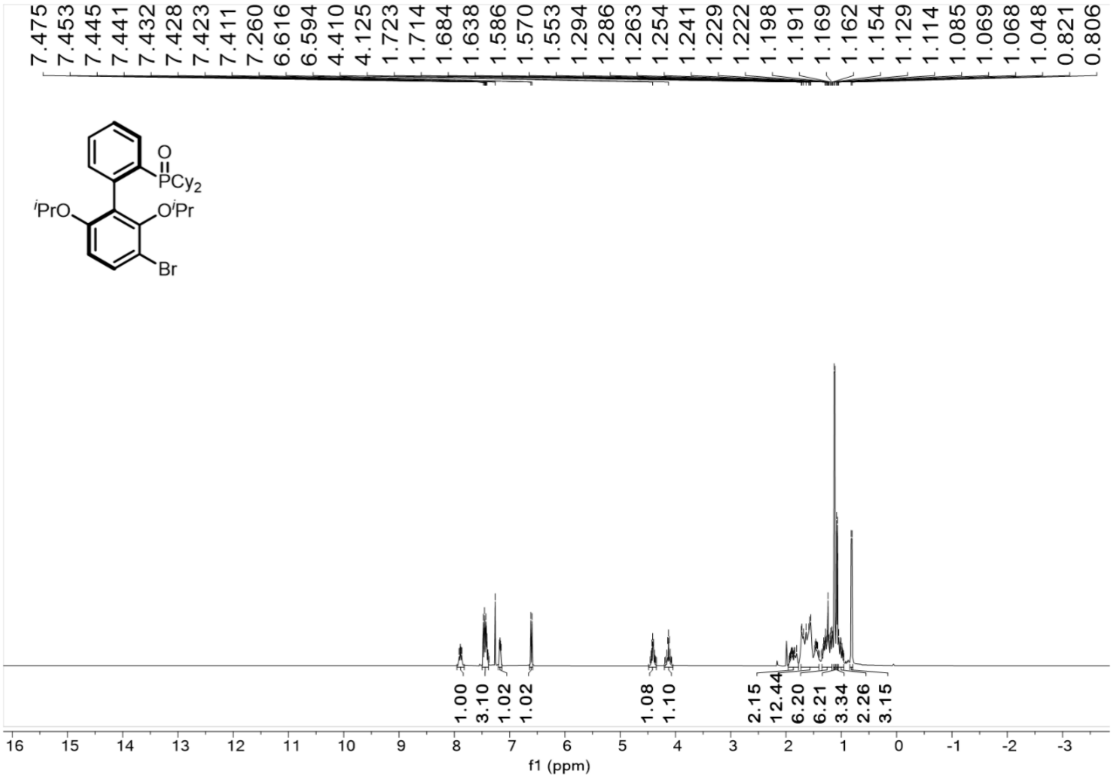

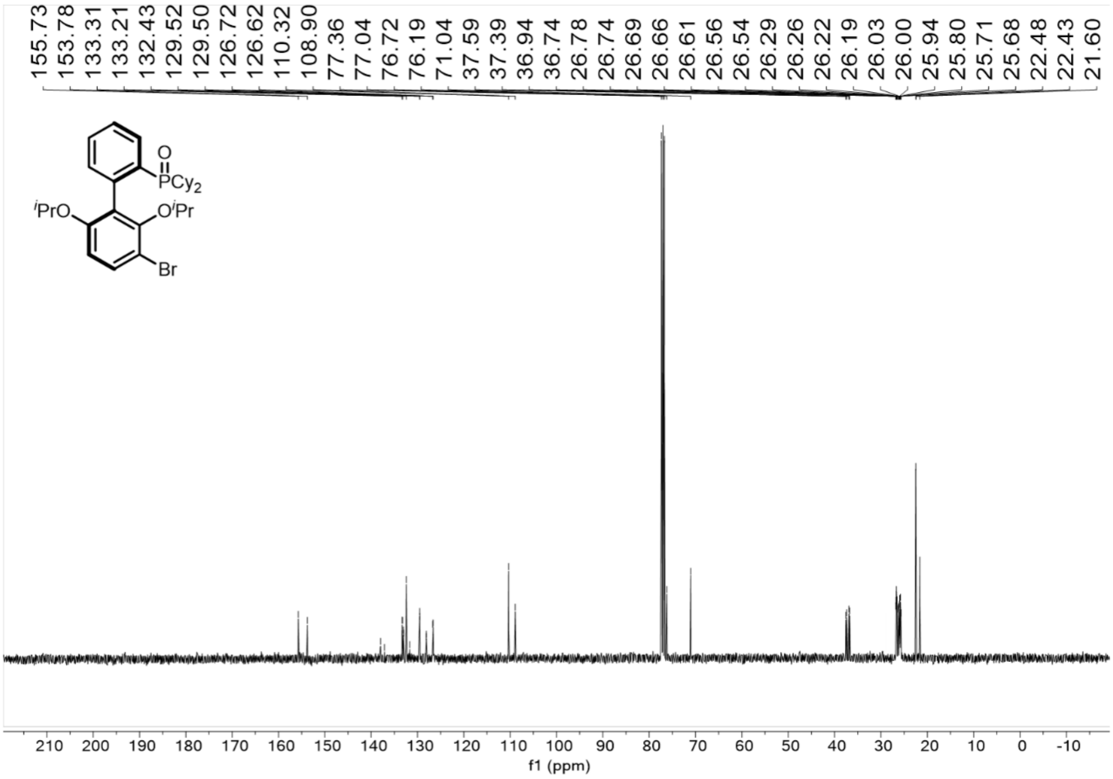

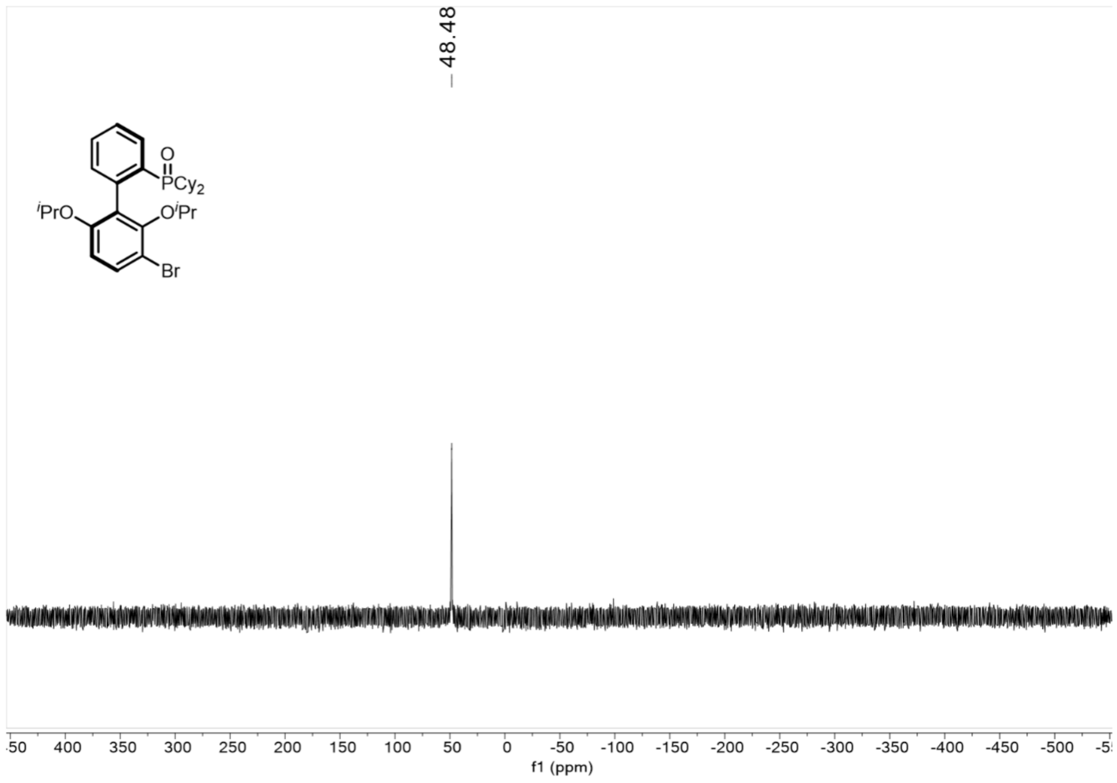
48.48
O
PCy2
iPrO
OiPr
Br
f1 (ppm)

(*R*)-(3'-bromo-2',6'-bis(hexyloxy)-[1,1'-biphenyl]-2-yl)dicyclohexylphosphine oxide (31); $CDCl_3$

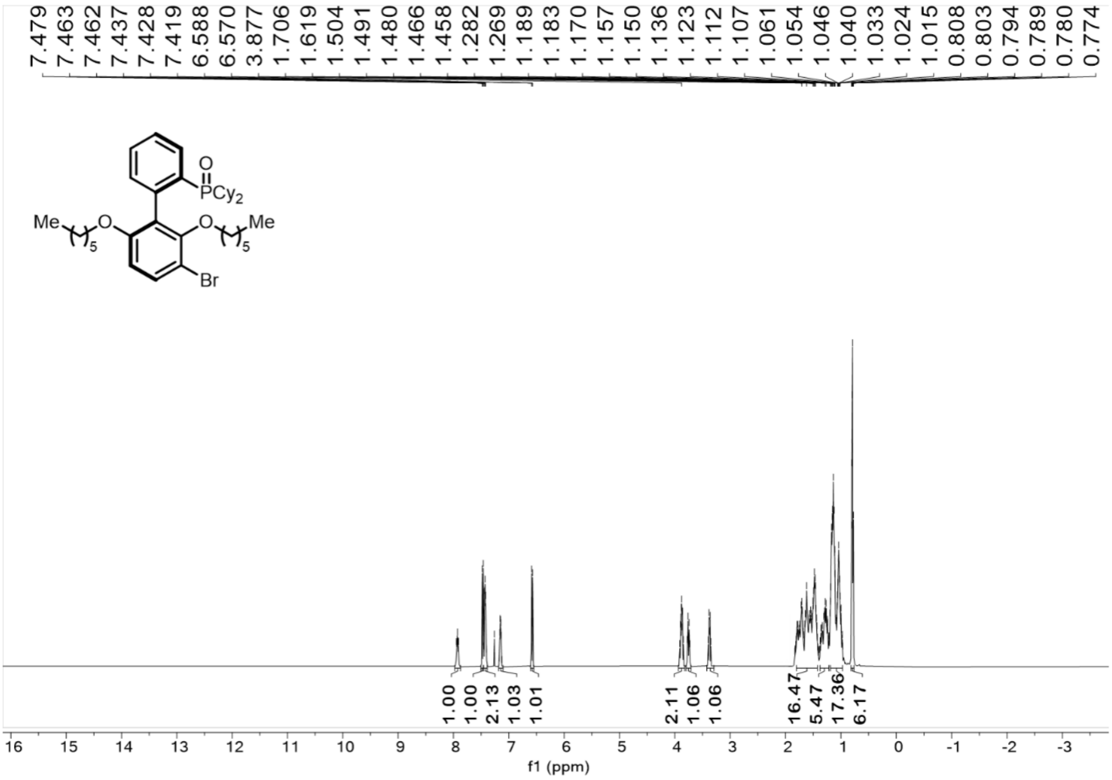

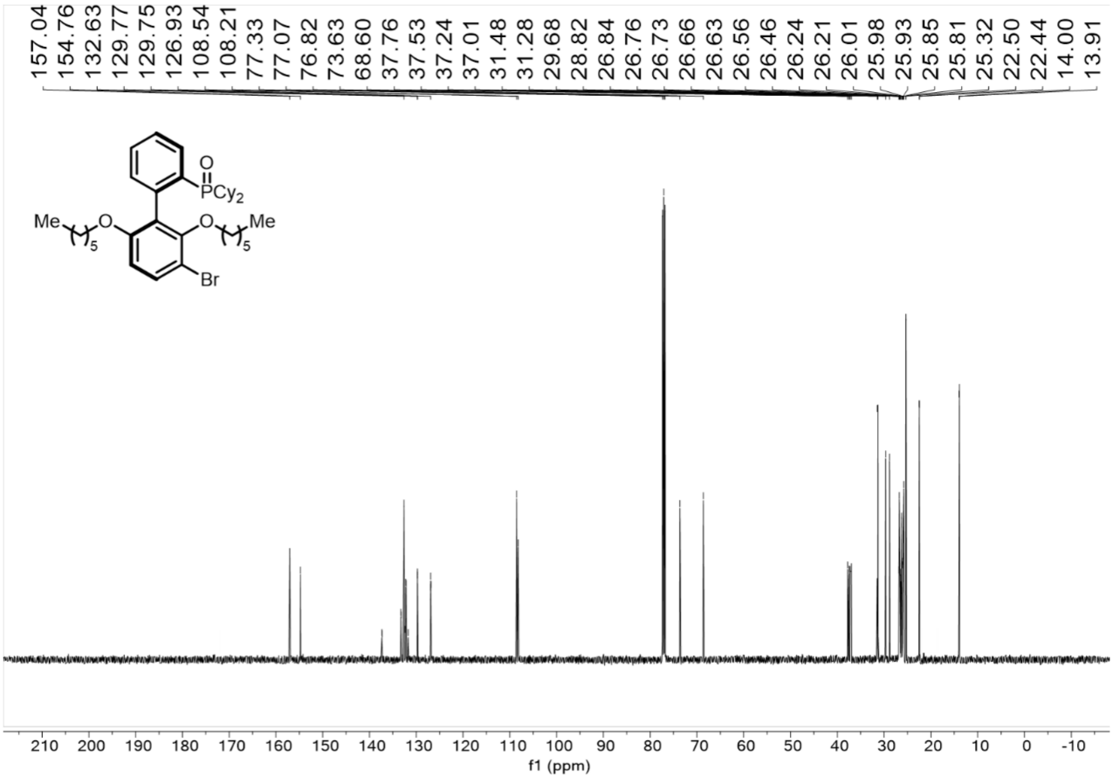

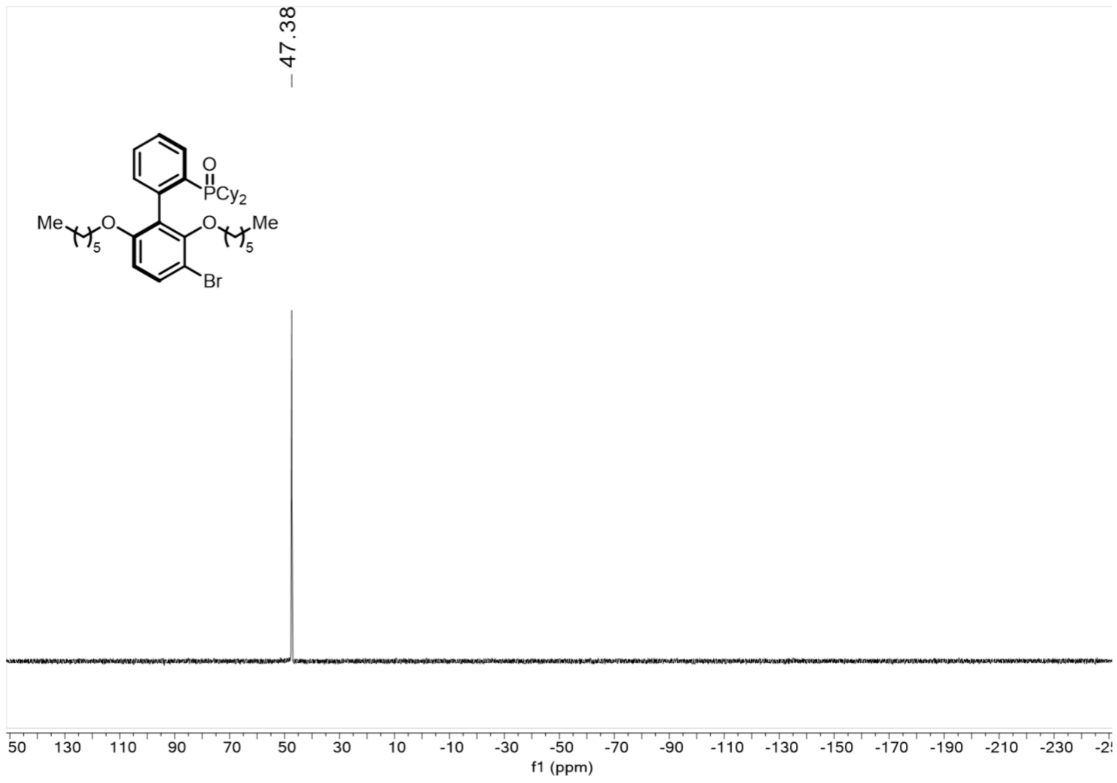
47.38
Me
O
O
Me
5
5
P
Cy2
Br
50
130
110
90
70
50
30
10
-10
-30
-50
-70
-90
-110
-130
-150
-170
-190
-210
-230
f1 (ppm)

(*R*)-(3'-bromo-2',6'-bis(dodecyloxy)-[1,1'-biphenyl]-2-yl)dicyclohexylphosphine oxide (32); $CDCl_3$

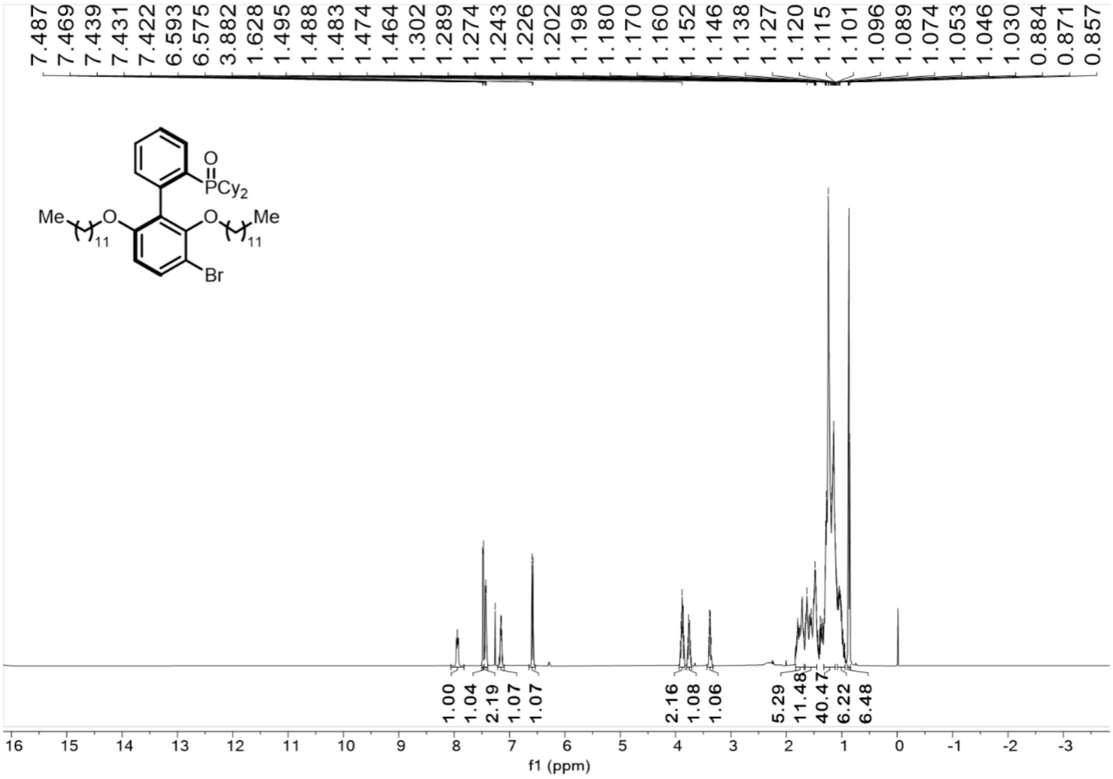

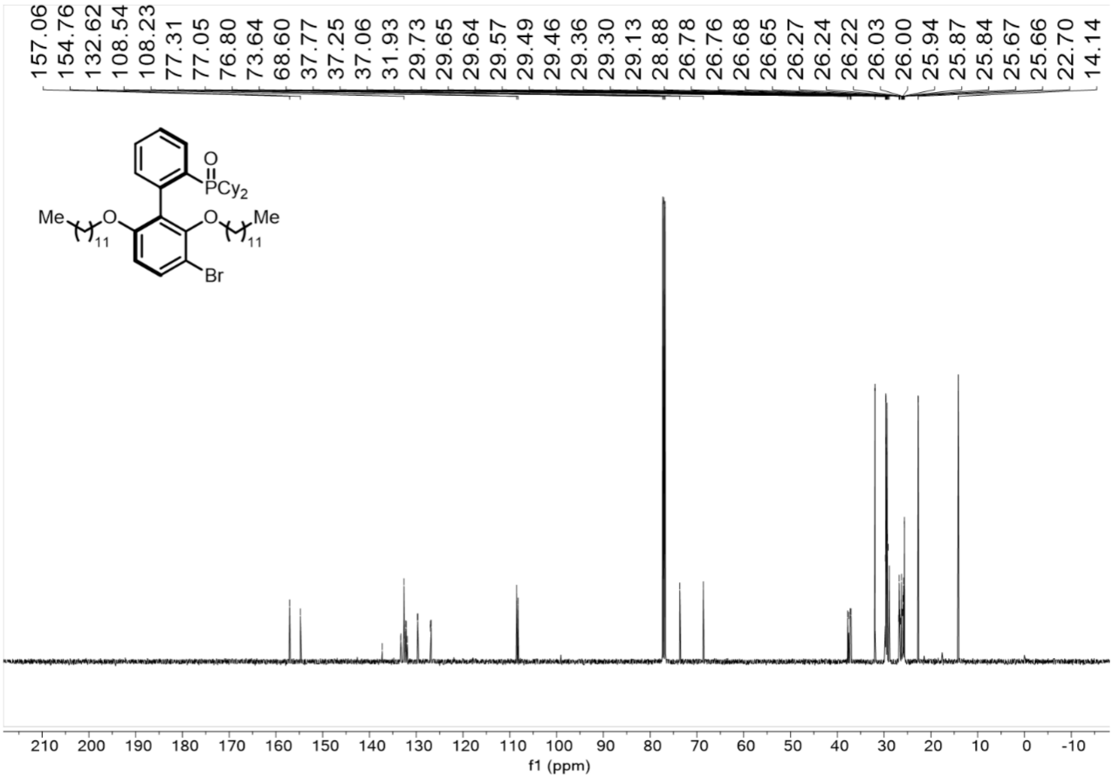

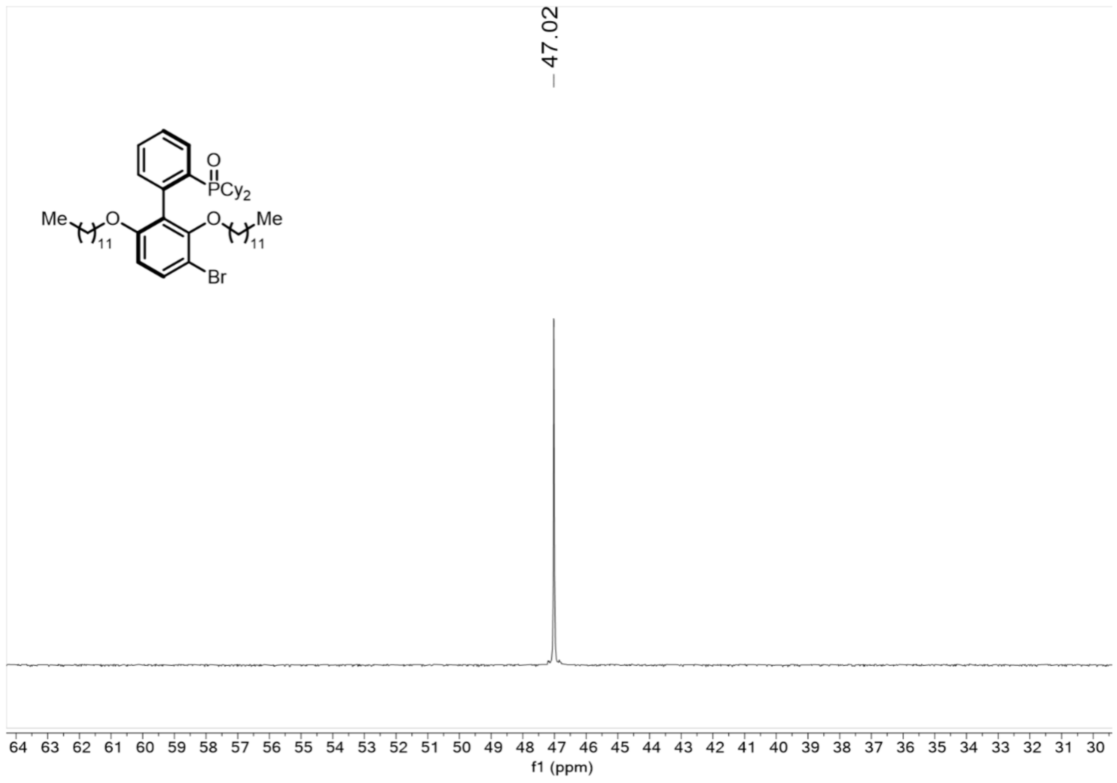

47.02
Me
O
11
O
Me
11
O
PCy2
Br
64 63 62 61 60 59 58 57 56 55 54 53 52 51 50 49 48 47 46 45 44 43 42 41 40 39 38 37 36 35 34 33 32 31 30
f1 (ppm)

(*R*)-(3'-bromo-2',6'-dimethoxy-4'-methyl-[1,1'-biphenyl]-2-yl)dicyclohexylphosphine oxide (33); $CDCl_3$

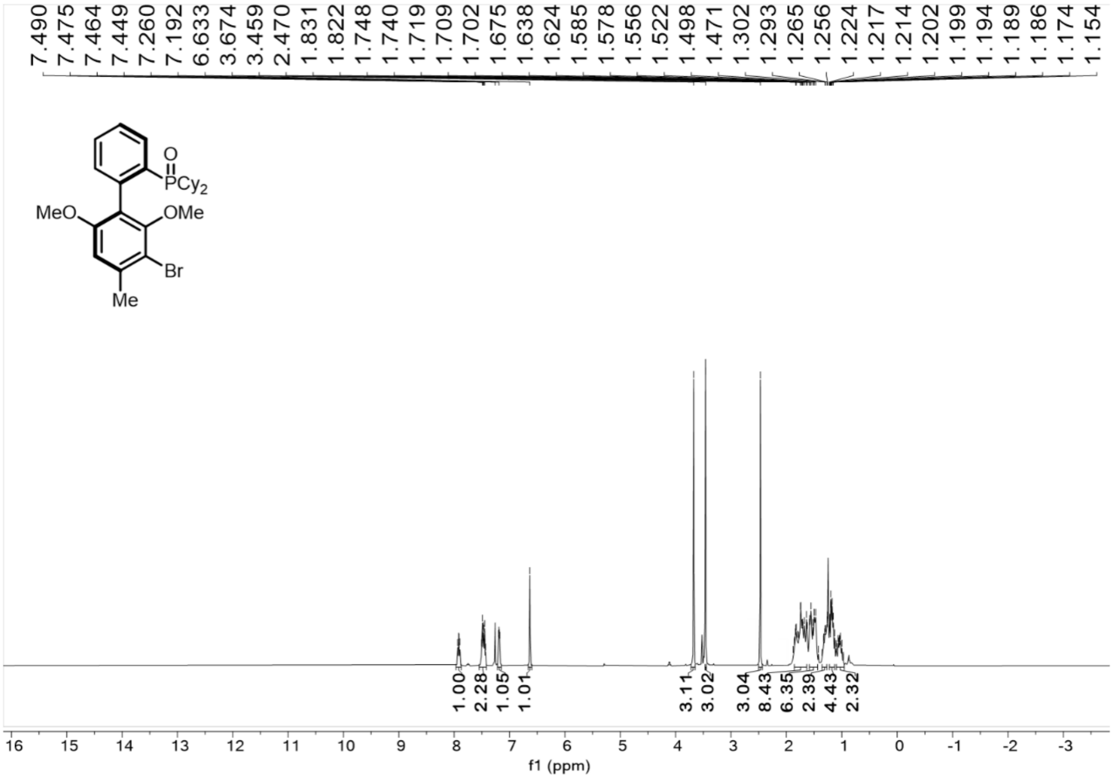

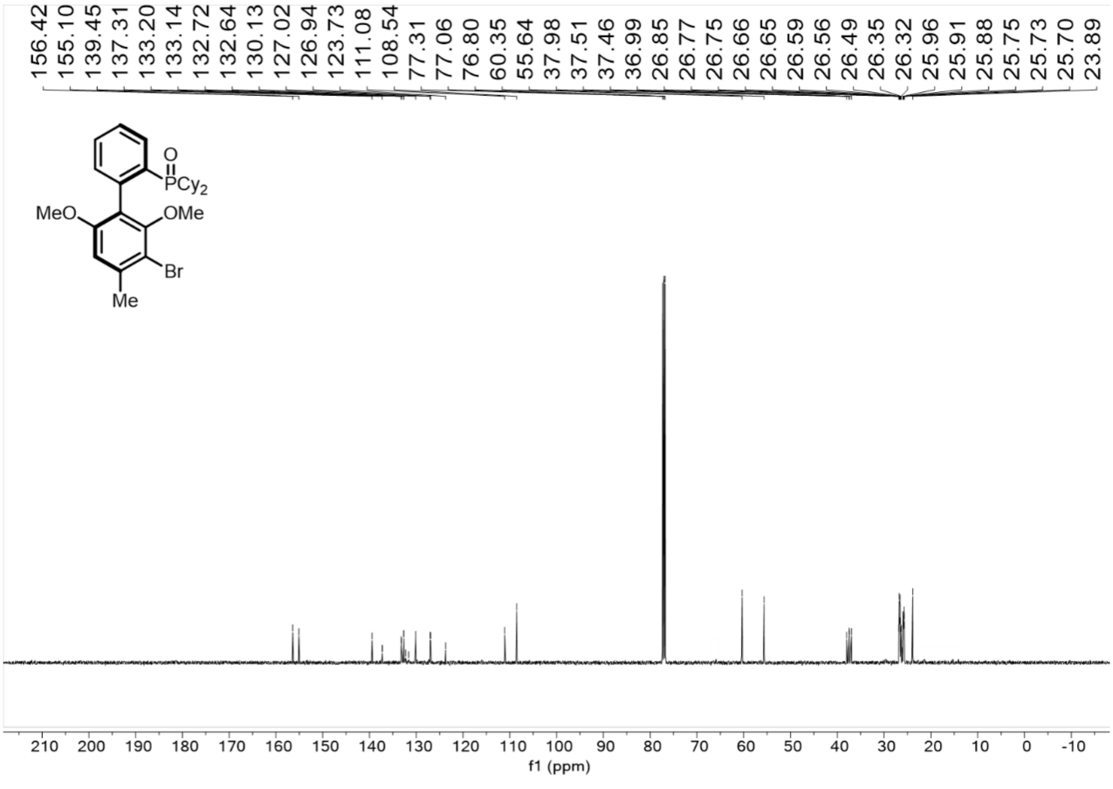

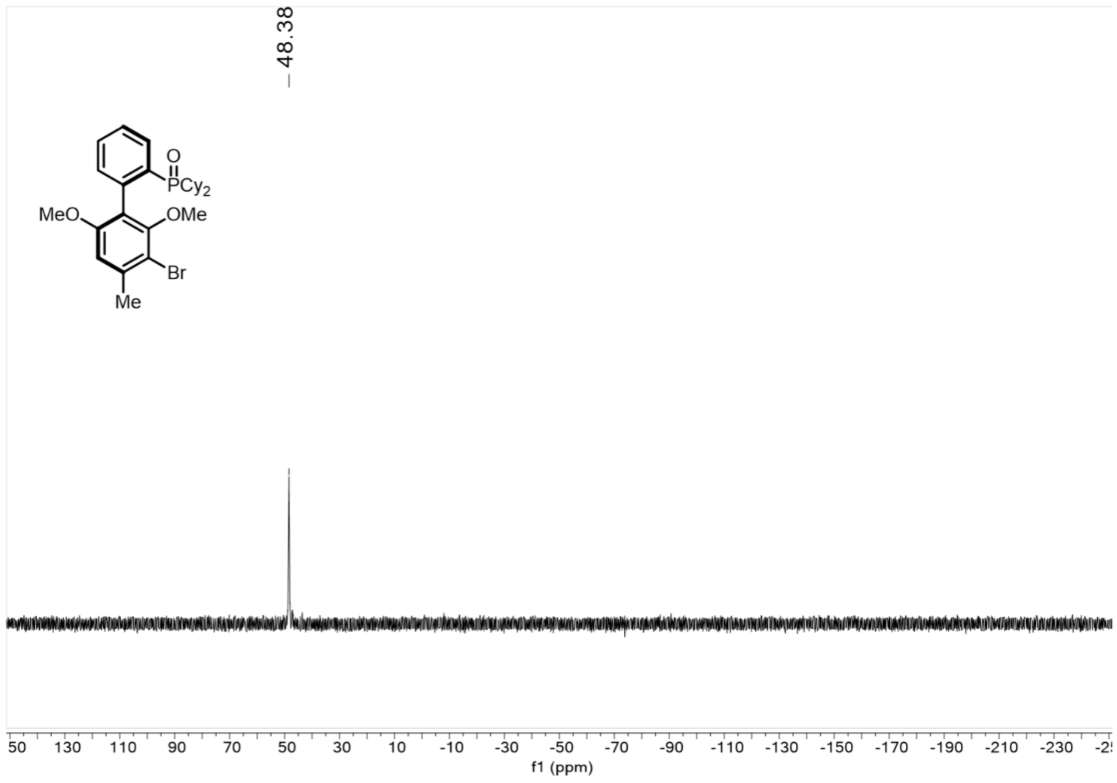
48.38
O
PCy2
MeO
OMe
Br
Me
50
130
110
90
70
50
30
10
-10
-30
-50
-70
-90
-110
-130
-150
-170
-190
-210
-230
f1 (ppm)

(*R*)-(3'-bromo-2',4',6'-trimethoxy-[1,1'-biphenyl]-2-yl)dicyclohexylphosphine oxide (34); $CDCl_3$

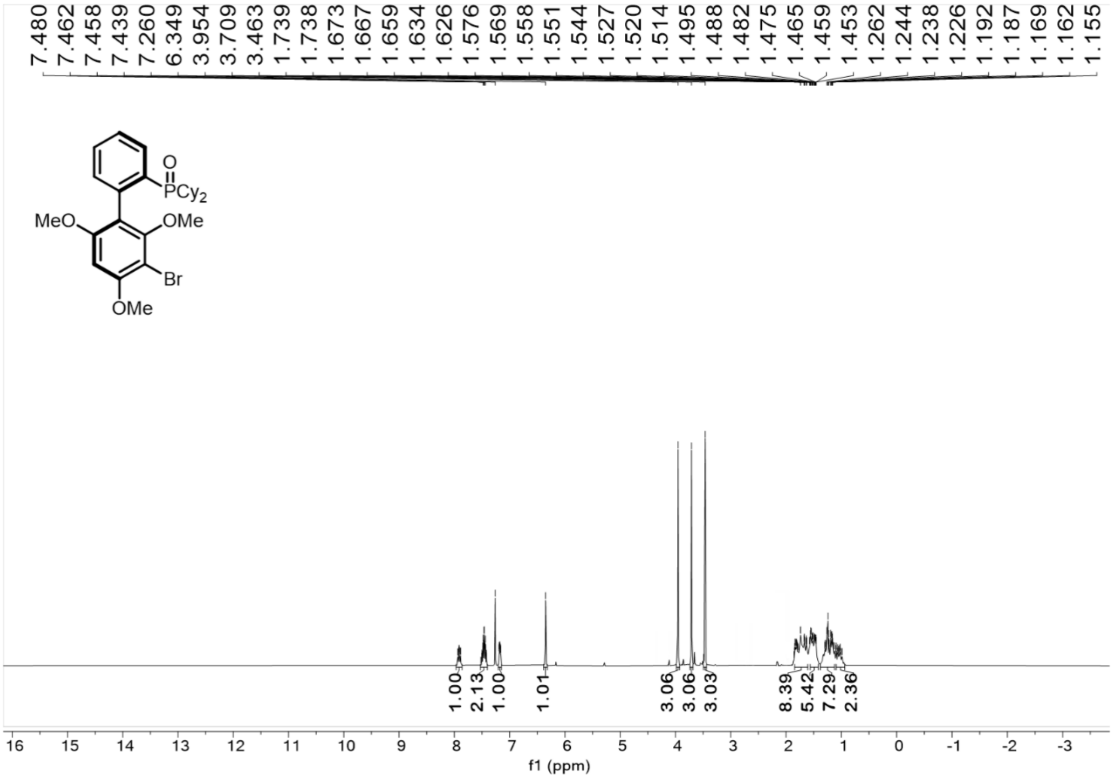

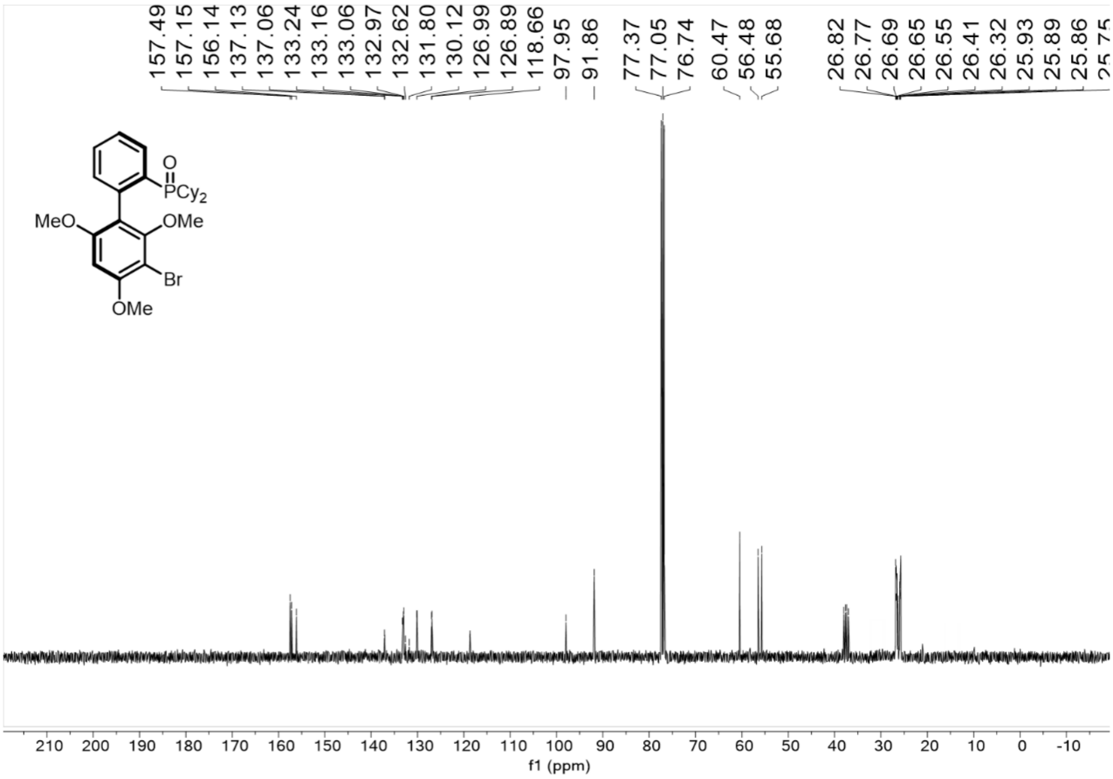

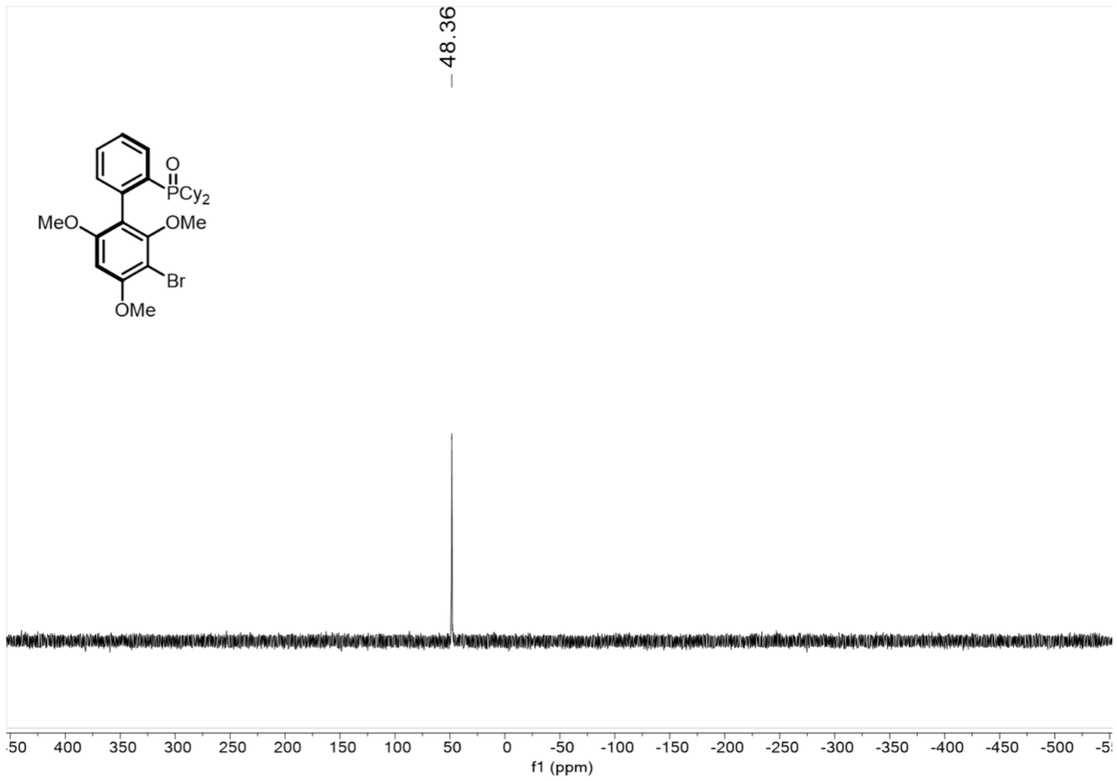

48.36
MeO
OMe
Br
OMe
PCy2
O
50
400
350
300
250
200
150
100
50
0
-50
-100
-150
-200
-250
-300
-350
-400
-450
-500
f1 (ppm)

(*R*)-(6-(3-bromo-2,6-dimethoxyphenyl)-2,3-dihydro-1H-inden-5-yl)dicyclohexylphosphine oxide (35); $CDCl_3$

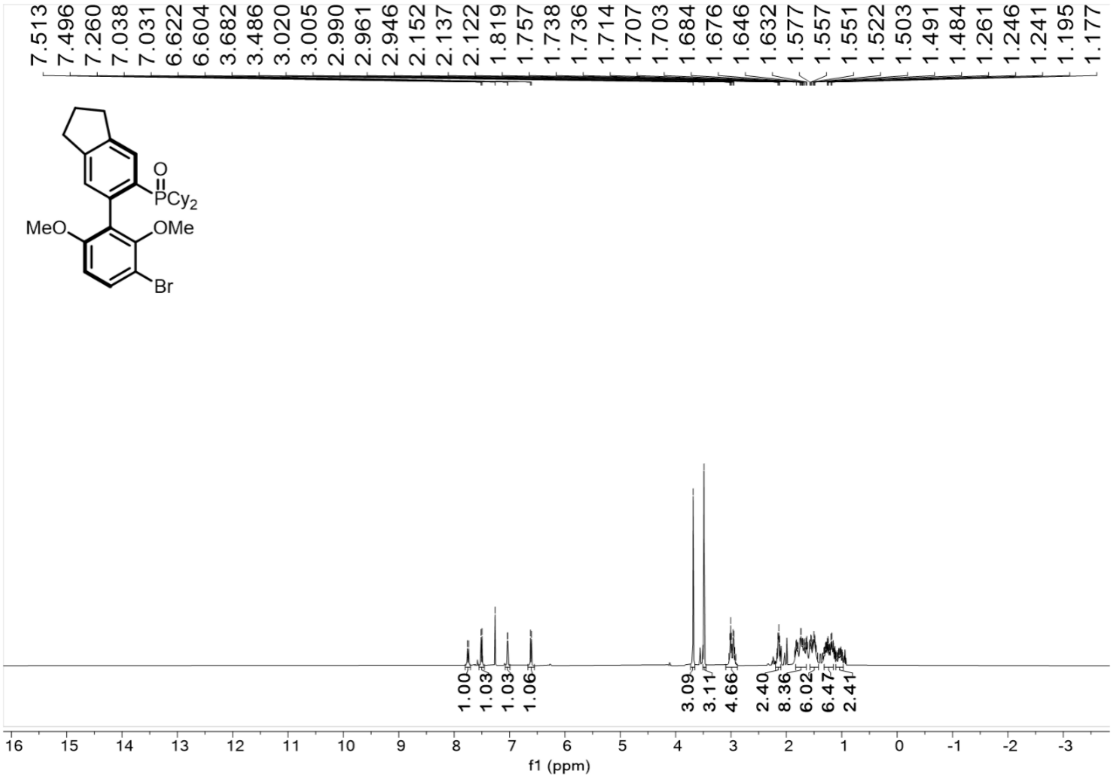

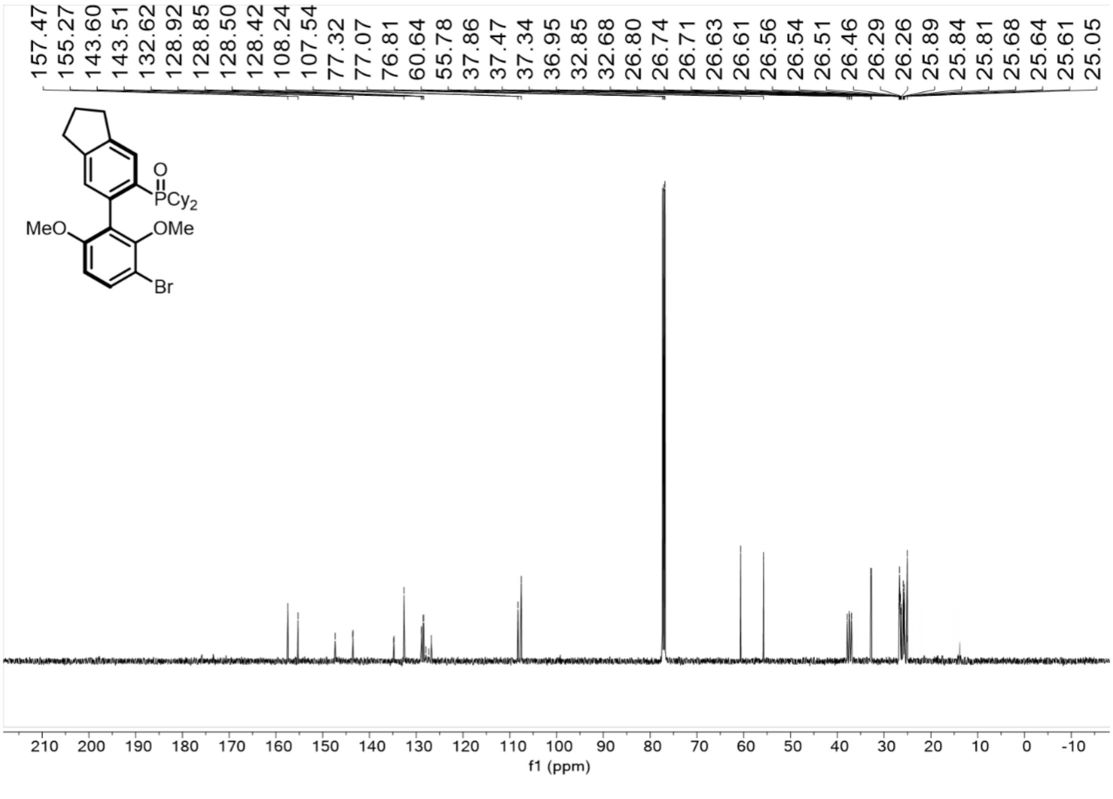

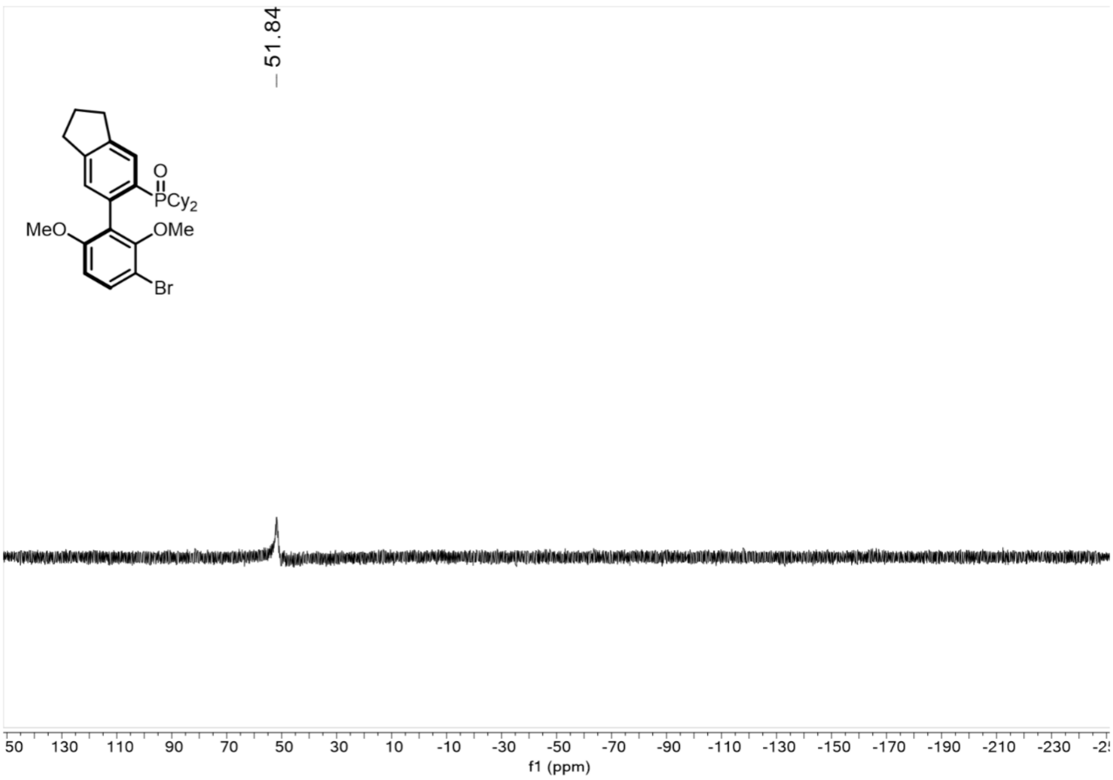

51.84
O
PCy2
MeO
OMe
Br
50
130
110
90
70
50
30
10
-10
-30
-50
-70
-90
-110
-130
-150
-170
-190
-210
-230
f1 (ppm)

(*R*)-(6-(3-bromo-2,6-dimethoxyphenyl)benzo[*d*][1,3]dioxol-5-yl)dicyclohexylphosphine oxide (36); $CDCl_3$

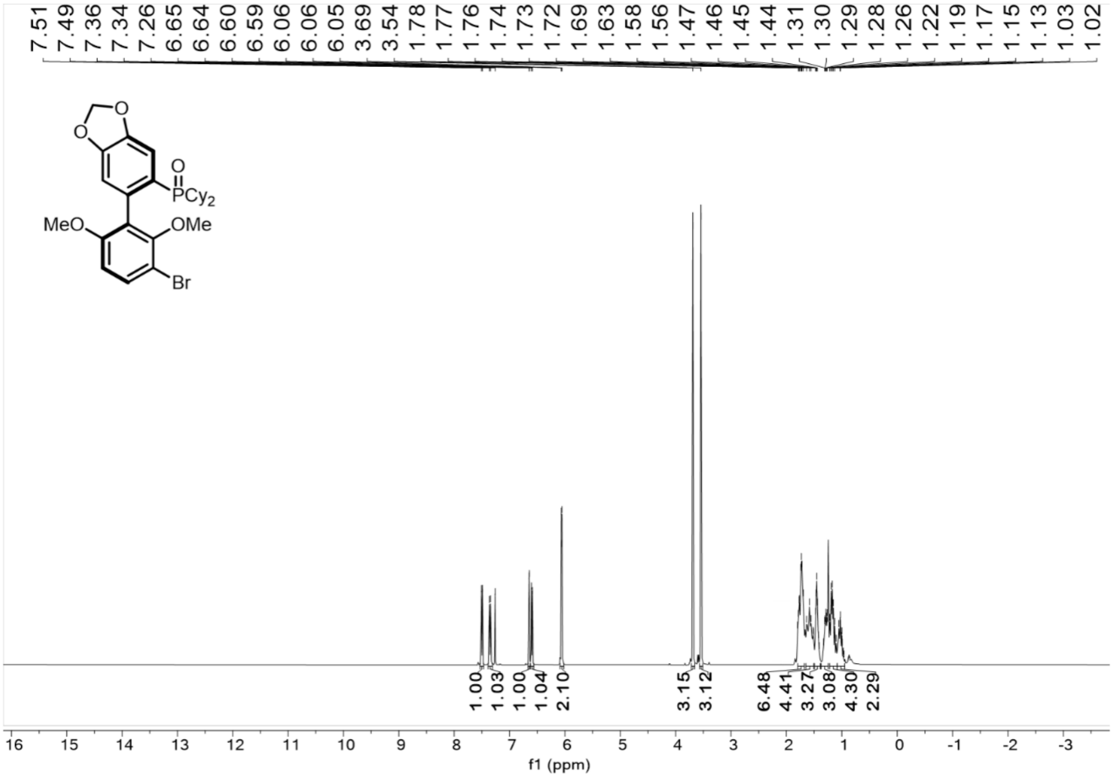

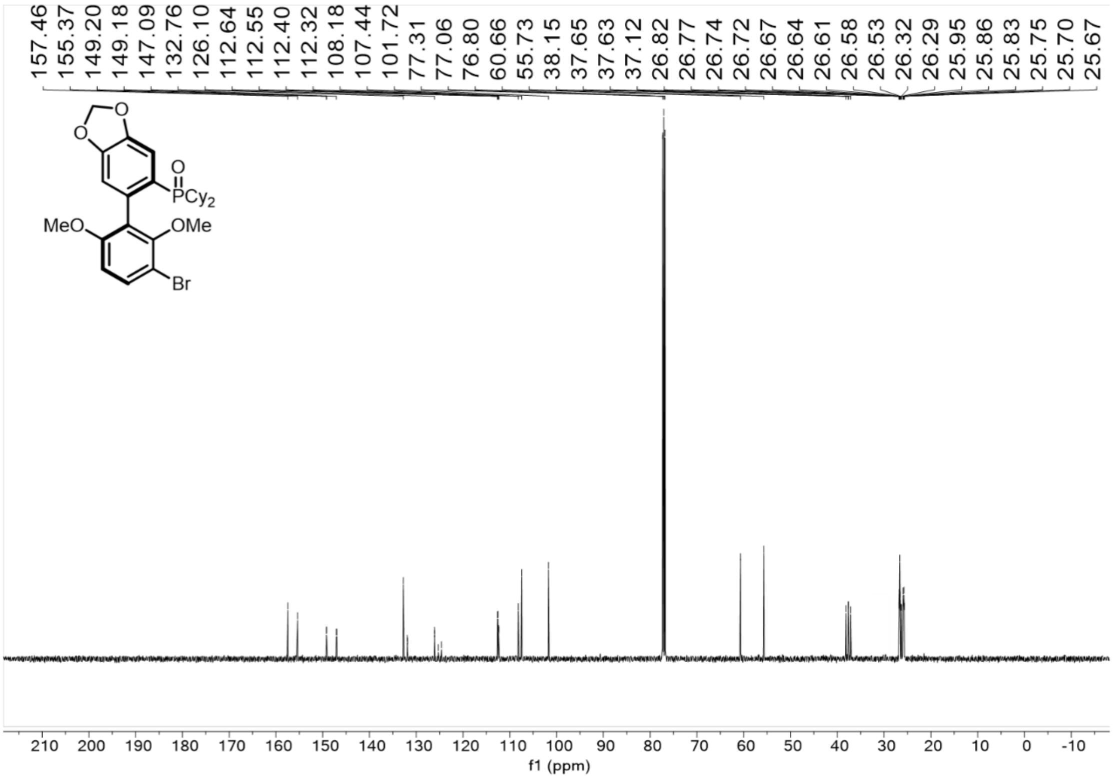

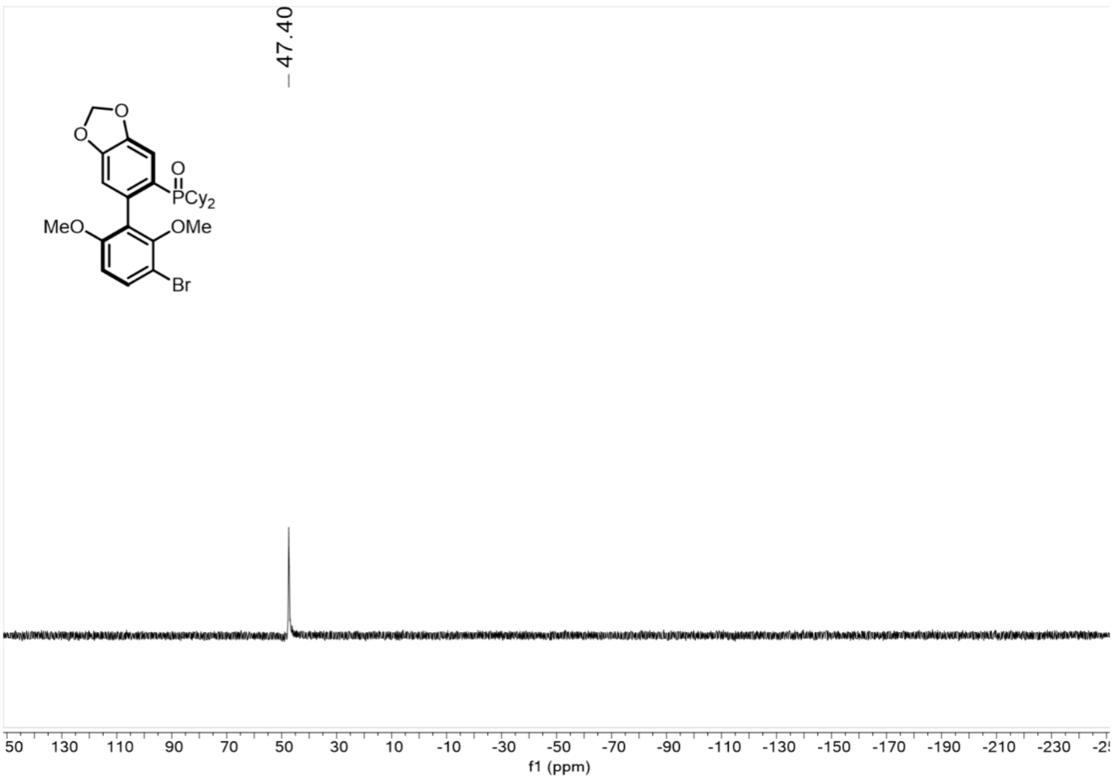
47.40
O
O
O
PCy2
MeO
OMe
Br
50
130
110
90
70
50
30
10
-10
-30
-50
-70
-90
-110
-130
-150
-170
-190
-210
-230
f1 (ppm)

(*R*)-(7-(3-bromo-2,6-dimethoxyphenyl)-2,3-dihydrobenzo[*b*][1,4]dioxin-6-yl)dicyclohexylphosphine oxide (37); $CDCl_3$

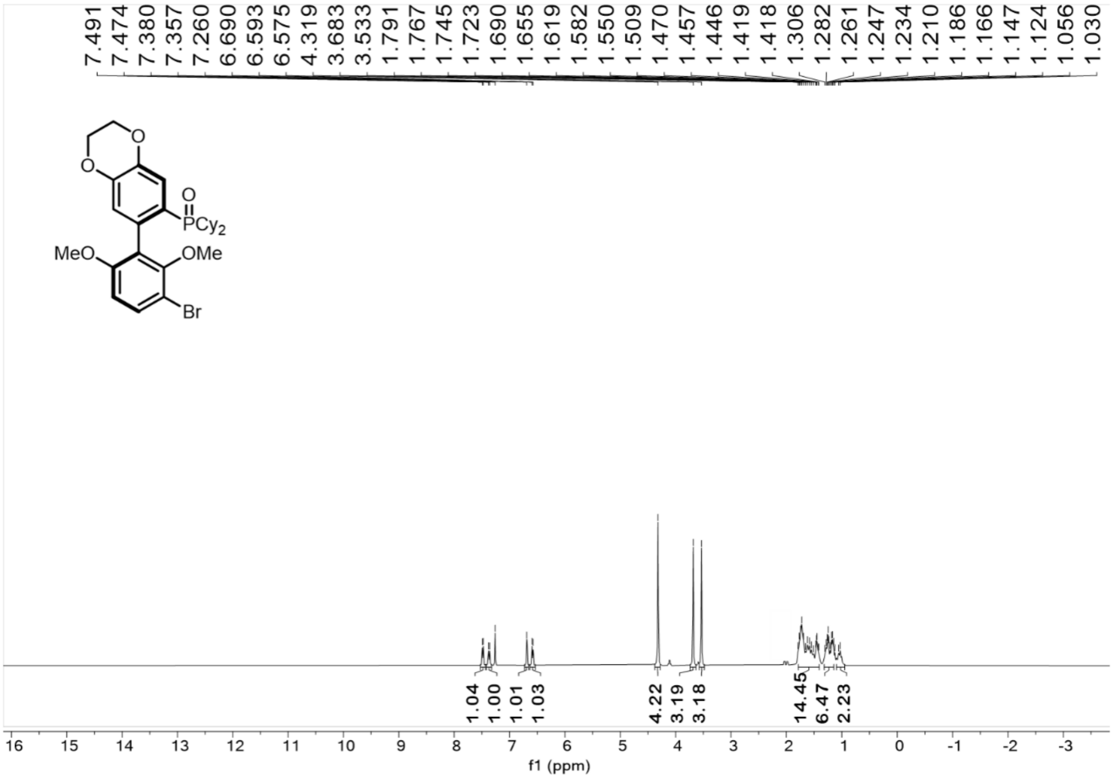

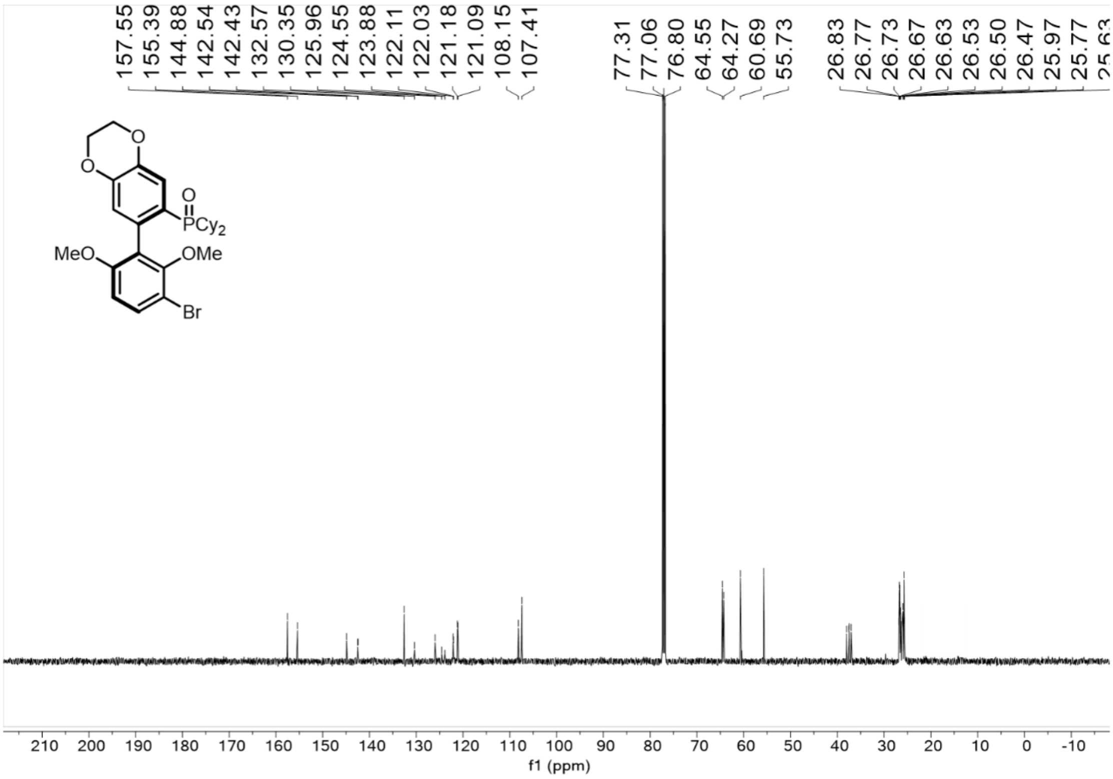

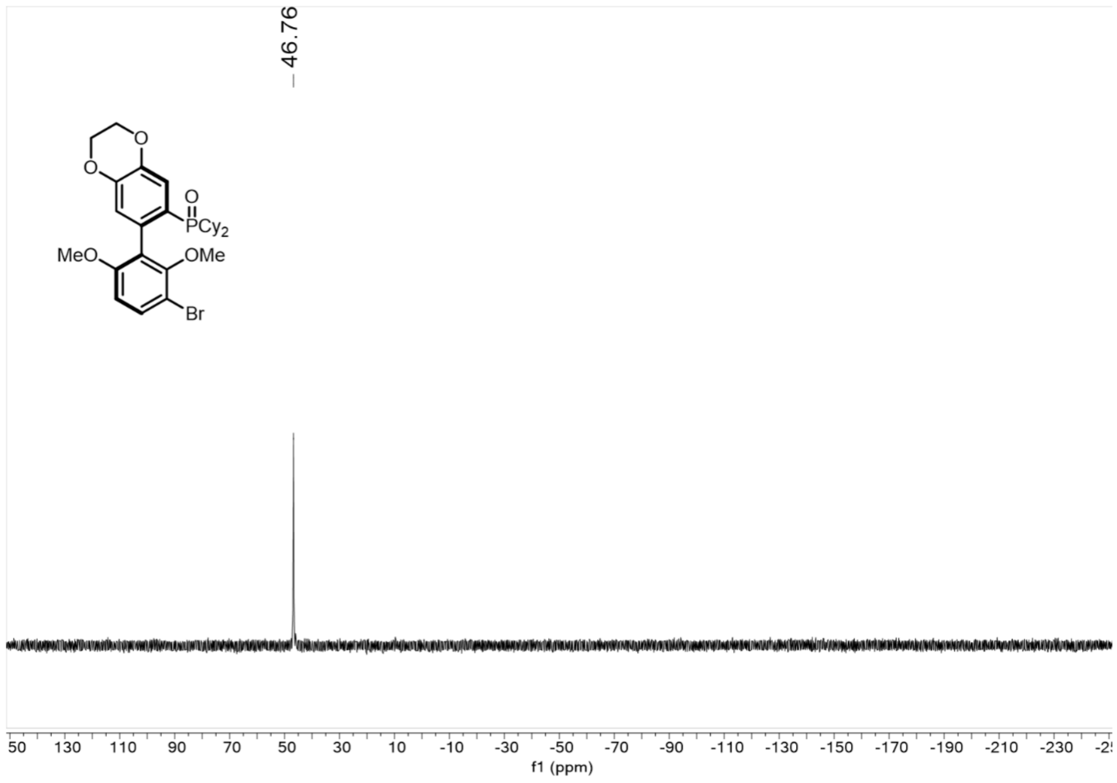

46.76
O
O
O
PCy2
MeO
OMe
Br
f1 (ppm)

(*R*)-(2-(3-bromo-2,6-dimethoxyphenyl)-1-methyl-1H-indol-3-yl)dicyclohexylphosphine oxide (38); $CDCl_3$

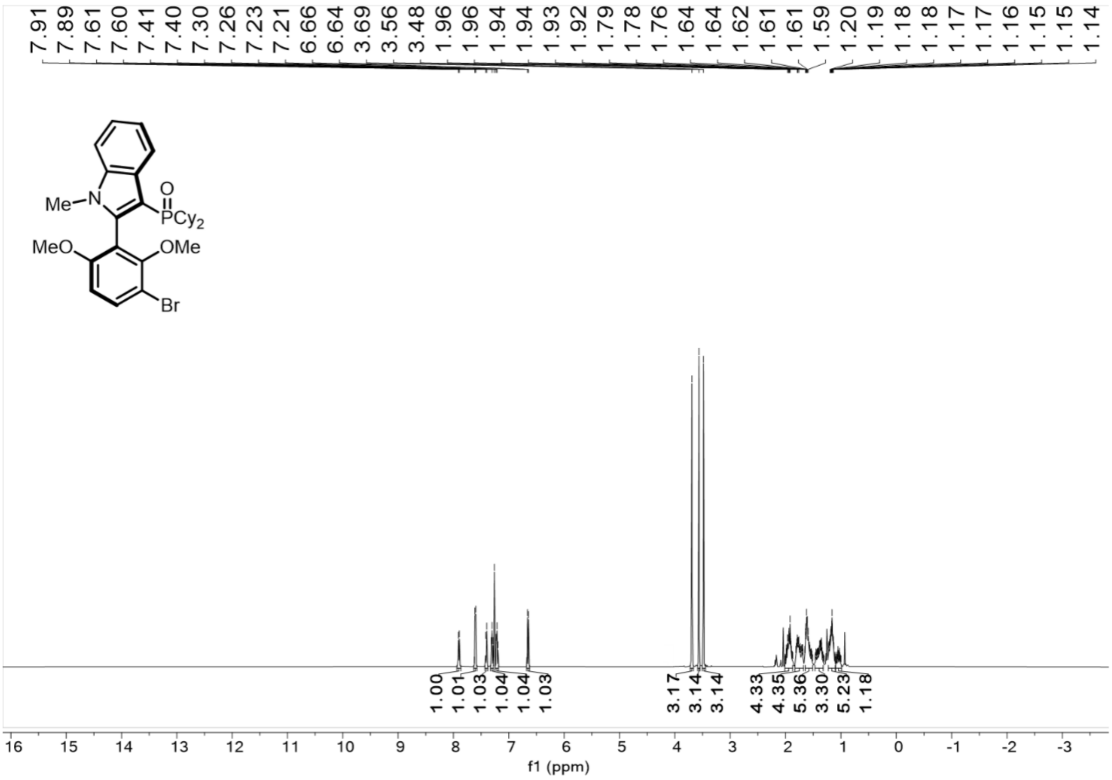

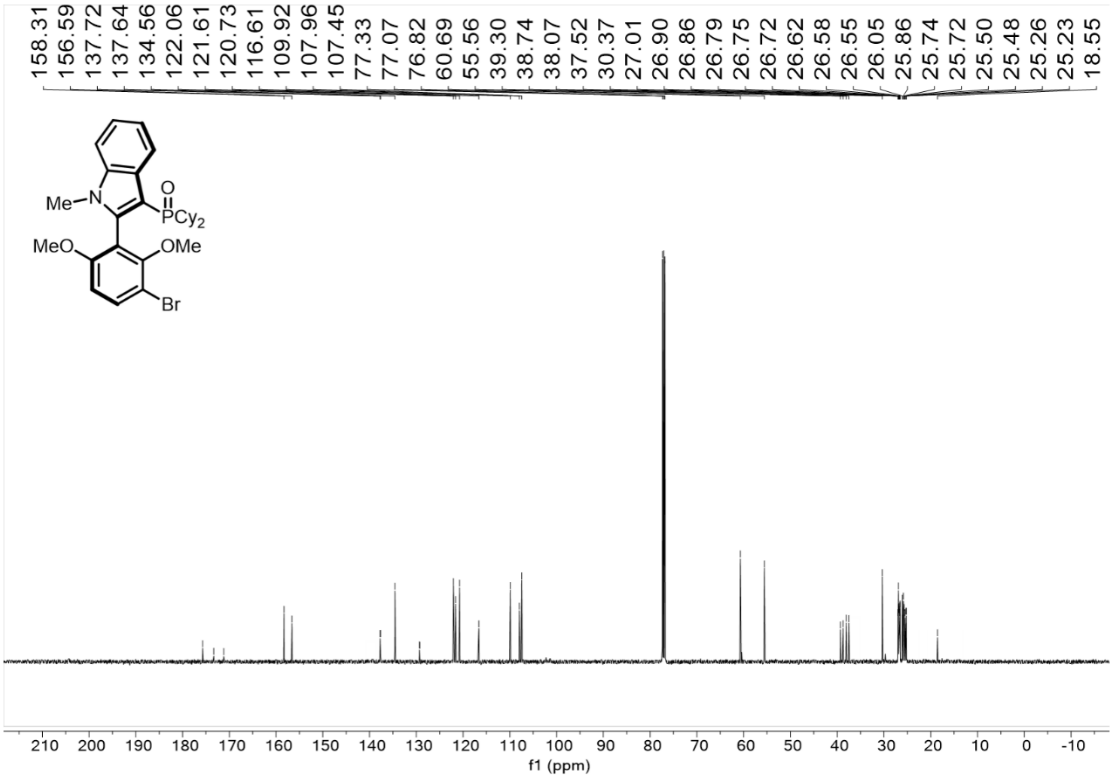

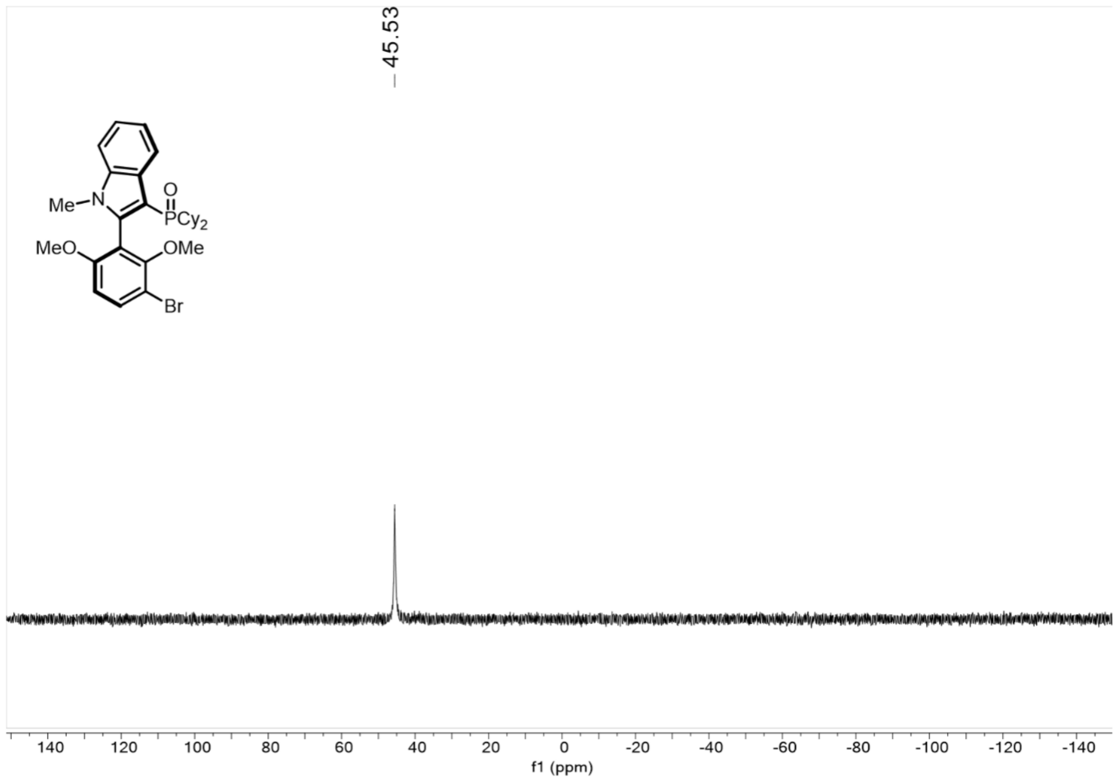

45.53
Me
N
O
PCy2
MeO
OMe
Br
140
120
100
80
60
40
20
0
-20
-40
-60
-80
-100
-120
-140
f1 (ppm)

(*R*)-(3-(3-bromo-2,6-dimethoxyphenyl)naphthalen-2-yl)dicyclohexylphosphine oxide (39); $CDCl_3$

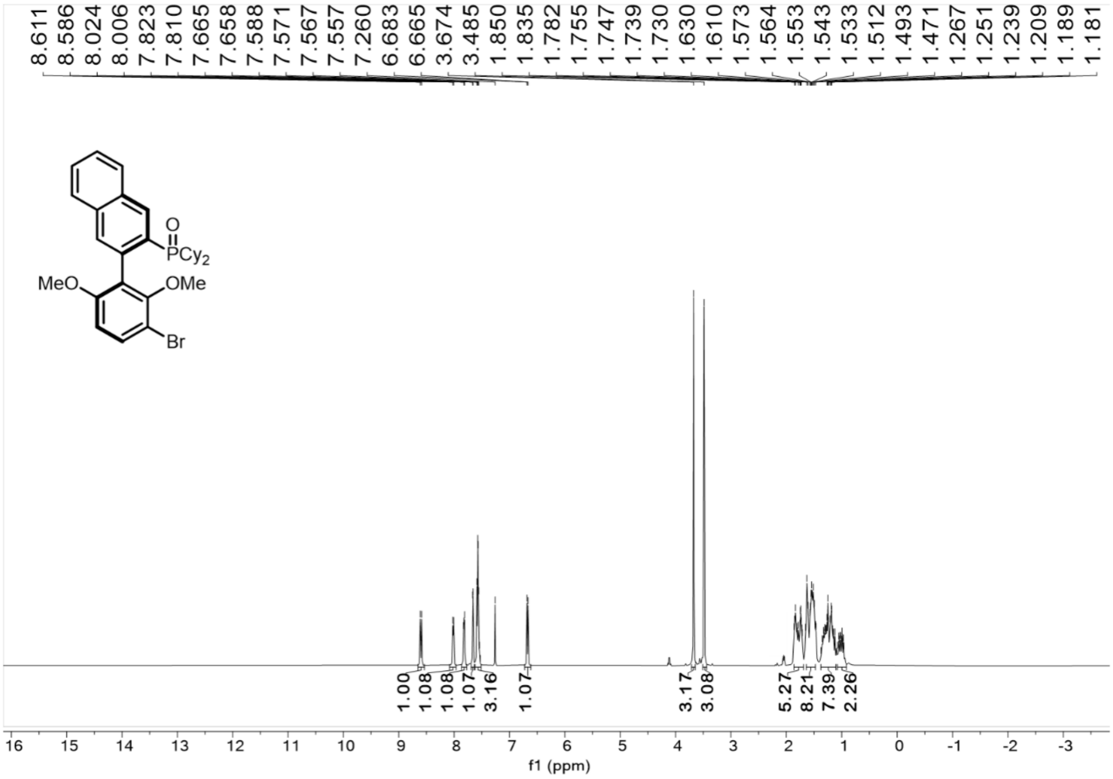

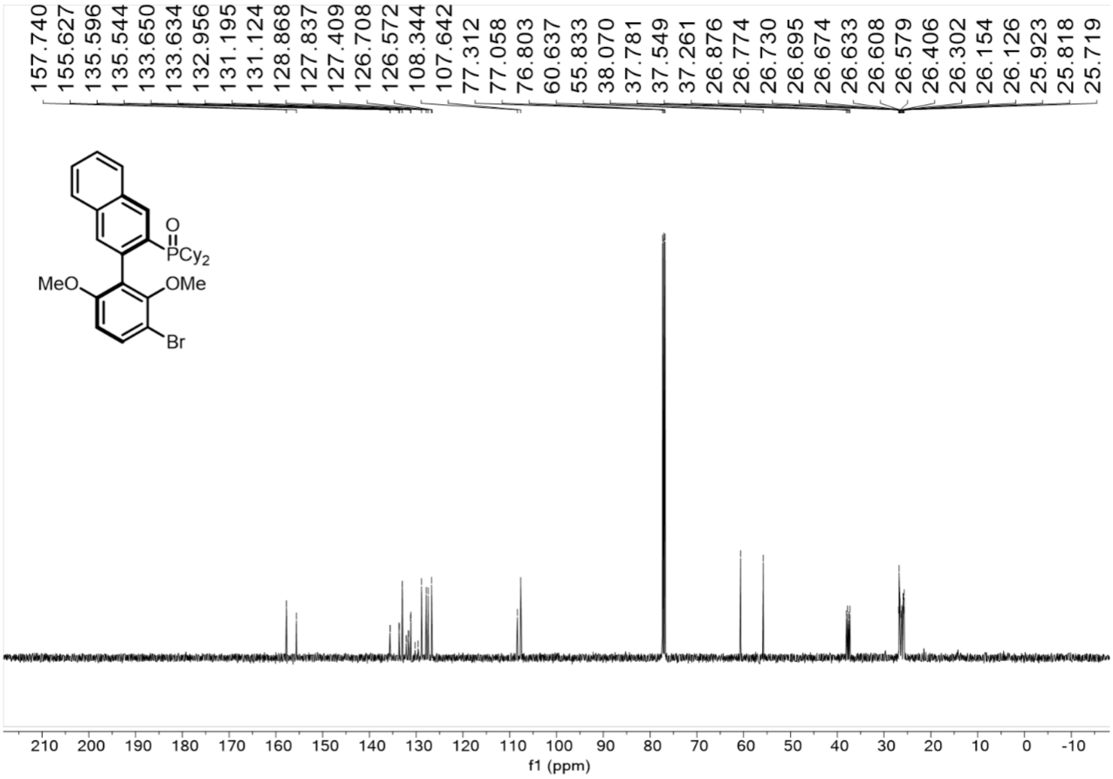

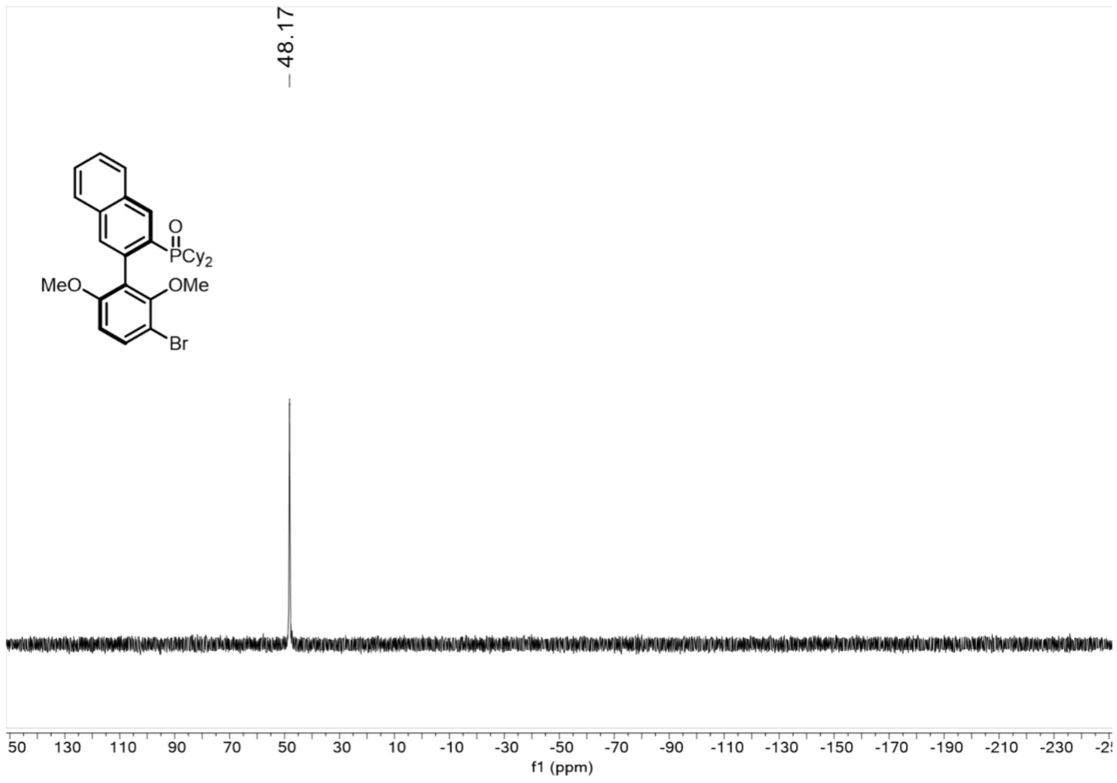

48.17
O
PCy2
MeO
OMe
Br
50
130
110
90
70
50
30
10
-10
-30
-50
-70
-90
-110
-130
-150
-170
-190
-210
-230
f1 (ppm)

(*R*)-(1-(3-bromo-2,6-dimethoxyphenyl)naphthalen-2-yl)dicyclohexylphosphine oxide (40); $CDCl_3$

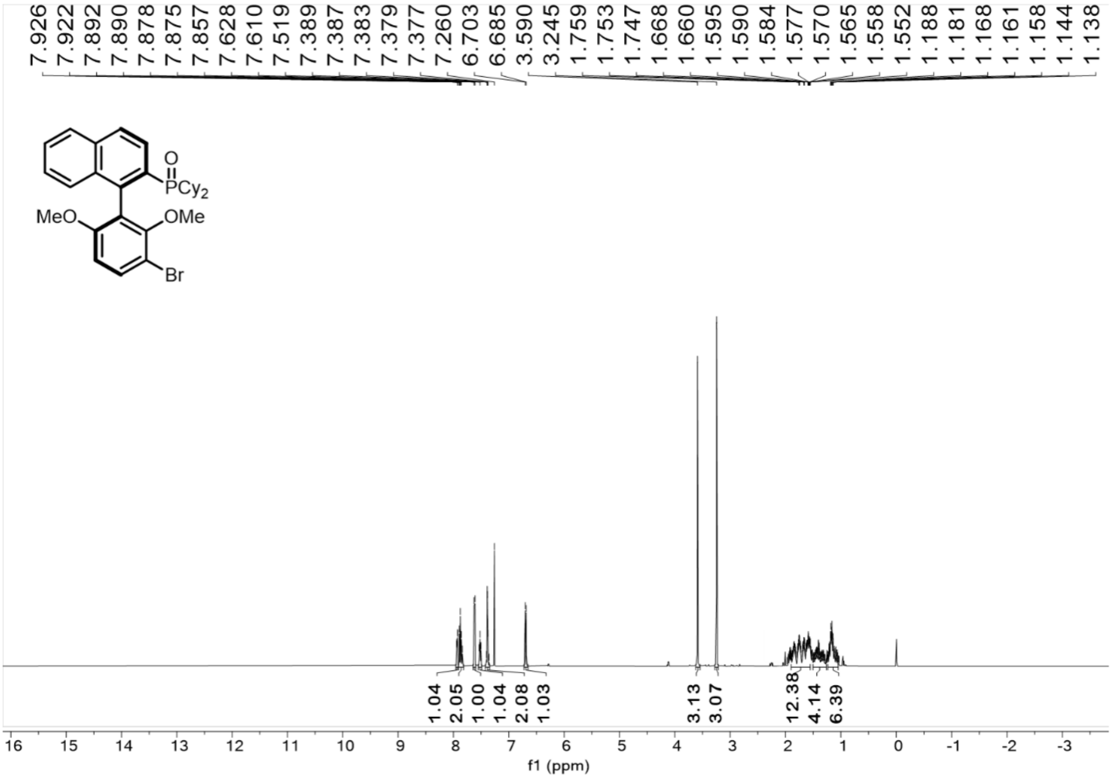

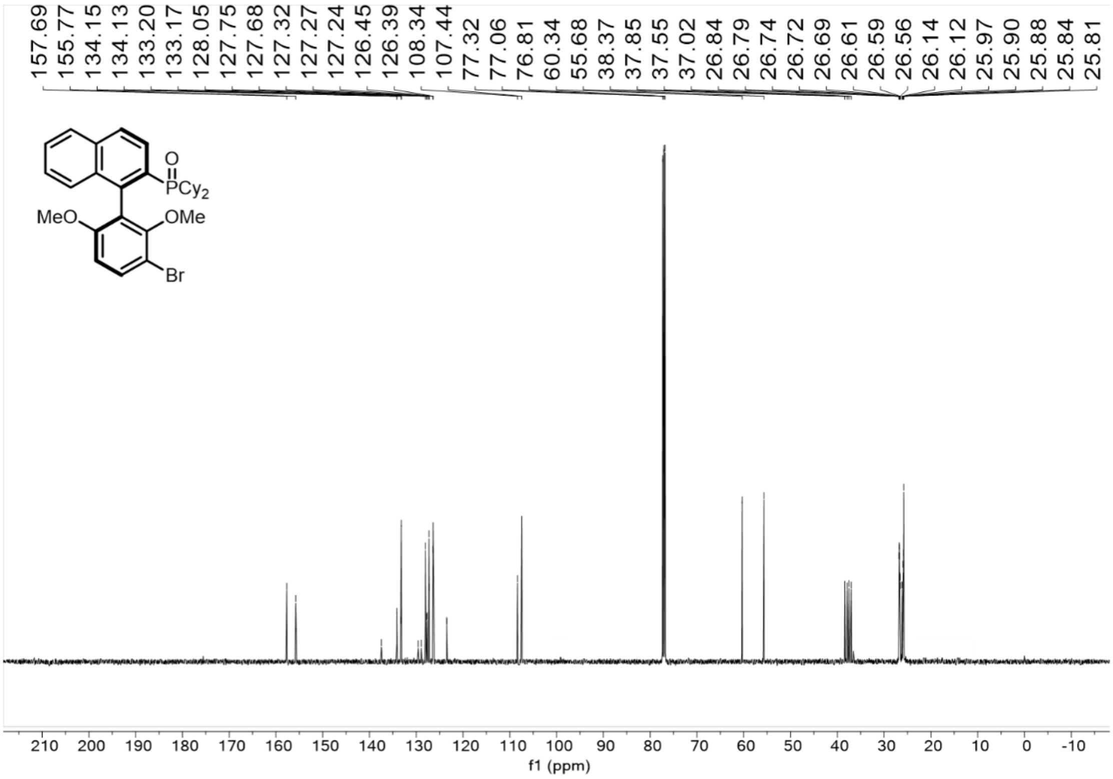

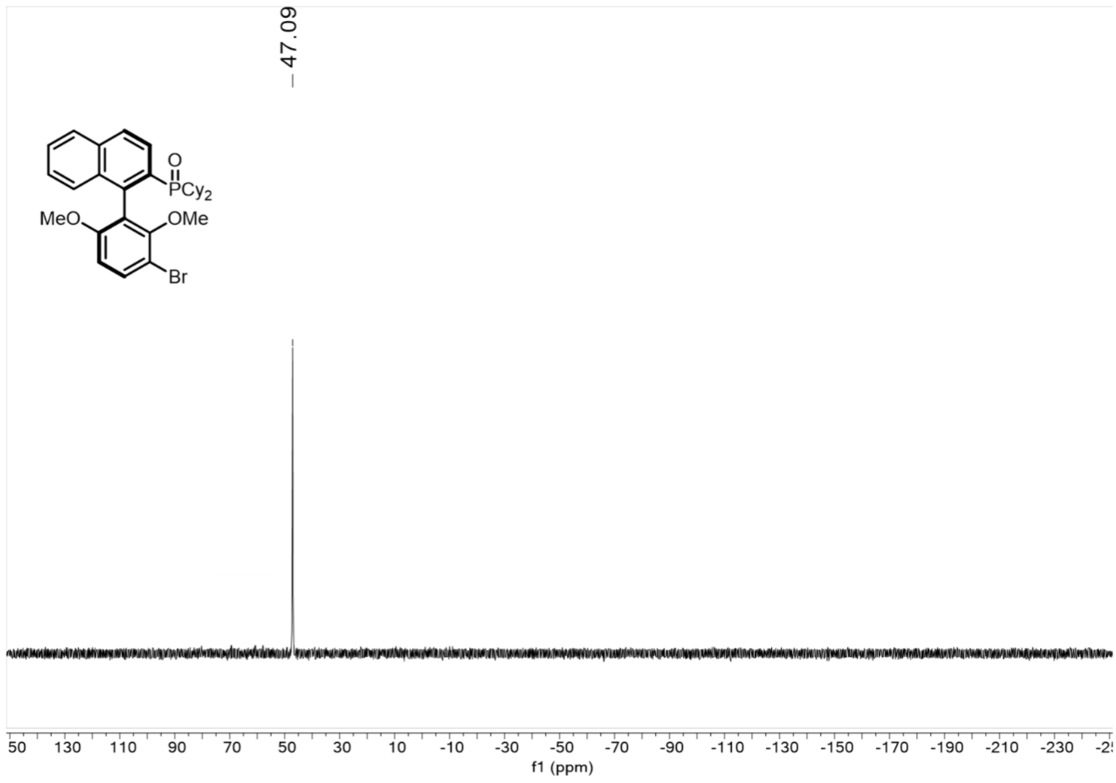

47.09
O
PCy2
MeO
OMe
Br
50
130
110
90
70
50
30
10
-10
-30
-50
-70
-90
-110
-130
-150
-170
-190
-210
-230
f1 (ppm)

(*R*)-(10-(3-bromo-2,6-dimethoxyphenyl)phenanthren-9-yl)dicyclohexylphosphine oxide (41); $CDCl_3$

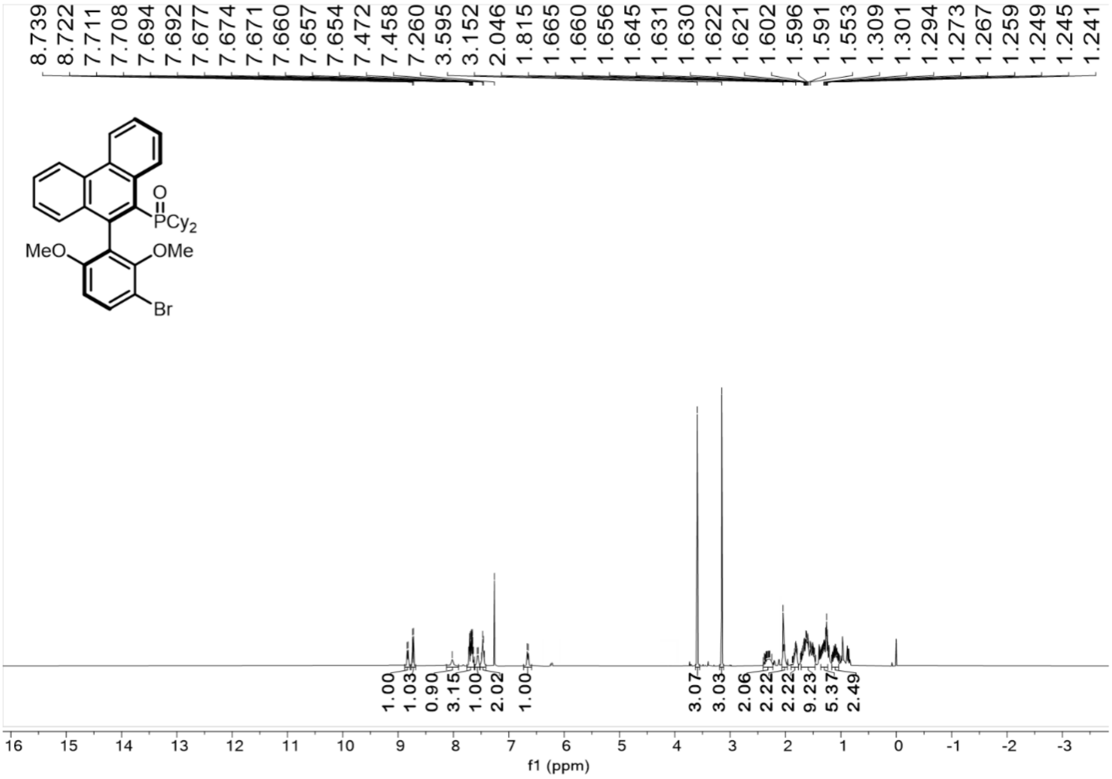

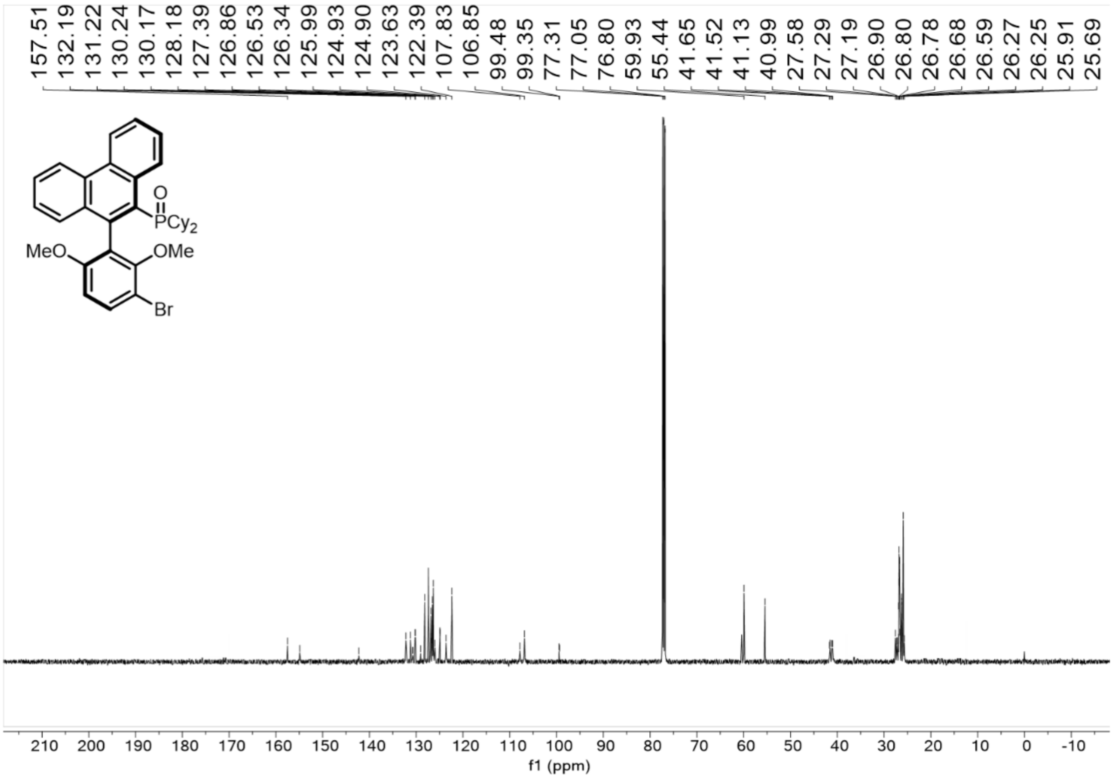

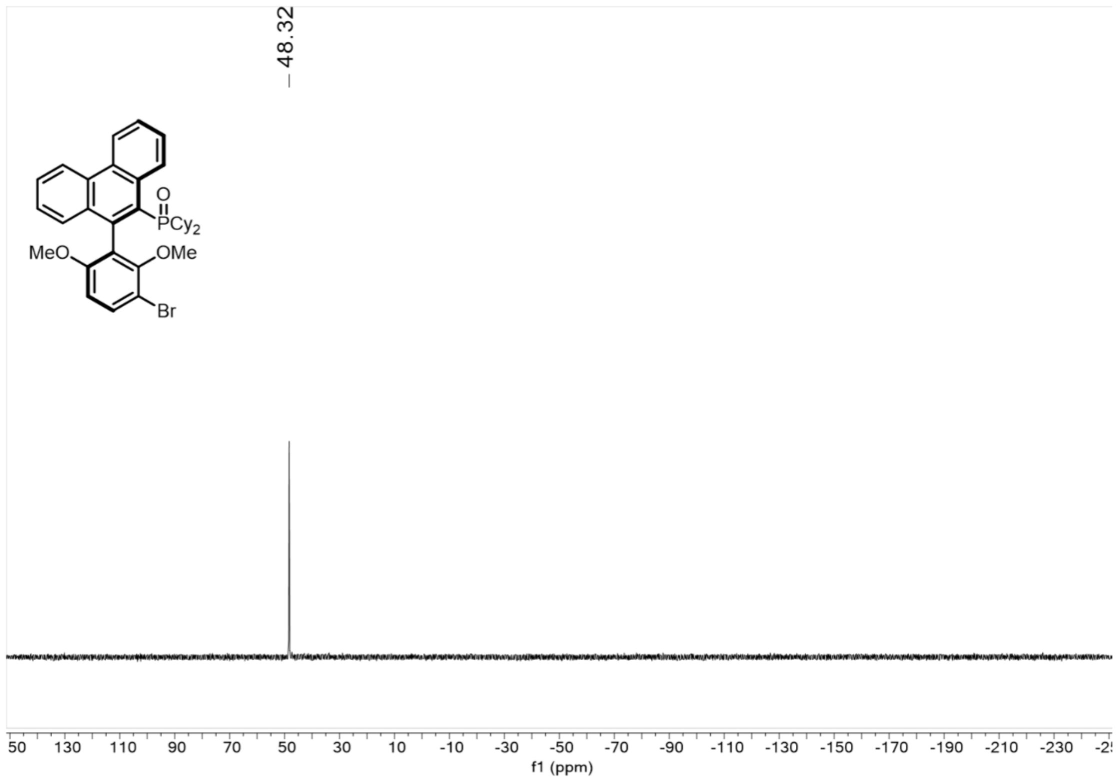

48.32
MeO
OMe
PCy2
O
Br
50
130
110
90
70
50
30
10
-10
-30
-50
-70
-90
-110
-130
-150
-170
-190
-210
-230
f1 (ppm)

(*R*)-(3'-bromo-5'-chloro-2',6'-dimethoxy-[1,1'-biphenyl]-2-yl)dicyclohexylphosphine oxide (42); $CDCl_3$

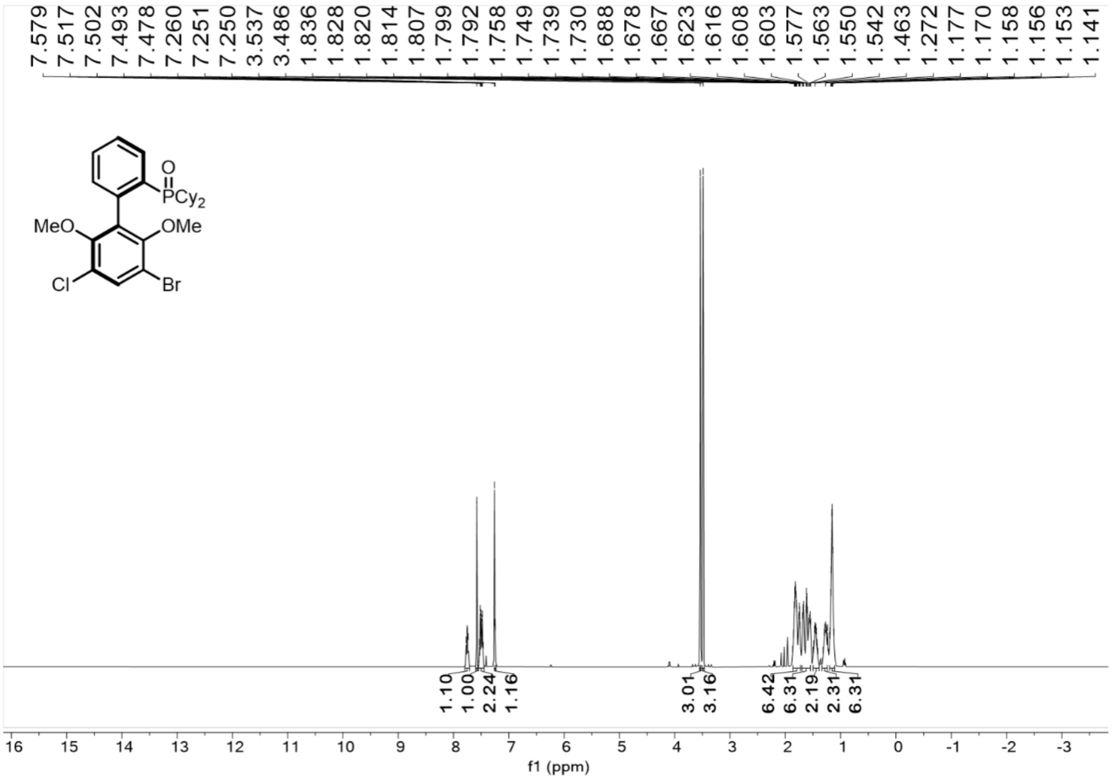

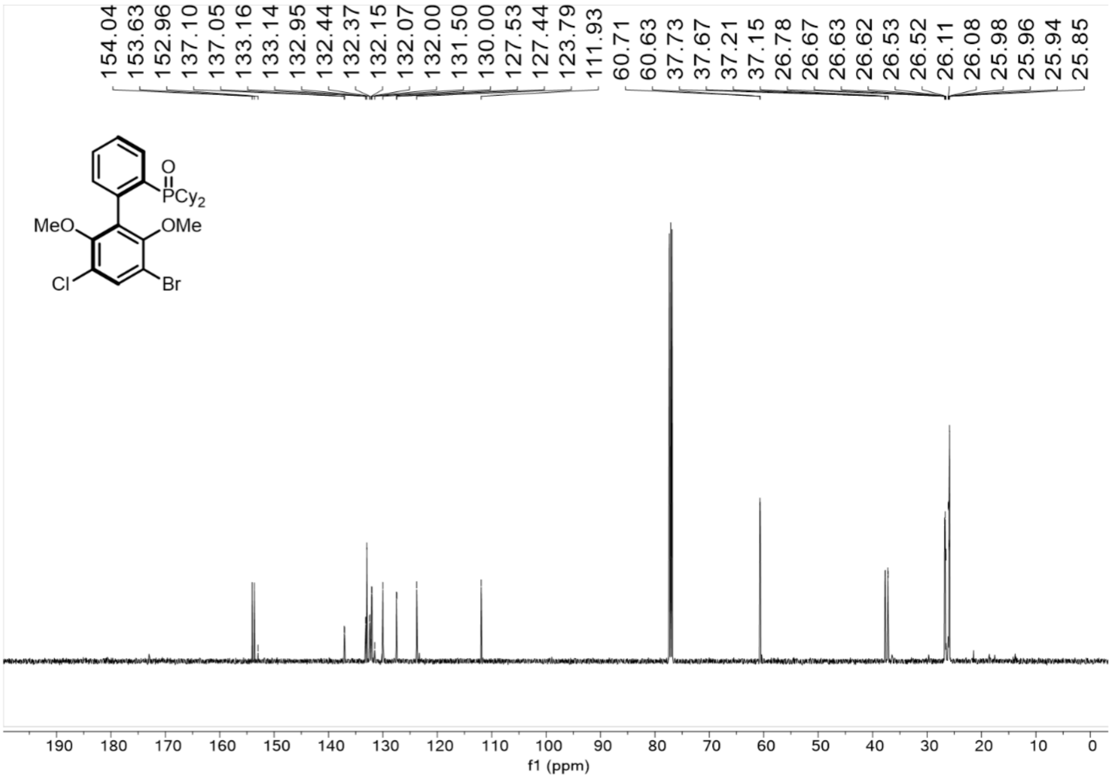

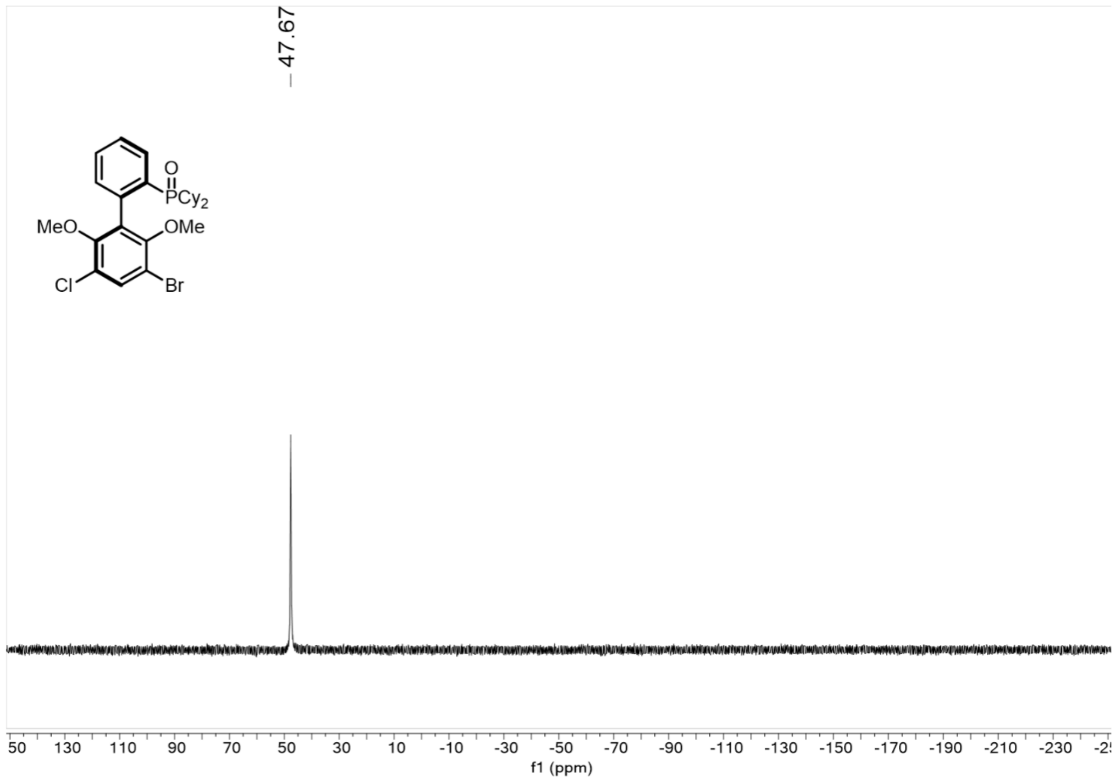

47.67
O
PCy2
MeO
OMe
Cl
Br
50
130
110
90
70
50
30
10
-10
-30
-50
-70
-90
-110
-130
-150
-170
-190
-210
-230
f1 (ppm)

(*R*)-(3'-bromo-2',6'-dimethoxy-[1,1'-biphenyl]-2-yl)dicyclohexylphosphane ((*R*)-SPhos-Br); CDCl3

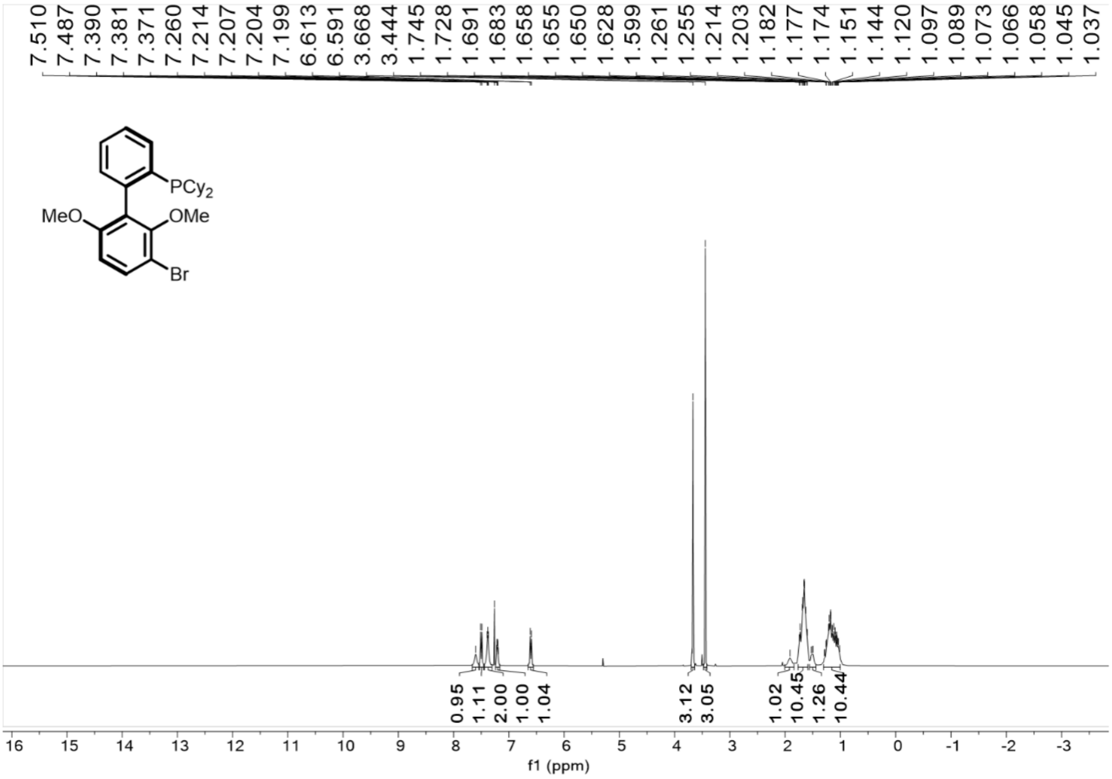

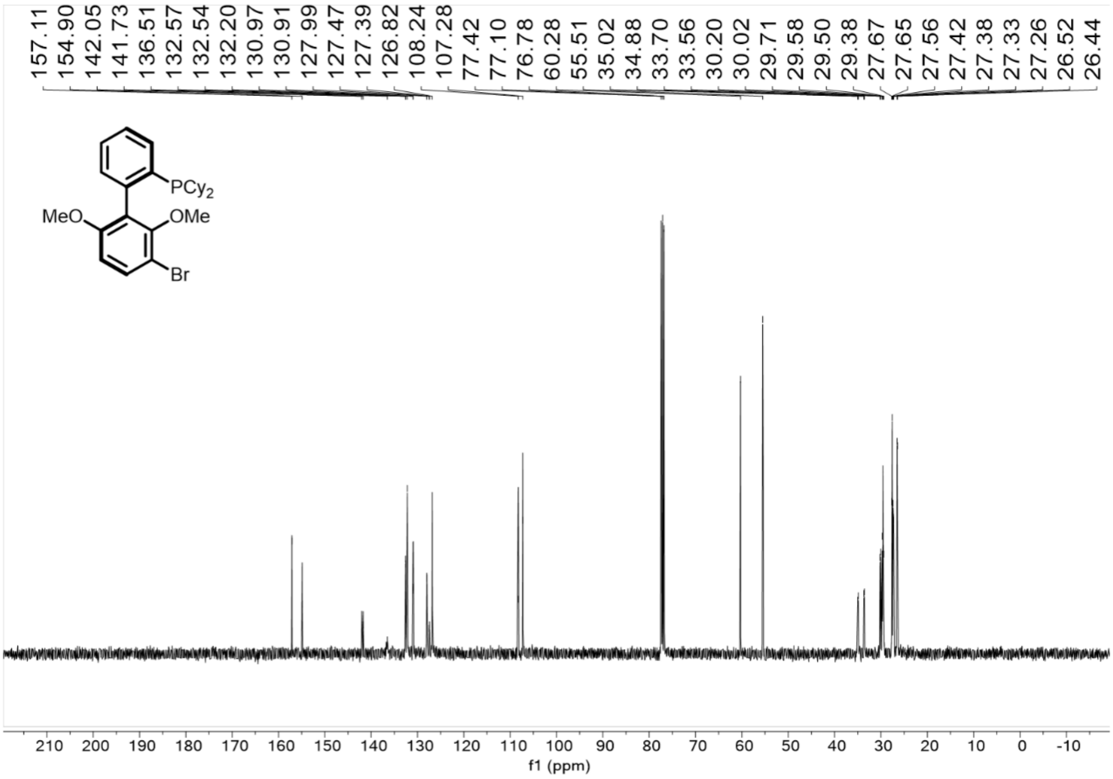

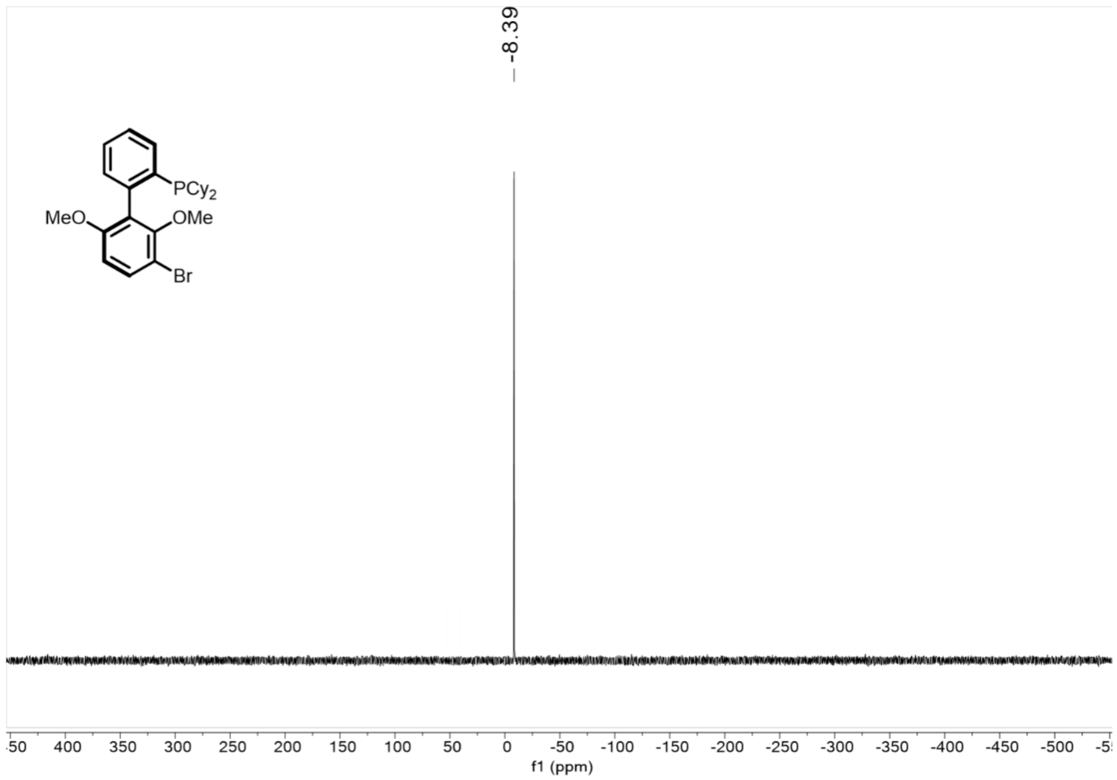

-8.39
PCy2
MeO
OMe
Br
f1 (ppm)

(*R*)-2'-(dicyclohexylphosphaneyl)-2,6-dimethoxy-[1,1'-biphenyl]-3-carboxylic acid ((*R*)-SPhos-CO_2H); Acetone-*d_6* and CD_3OD

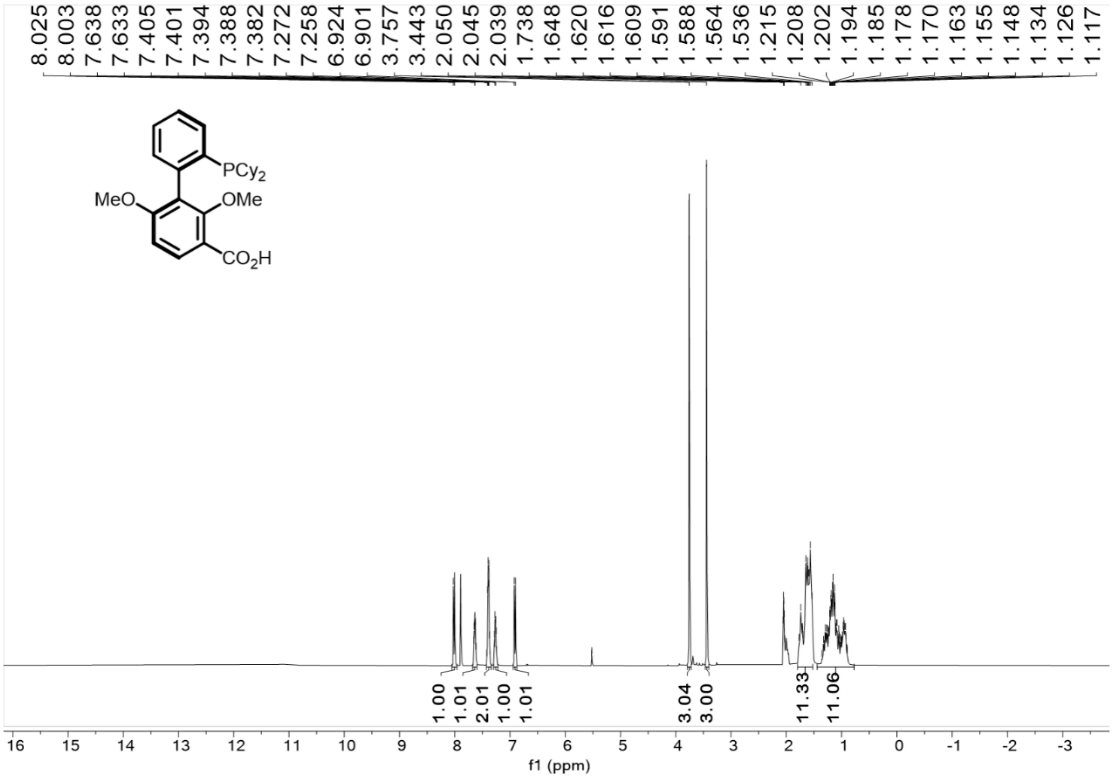

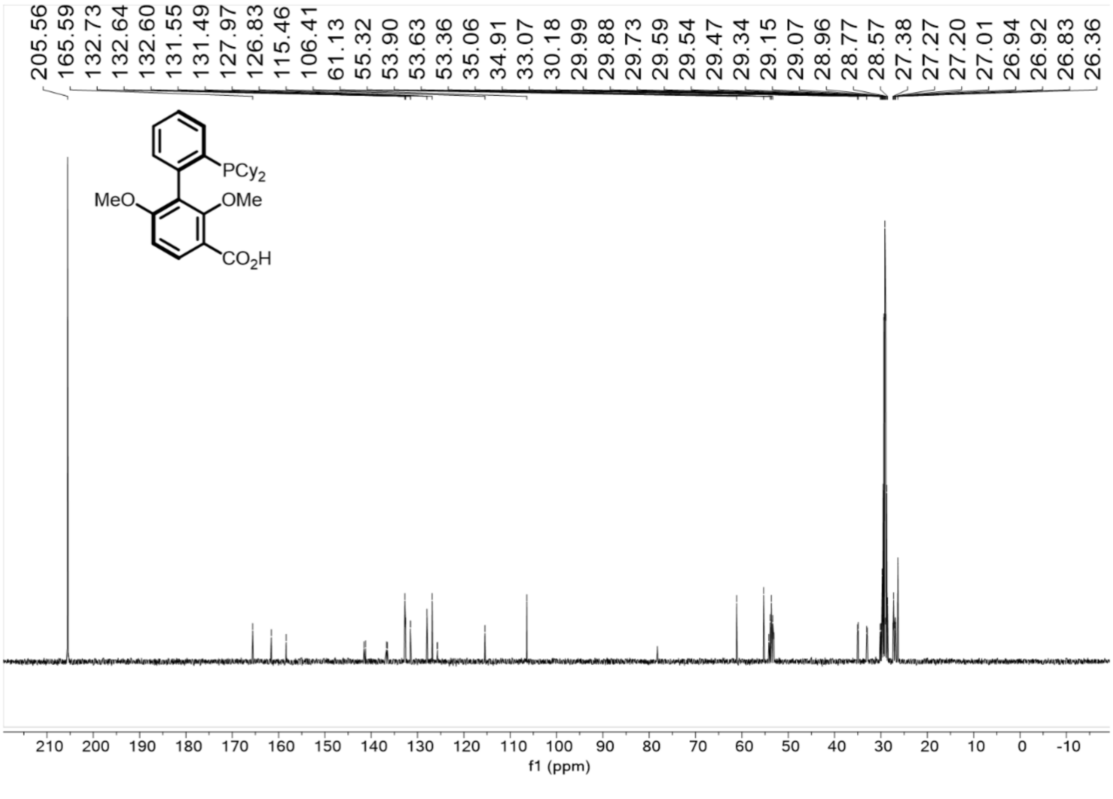

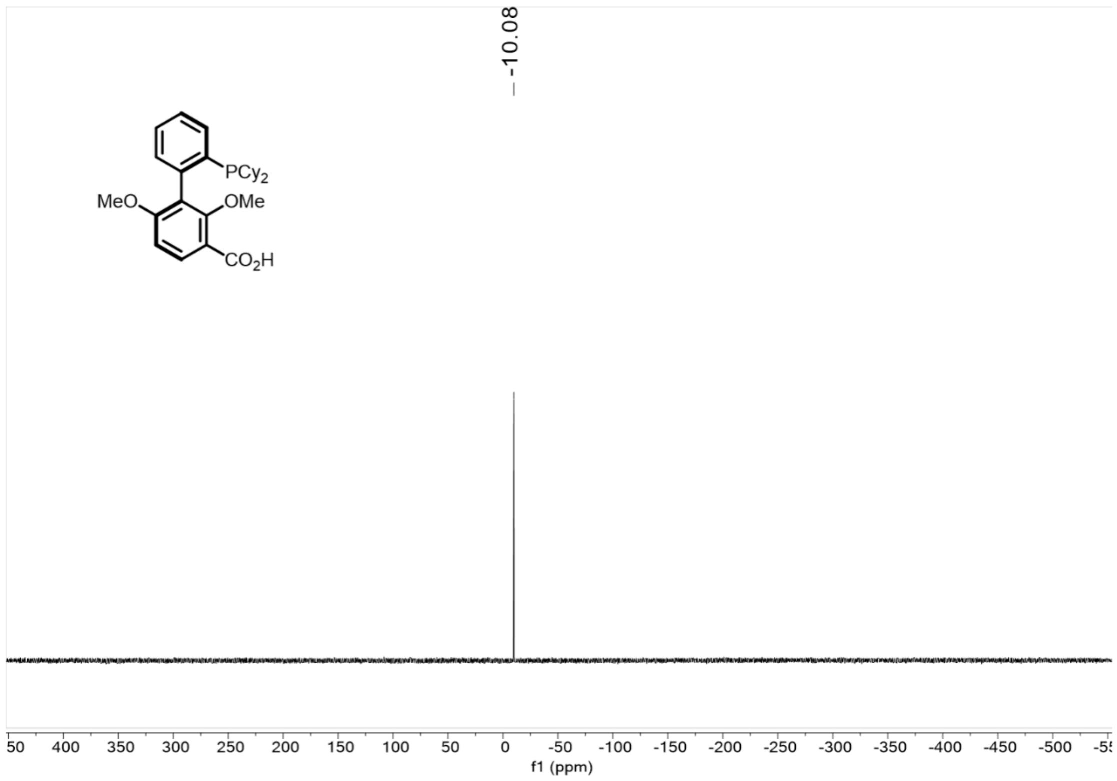
-10.08
PCy2
MeO
OMe
CO2H
50 400 350 300 250 200 150 100 50 0 -50 -100 -150 -200 -250 -300 -350 -400 -450 -500
f1 (ppm)

(*R*)-sodium 2'-(dicyclohexylphosphaneyl)-2,6-dimethoxy-[1,1'-biphenyl]-3-sulfonate ((*R*)-SPhos-SO_3Na); CD_3OD

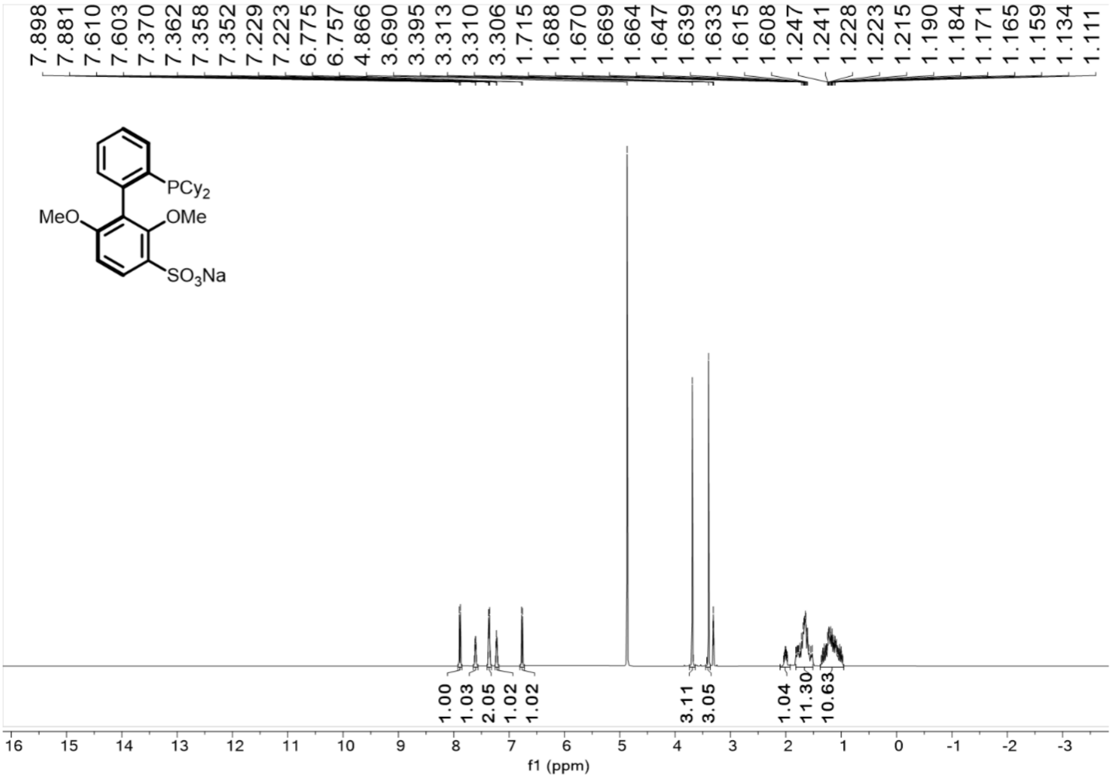

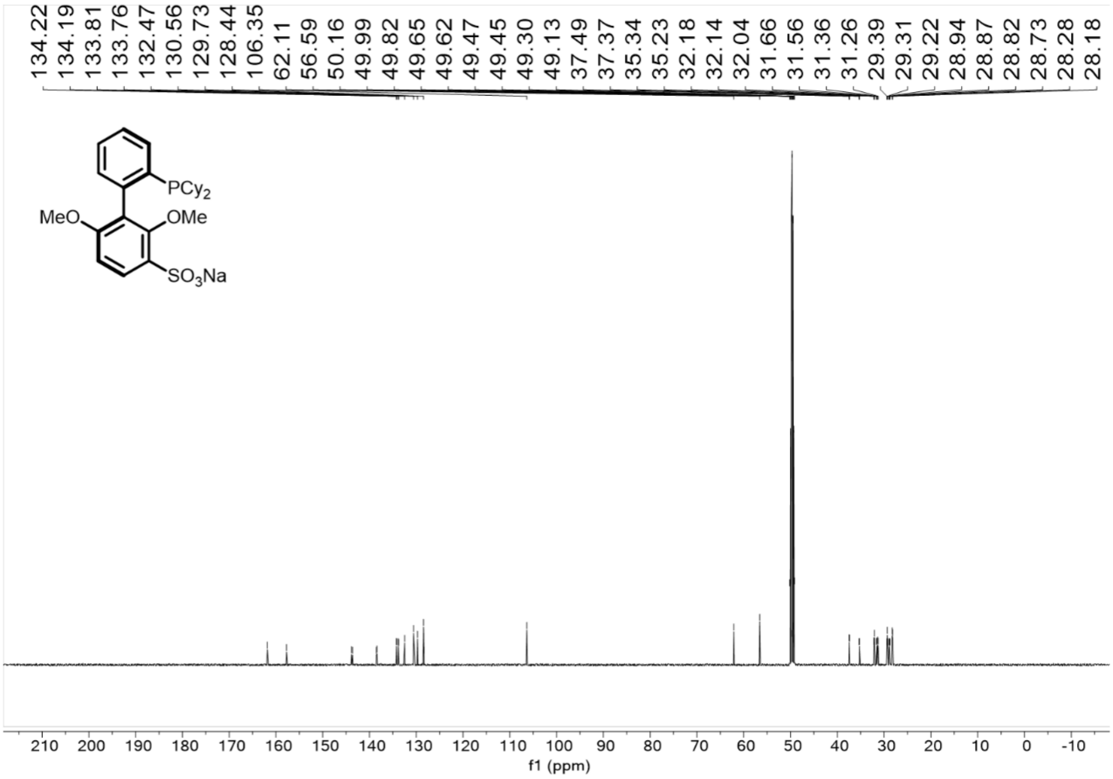

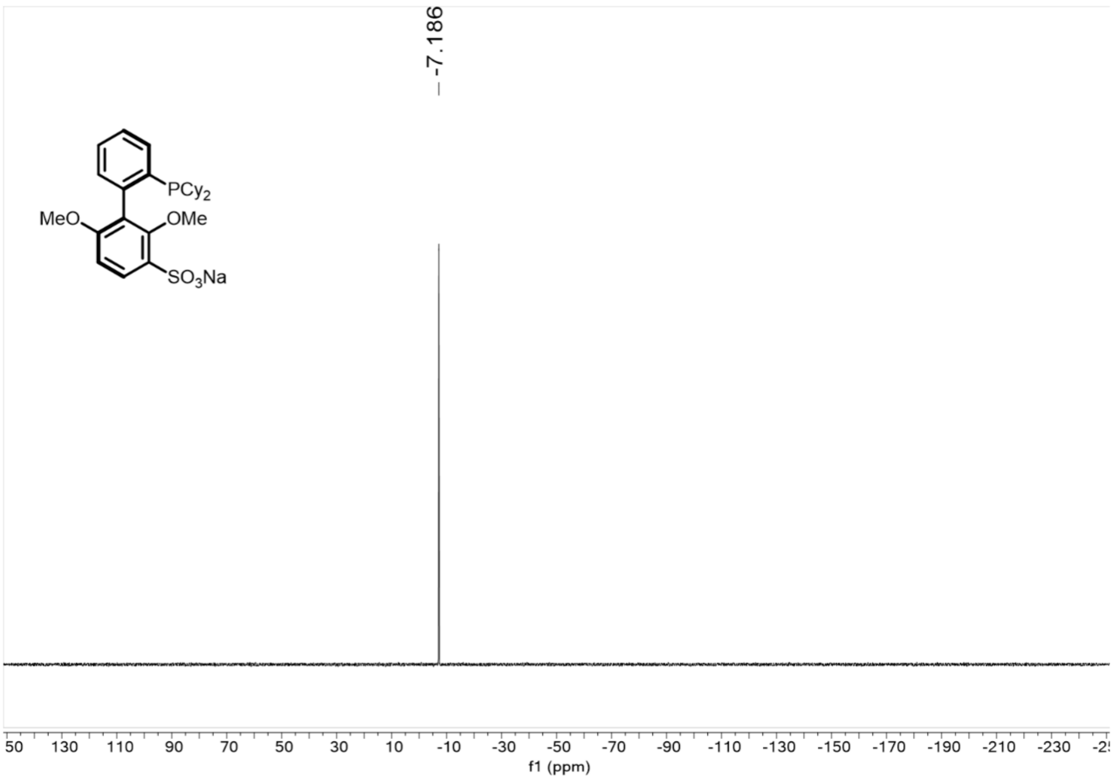
-7.186
PCy2
MeO
OMe
SO3Na
50
130
110
90
70
50
30
10
-10
-30
-50
-70
-90
-110
-130
-150
-170
-190
-210
-230
f1 (ppm)

(*R*)-dicyclohexyl(3'-(diphenylphosphaneyl)-2',6'-dimethoxy-[1,1'-biphenyl]-2-yl)phosphane ((*R*)-SPhos-PPh$_2$); CD_2Cl_2

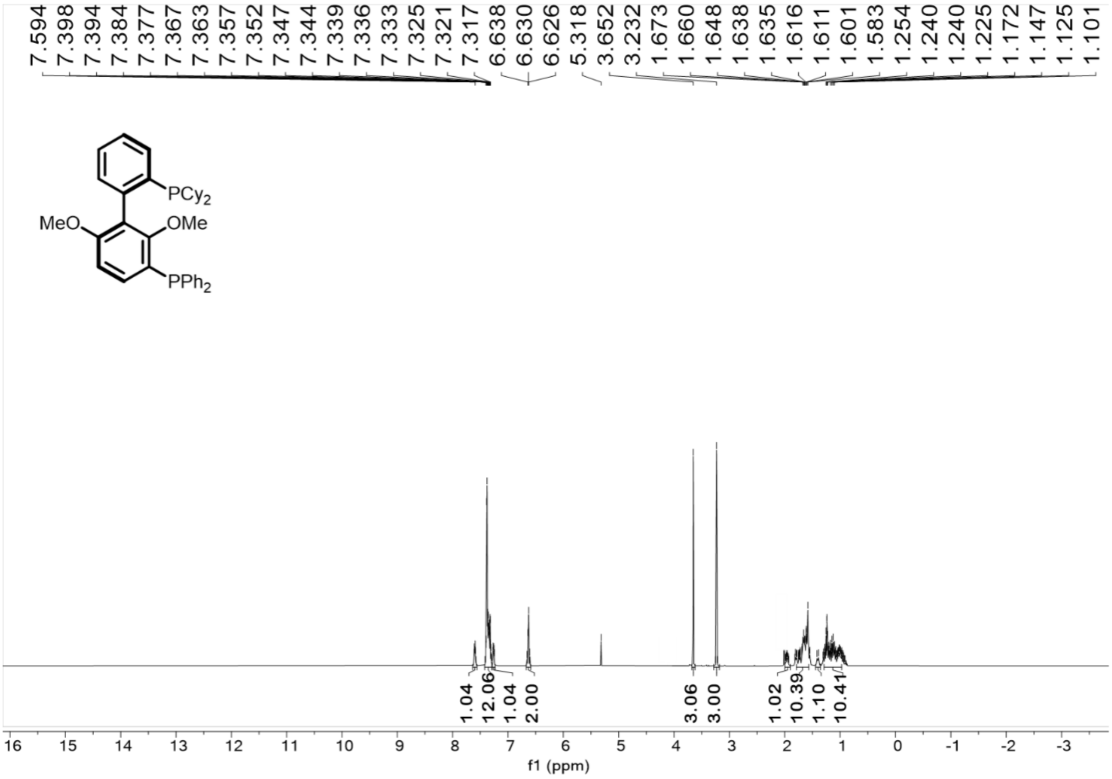

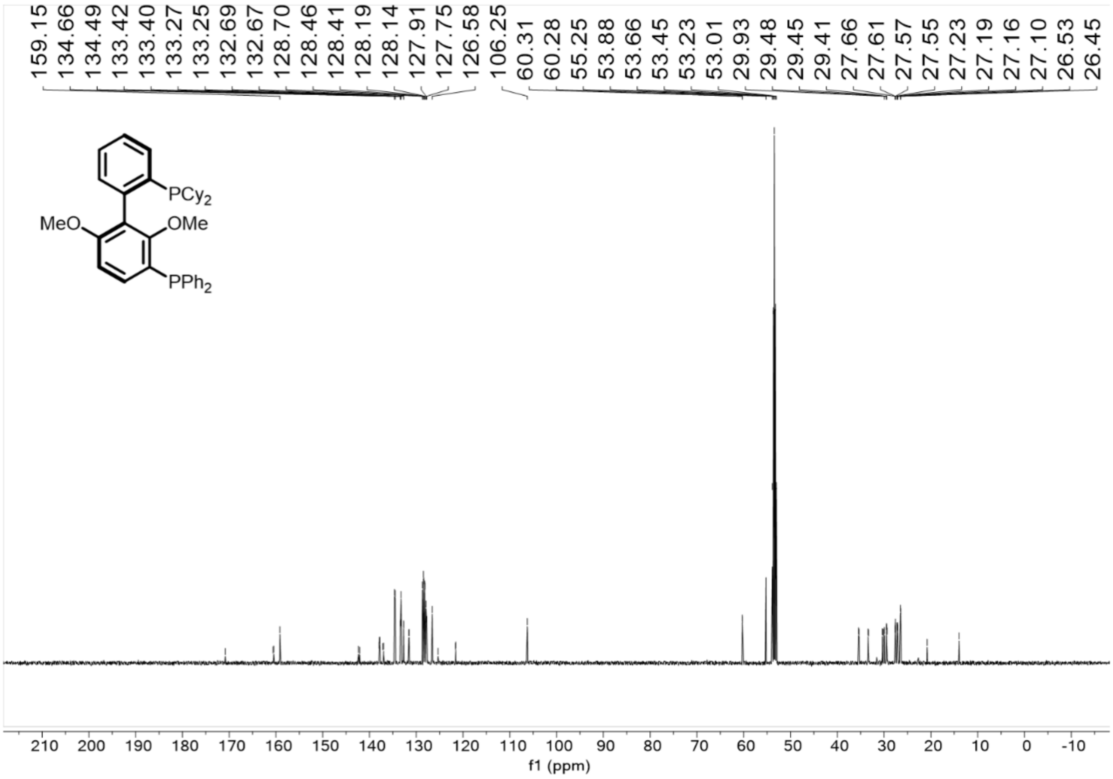

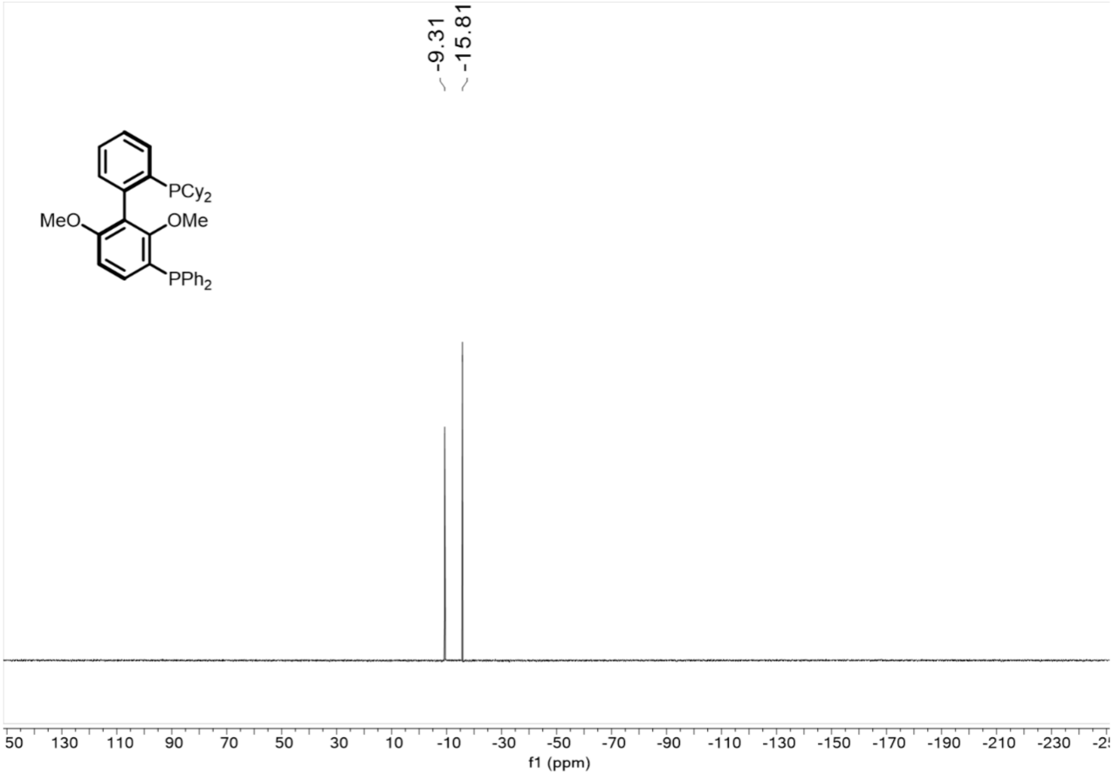

-9.31
-15.81
PCy2
MeO
OMe
PPh2
f1 (ppm)

(*R*)-dicyclohexyl(2',6'-dimethoxy-3'-(trimethylsilyl)-[1,1'-biphenyl]-2-yl)phosphane ((*R*)-SPhos-TMS); CD_2Cl_2

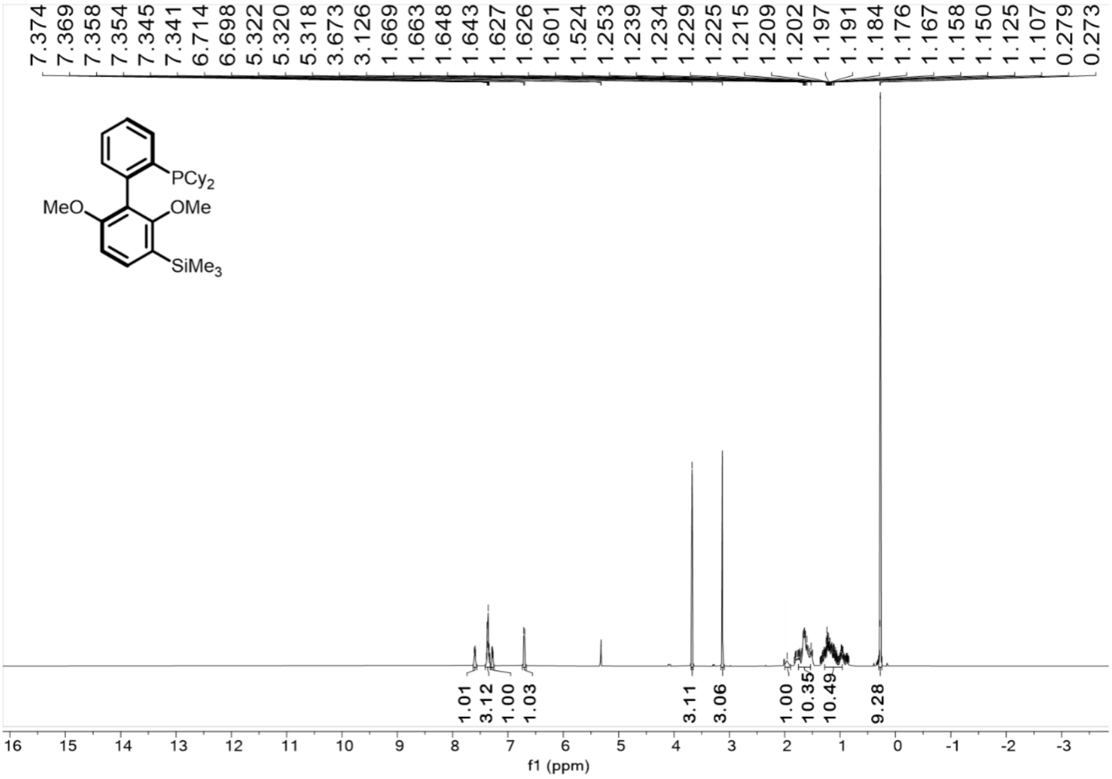

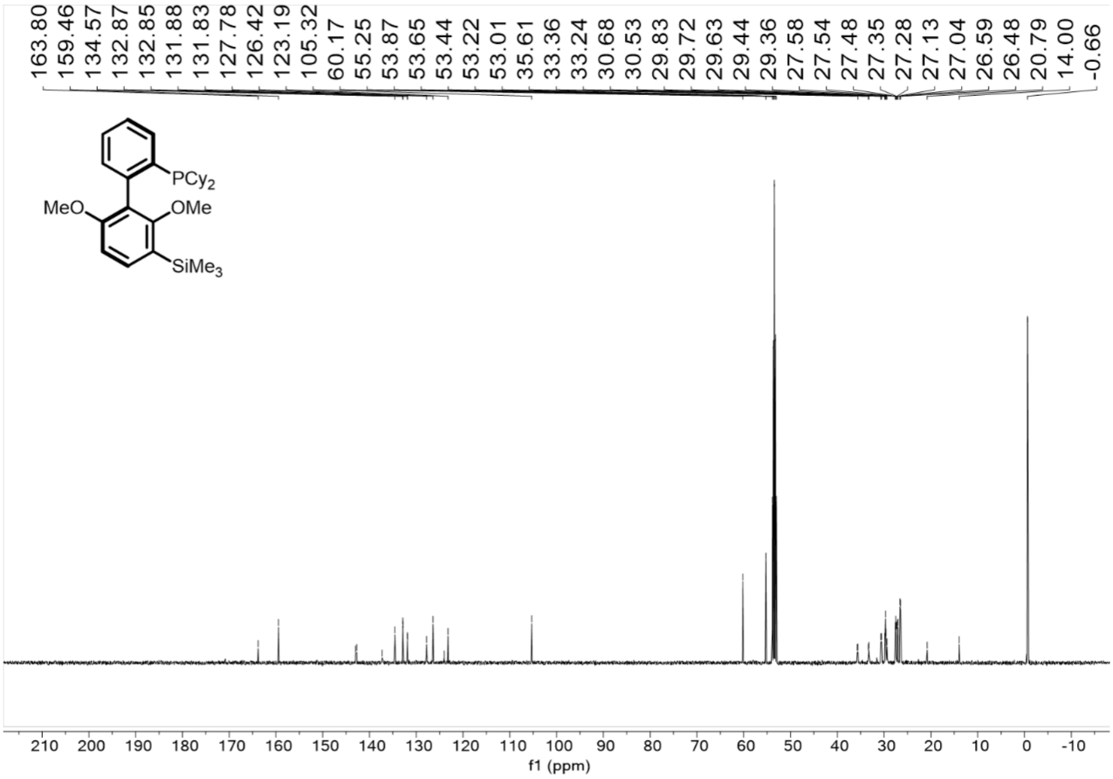

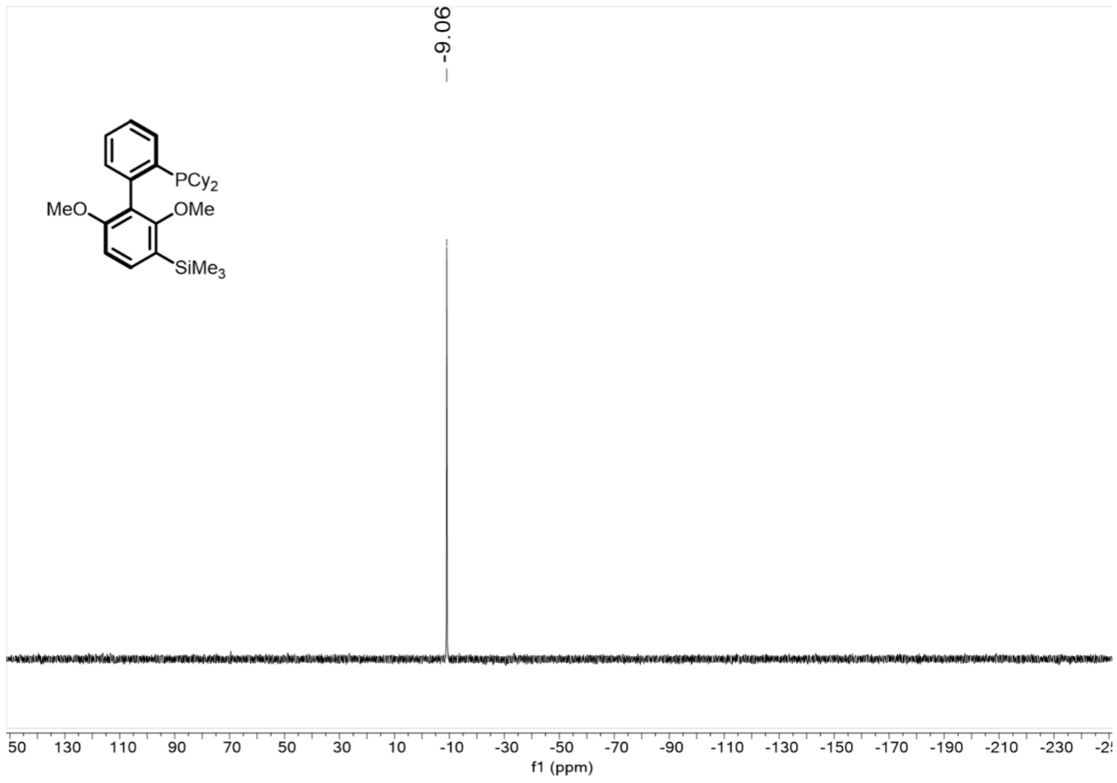
-9.06
PCy2
MeO
OMe
SiMe3
50 130 110 90 70 50 30 10 -10 -30 -50 -70 -90 -110 -130 -150 -170 -190 -210 -230
f1 (ppm)

(*R*)-dicyclohexyl(2',6'-dimethoxy-3'-methyl-[1,1'-biphenyl]-2-yl)phosphane ((*R*)-SPhos-Me); CD_2Cl_2

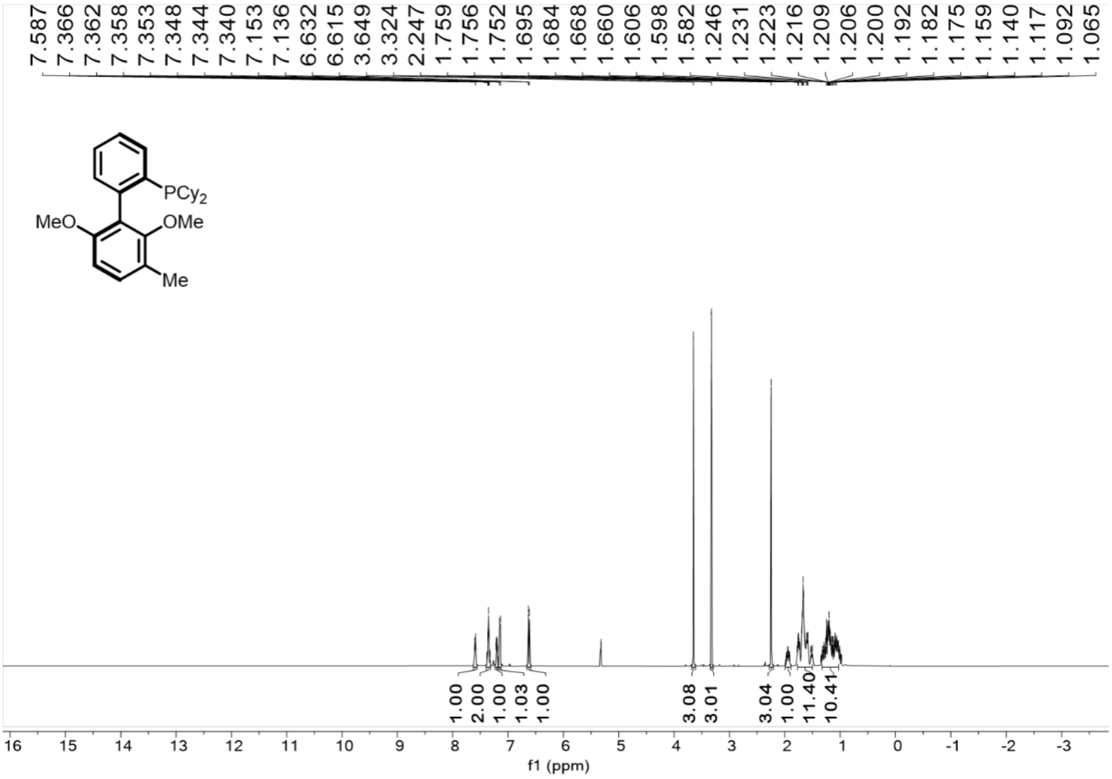

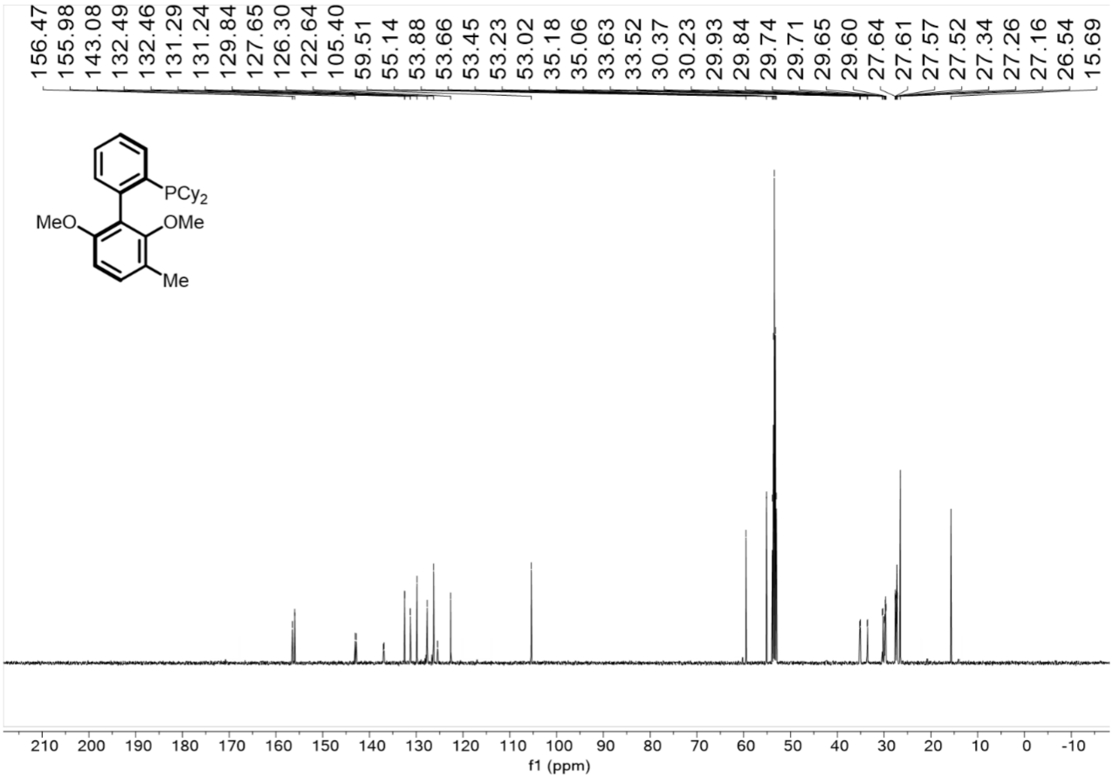

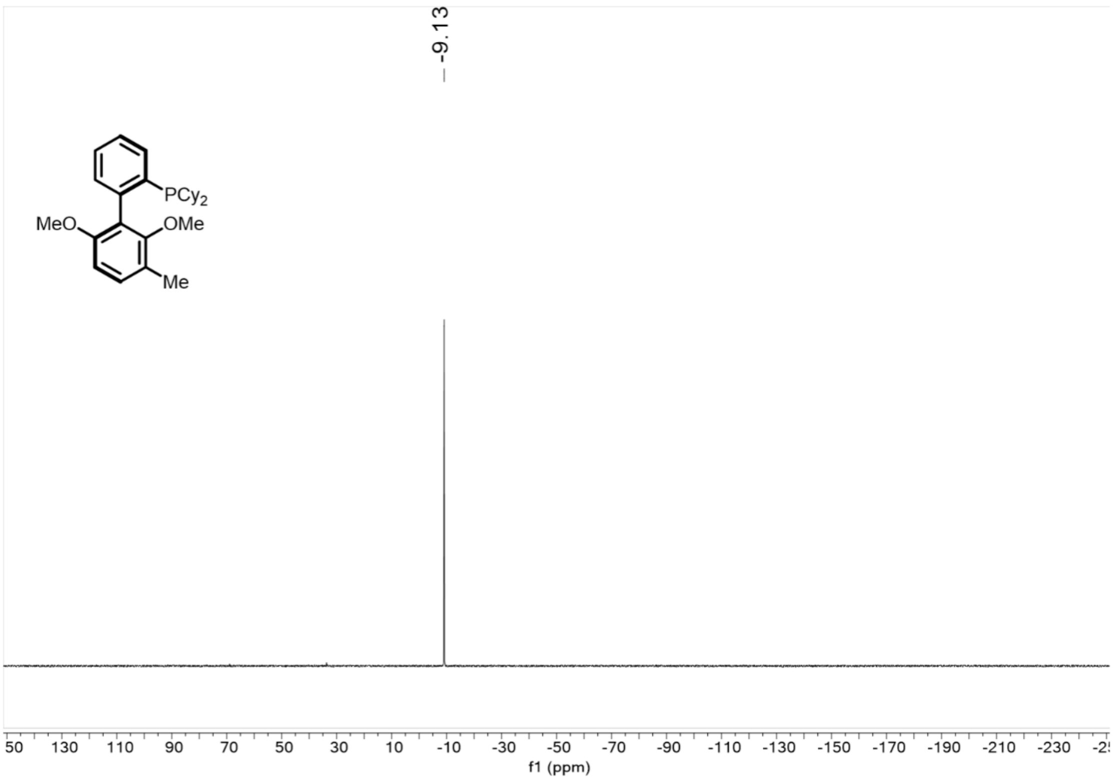
-9.13
PCy2
MeO
OMe
Me
50 130 110 90 70 50 30 10 -10 -30 -50 -70 -90 -110 -130 -150 -170 -190 -210 -230
f1 (ppm)

(*R*)-dicyclohexyl(2',6'-dimethoxy-[1,1':3',1''-terphenyl]-2-yl)phosphane ((*R*)-SPhos-Ph); CD_2Cl_2

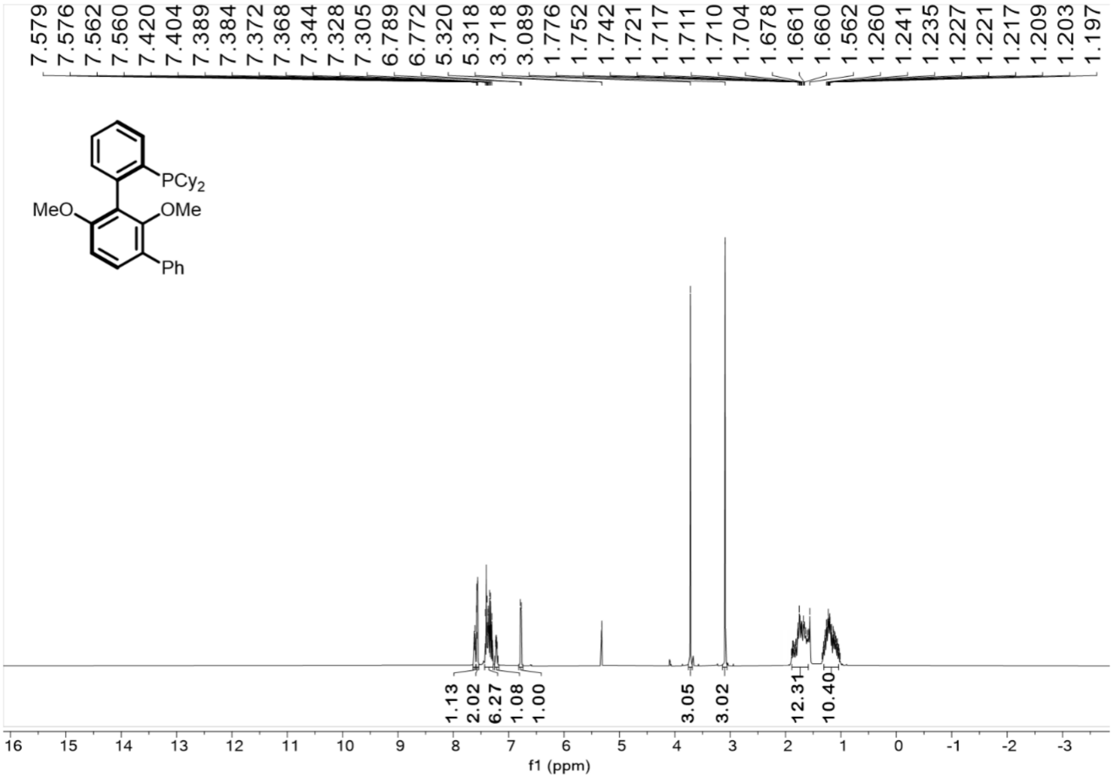

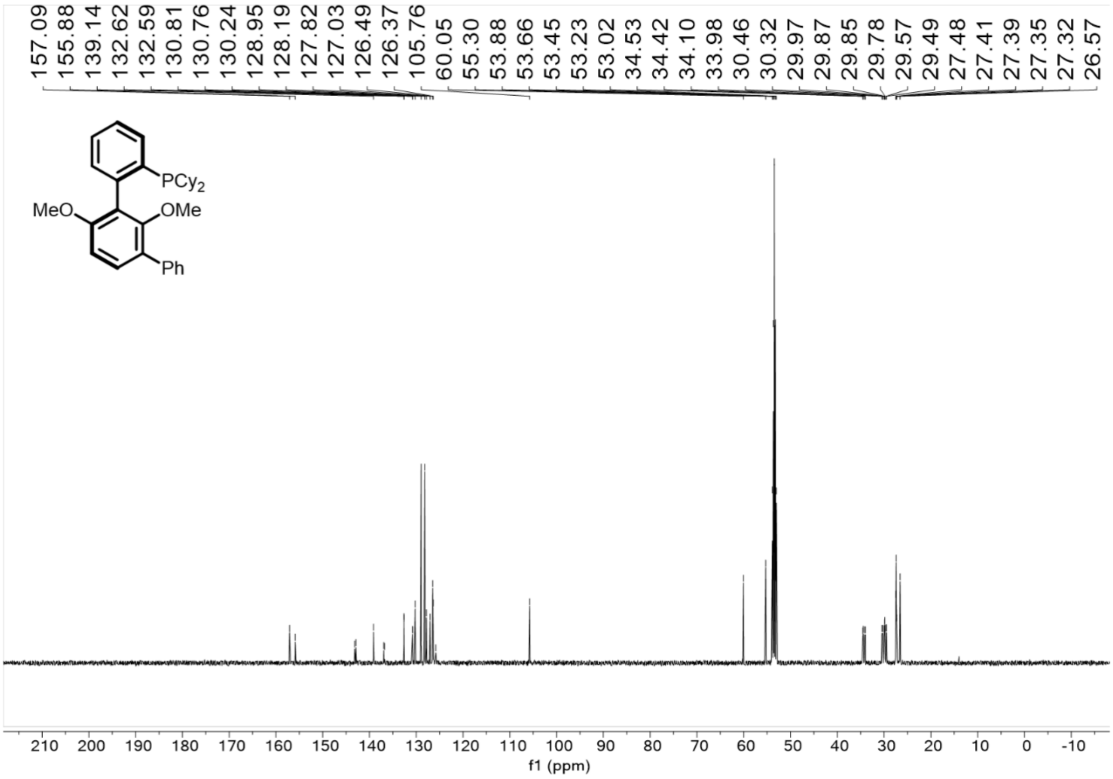

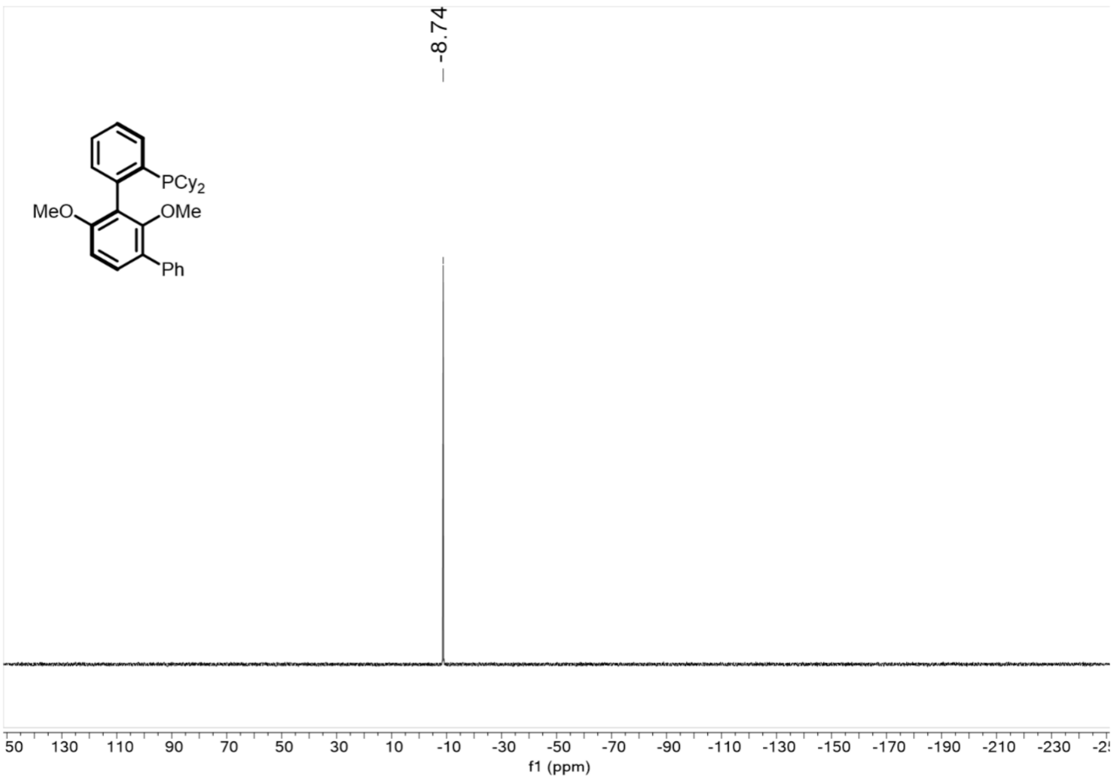

-8.74
PCy2
MeO
OMe
Ph
50 130 110 90 70 50 30 10 -10 -30 -50 -70 -90 -110 -130 -150 -170 -190 -210 -230
f1 (ppm)

(*R*)-(3'-butyl-2',6'-dimethoxy-[1,1'-biphenyl]-2-yl)dicyclohexylphosphane ((*R*)-SPhos-*ⁿ*Bu); CD_2Cl_2

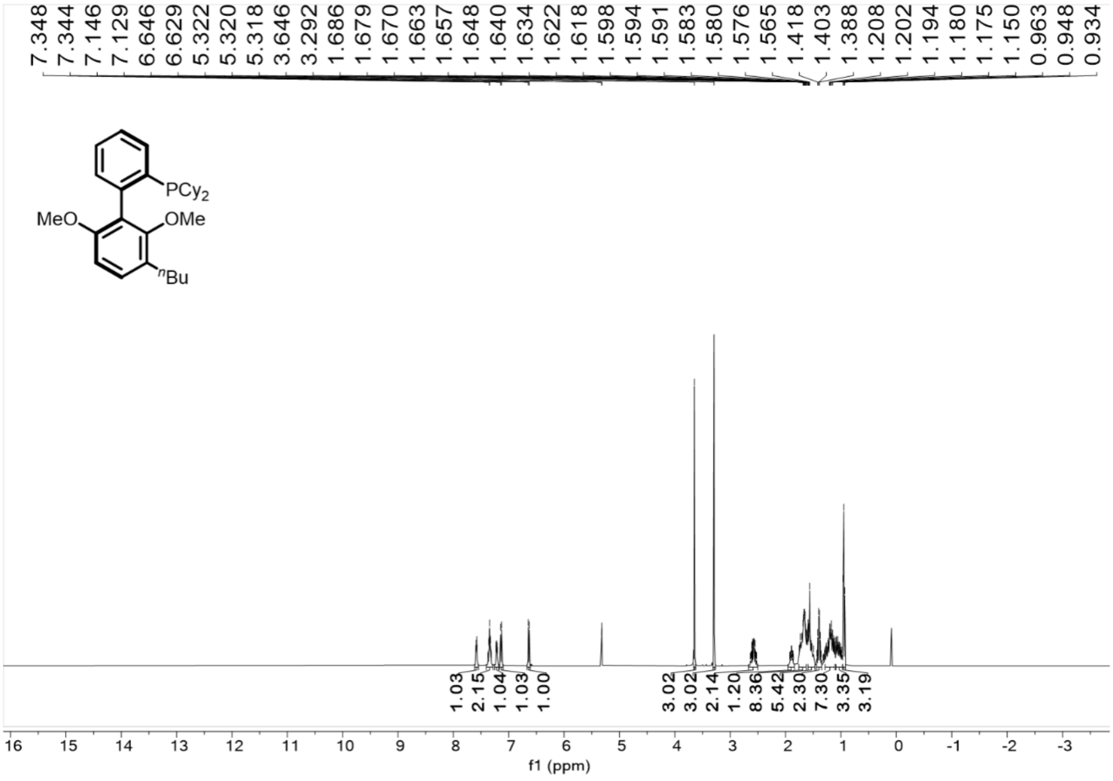

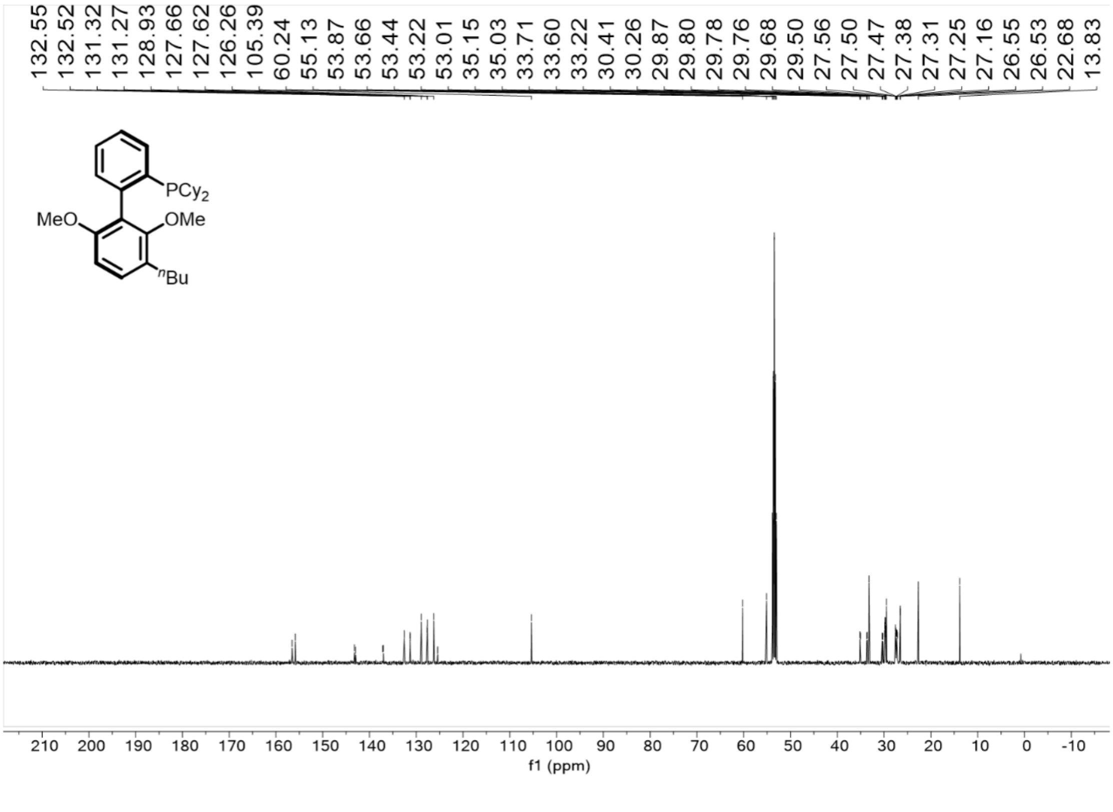

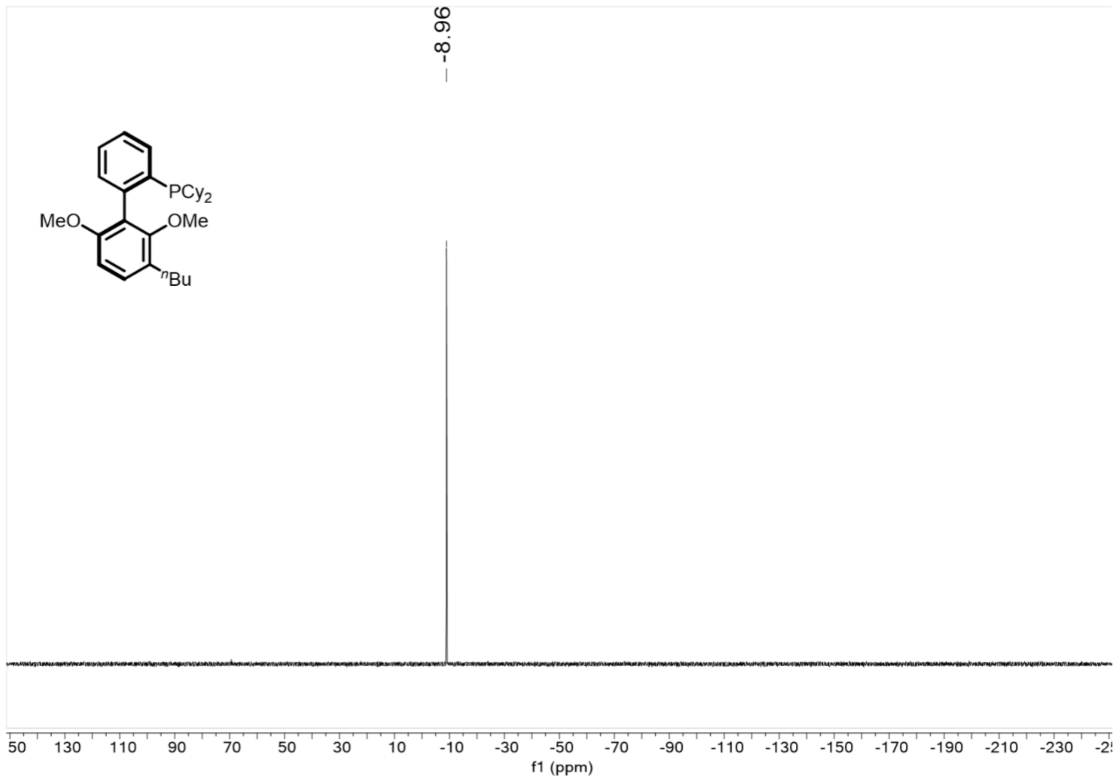
-8.96
PCy2
MeO
OMe
nBu
f1 (ppm)

(*R*)-(3'-(anthracen-9-yl)-2',6'-dimethoxy-[1,1'-biphenyl]-2-yl)dicyclohexylphosphane ((*R*)-SPhos-Ant); CD_2Cl_2

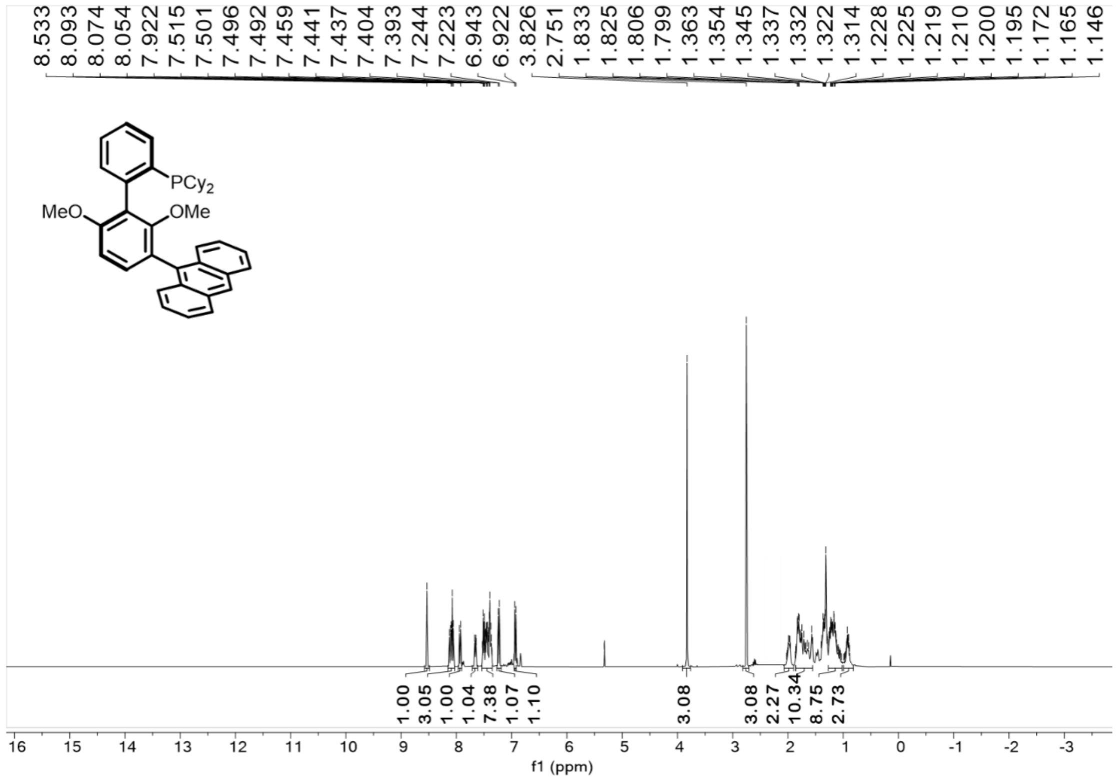

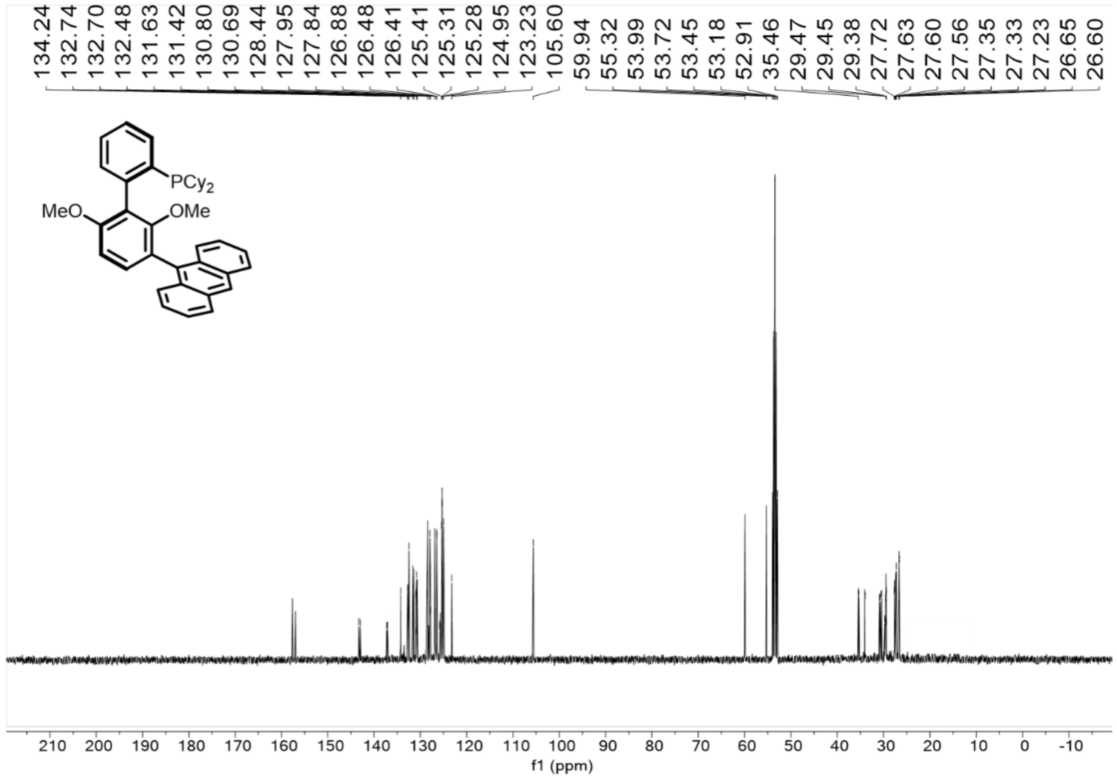

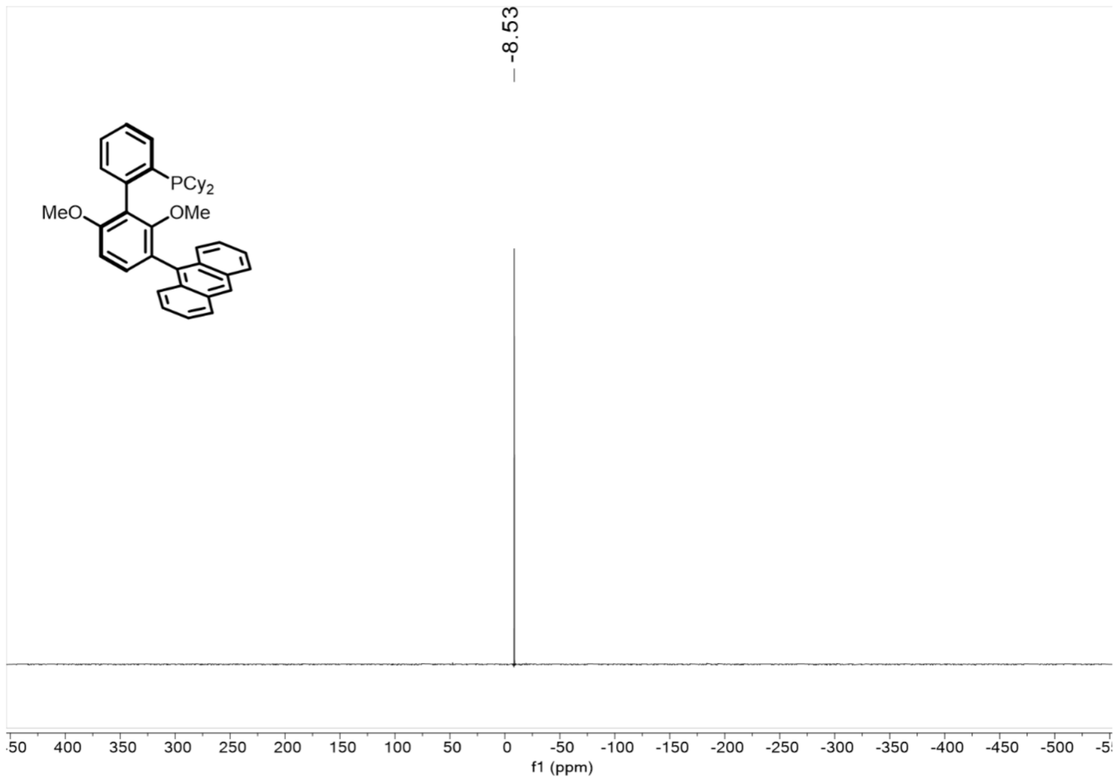

-8.53
PCy2
MeO
OMe
450 400 350 300 250 200 150 100 50 0 -50 -100 -150 -200 -250 -300 -350 -400 -450 -500
f1 (ppm)

(*S*)-1-benzyl-3-hydroxy-3-phenylindolin-2-one (45); $CDCl_3$

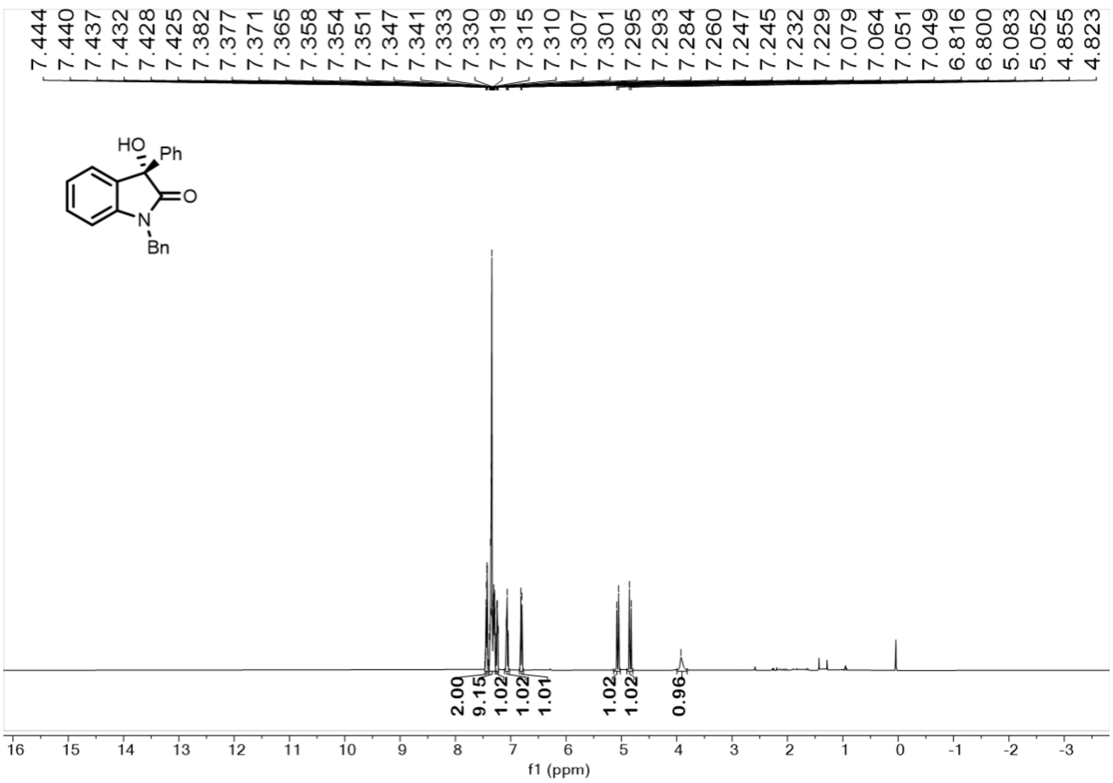

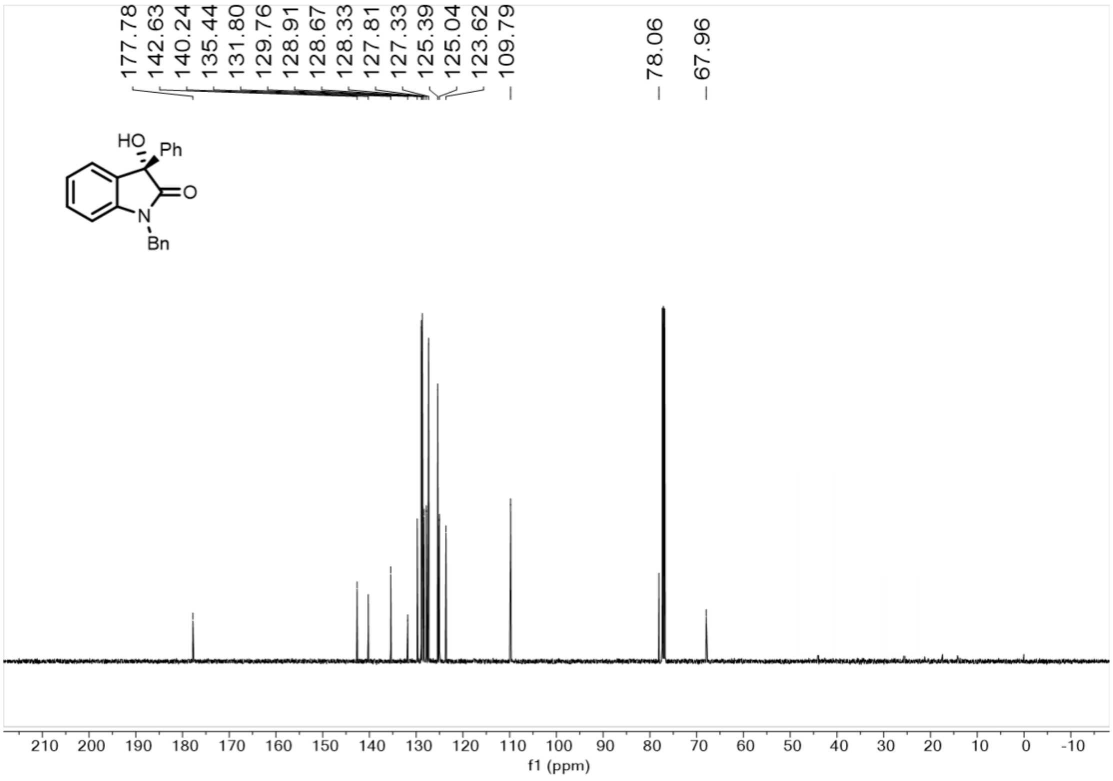

diethyl (*R*)-[1,1'-binaphthalen]-2-ylphosphonate (48); $CDCl_3$

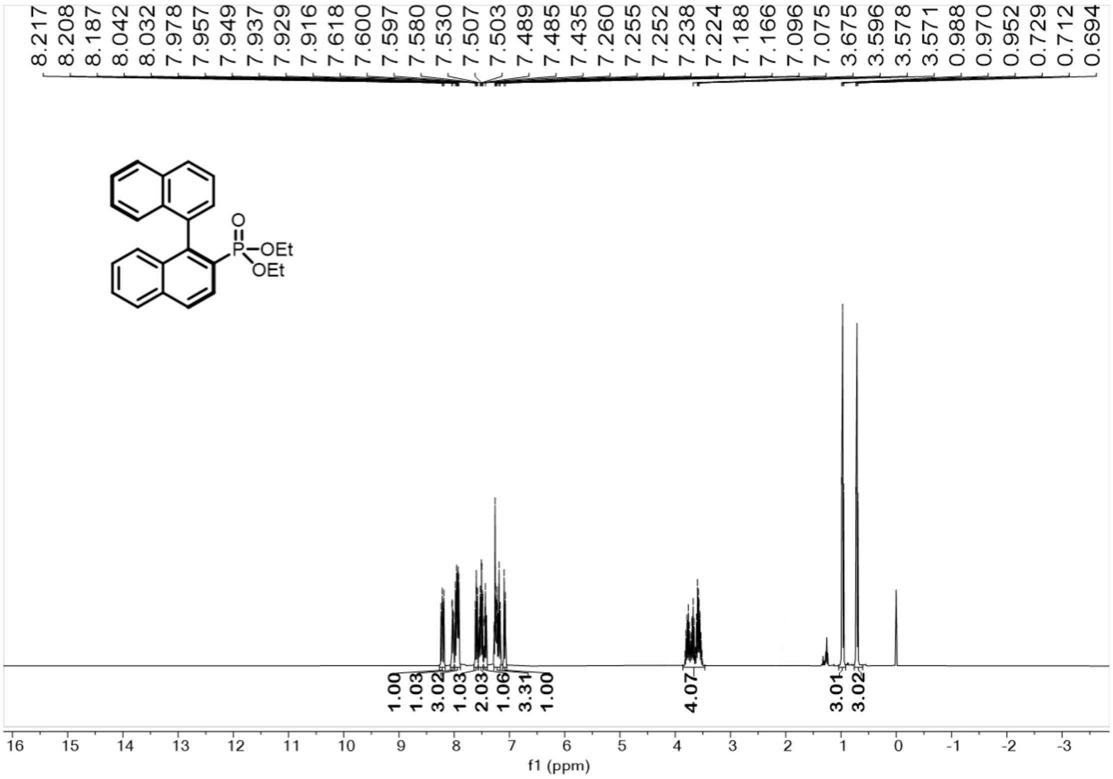

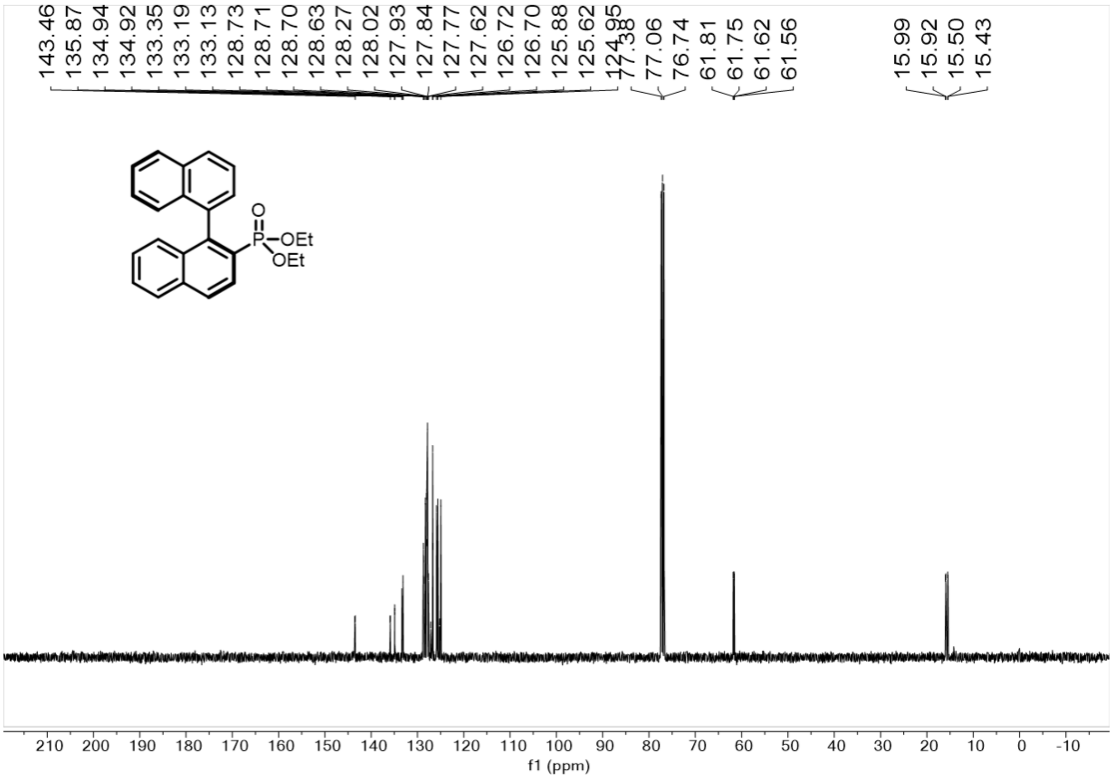

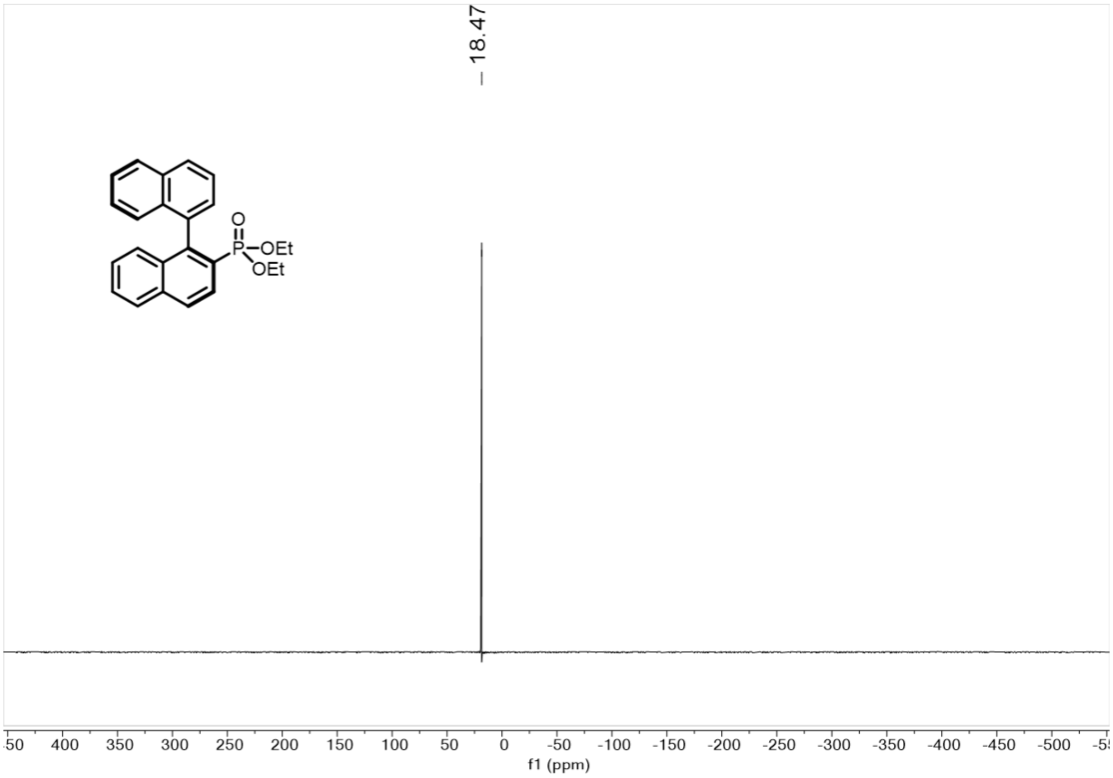
18.47
O
P
OEt
OEt
50
400
350
300
250
200
150
100
50
0
-50
-100
-150
-200
-250
-300
-350
-400
-450
-500
f1 (ppm)

dimethyl (*S*,*E*)-2-(1,3-diphenylallyl)malonate (51); $CDCl_3$

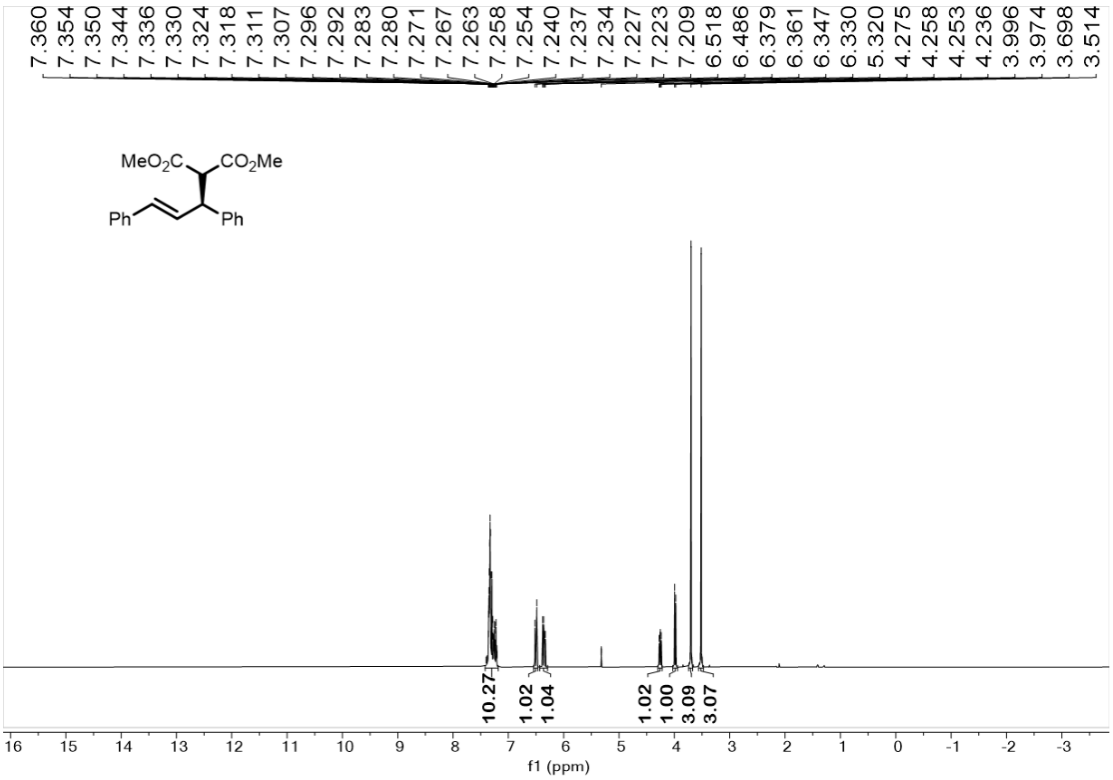

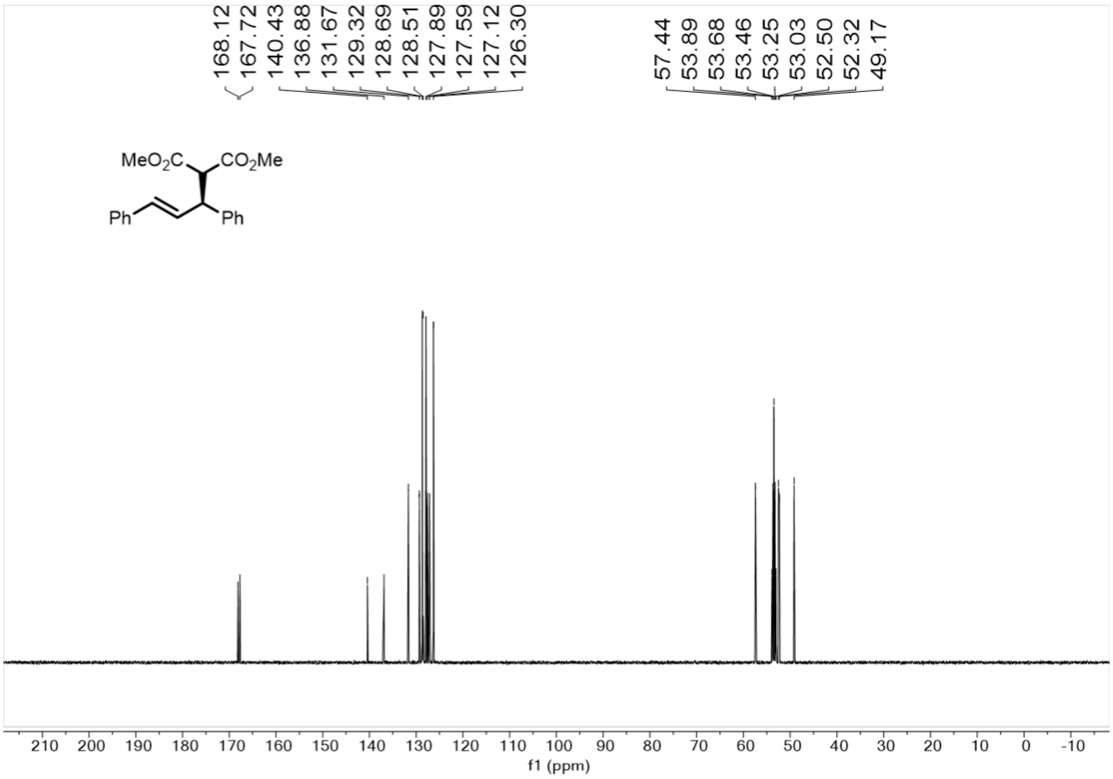

Cartesian coordinates

CPA-4

C	-2.86705700	0.50325700	-0.30857800
H	-4.43469900	1.60411800	-1.25636600
C	-3.37998000	1.60629300	-0.98901100
C	-0.70261300	1.65042700	-0.24856400
C	-2.58345300	2.68612700	-1.37319000
C	-1.51634000	0.55012600	0.03263600
C	-1.22270500	2.70236300	-1.02757500
C	1.53747300	0.59726700	-0.08547200
C	0.69696700	1.64933700	0.26919400
C	3.33614000	1.54978600	1.13515000
C	1.16715800	2.62824100	1.17116300
C	2.87022300	0.52272200	0.32005200
C	2.50680500	2.58200800	1.58452200
H	4.37460200	1.52627100	1.45974400
O	-0.95977000	-0.53394500	0.70806600
O	1.01887900	-0.42971600	-0.86773900
P	0.04120300	-1.48143900	-0.12533000
O	0.85490300	-2.04829000	1.12287700
O	-0.51342900	-2.45098700	-1.07234000
H	3.76511700	-2.47791400	4.11868200
C	4.12199500	-2.50529000	3.09370900
H	3.02102200	-0.77769300	2.52201100
C	3.71185300	-1.55251600	2.20769000
C	5.45042600	-3.60535500	1.40178500
C	4.15833100	-1.56886700	0.84342500
C	5.01156300	-3.54556700	2.68991400

C	5.03727000	-2.63169500	0.43638400
C	3.74870700	-0.60631300	-0.10157500
H	5.32834200	-4.29028500	3.41350900
H	6.12990300	-3.50783100	-1.19174400
H	6.12067400	-4.39681200	1.07660700
C	4.18342200	-0.69157800	-1.44019500
C	3.79653700	0.26189900	-2.43759300
C	5.05641300	-1.76151900	-1.83524000
H	6.15236300	-2.65880400	-3.47591600
C	5.46557600	-2.70122600	-0.88873400
C	4.23500100	0.15171700	-3.72425100
H	3.14598200	1.08344900	-2.15581500
H	3.92743900	0.88393100	-4.46449300
C	5.09610700	-0.91632700	-4.11375500
H	5.43172700	-0.98524200	-5.14385300
C	5.49423600	-1.84050400	-3.19563800
H	-3.56141300	0.28087700	4.55543300
C	-3.94100300	-0.34763200	3.75554600
H	-2.93969100	0.75123800	2.23870200
C	-3.59129700	-0.08713200	2.46331400
C	-5.29347600	-2.23014900	3.07862100
C	-4.07372100	-0.90036500	1.38625400
C	-4.80325300	-1.43841100	4.07283200
C	-4.95183000	-1.98993300	1.70955700
C	-3.73268800	-0.65951800	0.03969400
H	-5.06694800	-1.62945700	5.10851400
H	-6.13652100	-3.60621000	0.93579900
H	-5.95564000	-3.06245300	3.30301500

C	-4.24009000	-1.48466600	-0.98399800
C	-3.88817300	-1.31149600	-2.36349500
C	-5.13126400	-2.56092600	-0.64801700
H	-6.33619700	-4.18871300	-1.42007200
C	-5.46452100	-2.78756600	0.68653800
C	-4.40699100	-2.12241100	-3.32921000
H	-3.18375000	-0.53141800	-2.63138100
H	-4.11937100	-1.97890000	-4.36621400
C	-5.31389300	-3.17106400	-2.99397300
H	-5.71622400	-3.80290300	-3.77986400
C	-5.65795100	-3.38457800	-1.69361900
C	3.08752400	3.60090500	2.54720400
H	3.25638800	3.10768300	3.51419600
H	4.07485200	3.91079600	2.18641400
C	0.21235400	3.66536900	1.73733400
H	-0.75176000	3.18614700	1.94062700
H	0.00853200	4.44339000	0.98978800
C	0.75259700	4.33894900	2.99789500
H	0.10012700	5.17376100	3.27237600
H	0.74061300	3.62916700	3.83528900
C	2.18304500	4.81162800	2.75835500
H	2.55048900	5.40783200	3.59944900
H	2.20634700	5.45624900	1.86959900
C	-0.30480500	3.76640900	-1.60189100
H	0.52104600	3.98686500	-0.92358900
H	0.16153300	3.33698800	-2.50094600
C	-3.22941300	3.83159400	-2.13054100
H	-3.99194500	3.43259900	-2.80798500

H -3.76363700 4.46183500 -1.40585200

C -1.04401200 5.04203300 -1.99741500

H -1.40417500 5.55828400 -1.09681100

H -0.35458600 5.72312400 -2.50658800

C -2.22955800 4.69652300 -2.89449300

H -1.86528700 4.15271300 -3.77563700

H -2.72599800 5.60230800 -3.25707200

H 1.38223800 -2.83413800 0.89700400

Zero-point correction= 0.733649 (Hartree/Particle)

Thermal correction to Energy= 0.763246

Thermal correction to Enthalpy= 0.764048

Thermal correction to Gibbs Free Energy= 0.676470

Sum of electronic and zero-point Energies= -2492.222594

Sum of electronic and thermal Energies= -2492.192997

Sum of electronic and thermal Enthalpies= -2492.192195

Sum of electronic and thermal Free Energies= -2492.279773

AP-8

C -0.00008500 1.26671400 0.00000000

C -0.00312200 -1.53261500 -0.00000000

C 1.19111500 0.53232400 0.00000000

C -1.19841100 0.54220000 -0.00000000

C -1.21066900 -0.84457000 -0.00000000

C 1.20785200 -0.85534400 0.00000000

H -2.15375800 -1.37821900 -0.00000000

H 2.14911300 -1.39201700 0.00000000

Br 2.82756100 1.48696200 0.00000000

Br -2.82715500 1.48799000 -0.00000000

Br -0.01062200 -3.42313200 -0.00000000

O -0.05479800 2.60719900 0.00000000

H 0.84842000 2.96927200 0.00000000

Zero-point correction= 0.076042 (Hartree/Particle)

Thermal correction to Energy= 0.083381

Thermal correction to Enthalpy= 0.084182

Thermal correction to Gibbs Free Energy= 0.044973

Sum of electronic and zero-point Energies= -8020.814216

Sum of electronic and thermal Energies= -8020.806877

Sum of electronic and thermal Enthalpies= -8020.806076

Sum of electronic and thermal Free Energies= -8020.845285

NBS

Br 0.00000000 0.00000000 1.67817400

N 0.00000000 -0.00000000 -0.15658900

C 0.00000000 1.18362300 -0.90420800

C -0.00000000 -1.18362300 -0.90420800

O -0.00000000 -2.29096100 -0.43959500

O 0.00000000 2.29096100 -0.43959500

C -0.00000000 -0.76690600 -2.36517200

H 0.88201100 -1.19933100 -2.84346900

H -0.88201100 -1.19933100 -2.84346900

C 0.00000000 0.76690600 -2.36517200

H -0.88201100 1.19933100 -2.84346900

H 0.88201100 1.19933100 -2.84346900

Zero-point correction= 0.082766 (Hartree/Particle)

Thermal correction to Energy= 0.088405

Thermal correction to Enthalpy= 0.089207

Thermal correction to Gibbs Free Energy= 0.055769

Sum of electronic and zero-point Energies= -2931.590031

Sum of electronic and thermal Energies= -2931.584392

Sum of electronic and thermal Enthalpies= -2931.583590

Sum of electronic and thermal Free Energies= -2931.617028

(*R*)-1

C	-1.05831100	0.66414500	1.19930600
C	-1.51644300	2.98698400	-0.30467600
C	-2.36887300	1.03763000	0.83792700
C	-0.00148500	1.50456900	0.85084700
C	-0.23116000	2.65327200	0.08628600
C	-2.59238400	2.19074000	0.08334000
H	-3.59343500	2.47505000	-0.21767100
H	-1.68630100	3.87566500	-0.90277600
C	-0.81283200	-0.61598200	1.92812500
C	-0.47971100	-2.97080900	3.41222700
C	-0.27743200	-1.76239100	1.30757200
C	-1.17836400	-0.67489700	3.27668700
C	-1.00546400	-1.83670800	4.02137400
C	-0.12914800	-2.92999100	2.06654400
H	-1.61022200	0.20887200	3.73885600
H	-1.29085300	-1.85556900	5.06885800
H	0.25678100	-3.81670700	1.57202700
H	-0.35031500	-3.88853300	3.97774400
P	0.29488200	-1.96817800	-0.43954400
O	0.36281000	-3.43611700	-0.77242300
C	1.94606100	-1.16175700	-0.55789400

C	4.11171700	-0.63700700	0.61676000
C	4.10421100	-1.05572100	-1.86827400
C	4.91423800	-1.16114500	-0.57532900
C	2.74435300	-1.74912700	-1.73790100
C	2.76164200	-1.34700700	0.73405900
H	3.92780900	0.43997300	0.48982800
H	3.94461900	0.00455000	-2.10995900
H	5.17004900	-2.21493500	-0.39776300
H	2.88419700	-2.82198500	-1.55742000
H	2.92362300	-2.42267900	0.89860200
H	1.78568300	-0.08564900	-0.71862600
H	4.68281100	-0.76022200	1.54461500
H	4.66325800	-1.49188600	-2.70398700
H	5.85962200	-0.61521700	-0.67184500
H	2.19313000	-1.66555700	-2.67861700
H	2.21028900	-0.96616700	1.59643900
C	-0.89295700	-1.06288100	-1.50996200
C	-1.54159400	-0.44776900	-3.87655600
C	-3.32785000	-0.96526300	-2.18859000
C	-2.93114500	-1.04761300	-3.66282000
C	-2.29471300	-1.66082400	-1.30102400
C	-0.48754300	-1.12200800	-2.99184600
H	-1.57315500	0.62433900	-3.63421600
H	-3.39408900	0.09365000	-1.89570400
H	-2.92434400	-2.10084500	-3.97536300
H	-2.25584300	-2.73006900	-1.54911900
H	-0.36008100	-2.17074300	-3.29189900
H	-0.90029900	-0.00823000	-1.20787600

H -1.24561100 -0.52785700 -4.92851300

H -4.31809600 -1.40781600 -2.02836800

H -3.66816600 -0.53235300 -4.28913000

H -2.58836300 -1.58552400 -0.24827700

H 0.47403600 -0.61924300 -3.13857100

O -3.35285500 0.20689900 1.25374000

O 1.27036000 1.18023600 1.22374500

C -4.69035800 0.54929100 0.93982000

H -4.96558400 1.51336900 1.38338500

H -5.30767100 -0.23797000 1.37267800

H -4.85371000 0.58254000 -0.14308000

C 1.65072200 1.72608600 2.48447000

H 2.68475900 1.42044000 2.65354900

H 1.01432400 1.33116500 3.28456600

H 1.59098400 2.82019700 2.46567500

Br 1.24181100 3.72650200 -0.41223400

Zero-point correction= 0.561966 (Hartree/Particle)

Thermal correction to Energy= 0.584275

Thermal correction to Enthalpy= 0.585077

Thermal correction to Gibbs Free Energy= 0.513689

Sum of electronic and zero-point Energies= -4148.977821

Sum of electronic and thermal Energies= -4148.955511

Sum of electronic and thermal Enthalpies= -4148.954709

Sum of electronic and thermal Free Energies= -4149.026097

S1

C -1.27372300 -1.26635700 0.79268500

C -2.39823700 -0.00641800 3.01557700

C	-2.62171800	-0.87506600	0.78623000
C	-0.52579400	-1.07547700	1.96182900
C	-1.07445300	-0.42820800	3.07342100
C	-3.18771700	-0.23438200	1.89373900
H	-0.48942100	-0.25780700	3.96857100
H	-4.22170000	0.08768700	1.88460100
H	-2.83086900	0.49986700	3.87313100
C	-0.66529700	-1.88168200	-0.42337000
C	0.32412200	-3.15461100	-2.72064200
C	0.18716800	-1.18169600	-1.29984200
C	-1.00498900	-3.20570900	-0.71824500
C	-0.51103100	-3.84559100	-1.84904800
C	0.65998900	-1.83339700	-2.44703300
H	-1.67384800	-3.73183300	-0.04304300
H	-0.78656800	-4.87634400	-2.05157200
H	1.28677400	-1.27347600	-3.13506200
H	0.70658800	-3.63684300	-3.61511700
P	0.81764100	0.54758800	-1.16135100
O	1.24812800	1.03088800	-2.52273800
C	2.24875100	0.52354300	0.01037500
C	4.29364900	-0.76587000	0.78735600
C	4.41475600	1.72957100	0.57757000
C	5.19650800	0.42227400	0.45894400
C	3.17456300	1.71200900	-0.31923300
C	3.04180000	-0.79155400	-0.09540100
H	3.98972200	-0.69292600	1.84118300
H	4.10896000	1.87572000	1.62373400
H	5.57258400	0.31974300	-0.56820800

H	3.47911900	1.61998500	-1.36859900
H	3.34626900	-0.93469300	-1.14276600
H	1.85437100	0.62339800	1.03369200
H	4.84082000	-1.70944600	0.67905000
H	5.04914900	2.58235100	0.31061700
H	6.07014400	0.43355700	1.12055500
H	2.64189600	2.66365800	-0.23911200
H	2.40551700	-1.63917800	0.17360000
C	-0.51673500	1.58720100	-0.44636200
C	-1.20346300	3.89432600	0.32465000
C	-2.85249300	2.44972300	-0.90169700
C	-2.38074400	3.88224400	-0.65023200
C	-1.70889500	1.57544000	-1.41857900
C	-0.04469600	3.02464700	-0.17427900
H	-1.54087500	3.50936800	1.29787500
H	-3.22710300	2.02602700	0.04197500
H	-2.06763900	4.33157500	-1.60274300
H	-1.36567000	1.95371500	-2.39065900
H	0.37472300	3.45241000	-1.09491500
H	-0.82525500	1.15057500	0.51073900
H	-0.85157300	4.91878300	0.49057300
H	-3.68467300	2.43713800	-1.61553300
H	-3.20211900	4.49498200	-0.26171400
H	-2.05730900	0.54885100	-1.57771700
H	0.75071000	3.02358700	0.57907000
O	-3.30474600	-1.14651300	-0.35393700
O	0.74314300	-1.55703800	1.92283800
C	-4.66872400	-0.77767300	-0.41519600

H -5.25526500 -1.29464100 0.35350700

H -5.01620300 -1.08524500 -1.40185700

H -4.79907000 0.30516800 -0.30810000

C 1.51152600 -1.49235100 3.10767400

H 2.43587900 -2.03077400 2.89646100

H 0.99433400 -1.98099600 3.94159400

H 1.74605100 -0.45607600 3.38179600

Zero-point correction= 0.572513 (Hartree/Particle)

Thermal correction to Energy= 0.593351

Thermal correction to Enthalpy= 0.594152

Thermal correction to Gibbs Free Energy= 0.527367

Sum of electronic and zero-point Energies= -1577.784606

Sum of electronic and thermal Energies= -1577.763768

Sum of electronic and thermal Enthalpies= -1577.762966

Sum of electronic and thermal Free Energies= -1577.829751

Succinimide

N 0.00000000 -0.00000000 0.95965200

C 0.00000000 1.16909400 0.21288000

C -0.00000000 -1.16909400 0.21288000

O -0.00000000 -2.28478000 0.67187600

O 0.00000000 2.28478000 0.67187600

C -0.00000000 -0.76608100 -1.25516500

H 0.88096800 -1.20097600 -1.73354100

H -0.88096800 -1.20097600 -1.73354100

C 0.00000000 0.76608100 -1.25516500

H -0.88096800 1.20097600 -1.73354100

H 0.88096800 1.20097600 -1.73354100

H 0.00000000 -0.00000000 1.97399900

Zero-point correction= 0.093476 (Hartree/Particle)

Thermal correction to Energy= 0.097970

Thermal correction to Enthalpy= 0.098771

Thermal correction to Gibbs Free Energy= 0.069228

Sum of electronic and zero-point Energies= -360.436398

Sum of electronic and thermal Energies= -360.431904

Sum of electronic and thermal Enthalpies= -360.431103

Sum of electronic and thermal Free Energies= -360.460646

AP-8-TCP1

C -2.18707500 -3.06645600 -0.67380500

H -2.90199400 -4.78407100 -1.72957200

C -3.16167200 -3.79942300 -1.34624800

C -3.86120800 -1.31331000 -0.30115000

C -4.44536800 -3.29639600 -1.57196600

C -2.56357000 -1.81896700 -0.18185500

C -4.79729000 -2.03253300 -1.06989500

C -3.42066900 1.10429000 0.02843300

C -4.18217500 -0.01193400 0.36206200

C -4.65852600 2.48697900 1.50127200

C -5.15755900 0.11360300 1.37530400

C -3.63472500 2.37259500 0.56784300

C -5.40369500 1.38057000 1.92692200

H -4.86165400 3.46254100 1.93845600

O -1.60438300 -1.06155200 0.47547600

O -2.37714100 0.93613300 -0.87710800

P -1.05357500 0.27659000 -0.24827100

O	-0.33621300	1.06211700	0.76858900
O	-0.34840300	-0.12043500	-1.58642700
H	-0.13854500	3.33075400	3.90776900
C	-0.35253500	3.79460100	2.94988000
H	-2.05574200	2.57656900	2.58353900
C	-1.42257400	3.37730600	2.21668900
C	0.27398700	5.39694500	1.25247300
C	-1.71107100	3.95289700	0.93494200
C	0.51446600	4.81848000	2.46069300
C	-0.84156600	4.99002900	0.45122400
C	-2.77787900	3.51401200	0.12877000
H	1.37069800	5.12140700	3.05538600
H	-0.40428300	6.33167200	-1.16751700
H	0.93093500	6.16977700	0.86147300
C	-3.00735900	4.09580000	-1.13461900
C	-4.08262700	3.68325600	-1.98711300
C	-2.13833500	5.13811700	-1.60259500
H	-1.70943000	6.50899200	-3.22543200
C	-1.07213800	5.55368100	-0.80278800
C	-4.27812500	4.26442300	-3.20581400
H	-4.75080800	2.89861200	-1.64619000
H	-5.10046600	3.93817100	-3.83533400
C	-3.41326800	5.30017600	-3.66652000
H	-3.58591400	5.74911100	-4.63986500
C	-2.37750100	5.72102800	-2.88721400
H	-1.76090800	-4.69356800	3.93192500
C	-1.04685300	-4.68841200	3.11403900
H	-2.40187000	-3.76927800	1.75678000

C	-1.40444200	-4.17161600	1.90304800
C	1.16623900	-5.22451600	2.30655700
C	-0.47928500	-4.14516500	0.80963000
C	0.25916500	-5.22033600	3.32364900
C	0.83056900	-4.69523500	1.02137800
C	-0.80997200	-3.59899100	-0.44792900
H	0.52496300	-5.61894300	4.29774700
H	2.74703800	-5.11107200	0.14378800
H	2.16741000	-5.62124800	2.45234000
C	0.14013900	-3.57567800	-1.48541600
C	-0.13506600	-2.99156800	-2.76589800
C	1.44195600	-4.14568600	-1.26614800
H	3.36477900	-4.59558500	-2.16450800
C	1.75733500	-4.69006700	-0.02210700
C	0.80100800	-2.99490300	-3.75733600
H	-1.10254800	-2.52937900	-2.93066000
H	0.57592300	-2.53986500	-4.71665000
C	2.08182700	-3.58779600	-3.54659700
H	2.80408300	-3.60017100	-4.35827900
C	2.39332600	-4.13580400	-2.33705600
H	0.64105000	-0.23011900	-1.57884100
C	3.15527900	-0.99703800	-1.63005100
N	3.18652100	-1.50948500	-0.35002500
C	4.38804200	-2.14760800	0.00559900
Br	1.77038400	-1.37656500	0.80443100
O	4.59504300	-2.65052500	1.07497100
O	2.22070300	-0.40174600	-2.12727400
C	5.29461800	-2.05745300	-1.20912600

H	6.20804500	-1.52538100	-0.92781200
H	5.57680800	-3.07123600	-1.50420300
C	4.48609600	-1.31365000	-2.27810600
H	4.95922900	-0.38156900	-2.59703800
H	4.28865800	-1.91293500	-3.16943900
C	5.39546400	1.36116300	-0.37586800
C	3.03621000	1.59150700	1.12665200
C	4.28408800	1.97079000	-0.94067700
C	5.35643500	0.84150800	0.91285200
C	4.17935100	0.95131500	1.63854200
C	3.11936200	2.07697100	-0.18875700
H	4.31441300	2.36258300	-1.95100800
H	6.21874100	0.34726000	1.34604100
O	1.96959300	1.71093900	1.91142200
H	1.12746100	1.75886400	1.39874300
Br	4.07485900	0.15945300	3.33997400
Br	1.60181000	2.88817400	-0.95907600
Br	6.98974500	1.21497200	-1.39037500
C	-6.43428000	1.59296700	3.02033300
H	-5.90411100	1.80039500	3.95980800
H	-7.01527100	2.49394300	2.79292200
C	-5.86582500	-1.12024300	1.90772100
H	-5.14595900	-1.94427700	1.96540200
H	-6.64168900	-1.44814500	1.20304900
C	-6.52060100	-0.88382400	3.26774900
H	-7.13554100	-1.75005000	3.53153800
H	-5.74838400	-0.78623700	4.04184500
C	-7.35457700	0.39275000	3.22360300

H -7.93356200 0.52058700 4.14358500

H -8.07272700 0.32673400 2.39543600

C -6.11555700 -1.39524100 -1.46980200

H -6.48782400 -0.73244200 -0.68717600

H -5.90844700 -0.74319100 -2.33116400

C -5.43819300 -4.15789000 -2.33083600

H -4.91113400 -4.71187700 -3.11508600

H -5.83556900 -4.91267900 -1.63784400

C -7.17549900 -2.41662000 -1.87335900

H -7.49446100 -2.99028800 -0.99226000

H -8.05882100 -1.89702600 -2.25829300

C -6.60413500 -3.36801000 -2.92118800

H -6.25808500 -2.78402200 -3.78378200

H -7.37104200 -4.05873100 -3.28607900

Zero-point correction= 0.894919 (Hartree/Particle)

Thermal correction to Energy= 0.939380

Thermal correction to Enthalpy= 0.940182

Thermal correction to Gibbs Free Energy= 0.819078

Sum of electronic and zero-point Energies= -13444.701167

Sum of electronic and thermal Energies= -13444.656706

Sum of electronic and thermal Enthalpies= -13444.655904

Sum of electronic and thermal Free Energies= -13444.777007

AP-8-TCP2

H -0.76256700 -0.55425400 1.33473600

C -3.27752400 0.09192100 2.03553700

N -3.31862900 1.39013100 1.57461000

C -4.54551900 2.05003500 1.78655600

Br	-1.95438700	2.16957400	0.60972100
O	-4.79748600	3.15139400	1.38438900
O	-2.35642500	-0.68717300	1.89032600
C	-5.41333600	1.10012000	2.59060000
H	-6.34479500	0.92855700	2.04491800
H	-5.66424000	1.58257300	3.53841200
C	-4.58177900	-0.17608100	2.75889400
H	-5.05732200	-1.05665300	2.31873400
H	-4.34837400	-0.40913200	3.80110600
C	-5.57573800	-0.49701000	-0.51325000
C	-3.20549000	0.39910800	-1.71485500
C	-4.46994700	-1.33450800	-0.54886300
C	-5.52407100	0.79079800	-1.03564400
C	-4.33994900	1.22344600	-1.61503200
C	-3.29829300	-0.87833500	-1.14196100
H	-4.50772500	-2.32981500	-0.12038500
H	-6.37915200	1.45465100	-0.97721400
O	-2.12910700	0.86515200	-2.34500500
H	-1.28735000	0.54546800	-1.93318200
Br	-4.19912100	3.00280000	-2.21054500
Br	-1.78831900	-2.01333600	-1.13572000
Br	-7.17582100	-1.11679600	0.29152100
C	2.36887100	-3.24788000	0.49298500
H	3.28024300	-4.84581700	1.58413400
C	3.43624200	-3.86384300	1.14228600
C	3.86076000	-1.36874200	-0.01789600
C	4.68084600	-3.24506200	1.28081600
C	2.61906600	-2.00016300	-0.07074800

C	4.89491600	-1.97159900	0.72618500
C	3.21102000	1.01901600	-0.24727600
C	4.02123700	-0.03394300	-0.67245600
C	4.43523400	2.55896300	-1.57227200
C	4.96132700	0.19894300	-1.69778400
C	3.43945800	2.34501500	-0.62376500
C	5.15872000	1.51091100	-2.15175300
H	4.64719600	3.58133300	-1.87834400
O	1.58160700	-1.35114600	-0.72530700
O	2.16242600	0.73237400	0.62345500
P	0.91717600	-0.07128700	-0.01160800
O	0.12870600	0.65831100	-1.02237400
O	0.23204200	-0.54149400	1.31243100
H	-1.07400600	3.35574600	-2.44710200
C	-0.49792400	3.83814800	-1.66252300
H	1.20312600	2.59270600	-1.94049600
C	0.76481200	3.40445100	-1.37285000
C	-0.38948700	5.51101600	0.08222600
C	1.51309300	3.97253700	-0.29285400
C	-1.08518200	4.91089300	-0.92398500
C	0.92261800	5.06175900	0.43722100
C	2.78018800	3.48829100	0.08223700
H	-2.09815900	5.22451900	-1.15831300
H	1.18009300	6.47439200	2.03405100
H	-0.82438700	6.32813800	0.65165600
C	3.47752900	4.07804200	1.15821200
C	4.76235400	3.61390600	1.59768300
C	2.88914100	5.18467000	1.86171300

H	3.14483000	6.62494900	3.46038700
C	1.62753100	5.64647200	1.48800000
C	5.41105400	4.21306500	2.63742500
H	5.21879700	2.76967200	1.09114500
H	6.38230900	3.84375000	2.95265900
C	4.82894900	5.32041300	3.32206400
H	5.36405200	5.78356600	4.14532600
C	3.60581100	5.78632400	2.94489600
H	1.46483400	-4.74130400	-4.10537200
C	0.87560400	-4.83975500	-3.19887900
H	2.30759500	-3.82768200	-1.99776000
C	1.34479300	-4.32790400	-2.02496400
C	-1.13579000	-5.63696500	-2.12449000
C	0.58126800	-4.42954600	-0.81639000
C	-0.38507500	-5.50399300	-3.25360000
C	-0.68375100	-5.10861300	-0.87408600
C	1.02241200	-3.88547300	0.40783300
H	-0.74109800	-5.89955000	-4.19975500
H	-2.42441400	-5.71862000	0.22680300
H	-2.09943100	-6.13908100	-2.15080300
C	0.20927300	-3.96158400	1.55550500
C	0.58379000	-3.36711400	2.80581000
C	-1.05677300	-4.63844900	1.48264300
H	-2.82902600	-5.23093400	2.58104800
C	-1.46557000	-5.20656300	0.27732800
C	-0.22959900	-3.45376200	3.89661400
H	1.52122200	-2.82483400	2.86682100
H	0.06880200	-2.98806400	4.83085100

C	-1.47843600	-4.14036800	3.82428700
H	-2.10840700	-4.19928100	4.70672500
C	-1.87767300	-4.70992500	2.65343100
C	6.13915700	1.83512000	-3.26216600
H	5.56818200	2.07095800	-4.17057900
H	6.68758600	2.74721600	-3.00095100
C	5.69914800	-0.96995400	-2.32765100
H	5.00593500	-1.81192200	-2.43218100
H	6.49732400	-1.31979500	-1.65910400
C	6.31874600	-0.61485900	-3.67861700
H	6.96273400	-1.43513000	-4.01100700
H	5.52838000	-0.49796500	-4.43134200
C	7.10159100	0.68977500	-3.56433700
H	7.65386300	0.90183000	-4.48519500
H	7.84050600	0.59899100	-2.75682600
C	6.16395200	-1.20365200	1.05170800
H	6.45399800	-0.54846200	0.22880600
H	5.92680700	-0.53273300	1.89084400
C	5.77767500	-3.98711600	2.02208800
H	5.33285200	-4.56964500	2.83595500
H	6.22517300	-4.71650800	1.33244100
C	7.32513800	-2.10762900	1.45786000
H	7.67260300	-2.67999800	0.58680200
H	8.16567100	-1.49363500	1.79694900
C	6.87762800	-3.07122300	2.55304500
H	6.50044700	-2.49400000	3.40712700
H	7.71736700	-3.67287400	2.91535200

Zero-point correction= 0.894666 (Hartree/Particle)

Thermal correction to Energy= 0.939121

Thermal correction to Enthalpy= 0.939923

Thermal correction to Gibbs Free Energy= 0.818548

Sum of electronic and zero-point Energies= -13444.699335

Sum of electronic and thermal Energies= -13444.654880

Sum of electronic and thermal Enthalpies= -13444.654078

Sum of electronic and thermal Free Energies= -13444.775453

AP-8-PTS*R*

C	3.49404000	-2.68355300	-0.02469800
C	1.28439300	-1.68585300	-1.40914700
C	3.57230100	-2.39932000	-1.39824800
C	2.26666200	-2.50127300	0.63100400
C	1.15503900	-1.99721400	-0.05663500
C	2.48113800	-1.86254000	-2.08376900
H	0.17781500	-1.94510800	0.41286300
H	2.54647400	-1.61307300	-3.13529400
H	0.41601600	-1.32567600	-1.94305600
C	4.69181600	-3.20166600	0.69542800
C	6.89058400	-4.34930300	2.01849500
C	5.88461100	-2.46066200	0.84577600
C	4.62531200	-4.48992700	1.23508000
C	5.70652500	-5.06587800	1.89119600
C	6.96794200	-3.05828900	1.50423100
H	3.69421200	-5.03887300	1.12986700
H	5.62490300	-6.07041800	2.29575300
H	7.89767100	-2.51180700	1.62891400

H	7.74987600	-4.78537800	2.51863500
O	2.22643000	-2.83440100	1.94859600
O	4.74432900	-2.71214000	-1.99757400
C	1.10036100	-2.39204700	2.68712200
H	0.18562500	-2.91819900	2.39088000
H	0.95052400	-1.31361700	2.55623800
H	1.31863000	-2.61345700	3.73322800
C	5.00890500	-2.09277200	-3.24059300
H	4.37336300	-2.50528100	-4.03465800
H	6.05166000	-2.31602200	-3.47359200
H	4.86912800	-1.00969000	-3.16632700
P	6.02533400	-0.68327900	0.37340300
O	5.24519300	-0.24647900	-0.83697400
H	-1.78020300	1.25404900	1.18651400
C	-0.18467600	3.32192400	1.35131100
N	0.99280700	2.62952100	1.19355900
C	2.15284400	3.40781400	1.35127100
Br	1.04594300	0.84633700	0.67145500
O	3.27205800	3.00710200	1.17988100
O	-1.29895700	2.85476500	1.20737500
C	1.69362500	4.78684300	1.78919300
H	2.12254200	5.53578000	1.11989400
H	2.08767800	4.96890600	2.79270700
C	0.16221200	4.74311200	1.74697200
H	-0.26571500	5.43037800	1.01143100
H	-0.31130900	4.94848500	2.71030800
C	1.06154500	5.03881900	-1.58636800
C	0.56283400	2.34228900	-2.12922300

C	-0.25114400	4.60033300	-1.68163100
C	2.13157800	4.16038400	-1.71631400
C	1.87782400	2.81601400	-1.96643100
C	-0.48402300	3.25669300	-1.94498100
H	-1.08433200	5.28208900	-1.55176400
H	3.15212500	4.50340600	-1.58797100
O	0.37353800	1.05858700	-2.43964800
H	-0.47857100	0.71455200	-2.08443700
Br	3.28950600	1.57373300	-1.89830900
Br	-2.26396200	2.63393400	-1.98128300
Br	1.38528900	6.87019300	-1.20981800
C	5.52484300	0.22157000	1.89148900
C	3.59622000	0.61693300	3.47908000
C	5.96911700	0.87488500	4.28776100
C	4.54435400	0.46659600	4.67071500
C	6.48134400	0.07739300	3.08227700
C	4.09912000	-0.18926300	2.28190600
H	3.53004300	1.67472600	3.19587300
H	5.98136400	1.94464300	4.03640900
H	4.54723700	-0.58226400	4.99929100
H	6.55605700	-0.98398200	3.35919600
H	4.08294200	-1.25630600	2.54214300
H	5.49372500	1.27375400	1.56936800
H	2.58416600	0.28875600	3.74809100
H	6.64772300	0.73895700	5.13767100
H	4.19647700	1.06587100	5.52019800
H	7.49110600	0.41535400	2.81840300
H	3.43885300	-0.05315100	1.42221200

C	7.81394000	-0.35666400	0.10872200
C	9.75919900	-0.81746900	-1.44591100
C	9.47627300	1.47357300	-0.43340200
C	9.95730900	0.68514600	-1.65257600
C	8.01589000	1.15424000	-0.10130400
C	8.29948600	-1.14154300	-1.12013800
H	10.39905000	-1.15732000	-0.61910100
H	10.10615900	1.22091100	0.43147200
H	9.38534800	1.00420300	-2.53470000
H	7.36569000	1.48436800	-0.92203900
H	7.66158200	-0.86557500	-1.97071700
H	8.39492400	-0.65927400	0.98939200
H	10.07341500	-1.37065800	-2.33812300
H	9.59018200	2.54975000	-0.60521100
H	11.01069400	0.90687800	-1.85759900
H	7.70686800	1.70922300	0.79287700
H	8.16947900	-2.21871300	-0.96136900
C	-5.49323800	1.09126300	0.96996400
H	-6.98816100	1.72582300	2.35906300
C	-6.56721100	0.87173200	1.83259100
C	-5.52396500	-1.30432100	0.46568200
C	-7.08447100	-0.39965400	2.08155500
C	-4.99458600	-0.02387300	0.29963400
C	-6.53912800	-1.51129800	1.41947700
C	-3.59430400	-2.71598000	-0.22188700
C	-4.95390300	-2.42227100	-0.34125400
C	-3.84522100	-4.59027400	-1.65397600
C	-5.74620500	-3.15634600	-1.24686400

C	-3.01077100	-3.82424800	-0.83957400
C	-5.17946200	-4.26109800	-1.89740800
H	-3.42224400	-5.46634800	-2.14133300
O	-3.91411400	0.12625300	-0.56828500
O	-2.81118500	-1.87514500	0.57137800
P	-2.48758100	-0.41532900	-0.03701800
O	-1.49728000	-0.40962100	-1.12955900
O	-2.17284800	0.34168100	1.29836000
H	-0.53199900	-3.51994000	-5.07975200
C	-0.24233500	-3.92552900	-4.11502300
H	-2.04412200	-3.30904500	-3.17041700
C	-1.09050100	-3.81242500	-3.05177500
C	1.42374500	-5.02614500	-2.75910900
C	-0.73210600	-4.32429900	-1.76084200
C	1.02798900	-4.55782700	-3.97556600
C	0.56952300	-4.91672200	-1.61592000
C	-1.59299600	-4.24675800	-0.64516500
H	1.68478400	-4.63838700	-4.83647000
H	1.97067600	-5.81367700	-0.26139400
H	2.40411200	-5.47638700	-2.62406400
C	-1.15717200	-4.69837300	0.61998100
C	-1.98965500	-4.66316200	1.78740500
C	0.16162600	-5.25438100	0.75787800
H	1.60503200	-6.11554500	2.12609600
C	0.98402900	-5.36634900	-0.36400600
C	-1.53721900	-5.11624000	2.99297300
H	-2.99934200	-4.27466300	1.70511100
H	-2.18776900	-5.07769100	3.86161500

C	-0.21954200	-5.64394600	3.12895600
H	0.12235500	-5.99283500	4.09852800
C	0.59975000	-5.71068800	2.04189000
H	-6.19190500	2.82204500	-3.60007700
C	-5.76292500	3.29170300	-2.72023400
H	-6.02627100	1.56548300	-1.50848100
C	-5.66754400	2.58872300	-1.55389500
C	-4.77224100	5.24404400	-1.70181800
C	-5.08544100	3.17491400	-0.38293300
C	-5.31030900	4.64140100	-2.79892400
C	-4.63202000	4.53548800	-0.46674400
C	-4.92373700	2.46127200	0.82114500
H	-5.39816700	5.18177700	-3.73628400
H	-3.71684800	6.17198500	0.58176300
H	-4.42468000	6.27337000	-1.74547600
C	-4.27980800	3.06228800	1.92162800
C	-3.99443600	2.34821100	3.13228300
C	-3.84980000	4.42969500	1.83129000
H	-2.91243400	6.07991100	2.87819600
C	-4.04438300	5.13579500	0.64624500
C	-3.35770700	2.95484300	4.17488700
H	-4.27064900	1.30153900	3.20021700
H	-3.14053900	2.39103900	5.07704200
C	-2.96937300	4.32524400	4.09560100
H	-2.48159900	4.79442600	4.94527000
C	-3.20802600	5.03606100	2.95732500
C	-5.96504300	-5.10512200	-2.88250600
H	-5.59787900	-4.88901200	-3.89508600

H	-5.74883600	-6.16354600	-2.69872100
C	-7.16804100	-2.71497100	-1.54884300
H	-7.19546700	-1.62126900	-1.60818300
H	-7.83512300	-2.98674100	-0.71978400
C	-7.71621400	-3.33488100	-2.83356000
H	-8.78406700	-3.11028400	-2.91888300
H	-7.22051800	-2.88709500	-3.70471000
C	-7.46708100	-4.84017000	-2.83157800
H	-7.95918600	-5.32432800	-3.68089400
H	-7.89334300	-5.27628900	-1.91817900
C	-6.94252000	-2.91582900	1.83186300
H	-6.89172600	-3.60781600	0.98976100
H	-6.18630900	-3.27050700	2.54802900
C	-8.24051500	-0.53601800	3.05467300
H	-8.12405400	0.19768500	3.85950000
H	-9.16617500	-0.26713100	2.52680200
C	-8.31404500	-2.97278000	2.50013500
H	-9.09767600	-2.76199700	1.75938300
H	-8.49372200	-3.98301000	2.88159900
C	-8.38900300	-1.94439000	3.62517500
H	-7.58352300	-2.14050600	4.34473800
H	-9.33508100	-2.02525500	4.16995900

Zero-point correction= 1.469221 (Hartree/Particle)

Thermal correction to Energy= 1.535326

Thermal correction to Enthalpy= 1.536128

Thermal correction to Gibbs Free Energy= 1.371261

Sum of electronic and zero-point Energies= -15022.512358

Sum of electronic and thermal Energies= -15022.446253

Sum of electronic and thermal Enthalpies= -15022.445451

Sum of electronic and thermal Free Energies= -15022.610318

AP-8-INT1*R*

C	3.65841200	-2.37456100	0.16996400
C	1.41421900	-1.48266200	-1.34092900
C	3.68999500	-2.20613900	-1.23552800
C	2.46265900	-2.15245500	0.82893800
C	1.27204800	-1.62601800	0.11773800
C	2.58027300	-1.68754300	-1.97752800
H	0.34005100	-2.13532100	0.38156600
H	2.65765100	-1.52932200	-3.04436400
H	0.52289800	-1.16616800	-1.86481000
C	4.88836700	-2.83132100	0.87747400
C	7.17501800	-3.84452800	2.13924400
C	6.08162700	-2.07923100	0.86208600
C	4.85515100	-4.05340100	1.55281700
C	5.98521700	-4.56146100	2.18298600
C	7.21355300	-2.61329500	1.49049900
H	3.91921400	-4.60273700	1.58269800
H	5.93629000	-5.51496000	2.69977000
H	8.14791200	-2.06040500	1.48760300
H	8.07007800	-4.23288600	2.61487000
O	2.42843000	-2.40183600	2.12309800
O	4.77554100	-2.64186800	-1.82817300
C	1.25146400	-2.17575300	2.91567400
H	0.35927300	-2.58770100	2.43678500
H	1.12436900	-1.10829200	3.09257800

H	1.43983000	-2.70205500	3.84964300
C	5.01944600	-2.28260200	-3.18980000
H	4.28890900	-2.76052200	-3.84887100
H	6.01575300	-2.66136700	-3.41350400
H	4.99331400	-1.19473800	-3.28704700
P	6.15827000	-0.36304700	0.18677600
O	5.21620900	-0.10223400	-0.96458700
H	-1.85652600	2.04999200	1.15359800
C	-0.60319200	3.48696500	1.54786400
N	0.48650500	2.79049900	1.41494900
C	1.57701000	3.63520000	1.64532900
Br	1.01298200	0.25269200	0.74395000
O	2.73346400	3.31576100	1.48386700
O	-1.80370800	3.04930900	1.35248400
C	1.09699800	5.01387100	2.11454000
H	1.55788000	5.79512100	1.50675100
H	1.41757100	5.16105900	3.15003800
C	-0.41964200	4.92859600	1.96479700
H	-0.81924700	5.59550700	1.19385700
H	-0.98160500	5.11260100	2.88446200
C	0.66212900	5.10349200	-1.30827400
C	0.35823300	2.38323900	-1.88320400
C	-0.61468100	4.58802800	-1.47980400
C	1.78807700	4.29396500	-1.38469900
C	1.62787000	2.94006900	-1.65333300
C	-0.74955900	3.23576500	-1.75982800
H	-1.49362300	5.21764500	-1.39694000
H	2.77688100	4.69730600	-1.20029600

O	0.25700300	1.10181300	-2.23820000
H	-0.56689700	0.66260400	-1.88362400
Br	3.12820600	1.80209400	-1.58951200
Br	-2.48210500	2.51193400	-1.92774700
Br	0.85973300	6.95681800	-0.94624300
C	5.84328600	0.71143900	1.63933900
C	4.13701700	1.34900400	3.39668800
C	6.58074600	1.45920300	3.94617800
C	5.18514600	1.15359300	4.49129700
C	6.90548500	0.57111300	2.74127100
C	4.42343000	0.47019800	2.17688200
H	4.12595900	2.39755300	3.08174700
H	6.62582100	2.51363300	3.64135900
H	5.15834000	0.11539400	4.85253800
H	6.93925500	-0.47378200	3.07907600
H	4.31897900	-0.58592800	2.46933900
H	5.87479400	1.73324100	1.23091200
H	3.13057700	1.12733900	3.77163500
H	7.34188000	1.31676500	4.72175300
H	4.96623600	1.79689100	5.35114800
H	7.90156200	0.81856800	2.35317900
H	3.69004100	0.67644500	1.39097600
C	7.89557900	-0.09711700	-0.34067200
C	9.62246600	-0.75432300	-2.07180600
C	9.49699100	1.63010100	-1.26643300
C	9.80791200	0.71551100	-2.45190800
C	8.08856600	1.38213700	-0.71869200
C	8.21161600	-1.00957800	-1.53633000

H	10.35724700	-1.02608600	-1.30098400
H	10.23008700	1.44805000	-0.46793200
H	9.13135700	0.96015100	-3.28228100
H	7.34068200	1.65387500	-1.47555300
H	7.47735300	-0.80043800	-2.32599500
H	8.57962100	-0.33196200	0.48489200
H	9.81509900	-1.40055300	-2.93549900
H	9.59901400	2.68122800	-1.55728100
H	10.82847000	0.89086900	-2.80951600
H	7.91111100	2.02522600	0.15127400
H	8.09062300	-2.06287300	-1.25425100
C	-5.66909500	0.74686300	0.73188100
H	-7.29174600	1.28951300	2.01262800
C	-6.74358100	0.45804600	1.57367300
C	-5.38426000	-1.65651200	0.42006700
C	-7.11070200	-0.84507200	1.90875300
C	-4.99225000	-0.33800000	0.17140600
C	-6.40731000	-1.92235700	1.34877500
C	-3.28166500	-2.85658900	-0.13142400
C	-4.66469700	-2.74177200	-0.30424000
C	-3.27792300	-4.80616400	-1.50886800
C	-5.34015000	-3.59685600	-1.19846500
C	-2.56924100	-3.91875900	-0.69878300
C	-4.63333000	-4.64864000	-1.79683800
H	-2.74078000	-5.64351900	-1.95045400
O	-3.91799300	-0.13396500	-0.66910800
O	-2.63483300	-1.91575700	0.64230500
P	-2.42305700	-0.41443700	-0.03258500

O	-1.48810300	-0.56321500	-1.19237500
O	-2.18423000	0.51317400	1.11692900
H	0.02964000	-3.48341700	-4.85191900
C	0.31131000	-3.85542800	-3.87174700
H	-1.53621800	-3.29031800	-2.97492100
C	-0.56609000	-3.75370600	-2.83053800
C	1.99140600	-4.85568300	-2.44952600
C	-0.22046400	-4.23243500	-1.52236300
C	1.59969400	-4.43982700	-3.68789300
C	1.10703400	-4.74888200	-1.32830800
C	-1.12790100	-4.19955900	-0.44155600
H	2.26949900	-4.53659900	-4.53779400
H	2.50860900	-5.55326500	0.08305200
H	2.98087600	-5.27686000	-2.28552700
C	-0.70292300	-4.59657000	0.84742700
C	-1.57342500	-4.58238100	1.98685800
C	0.64350200	-5.06521800	1.03983700
H	2.08161100	-5.83797100	2.46790500
C	1.50821100	-5.14743300	-0.05369200
C	-1.13460400	-4.98354800	3.21581900
H	-2.59671800	-4.24589000	1.86315400
H	-1.81235600	-4.96045600	4.06356300
C	0.20282000	-5.44111300	3.40181100
H	0.52842400	-5.76094700	4.38705200
C	1.06239600	-5.47970600	2.34465300
H	-6.33639600	1.96250700	-4.00627300
C	-6.04023700	2.56478900	-3.15280400
H	-6.10639200	0.92424000	-1.80287300

C	-5.91066200	1.98452200	-1.92416000
C	-5.41958000	4.72493400	-2.26917600
C	-5.49731600	2.74876100	-0.78474400
C	-5.79210100	3.95731700	-3.33166000
C	-5.25030200	4.15173800	-0.96964600
C	-5.29421200	2.16758900	0.48308000
H	-5.90086800	4.39863800	-4.31764400
H	-4.64574500	5.98896600	-0.03447100
H	-5.22519400	5.78776500	-2.39046600
C	-4.81228900	2.94925700	1.55148100
C	-4.50126000	2.38818700	2.83399800
C	-4.58463400	4.35432700	1.35837700
H	-3.95363000	6.20567500	2.29354000
C	-4.81962600	4.92403500	0.10882200
C	-4.03768300	3.16985000	3.85034100
H	-4.61300200	1.31910600	2.97652700
H	-3.79796700	2.72240600	4.81030400
C	-3.84855900	4.57146000	3.66487500
H	-3.48898400	5.17815000	4.49141900
C	-4.10736700	5.14104800	2.45428100
C	-5.28706400	-5.61244100	-2.76854500
H	-4.92712100	-5.38618200	-3.78171500
H	-4.95052900	-6.63139400	-2.54532900
C	-6.79598600	-3.33894500	-1.54818500
H	-6.95059200	-2.25917200	-1.65007800
H	-7.44689400	-3.65695500	-0.72262900
C	-7.23658700	-4.06530500	-2.81844800
H	-8.32129300	-3.97288300	-2.93346100

H -6.77680900 -3.59294300 -3.69642200

C -6.81078400 -5.52909800 -2.75550000

H -7.22263800 -6.09876800 -3.59470200

H -7.20230800 -5.98032500 -1.83385700

C -6.66045400 -3.33625100 1.84149800

H -6.49396300 -4.06829600 1.04910700

H -5.90009800 -3.55458700 2.60632400

C -8.28145200 -1.05497700 2.85120800

H -8.28414000 -0.26319100 3.60827600

H -9.21234100 -0.93426600 2.27934100

C -8.04346500 -3.51371100 2.46340000

H -8.81359900 -3.44349300 1.68267500

H -8.12285200 -4.51208100 2.90612400

C -8.28566300 -2.43145000 3.51220200

H -7.49184600 -2.48225500 4.26891000

H -9.23668700 -2.59141700 4.03075900

Zero-point correction= 1.469504 (Hartree/Particle)

Thermal correction to Energy= 1.535356

Thermal correction to Enthalpy= 1.536158

Thermal correction to Gibbs Free Energy= 1.372591

Sum of electronic and zero-point Energies= -15022.497809

Sum of electronic and thermal Energies= -15022.431956

Sum of electronic and thermal Enthalpies= -15022.431155

Sum of electronic and thermal Free Energies= -15022.594722

AP-8-INT2^{R}

C -2.24376000 -0.36672900 -3.13753400

H -2.57470600 0.07423000 -5.20387100

C	-3.01928800	-0.32168900	-4.29258800
C	-4.12289500	-1.44333400	-2.00132200
C	-4.35278500	-0.73965900	-4.31051200
C	-2.83385300	-0.89487500	-1.98640500
C	-4.91879600	-1.29514100	-3.15286800
C	-4.48405500	-1.55333000	0.44834700
C	-4.54647300	-2.20397700	-0.78773500
C	-5.03580700	-3.58107600	1.57462500
C	-4.81648600	-3.58936100	-0.83472200
C	-4.69244700	-2.23464600	1.65090000
C	-5.10573300	-4.26722100	0.35997900
H	-5.21430600	-4.12617800	2.49998600
O	-2.12119000	-0.90077100	-0.80491400
O	-4.19341300	-0.21146100	0.46207700
P	-2.61182000	0.23320300	0.31758600
O	-1.86819900	-0.07607200	1.56861000
O	-2.63456800	1.59355200	-0.30526500
C	2.60750800	-2.64530600	1.77122400
C	0.46280400	-2.53737200	-0.10711600
C	1.65495000	-1.64504100	1.90597900
C	2.48250700	-3.53789100	0.69628600
C	1.36609800	-3.52552800	-0.21074900
C	0.55578300	-1.48861400	0.92262700
H	1.28863500	-4.26365800	-0.99777900
H	-0.42325200	-1.25922800	1.37428600
H	-0.37261300	-2.44636200	-0.79389200
C	3.84988800	-2.61664800	2.59277500
C	6.11562800	-2.45065100	4.22227600

C	5.06979300	-2.18221200	2.04066300
C	3.78475300	-2.96804700	3.94215900
C	4.90980800	-2.89419400	4.75534000
C	6.18846900	-2.09446300	2.87897800
H	2.83425700	-3.29763300	4.35217300
H	4.84155800	-3.17129400	5.80264400
H	7.13347400	-1.72467000	2.49197500
H	6.99781800	-2.37325000	4.84997000
O	3.45650800	-4.41589000	0.57704700
O	1.79451200	-0.81488800	2.90578700
C	3.50632900	-5.27382800	-0.56640600
H	2.68506000	-5.99514300	-0.54083300
H	3.47123800	-4.68167800	-1.48452500
H	4.45545000	-5.80298200	-0.48972400
C	0.92213900	0.31989100	3.11934300
H	-0.10078700	0.12963000	2.78670500
H	0.94992700	0.48518500	4.19573900
H	1.33980200	1.17994600	2.59110900
P	5.23337400	-1.70401200	0.26745000
O	3.93105500	-1.65087500	-0.48864700
H	-0.25320800	-3.45312800	3.31399600
C	-1.02594400	-2.82469500	3.75208200
H	-2.36568700	-3.09475500	2.12078900
C	-2.20499900	-2.63656600	3.09190600
C	-1.74936500	-1.40885100	5.57239000
C	-3.24286900	-1.81397700	3.64273400
C	-0.79014300	-2.19999200	5.01467400
C	-3.00058900	-1.18623900	4.91088900

C	-4.45600200	-1.57485100	2.96805100
H	0.15633400	-2.35626500	5.52453200
H	-3.79247100	0.11071600	6.43058400
H	-1.58304200	-0.92353100	6.53091100
C	-5.42623800	-0.72664100	3.53052100
C	-6.66689300	-0.44601000	2.87182300
C	-5.18195100	-0.11633100	4.80754200
H	-5.98487700	1.19211400	6.33886800
C	-3.97767500	-0.36131300	5.46785400
C	-7.59459300	0.37471600	3.44220500
H	-6.85566000	-0.89802100	1.90317900
H	-8.52773900	0.57958400	2.92631200
C	-7.35185400	0.97551500	4.71349700
H	-8.10340200	1.62697300	5.14906100
C	-6.18335300	0.73629100	5.37218700
H	0.51710600	-4.32890100	-3.87042500
C	0.80001600	-3.29573100	-3.68680300
H	-1.21598900	-2.62457800	-3.55645700
C	-0.16728500	-2.34650900	-3.51318000
C	2.54681200	-1.64480900	-3.42947200
C	0.17294600	-0.96902500	-3.30389800
C	2.18088400	-2.94300600	-3.63623800
C	1.56434800	-0.61618000	-3.28626900
C	-0.80544800	0.03512500	-3.13852700
H	2.93844700	-3.71039300	-3.77605500
H	2.98649900	0.98708900	-3.11058000
H	3.59464100	-1.36430900	-3.36928500
C	-0.42571400	1.38297900	-3.01651000

C	-1.38626900	2.44312800	-2.91385300
C	0.97135500	1.72776700	-3.01397300
H	2.40735200	3.34969100	-2.88520200
C	1.93102300	0.72239700	-3.13376400
C	-0.98585000	3.74552500	-2.87481400
H	-2.43897700	2.18874200	-2.85965900
H	-1.72710000	4.53520500	-2.79957500
C	0.40154900	4.08483900	-2.86816300
H	0.69413200	5.12980900	-2.80591400
C	1.34800600	3.10621300	-2.91030800
Br	0.98166600	0.16232700	-0.06566800
C	0.55194800	6.87923400	0.21800400
C	-0.21219500	4.16630200	0.20760500
C	-0.78722700	6.54268800	0.08718700
C	1.52637000	5.89535500	0.34193200
C	1.13105100	4.56930100	0.33362400
C	-1.15485100	5.20232000	0.08630900
H	-1.54608300	7.30986200	-0.01309100
H	2.57403500	6.15629200	0.43521200
O	-0.46403200	2.87341200	0.20269700
H	-1.40608400	2.56038700	0.00047400
Br	2.43169100	3.20300400	0.44431800
Br	-2.97284200	4.75094100	-0.11530000
Br	1.06300800	8.70517900	0.22249200
C	-5.42149000	-5.75184100	0.39555500
H	-4.58218100	-6.27674800	0.87257100
H	-6.29005600	-5.91766700	1.04327600
C	-4.71289300	-4.35293600	-2.14465300

H	-3.86528500	-3.96354800	-2.71930600
H	-5.60095200	-4.17035400	-2.76402600
C	-4.57670000	-5.86130500	-1.94315300
H	-4.64462200	-6.36692700	-2.91189500
H	-3.58930300	-6.09589300	-1.52308100
C	-5.65925300	-6.35193500	-0.98715500
H	-5.66950100	-7.44482200	-0.92298700
H	-6.64134600	-6.04078200	-1.36705300
C	-6.40126400	-1.61432200	-3.10518600
H	-6.60218800	-2.44565100	-2.42648200
H	-6.90481900	-0.74472200	-2.65858100
C	-5.13293500	-0.62930000	-5.60807900
H	-4.85978000	0.30130900	-6.11755600
H	-4.81457700	-1.44528200	-6.27245000
C	-6.99932400	-1.86769500	-4.48634200
H	-6.60192100	-2.80553200	-4.89935300
H	-8.08496100	-1.98451700	-4.40427400
C	-6.64638500	-0.71064100	-5.41819300
H	-7.01513000	0.22503900	-4.97854500
H	-7.13587600	-0.82450700	-6.39106600
C	6.36291300	-2.97089500	-0.46393600
C	8.66254800	-3.95825600	-0.79005900
C	6.90829400	-4.23789300	-2.57953100
C	8.40273800	-4.08122100	-2.29213300
C	6.11071200	-3.07922100	-1.97846600
C	7.86089700	-2.80863300	-0.16799100
H	8.37752100	-4.89720900	-0.29546700
H	6.55462300	-5.18566700	-2.14776900

H	8.77092800	-3.17757900	-2.79759400
H	6.41600900	-2.14497600	-2.46864000
H	8.21661000	-1.85497900	-0.58171300
H	6.02737500	-3.91202700	0.00088600
H	9.73061000	-3.80974800	-0.59736100
H	6.72984000	-4.29381300	-3.65907900
H	8.96310300	-4.92692000	-2.70513000
H	5.03764600	-3.18677400	-2.16616100
H	8.04487700	-2.78288100	0.91064600
C	6.04612100	-0.06257400	0.32286500
C	5.71132300	2.32659700	1.08473300
C	6.89748300	1.86727300	-1.08558600
C	5.97624900	2.82793000	-0.33518300
C	6.32837000	0.44566700	-1.10094000
C	5.12024100	0.91433400	1.07271400
H	6.65440200	2.31507000	1.64912600
H	7.88270600	1.85467100	-0.59849700
H	5.01850200	2.90769800	-0.86751900
H	5.38642700	0.42976600	-1.66550700
H	4.13857900	0.91077300	0.57322700
H	6.99788200	-0.14923200	0.86697100
H	5.03519700	3.00763200	1.61151500
H	7.05586400	2.21026800	-2.11394100
H	6.41127900	3.83313100	-0.31026400
H	7.02491300	-0.22481800	-1.61687200
H	4.95152400	0.56704600	2.09872800

Zero-point correction= 1.373912 (Hartree/Particle)

Thermal correction to Energy= 1.434890

Thermal correction to Enthalpy= 1.435692

Thermal correction to Gibbs Free Energy= 1.279970

Sum of electronic and zero-point Energies= -14662.037168

Sum of electronic and thermal Energies= -14661.976190

Sum of electronic and thermal Enthalpies= -14661.975388

Sum of electronic and thermal Free Energies= -14662.131110

AP-8-TS1*R*

C	3.44582900	-2.50362000	0.09141000
C	1.26151900	-1.59856800	-1.45196100
C	3.53919000	-2.30670300	-1.30247700
C	2.22044800	-2.26931800	0.70989200
C	1.09722300	-1.74430100	-0.03222900
C	2.45808100	-1.80296000	-2.06591600
H	0.09420300	-1.98890800	0.31317200
H	2.55704900	-1.63640900	-3.12992100
H	0.39190600	-1.28629400	-2.01374600
C	4.62900200	-3.00777500	0.84680100
C	6.81158500	-4.13085400	2.20749300
C	5.84092600	-2.29103100	0.93530500
C	4.52940900	-4.25499800	1.46904200
C	5.60475900	-4.81744600	2.14695200
C	6.91826700	-2.87736600	1.61149100
H	3.58152600	-4.78175600	1.41599300
H	5.50043900	-5.78960700	2.61920400
H	7.86422500	-2.34948000	1.68576600
H	7.66600600	-4.56078300	2.72086100
O	2.14386100	-2.51888000	2.01623900

O	4.68346100	-2.68705000	-1.85894400
C	0.93482400	-2.22562300	2.72238400
H	0.09345200	-2.80107900	2.32722300
H	0.71435100	-1.15811700	2.66709500
H	1.12536700	-2.52212600	3.75328400
C	4.98486900	-2.21323300	-3.16650100
H	4.33409800	-2.68091300	-3.91267200
H	6.01648800	-2.50955700	-3.35574800
H	4.89412300	-1.12384000	-3.19551800
P	6.00486200	-0.54995500	0.35275900
O	5.14059200	-0.18885400	-0.82643600
H	-1.58261500	1.72544300	1.10757700
C	-0.14765400	3.18682400	1.39052400
N	0.94451400	2.48689700	1.15440300
C	2.09389900	3.27118600	1.33952800
Br	1.07894300	0.39564400	0.52388600
O	3.21728600	2.88935300	1.12569200
O	-1.34713700	2.79272700	1.28465500
C	1.67592500	4.64514300	1.85829000
H	2.09514500	5.42363600	1.21716900
H	2.09290400	4.77520400	2.86095800
C	0.14895200	4.59906300	1.84512400
H	-0.30525300	5.31161100	1.15012500
H	-0.31560300	4.75028900	2.82313600
C	0.90687700	5.01425500	-1.48246200
C	0.43474800	2.32985700	-2.12387800
C	-0.40085000	4.56237000	-1.58054400
C	1.98474500	4.15757500	-1.66696700

C	1.74305200	2.82140600	-1.96681900
C	-0.61915200	3.22821300	-1.89708100
H	-1.24133800	5.22662500	-1.41273400
H	3.00299200	4.50798500	-1.54183600
O	0.24959000	1.06037300	-2.49013500
H	-0.57386800	0.66797400	-2.09486500
Br	3.18672900	1.61761100	-1.98009700
Br	-2.39318000	2.59643000	-1.96528200
Br	1.21531100	6.83368400	-1.03999300
C	5.65329700	0.45311000	1.85209200
C	3.92561700	0.95830100	3.63017900
C	6.36801600	1.20940200	4.15921500
C	4.99414300	0.83310700	4.71643000
C	6.73141100	0.35209200	2.94196100
C	4.26793400	0.09828400	2.41184900
H	3.85167800	2.00597300	3.31401700
H	6.35873300	2.26698800	3.86149300
H	5.02479400	-0.20308300	5.08202400
H	6.82301600	-0.69541400	3.26290800
H	4.25344200	-0.95967900	2.71051600
H	5.60420700	1.48548300	1.47359600
H	2.94127400	0.66715900	4.01728100
H	7.13986700	1.10098700	4.92975400
H	4.74800300	1.46778100	5.57549500
H	7.71045200	0.65927600	2.55528100
H	3.51286800	0.23656400	1.63387500
C	7.77671700	-0.30928400	-0.06378200
C	9.59405100	-0.94388700	-1.70751000

C	9.45943700	1.41680500	-0.83262400
C	9.83143200	0.53361700	-2.02470900
C	8.01424400	1.17632400	-0.38745700
C	8.14826500	-1.18790000	-1.26826200
H	10.27436300	-1.25649100	-0.90278100
H	10.13554200	1.19636500	0.00567100
H	9.21511400	0.81866600	-2.88843500
H	7.32296400	1.47577600	-1.18616800
H	7.46696200	-0.93707300	-2.09247600
H	8.40848100	-0.58110100	0.79179300
H	9.82827900	-1.56461200	-2.57966800
H	9.59832900	2.47433000	-1.08282100
H	10.87635000	0.69950200	-2.30949700
H	7.78283100	1.80144700	0.48330900
H	7.98991200	-2.24722000	-1.03185900
C	-5.50238600	1.02468100	0.95090500
H	-6.99235600	1.62811700	2.35967100
C	-6.55078100	0.78118000	1.83811400
C	-5.45574100	-1.37291300	0.48481500
C	-7.01910600	-0.50310000	2.11566200
C	-4.96831200	-0.07904000	0.28549200
C	-6.44676400	-1.60301600	1.45750300
C	-3.48094500	-2.70960600	-0.20496900
C	-4.85466300	-2.47787800	-0.31543700
C	-3.68129800	-4.61560400	-1.62032800
C	-5.62772400	-3.25054700	-1.20536900
C	-2.87377300	-3.81019100	-0.81783600
C	-5.02858300	-4.33793200	-1.85575000

H	-3.23090500	-5.48277700	-2.09961700
O	-3.92711800	0.08645700	-0.60935300
O	-2.72661300	-1.83871500	0.56188100
P	-2.43824400	-0.35262100	-0.08486600
O	-1.55872700	-0.48418500	-1.28017600
O	-2.06833400	0.49113000	1.11393300
H	-0.34093300	-3.49264500	-5.02895000
C	-0.05661200	-3.88082500	-4.05570800
H	-1.87526200	-3.27265900	-3.13263400
C	-0.91648900	-3.76287900	-3.00229000
C	1.61090400	-4.94325900	-2.66730000
C	-0.56588600	-4.25829300	-1.70205700
C	1.21763500	-4.50210700	-3.89505600
C	0.74626200	-4.82098900	-1.53245900
C	-1.44932900	-4.19541600	-0.60410500
H	1.87726700	-4.60041700	-4.75231200
H	2.14838000	-5.66885200	-0.14740300
H	2.59196700	-5.38853600	-2.51880000
C	-1.02383500	-4.63118100	0.67132100
C	-1.88104800	-4.61373800	1.82121200
C	0.30851500	-5.14557200	0.83888700
H	1.74241000	-5.97238200	2.24021600
C	1.15511600	-5.24090800	-0.26767600
C	-1.44073800	-5.04757800	3.03839400
H	-2.89790600	-4.25175300	1.71483300
H	-2.10940300	-5.02292800	3.89344400
C	-0.11231100	-5.53827500	3.20287100
H	0.21794700	-5.87780400	4.17981800

C	0.73186100	-5.58609800	2.13373300
H	-6.39432200	2.65725500	-3.61797700
C	-5.96150500	3.15707700	-2.75678300
H	-6.12446900	1.44202100	-1.51051800
C	-5.80975100	2.47808700	-1.58240900
C	-5.01557700	5.16093300	-1.79585400
C	-5.21967000	3.10497000	-0.43692000
C	-5.55962200	4.52063500	-2.86859600
C	-4.81808500	4.47920000	-0.55362000
C	-4.99243700	2.41350800	0.76902700
H	-5.68968700	5.04160700	-3.81215200
H	-3.92901500	6.16213300	0.44342900
H	-4.70442600	6.20040600	-1.86610000
C	-4.33814800	3.05302200	1.84052400
C	-3.99656600	2.36626000	3.05205500
C	-3.95934400	4.43323200	1.71908600
H	-3.04709200	6.12810100	2.71707100
C	-4.21761900	5.11620700	0.53200700
C	-3.36323800	3.01191200	4.07262700
H	-4.22625800	1.30980900	3.13521000
H	-3.10499100	2.47015000	4.97759000
C	-3.02542000	4.39353900	3.96360000
H	-2.53511100	4.89300300	4.79463600
C	-3.30972600	5.07753300	2.81943200
C	-5.79267000	-5.21685800	-2.82747800
H	-5.44881000	-4.99190200	-3.84643800
H	-5.53418400	-6.26575200	-2.64254200
C	-7.06881700	-2.86546600	-1.49318800

H	-7.13757200	-1.77409300	-1.56025100
H	-7.71505300	-3.15461400	-0.65345600
C	-7.60978900	-3.51516100	-2.76609500
H	-8.68657900	-3.33224800	-2.83940600
H	-7.14261900	-3.05517000	-3.64667300
C	-7.30323000	-5.00974300	-2.75791200
H	-7.78809800	-5.51865100	-3.59707000
H	-7.69977700	-5.45514800	-1.83558200
C	-6.79473200	-3.01576800	1.89256500
H	-6.73827100	-3.71446400	1.05612300
H	-6.01370600	-3.33905600	2.59709400
C	-8.15165900	-0.66654200	3.11223300
H	-8.04610900	0.08044200	3.90648000
H	-9.09734900	-0.43780300	2.60097000
C	-8.15035900	-3.11117500	2.58843300
H	-8.95463100	-2.93538300	1.86068100
H	-8.28941800	-4.12226000	2.98492300
C	-8.23910900	-2.07194300	3.70262900
H	-7.41258600	-2.23076400	4.40756300
H	-9.17055800	-2.17953800	4.26799400

Zero-point correction= 1.466857 (Hartree/Particle)

Thermal correction to Energy= 1.532051

Thermal correction to Enthalpy= 1.532853

Thermal correction to Gibbs Free Energy= 1.371095

Sum of electronic and zero-point Energies= -15022.496104

Sum of electronic and thermal Energies= -15022.430910

Sum of electronic and thermal Enthalpies= -15022.430109

Sum of electronic and thermal Free Energies= -15022.591867

AP-8-TS1^{R2}

C	3.56101900	-2.26797700	0.01143500
C	1.29276700	-1.42836400	-1.44395300
C	3.60208000	-2.04092500	-1.38064100
C	2.34939100	-2.07978800	0.67355100
C	1.18247200	-1.58374000	-0.01947900
C	2.47557600	-1.57088100	-2.09874600
H	0.20431300	-1.88241900	0.35411600
H	2.52896700	-1.39600700	-3.16473200
H	0.39057800	-1.16817300	-1.97742200
C	4.77353500	-2.78557700	0.70984100
C	7.00384900	-3.96854600	1.94342300
C	6.00106400	-2.09229600	0.75145000
C	4.68609400	-4.04193600	1.31817400
C	5.78335700	-4.63433100	1.93104700
C	7.09817900	-2.70633200	1.36690800
H	3.72748400	-4.55028000	1.30585200
H	5.68493300	-5.61251600	2.39206600
H	8.04697000	-2.18266100	1.41612400
H	7.87463600	-4.41920300	2.40930300
O	2.32416900	-2.35139900	1.97826700
O	4.73637500	-2.37025400	-1.98556100
C	1.11706500	-2.14218600	2.71717700
H	0.30530500	-2.76896700	2.33720500
H	0.82604100	-1.09058300	2.67812600
H	1.35255500	-2.43329200	3.74030700
C	4.97605800	-1.85005400	-3.28883600

H	4.32604700	-2.33023000	-4.02754600
H	6.01418400	-2.09183100	-3.51552300
H	4.83503200	-0.76597400	-3.28737400
P	6.15774600	-0.32816000	0.25494600
O	5.28454900	0.08268000	-0.90310900
H	-1.71164700	1.62704400	1.15664200
C	-0.41188200	3.25424800	1.37136100
N	0.75166400	2.67082800	1.14191500
C	1.79863100	3.60280100	1.24713100
Br	1.04771700	0.56223600	0.55802700
O	2.94764400	3.37723000	0.96356300
O	-1.56063200	2.73120800	1.30608700
C	1.23893400	4.92064600	1.77415500
H	1.58217200	5.74408200	1.14544200
H	1.63475700	5.07693900	2.78211500
C	-0.27226300	4.71135800	1.75713700
H	-0.78903600	5.33127200	1.01782900
H	-0.76531600	4.86291300	2.72064500
C	0.65668800	5.11620500	-1.60772900
C	0.30440700	2.39229600	-2.13978600
C	-0.63013900	4.60115400	-1.67245500
C	1.77138000	4.30288000	-1.76604600
C	1.59000700	2.94567800	-2.00775100
C	-0.78955800	3.24742500	-1.93324900
H	-1.49877100	5.23384800	-1.52684600
H	2.77222700	4.70369200	-1.65600200
O	0.17355400	1.10598500	-2.46637600
H	-0.63708000	0.69365200	-2.06702500

Br	3.09060900	1.80683700	-1.98788200
Br	-2.53239100	2.52881400	-1.95761200
Br	0.88425400	6.96637100	-1.25296200
C	5.74963200	0.57596300	1.80554100
C	3.77917900	1.48103100	3.11416400
C	5.87916500	0.79655700	4.31677700
C	4.35392200	0.88977200	4.40164800
C	6.30786800	-0.03299400	3.10177300
C	4.22023200	0.68693300	1.88524800
H	4.11183400	2.52128400	3.00790400
H	6.30183300	1.80799700	4.23732100
H	3.94336200	-0.11878800	4.55484200
H	5.90653100	-1.04818900	3.21642900
H	3.80343500	-0.32752700	1.96235500
H	6.16654800	1.58684000	1.66797900
H	2.68174700	1.49959200	3.14311700
H	6.28868600	0.35017100	5.23017300
H	4.05748200	1.49069500	5.26922900
H	7.39943400	-0.12612500	3.07019900
H	3.83028900	1.13685800	0.97025300
C	7.90739500	0.04067200	-0.20216000
C	10.25201700	0.83907200	0.22892500
C	9.73399400	-0.45527300	-1.87402300
C	10.78265300	-0.16215300	-0.79913300
C	8.42879100	-0.95624000	-1.24994000
C	8.95592100	0.34030000	0.87945100
H	10.05564800	1.79858300	-0.26880100
H	9.53151500	0.46315100	-2.44163100

H	11.04605000	-1.09866800	-0.28713900
H	8.60414500	-1.93325100	-0.78140600
H	9.18564300	-0.55603400	1.46939200
H	7.72817900	0.99107600	-0.73112000
H	11.00454500	1.02921200	1.00215000
H	10.11448100	-1.19453300	-2.58768500
H	11.70201400	0.21774100	-1.25802800
H	7.66534800	-1.10395100	-2.02252000
H	8.57741600	1.09476400	1.57796100
C	-5.57816900	0.77127100	1.01765900
H	-7.10788000	1.26904500	2.42540900
C	-6.62283200	0.45710800	1.88709000
C	-5.41621200	-1.60964600	0.49110400
C	-7.03420000	-0.85396000	2.12634900
C	-4.98920500	-0.28911500	0.32872700
C	-6.40368800	-1.90937500	1.44835300
C	-3.37323200	-2.83130700	-0.21090300
C	-4.75572300	-2.66648900	-0.32776200
C	-3.46032600	-4.70862100	-1.67216000
C	-5.48039300	-3.45823600	-1.24120500
C	-2.70207400	-3.88011800	-0.84538100
C	-4.81939600	-4.49773300	-1.91023700
H	-2.96139700	-5.53806300	-2.16967300
O	-3.95261000	-0.04865400	-0.55499700
O	-2.67009000	-1.93992600	0.58074400
P	-2.44666700	-0.43750200	-0.04514100
O	-1.56877300	-0.50678400	-1.24571000
O	-2.10133800	0.39403600	1.17176200

H	-0.20080200	-3.34438000	-5.04975800
C	0.10509800	-3.73776800	-4.08517500
H	-1.73350800	-3.22642800	-3.14300300
C	-0.75396400	-3.67751100	-3.02630000
C	1.82614100	-4.74770000	-2.72297600
C	-0.37545600	-4.18167200	-1.73714300
C	1.40665300	-4.30443600	-3.94127300
C	0.96210000	-4.68525800	-1.58281800
C	-1.25748900	-4.18482400	-0.63621200
H	2.06508300	-4.35888500	-4.80337800
H	2.40442800	-5.50362200	-0.22225500
H	2.82736800	-5.14966000	-2.58614400
C	-0.80398900	-4.61630800	0.63132900
C	-1.65294400	-4.65279400	1.78677600
C	0.55086200	-5.07392900	0.78371800
H	2.02446400	-5.87162100	2.16164400
C	1.39441300	-5.11301000	-0.32866900
C	-1.18622300	-5.08462700	2.99484400
H	-2.68496600	-4.33336000	1.69287900
H	-1.84923700	-5.09933200	3.85449200
C	0.16171000	-5.52444300	3.14307000
H	0.51162500	-5.86737700	4.11193700
C	0.99963100	-5.52103700	2.06796400
H	-6.44580100	2.47633500	-3.53083700
C	-6.04858900	2.97185700	-2.65021300
H	-6.16959000	1.22312300	-1.44718600
C	-5.89275200	2.27150700	-1.48897600
C	-5.20131400	4.98751900	-1.62464100

C	-5.34953800	2.89326100	-0.31753700
C	-5.69851400	4.35220700	-2.72268400
C	-5.00226300	4.28507200	-0.39401000
C	-5.12613800	2.18483200	0.87919200
H	-5.83140400	4.89004100	-3.65633200
H	-4.21832500	5.98227500	0.66496800
H	-4.93288500	6.04051000	-1.66391600
C	-4.52880400	2.82745700	1.98204600
C	-4.18185300	2.12767200	3.18444400
C	-4.21147800	4.22584400	1.90262600
H	-3.41178600	5.93815800	2.96473800
C	-4.46249000	4.92290600	0.72237300
C	-3.60332200	2.77662200	4.23520100
H	-4.36205000	1.05972400	3.23709900
H	-3.33797700	2.22457500	5.13179500
C	-3.33237200	4.17551200	4.16911200
H	-2.88612500	4.67642200	5.02355500
C	-3.62414700	4.87366000	3.03536400
C	-5.52914200	-5.39395100	-2.90704000
H	-5.19029300	-5.12882500	-3.91798100
H	-5.21684900	-6.43154400	-2.74262000
C	-6.93732700	-3.14257800	-1.53373500
H	-7.06246000	-2.05505100	-1.57799900
H	-7.57418300	-3.48327900	-0.70630600
C	-7.43373300	-3.79192900	-2.82493100
H	-8.51803500	-3.66406100	-2.90374400
H	-6.98459000	-3.28933100	-3.69147200
C	-7.04885200	-5.26821600	-2.84564900

H	-7.50009300	-5.78366200	-3.69944000
H	-7.42807800	-5.75368400	-1.93634900
C	-6.68765300	-3.34657200	1.84821800
H	-6.58374700	-4.02388100	0.99892300
H	-5.90247000	-3.64569900	2.55875100
C	-8.17008900	-1.09473900	3.10342100
H	-8.10916500	-0.36388000	3.91710300
H	-9.11872000	-0.89665900	2.58486500
C	-8.04703100	-3.52302600	2.52026700
H	-8.84813700	-3.36712100	1.78453200
H	-8.14367700	-4.54901000	2.89018300
C	-8.20055400	-2.51709700	3.65785600
H	-7.37801400	-2.65528300	4.37170900
H	-9.13421000	-2.68162900	4.20556000

Zero-point correction= 1.466540 (Hartree/Particle)

Thermal correction to Energy= 1.531663

Thermal correction to Enthalpy= 1.532465

Thermal correction to Gibbs Free Energy= 1.371237

Sum of electronic and zero-point Energies= -15022.489233

Sum of electronic and thermal Energies= -15022.424109

Sum of electronic and thermal Enthalpies= -15022.423307

Sum of electronic and thermal Free Energies= -15022.584535

AP-8-TS1^{R3}

C	3.34762000	-2.64761000	-0.02191500
C	1.19710200	-1.65710000	-1.55989700
C	3.45644200	-2.42181100	-1.40727200
C	2.13124300	-2.37483200	0.59757800

C	1.02854200	-1.80611600	-0.14153700
C	2.38842100	-1.89466600	-2.17273500
H	0.01643000	-2.00660500	0.20464400
H	2.49649000	-1.72128800	-3.23487200
H	0.33925800	-1.31051600	-2.12008500
C	4.51384100	-3.15070300	0.76013600
C	6.58870500	-4.20990400	2.32733800
C	5.71175800	-2.42173400	0.91744900
C	4.38617100	-4.39201900	1.39030100
C	5.41010600	-4.92768100	2.16176400
C	6.72568000	-2.96718400	1.71634000
H	3.45069900	-4.93107200	1.27577400
H	5.28286700	-5.89537500	2.63764200
H	7.63969100	-2.40940300	1.88454600
H	7.39625300	-4.60588000	2.93524100
O	2.04803200	-2.61972400	1.90482900
O	4.61099600	-2.78720800	-1.95856700
C	0.84755100	-2.29337800	2.61012700
H	-0.01244700	-2.83463300	2.20663100
H	0.66557000	-1.21782700	2.56733400
H	1.02414500	-2.60779800	3.63821900
C	4.93638300	-2.25133100	-3.23644100
H	4.32143000	-2.70483000	-4.02085000
H	5.98157800	-2.51018800	-3.40743200
H	4.81455100	-1.16439400	-3.22436500
P	5.90359800	-0.70466500	0.27906500
O	4.89101200	-0.31719700	-0.76499800
H	-1.47967700	1.70846400	1.10651900

C	-0.00523800	3.14041800	1.34160300
N	1.06589100	2.42385100	1.06462000
C	2.23373000	3.18981300	1.21063400
Br	1.12103500	0.33114100	0.42309100
O	3.34270100	2.79045900	0.95897300
O	-1.21488300	2.76717400	1.27933700
C	1.85594800	4.56954700	1.74549000
H	2.26287000	5.34253400	1.08990700
H	2.31180700	4.69206100	2.73210300
C	0.32914500	4.54655000	1.78892100
H	-0.14063700	5.26812900	1.11375900
H	-0.09613700	4.70036300	2.78431300
C	0.95931800	4.98339700	-1.56185300
C	0.41482300	2.31114600	-2.19920500
C	-0.35911700	4.55687500	-1.61952700
C	2.01401200	4.10804600	-1.78766600
C	1.73644900	2.77860400	-2.08559400
C	-0.61340200	3.22894800	-1.93606400
H	-1.18050100	5.23626700	-1.42132200
H	3.04232600	4.43849500	-1.69514700
O	0.19456200	1.04622100	-2.56177900
H	-0.62130400	0.66577800	-2.13926300
Br	3.15159800	1.54516200	-2.14692000
Br	-2.40053600	2.63298200	-1.95840600
Br	1.31756300	6.79290300	-1.11708500
C	5.72932900	0.34722800	1.77536300
C	4.09348400	1.02840400	3.57690600
C	6.56714300	1.20290400	3.99649700

C	5.20073500	0.92692100	4.62710000
C	6.83938300	0.24710000	2.82932500
C	4.34870000	0.08470200	2.39967000
H	4.04926700	2.05708500	3.19835300
H	6.59371400	2.23760200	3.62787500
H	5.20249300	-0.08341900	5.06015900
H	6.87699800	-0.77720500	3.22311800
H	4.30170200	-0.95523100	2.75551000
H	5.71183600	1.36651400	1.36012100
H	3.11557400	0.80487200	4.02080600
H	7.36242500	1.10895200	4.74477300
H	5.01579100	1.62641500	5.45046200
H	7.82003300	0.46446400	2.38927200
H	3.57362300	0.21243500	1.63851700
C	7.56985200	-0.47613500	-0.49211800
C	8.98305800	1.33769800	-1.56172500
C	10.03994100	-0.83239500	-0.85691500
C	10.24673100	0.67370100	-1.01484600
C	8.83851600	-1.15510000	0.04040500
C	7.77699400	1.04248800	-0.66791100
H	8.78257100	0.95988900	-2.57375400
H	9.87148700	-1.27746000	-1.84753100
H	10.48915800	1.11064900	-0.03576500
H	9.05487100	-0.81716500	1.06306800
H	7.94613400	1.50137500	0.31683600
H	7.37189900	-0.89430500	-1.49211700
H	9.12338300	2.42076400	-1.64812700
H	10.93987700	-1.30397200	-0.44672400

H	11.10025700	0.87284300	-1.67222300
H	8.70155100	-2.23974000	0.07470600
H	6.87023500	1.49425300	-1.08523800
C	-5.42641100	1.12064700	1.08377700
H	-6.85219000	1.73613800	2.55249100
C	-6.45602600	0.88913400	1.99576300
C	-5.47524100	-1.26409900	0.55590400
C	-6.96017000	-0.38553900	2.25406800
C	-4.95059000	0.01781100	0.37413000
C	-6.44507900	-1.48635000	1.55154700
C	-3.56145800	-2.64421000	-0.21087900
C	-4.92943300	-2.36864500	-0.28368900
C	-3.85266700	-4.51464800	-1.65725800
C	-5.74638000	-3.09966800	-1.16965300
C	-3.00312400	-3.75130100	-0.85743700
C	-5.19563600	-4.19054500	-1.85596200
H	-3.44083600	-5.38625600	-2.16249200
O	-3.93142300	0.17228200	-0.54760100
O	-2.76226700	-1.81139100	0.55304100
P	-2.44174200	-0.32546700	-0.07985500
O	-1.60482500	-0.46879300	-1.30442300
O	-2.00310800	0.48510100	1.11840300
H	-0.52100700	-3.45305100	-5.10321700
C	-0.23552600	-3.86413000	-4.13975000
H	-2.02176300	-3.21361400	-3.18184000
C	-1.07717700	-3.73575000	-3.07281600
C	1.41835300	-4.99477800	-2.78969200
C	-0.72362500	-4.25993500	-1.78486300

C	1.02145700	-4.52546600	-4.00576100
C	0.57395600	-4.86225400	-1.64091100
C	-1.58913000	-4.18578000	-0.67356100
H	1.66685900	-4.62983100	-4.87304400
H	1.97279900	-5.76082200	-0.28419200
H	2.38820000	-5.46973700	-2.66099900
C	-1.15852500	-4.64894600	0.59031900
C	-1.99938600	-4.62318600	1.75222400
C	0.16238900	-5.19842100	0.73370000
H	1.59689400	-6.06844600	2.10792800
C	0.98947700	-5.30604800	-0.38633000
C	-1.55188900	-5.07661500	2.95944400
H	-3.00928400	-4.23791900	1.66360700
H	-2.20780100	-5.04435600	3.82407300
C	-0.23238400	-5.59750700	3.10112400
H	0.10459200	-5.95095000	4.07081800
C	0.59430700	-5.65778000	2.01909700
H	-6.41317900	2.91008700	-3.40460500
C	-5.93310800	3.37015000	-2.54627100
H	-6.11858300	1.62908100	-1.33956000
C	-5.76758300	2.65441500	-1.39582700
C	-4.88166700	5.31146100	-1.56673000
C	-5.11564800	3.22779700	-0.25577900
C	-5.48454200	4.72068300	-2.63640900
C	-4.66782800	4.58936700	-0.35003000
C	-4.87399100	2.49565500	0.92271300
H	-5.62693500	5.27157700	-3.56100000
H	-3.68879600	6.21287100	0.66134700

H	-4.53565500	6.34089600	-1.62042200
C	-4.16291300	3.08222400	1.98830400
C	-3.80431900	2.34960100	3.16753500
C	-3.74103500	4.45173400	1.89178300
H	-2.73941600	6.08599800	2.90487000
C	-4.01164800	5.17560500	0.73194100
C	-3.11338600	2.94319300	4.18234700
H	-4.06809200	1.29962300	3.22906100
H	-2.84246600	2.36688700	5.06186300
C	-2.73283000	4.31553900	4.10022000
H	-2.19730700	4.77402900	4.92691700
C	-3.03375300	5.04202800	2.98683300
C	-6.00874300	-5.02589900	-2.82618700
H	-5.68540800	-4.78753500	-3.84883200
H	-5.77520300	-6.08543200	-2.67114100
C	-7.18208100	-2.66687900	-1.41324200
H	-7.21963700	-1.57284900	-1.45851000
H	-7.81498800	-2.95255200	-0.56227700
C	-7.77481400	-3.27623900	-2.68313600
H	-8.84757300	-3.06209700	-2.72383100
H	-7.31836400	-2.81219500	-3.56720000
C	-7.51039900	-4.77872000	-2.71189500
H	-8.03149200	-5.25711000	-3.54728900
H	-7.89468700	-5.23139300	-1.78789900
C	-6.82681100	-2.89759200	1.96287800
H	-6.81876400	-3.57677400	1.10868800
H	-6.03617800	-3.26347800	2.63515900
C	-8.06808400	-0.53707400	3.27968600

H	-7.91535600	0.18641200	4.08787200
H	-9.02019500	-0.26574400	2.80228500
C	-8.16332800	-2.96650400	2.69788300
H	-8.98293700	-2.74697200	1.99973700
H	-8.32346700	-3.98214900	3.07426700
C	-8.18424100	-1.95309000	3.83882300
H	-7.34261900	-2.15596300	4.51397400
H	-9.10139400	-2.04464100	4.42980000

Zero-point correction= 1.466983 (Hartree/Particle)

Thermal correction to Energy= 1.532176

Thermal correction to Enthalpy= 1.532978

Thermal correction to Gibbs Free Energy= 1.371217

Sum of electronic and zero-point Energies= -15022.485910

Sum of electronic and thermal Energies= -15022.420717

Sum of electronic and thermal Enthalpies= -15022.419915

Sum of electronic and thermal Free Energies= -15022.581676

AP-8-TS1*R4*

C	3.45875100	-2.40328800	0.39433500
C	1.27538700	-1.62417300	-1.21987400
C	3.50902100	-2.44899800	-1.01349400
C	2.23648000	-2.11360800	0.99898100
C	1.09869700	-1.66536800	0.21575500
C	2.44561600	-1.95308700	-1.81909300
H	0.11060300	-1.99235300	0.53960300
H	2.54505800	-1.90048500	-2.89494300
H	0.41779700	-1.31622800	-1.80293100
C	4.66542500	-2.71805400	1.20776200

C	6.82606500	-3.32758000	2.89275000
C	5.85343800	-1.96672200	1.11474500
C	4.59399100	-3.77193100	2.12681700
C	5.65919500	-4.08295200	2.96023200
C	6.91222900	-2.28507500	1.97833900
H	3.66916900	-4.33802500	2.18809400
H	5.57673100	-4.90560500	3.66406700
H	7.83004700	-1.70340100	1.93967400
H	7.66522700	-3.54761400	3.54516100
O	2.18090500	-2.23641700	2.31908500
O	4.57220200	-3.04923300	-1.52317100
C	0.98293100	-1.97050200	3.05638400
H	0.10500300	-2.41671900	2.58187100
H	0.84484000	-0.89564600	3.16716300
H	1.14196700	-2.43456600	4.02883300
C	4.82281900	-2.92739900	-2.92005300
H	4.03060500	-3.41316700	-3.49828300
H	5.76563700	-3.44557400	-3.09455400
H	4.91690000	-1.87135900	-3.18336700
P	6.10937000	-0.51929700	-0.00713100
O	5.23829600	-0.46806100	-1.23962100
H	-1.90892500	1.66625300	1.05371700
C	-0.47783300	3.21598400	1.63185100
N	0.63249600	2.48787900	1.56445000
C	1.71030800	3.16882600	2.10788900
Br	0.93363100	0.39681300	0.79971600
O	2.82576400	2.70927200	2.22651800
O	-1.62442600	2.86621900	1.26233000

C	1.26913900	4.56533000	2.54285000
H	1.82604500	5.30814200	1.96520500
H	1.51761900	4.70745100	3.59740500
C	-0.22938900	4.58119000	2.25125600
H	-0.53933200	5.36748400	1.55676900
H	-0.85297000	4.66906300	3.14531600
C	0.99881600	4.95174000	-0.95557400
C	0.51061100	2.33617300	-1.81963400
C	-0.30788700	4.54751100	-1.18269200
C	2.06751400	4.07834800	-1.12094700
C	1.81545200	2.77882800	-1.54148200
C	-0.53488900	3.24410000	-1.60427300
H	-1.14278400	5.22150000	-1.02600400
H	3.08231200	4.39629500	-0.90918000
O	0.32568000	1.09885800	-2.28718200
H	-0.52355900	0.70446000	-1.96390000
Br	3.23142100	1.53841100	-1.63480700
Br	-2.31595200	2.66802000	-1.83310300
Br	1.32212800	6.73588600	-0.39469100
C	5.81596700	0.95392700	1.05757500
C	5.97150100	2.14625800	3.27394600
C	5.84004400	3.47734800	1.13932200
C	6.38913900	3.43658800	2.56630000
C	6.28188700	2.24154600	0.35474200
C	6.38006200	0.89701200	2.48407000
H	4.88145200	2.14576300	3.38651900
H	4.74486500	3.50238500	1.17644600
H	7.48705600	3.49900600	2.53445200

H	7.37981700	2.23816200	0.29744400
H	7.47881600	0.83235600	2.44097400
H	4.71396700	0.98409000	1.11078900
H	6.40858000	2.10253300	4.27846700
H	6.18088400	4.38292900	0.62334100
H	6.03718000	4.30517700	3.13428800
H	5.89247100	2.27645200	-0.66792100
H	6.02357400	0.00394700	3.00583000
C	7.88544900	-0.62936900	-0.52666100
C	9.69339800	-2.15516900	-1.45883800
C	9.56055200	0.22747400	-2.23516900
C	9.93892400	-1.19991700	-2.62525700
C	8.11598600	0.30512000	-1.73350800
C	8.25294400	-2.06684400	-0.94701600
H	10.37883600	-1.90598400	-0.63633900
H	10.23906800	0.58105100	-1.44602200
H	9.32615000	-1.51230400	-3.48223000
H	7.42767900	0.00016200	-2.53130700
H	7.55999200	-2.37257700	-1.74443500
H	8.53009500	-0.30962700	0.30624700
H	9.91519000	-3.18729100	-1.75285400
H	9.68704700	0.90400100	-3.08770200
H	10.98544300	-1.24779900	-2.94627900
H	7.86786000	1.33968400	-1.49028400
H	8.11423500	-2.77392900	-0.12628900
C	-5.73523700	0.95554100	0.48288300
H	-7.36463000	1.65404400	1.67659500
C	-6.82738300	0.77349700	1.32973800

C	-5.49966900	-1.47552100	0.40240800
C	-7.22554100	-0.48561400	1.78185000
C	-5.07936400	-0.19397400	0.04499100
C	-6.54187800	-1.62774400	1.33702100
C	-3.42791800	-2.78940300	-0.02487400
C	-4.80456500	-2.64126500	-0.21532000
C	-3.48428300	-4.86650000	-1.18926800
C	-5.50346500	-3.55688300	-1.02881100
C	-2.74711300	-3.92899800	-0.46605600
C	-4.82980500	-4.68722100	-1.51062700
H	-2.97660600	-5.76536000	-1.53374100
O	-3.97637300	-0.08183800	-0.78538100
O	-2.74645400	-1.79571700	0.66178200
P	-2.52342100	-0.36420900	-0.10391800
O	-1.52551800	-0.49042400	-1.19604300
O	-2.34222400	0.59061800	1.07292500
H	-0.06356100	-4.05545500	-4.58993400
C	0.19367200	-4.31100100	-3.56660000
H	-1.66550700	-3.62975200	-2.78758000
C	-0.70339700	-4.07870700	-2.56434600
C	1.83056000	-5.15190600	-2.00039000
C	-0.39291200	-4.40455100	-1.20183400
C	1.47070000	-4.88130900	-3.28735000
C	0.92245300	-4.91184200	-0.91945400
C	-1.32088000	-4.23526900	-0.15210600
H	2.15751500	-5.08199800	-4.10469900
H	2.28567900	-5.55834600	0.60835700
H	2.81330900	-5.55460000	-1.76563500

C	-0.93920600	-4.51536700	1.17974900
C	-1.84087100	-4.38668900	2.28755200
C	0.39364500	-4.98114400	1.45408000
H	1.77963500	-5.63674700	2.98699800
C	1.28858800	-5.17419100	0.39998900
C	-1.44450900	-4.68419800	3.55970700
H	-2.85626000	-4.05174800	2.10511100
H	-2.14675800	-4.57869700	4.38091800
C	-0.12027200	-5.14002100	3.82604000
H	0.17170300	-5.37488900	4.84509900
C	0.76903400	-5.28041600	2.80264400
H	-6.65428600	1.71319000	-4.29491800
C	-6.26423200	2.38058900	-3.53254200
H	-6.31498300	0.89338800	-2.01496800
C	-6.07257800	1.92191100	-2.26184200
C	-5.46544500	4.57832600	-2.92856300
C	-5.54580400	2.77505300	-1.23867800
C	-5.95613100	3.72954000	-3.87523600
C	-5.24165800	4.13499900	-1.58754900
C	-5.31240800	2.32648900	0.07866500
H	-6.11232900	4.07299600	-4.89321100
H	-4.47010400	6.01636500	-0.89409600
H	-5.22174400	5.60848500	-3.17551500
C	-4.75575500	3.19265400	1.03879600
C	-4.48677400	2.77922800	2.38550800
C	-4.44148200	4.54612300	0.67176900
H	-3.66516200	6.44784900	1.36669000
C	-4.70015900	4.98714700	-0.62468700

C	-3.98062400	3.65328600	3.30078300
H	-4.67906500	1.74872400	2.66369200
H	-3.78695100	3.32105200	4.31651300
C	-3.68338300	5.00007900	2.93626500
H	-3.28538400	5.68249200	3.68221200
C	-3.89249200	5.42523200	1.65890200
C	-5.51026500	-5.71985800	-2.38847600
H	-5.13453000	-5.60739200	-3.41474200
H	-5.21044900	-6.72197600	-2.06144900
C	-6.94708400	-3.28574800	-1.41678800
H	-7.06374700	-2.21708700	-1.62776100
H	-7.61362700	-3.49770400	-0.57009300
C	-7.40392300	-4.11990800	-2.61290100
H	-8.48388200	-4.00231600	-2.74718200
H	-6.92218600	-3.75341500	-3.52877700
C	-7.03009000	-5.58371500	-2.40006800
H	-7.45515500	-6.21953900	-3.18312500
H	-7.44394400	-5.92754200	-1.44260000
C	-6.83700100	-2.98283400	1.95547500
H	-6.67829600	-3.78989300	1.23783400
H	-6.09229400	-3.14544500	2.74894700
C	-8.41170800	-0.57867300	2.72378600
H	-8.40243500	0.28006300	3.40379200
H	-9.33174500	-0.48805000	2.12943200
C	-8.23194100	-3.06804000	2.57003900
H	-8.99013900	-3.05326500	1.77482900
H	-8.34142700	-4.01825500	3.10281700
C	-8.45944600	-1.88697200	3.50955700

H -7.67713800 -1.88617500 4.27973800

H -9.42073800 -1.97430900 4.02620600

Zero-point correction= 1.465132 (Hartree/Particle)

Thermal correction to Energy= 1.530497

Thermal correction to Enthalpy= 1.531298

Thermal correction to Gibbs Free Energy= 1.369482

Sum of electronic and zero-point Energies= -15022.489494

Sum of electronic and thermal Energies= -15022.424130

Sum of electronic and thermal Enthalpies= -15022.423328

Sum of electronic and thermal Free Energies= -15022.585144

AP-8-TS1*R5*

C 3.43289500 -2.41732400 0.26817900

C 1.20667800 -1.64080700 -1.28547000

C 3.45026200 -2.45085200 -1.14084700

C 2.22435600 -2.13928100 0.90411200

C 1.06655300 -1.69229600 0.15248700

C 2.36570700 -1.95355500 -1.91565200

H 0.08588600 -2.01196800 0.50426500

H 2.43705600 -1.89046600 -2.99309100

H 0.33266300 -1.33610600 -1.84536400

C 4.65384800 -2.75729500 1.04986900

C 6.84223800 -3.46201700 2.66051100

C 5.84418300 -2.00365600 0.97510700

C 4.59397800 -3.85248200 1.91855100

C 5.67273300 -4.21174600 2.71558700

C 6.91535300 -2.37386200 1.79827800

H 3.66672000 -4.41563000 1.96965700

H	5.59738900	-5.06760100	3.37951700
H	7.82962900	-1.79258200	1.77728100
H	7.69390400	-3.71847800	3.28276800
O	2.20062100	-2.27253200	2.22523000
O	4.50117900	-3.04489900	-1.68224700
C	1.01566700	-2.01578600	2.98571800
H	0.13600200	-2.48621400	2.53892800
H	0.85902000	-0.94184500	3.08195800
H	1.20487900	-2.46034700	3.96198600
C	4.71897400	-2.90121100	-3.08230100
H	3.91065600	-3.37241200	-3.65021200
H	5.65387900	-3.42194700	-3.28878100
H	4.81339200	-1.84116900	-3.32974300
P	6.08287400	-0.51686800	-0.09450800
O	5.21922400	-0.48480900	-1.33374500
H	-1.88672900	1.67638400	1.07180000
C	-0.42728100	3.20519800	1.59155400
N	0.67407400	2.47199800	1.47130700
C	1.78794800	3.16004300	1.92620000
Br	0.92764500	0.37906200	0.73182200
O	2.91279400	2.70887300	1.94214400
O	-1.59304000	2.85924200	1.28091100
C	1.37079200	4.54823400	2.40799400
H	1.90494400	5.30408200	1.82660200
H	1.66409500	4.66309000	3.45464900
C	-0.14027400	4.57309200	2.18695100
H	-0.47381000	5.35549500	1.49898000
H	-0.72229900	4.67575300	3.10700400

C	0.98539200	4.93875700	-1.04677900
C	0.45899700	2.31918900	-1.87666200
C	-0.32941800	4.54002000	-1.23445600
C	2.04393300	4.05693500	-1.23065200
C	1.77297200	2.75501400	-1.63220200
C	-0.57547000	3.23480300	-1.63911800
H	-1.15581600	5.22053400	-1.06161000
H	3.06565400	4.36918800	-1.04558100
O	0.25523100	1.08124300	-2.33370000
H	-0.58827600	0.69216200	-1.98837100
Br	3.17877800	1.50446000	-1.73620000
Br	-2.36584700	2.66807100	-1.81088300
Br	1.33283300	6.72628000	-0.51168400
C	5.77916400	0.96153900	0.94610100
C	6.03508800	2.20163300	3.13054200
C	5.96391100	3.48876100	0.97055200
C	6.56447800	3.42263900	2.37590400
C	6.26590000	2.21158100	0.18470700
C	6.33323900	0.90094800	2.37720200
H	4.94954100	2.30297100	3.24363000
H	4.87754300	3.60633400	1.05485700
H	7.66109100	3.36335100	2.30370700
H	7.34939000	2.15032300	0.01493600
H	7.42024100	0.73716100	2.35687100
H	4.67881000	1.01727500	0.99470200
H	6.47114000	2.15144700	4.13514100
H	6.35537000	4.35573300	0.42517900
H	6.33186400	4.33824900	2.93144400

H	5.78544000	2.24685500	-0.79819400
H	5.89447600	0.05129600	2.91119300
C	7.84046700	-0.53002600	-0.68302200
C	10.28883700	0.02226000	-0.69187300
C	9.40173400	-1.76660300	-2.23742600
C	10.59675100	-1.29517800	-1.40635500
C	8.13510400	-1.86806600	-1.38327300
C	9.03533200	-0.09986700	0.18429600
H	10.13010100	0.81412200	-1.43688100
H	9.22516700	-1.05390300	-3.05454100
H	10.83729900	-2.06108100	-0.65553500
H	8.26550900	-2.65324500	-0.62616100
H	9.24164400	-0.84097900	0.96536700
H	7.74128000	0.22847500	-1.47667800
H	11.14124000	0.33008600	-0.07618000
H	9.61882400	-2.73569700	-2.70073100
H	11.48240800	-1.18481500	-2.04174100
H	7.27750500	-2.15589500	-2.00143500
H	8.84169300	0.85078600	0.68934200
C	-5.73599300	0.98871800	0.58943400
H	-7.32858500	1.69571500	1.82713700
C	-6.80838000	0.81219100	1.46231900
C	-5.52502300	-1.44393800	0.49069300
C	-7.20684200	-0.44564500	1.91772800
C	-5.10188300	-0.16436300	0.12916400
C	-6.54501300	-1.59155900	1.45021800
C	-3.47663400	-2.77378500	0.00357900
C	-4.85646300	-2.61254800	-0.15067200

C	-3.58098700	-4.84159800	-1.17425200
C	-5.58409300	-3.51651100	-0.95189300
C	-2.81727700	-3.91560800	-0.46390200
C	-4.93291500	-4.64901900	-1.45889500
H	-3.08995700	-5.74176300	-1.53883700
O	-4.01925100	-0.05799800	-0.72777200
O	-2.76972700	-1.79090600	0.67948800
P	-2.55174100	-0.35625400	-0.08312600
O	-1.58407900	-0.48930500	-1.20175000
O	-2.33158800	0.59246800	1.09026200
H	-0.24595000	-4.00588100	-4.65955800
C	0.03712500	-4.27576200	-3.64677900
H	-1.79875300	-3.59945900	-2.81071300
C	-0.83255200	-4.05384700	-2.61846700
C	1.71102400	-5.14406700	-2.13575700
C	-0.48715600	-4.39751400	-1.26878800
C	1.31908200	-4.85393400	-3.40886500
C	0.83270900	-4.91478100	-1.02822300
C	-1.38517900	-4.23518900	-0.19232800
H	1.98383700	-5.04507700	-4.24648200
H	2.23224000	-5.58980600	0.45348100
H	2.69804100	-5.55326400	-1.93213400
C	-0.96924500	-4.53364100	1.12516400
C	-1.83966100	-4.41208500	2.25844500
C	0.36760700	-5.01090100	1.35758700
H	1.79040700	-5.69220500	2.84507000
C	1.23263900	-5.19618900	0.27731900
C	-1.41089000	-4.72685400	3.51590900

H	-2.85731400	-4.06835800	2.10727900
H	-2.08984300	-4.62645800	4.35711000
C	-0.08279000	-5.19363300	3.74080600
H	0.23545700	-5.44176100	4.74878600
C	0.77738300	-5.32798500	2.69199900
H	-6.76644900	1.78696300	-4.15945600
C	-6.35057500	2.44542500	-3.40297800
H	-6.37896000	0.94900100	-1.89385300
C	-6.13208200	1.97655800	-2.14036000
C	-5.51348700	4.63064400	-2.80568000
C	-5.57122100	2.81757900	-1.12527800
C	-6.03686900	3.79327100	-3.74496200
C	-5.26106900	4.17636400	-1.47344000
C	-5.30975000	2.35803900	0.18300800
H	-6.21474900	4.14487800	-4.75658100
H	-4.45153300	6.04484300	-0.78845800
H	-5.26507200	5.65975900	-3.05232500
C	-4.72038200	3.21246700	1.13422500
C	-4.42299000	2.78788700	2.47146300
C	-4.40001200	4.56460700	0.76747500
H	-3.58370800	6.45270700	1.45360300
C	-4.68615200	5.01651900	-0.51947800
C	-3.88341600	3.65022500	3.37874300
H	-4.62058600	1.75804600	2.74842900
H	-3.66843000	3.30956200	4.38735000
C	-3.57869000	4.99539000	3.01447800
H	-3.15363900	5.66842500	3.75401000
C	-3.81560300	5.43100300	1.74547300

C	-5.64464100	-5.66963600	-2.32592200
H	-5.29456000	-5.55316300	-3.36078900
H	-5.34517200	-6.67654300	-2.01360500
C	-7.03485800	-3.23040900	-1.30076400
H	-7.14773000	-2.15929600	-1.50114100
H	-7.68127700	-3.44295800	-0.43873500
C	-7.52950600	-4.05217100	-2.49042200
H	-8.61150800	-3.92446800	-2.59599000
H	-7.06830500	-3.68331400	-3.41586700
C	-7.16299600	-5.52054400	-2.29725700
H	-7.61367400	-6.14725500	-3.07331600
H	-7.55497500	-5.86754800	-1.33174600
C	-6.83713300	-2.94720600	2.06887700
H	-6.70369100	-3.75174100	1.34327700
H	-6.07444600	-3.12086200	2.84268500
C	-8.37017500	-0.53300600	2.88823000
H	-8.33640100	0.32195500	3.57220700
H	-9.30373200	-0.43093800	2.31725000
C	-8.21711500	-3.02309700	2.71747600
H	-8.99464000	-2.99727500	1.94143400
H	-8.32208400	-3.97506500	3.24803500
C	-8.41049000	-1.84497100	3.66830100
H	-7.60936800	-1.85531900	4.41881600
H	-9.35949200	-1.92633400	4.20815000

Zero-point correction= 1.465040 (Hartree/Particle)

Thermal correction to Energy= 1.530445

Thermal correction to Enthalpy= 1.531247

Thermal correction to Gibbs Free Energy= 1.369693

Sum of electronic and zero-point Energies= -15022.487824

Sum of electronic and thermal Energies= -15022.422419

Sum of electronic and thermal Enthalpies= -15022.421617

Sum of electronic and thermal Free Energies= -15022.583171

AP-8-TS1*R6*

C	3.57248400	-2.40916200	0.08071500
C	1.16376600	-1.59816500	-1.15547100
C	3.41268300	-2.37693300	-1.32480400
C	2.46869400	-2.10048600	0.87479500
C	1.21556600	-1.65828000	0.28656500
C	2.23883300	-1.86473500	-1.93793600
H	0.30853700	-2.04520000	0.75154200
H	2.16431700	-1.79103200	-3.01462600
H	0.21299700	-1.32652700	-1.59310600
C	4.83664900	-2.96034100	0.65008300
C	7.13988400	-4.26155200	1.58047700
C	6.10729200	-2.40537800	0.37952000
C	4.75037200	-4.12369400	1.41991200
C	5.88633500	-4.76995100	1.89353400
C	7.24018900	-3.08860700	0.83676500
H	3.76547900	-4.52020200	1.64605400
H	5.78971500	-5.67265900	2.48906000
H	8.23165500	-2.71107300	0.61693800
H	8.04037100	-4.76527700	1.91777400
O	2.62312900	-2.25348200	2.18459000
O	4.38519000	-2.93259900	-2.02326500
C	1.57107700	-1.96665900	3.11252000

H	0.60183200	-2.32903600	2.75886400
H	1.52877300	-0.89409700	3.30265700
H	1.84394800	-2.50151100	4.02174600
C	4.46340400	-2.66763300	-3.42151400
H	3.64973300	-3.17049200	-3.95350800
H	5.42108200	-3.07768400	-3.74048400
H	4.44821000	-1.59007000	-3.59443400
P	6.29450700	-0.73938300	-0.39561100
O	5.52922800	-0.52816200	-1.68143400
H	-2.02617600	1.66888000	0.96436100
C	-0.59820300	3.23264200	1.65946500
N	0.47690900	2.44156600	1.74807400
C	1.46579900	3.04358200	2.50967700
Br	0.90429100	0.39421700	0.92360200
O	2.47674300	2.49430400	2.89022600
O	-1.69202200	2.94674100	1.13304200
C	1.08068700	4.49075900	2.80382600
H	1.74685200	5.14437600	2.23172700
H	1.22759500	4.70437000	3.86445000
C	-0.36601100	4.57739900	2.33152600
H	-0.56305500	5.38769400	1.62382800
H	-1.08616600	4.67248300	3.14930800
C	1.11639500	4.86076800	-0.82117700
C	0.55916400	2.28889200	-1.76595500
C	-0.19329900	4.51978800	-1.12181000
C	2.15703800	3.94996600	-0.97120300
C	1.86943600	2.67436800	-1.43628100
C	-0.45567800	3.23642800	-1.58381200

H	-1.00657600	5.22398100	-0.98553000
H	3.17505400	4.22302900	-0.71681600
O	0.34490300	1.06456800	-2.25437800
H	-0.54082600	0.71746800	-1.98486500
Br	3.25280600	1.39984200	-1.58845200
Br	-2.24512700	2.74344900	-1.92414400
Br	1.48996900	6.60939400	-0.18635900
C	5.68802700	0.41809000	0.88682700
C	5.26685400	1.08411500	3.25889600
C	5.22746300	2.82104800	1.42072600
C	5.52376800	2.54924100	2.89779400
C	6.01678500	1.87529000	0.51676500
C	6.04474500	0.12719200	2.35015100
H	4.19470200	0.88365500	3.14911300
H	4.15712700	2.66583400	1.23423600
H	6.57608800	2.79636800	3.10485900
H	7.09184700	2.05638900	0.65024600
H	7.12320000	0.26102300	2.50813200
H	4.59597400	0.30061200	0.77622200
H	5.53125100	0.89938200	4.30685800
H	5.46093700	3.86354800	1.17055100
H	4.90261700	3.19524200	3.52480600
H	5.78194900	2.06279800	-0.53559400
H	5.80541800	-0.91086100	2.60688000
C	8.10622000	-0.52904200	-0.69951000
C	9.80173500	0.52345000	-2.24017200
C	10.47133900	-0.17255100	0.09184100
C	10.71480400	0.81801900	-1.04839700

C	8.99629500	-0.18456300	0.50743800
C	8.32795600	0.49699600	-1.82682700
H	10.07059900	-0.45197500	-2.66967500
H	10.76292300	-1.18176700	-0.23270700
H	10.51387500	1.83548400	-0.68502900
H	8.72785100	0.81158000	0.87961000
H	8.03024000	1.49426500	-1.47696600
H	8.42523100	-1.50477600	-1.09931500
H	9.95233900	1.26922100	-3.02858100
H	11.09790700	0.07716700	0.95521900
H	11.76587900	0.79236000	-1.35658000
H	8.84298100	-0.87584800	1.34214200
H	7.68532300	0.25513200	-2.67715000
C	-5.80074500	1.01870700	0.44529000
H	-7.40977500	1.77842000	1.63025600
C	-6.87666800	0.88157300	1.32082600
C	-5.56156900	-1.41613300	0.49170700
C	-7.26234000	-0.35248200	1.84787300
C	-5.15532300	-0.15421000	0.05794200
C	-6.58411200	-1.51704200	1.45484000
C	-3.49641200	-2.74688900	0.06127300
C	-4.88009000	-2.61145200	-0.08550400
C	-3.57523900	-4.86595500	-1.01793600
C	-5.59995200	-3.56615500	-0.83346700
C	-2.81937200	-3.89306300	-0.36369400
C	-4.93449800	-4.71216100	-1.29014200
H	-3.07156200	-5.77131100	-1.35088300
O	-4.06676800	-0.08068000	-0.79716300

O	-2.79343900	-1.72675500	0.68505200
P	-2.60919800	-0.32284000	-0.12424200
O	-1.61668300	-0.42556600	-1.21980600
O	-2.44253300	0.65563900	1.04519000
H	-0.37655700	-3.97337600	-4.62710800
C	-0.06177300	-4.23666500	-3.62197800
H	-1.87460500	-3.56477000	-2.73458000
C	-0.90064000	-4.01318900	-2.56927100
C	1.65933000	-5.09760000	-2.15957500
C	-0.51002100	-4.34294300	-1.22813000
C	1.22931000	-4.81056000	-3.42070100
C	0.81584100	-4.86089900	-1.02683700
C	-1.37114800	-4.16799700	-0.12431200
H	1.86786700	-5.00540700	-4.27750500
H	2.24941800	-5.55626000	0.40805500
H	2.65175800	-5.50634700	-1.98420600
C	-0.90405100	-4.42930400	1.18434100
C	-1.72254800	-4.25571200	2.34825900
C	0.43341400	-4.92385600	1.37497100
H	1.89872400	-5.60836700	2.81884600
C	1.25394900	-5.14087200	0.26588500
C	-1.24838500	-4.54726300	3.59522600
H	-2.73633400	-3.88832400	2.22984300
H	-1.88736800	-4.40447800	4.46119600
C	0.07478600	-5.04565700	3.77726300
H	0.42759900	-5.27874100	4.77730700
C	0.88825400	-5.22503200	2.69822200
H	-6.65142300	1.47105700	-4.38705200

C	-6.26353400	2.18320200	-3.66511800
H	-6.35779300	0.80148700	-2.05405000
C	-6.09733800	1.80828400	-2.36383700
C	-5.44819800	4.40691200	-3.19575600
C	-5.57585100	2.72109200	-1.39047700
C	-5.93220200	3.50207000	-4.09249900
C	-5.25646500	4.05299200	-1.82322600
C	-5.37296000	2.36155500	-0.04038700
H	-6.06634900	3.77789000	-5.13390700
H	-4.49439500	5.97552700	-1.24184200
H	-5.18892200	5.41604900	-3.50567100
C	-4.83096900	3.28939000	0.87008900
C	-4.58239800	2.96653900	2.24542400
C	-4.50855600	4.61594900	0.42076800
H	-3.75064500	6.56249300	1.00183200
C	-4.73561300	4.96813100	-0.90772800
C	-4.09456500	3.90071900	3.10992600
H	-4.77663400	1.95638000	2.58896600
H	-3.91702800	3.63596600	4.14831400
C	-3.79629800	5.22278400	2.66402100
H	-3.41376900	5.95404000	3.37067100
C	-3.98135900	5.56111500	1.35769200
C	-5.63744800	-5.78746400	-2.09641700
H	-5.29488700	-5.72148000	-3.13818400
H	-5.32140600	-6.77177600	-1.73272000
C	-7.05733400	-3.32042000	-1.18572900
H	-7.18841300	-2.26240000	-1.43794600
H	-7.69519200	-3.50022200	-0.31014000

C	-7.54625800	-4.20742800	-2.32998900
H	-8.63049300	-4.10031200	-2.43509900
H	-7.09544400	-3.87885500	-3.27551800
C	-7.15760100	-5.65880900	-2.06560700
H	-7.60328500	-6.33029800	-2.80622700
H	-7.53918500	-5.96224600	-1.08150000
C	-6.86249200	-2.83518900	2.15510500
H	-6.70943000	-3.68178000	1.48322400
H	-6.10522000	-2.94759700	2.94524500
C	-8.43186900	-0.39604900	2.81409000
H	-8.41316600	0.49856100	3.44589200
H	-9.36173000	-0.33891600	2.23099800
C	-8.24730700	-2.89043100	2.79526300
H	-9.01804600	-2.92245000	2.01271700
H	-8.34450700	-3.80961000	3.38205500
C	-8.46390800	-1.65937300	3.67110600
H	-7.67029500	-1.61459600	4.42825900
H	-9.41710200	-1.71982200	4.20622800

Zero-point correction= 1.466695 (Hartree/Particle)

Thermal correction to Energy= 1.531907

Thermal correction to Enthalpy= 1.532708

Thermal correction to Gibbs Free Energy= 1.371410

Sum of electronic and zero-point Energies= -15022.485644

Sum of electronic and thermal Energies= -15022.420433

Sum of electronic and thermal Enthalpies= -15022.419631

Sum of electronic and thermal Free Energies= -15022.580929

AP-8-TS1[R7]

C	3.45282200	-2.50951000	0.23864800
C	1.30365600	-1.58434600	-1.33882400
C	3.57415400	-2.30798800	-1.15112500
C	2.22251700	-2.24962800	0.83864100
C	1.11719400	-1.71421700	0.07992500
C	2.50758900	-1.80062500	-1.93289700
H	0.10732300	-1.94852100	0.41179800
H	2.62189700	-1.63887900	-2.99588400
H	0.44522400	-1.27515100	-1.91792700
C	4.60792100	-3.01851400	1.03095300
C	6.69932800	-4.07785300	2.57773700
C	5.81950100	-2.31426800	1.14401000
C	4.46699900	-4.23834200	1.70269100
C	5.49782600	-4.77157700	2.46455900
C	6.84544200	-2.85748100	1.92937500
H	3.51798300	-4.75924900	1.62743500
H	5.36208700	-5.72146400	2.97281600
H	7.78105300	-2.31380400	2.04304300
H	7.51390100	-4.47824500	3.17322800
O	2.12306200	-2.49041300	2.14545700
O	4.72831600	-2.68916800	-1.68914200
C	0.89933800	-2.20005600	2.82682100
H	0.06640200	-2.77381500	2.41141000
H	0.68066700	-1.13175500	2.77363100
H	1.06814800	-2.50148300	3.85997800
C	5.03441100	-2.23631400	-3.00329700
H	4.36992300	-2.69612700	-3.74182500

H	6.05687200	-2.55924300	-3.19747000
H	4.96865600	-1.14519000	-3.04358000
P	6.07601200	-0.60616200	0.49887400
O	5.16939700	-0.17896900	-0.62439200
H	-1.62763700	1.65696400	1.13690400
C	-0.24548900	3.20770400	1.38141300
N	0.88579900	2.55676800	1.17365500
C	1.98406200	3.41616700	1.31123500
Br	1.09934700	0.43890400	0.61608600
O	3.12505500	3.10663300	1.06958600
O	-1.42052000	2.75151500	1.28872100
C	1.49735700	4.77204500	1.81253900
H	1.89332700	5.56506900	1.17531700
H	1.89092700	4.92305400	2.82234000
C	-0.02433800	4.65187400	1.77857200
H	-0.49747100	5.30822200	1.04172800
H	-0.51563400	4.82478400	2.73955800
C	0.88965400	5.01481200	-1.56091700
C	0.45581200	2.30384800	-2.10603900
C	-0.41183600	4.54327400	-1.65217000
C	1.97931500	4.16541800	-1.70399800
C	1.75658400	2.81592100	-1.95498700
C	-0.61146600	3.19597800	-1.91961500
H	-1.26133300	5.20413900	-1.51993300
H	2.99149300	4.53209700	-1.57804900
O	0.28910300	1.02108500	-2.43106100
H	-0.54031300	0.63525700	-2.04342500
Br	3.21413100	1.62767100	-1.91620300

Br	-2.37667300	2.53861200	-1.97739200
Br	1.17044700	6.85408900	-1.18830000
C	5.86046100	0.33909100	2.06201600
C	6.24781400	2.40426200	3.43464200
C	4.09957900	1.10043700	3.71625500
C	4.76175400	2.47939800	3.78585300
C	4.35712500	0.39432200	2.38116400
C	6.46409100	1.74880600	2.06622500
H	6.77956600	1.81659400	4.19712400
H	4.48533000	0.47401400	4.53377400
H	4.27110600	3.14934800	3.07206400
H	3.84660100	0.92264700	1.56853800
H	5.98139400	2.35253300	1.28578000
H	6.37317200	-0.25428500	2.83543800
H	6.69020600	3.40694900	3.44054300
H	3.01699100	1.19106100	3.87075400
H	4.63177300	2.90877100	4.78658100
H	3.93775300	-0.61949700	2.42237700
H	7.53745700	1.71102100	1.84608600
C	7.84610700	-0.50516000	-0.01282500
C	9.73036900	-1.58840300	-1.31571400
C	9.48820600	0.91683100	-1.31992300
C	9.93971800	-0.30513000	-2.11977800
C	8.03640500	0.77101400	-0.85755700
C	8.28121100	-1.72680200	-0.84065700
H	10.39286400	-1.57813900	-0.43852300
H	10.13914100	1.03876800	-0.44255800
H	9.35311800	-0.36216300	-3.04720900

H	7.37220300	0.70227000	-1.72796400
H	7.61586000	-1.80188900	-1.71200800
H	8.47019500	-0.43448200	0.89145700
H	10.00911000	-2.46461700	-1.91188900
H	9.59303400	1.82668400	-1.92119900
H	10.99078500	-0.20569400	-2.41291700
H	7.72511100	1.66222100	-0.30539500
H	8.16192300	-2.65468500	-0.27438900
C	-5.53918200	0.96385500	0.90023000
H	-7.08060700	1.55039200	2.25995600
C	-6.61598800	0.70908000	1.74944800
C	-5.46212200	-1.43067700	0.42132600
C	-7.08598000	-0.57961400	2.00270000
C	-4.97573800	-0.13268900	0.24759800
C	-6.48464500	-1.67214900	1.35806200
C	-3.45780900	-2.75128100	-0.20890200
C	-4.82806300	-2.52780900	-0.36416300
C	-3.59415400	-4.65063000	-1.63868000
C	-5.56439100	-3.30170100	-1.28364900
C	-2.82058200	-3.84301200	-0.80554000
C	-4.93487300	-4.38132400	-1.91828300
H	-3.12144300	-5.51194500	-2.10684100
O	-3.90724800	0.04722300	-0.61210500
O	-2.73499500	-1.87734000	0.58529000
P	-2.43194800	-0.39538800	-0.05744900
O	-1.52622400	-0.52264400	-1.23245100
O	-2.08186600	0.44328200	1.15346000
H	-0.15981800	-3.52869100	-4.93700000

C	0.09589200	-3.91082500	-3.95347200
H	-1.75038600	-3.30026800	-3.08832600
C	-0.79528500	-3.78848200	-2.92683200
C	1.72378300	-4.96012800	-2.50916700
C	-0.48309500	-4.27655900	-1.61405700
C	1.36587000	-4.52859300	-3.75119600
C	0.82513400	-4.83325400	-1.40161900
C	-1.40050200	-4.21368700	-0.54424300
H	2.05022700	-4.63236900	-4.58821500
H	2.18593400	-5.67294700	0.02831400
H	2.70090400	-5.40228800	-2.32864300
C	-1.01092300	-4.63670700	0.74703700
C	-1.90094000	-4.61333200	1.87168400
C	0.31762900	-5.14536700	0.95744200
H	1.71037100	-5.96241700	2.40579900
C	1.19642300	-5.24625000	-0.12310600
C	-1.49421000	-5.03419500	3.10495000
H	-2.91585800	-4.25691200	1.73380700
H	-2.18720700	-5.00374900	3.94019200
C	-0.17018200	-5.52054300	3.31154900
H	0.13252100	-5.85172900	4.30020700
C	0.70387200	-5.57609300	2.26720800
H	-6.29832900	2.61148300	-3.68649500
C	-5.89269700	3.10917600	-2.81095800
H	-6.08888300	1.38918400	-1.57628300
C	-5.77455600	2.42621700	-1.63498300
C	-4.98146600	5.11178700	-1.81471800
C	-5.22106400	3.05047900	-0.46983500

C	-5.49130000	4.47409500	-2.90569300
C	-4.82026800	4.42629600	-0.56921800
C	-5.03162800	2.35624500	0.74098500
H	-5.59483100	4.99837400	-3.85069600
H	-3.97459800	6.11039300	0.46320600
H	-4.67252700	6.15277400	-1.87153200
C	-4.41759000	2.99528100	1.83618400
C	-4.11229100	2.30566900	3.05568600
C	-4.04333300	4.37807700	1.73267500
H	-3.17995800	6.07564900	2.76864600
C	-4.26110100	5.06282700	0.53852000
C	-3.51772800	2.95111600	4.09946500
H	-4.33805900	1.24747300	3.12720500
H	-3.28550800	2.40714600	5.01009200
C	-3.18672500	4.33564200	4.00789500
H	-2.72790100	4.83464200	4.85687100
C	-3.43783800	5.02265800	2.85772500
C	-5.65873100	-5.26061000	-2.92003600
H	-5.28108900	-5.02832000	-3.92528900
H	-5.39949300	-6.30859500	-2.73107700
C	-6.99746800	-2.92547800	-1.61905700
H	-7.07196500	-1.83425500	-1.68241200
H	-7.67016800	-3.22418900	-0.80383300
C	-7.48919000	-3.57189900	-2.91344600
H	-8.56409600	-3.39629400	-3.02314000
H	-6.99517600	-3.10378500	-3.77486200
C	-7.17221000	-5.06427900	-2.90226500
H	-7.62370900	-5.57194200	-3.76057800

H	-7.59767800	-5.51757400	-1.99682400
C	-6.83929600	-3.08935000	1.77257900
H	-6.74747200	-3.78327800	0.93535800
H	-6.08275100	-3.41140700	2.50388000
C	-8.25241200	-0.75572500	2.95729200
H	-8.17879800	-0.01389000	3.75990800
H	-9.18023000	-0.52750900	2.41405300
C	-8.21896400	-3.19678500	2.41744100
H	-8.99690900	-3.02043700	1.66173300
H	-8.36670500	-4.21122400	2.80200100
C	-8.35419700	-2.16564000	3.53449000
H	-7.55330800	-2.32517200	4.26824800
H	-9.30519600	-2.28175700	4.06446800

Zero-point correction= 1.466639 (Hartree/Particle)

Thermal correction to Energy= 1.531814

Thermal correction to Enthalpy= 1.532615

Thermal correction to Gibbs Free Energy= 1.370822

Sum of electronic and zero-point Energies= -15022.491021

Sum of electronic and thermal Energies= -15022.425847

Sum of electronic and thermal Enthalpies= -15022.425045

Sum of electronic and thermal Free Energies= -15022.586839

AP-8-TS1*R8*

C	3.50028900	-2.41520000	0.15467400
C	1.27712500	-1.53539000	-1.34091500
C	3.55479500	-2.24424300	-1.24456100
C	2.29617800	-2.15945100	0.80553600
C	1.14940400	-1.65367500	0.08601600

C	2.45610600	-1.74380700	-1.98420700
H	0.16054300	-1.92021900	0.45557600
H	2.52311700	-1.59479200	-3.05303900
H	0.39036700	-1.24807300	-1.88682300
C	4.69765200	-2.91436000	0.88773700
C	6.88330400	-3.98590800	2.28520300
C	5.91836600	-2.21126800	0.92044000
C	4.59556800	-4.13048200	1.56962800
C	5.67327100	-4.66972200	2.26142400
C	6.99140500	-2.76453700	1.62770000
H	3.64052000	-4.64569000	1.55910100
H	5.56615200	-5.61745800	2.78043300
H	7.93449400	-2.23135100	1.67696600
H	7.73678300	-4.39158900	2.81944900
O	2.25432500	-2.38983400	2.11738600
O	4.67429300	-2.65451300	-1.82919700
C	1.05189000	-2.12234200	2.84397100
H	0.21497000	-2.71259800	2.46028900
H	0.81052700	-1.05872800	2.79839400
H	1.26468600	-2.41952100	3.87030000
C	4.91925300	-2.24323000	-3.16936100
H	4.20616900	-2.70731200	-3.85832700
H	5.92328900	-2.59428900	-3.40684500
H	4.87417500	-1.15240600	-3.23468100
P	6.09409400	-0.49445500	0.27772500
O	5.16503300	-0.13583200	-0.85299700
H	-1.73927400	1.62520200	1.11674900
C	-0.39325800	3.23973600	1.34683500

N	0.75716200	2.61141500	1.15700400
C	1.83235400	3.49206000	1.30234500
Br	1.05415100	0.49820200	0.60941000
O	2.98411400	3.20218800	1.08545900
O	-1.55260900	2.75606500	1.24455900
C	1.31436200	4.84401000	1.78269400
H	1.70160100	5.63737700	1.14059400
H	1.69493700	5.01453900	2.79429900
C	-0.20379300	4.69232700	1.73460200
H	-0.68337000	5.33329000	0.98852300
H	-0.70742600	4.86187400	2.68977800
C	0.75704100	5.05177000	-1.59265700
C	0.38421300	2.32953900	-2.11994000
C	-0.53388500	4.55345000	-1.69179300
C	1.86517300	4.22353900	-1.71757700
C	1.67347700	2.86791500	-1.96094700
C	-0.70321800	3.20013400	-1.94924400
H	-1.39784500	5.19795200	-1.57299600
H	2.86820900	4.61236100	-1.58487900
O	0.24452900	1.04096100	-2.43767700
H	-0.58293100	0.64798400	-2.05687400
Br	3.15658800	1.71009000	-1.90600900
Br	-2.45441100	2.50488100	-2.01568600
Br	0.99794300	6.89989000	-1.23621800
C	5.88684500	0.50337100	1.79372300
C	5.99195000	2.79970400	2.79968400
C	4.55710000	0.98616100	3.85855100
C	4.76140500	2.48984300	3.65789700

C	4.56664800	0.22424700	2.52872000
C	5.99577600	2.00445900	1.48908800
H	6.90386500	2.55293000	3.36389500
H	5.35892200	0.58676300	4.49657700
H	3.88113700	2.90074000	3.15588100
H	3.72474500	0.54697400	1.89956500
H	5.14059500	2.28720600	0.86424900
H	6.71592800	0.20010800	2.45533400
H	6.03292800	3.87322100	2.58332100
H	3.61330700	0.80292800	4.38559500
H	4.84906500	2.99003100	4.62996600
H	4.44128700	-0.84805100	2.71752800
H	6.90654500	2.23140200	0.92011700
C	7.83300100	-0.24089900	-0.29220600
C	10.22949600	0.46773500	-0.02529200
C	9.55768300	-0.98062500	-1.98158900
C	10.66844000	-0.63949300	-0.98589700
C	8.26160100	-1.35664000	-1.25859700
C	8.94512800	0.08305300	0.71861800
H	10.05117400	1.39169000	-0.59243200
H	9.36913100	-0.11057900	-2.62521600
H	10.92060700	-1.53858600	-0.40588100
H	8.41271500	-2.29006100	-0.70013500
H	9.16310800	-0.78328600	1.35482600
H	7.68264300	0.66676400	-0.89885200
H	11.02502100	0.68504400	0.69600100
H	9.87194000	-1.80060700	-2.63702600
H	11.57774900	-0.34065400	-1.51887100

H	7.45916500	-1.54298700	-1.98146200
H	8.64181100	0.89744000	1.38531200
C	-5.59269600	0.86860500	0.92376700
H	-7.13798300	1.43004600	2.28964600
C	-6.65636900	0.59540400	1.78400600
C	-5.45770800	-1.52915800	0.47097800
C	-7.09069900	-0.70187600	2.05561500
C	-5.00834200	-0.22061000	0.27738300
C	-6.46513700	-1.78607900	1.41991400
C	-3.42386100	-2.80435700	-0.16131000
C	-4.80112700	-2.61955200	-0.30577100
C	-3.52138800	-4.72436200	-1.56485300
C	-5.52495300	-3.42590300	-1.20706600
C	-2.76152300	-3.88457000	-0.75085100
C	-4.87199900	-4.49661700	-1.83315800
H	-3.02967500	-5.57788600	-2.02759200
O	-3.95202900	-0.02235200	-0.59467600
O	-2.71752300	-1.90038300	0.61480600
P	-2.46331800	-0.42123900	-0.04888600
O	-1.56014000	-0.52708600	-1.22612500
O	-2.13143500	0.43599800	1.15840900
H	-0.18030900	-3.53679400	-4.92686000
C	0.10067700	-3.90636300	-3.94543100
H	-1.74037300	-3.32343300	-3.05088500
C	-0.77361000	-3.79291400	-2.90347900
C	1.77501700	-4.91294900	-2.52361200
C	-0.42811200	-4.26464300	-1.59286800
C	1.38563800	-4.49907400	-3.76226900

C	0.89450200	-4.79387000	-1.40054300
C	-1.32731900	-4.21183800	-0.50716300
H	2.05527300	-4.59854200	-4.61168400
H	2.29649200	-5.59771800	0.00950900
H	2.76373600	-5.33459400	-2.35771200
C	-0.90392900	-4.61126500	0.78119200
C	-1.77128500	-4.59088300	1.92320800
C	0.43761300	-5.09378300	0.97039800
H	1.87205100	-5.87504600	2.39790200
C	1.29706000	-5.18939000	-0.12623700
C	-1.33307800	-4.99266700	3.15216900
H	-2.79439300	-4.25222400	1.80240600
H	-2.00966000	-4.96489200	4.00085600
C	0.00233000	-5.45612100	3.33674800
H	0.32958400	-5.77369500	4.32204600
C	0.85665800	-5.50658900	2.27586900
H	-6.34861900	2.45209500	-3.68894200
C	-5.96160900	2.96760700	-2.81528500
H	-6.13047400	1.25633000	-1.56530300
C	-5.83802000	2.29919300	-1.63156300
C	-5.10610200	5.00060100	-1.83167700
C	-5.30855300	2.94716900	-0.46809200
C	-5.59119800	4.34054600	-2.92049000
C	-4.93971100	4.33122400	-0.57797300
C	-5.11713300	2.27073500	0.75245500
H	-5.69897600	4.85299000	-3.87148200
H	-4.14768800	6.04638500	0.44560800
H	-4.82183100	6.04817600	-1.89586600

C	-4.53039500	2.93629500	1.84734800
C	-4.21784000	2.26721900	3.07642200
C	-4.18917800	4.32667900	1.73293500
H	-3.37868700	6.05621500	2.75848800
C	-4.40939300	4.99296600	0.52896900
C	-3.64837200	2.93790900	4.11836600
H	-4.41712400	1.20442800	3.15751900
H	-3.40945200	2.40882100	5.03600200
C	-3.35228500	4.32949400	4.01585100
H	-2.91319400	4.84831100	4.86330300
C	-3.61109400	4.99813200	2.85660600
C	-5.58127300	-5.41000200	-2.81454200
H	-5.22111300	-5.18140700	-3.82702500
H	-5.29031600	-6.44741400	-2.61388800
C	-6.97113600	-3.09403200	-1.53299400
H	-7.07619700	-2.00635000	-1.61188100
H	-7.62699100	-3.39850600	-0.70629000
C	-7.45827800	-3.77343700	-2.81220500
H	-8.53877800	-3.62949200	-2.91249700
H	-6.98680700	-3.30478200	-3.68588200
C	-7.09930800	-5.25606100	-2.78252900
H	-7.54563700	-5.78903700	-3.62807500
H	-7.50189000	-5.70741000	-1.86573100
C	-6.77713300	-3.20655800	1.85687500
H	-6.67109500	-3.90971200	1.02914600
H	-6.00716800	-3.49651800	2.58757800
C	-8.24503300	-0.89697000	3.02115800
H	-8.18584400	-0.14293000	3.81352200

H -9.18246000 -0.70099800 2.48186300

C -8.14897900 -3.34314100 2.51278600

H -8.93649900 -3.19805600 1.76035200

H -8.26631600 -4.35608300 2.91148500

C -8.30482500 -2.30141300 3.61726400

H -7.49534600 -2.42969800 4.34770900

H -9.24897700 -2.43604400 4.15502900

Zero-point correction= 1.465587 (Hartree/Particle)

Thermal correction to Energy= 1.530995

Thermal correction to Enthalpy= 1.531797

Thermal correction to Gibbs Free Energy= 1.369276

Sum of electronic and zero-point Energies= -15022.490006

Sum of electronic and thermal Energies= -15022.424597

Sum of electronic and thermal Enthalpies= -15022.423796

Sum of electronic and thermal Free Energies= -15022.586317

AP-8-TS1^{R9}

C 3.39876000 -2.58761500 0.13777700

C 1.20792800 -1.60447700 -1.34337000

C 3.46047800 -2.38898800 -1.25820400

C 2.20534600 -2.29536800 0.79388100

C 1.07960400 -1.73335400 0.08288900

C 2.37817300 -1.84557400 -1.99076000

H 0.08120600 -1.96251300 0.45192400

H 2.45007600 -1.69496100 -3.05948800

H 0.32996200 -1.28266200 -1.88376700

C 4.57543200 -3.16239900 0.84807100

C 6.74848200 -4.41386500 2.10682500

C	5.83842100	-2.53682600	0.84716900
C	4.42257300	-4.38480700	1.50779000
C	5.49320300	-5.00949900	2.13722100
C	6.90899100	-3.18538200	1.47229100
H	3.43753000	-4.83989700	1.52603100
H	5.34698000	-5.95981800	2.64175500
H	7.89383700	-2.72962300	1.47258300
H	7.59908600	-4.89443500	2.58012000
O	2.15525900	-2.54279500	2.10183300
O	4.56963800	-2.81287000	-1.85229500
C	0.96312600	-2.24013800	2.83217900
H	0.10668800	-2.79971800	2.44541200
H	0.75762300	-1.16865100	2.79400300
H	1.16721000	-2.55114200	3.85616300
C	4.86521000	-2.31593300	-3.15391800
H	4.22060900	-2.78455500	-3.90485700
H	5.90258600	-2.59298800	-3.34414400
H	4.75991100	-1.22827800	-3.16967400
P	6.09480300	-0.80980400	0.25595000
O	5.18681200	-0.35611400	-0.85647300
H	-1.67287400	1.65093600	1.14048800
C	-0.26953300	3.20332300	1.39118100
N	0.85700900	2.53439300	1.20085100
C	1.96331300	3.37533100	1.35669200
Br	1.07378100	0.41516900	0.63419300
O	3.10420800	3.04625200	1.14070700
O	-1.44670500	2.76473000	1.28069300
C	1.49252400	4.74060600	1.84740700

H	1.91147500	5.52517900	1.21460300
H	1.87449500	4.88740400	2.86217700
C	-0.02976000	4.64466700	1.79139600
H	-0.48259000	5.30818900	1.04830700
H	-0.53170000	4.82478500	2.74555800
C	0.97933800	4.99810800	-1.53133600
C	0.49594800	2.29781100	-2.08093400
C	-0.33076600	4.55411500	-1.63808400
C	2.05265500	4.12547300	-1.65841700
C	1.80585600	2.78077300	-1.91280800
C	-0.55537500	3.21117400	-1.90664500
H	-1.16784700	5.23276300	-1.51717400
H	3.07045200	4.47129500	-1.51924000
O	0.30441800	1.01903900	-2.40999000
H	-0.54259400	0.65862700	-2.03998900
Br	3.24065900	1.56195800	-1.86163200
Br	-2.33428100	2.59061200	-1.98416700
Br	1.29366400	6.83264500	-1.16178300
C	5.91062300	0.16680600	1.79229900
C	5.98898500	2.46411100	2.80579200
C	4.60831000	0.61815200	3.88096800
C	4.78373500	2.12586100	3.68921100
C	4.60090400	-0.12674800	2.54199100
C	5.99169500	1.67153800	1.49332100
H	6.91699000	2.24033800	3.35303500
H	5.42928400	0.22359500	4.49720300
H	3.88452100	2.52427700	3.21174400
H	3.75052900	0.20741500	1.92901600

H	5.12872300	1.94238600	0.87453900
H	6.75021900	-0.12918700	2.44177500
H	5.99826600	3.53816000	2.58864700
H	3.67853100	0.41467400	4.42527200
H	4.88499000	2.62058000	4.66278900
H	4.47705300	-1.20057000	2.71894100
H	6.89312500	1.91545400	0.91752000
C	7.87580400	-0.73360100	-0.26991200
C	9.41921200	0.15456800	-2.06349400
C	10.32019800	-0.34620700	0.23602600
C	10.44791800	0.54399200	-1.00090800
C	8.89683100	-0.31023400	0.80209800
C	7.99598000	0.18196900	-1.50211200
H	9.64223500	-0.85845200	-2.42763300
H	10.57367700	-1.38144300	-0.03290000
H	10.28354400	1.59021200	-0.70779300
H	8.67150500	0.71230000	1.13239200
H	7.73257100	1.20976500	-1.21732000
H	8.12167800	-1.75639300	-0.59525200
H	9.49010300	0.82513900	-2.92707000
H	11.03286100	-0.03423400	1.00756200
H	11.46264400	0.48357400	-1.40961300
H	8.83668800	-0.94215500	1.69482100
H	7.26974000	-0.12062000	-2.26125800
C	-5.55609600	1.03965900	0.92366800
H	-7.08290200	1.64565000	2.29146200
C	-6.63279000	0.79872000	1.77730600
C	-5.51391900	-1.35618300	0.44323400

C	-7.11865900	-0.48361500	2.03236400
C	-5.01282700	-0.06422600	0.26663400
C	-6.53412900	-1.58427300	1.38591300
C	-3.52946100	-2.70391600	-0.19741400
C	-4.89783200	-2.46253300	-0.34405700
C	-3.70017200	-4.60522200	-1.61987100
C	-5.65026300	-3.22949400	-1.25622200
C	-2.90969100	-3.80479100	-0.79538000
C	-5.03924000	-4.31981600	-1.89073300
H	-3.24186900	-5.47367500	-2.08913700
O	-3.94633300	0.10239100	-0.59900800
O	-2.78973600	-1.83774200	0.59016900
P	-2.47644200	-0.35989200	-0.05227800
O	-1.57493300	-0.48439600	-1.22906500
O	-2.11597900	0.46818700	1.16590000
H	-0.30026800	-3.51976300	-4.95939700
C	-0.03725000	-3.90840300	-3.98034900
H	-1.86166900	-3.27091000	-3.08949700
C	-0.91230300	-3.77428600	-2.94147900
C	1.59257400	-4.98863000	-2.56136500
C	-0.59008600	-4.26965200	-1.63373100
C	1.22508700	-4.54771900	-3.79725800
C	0.71118300	-4.84940000	-1.44143400
C	-1.49072400	-4.19237200	-0.55057800
H	1.89578200	-4.66098000	-4.64403400
H	2.07561600	-5.72039600	-0.03517000
H	2.56472300	-5.44691000	-2.39493500
C	-1.08803900	-4.62045500	0.73522700

C	-1.95827200	-4.57793600	1.87452000
C	0.23299100	-5.15671000	0.92418300
H	1.63017300	-6.00892800	2.34771400
C	1.09292100	-5.27319600	-0.16993000
C	-1.54108000	-5.01021500	3.10047900
H	-2.96698600	-4.19834300	1.75381300
H	-2.21948500	-4.96520000	3.94695800
C	-0.22566200	-5.52792900	3.28473400
H	0.08493900	-5.86887700	4.26760400
C	0.63035100	-5.59983200	2.22645800
H	-6.21906400	2.70901400	-3.67449000
C	-5.81727400	3.19774800	-2.79215200
H	-6.06022500	1.47843700	-1.56527700
C	-5.72715500	2.50992600	-1.61649200
C	-4.88792800	5.18260600	-1.77783300
C	-5.17982400	3.12175900	-0.44181500
C	-5.39271400	4.55630900	-2.87757500
C	-4.75565200	4.49127700	-0.53202300
C	-5.02331600	2.42324500	0.77134800
H	-5.47479000	5.08463500	-3.82241900
H	-3.90294300	6.16083400	0.51777700
H	-4.56189700	6.21875400	-1.82703400
C	-4.41605300	3.05091500	1.87736600
C	-4.13462800	2.35359700	3.09829200
C	-4.01993500	4.42808300	1.78284300
H	-3.14855000	6.11123900	2.83521100
C	-4.20642100	5.11759400	0.58624200
C	-3.54404100	2.98759200	4.15139900

H	-4.37389400	1.29797300	3.16362100
H	-3.32900900	2.43716900	5.06235700
C	-3.19458100	4.36808000	4.06920000
H	-2.73983500	4.85824800	4.92541900
C	-3.42202500	5.06168600	2.91803500
C	-5.78150300	-5.19366400	-2.88375300
H	-5.40763900	-4.97070100	-3.89251400
H	-5.53485400	-6.24414500	-2.69189600
C	-7.08029000	-2.83532100	-1.58402500
H	-7.14027500	-1.74347900	-1.65183800
H	-7.75165700	-3.12115200	-0.76313200
C	-7.58918200	-3.48070700	-2.87226000
H	-8.66233800	-3.29116600	-2.97554300
H	-7.09478300	-3.02296300	-3.73901400
C	-7.29209000	-4.97713500	-2.85654700
H	-7.75617400	-5.48251600	-3.70945600
H	-7.71727300	-5.42070600	-1.94617300
C	-6.90303900	-2.99645900	1.80517800
H	-6.82410300	-3.69318600	0.96899700
H	-6.14597500	-3.32571900	2.53271200
C	-8.28233900	-0.64435200	2.99295300
H	-8.19644800	0.09846300	3.79342300
H	-9.21014400	-0.40699100	2.45364200
C	-8.28004200	-3.08671000	2.45838100
H	-9.06043700	-2.90323000	1.70691700
H	-8.43692400	-4.09858000	2.84605900
C	-8.39701100	-2.05171700	3.57389600
H	-7.59382600	-2.21881300	4.30344500

H -9.34622900 -2.15576100 4.10953200

Zero-point correction= 1.466105 (Hartree/Particle)

Thermal correction to Energy= 1.531363

Thermal correction to Enthalpy= 1.532165

Thermal correction to Gibbs Free Energy= 1.370135

Sum of electronic and zero-point Energies= -15022.489654

Sum of electronic and thermal Energies= -15022.424395

Sum of electronic and thermal Enthalpies= -15022.423593

Sum of electronic and thermal Free Energies= -15022.585624

AP-8-TS1^{HB}

C	2.90496300	-3.17491900	-0.21541700
C	0.77645000	-1.95127000	-1.61775400
C	3.01358900	-2.78911900	-1.56625800
C	1.72546400	-2.89297900	0.45887100
C	0.65186800	-2.16450400	-0.19330600
C	1.93250400	-2.21151200	-2.28032800
H	-0.36591700	-2.31878600	0.15966500
H	2.02410400	-1.96700400	-3.32953200
H	-0.08318500	-1.51072600	-2.10814200
C	4.07489700	-3.78962200	0.47220000
C	6.24602800	-5.09402000	1.66778000
C	5.22997500	-3.05444000	0.80163700
C	4.02272000	-5.15543400	0.76155600
C	5.09452300	-5.80905600	1.35801300
C	6.30523000	-3.73142800	1.39288700
H	3.12317100	-5.70654800	0.50216500
H	5.03260600	-6.87154900	1.57239900

H	7.20759900	-3.19086100	1.66121200
H	7.09634600	-5.59010800	2.12516100
O	1.65328400	-3.24843800	1.73745900
O	4.18898200	-3.02374800	-2.13647700
C	0.56659300	-2.78781900	2.55279200
H	-0.39907500	-3.11970200	2.16273000
H	0.58339900	-1.69897800	2.62017100
H	0.73648100	-3.23536100	3.53104500
C	4.45491400	-2.43178100	-3.40661300
H	3.83415800	-2.88347200	-4.18611000
H	5.50272100	-2.64126400	-3.61797300
H	4.28788800	-1.35119100	-3.36118900
P	5.32865900	-1.22430600	0.60727500
O	4.29609700	-0.66537100	-0.34404800
H	-1.30521800	1.50768900	1.15972600
C	0.31864600	2.78670900	1.48760000
N	1.32535800	1.95838000	1.29175300
C	2.53944700	2.60411500	1.54085600
Br	1.04650700	-0.17441000	0.48373300
O	3.62953700	2.10147200	1.38797100
O	-0.91401000	2.54230800	1.33065000
C	2.26740900	4.02640900	2.04298600
H	2.80307300	4.74765700	1.42087500
H	2.65256300	4.11298400	3.06298200
C	0.75003500	4.15616200	1.95941500
H	0.41107700	4.90878300	1.24063100
H	0.25836500	4.37028500	2.91172300
C	1.81885600	4.72180800	-1.30314900

C	2.39548800	2.03313900	-1.84558400
C	3.14150300	4.30108700	-1.29492400
C	0.78536700	3.85312400	-1.61740100
C	1.08053000	2.52389600	-1.89588500
C	3.40535200	2.96722900	-1.57444100
H	3.94937400	4.98707600	-1.06839100
H	-0.24650900	4.18637600	-1.61987100
O	2.65540200	0.74013400	-2.09330600
H	3.28984600	0.36812700	-1.42743300
Br	-0.35110500	1.35950500	-2.23069700
Br	5.18790000	2.35736800	-1.56866800
Br	1.40395800	6.51451500	-0.84407800
C	5.17715600	-0.55538400	2.30401800
C	3.59344200	-0.03568200	4.18348900
C	6.06185200	-0.31513000	4.64320500
C	4.64639800	-0.47076800	5.20509600
C	6.21169600	-1.06923600	3.31719200
C	3.76140900	-0.77145700	2.85205400
H	3.68943100	1.04276500	3.99572700
H	6.27526000	0.74979600	4.47674100
H	4.48133600	-1.52622600	5.46356900
H	6.03395400	-2.13738700	3.49848700
H	3.58719000	-1.84917100	2.99488600
H	5.30803100	0.52675700	2.15500800
H	2.58482700	-0.20365700	4.57907700
H	6.80049600	-0.68097800	5.36535100
H	4.54201500	0.10354400	6.13260100
H	7.23844200	-0.97554500	2.94266900

H	3.03040900	-0.41293500	2.12623300
C	7.04418300	-0.87030100	0.02753700
C	8.39524400	-0.65649300	-2.11870700
C	8.86350000	0.88699600	-0.19496400
C	8.79362200	0.76357300	-1.71692300
C	7.52772600	0.52591800	0.46168200
C	7.04678700	-1.03163900	-1.50302400
H	9.16845700	-1.35902400	-1.77759800
H	9.64678100	0.21421500	0.18220100
H	8.04635900	1.47208800	-2.10465400
H	6.77649400	1.27692000	0.19571300
H	6.26352400	-0.38543400	-1.91948100
H	7.72729100	-1.61505300	0.45600800
H	8.34037400	-0.74746700	-3.20949400
H	9.15006800	1.90333800	0.09680900
H	9.75383100	1.03718700	-2.16749300
H	7.63201000	0.57257900	1.55061200
H	6.77459200	-2.06449200	-1.75399200
C	-5.18298600	2.08045500	0.65991600
H	-6.55252600	3.32119000	1.73854500
C	-6.34287500	2.30739800	1.40281300
C	-5.85735700	-0.27204400	0.49932600
C	-7.21098500	1.28139100	1.77483200
C	-4.96646100	0.77233800	0.21462700
C	-6.94708900	-0.03302500	1.35691400
C	-4.36261000	-2.21124900	0.05899300
C	-5.60539100	-1.60137400	-0.13660000
C	-4.99426400	-4.02179300	-1.34868400

C	-6.53401400	-2.18520100	-1.02473000
C	-4.02051600	-3.41996800	-0.55368500
C	-6.23150700	-3.42740900	-1.60244600
H	-4.75996700	-4.97108300	-1.82712900
O	-3.86816300	0.50213400	-0.56782500
O	-3.44038900	-1.55858600	0.85074800
P	-2.62836800	-0.36304700	0.06541700
O	-1.87081200	-0.91951500	-1.07921700
O	-1.99605700	0.40802100	1.20385200
H	-1.74637600	-3.25481000	-4.89433500
C	-1.46884000	-3.74618200	-3.96676600
H	-3.09312100	-2.90730100	-2.87767200
C	-2.22834100	-3.55993000	-2.84834000
C	0.09184400	-5.13950700	-2.76006900
C	-1.88087700	-4.18533200	-1.60497900
C	-0.30314800	-4.56769900	-3.93171400
C	-0.66693700	-4.95372500	-1.55998800
C	-2.67135000	-4.05085500	-0.44137000
H	0.27792300	-4.71295700	-4.83770600
H	0.66637400	-6.09725300	-0.32116700
H	0.99662000	-5.74034900	-2.70633400
C	-2.22333200	-4.59860100	0.77900400
C	-2.96319400	-4.46561900	2.00005400
C	-0.98738000	-5.33463500	0.82039000
H	0.39234800	-6.44320600	2.07327700
C	-0.24735100	-5.50647800	-0.34945100
C	-2.50485900	-5.00780300	3.16547100
H	-3.89936000	-3.91787200	1.98705100

H	-3.07908800	-4.88771600	4.07900800
C	-1.27900000	-5.73726000	3.20066600
H	-0.93557000	-6.16284200	4.13872900
C	-0.54642000	-5.89482500	2.06260600
H	-4.51900900	2.82551700	-4.18941200
C	-4.08024200	3.32191100	-3.32937600
H	-5.18586100	2.16468100	-1.93589000
C	-4.45021100	2.94975700	-2.07040500
C	-2.58796100	5.00940100	-2.46316300
C	-3.88205000	3.57397400	-0.91211300
C	-3.12227500	4.35817800	-3.53378300
C	-2.95216200	4.64870600	-1.12546600
C	-4.21242100	3.18493800	0.40504600
H	-2.83473300	4.63230800	-4.54417500
H	-1.72157200	6.14892000	-0.20093300
H	-1.87015900	5.81605000	-2.59876200
C	-3.64372300	3.86858100	1.50372400
C	-3.86041000	3.47159300	2.86528000
C	-2.76861400	4.98482200	1.27273300
H	-1.59960000	6.56330000	2.18641200
C	-2.42494200	5.33459000	-0.03134500
C	-3.31771200	4.17031700	3.90381000
H	-4.45022400	2.58453700	3.06558700
H	-3.49311600	3.84245800	4.92402800
C	-2.51032500	5.32155300	3.66775800
H	-2.10347300	5.87570500	4.50864600
C	-2.23626200	5.70459900	2.38905600
C	-7.18103400	-4.12974600	-2.55579900

H	-6.75096700	-4.09303100	-3.56612100
H	-7.23779700	-5.19098600	-2.28734900
C	-7.80010700	-1.44020100	-1.41317100
H	-7.56415800	-0.37677300	-1.52915700
H	-8.54037700	-1.49634400	-0.60405600
C	-8.44226800	-1.98845700	-2.68664800
H	-9.41817000	-1.51525200	-2.83534000
H	-7.82300800	-1.73452700	-3.55696200
C	-8.57281600	-3.50499400	-2.58687400
H	-9.14515800	-3.91172600	-3.42675300
H	-9.11892800	-3.76026000	-1.66884000
C	-7.75237700	-1.18589800	1.92818600
H	-7.81040100	-2.01696800	1.22337100
H	-7.19060000	-1.57355300	2.79088900
C	-8.43685400	1.62685000	2.60097600
H	-8.18815600	2.43077800	3.30246600
H	-9.20213500	2.03631400	1.92655800
C	-9.14147400	-0.76662300	2.40227200
H	-9.76397100	-0.49425500	1.53856300
H	-9.63129600	-1.60945900	2.90096600
C	-9.02651700	0.42974400	3.34363800
H	-8.37706100	0.15930200	4.18641000
H	-10.00254600	0.69728000	3.76165000

Zero-point correction= 1.466275 (Hartree/Particle)

Thermal correction to Energy= 1.531787

Thermal correction to Enthalpy= 1.532589

Thermal correction to Gibbs Free Energy= 1.368528

Sum of electronic and zero-point Energies= -15022.484969

Sum of electronic and thermal Energies= -15022.419456

Sum of electronic and thermal Enthalpies= -15022.418655

Sum of electronic and thermal Free Energies= -15022.582716

AP-8-TS1[S1]

H	2.59510000	-1.34582900	1.19954000
C	1.92070000	-3.18478400	1.79540400
N	0.63243500	-3.02112200	1.57702200
C	-0.09601700	-4.13592300	2.01165800
Br	-0.44645000	-1.29347200	0.72181500
O	-1.29188300	-4.25275800	1.89827200
O	2.85057200	-2.36935700	1.51223200
C	0.86524000	-5.14458900	2.63741200
H	0.76648500	-6.10517300	2.12640100
H	0.58553300	-5.29163000	3.68388100
C	2.24158000	-4.50341900	2.46311900
H	2.91871500	-5.08336900	1.82861200
H	2.76519400	-4.29766500	3.40067200
C	1.56832600	-5.53455000	-0.73828100
C	0.81385100	-3.02181500	-1.73618300
C	2.54585800	-4.58655700	-1.00463600
C	0.22165000	-5.25639400	-0.93286500
C	-0.13866100	-4.00427800	-1.41356600
C	2.15981700	-3.34634200	-1.49493900
H	3.59557000	-4.79878600	-0.83432000
H	-0.54098200	-5.98779000	-0.69167600
O	0.41714700	-1.86561800	-2.26549400
H	0.98228100	-1.09922000	-1.96920600

Br	-1.97310600	-3.58927600	-1.54595200
Br	3.49044600	-2.04797400	-1.80026900
Br	2.09109600	-7.23361700	-0.07866300
C	5.68181600	1.10029600	0.71061300
H	7.32203600	1.33297700	2.06178900
C	6.51824700	1.84814800	1.53945000
C	4.49091800	3.17032100	0.19128900
C	6.33126700	3.21214200	1.76401200
C	4.67329200	1.79417000	0.04200800
C	5.28317600	3.88171900	1.11255000
C	2.09018400	3.39479100	-0.42471300
C	3.40898700	3.82841400	-0.59668300
C	1.32564000	5.16683700	-1.82413900
C	3.69744100	4.85332400	-1.52049200
C	1.01569600	4.09094700	-0.99286900
C	2.63640000	5.53286000	-2.13299700
H	0.50456200	5.72734900	-2.26714300
O	3.80662300	1.12035100	-0.79763300
O	1.85705900	2.28990100	0.37585800
P	2.31770300	0.81325600	-0.19062400
O	1.45477300	0.41262700	-1.33761400
O	2.46269300	-0.02123900	1.06013400
H	-1.33132500	2.62723200	-5.07815000
C	-1.70359500	2.81469400	-4.07516600
H	0.23176900	3.23536700	-3.29836600
C	-0.82973700	3.16338200	-3.08605700
C	-3.57469300	2.86333200	-2.54554000
C	-1.28859000	3.41858400	-1.74997300

C	-3.09853400	2.67471700	-3.80857600
C	-2.69159100	3.24539100	-1.48444200
C	-0.41780500	3.79200500	-0.70364600
H	-3.77067200	2.39180500	-4.61378600
H	-4.24491900	3.28866000	-0.01081200
H	-4.62005300	2.70434900	-2.29447000
C	-0.93224700	4.01558300	0.59450300
C	-0.10746500	4.42175700	1.69553100
C	-2.34111900	3.85885400	0.83929900
H	-3.93203400	4.03910800	2.29903200
C	-3.18532500	3.45379600	-0.19651700
C	-0.64064000	4.66309600	2.92930100
H	0.95816600	4.54821100	1.53708200
H	0.00714600	4.97131600	3.74439900
C	-2.04124700	4.53041000	3.16007500
H	-2.44715200	4.74354000	4.14452400
C	-2.86091500	4.13915300	2.14402300
H	6.96555400	-0.22586100	-3.87312200
C	6.87222600	-0.81117600	-2.96348200
H	6.27251900	0.85812500	-1.79172200
C	6.48344600	-0.20605700	-1.80350600
C	7.04876000	-2.95397200	-1.86311500
C	6.32652400	-0.95779200	-0.59387000
C	7.16235900	-2.20694900	-2.99693300
C	6.61871300	-2.36390800	-0.63293100
C	5.86858400	-0.37482900	0.60457700
H	7.47190300	-2.66742300	-3.93010700
H	6.68091800	-4.19836800	0.48341700

H	7.26456500	-4.01949200	-1.87363000
C	5.64582200	-1.17679900	1.74215600
C	5.08393100	-0.65388600	2.95379500
C	5.95285200	-2.57930700	1.69391500
H	5.99322600	-4.43780800	2.80985600
C	6.44478900	-3.13617400	0.51498600
C	4.88267200	-1.45401600	4.03937700
H	4.79189200	0.38979800	2.98541000
H	4.44766000	-1.03876900	4.94355900
C	5.22754800	-2.83769600	3.99990200
H	5.07444000	-3.45406400	4.88156000
C	5.73775100	-3.38172700	2.85925400
C	2.86442400	6.65075500	-3.13196600
H	2.62651800	6.27677500	-4.13736100
H	2.15453400	7.46138300	-2.93179200
C	5.13986300	5.17295100	-1.87588700
H	5.70005300	4.23521100	-1.96132800
H	5.61764000	5.73267300	-1.06062500
C	5.26430800	5.98956900	-3.16178000
H	6.29892200	6.32707900	-3.28032800
H	5.02862800	5.35833700	-4.02856400
C	4.29915300	7.17061800	-3.12435400
H	4.45830000	7.83983600	-3.97593100
H	4.47950900	7.75659300	-2.21299300
C	4.92922400	5.30487600	1.50781400
H	4.51557400	5.86162400	0.66542400
H	4.11472200	5.24193000	2.24506900
C	7.28287800	3.93328000	2.70065300

H	7.57474600	3.25736900	3.51179300
H	8.20437600	4.16294700	2.14731000
C	6.09798600	6.06052500	2.13523200
H	6.86329200	6.26072100	1.37253600
H	5.75117200	7.03038800	2.50667800
C	6.70996200	5.23251800	3.26184300
H	5.93237300	5.00587400	4.00297500
H	7.49612500	5.79178200	3.77954400
C	-3.71112100	-0.51529900	0.06040700
C	-1.48369200	0.30778200	-1.45200000
C	-3.62852800	-0.73319000	-1.31576400
C	-2.69587300	0.20806600	0.68419900
C	-1.47274000	0.49813800	-0.02307200
C	-2.53119500	-0.27078300	-2.09099700
H	-0.81174900	1.27710100	0.35002300
H	-2.49128200	-0.43998700	-3.15871600
H	-0.59794400	0.61315700	-1.99264900
C	-4.73181300	-1.22262600	0.88327300
C	-6.43557000	-2.71675800	2.52180300
C	-6.06943700	-0.82974300	1.02925700
C	-4.26280000	-2.36320400	1.54865300
C	-5.10986600	-3.11148100	2.35561700
C	-6.90339700	-1.58037200	1.87206300
H	-3.21543000	-2.64215200	1.43847500
H	-4.72900800	-3.99352000	2.86148900
H	-7.92913500	-1.26608600	2.04214700
H	-7.10182000	-3.28423500	3.16440700
O	-2.86763400	0.48545500	1.97206100

O	-4.61744300	-1.46123800	-1.83896500
C	-1.78813100	1.00890700	2.75193700
H	-1.32565600	1.87819400	2.27972000
H	-1.04079200	0.23026100	2.91427900
H	-2.23765800	1.30832800	3.69808000
C	-4.63591800	-1.68942400	-3.24371300
H	-4.67382300	-0.74332600	-3.79280000
H	-5.54249900	-2.26344200	-3.43601900
H	-3.76203800	-2.27112200	-3.55402200
P	-6.71650900	0.70277900	0.26855900
O	-5.70136900	1.47813800	-0.53338100
C	-8.16219600	0.28619800	-0.78350800
C	-8.85972500	-0.73635400	-2.98418400
C	-10.53057900	-0.49808700	-1.10650500
C	-10.09684700	-1.33558000	-2.31142700
C	-9.38194300	-0.34784300	-0.10336600
C	-7.71064100	-0.55318700	-1.98889400
H	-9.11975100	0.24155400	-3.41248900
H	-10.84563300	0.49788200	-1.44731100
H	-9.86461300	-2.35479300	-1.97262000
H	-9.11325000	-1.34511700	0.26671900
H	-7.36602700	-1.53353600	-1.62930500
H	-8.45776100	1.27833300	-1.16161100
H	-8.53506700	-1.37062900	-3.81723400
H	-11.39603700	-0.95627200	-0.61530400
H	-10.91810300	-1.41899400	-3.03165800
H	-9.70592400	0.24739700	0.75973800
H	-6.85390000	-0.06884700	-2.46932500

C -7.32013400 1.66196600 1.71313000

C -6.47369400 2.89400700 3.77309300

C -8.36659700 3.85926400 2.42700100

C -7.13404200 4.17926700 3.27308400

C -8.00730200 2.95437400 1.24515500

C -6.11041100 1.96305600 2.61504800

H -7.16463400 2.37524400 4.45221400

H -9.11450000 3.35487200 3.05485500

H -6.41642600 4.74420000 2.66091600

H -7.32526400 3.48981900 0.57029500

H -5.32975500 2.42606700 1.99655800

H -8.04269100 1.04979700 2.27201300

H -5.57298400 3.12439300 4.35477200

H -8.82964200 4.78127700 2.05868900

H -7.40726400 4.82174600 4.11734500

H -8.90940500 2.71793900 0.66732700

H -5.68480000 1.02869500 2.99722900

Zero-point correction= 1.466237 (Hartree/Particle)

Thermal correction to Energy= 1.531777

Thermal correction to Enthalpy= 1.532579

Thermal correction to Gibbs Free Energy= 1.368761

Sum of electronic and zero-point Energies= -15022.491206

Sum of electronic and thermal Energies= -15022.425666

Sum of electronic and thermal Enthalpies= -15022.424864

Sum of electronic and thermal Free Energies= -15022.588682

AP-8-TS1[S2]

C -3.71661800 -2.26293600 0.08110900

C	-1.16709100	-1.64364900	-0.94578900
C	-2.62560200	-2.20433900	0.94460200
C	-3.50267500	-1.98705700	-1.28464600
C	-2.22767500	-1.64604400	-1.79659000
C	-1.32407200	-1.80770200	0.47064400
H	-2.08042400	-1.45030200	-2.84969600
H	-0.44504100	-2.10023000	1.04172500
H	-0.16767500	-1.46962700	-1.32028300
C	-5.08392400	-2.62337000	0.55170300
C	-7.63472400	-3.47632200	1.34631300
C	-6.17241200	-1.73043700	0.47766200
C	-5.29106900	-3.91241000	1.04947300
C	-6.55167400	-4.34131600	1.44948000
C	-7.43823300	-2.18268700	0.87055900
H	-4.43913400	-4.58262900	1.11727400
H	-6.68680400	-5.34875400	1.83117100
H	-8.29437100	-1.51701600	0.81453100
H	-8.62858800	-3.79964400	1.63997200
O	-4.56461000	-2.12413600	-2.07362800
O	-2.83333500	-2.48479800	2.22982300
C	-4.50653700	-1.54035600	-3.36947900
H	-3.80528000	-2.08158700	-4.01312000
H	-4.22386800	-0.48616700	-3.28876000
H	-5.51181500	-1.63173300	-3.78017200
C	-1.75643000	-2.28919700	3.15579500
H	-0.89609300	-2.91721500	2.90949700
H	-2.15767600	-2.57919100	4.12646800
H	-1.45105300	-1.24084800	3.16239000

P	-5.95761900	0.05182800	0.06271000
O	-4.78114600	0.36287400	-0.82631400
H	1.32776900	1.71706700	0.92145300
C	-0.04386200	3.20274000	1.38465300
N	-1.16627600	2.51030300	1.35347100
C	-2.27501600	3.35826100	1.48898300
Br	-1.29897600	0.35499500	0.96094200
O	-3.42094800	2.98715700	1.51194900
O	1.13262400	2.75899000	1.24596300
C	-1.78454500	4.80195800	1.61080500
H	-2.20745900	5.24937500	2.51278500
H	-2.15357700	5.37058000	0.75193300
C	-0.26265100	4.67741000	1.62777800
H	0.19382200	4.94252100	2.58621300
H	0.24385200	5.25485000	0.84951400
C	-7.52178700	0.58391500	-0.73676400
C	-8.73766800	2.59156300	-1.68055500
C	-8.92883000	0.35377500	-2.82757400
C	-8.90105700	1.87122400	-3.02006900
C	-7.67932200	-0.12973400	-2.08777300
C	-7.49068900	2.10950700	-0.93410900
H	-9.62413800	2.40307700	-1.05843900
H	-9.82193400	0.07699800	-2.24980100
H	-8.05799700	2.13424200	-3.67377700
H	-6.78813400	0.09076700	-2.69023400
H	-6.58937400	2.37048100	-1.50474900
H	-8.37724100	0.33535500	-0.09514000
H	-8.68225800	3.67494200	-1.83404000

H	-9.00867500	-0.15276200	-3.79609900
H	-9.81339500	2.20845000	-3.52450900
H	-7.70913500	-1.21690100	-1.94362700
H	-7.41313200	2.62071400	0.03294700
C	-5.87052400	0.87583500	1.70036300
C	-7.01047100	1.59092500	3.83575200
C	-4.56049000	1.01465500	3.85833600
C	-5.86510100	0.99511000	4.65622700
C	-4.71447900	0.28609300	2.52201200
C	-7.18083700	0.85681900	2.50111500
H	-6.79866100	2.65034600	3.63569600
H	-4.26702600	2.05318000	3.66125800
H	-6.11487600	-0.04245400	4.92004200
H	-4.90647600	-0.77934900	2.71373600
H	-7.46810500	-0.18427800	2.70497800
H	-5.60367800	1.91383300	1.45535200
H	-7.94880000	1.55322000	4.40100800
H	-3.75003200	0.55398800	4.43606000
H	-5.74903000	1.54416600	5.59773100
H	-3.78926800	0.36287000	1.94562100
H	-7.99845700	1.31424500	1.93069300
C	5.19884000	0.99131200	0.51184400
H	6.50158600	2.42435600	-0.39310500
C	5.99313800	1.47124700	-0.52536100
C	4.78498900	-1.00856500	-0.84171700
C	6.10438300	0.80807500	-1.75198400
C	4.56924600	-0.24192500	0.30337700
C	5.48960700	-0.44077900	-1.92278400

C	2.94730300	-2.69435600	-0.61039600
C	4.29978300	-2.42179100	-0.83802000
C	3.37096000	-5.03542000	-0.72879500
C	5.21849400	-3.49122000	-0.92741800
C	2.45564300	-4.00077700	-0.55630400
C	4.73749700	-4.80836500	-0.89856700
H	3.00523900	-6.06005000	-0.69982200
O	3.72477900	-0.74320700	1.27427700
O	2.07313700	-1.63538100	-0.46073500
P	2.12995500	-0.72122800	0.91271300
O	1.38277700	-1.33059800	2.03138500
O	1.78579700	0.64497500	0.32293000
H	1.67708900	-4.94506700	4.20400500
C	1.01558700	-5.02751600	3.34733800
H	2.44157800	-4.20725900	1.99573700
C	1.44596700	-4.62068700	2.11755900
C	-1.15755200	-5.62080600	2.46888000
C	0.59027000	-4.70947300	0.97094700
C	-0.30029100	-5.54899500	3.52647100
C	-0.74817100	-5.19889100	1.16347300
C	1.01809600	-4.33808500	-0.32085400
H	-0.61811500	-5.87203200	4.51325700
H	-2.62357700	-5.65801300	0.21637200
H	-2.16957100	-5.99807100	2.59388400
C	0.12690100	-4.40520000	-1.40852500
C	0.50359600	-4.00836500	-2.73413500
C	-1.21532800	-4.87777500	-1.20324400
H	-3.12387500	-5.30421300	-2.14035200

C	-1.61730600	-5.26930900	0.07326600
C	-0.38234500	-4.08329500	-3.76938700
H	1.51136300	-3.64088500	-2.90255300
H	-0.07733700	-3.77492400	-4.76486500
C	-1.70929100	-4.56715000	-3.56265000
H	-2.39402300	-4.62832000	-4.40352400
C	-2.11178700	-4.94738600	-2.31688900
H	7.10550500	-1.69208400	4.15119500
C	6.64851700	-0.70727800	4.16529300
H	6.27333200	-0.71645600	2.07934900
C	6.17898300	-0.16247100	3.00667100
C	5.99271800	1.24274400	5.42407200
C	5.56827700	1.13470300	2.98556500
C	6.54968400	0.00042700	5.39885400
C	5.49376900	1.85100500	4.22817500
C	5.07197400	1.72897000	1.80287200
H	6.92544000	-0.45373200	6.31060200
H	4.90608800	3.67760500	5.19277900
H	5.91640200	1.80241600	6.35280900
C	4.52037500	3.02906100	1.84140400
C	3.97891400	3.68223200	0.68413500
C	4.47200800	3.74008700	3.09115300
H	3.91078700	5.58327800	4.08257300
C	4.95173300	3.13496600	4.25069800
C	3.47577000	4.94854300	0.76022300
H	3.93167900	3.12793800	-0.24729400
H	3.04652100	5.42602700	-0.11613900
C	3.45779500	5.65800000	1.99639900

H	3.05261800	6.66542700	2.02949500
C	3.92999500	5.06426300	3.12752500
C	5.65766900	-6.01096500	-0.99626600
H	5.70246000	-6.49575800	-0.01144000
H	5.21391600	-6.74570900	-1.67777800
C	6.71270200	-3.21813300	-0.98680100
H	6.95149400	-2.38593300	-0.31577500
H	6.99611800	-2.88022100	-1.99267500
C	7.55528500	-4.44360600	-0.63673400
H	8.60955700	-4.22937700	-0.83900500
H	7.46918000	-4.66201400	0.43579900
C	7.07328100	-5.64989500	-1.43577700
H	7.73699100	-6.50938600	-1.29787600
H	7.08213700	-5.40168700	-2.50550300
C	5.42875600	-1.09551400	-3.29107500
H	5.48261900	-2.18377200	-3.21101300
H	4.42767700	-0.88064900	-3.69623500
C	6.87577500	1.47646900	-2.87421300
H	6.73079000	2.56060000	-2.81041100
H	7.95000000	1.29807600	-2.72614600
C	6.47753300	-0.56269900	-4.26336400
H	7.47413300	-0.92156900	-3.97107400
H	6.27708000	-0.94965900	-5.26767600
C	6.46830400	0.96380100	-4.25341700
H	5.45590300	1.32065200	-4.48821500
H	7.14031800	1.36837800	-5.01701300
C	0.30280200	2.02515000	-2.00458000
C	-0.87134900	2.78363600	-1.89644000

C -0.81362200 4.17362000 -1.82906200

C 0.42216100 4.80500300 -1.89032000

C 1.59631100 4.09111000 -2.08338900

C 1.51840100 2.70834100 -2.14185100

H -1.72611900 4.74891900 -1.71880400

H 2.55163000 4.59325000 -2.18205900

Br -2.53259300 1.91109400 -1.74021800

O 0.25038300 0.68233000 -2.00977100

H 0.97209700 0.37128500 -1.41633300

Br 3.09494800 1.70323000 -2.40676800

Br 0.52432500 6.68232200 -1.65312200

Zero-point correction= 1.466828 (Hartree/Particle)

Thermal correction to Energy= 1.532555

Thermal correction to Enthalpy= 1.533357

Thermal correction to Gibbs Free Energy= 1.369357

Sum of electronic and zero-point Energies= -15022.487454

Sum of electronic and thermal Energies= -15022.421727

Sum of electronic and thermal Enthalpies= -15022.420926

Sum of electronic and thermal Free Energies= -15022.584925

AP-8-TS1*S3*

C 3.96253100 -3.02455200 1.48490800

H 5.09103100 -4.17898300 2.88556900

C 5.15123900 -3.47699400 2.05607800

C 5.30692900 -1.74413000 -0.10061100

C 6.40424300 -3.03729600 1.62953700

C 4.06455100 -2.14035500 0.41023300

C 6.48850800 -2.14051800 0.55307000

C	4.66792200	0.37089500	-1.20897200
C	5.32061000	-0.85861600	-1.30121500
C	5.32411900	0.91844700	-3.42789400
C	5.90031100	-1.25051300	-2.52478800
C	4.67198400	1.29413600	-2.25628400
C	5.91221400	-0.33907200	-3.59043000
H	5.35007100	1.62432500	-4.25602000
O	2.92128100	-1.64643000	-0.19000400
O	3.99750200	0.65831800	-0.03403100
P	2.53784500	-0.07788800	0.11735300
O	1.61744200	0.35893900	-0.96956600
O	2.19100200	0.01637200	1.57927700
C	-3.19150800	-1.92937300	-2.23997100
C	-0.93633500	-0.23010300	-2.31346800
C	-3.14961800	-0.77580200	-3.04886200
C	-2.08660500	-2.22212000	-1.44799100
C	-0.93279100	-1.34347000	-1.39803600
C	-2.02460400	0.09005700	-3.06193200
H	0.03889900	-1.79251700	-1.20139100
H	-2.01542900	0.97729700	-3.68228000
H	-0.03814000	0.37115900	-2.35470200
C	-4.37294600	-2.84085500	-2.30058000
C	-6.50134300	-4.65109500	-2.54374200
C	-5.65255400	-2.47983100	-1.83207800
C	-4.18497600	-4.10323900	-2.87101500
C	-5.23483500	-5.00614300	-2.99310000
C	-6.70013400	-3.39986500	-1.96781200
H	-3.19430600	-4.37146800	-3.22646800

H	-5.06315700	-5.97950700	-3.44233500
H	-7.69590500	-3.14357200	-1.61860400
H	-7.33356800	-5.34165900	-2.63841400
O	-2.15381700	-3.33603500	-0.72840600
O	-4.18931300	-0.59495000	-3.84732700
C	-1.06910900	-3.72006800	0.12079400
H	-0.21518700	-4.06235900	-0.47482600
H	-0.77520600	-2.89805200	0.77649600
H	-1.45915400	-4.54859900	0.70853200
C	-4.41504800	0.70892900	-4.37691900
H	-3.68455600	0.94760400	-5.15599900
H	-5.41112200	0.67670100	-4.81720800
H	-4.38477200	1.44620800	-3.57024700
P	-5.96275000	-0.89257700	-0.95212800
O	-5.08383400	0.24301000	-1.40551700
H	0.40813600	1.45263300	-4.71204800
C	0.93776200	2.22512400	-4.16043900
H	2.49126900	0.90710500	-3.53953000
C	2.10315700	1.91964500	-3.52025900
C	1.04392900	4.51805100	-3.40840400
C	2.82804700	2.91898200	-2.79121300
C	0.39647600	3.54412900	-4.10538000
C	2.27714800	4.24516600	-2.73428100
C	4.04235200	2.64163000	-2.13536200
H	-0.53826200	3.76253900	-4.61343200
H	2.53688900	6.24677500	-1.99917300
H	0.63917900	5.52511200	-3.34943600
C	4.71038800	3.65424200	-1.41615400

C	5.93303800	3.41555000	-0.70776200
C	4.15564600	4.97909700	-1.37598100
H	4.40012000	6.99279200	-0.61402000
C	2.95581200	5.24327800	-2.03661000
C	6.54802900	4.41272100	-0.00846700
H	6.36618200	2.42059100	-0.72993000
H	7.46986100	4.20814200	0.52759400
C	5.99359500	5.72584500	0.02949000
H	6.49878600	6.50454100	0.59277100
C	4.83614000	5.99730900	-0.63684400
H	2.46917700	-6.80670100	-1.24135800
C	2.04979600	-6.37650300	-0.33694200
H	3.49293900	-4.81545400	-0.24401700
C	2.61966200	-5.26682800	0.21650800
C	0.36954100	-6.45619200	1.39805100
C	2.08349100	-4.67332600	1.40683400
C	0.90969300	-6.98773800	0.26545900
C	0.92368500	-5.28004800	1.99869800
C	2.66176000	-3.53848900	2.01005100
H	0.47804200	-7.87676800	-0.18410300
H	-0.53082700	-5.16403700	3.57762300
H	-0.50116900	-6.90974700	1.86608400
C	2.08513700	-2.97659700	3.16505400
C	2.64228000	-1.82854300	3.81749100
C	0.90007400	-3.56695100	3.72575600
H	-0.57927000	-3.44099500	5.30668600
C	0.35307500	-4.70683900	3.13620000
C	2.06121800	-1.30663000	4.93497600

H	3.52084600	-1.36141400	3.38787900
H	2.49009700	-0.42747200	5.40564200
C	0.88158200	-1.89220900	5.48555000
H	0.43843200	-1.46337300	6.37994300
C	0.31782200	-2.98387900	4.89656900
H	1.37764000	0.71906800	2.33068100
C	-0.35568800	1.29205300	3.29315200
N	-1.26099100	0.82404900	2.45730300
C	-2.55279500	1.13288200	2.91464900
Br	-1.10384300	-0.25819200	0.50605000
O	-3.56468400	0.89355800	2.30343100
O	0.90573000	1.26200700	3.16687300
C	-2.45855600	1.80410000	4.28313400
H	-2.97294500	2.76782500	4.25419600
H	-2.96895200	1.17240300	5.01516500
C	-0.95796000	1.92803500	4.52784400
H	-0.61350700	2.96214300	4.61617000
H	-0.60159500	1.38173800	5.40580800
C	-1.44671600	4.46213400	2.28842700
C	-0.56859000	3.01773900	0.05269500
C	-0.08583800	4.28526700	2.08891400
C	-2.38095900	3.91720500	1.41733300
C	-1.93886600	3.18781400	0.31987800
C	0.33359100	3.56381000	0.97845300
H	0.64203900	4.69073600	2.78232100
H	-3.44382600	4.02158100	1.60355200
O	-0.19379900	2.37203700	-1.05410000
H	0.65401900	1.87091000	-0.94063300

Br	-3.20982100	2.26542000	-0.71135100
Br	2.17274800	3.26051200	0.72618300
Br	-2.03724900	5.43030000	3.80939700
C	6.51835000	-0.68466600	-4.93754100
H	5.70443500	-0.82663700	-5.66186700
H	7.10154900	0.17027300	-5.29827900
C	6.43815400	-2.66085000	-2.69624800
H	5.76141900	-3.36179100	-2.19475800
H	7.40542800	-2.76358300	-2.18615300
C	6.62137000	-3.04931300	-4.16278800
H	7.15460500	-4.00345700	-4.22428600
H	5.64099400	-3.19505800	-4.63514500
C	7.37497300	-1.94721100	-4.90128000
H	7.62905500	-2.25471300	-5.92073900
H	8.31951500	-1.74337000	-4.37929800
C	7.82032000	-1.50445400	0.19587700
H	7.88135100	-1.28411400	-0.87119800
H	7.85629000	-0.52673300	0.69896500
C	7.64128500	-3.56944700	2.32907100
H	7.42839000	-3.69429800	3.39631400
H	7.85257500	-4.57534300	1.93948000
C	9.01991400	-2.33271500	0.64938100
H	9.08300700	-3.25340800	0.05279300
H	9.94258400	-1.77012900	0.47338500
C	8.87528900	-2.69355300	2.12535000
H	8.77616600	-1.77065900	2.71139200
H	9.76517000	-3.21524100	2.49244600
C	-5.78160500	-1.30771600	0.82802300

C	-4.19178400	-2.23490000	2.56794000
C	-6.66871700	-2.54611100	2.84953000
C	-5.28947300	-3.16385800	3.08740000
C	-6.89157600	-2.22285800	1.36791100
C	-4.39517600	-1.91434000	1.08608100
H	-4.20793200	-1.29842900	3.13845200
H	-6.75303200	-1.61897200	3.43311100
H	-5.23147500	-4.12841400	2.56309700
H	-6.89332700	-3.16266100	0.79684900
H	-4.29395500	-2.84367200	0.50884000
H	-5.82140700	-0.33274500	1.33671500
H	-3.19937900	-2.68186300	2.71218700
H	-7.45883100	-3.21867400	3.20247700
H	-5.14820800	-3.37131100	4.15444000
H	-7.87828600	-1.76272900	1.23670900
H	-3.62186100	-1.21980100	0.74758600
C	-7.72726600	-0.47743100	-1.24250500
C	-9.38994600	0.25909500	-3.00351100
C	-9.53240900	1.20127700	-0.66969900
C	-9.75586700	1.47944300	-2.15696100
C	-8.09419700	0.75358100	-0.39457600
C	-7.95092700	-0.18769400	-2.73500800
H	-10.07525800	-0.56664600	-2.76545400
H	-10.22401300	0.41245400	-0.34135000
H	-9.12849300	2.32852200	-2.46125300
H	-7.39770700	1.56672500	-0.63809000
H	-7.25636200	0.60970300	-3.03197300
H	-8.36978600	-1.31246900	-0.93407500

H -9.51835900 0.48033600 -4.06901200

H -9.76234900 2.09255600 -0.07557100

H -10.79659700 1.77058200 -2.33742200

H -7.96820600 0.53618800 0.67289300

H -7.70283600 -1.06966600 -3.33771600

Zero-point correction= 1.466854 (Hartree/Particle)

Thermal correction to Energy= 1.532231

Thermal correction to Enthalpy= 1.533032

Thermal correction to Gibbs Free Energy= 1.370443

Sum of electronic and zero-point Energies= -15022.488527

Sum of electronic and thermal Energies= -15022.423151

Sum of electronic and thermal Enthalpies= -15022.422349

Sum of electronic and thermal Free Energies= -15022.584939

AP-8-TS2^{R}

C -3.41559500 1.91078500 -2.33225000

H -4.16830400 3.63737300 -3.34653600

C -4.42515300 2.79547800 -2.70631800

C -5.10591400 0.60915600 -1.13105000

C -5.74021800 2.66032700 -2.25658500

C -3.78152600 0.83320500 -1.52457700

C -6.08516700 1.57200300 -1.43944900

C -4.68003200 -0.92289100 0.77147800

C -5.39936900 -0.65751400 -0.39648700

C -5.74692000 -3.03894900 0.98921100

C -6.26163600 -1.64442800 -0.92045300

C -4.82563000 -2.11769400 1.47975200

C -6.45756800 -2.82966700 -0.19498700

H	-5.88663400	-3.97321800	1.53026700
O	-2.80820100	-0.05363600	-1.10692000
O	-3.79062200	0.02951100	1.22118300
P	-2.34104700	0.09689300	0.46039900
O	-1.59931100	-1.18349100	0.78088100
O	-1.77164800	1.43914600	0.75793600
C	2.21173400	-3.41099100	0.47956800
C	0.25601100	-2.94013700	-1.49657100
C	1.58653900	-2.14914700	0.43061700
C	1.82397100	-4.39854200	-0.41601300
C	0.80714600	-4.17204300	-1.40197900
C	0.55311000	-1.86783400	-0.56291600
H	0.51722600	-4.95833300	-2.08707100
H	-0.48124900	-1.64133900	0.08346900
H	-0.47015300	-2.70995500	-2.27076200
C	3.35109900	-3.60151700	1.42155100
C	5.44364200	-3.93050700	3.24445000
C	4.55843600	-2.90059800	1.22758800
C	3.20985100	-4.45148100	2.51737400
C	4.25093600	-4.62110800	3.42531100
C	5.59224700	-3.07783900	2.15255700
H	2.26463800	-4.96639200	2.66213100
H	4.12531600	-5.28378600	4.27597100
H	6.53070300	-2.54468000	2.03187400
H	6.25847800	-4.04796100	3.95199000
O	2.47508900	-5.55943900	-0.32235700
O	1.90524100	-1.35893800	1.41601200
C	2.15937600	-6.61874100	-1.22106800

H	1.11921100	-6.93706700	-1.10255500
H	2.34976000	-6.32492800	-2.25751300
H	2.82284800	-7.43794400	-0.94672500
C	1.51477700	0.02014700	1.48529800
H	0.43436300	0.12336700	1.37327400
H	1.81979900	0.33758400	2.48296300
H	2.04900600	0.57578300	0.71679300
P	4.82341900	-1.89156400	-0.29334800
O	3.61210600	-1.15238300	-0.80503000
H	-1.43534100	-5.46309600	0.32498300
C	-1.66752600	-4.98961400	1.27469000
H	-3.18488300	-3.75375400	0.44236300
C	-2.64997800	-4.04643500	1.33928500
C	-1.19216000	-4.75046900	3.63205200
C	-2.97886900	-3.40176400	2.57767900
C	-0.93095800	-5.35785300	2.44059000
C	-2.21344500	-3.75176500	3.74158800
C	-3.99360100	-2.42918200	2.67760300
H	-0.16041100	-6.12101600	2.36866500
H	-1.89212400	-3.38053300	5.83348800
H	-0.63065900	-5.00994700	4.52613200
C	-4.23765500	-1.78124300	3.90349200
C	-5.25031200	-0.77856200	4.05167700
C	-3.46025200	-2.13228000	5.05924800
H	-3.12242000	-1.74602900	7.16557500
C	-2.47396800	-3.11323800	4.95370900
C	-5.46717000	-0.16948300	5.25230900
H	-5.84610800	-0.50589900	3.18654000

H	-6.23634100	0.59152600	5.34239300
C	-4.69114200	-0.51844600	6.39745800
H	-4.87922200	-0.02006900	7.34358800
C	-3.72115100	-1.47016400	6.30120800
H	-2.12164500	-1.73058400	-5.36906800
C	-1.56232600	-0.86764200	-5.01935300
H	-3.18058700	-0.12259300	-3.86186000
C	-2.15401500	0.02967300	-4.17864800
C	0.49011100	0.40429700	-5.03403000
C	-1.44390800	1.17693900	-3.69783300
C	-0.21533600	-0.68317900	-5.45216300
C	-0.09524500	1.36859500	-4.15344100
C	-2.00809900	2.09358900	-2.78732600
H	0.23940700	-1.41123700	-6.11731400
H	1.66096900	2.60299600	-4.04039600
H	1.51774500	0.55974000	-5.35285000
C	-1.25101800	3.18576300	-2.31881600
C	-1.74667500	4.10162400	-1.33296800
C	0.08684700	3.38157300	-2.80435400
H	1.85693600	4.62974400	-2.71568200
C	0.63696300	2.46555200	-3.69885700
C	-0.99279700	5.15921400	-0.91409800
H	-2.72732800	3.92729900	-0.90308200
H	-1.37906400	5.82868000	-0.15150100
C	0.31878100	5.37326400	-1.43361700
H	0.90440500	6.21651300	-1.07758000
C	0.84451100	4.50298700	-2.34034500
Br	0.67132200	-0.18251700	-1.51871100

C	3.11757100	5.14115400	1.90825500
C	1.32179900	3.31918700	0.74615900
C	1.81192700	5.07738300	2.37073400
C	3.55728700	4.31159800	0.88187600
C	2.66233400	3.41476900	0.32393000
C	0.93164400	4.17081000	1.79024800
H	1.47285900	5.72182500	3.17324900
H	4.58016100	4.36075500	0.52652700
O	0.54539700	2.45374700	0.11498900
H	-0.36634400	2.28566100	0.48457100
Br	3.21326600	2.24965700	-1.05506300
Br	-0.84534700	4.07551000	2.41083500
Br	4.32819900	6.37389100	2.68805400
C	-7.37630400	-3.93473800	-0.68374600
H	-6.75996400	-4.79014300	-0.99323400
H	-7.98808100	-4.28841200	0.15413900
C	-6.90053800	-1.45967800	-2.28674000
H	-6.17457700	-0.98777100	-2.95809500
H	-7.74543500	-0.76121500	-2.22230100
C	-7.40991900	-2.77057400	-2.88388900
H	-7.98338600	-2.55921900	-3.79215400
H	-6.56141200	-3.40335600	-3.17625400
C	-8.25832400	-3.51061600	-1.85459800
H	-8.74189600	-4.38887700	-2.29433400
H	-9.05660100	-2.84488200	-1.50040700
C	-7.45554900	1.51173400	-0.79033600
H	-7.77004400	0.47929600	-0.62786300
H	-7.35627200	1.94829000	0.21429300

C	-6.77024200	3.68247300	-2.70222600
H	-6.30335800	4.67251300	-2.74628700
H	-7.07157500	3.44105600	-3.73140900
C	-8.51449700	2.29660500	-1.56008500
H	-8.72332300	1.80062000	-2.51825700
H	-9.45030700	2.30974100	-0.99171100
C	-8.01624100	3.71617800	-1.81964100
H	-7.77748000	4.19119700	-0.85928600
H	-8.79142900	4.32489600	-2.29661100
C	5.39486700	-3.17348200	-1.48608100
C	7.19310000	-4.76846700	-2.24004400
C	5.47817100	-3.91547500	-3.88761500
C	6.92693600	-4.36958500	-3.69346600
C	5.12456600	-2.76481000	-2.94167500
C	6.84584800	-3.62728500	-1.27860600
H	6.58205200	-5.64603400	-1.98681200
H	4.80529200	-4.76265200	-3.69039500
H	7.60030400	-3.54565000	-3.96865100
H	5.72961500	-1.88689100	-3.20231900
H	7.52371800	-2.78279000	-1.46581400
H	4.74145400	-4.03304600	-1.26040500
H	8.24062900	-5.06140800	-2.10927600
H	5.30914300	-3.61106800	-4.92635900
H	7.15815200	-5.20503000	-4.36349000
H	4.07654000	-2.46813200	-3.04985500
H	7.00402300	-3.94553300	-0.24060500
C	6.16699800	-0.70666900	0.10165200
C	6.64813500	1.38341700	1.46755900

C	7.56340300	1.17732800	-0.86596100
C	7.01068700	2.13702600	0.18733100
C	6.56038800	0.06220500	-1.17315500
C	5.64163700	0.26842500	1.17453800
H	7.55865000	0.95476300	1.91011200
H	8.50060800	0.73361000	-0.50088400
H	6.10399900	2.60996600	-0.21430500
H	5.65110200	0.49631900	-1.60937400
H	4.71669700	0.71781700	0.79093900
H	7.05130700	-1.24129500	0.47522600
H	6.22551000	2.07075200	2.20928500
H	7.80460500	1.71704100	-1.78842100
H	7.73178300	2.93419000	0.39911000
H	6.98380600	-0.62185400	-1.91717900
H	5.38412300	-0.27108700	2.09331900

Zero-point correction= 1.369103 (Hartree/Particle)

Thermal correction to Energy= 1.429912

Thermal correction to Enthalpy= 1.430714

Thermal correction to Gibbs Free Energy= 1.274798

Sum of electronic and zero-point Energies= -14662.035788

Sum of electronic and thermal Energies= -14661.974980

Sum of electronic and thermal Enthalpies= -14661.974178

Sum of electronic and thermal Free Energies= -14662.130093

AP-9-TS1^{R}

C	3.59396900	-1.93440000	0.09522600
C	1.51278400	-1.23242800	-1.69270000
C	3.77450200	-1.91718700	-1.30254900

C	2.32383000	-1.65745900	0.59573600
C	1.24928000	-1.23213600	-0.27639300
C	2.74726600	-1.50503800	-2.19354800
H	0.22934900	-1.47686200	0.01430400
H	2.92536100	-1.46375800	-3.25965300
H	0.69020500	-0.96606300	-2.34515000
C	4.72073200	-2.33968700	0.98416300
C	6.77940600	-3.29519800	2.63420000
C	5.93652600	-1.62883300	1.06243700
C	4.55613400	-3.49496600	1.75305300
C	5.56886400	-3.97530400	2.57379500
C	6.95017700	-2.13241400	1.88890800
H	3.60382700	-4.01132900	1.69868200
H	5.41336200	-4.87737800	3.15797900
H	7.89708500	-1.60755900	1.96667200
H	7.58713900	-3.65922900	3.26149100
O	2.15383600	-1.75802900	1.90993000
O	4.94118600	-2.37791600	-1.72973200
C	0.87206400	-1.47805300	2.48596800
H	0.10520600	-2.14904800	2.08987500
H	0.59228700	-0.43933800	2.30169600
H	0.99529900	-1.65491000	3.55370800
C	5.33814600	-2.09857800	-3.06803300
H	4.70636700	-2.63357500	-3.78443800
H	6.36106600	-2.46515600	-3.15095600
H	5.30887600	-1.02010000	-3.24229300
P	6.19293000	0.01029400	0.25365100
O	5.42588500	0.23034900	-1.02350400

H	-1.42843700	2.13152700	0.88089700
C	-0.13383400	3.70111400	0.82422800
N	0.97263900	3.08165100	0.49260300
C	2.04224500	3.98865700	0.40240700
Br	1.17277000	0.92151300	0.06116100
O	3.16477600	3.68554100	0.08732700
O	-1.28771500	3.18358700	0.98593000
C	1.54631800	5.37840700	0.80199000
H	1.76818200	6.08030700	-0.00443000
H	2.09563800	5.69918800	1.69160900
C	0.05310100	5.18061300	1.05935100
H	-0.59827100	5.72961400	0.37358800
H	-0.26102100	5.41451800	2.08026200
C	0.37753900	5.35659400	-2.36905700
C	0.13529000	2.57881400	-2.70688200
C	-0.85947600	4.72124200	-2.25513600
C	1.49567200	4.56224500	-2.63489600
C	1.39931300	3.18205900	-2.80324400
C	-1.00803100	3.34351700	-2.42558200
H	-1.75198600	5.31278000	-2.04673700
H	2.47802400	5.02742100	-2.70327200
O	0.07098100	1.24411900	-2.94429800
H	-0.62285800	0.81398200	-2.39092800
C	5.74350000	1.22404300	1.55336600
C	3.79764400	2.14187500	2.87595000
C	6.11812800	2.21416000	3.84471900
C	4.62718000	2.06184600	4.15846700
C	6.58916000	1.16338300	2.83189800

C	4.24765300	1.08909000	1.86334800
H	3.91723800	3.13656700	2.42789200
H	6.29782700	3.21605800	3.43078000
H	4.45839700	1.08938200	4.64210500
H	6.49052300	0.16682200	3.28395700
H	4.04737600	0.08956200	2.27871200
H	5.89347200	2.19907400	1.06460500
H	2.72827400	2.00985300	3.08433600
H	6.71356300	2.13623500	4.76150000
H	4.31257300	2.83209300	4.87201800
H	7.65287100	1.31367600	2.60678700
H	3.67714800	1.18298500	0.93785700
C	8.00191700	0.15297300	-0.03616000
C	9.91783500	-0.74371300	-1.43008700
C	9.81277700	1.71137100	-0.87221800
C	10.23611200	0.67267200	-1.91175700
C	8.32915800	1.57747200	-0.51670600
C	8.43429700	-0.88619800	-1.08248000
H	10.52179200	-0.96871700	-0.53972100
H	10.41435400	1.57670800	0.03787900
H	9.69689700	0.86264300	-2.85000800
H	7.71335800	1.80294100	-1.39724200
H	7.82895400	-0.72835600	-1.98542100
H	8.54873300	-0.02307100	0.89929400
H	10.19363000	-1.47899700	-2.19434400
H	10.01451200	2.72374000	-1.23915700
H	11.30492100	0.76948500	-2.13246800
H	8.06519500	2.31069800	0.25462200

H	8.21757500	-1.90005700	-0.72434200
C	-5.41998900	1.21588500	0.85086500
H	-6.90381100	1.85142700	2.25247800
C	-6.43795200	0.99065800	1.77652800
C	-5.28685900	-1.20225800	0.53270700
C	-6.84798400	-0.29130100	2.14447400
C	-4.85138400	0.09418200	0.24695200
C	-6.24878300	-1.40761700	1.53977000
C	-3.28248700	-2.50397900	-0.13435500
C	-4.66551600	-2.32923600	-0.22055400
C	-3.43761400	-4.48639500	-1.44675700
C	-5.42775900	-3.17761600	-1.04956700
C	-2.64676800	-3.61110900	-0.70438000
C	-4.80056100	-4.27214500	-1.66076800
H	-2.96521500	-5.36037500	-1.89132400
O	-3.83651200	0.24798100	-0.67603300
O	-2.54178300	-1.56886900	0.56566600
P	-2.31613700	-0.11539300	-0.17838300
O	-1.48985800	-0.29801100	-1.40644700
O	-1.91608300	0.80899900	0.94308400
H	-0.13970100	-3.48563100	-4.94860400
C	0.16252400	-3.79283100	-3.95211100
H	-1.67296600	-3.18485100	-3.05995800
C	-0.69677900	-3.63138900	-2.90360000
C	1.87620700	-4.68540400	-2.50129800
C	-0.32278000	-4.02191700	-1.57498400
C	1.45935600	-4.35346400	-3.75566600
C	1.01289000	-4.51380200	-1.37189200

C	-1.20767500	-3.92566300	-0.48062900
H	2.11824900	-4.49031600	-4.60823900
H	2.45667000	-5.19028000	0.06598200
H	2.87446200	-5.08108200	-2.32820700
C	-0.76199400	-4.25105000	0.82001500
C	-1.61909400	-4.19207800	1.96920700
C	0.59483400	-4.68308300	1.01923800
H	2.06356800	-5.33909000	2.47469000
C	1.44358400	-4.82048600	-0.08134000
C	-1.15367400	-4.49473500	3.21569600
H	-2.65525200	-3.89985200	1.84026300
H	-1.82155400	-4.43569800	4.06960600
C	0.19913700	-4.89935400	3.41137700
H	0.54880200	-5.14131400	4.41029700
C	1.03982700	-4.99859800	2.34335000
H	-6.70889100	2.51415900	-3.72436200
C	-6.22337300	3.08073000	-2.93572200
H	-6.22053000	1.44593000	-1.57600700
C	-5.94985900	2.48408300	-1.73936800
C	-5.26615600	5.16885600	-2.18619800
C	-5.29675800	3.20258900	-0.68633200
C	-5.87781800	4.44498400	-3.16630700
C	-4.95087100	4.57648700	-0.92214200
C	-4.97512200	2.60666800	0.55006800
H	-6.10490800	4.90065900	-4.12524800
H	-4.05686400	6.35063400	-0.10269900
H	-4.99681100	6.20985000	-2.34740700
C	-4.28830800	3.34228700	1.53539800

C	-3.86986000	2.75871400	2.77671600
C	-3.96260500	4.72071100	1.29462400
H	-3.06282200	6.51237400	2.12004300
C	-4.30427300	5.30580900	0.07690400
C	-3.21335600	3.49840000	3.71511600
H	-4.06225200	1.70538700	2.94840200
H	-2.89755700	3.03490300	4.64492300
C	-2.92512600	4.87695400	3.48766500
H	-2.41387800	5.45121500	4.25528400
C	-3.28374400	5.46463300	2.31169400
C	-5.55200600	-5.22843500	-2.56709300
H	-5.24452500	-5.04066300	-3.60503800
H	-5.24504900	-6.25551300	-2.33874700
C	-6.89024300	-2.86565300	-1.31739200
H	-7.00554900	-1.78279000	-1.43765800
H	-7.50230100	-3.13645700	-0.44652400
C	-7.43665900	-3.60127300	-2.54031100
H	-8.52171800	-3.46608000	-2.59235300
H	-7.01218600	-3.16868300	-3.45566600
C	-7.06749300	-5.07978800	-2.46555100
H	-7.55231800	-5.65047400	-3.26403200
H	-7.42078900	-5.49284800	-1.51112800
C	-6.53602600	-2.80335900	2.06470500
H	-6.46883500	-3.54810800	1.26973600
H	-5.73093500	-3.05562700	2.77117600
C	-7.95203000	-0.43517400	3.17557700
H	-7.85525000	0.35926000	3.92359800
H	-8.91615700	-0.26784500	2.67504300

C	-7.87403100	-2.90501700	2.79294500
H	-8.69755400	-2.79947900	2.07316700
H	-7.96981800	-3.89542700	3.24986700
C	-7.97797800	-1.80623400	3.84713300
H	-7.13267700	-1.89530700	4.54200000
H	-8.89375200	-1.91263400	4.43776900
C	2.61276000	2.32124000	-3.01993200
H	2.74166600	1.64074300	-2.17173400
H	2.51237400	1.70609000	-3.92083300
H	3.51599000	2.93077500	-3.10715100
C	-2.36780500	2.71102400	-2.29032500
H	-2.43935700	1.75597900	-2.81636900
H	-2.61749800	2.53000000	-1.23715100
H	-3.14034100	3.37580900	-2.68953800
C	0.49106600	6.85526400	-2.23392800
H	-0.02255400	7.36893400	-3.05481900
H	0.04127700	7.21883200	-1.30129800
H	1.53695000	7.17748500	-2.25007300

Zero-point correction= 1.583094 (Hartree/Particle)

Thermal correction to Energy= 1.648368

Thermal correction to Enthalpy= 1.649170

Thermal correction to Gibbs Free Energy= 1.489107

Sum of electronic and zero-point Energies= -7426.703378

Sum of electronic and thermal Energies= -7426.638104

Sum of electronic and thermal Enthalpies= -7426.637302

Sum of electronic and thermal Free Energies= -7426.797364

AP-9-TS1^{S}

C	-3.60273700	-0.87531800	0.37138600
C	-1.50836200	-0.42421600	-1.50192700
C	-3.73559400	-1.15858100	-0.99552900
C	-2.40277000	-0.30929000	0.80561800
C	-1.25387100	-0.20935400	-0.08688500
C	-2.69964900	-0.89079700	-1.94326600
H	-0.52176300	0.56964700	0.12497500
H	-2.85156600	-1.08200500	-2.99660900
H	-0.68180900	-0.23939000	-2.17743600
C	-4.60762300	-1.32809800	1.37582800
C	-6.27061500	-2.33685300	3.39715600
C	-5.94597300	-0.90585100	1.45036800
C	-4.13345200	-2.26790700	2.30253100
C	-4.95208400	-2.77380800	3.30142500
C	-6.75496500	-1.41404000	2.47867900
H	-3.10195700	-2.60132700	2.21412600
H	-4.56086400	-3.50435700	4.00239900
H	-7.78244300	-1.07589200	2.57565700
H	-6.92050600	-2.71245000	4.18133900
O	-2.31290400	0.01934300	2.07998700
O	-4.87261300	-1.74157800	-1.35044600
C	-1.06200000	0.44105300	2.64718800
H	-0.64002900	1.29030300	2.10570900
H	-0.35802900	-0.39227000	2.64672000
H	-1.30248300	0.73977900	3.66576900
C	-5.09669200	-2.05799400	-2.72251200
H	-5.07002800	-1.15387800	-3.33751500

H	-6.09071700	-2.50359000	-2.76126800
H	-4.35764300	-2.78324500	-3.07594500
P	-6.70619100	0.24699800	0.24504900
O	-5.71632500	0.99264500	-0.61429800
H	2.70425300	-1.97944300	1.14098000
C	2.25608300	-3.90573200	1.38306200
N	0.97351900	-3.85570400	1.15771900
C	0.41366500	-5.13288300	1.30744800
Br	-0.27138500	-1.97286000	0.47401200
O	-0.75794600	-5.38167300	1.16543100
O	3.08180400	-2.92176700	1.34226300
C	1.51138300	-6.12863500	1.69583600
H	1.55662100	-6.91347700	0.93721800
H	1.24864600	-6.59517500	2.64864900
C	2.77383300	-5.27124400	1.76530300
H	3.56257900	-5.56248300	1.06625600
H	3.21726400	-5.21248000	2.76348600
C	2.37486000	-5.92443500	-1.59225400
C	1.31722100	-3.38886800	-2.18291800
C	3.19323500	-4.79588100	-1.65266200
C	1.00687000	-5.74875300	-1.81414100
C	0.46288300	-4.50029400	-2.10827800
C	2.69488000	-3.52592300	-1.95053900
H	4.26563400	-4.89974300	-1.48309500
H	0.34018100	-6.60790900	-1.75342600
O	0.75497600	-2.20599000	-2.53790600
H	1.21006800	-1.44441400	-2.10380300
C	-7.84749000	-0.81261200	-0.75357000

C	-10.03438200	-2.04931900	-0.99076300
C	-8.87052300	-1.15353800	-3.03842100
C	-10.22159500	-1.45850200	-2.38879200
C	-8.03229900	-0.22088200	-2.16093000
C	-9.20282300	-1.12122200	-0.09859100
H	-9.52373200	-3.01873900	-1.07185300
H	-8.32725800	-2.09729200	-3.19147300
H	-10.80175800	-0.52811400	-2.31253100
H	-8.53778700	0.75110900	-2.08976200
H	-9.75968100	-0.18797100	0.06132200
H	-7.30202700	-1.76235900	-0.85348500
H	-11.00593000	-2.23977100	-0.52176700
H	-9.01306100	-0.70541500	-4.02797200
H	-10.80113000	-2.14404700	-3.01650800
H	-7.05322100	-0.02440300	-2.60834400
H	-9.06137700	-1.58680500	0.88259200
C	-7.70500000	1.41016600	1.26081500
C	-7.55334700	3.18656200	3.06587200
C	-9.18614900	3.46570600	1.17140600
C	-8.26462300	4.18718500	2.15483300
C	-8.42323300	2.42185600	0.35066100
C	-6.78415500	2.14469200	2.25035600
H	-8.29529500	2.67601500	3.69572100
H	-9.99109100	2.96634200	1.72900700
H	-7.51355300	4.75843300	1.59228200
H	-7.67256000	2.91821000	-0.27830600
H	-5.99625100	2.64762900	1.67209800
H	-8.45986200	0.84172100	1.82243500

H	-6.86548100	3.70575000	3.74257100
H	-9.66515700	4.18445900	0.49753200
H	-8.83420000	4.90853700	2.75094900
H	-9.11612500	1.90814400	-0.32481000
H	-6.28846900	1.42902600	2.91601600
C	5.78730900	0.73879300	0.70802100
H	7.43949600	0.97580600	2.04372400
C	6.61546900	1.48411000	1.54665500
C	4.54689600	2.79122900	0.24738700
C	6.40015400	2.83795800	1.80561400
C	4.74792500	1.41937600	0.07249500
C	5.33520100	3.49937800	1.17405600
C	2.14928000	2.97554300	-0.37001900
C	3.45579800	3.44323800	-0.53182500
C	1.34289900	4.70780100	-1.79677900
C	3.72133900	4.47715700	-1.45334100
C	1.06219600	3.62646400	-0.96405100
C	2.64607800	5.12008300	-2.08164100
H	0.50989000	5.23408300	-2.25927700
O	3.88875200	0.74194900	-0.76765700
O	1.93729000	1.87030700	0.43196700
P	2.39401400	0.40245800	-0.17399800
O	1.53575400	0.06244700	-1.34861700
O	2.51670500	-0.48576700	1.03112200
H	-1.18328100	2.06175200	-5.07091500
C	-1.56793300	2.22517700	-4.06851200
H	0.34714300	2.70237600	-3.27089000
C	-0.71254600	2.58938300	-3.06858800

C	-3.44976400	2.18684800	-2.55045000
C	-1.19104100	2.81445700	-1.73417100
C	-2.96083600	2.04491700	-3.81494800
C	-2.58201700	2.56149100	-1.47333700
C	-0.35034600	3.24501000	-0.68626800
H	-3.62034200	1.76647300	-4.63223000
H	-4.11795800	2.42237200	0.01323100
H	-4.49263500	1.99655600	-2.30920800
C	-0.87261800	3.40950400	0.61672900
C	-0.08378300	3.89459100	1.71244100
C	-2.25057300	3.09365100	0.87434200
H	-3.80392000	2.96194300	2.37784900
C	-3.07456800	2.67219300	-0.17176900
C	-0.61738200	4.03011200	2.96180100
H	0.95268300	4.16276400	1.53781600
H	0.00043900	4.40222100	3.77356900
C	-1.97796100	3.68878700	3.21762100
H	-2.37878600	3.79542200	4.22118300
C	-2.76557800	3.23213000	2.20315000
H	7.31255000	-0.26007900	-3.87294200
C	7.18891300	-0.90587900	-3.00901700
H	6.47772600	0.66553200	-1.76618900
C	6.72202400	-0.38947900	-1.83550500
C	7.36623500	-3.11269900	-2.04148600
C	6.53322400	-1.22027700	-0.68408400
C	7.51793000	-2.28927400	-3.11733800
C	6.86635900	-2.61292300	-0.79705000
C	6.02643200	-0.72241000	0.53369500

H	7.88910400	-2.67962800	-4.06007100
H	6.95348200	-4.50789900	0.21326700
H	7.61283400	-4.16956400	-2.10866900
C	5.81138400	-1.59237300	1.62076300
C	5.22224800	-1.15135600	2.85165900
C	6.16349000	-2.97999100	1.50112900
H	6.25249300	-4.89274900	2.51834500
C	6.68645400	-3.45610600	0.30048800
C	5.03578700	-2.01415200	3.89067700
H	4.89983500	-0.11934500	2.93409500
H	4.57954600	-1.66178400	4.81089300
C	5.42293500	-3.38280600	3.78130400
H	5.27826400	-4.05005500	4.62649600
C	5.96261900	-3.84927300	2.62029800
C	2.85060300	6.24284400	-3.08071300
H	2.63302500	5.86017900	-4.08745400
H	2.11527500	7.03264800	-2.88894500
C	5.15653200	4.84060100	-1.79545500
H	5.74691400	3.92058600	-1.86959600
H	5.60780200	5.41956200	-0.97843400
C	5.26928400	5.65403800	-3.08432500
H	6.29447000	6.02175400	-3.19496400
H	5.06071900	5.01168100	-3.94988800
C	4.26889600	6.80570900	-3.06176700
H	4.41599400	7.47576400	-3.91490200
H	4.42285800	7.40077100	-2.15148100
C	4.96036400	4.91011500	1.59321100
H	4.53165200	5.47146000	0.76133800

H	4.15209400	4.82315600	2.33486300
C	7.34465200	3.55921500	2.74941600
H	7.65066800	2.87554700	3.54881700
H	8.26019700	3.81206900	2.19622900
C	6.12119000	5.67537200	2.22362500
H	6.87785400	5.89881600	1.45873600
H	5.76204900	6.63409400	2.61202500
C	6.75397000	4.84031300	3.33359100
H	5.98474800	4.58957200	4.07572200
H	7.53399700	5.40458500	3.85522300
C	-1.01513000	-4.30390300	-2.30192500
H	-1.39690500	-3.61494600	-1.54116300
H	-1.23531100	-3.86406100	-3.28143200
H	-1.54995700	-5.25363400	-2.21380900
C	3.62657300	-2.34408200	-2.00737100
H	3.23403400	-1.53229300	-2.62473100
H	3.81367900	-1.93878900	-1.00488000
H	4.59721300	-2.64152000	-2.41655700
C	2.95895600	-7.29113000	-1.33081700
H	3.60425800	-7.61176500	-2.15688500
H	3.57331900	-7.31041800	-0.42209100
H	2.17237700	-8.04401600	-1.21912800

Zero-point correction= 1.583963 (Hartree/Particle)

Thermal correction to Energy= 1.649367

Thermal correction to Enthalpy= 1.650169

Thermal correction to Gibbs Free Energy= 1.488611

Sum of electronic and zero-point Energies= -7426.699393

Sum of electronic and thermal Energies= -7426.633990

Sum of electronic and thermal Enthalpies= -7426.633188

Sum of electronic and thermal Free Energies= -7426.794745

CPA-4-TS1^{R}

C	3.67066800	-1.40399700	0.17258500
C	1.53527500	-0.95719600	-1.64953900
C	3.78883900	-1.64429800	-1.21168300
C	2.42248300	-1.03492500	0.66336500
C	1.31170900	-0.73301700	-0.23130600
C	2.73934700	-1.34221100	-2.13259500
H	0.31759000	-1.01990100	0.11265900
H	2.88926900	-1.47757500	-3.19507600
H	0.68328000	-0.76167300	-2.29267700
C	4.83498400	-1.64417600	1.07291800
C	6.96426600	-2.25779100	2.79365100
C	6.05290500	-0.94272800	0.95017400
C	4.70433900	-2.61559900	2.06928900
C	5.75299100	-2.92641700	2.92564400
C	7.10128400	-1.27390800	1.81935100
H	3.75018900	-3.12122400	2.16803700
H	5.62384100	-3.68784700	3.68901100
H	8.04929400	-0.75020100	1.74882700
H	7.79836400	-2.49093300	3.44806700
O	2.29527000	-0.92679500	1.97697300
O	4.90743300	-2.23520400	-1.58704600
C	1.02683300	-0.59050700	2.55786400
H	0.25844400	-1.31311400	2.26960900
H	0.72943400	0.41514900	2.25800500
H	1.18782400	-0.63667200	3.63385900

C	5.24638800	-2.26444500	-2.97074300
H	4.53908900	-2.88402000	-3.53104000
H	6.23698900	-2.71579300	-3.01706300
H	5.27780000	-1.24405000	-3.35972300
P	6.28226000	0.48796800	-0.19537300
O	5.47773000	0.42969900	-1.46833100
H	-1.64378200	2.39459000	0.36866800
C	-0.48889100	4.16201100	-0.11797700
N	0.72615200	3.64967800	-0.20631000
C	1.62871800	4.61282800	-0.65021800
Br	1.14862400	1.37096800	-0.19428300
O	2.80203800	4.41308600	-0.86589900
O	-1.55349100	3.55647200	0.18537100
C	0.90794400	5.95400900	-0.82650000
H	1.00798300	6.27188400	-1.86745200
H	1.39508300	6.70584700	-0.20097800
C	-0.53098800	5.64770000	-0.42052400
H	-1.27398700	5.83029800	-1.20189000
H	-0.86196400	6.17594400	0.47786900
C	5.86046900	1.95605800	0.81768200
C	3.90990900	3.18345000	1.86269600
C	6.20049600	3.35144400	2.89206600
C	4.69486900	3.31199400	3.16876200
C	6.65773100	2.10844800	2.11942400
C	4.34801900	1.95027400	1.07450400
H	4.06992000	4.07508600	1.24480800
H	6.43477300	4.24838600	2.30224900
H	4.47180200	2.45261800	3.81720400
H	6.49636900	1.22288600	2.74909100
H	4.07937600	1.04948800	1.64850300

H	6.08058000	2.80979800	0.15865600
H	2.83011400	3.11893000	2.04654700
H	6.76268300	3.42982200	3.82964400
H	4.38982000	4.21159000	3.71553000
H	7.73472800	2.16804700	1.91408700
H	3.81978900	1.90467500	0.12020400
C	8.08237400	0.55155700	-0.56060700
C	9.95947000	-0.65342500	-1.76392300
C	9.86837000	1.86372400	-1.78450200
C	10.26248300	0.61136800	-2.56852300
C	8.39572800	1.82479300	-1.36576500
C	8.48676900	-0.70342900	-1.35089300
H	10.58863100	-0.66965700	-0.86279800
H	10.49610000	1.93839600	-0.88530500
H	9.69627600	0.58292900	-3.50971800
H	7.75457000	1.84571400	-2.25672200
H	7.85541200	-0.75499900	-2.24857500
H	8.65385800	0.59432400	0.37596900
H	10.21386600	-1.54729600	-2.34445600
H	10.05855400	2.76347200	-2.37981300
H	11.32439700	0.64856600	-2.83563400
H	8.15540200	2.71742200	-0.77675800
H	8.28209600	-1.60690300	-0.76394200
C	-5.50047600	1.49516500	0.25940000
H	-7.09758300	2.28085900	1.44374600
C	-6.55922100	1.37897600	1.15851800
C	-5.22969900	-0.92934300	0.38862700
C	-6.92428600	0.16063100	1.73336600
C	-4.83755300	0.32038400	-0.09745400
C	-6.23854500	-1.00835100	1.36765300

C	-3.14287100	-2.22435500	0.02890200
C	-4.52693200	-2.13574600	-0.13584500
C	-3.14969000	-4.39548300	-0.95184500
C	-5.21317200	-3.14532800	-0.84177300
C	-2.43200600	-3.37194600	-0.33484700
C	-4.51095600	-4.29047700	-1.24285300
H	-2.61914300	-5.30293800	-1.23432900
O	-3.78076600	0.37592400	-0.98102700
O	-2.47869900	-1.15377100	0.59517600
P	-2.27058300	0.15878300	-0.38276000
O	-1.37184200	-0.13720100	-1.51771000
O	-1.98181400	1.24830100	0.64429600
H	0.18938600	-3.84942300	-4.48125400
C	0.47464600	-3.97156100	-3.44082400
H	-1.39981800	-3.27984600	-2.70446200
C	-0.41569800	-3.66085100	-2.45332900
C	2.17432100	-4.54831000	-1.82267900
C	-0.06803500	-3.81154400	-1.06950700
C	1.78038600	-4.45035200	-3.12434400
C	1.27568300	-4.21652800	-0.75807600
C	-0.98748400	-3.57002100	-0.02637300
H	2.46159300	-4.71783900	-3.92723100
H	2.69684900	-4.60276700	0.80311900
H	3.17722600	-4.87974300	-1.56275100
C	-0.56458000	-3.65054600	1.31993300
C	-1.45135900	-3.42460800	2.42493600
C	0.79848400	-3.99855300	1.61786900
H	2.24617400	-4.36022500	3.19337200
C	1.67923600	-4.29197400	0.57458200
C	-1.00938000	-3.49781300	3.71394400

H	-2.49112800	-3.18992500	2.22767000
H	-1.69970500	-3.31445800	4.53149100
C	0.34754100	-3.82464900	4.00335500
H	0.67721100	-3.88621400	5.03592000
C	1.21775500	-4.07628700	2.98515200
H	-6.55183600	1.76978300	-4.54178000
C	-6.13108200	2.50682400	-3.86458000
H	-6.15453000	1.18729900	-2.19905300
C	-5.90788700	2.18079100	-2.55901700
C	-5.29207400	4.74428500	-3.51530800
C	-5.34804900	3.12958300	-1.64319200
C	-5.81719400	3.80835400	-4.35487300
C	-5.04173000	4.44172500	-2.13934000
C	-5.10802300	2.82479700	-0.28697400
H	-6.00138600	4.04709100	-5.39794100
H	-4.30745700	6.40076500	-1.64683800
H	-5.05164500	5.74231000	-3.87314700
C	-4.55003800	3.79137200	0.57076800
C	-4.21550900	3.50656200	1.93573100
C	-4.26652200	5.10652600	0.06735300
H	-3.52449300	7.08333700	0.55955400
C	-4.52128600	5.40196300	-1.27022700
C	-3.67452000	4.46360000	2.74182500
H	-4.37598200	2.50268600	2.31337000
H	-3.41769400	4.22385000	3.76926500
C	-3.42927900	5.77961400	2.24861800
H	-3.00903500	6.53059000	2.91149800
C	-3.71408200	6.08666200	0.95210900
C	-5.17786600	-5.41863800	-2.00683600
H	-4.83224900	-5.38652900	-3.04928000

H	-4.83644700	-6.37798800	-1.60143200
C	-6.67486600	-2.95763100	-1.21046400
H	-6.83458300	-1.91551000	-1.50831100
H	-7.31212100	-3.11776600	-0.33036100
C	-7.13181900	-3.90706700	-2.31710800
H	-8.21826700	-3.83751600	-2.43161900
H	-6.68551800	-3.60635700	-3.27399800
C	-6.70153200	-5.33342700	-1.98838300
H	-7.12344900	-6.04941200	-2.70091900
H	-7.07940400	-5.60491400	-0.99350800
C	-6.49120500	-2.30537500	2.11595200
H	-6.34248800	-3.17202500	1.46934400
H	-5.71857500	-2.38589500	2.89531900
C	-8.07807500	0.13698100	2.71912400
H	-8.06097000	1.05325500	3.31929000
H	-9.01865500	0.16148900	2.15096500
C	-7.86361700	-2.35361600	2.78288600
H	-8.64745300	-2.42036800	2.01566600
H	-7.94042100	-3.25341100	3.40207200
C	-8.07942100	-1.09578100	3.62005200
H	-7.27254300	-1.01563100	4.36019300
H	-9.02189900	-1.14897700	4.17483100

Zero-point correction= 1.387067 (Hartree/Particle)

Thermal correction to Energy= 1.444898

Thermal correction to Enthalpy= 1.445700

Thermal correction to Gibbs Free Energy= 1.297486

Sum of electronic and zero-point Energies= -7001.625080

Sum of electronic and thermal Energies= -7001.567249

Sum of electronic and thermal Enthalpies= -7001.566448

Sum of electronic and thermal Free Energies= -7001.714662

E(RM062X/6-311+G(2d,p)) = -7006.57145654
